# Enantioconvergent Access to Chiral S(VI) Stereocenters by Kinetic Resolution of Sulfonimidoyl Chlorides

**DOI:** 10.1002/anie.202519733

**Published:** 2025-11-17

**Authors:** Arko Das, Shree Krishna Dhakal, Ramon Trevino, Seth O. Fremin, Vy T. B. Nguyen, Arka Porey, Sachchida Nand, Chandan Kumar Giri, Daniel J. Wherritt, Hadi D. Arman, Oleg V. Larionov

**Affiliations:** ^1^ Department of Chemistry The University of Texas at San Antonio One UTSA Circle San Antonio TX 78249 USA

**Keywords:** Asymmetric catalysis, Kinetic resolution, Sulfonimidates, Sulfonimidoyl chlorides, Sulfur chirality

## Abstract

Sulfur chirality has recently gained in importance across a wide range of chemical domains. However, the dearth of catalytic approaches for sulfur(VI) stereocenters continues to present a challenge for the broader utilization of chiral‐at‐sulfur motifs. We report herein the development of a previously unexplored approach for the kinetic resolution of chiral sulfur(VI) functionalities that leverages the electrophilic reactivity of sulfur(VI) stereocenters. This catalytic strategy provides enantioconvergent access to sulfonimidoyl chlorides and sulfonimidates, which are some of the least explored and catalytically accessible stereogenic S(VI) functionalities. This study also elucidates the key roles of noncovalent interactions and highlights the importance of the unfunctionalized backbone of the catalyst in imparting high enantioselectivity to the kinetic resolution of heteroatom stereocenters.

## Graphical abstract

An enantioconvergent approach for the kinetic resolution of S(VI) stereocenters that leverages the electrophilic reactivity of the chiral sulfur atom is described. The catalytic process provides access to sulfonimidoyl chlorides and sulfonimidates, which are among the least studied S(VI) functionalities. Mechanistic studies reveal key roles of substrate interactions with the unfunctionalized catalyst backbone in the enantioselective process.

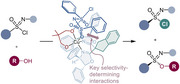

## Introduction

**Figure 1:**
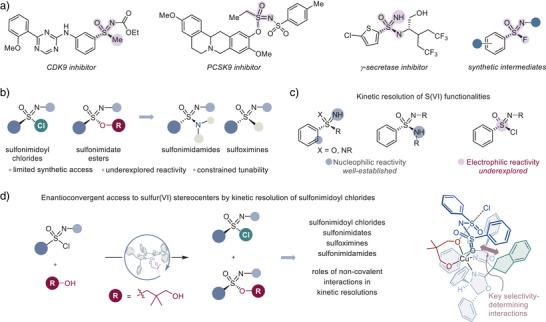
Kinetic resolution of sulfonimidoyl chlorides. a). Applications of chiral S(VI) functionalities. b). Sulfonimidoyl chlorides and sulfonimidate esters. c). Approaches for the kinetic resolution of S(VI) stereogenic centers. d). Kinetic resolution of sulfonimidoyl chlorides enabled by the copper‐catalyzed nucleophilic substitution at the S(VI) stereocenter.

Sulfur(VI) stereocenters have recently emerged at the forefront of drug discovery, agrochemistry, asymmetric catalysis, and supramolecular and macromolecular applications.^[^ ^1^, ^2^, ^3^, ^4^, ^5^, ^6^, ^7^, ^8^, ^9^, ^10^, ^11^, ^12^, ^13^, ^14^ ^]^ S(VI) stereogenic functional groups, such as sulfonimidamides, sulfoximines, sulfonimidoyl halides, and sulfonimidates, exhibit a diverse range of physicochemical properties and reactivities, which can be systematically fine‐tuned by modifying the substitution pattern around the stereogenic sulfur atom (Figure 1a). Furthermore, chiral S(VI) functionalities, such as sulfonimidamides and sulfoximines, can offer distinct advantages over well‐established sulfonamides and sulfones, including increased polarity and solubility, and additional molecular vectors for noncovalent interactions.^[^ ^4^, ^5^, ^7^, ^8^ ^]^ However, the limited synthetic accessibility of the various chiral S(VI) functionalities remains a major obstacle to the broader adoption of S(VI) stereocenters.^[^ ^3^, ^4^, ^7^, ^8^, ^9^ ^]^ Furthermore, their divergent reactivities pose a challenge to the development of generalizable synthetic strategies that enable the construction of diverse chiral S(VI) functionalities from readily accessible S(VI) precursors.^[^ ^15^, ^16^, ^17^, ^18^, ^19^, ^20^, ^21^, ^22^, ^23^, ^24^, ^25^, ^26^, ^27^, ^28^, ^29^, ^30^, ^31^, ^32^, ^33^, ^34^ ^]^ As a result, most synthetic strategies to S(VI) stereocenters rely on the oxidative conversion of chiral S(IV) compounds. Although substantial progress in the development of new approaches to chiral S(IV) compounds has recently been achieved, the synthetic availability and structural diversity of chiral S(IV) precursors continue to pose significant challenges.^[^ ^35^, ^36^, ^37^, ^38^, ^39^, ^40^, ^41^, ^42^, ^43^, ^44^, ^45^, ^46^, ^47^, ^48^, ^49^, ^50^, ^51^, ^52^, ^53^ ^]^ Additionally, the scope of methods for oxidative conversion of S(IV) to S(VI) functionalities also remains underdeveloped.^[^ ^3^, ^7^, ^8^, ^9^, ^15^ ^]^

Sulfonimidoyl chlorides and sulfonimidates are among the least explored chiral S(VI) functionalities. First, synthesized in the enantioenriched form from sulfinamides by Johnson in 1971,^[^ ^54^ ^]^ sulfonimidoyl chlorides can be converted to other chiral S(VI) functionalities by reactions with nucleophiles (Figure 1b).^[^ ^28^, ^54^ ^]^ Sulfonimidoyl chlorides bearing electron‐withdrawing groups on the nitrogen atom are stable at ambient temperature and can be handled without any special precautions. Although this versatility can be advantageous for the late‐stage diversification of chemical libraries and the generation of neglected S(VI) pharmacophores, asymmetric synthesis of sulfonimidoyl chlorides continues to be challenging.

The synthetic strategies for the introduction of sulfonimidate groups into organic molecules have similarly been largely confined to the oxidative conversion of chiral S(IV) precursors.^[^ ^55^, ^56^, ^57^, ^58^, ^59^, ^60^ ^]^ Despite these challenges, recent applications of sulfonimidates as chiral S(VI) electrophiles and biological probes underscore their untapped potential in synthetic and medicinal chemistry.^[^ ^55^, ^56^, ^57^, ^58^, ^59^, ^60^, ^61^, ^62^ ^]^ Accordingly, the development of catalytic asymmetric approaches for sulfonimidoyl chlorides and sulfonimidates has recently gained attention in the chemistry of stereogenic sulfur functionalities,^[^ ^28^, ^34^ ^]^ yet persistent limitations in the structural diversity and synthetic accessibility remain largely unresolved.

To this end, we posited that the electrophilic reactivity of sulfonimidoyl chlorides could be leveraged for the development of a kinetic resolution reaction via a catalytic stereospecific S_N_2 substitution at the stereogenic sulfur atom with an appropriate alcohol nucleophile. This unprecedented approach would enable enantioconvergent access to chiral sulfonimidoyl chlorides and sulfonimidates, which could also be readily converted to other chiral S(VI) functionalities. Furthermore, it would provide insight into catalytic systems, which could promote kinetic resolution of electrophilic S(VI) stereocenters.

Although this strategy has not been experimentally validated, recent studies, facilitated by the nucleophilic reactivity of sulfoximines, sulfondiimides, and sulfonimidamides, have highlighted the synthetic potential of kinetic resolution reactions in chiral S(VI) settings (Figure 1c).^[^ ^19^, ^23^, ^24^, ^25^, ^26^, ^27^, ^30^ ^]^ For example, the organocatalytic acylation and alkylation of the nucleophilic nitrogen atom in sulfoximines and sulfonimidamides was employed by Bolm,^[^ ^19^ ^]^ Willis,^[^ ^25^ ^]^ and Biju.^[^ ^30^ ^]^ On the other hand, several approaches based on the transition metal‐catalyzed C─H functionalization of the *ortho* position in sulfoximines were developed by Cramer,^[^ ^23^ ^]^ Shi,^[^ ^24^, ^27^ ^]^ and Sahoo and Gandon.^[^ ^26^ ^]^ Additionally, elegant and mechanistically distinct stereoablative approaches based on sulfonimidoyl chlorides have recently been disclosed by Jiang^[^ ^33^ ^]^ and Wu.^[^ ^34^ ^]^ We report herein the development of a previously unexplored kinetic resolution reaction of sulfonimidoyl chlorides that enables synthetic access to a variety of chiral S(VI) functionalities, including sulfonimidates, sulfonimidamides, and sulfoximines (Figure 1d). This study provides a blueprint for the exploration of asymmetric catalytic approaches that are based on the electrophilic reactivity of S(VI) stereocenters. It also reveals the steering effect of stabilizing interactions between the substrate and the unfunctionalized backbone of the chiral catalyst, which can guide the development of other kinetic resolution approaches for heteroatom chirality.

## Results and Discussion

Initial optimization studies revealed that the kinetic resolution of sulfonimidoyl chloride *rac*‐**1a** can be accomplished with diol **2** (Table 1). The selection of diol **2** was based on the premise that its chelating ability and the Thorpe–Ingold effect of the *gem*‐dimethyl moiety^[^ ^63^ ^]^ can facilitate complexation with a metal catalyst. Under optimal conditions, the reaction was catalyzed by copper(II) triflate and bisoxazoline ligand **L1** (entry 1, Table 1) with silver carbonate as the base and dichloromethane as the solvent. Sulfonimidate ester **3a** and sulfonimidoyl chloride **1a** were produced with 91% and 96% ee, pointing to an efficient kinetic resolution process (*s* = 83). Catalysts based on other copper(I) and copper(II) salts (entries 2 and 3) and other metals (entries 4 and 5) demonstrated inferior catalytic activity. Other solvents and basic reagents also afforded products **3a** and **1a** with lower enantioselectivities and yields (entries 6–9). Notably, an evaluation of a wide range of alcohols and diols revealed that the use of diol **2** was crucial for achieving high selectivity (entries 10 and 11). Ligand selection was similarly important for achieving high enantioselectivity and efficiency. Structural adjustments in the optimal *trans*‐diphenyloxazoline fragment resulted in diminished selectivity (**L2**). Notably, the indane backbone was critical for achieving high enantioselectivity (**L3**), and more drastic changes in the ligand structure proved broadly detrimental to the reaction performance (**L4**‐**L6**). Furthermore, no formation of S(IV) by‐products was observed under the Lewis acid‐catalyzed reaction conditions. This result contrasts with the facile formation of S(IV) products in Lewis base‐catalyzed reactions with alcohol nucleophiles recently reported by Wu,^[^ ^52^ ^]^ underscoring the synthetic versatility of sulfonimidoyl chlorides.

**Table 1:** Kinetic resolution of sulfonimidoyl chlorides ^a)^

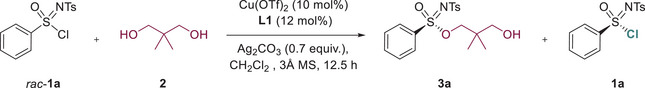						
		1a		3a		
Entry	Variation from standard conditions	Yield, % ^b)^	ee, % ^c)^	Yield, % ^b)^	ee, % ^c)^	*s*
1^d)^	None	52	96	43	91	83
2	With CuI	43	12	10	53	3
3	With CuCl_2_	94	3	3	84	12
4	With Fe(OTf)_2_	90	2	3	46	3
5	With Co(OTf)_2_	82	14	13	89	20
6	In EtOAc	58	32	38	65	6
7	In toluene	62	36	30	80	13
8	With K_2_CO_3_	68	24	17	94	41
9	With Cs_2_CO_3_	85	8	10	72	7
10	With ethylene glycol	73	2	23	2	1
11	With benzyl alcohol	98	1	0	0	–

^a)^ Reaction conditions: sulfonimidoyl chloride *rac*‐**1a** (0.1 mmol), diol **2** (0.17 mmol), Cu(OTf)_2_ (10 mol%), **L1** (12 mol%), 3 Å molecular sieves (MS) (40 mg), Ag_2_CO_3_ (0.07 mmol), CH_2_Cl_2_ (1 mL), 12.5 h.

^b)^ Yields were determined by ^1^H NMR with 1,3,5‐trimethoxybenzene as an internal standard.

^c)^ ee values were determined by HPLC on a chiral stationary phase.

^d)^ Yields and ee are reported for isolated **1a** and **3a**. *s*, selectivity factor; *s* = ln[(1–*c*)(1–ee**_1a_**)]/ln[(1–*c*)(1 + ee**_1a_**)]; *c*, conversion; *c* = ee**_1a_**/(ee**_1a_** + ee**_3a_**). Ts, *p*‐toluenesulfonyl; Tf, trifluoromethanesulfonyl.
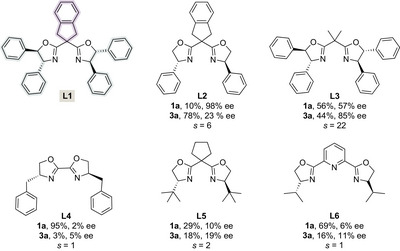

**Figure 2:**
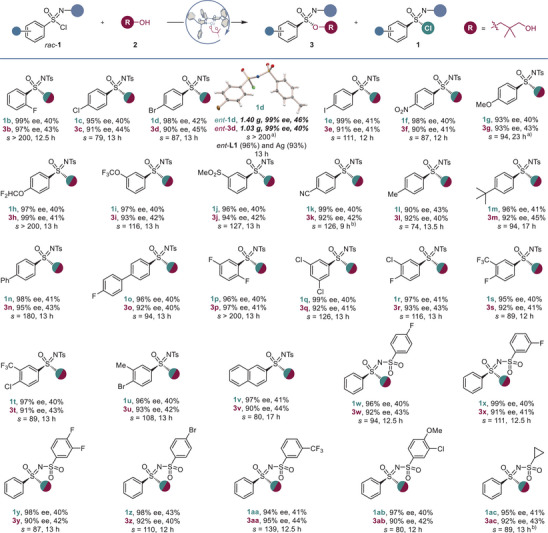
Scope of the kinetic resolution of sulfonimidoyl chlorides. The yields and ee are reported for the isolated products. Reaction conditions: sulfonimidoyl chloride (0.1 mmol), diol **2** (0.17 mmol), Cu(OTf)_2_ (10 mol%), **L1** (12 mol%), 3 Å molecular sieves (40 mg), Ag_2_CO_3_ (0.03–0.09 mmol), CH_2_Cl_2_ (1 mL), 9–23 h. ^a)^ The preparative scale synthesis was carried out with *ent*‐**L1**. ^a)^ 30 °C. ^b)^ 0 °C.

With the optimal conditions established, we next explored the substrate scope of the kinetic resolution reaction (Figure 2). Substrates with various halogen substituents readily produced corresponding sulfonimidate esters **3b**‐**3e** with 90%–99% ee and sulfonimidoyl chlorides **1b**‐**1e** with 95%–99% ee (*s *= 79 – >200^[^ ^64^ ^]^). The nitro group was also well tolerated (90% ee for **3f** and 98% ee for **1f**; *s* = 87). High selectivity (*s* = 94 – >200) was also observed for the substrates with pendant methoxy and di‐ and trifluoromethoxy groups (**3g‐3i**, 93%–99% ee, and **1g**‐**1i**, 93%–97% ee). Sulfone and cyano groups were likewise compatible with the developed method (**3j** and **3k,** and **1j** and **1k**). Furthermore, substrates with diverse alkyl and aryl groups afforded sulfonimidate esters **3l**‐**3o** and recovered sulfonimidoyl chlorides **1l**–**1o** with high stereoinduction (*s* factors up to 180). The reaction also demonstrated excellent resolution performance, with *s* values in the range of 89 – >200 for sulfonimidoyl chlorides with various combinations of halogen, alkyl, and trifluoromethyl groups (**3p**‐**3u** and **1p**‐**1u**). High enantioselectivity (*s* = 80) was also observed for naphthalene‐derived sulfonimidoyl chloride *rac*‐**1v**. Substrates with diverse substituents in the *N*‐sulfonyl residue were also amenable to efficient kinetic resolution. Halogen substituents in the *N*‐sulfonyl residue were well tolerated (**3w‐3z** and **1w‐1z**) and provided *s* factors of up to 111. Sulfonimidoyl chlorides bearing trifluoromethyl and methoxy groups were equally compatible (**3aa** and **3ab**, and **1aa** and **1ab**). Finally, the substrate *rac*‐**1ac** displaying an aliphatic *N*‐sulfonyl group was also tested, and sulfonimidate ester **3ac** and recovered sulfonimidoyl chloride **1ac** were afforded with 92% and 95% ee. X‐ray crystallographic analysis of sulfonimidoyl chloride **1d** (Figure 2), as well as *ent*‐**1b** and *ent*‐**1g** and sulfonimidate *ent*‐**3x** obtained with *ent*‐**L1** (see the Supporting Information) confirmed the absolute configurations of the kinetic resolution products. Furthermore, the developed method can be used to access gram quantities of the enantioenriched sulfonimidate and sulfonimidoyl chloride products (*ent*‐**3d** and *ent*‐**1d**) with concomitant ligand and silver(I) recovery. Lastly, in contrast to the high selectivity observed in the reactions of sulfonimidoyl chlorides **1a‐1ac**, no kinetic resolution was observed when the aromatic group on the sulfur was replaced with an alkyl group or when the sulfonyl group on the nitrogen atom was replaced with the benzoyl group.

**Figure 3:**
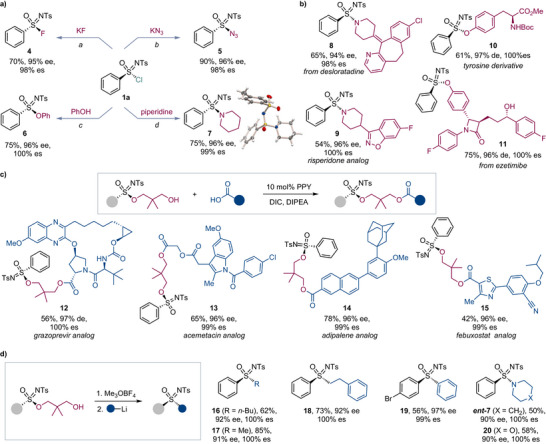
Synthetic applications of sulfonimidoyl chlorides and mechanistic studies of the kinetic resolution reaction. a). Conversion to synthetically and medicinally important chiral sulfur(VI) functionalities. Reaction conditions: *a*. KF (1 equiv.), dibenzo‐18‐crown‐6 (2 equiv.), PhCF_3_/cyclohexane (2: 1); *b*. KN_3_, THF; *c*. phenol (1.1 equiv.), Cs_2_CO_3_ (1.1 equiv.), 1,2‐dichloroethane; *d*. piperidine (1.1 equiv.), Na_2_CO_3_ (1.1 equiv.), CH_2_Cl_2_. b). Construction of sulfonimidate and sulfonimidamide derivatives of drugs and natural products from *ent*‐**1a** obtained with *ent*‐**L1**. c). Sulfonimidate tagging of drugs. Reaction conditions: carboxylic acid (1.5 equiv.), diisopropylethylamine (DIPEA, 2 equiv.), diisopropylcarbodiimide (DIC, 2 equiv.), 4‐pyrrolidinopyridine (PPY, 10 mol%), CH_2_Cl_2_. d). Construction of sulfoximines and sulfonimidamides from sulfonimidates **3**. Reaction conditions: *a*. Me_3_OBF_4_, (2 equiv.), proton sponge (1.5 equiv.), CH_2_Cl_2_, 10 °C. *b*. RLi, Et_2_O, –78 °C (for **16–18**, **ent‐7**, and **20**) or –100 °C (for **19**).

We next determined whether the developed kinetic resolution method could facilitate synthetic access to other valuable chiral sulfur functionalities by stereospecific substitution reactions (Figure 3a). Sulfonimidoyl fluoride **4** could be readily produced from **1a** with high enantiospecificity in a reaction with potassium fluoride in conjunction with a crown ether and in a nonpolar medium to suppress racemization. The substitution of chloride with azide likewise proceeded with excellent enantiospecificity (**5**). Furthermore, the sulfonimidoyl group could be efficiently transferred onto the oxygen atom in phenol, thereby producing aryl sulfonimidate **6**. Similarly, high stereochemical fidelity was observed in a reaction with an amine that produced sulfonimidamide **7**. Stereospecific sulfonimidoyl transfer could also be readily accomplished in the more complex structural settings of drugs and natural products following the synthetic procedures developed for compounds **6** and **7** (Figure 3b), thus yielding sulfonimidamide analogs of the antihistamine desloratadine (**8**) and the antipsychotic risperidone (**9**) and sulfonimidate analogs of tyrosine (**10**) and ezetimibe (**11**). Furthermore, the hydroxy group in products **3** can be used to introduce sulfonimidate residues into bioactive molecules (Figure 3c). To that end, carbodiimide‐mediated esterification enabled the construction of a sulfonimidate‐containing analog of the hepatitis C drug grazoprevir **12**. Sulfonimidate analogs of the anti‐inflammatory and retinoid drugs acemetacin (**13**) and adapalene (**14**) were likewise readily synthesized. Lastly, an analog of the xanthine oxidase inhibitor febuxostat (**15**) was also accessed with high stereochemical fidelity.

Sulfonimidates **3** can also be readily converted to sulfoximines **16**–**19** (Figure 3d) via a trimethyloxonium salt‐mediated methylation of the hydroxy group, which is followed by a stereospecific substitution reaction with an organolithium reagent. Sulfonimidamides ***ent*‐7** and **20** could also be prepared using the same procedure with the corresponding lithium amides as nucleophiles. Collectively, these results indicate that the developed kinetic resolution method can serve as a general synthetic platform for the construction of a variety of chiral sulfur(VI) functional groups via nucleophilic substitution at the sulfur stereocenter in both the sulfonimidate products and the recovered sulfonimidoyl chlorides with high stereochemical fidelity. Furthermore, the enantioconvergent approach enables the efficient generation of libraries of diverse stereogenic S(VI) functionalities from a single racemic precursor, such as sulfonimidates, sulfonimidamides, and sulfoximines **8–11**, **16–20**, from *rac*‐**1a**.

Prompted by the observed high enantioselectivity of the kinetic resolution reaction of sulfonimidoyl chlorides, we set out to elucidate the mechanistic details of the catalytic process.

**Figure 4:**
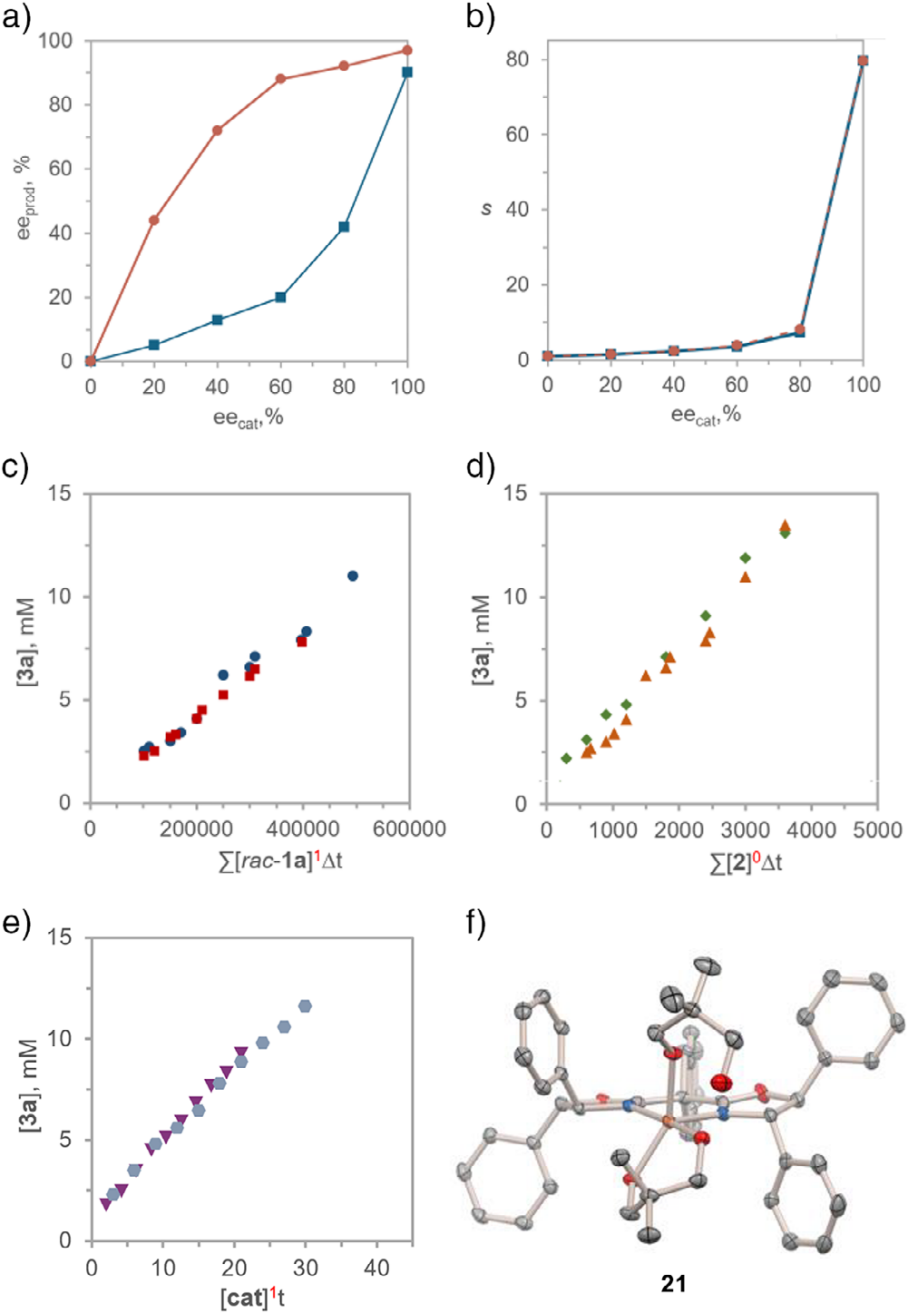
Mechanistic studies of the kinetic resolution reaction. a). Dependence of the ee of sulfonimidate **3a** (●) and recovered sulfonimidoyl chloride **1a** (■) (ee_prod_) on the ee of the catalyst (ee_cat_). b). Dependence of the experimental (■) and theoretical (●) selectivity factors *s* on the ee of the catalyst. The experimental *s* values were calculated as shown in Table 1. The theoretical *s* values were calculated as a function of ee_cat_: *s* = [*s“*(1 + ee_cat_)–(1–ee_cat_)]/[(1 + ee_cat_) + *s”*(1–ee_cat_)] for *s'* = 83 obtained with enantiomerically pure **L1**. c). Variable time normalization analysis (VTNA) for sulfonimidoyl chloride *rac*‐**1a** (■, 0.08 M; ●, 0.1 M). d). VTNA for diol **2** (▲, 0.17 M; ◆ 0.25 M). e). VTNA for the catalyst (▼, 7 mol%; ●, 10 mol%). f). X‐ray crystal structure of the catalyst–diol adduct Cu(*ent*‐**L1**)(*κ* ^2^O‐**2**)(*κ* ^1^O‐**2**)[OTf]_2_, **21**. Hydrogen atoms and triflate anions are omitted for clarity.

To investigate the nature of the catalyst speciation, the influence of the enantiomeric purity of the catalyst on the selectivity of the kinetic resolution was first examined. In asymmetric catalysis, the dependence of the product ee on the enantiopurity of the catalyst is linear in the absence of higher order effects, whereas deviations from linearity (nonlinear effects, NLE) indicate catalyst aggregation or the involvement of multiple catalyst species.^[^ ^65^ ^]^ In contrast, normal catalytic behaviors in kinetic resolution exhibit intrinsically nonlinear dependence of the ee values of the product and recovered reactant on ee_cat_. Therefore, higher‐order effects are indicated by a deviation from the theoretically derived hyperbolic dependence of the experimental stereoselectivity factor *s* on the catalyst enantiopurity.^[^ ^66^ ^]^ In line with these considerations, both the ee of product **3a** and that of recovered sulfonimidoyl chloride **1a** were influenced by the catalyst enantiopurity (Figure 4a). Furthermore, the experimental stereoselectivity factor *s* demonstrated an excellent fit with the theoretical hyperbolic curve (Figure 4b) that was derived for the normal behavior of a catalytic system with the experimentally observed intrinsic stereoselectivity factor (*s* = 83) for the enantiomerically pure catalyst (See page S4 in the Supporting Information for further details). These results indicate that the catalytically active species contains only one molecule of the Cu/**L1** complex. Furthermore, these observations are consistent with the results of the variable time normalization analysis (VTNA)^[^ ^67^ ^]^ (Figure 4c–e), which revealed that the reaction is first order for the Cu catalyst and sulfonimidoyl chloride *rac*‐**1a**. and zero‐order for diol **2**.

The nature of the catalyst in the resting state was next studied by means of diffusion‐ordered nuclear magnetic resonance spectroscopy (DOSY NMR).^[^ ^68^ ^]^ This method can reveal the complexation behavior of solutes by experimentally correlating their diffusion coefficients and molecular weights. The experimentally determined molecular weight of the Cu/**L1**‐containing species (MW = 851.8, ±2.3%) was consistent with that of a 1:2 (Cu/**L1**)–diol **2** complex (MW = 832.5).

### The Structural Identity of the Catalytically Active Species was Further Probed by X‐Ray Crystallographic Analysis

Crystallographic studies revealed a (Cu/*ent*‐**L1**)–diol **2** complex with a 1: 2 Cu/diol **2** stoichiometry (**21**, Figure 4f), in accordance with the solution‐phase DOSY NMR data. Complex **21** features a copper center that is chelated by diol **2** and *ent*‐**L1**, with the second loosely bound (*d*_Cu–O_ = 2.18 Å) monocoordinated diol **2** ligand occupying the apical position.

**Figure 5:**
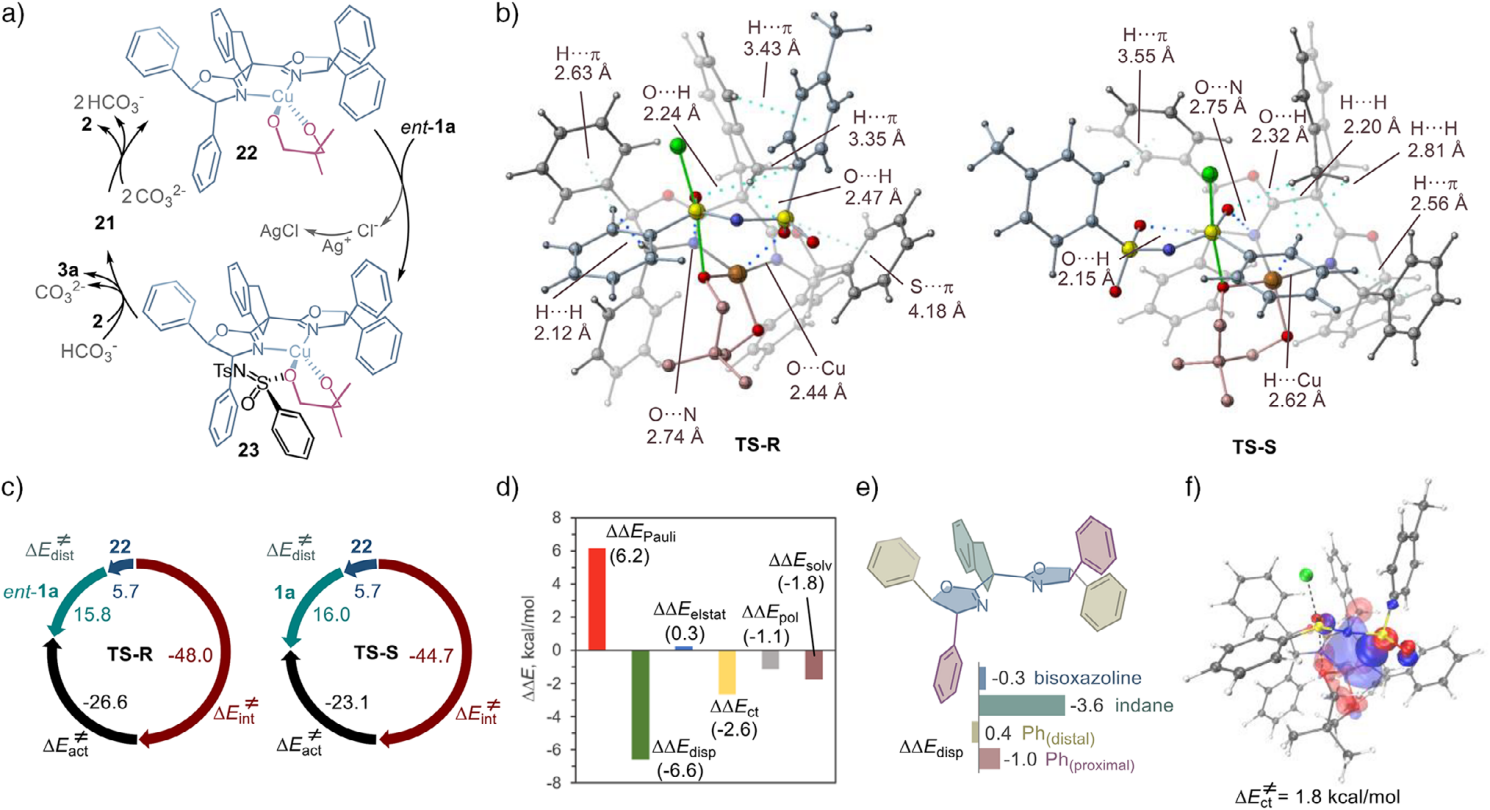
Mechanistic computational studies of the kinetic resolution reaction of sulfonimidoyl chlorides. a). Catalytic cycle of kinetic resolution. b). Transition state structures **TS‐R** and **TS‐S** for the Cu‐catalyzed nucleophilic displacement process. c). Distortion–interaction analysis of transition state structures **TS‐R** and **TS‐S**, Δ*E*, kcal mol^−1^. d). Energy decomposition analysis of transition state structures **TS‐R** and **TS‐S**, ΔΔ*E^‡^* = Δ*E^‡^* **_TS‐R_**–Δ*E^‡^* **_TS‐S_**, kcal mol^−1^. e). Contribution of the individual **L1** fragments to the dispersion component of the substrate–catalyst interaction in **TS‐R** and **TS‐S**, ΔΔ*E^‡^*_disp_ = Δ*E^‡^*_disp(_ **_TS‐R_**_)_–Δ*E^‡^*_disp(_ **_TS‐S_**_)_, kcal mol^−1^. f). Complementary occupied–virtual pairs (COVPs) in the *β* space for the *n*_O(_ *_ent_*_‐_ **_1a_**_)_→σ*_22_ interaction in **TS‐R**.

Taken together, the results of these experimental studies are consistent with a mechanism that involves the formation of complex **21** that, upon dissociation of the apical *κ* ^1^O‐diol **2** and subsequent deprotonation with the base, can produce Cu(II) dialkoxide intermediate **22** (Figure 5a). The subsequent stereoselective reaction favors *ent*‐**1a** and affords Cu‐bound sulfonimidate **23**. Product **3a** is then released via ligand exchange with diol **2**. The byproduct chloride is sequestered by Ag^I^, which prevents deactivation of the dicationic Cu^II^ catalyst by the negatively charged halide ligand.

Density functional theory (DFT) analysis was carried out next to gain insight into the origin of the enantioselectivity in the Cu‐catalyzed nucleophilic displacement reaction (Figures 5b–f and S9–S19). The reaction of complex **22** with the racemic sulfonimidoyl chloride substrate proceeds over accessible barriers and favors **TS‐R** (Δ*G^‡^* = 10.5 kcal mol^−1^ for **TS‐R** and 13.5 kcal mol^−1^ for **TS‐S**; ΔΔ*G^‡^* = 3.0 kcal mol^−1^; Figure 5b). The stereochemical outcome of the pathway that traverses the more favorable transition state structure **TS‐R** produces sulfonimidate **3a** from sulfonimidoyl chloride *ent*‐**1a**, which can result in the accumulation of less reactive enantiomer **1a**, in accordance with the experimentally observed outcome of the kinetic resolution process.

Furthermore, distortion–interaction activation strain model (ASM) analysis^[^ ^69^ ^]^ revealed that both **TS‐R** and **TS‐S** exhibit greater distortion in the substrate fragment caused by structural adjustment at the sulfur center, which is required to accommodate the incoming nucleophile (Figure 5c). However, the analysis also indicated that both transition state structures exhibit similar levels of distortion and that the enantioselectivity is largely driven by the substantially stronger interactions in **TS‐R**.

Energy decomposition analysis (EDA)^[^ ^70^ ^]^ confirmed that **TS‐R** benefits from stronger attractive interactions, particularly dispersion and charge transfer (orbital interactions between the fragments), which compensate for greater Pauli (steric) repulsion (Figure 5d).

Analysis of noncovalent interactions (NCIs) using the independent gradient model based on Hirshfeld partition (IGMH)^[^ ^71^ ^]^ was performed next to clarify the origin of these interactions and the roles of the **L1** *trans*‐diphenyloxazoline and indane moieties in the preferential stabilization of **TS‐R** (Figure 5b). In broad agreement with the results of EDA, NCI analysis revealed that **TS‐R** benefits from more extended stabilizing dispersion interactions between the sulfonyl residue of the substrate and both the *trans*‐diphenyloxazoline and the indane fragments of the ligand. *trans*‐Diphenyloxazoline fragment is also engaged in stabilizing H···*π* interactions with the aryl group in the sulfonimidoyl residue of the substrate. These stronger interactions compensate for the Pauli repulsion between the α‐C─H in the oxazoline moiety and the arenesulfonimidoyl residue in **TS‐R**, as revealed by EDA. Additionally, an analysis of the contributions of individual fragments in ligand **L1** (proximal and distal phenyl rings and indane and bisoxazoline residues) to the dispersion interactions with the substrate in **TS‐R** and **TS‐S** indicated that the indane moiety provided a decisive contribution to the stabilization of **TS‐R** (Figure 5e). Furthermore, in line with the results of EDA, an analysis of complementary occupied–virtual pairs (COVPs)^[^ ^72^ ^]^ also revealed that **TS‐R** is stabilized by stronger charge transfer interactions. In particular, **TS‐R** benefits from an *n*_O(_ *_ent_*_‐_ **_1a_**_)_→σ*_22_ interaction between the oxygen lone pair in the sulfonyl group of the substrate and a σ* for the Cu─N/O_(_ **_22)_** and the proximal C─H bond in the indane methylene group, which was not observed in **TS‐S** (Figure 5f).

Lastly, effective oxidation state (EOS)^[^ ^73^ ^]^ analysis indicated that both complex **22** and transition state **TS‐R** feature a Cu^II^ metal center, which rules out the involvement of metal‐mediated single electron transfer processes.

Collectively, the results of these studies point to an intricate synergy of dispersion and charge transfer (orbital) effects in imparting high enantioselectivity via the *trans*‐diphenyloxazoline and indane moieties of the Cu/**L1** catalyst. Furthermore, they underscore the importance of substrate interactions with remote and unfunctionalized structural elements of a chiral catalyst for achieving efficient enantiocontrol in kinetic resolution reactions.

## Conclusion

In summary, we developed a previously unexplored catalytic kinetic resolution approach for sulfonimidoyl chlorides that leverages the electrophilic reactivity of S(VI) stereocenters.^[^ ^74^ ^]^ The approach provides a blueprint for the expansion of kinetic resolution strategies to S(VI) chirality via mechanistically distinct transformations that complement the well‐established nucleophilic reactivity‐based approaches to sulfoximines and sulfonimidamides. Furthermore, the method provides catalytic access to synthetically challenging sulfonimidate esters that have been conventionally generated by the oxidative conversion of chiral S(IV) precursors. Additionally, experimental studies revealed the structural effects and mechanistic details of the copper‐catalyzed asymmetric process. Further mechanistic insight was gleaned from computational investigations of the key catalytic nucleophilic displacement step. These studies revealed the critical role of noncovalent interactions between the substrate and the unfunctionalized backbone of the chiral ligand in achieving the experimentally observed high enantioselectivity. Taken together, these studies suggest future avenues for the expansion of nascent kinetic resolution strategies to heteroatom chirality by harnessing mechanistically unexplored reactivity in well‐defined molecular settings.

## Conflict of Interests

The authors declare no conflict of interest.

## Supporting information



Supporting Information

Supporting Information

## Data Availability

The data supporting the findings of this study are available in the Supporting Information for this article.
