# Supplementary material for: Enantioconvergent Access to Chiral S(VI) Stereocenters by Kinetic Resolution of Sulfonimidoyl Chlorides
Source: Angew Chem Int Ed Engl. 2025 Nov 17;65(2):e19733. doi: 10.1002/anie.202519733 (PMC12790358; doi:10.1002/anie.202519733)
Supplement: Supplementary file 1 — Supporting Information [file ANIE-65-e19733-s002.pdf]

Supporting Information

Enantioconvergent Access to Chiral S(VI) Stereocenters by Kinetic Resolution of Sulfonimidoyl Chlorides

Arko Das,¶ Shree Krishna Dhakal,¶ Ramon Trevino,¶ Seth O. Fremin, Vy T. B. Nguyen, Arka Porey, Sachchida Nand, Chandan Kumar Giri, Daniel J. Wherritt, Hadi D. Arman, and Oleg V. Larionov*

Department of Chemistry, The University of Texas at San Antonio, San Antonio, Texas 78249, United States
oleg.larionov@utsa.edu

Contents

Materials and experimental details S1

General procedures S2

Additional experimental studies S3

Synthesis of sulfonimidoyl chlorides S9

Kinetic resolution products S18

Computational data S48

X-ray crystallographic data S64

NMR spectroscopic data S70

HPLC data S222

References S298

Materials and experimental details

Materials: Anhydrous dichloromethane and toluene were collected under argon from an LC Technologies solvent purification system, having been passed through two columns packed with molecular sieves. Anhydrous chloramine-T was dried in vacuo for 3 h at 80 °C before use. All other commercially available reagents were used without further purification.

Purification: Column chromatography was performed using CombiFlash Rf-200 (Teledyne-Isco) automated flash chromatography system as well as manually. Preparative thin-layer chromatography was carried out on silica gel-coated (UV254) glass plates. Thin-layer chromatography was carried out on silica gel-coated glass plates (Merck Kieselgel 60 F254). Plates were visualized under ultraviolet light (254 nm) and using a potassium permanganate stain.

Characterization: ^{1}H, ^{13}C and ^{19}F NMR spectra were recorded at 500 MHz (^{1}H), 125 MHz (^{13}C) and 470.5 MHz (^{19}F) on Bruker AVANCE III 500 instruments in $CDCl_3$ or other specified deuterated solvents with and without tetramethylsilane (TMS) as an internal standard at 25 °C, unless specified otherwise. Chemical shifts (δ) are reported in parts per million (ppm) from tetramethylsilane (^{1}H and ^{13}C) and $CFCl_3$ (^{19}F). Coupling constants (J) are in Hz. Proton multiplicity is assigned using the following abbreviations: singlet (s), doublet (d), triplet (t), quartet (q), quintet (quint.), septet (sept.), heptet (hept.), multiplet (m), broad (br).

Infrared measurements were carried out neat on a Bruker Vector 22 FT-IR spectrometer fitted with a Specac diamond attenuated total reflectance (ATR) module.

¶ These authors contributed equally

High performance liquid chromatography was performed on Agilent 1260 Infinity Series HPLC, using IBN, IC, IH, IF, IG, IJ chiral columns with a mixture of hexane and isopropanol as an eluent. Optical rotation was measured on a Anton Paar MCP100 instrument.

General Procedures

General procedure for the conversion of sulfonyl chlorides to sulfonimidoyl chlorides (GP1)

To an oven dried pressure tube equipped with a magnetic stir bar were added sodium sulfite (8.0 mmol, 1.6 equiv.), sodium bicarbonate (8.0 mmol, 1.6 equiv.), the corresponding aryl sulfonyl chloride (5.0 mmol, 1.0 equiv.), water (10 mL) and dioxane (5 mL) under argon. The reaction mixture was stirred for 12 h at 110 °C. After cooling down to room temperature, the water and dioxane were removed under reduced pressure and dried by azeotropic concentration with benzene 3 times, backfilling the rotary evaporator with argon to prevent oxidation of the sulfinate product. The crude sodium sulfinate was purified by column chromatography (90 : 10 – 70 : 30 dichloromethane/methanol). The purified sodium sulfinate was added to an oven dried flask equipped with a magnetic stir bar and cooled to –30 °C. Thionyl chloride (3 equiv.) was added dropwise to the reaction mixture. The reaction mixture was allowed to warm to room temperature and stirred for 4 h. The resultant solid was filtered through a pad of anhydrous sodium sulfate and washed with diethyl ether. The filtrate was concentrated under reduced pressure to afford the corresponding sulfinyl chloride that was used for the reactions with chloramine-T without further purification. The chloramine-T was dried at 80 °C with simultaneous vacuum for 3 hours. The sulfinyl chloride (5 mmol, 1.0 equiv.) was added to an oven dried 75 mL pressure tube equipped with a magnetic stir bar followed by anhydrous chloramine-T (5 mmol, 1.0 equiv.), and toluene or dichloromethane (25 mL) under argon. The reaction mixture was stirred for 4 h at 85 °C. After cooling to room temperature, the reaction mixture was filtered through celite, and the celite pad was washed with toluene or dichloromethane (10 mL). The filtrate was collected, and the solvent was removed in vacuo. The corresponding sulfonimidoyl chloride was purified by column chromatography (hexane/ethyl acetate, 80 : 20 – 60 : 40).

General procedure for the conversion of sulfonyl chlorides and sulfonamides to sulfonimidoyl chlorides (GP2)

To a solution of sulfonyl chloride (5.0 mmol) in dichloromethane (10 mL) was added ammonium hydroxide (20 mL) dropwise at 0 °C and stirred for 1 h. Upon reaction completion (as monitored by TLC), the reaction was concentrated under reduced pressure and dried by azeotropic concentration with benzene (3 × 10 mL) to give a crude sulfonamide that was used directly without further purification for the next step. To a solution of sulfonamide (5 mmol, 1.0 equiv.) in methanol (15 mL) was added trichloroisocyanuric acid (TCCA, 0.33 equiv.). The reaction mixture was stirred at room temperature for 1 h, and the solvent was removed under reduced pressure. Toluene or dichloromethane (10 mL) was added, and the mixture was filtered through a celite pad that was washed with toluene or dichloromethane (10 mL). The filtrate was concentrated under reduced pressure, and to the resulting liquid was subsequently added methanol. The solution was then cooled to 0 °C and a solution of sodium methoxide (1.0 equiv.) in methanol (15 mL) was slowly added. The reaction was stirred at room temperature for 30 min. The solvent was removed under reduced pressure to afford the intermediate *N*-chlorosulfonamide that was transferred to an oven dried pressure tube equipped with a magnetic stirbar. Sulfinyl chloride (5 mmol, 1.0 equiv.) and toluene or dichloromethane (25 mL) were added under argon, and the reaction mixture was stirred for 4 h at 85 °C. After cooling to room temperature, the reaction mixture was filtered through a celite pad that was washed with toluene or dichloromethane (10 mL). The filtrate was concentrated under reduced pressure to give the corresponding sulfonimidoyl chloride that was purified by column chromatography (hexane/ethyl acetate, 80 : 20 – 60 : 40).

General procedure for the kinetic resolution of sulfonimidoyl chlorides (GP3)

To a 8 mL test tube equipped with a magnetic stir bar, ligand **L1** (6.7 mg, 0.012 mmol, 12 mol%), $Cu(OTf)_2$ (3.6 mg, 0.01 mmol, 10 mol%), 3Å molecular sieves (40 mg) and anhydrous dichloromethane (1 mL) were added in a glove-box. After stirring for 30 min, diol **2** (17.6 mg, 0.17 mmol, 1.7 equiv.), sulfonimidoyl chloride (0.1 mmol, 1.0 equiv.), and silver carbonate (0.3–0.8 equiv.) were added sequentially. The reaction tube was capped, and the reaction mixture

was stirred for 12–18 h at 25–27 °C. For work-up, a saturated solution of EDTA (1.5 mL) was added, followed by dichloromethane (5 mL). The reaction mixture was then extracted with dichloromethane (3 × 5 mL). The organic phases were combined, dried over anhydrous Na_2SO_4, concentrated under reduced pressure, and the remaining material was purified by preparative TLC (hexane/ethyl acetate, 80 : 20 – 50 : 50) to give the enantioenriched sulfonimidate product and sulfonimidoyl chloride.

General procedure for nucleophilic transformations of sulfonimidoyl chlorides (GP4)

To a 8 mL test tube equipped with a magnetic stir bar, sulfonimidoyl chloride **1** (0.1 mmol), Nucleophile (1.0 to 1.5 equiv.), and solvent (1 mL) were added in a glove-box. The reaction tube was capped, and the reaction mixture was stirred for the required time at 25–27 °C. For work-up, a saturated solution of brine (1.5 mL) was added, followed by dichloromethane (5 mL). The reaction mixture was then extracted with dichloromethane (3 × 5 mL). The organic phases were combined, dried over anhydrous Na_2SO_4, concentrated under reduced pressure, and the remaining material was purified by preparative TLC (hexane/ethyl acetate, 80 : 20 – 60 : 40) to give the enantioenriched products.

General procedure for the carbodiimide-mediated sulfonimidate tagging (GP5)

To a 8 mL test tube equipped with a magnetic stir bar, carboxylic acids (0.15 mmol, 1.5 equiv.), *N*,*N*-diisopropylethylamine (DIPEA) (0.20 mmol, 2 equiv.), *N*,*N*'-diisopropylcarbodiimide (DIC) (0.20 mmol, 2 equiv.), 4-pyrrolidinopyridine (PPY) (0.01 mmol, 0.1 equiv.), sulfonimidate **3a** (0.1 mmol, 1.0 equiv.), and dichloromethane (1 mL) were added in a glove-box. The reaction tube was capped, and the reaction mixture was stirred for overnight at 22–23 °C. For work-up, a saturated solution of brine (1.5 mL) was added, followed by dichloromethane (5 mL). The reaction mixture was then extracted with dichloromethane (3 × 5 mL). The organic phases were combined, dried over anhydrous Na_2SO_4, concentrated under reduced pressure, and the remaining material was purified by preparative TLC (hexane/ethyl acetate, 80 : 20 – 60 : 40) to give the enantioenriched products.

General procedure for the methylation of sulfonimidates 3 (GP6)

To a 10 mL screw capped reaction tube equipped with a magnetic stirbar were added sulfonimidate **3** (0.3 mmol), Proton sponge (96 mg, 0.45 mmol, 1.5 equiv.), trimethyloxonium tetrafluoroborate (89 mg, 0.6 mmol, 2 equiv.), and dichloromethane (3 mL), and the reaction mixture was stirred at 10 °C for the 2 h. After the completion of reaction (monitored by thin layer chromatography), the reaction mixture was filtered through small pad of celite, and organic layer was concentrated under reduced pressure. The crude material was purified by column chromatography on silica gel with a mixture of hexane and ethyl acetate as an eluent to give the methyl-capped sulfonimidate intermediate.

General procedure for the construction of sulfoximines with organolithium reagents (GP7)

To a 10 mL screw capped reaction tube equipped with a magnetic stirbar were added methyl-capped sulfonimidate intermediate obtained in GP6 (0.1 mmol) and diethyl ether (1 mL), and the reaction mixture was cooled to –78 °C or –100 °C and then respective organolithium reagents were slowly added. After the completion of reaction (monitored by thin layer chromatography), the reaction mixture was quenched with mixture of EDTA and ammonium hydroxide (pH = 9) and the organic layer was separated, and the aqueous layer was extracted with ethyl acetate (3 × 2 mL). The combined organic layers were dried over sodium sulfate and concentrated under reduced pressure. The crude mixture was purified by column chromatography on silica gel with a mixture of hexane and ethyl acetate as an eluent to give the desired product.

Additional experimental studies

Linear regression analysis of the kinetic resolution reaction

Linear regression analysis was carried out to rule out the involvement of complex higher-order processes (such as reversible resiolution, substantially different catalyst binding constants for the substarte eneantiomers, and product ineractions with the catalyst) in the kinetic resolution reaction. The reaction was carried out as described in GP3 with substrate *rac*-**1a** and diol **2**, and aliquots were taken as the reaction progressed. The ee of product **3a** and recovered

starting material **1a** were determined by HPLC. The regression plot (Figure S1) revealed linear dependence. This result is consistent with a process, which is first-order in substarte and is devoid of complex kinetic behavior, making the use of selectivity factor *s* appropriate for decribing the present kinetic resolution reaction.[1]

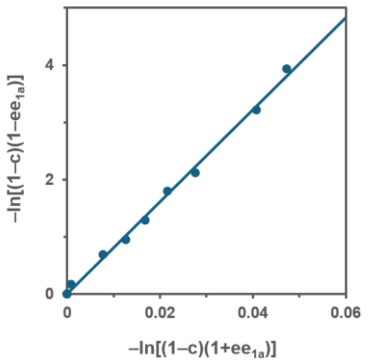

Figure S1. Linear regression analysis of the kinetic resolution reaction of sulfonimidoyl chlorides.

Variable time normalization analysis for the kinetic resolution of sulfonimidoyl chloride (1a)

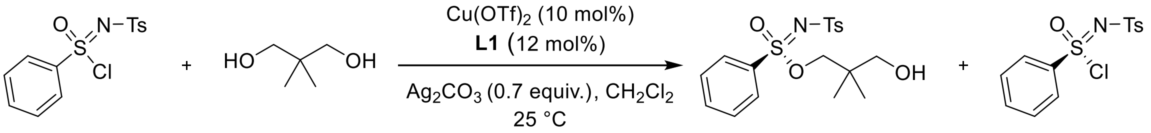

According to GP3, the reaction was carried out with sulfonimidoyl chloride *rac*-**1a** (33 mg, 0.1 mmol), and diol **2** (17.7 mg, 0.17 mmol) in anhydrous dichloromethane (1 mL). After every 5 minutes, an aliquot (0.03 mL) was withdrawn and EDTA work-up was done to quench the reaction. After concentrating the reaction mixture, a ^{1}H NMR spectrum was recorded. Three identical experiments were carried out, and each reaction was used to withdraw 3–5 aliquots to get 9–15 data points. The reaction was repeated with *rac*-**1a** (43 mg, 0.13 mmol and 26.4 mg, 0.08 mmol) to determine the reaction order of *rac*-**1a**, and with diol **2** (26 mg, 0.25 mmol and 12.5 mg, 0.12 mmol) to determine the reaction order of diol **2**. The reaction was also repeated according to GP3 with $Cu(OTf)_2$ (2.25 mg, 0.007 mmol) and **L1** (4.92 mg, 0.0088 mmol) to determine the reaction order for the catalyst.

Kinetic study of the catalyst rate order

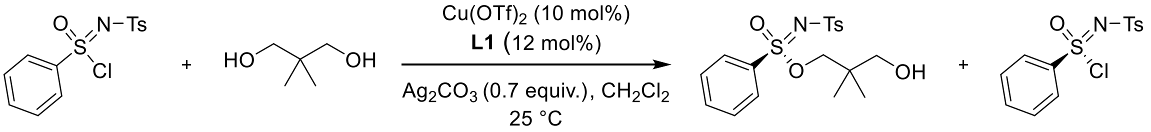

According to GP3, the reaction was carried out with *N*-tosylbenzenesulfonimidoyl chloride *rac*-**1a** (33 mg, 0.1 mmol), and diol **2** (17.7 mg, 0.17 mmol) in anhydrous dichloromethane (1 mL). After every 5 minutes, an aliquot (0.03 mL) was withdrawn and EDTA work-up was done to quench the reaction. After concentrating the reaction mixture, a ^{1}H NMR spectrum was recorded. Each reaction was used to withdraw three aliquots to give three data points. The reaction was repeated with different catalyst loadings (7 mol %, 13 mol %, 16 mol %) following the same procedure. The rate order was determined from the linear dependence (R^2 = 0.98) of the initial reaction rate on the initial concentration of copper catalyst (Figure S2.B) and is consistent with the result of the VTNA analysis (Figure 4.E).

Additional studies of the role of silver carbonate in the kinetic resolution reaction

To investigate the roles of silver carbonate in the reaction, experiments were carried out in the absence of the Cu/**L1** catalyst or $Cu(OTf)_2$. No reaction was observed without the Cu/**L1** catalyst (98% recovered ***rac*-1a**). Similarly, a 2% yield of sulfonimidate **3a** and 86% recovered sulfonimidoyl chloride ***rac*-1a** was observed without $Cu(OTf)_2$. Furthermore, a significant deterioration of the reaction performance was observed, when the reaction was carried out in the presence of 5 mol% silver tetrafluoroborate (15% yield and 33% ee for **3a** and 65% yield and 45% ee for **1a**).

Additionally, no complexation between the silver and diol **2** was observed by DOSY NMR experiments when diol **2** was treated with silver carbonate. Taken together, these results indicate that silver(I) cannot mediate sulfonimidate formation and the additional silver(I) does not accelerate the reaction, suggesting that silver coordination or chelation may not have a significant role under the reaction conditions.

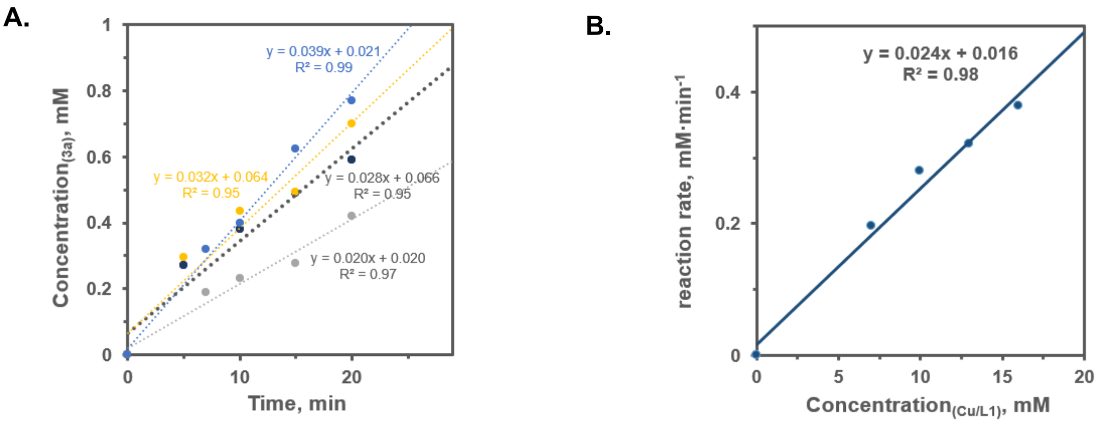

Figure S2. Catalyst rate order study. **A.** Kinetic profiles of the kinetic resolution reaction at different catalyst loadings. (●) 7 mol%; (●) 10 mol%; (●) 13 mol%; (●) 16 mol%. **B.** Dependence of the reaction rate on the catalyst concentration.

Study of the influence of the enantiomeric purity of the catalyst on the kinetic resolution reaction of sulfonimidoyl chlorides

The reactions were carried out as described in GP1 with the ligand **L1** of varied enantiomeric purity (prepared by mixing aliquots of solutions of the enantiomers of ligand **L1**). The enantioselectivity and conversion at specific enantiopurities of ligand **L1** were used to calculate the stereoselectivity factor (*s*) according to eq. 1. The intrinsic stereoselectivity factor (*s′* = 83) obtained experimental with enantiomerically pure ligand **L1** was then used to calculate the theoretical (s_{theor}) stereoselectivity factors as a function of ee of scalemic **L1** (ee_{L1}) according to eq. 2:[2]

$$s = \frac{\ln[(1-c)(1-ee_{sm})]}{\ln[(1-c)(1+ee_{sm})]} \qquad (1)$$

$$s_{theor} = \frac{s'(1+ee_{\mathbf{L1}})-(1-ee_{\mathbf{L1}})}{(1+ee_{\mathbf{L1}})+s'(1-ee_{\mathbf{L1}})} \qquad (2)$$

Preparation and X-ray crystal structure of complex 21

Ligand *ent*-**L1** (5.6 mg, 0.01 mmol, 10 mol%), $Cu(OTf)_2$ (3.6 mg, 0.01 mmol, 10 mol%) and diol **2** (20.8 mg, 0.2 mmol) were dissolved in a minimum volume of diethyl ether to give a clear solution. Slow diffusion of pentane into the obtained solution to give blue crystals for complex **21**.

Diffusion-ordered ^{1}H NMR spectroscopy (DOSY) studies of the catalyst speciation

The DOSY experiment was run using the dstebpgp3s pulse sequence with LED and convection compensation using the following: d20 = 0.07 s, p30 = 850 µs, d1 = 15 s, ns = 16, gpz6 = 5-95% using a squared (q) ramp.

The data was processed using MestreNova (version 14.3) by Whittaker Smoother baseline correction of the first spectrum in the array which was then applied to all other spectra. The DOSY was transformed using a Bayesian transform (0.1 resolution factor, 0 repetitions) to give a 2D DOSY spectrum with logD values between –2 and –8 cm^2/s.

Construction of an internal calibration curve

Perylene, 1,3-bis(trifluoromethyl)benzene, fluorobenzene were taken as the known reference for the molecular weight determination of the desired complex (Figure S3). Equimolar solutions (0.1 M) of each component were measured and dissolved in 0.5 mL $CDCl_3$ to prepare the DOSY NMR sample inside a glovebox.

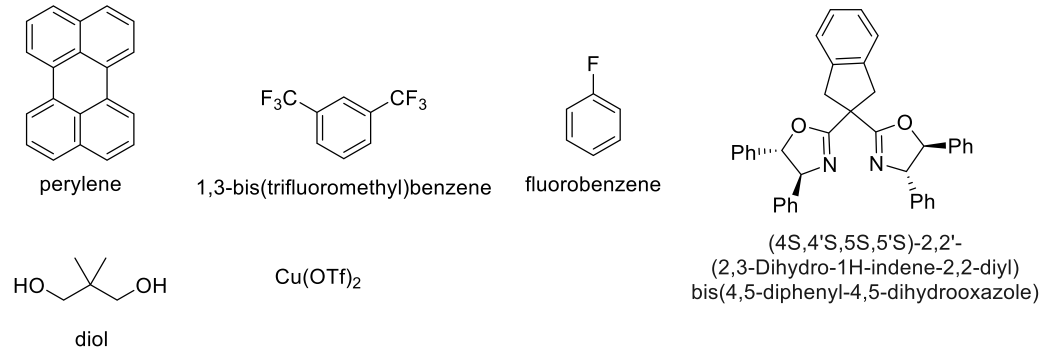

Figure S3. Reference compounds used in the DOSY NMR study.

After the experiment, from the NMR data the logD (D = diffusion coefficient) values for perylene, 1,3-bis(trifluoromethyl)benzene, fluorobenzene and the complex were obtained. These values were plotted (series 1) against the logarithmic value of molecular masses of the known references (perylene, 1,3-bis(trifluoromethyl)benzene, fluorobenzene).

Table S1. Results of the DOSY NMR study of the copper catalyst speciation.

	**MW**	**log MW (known)**	**Log D (obtained from DOSY NMR)**
**fluorobenzene**	96.1	1.98272339	-4.5
**bis CF_3Ph**	214.1	2.33061667	-4.65
**perylene**	252.3	2.40193446	-4.73
**complex DOSY**	851.8 (experimental)	2.93035994 (obtained from plot)	-4.98
**complex theoretical MW**	832.5		
**error (Δ MW)**	2.3%		

$$\Delta MW(\%) = \{(MWexpected - MWmeasured)/MWexpected\} \times 100\%$$

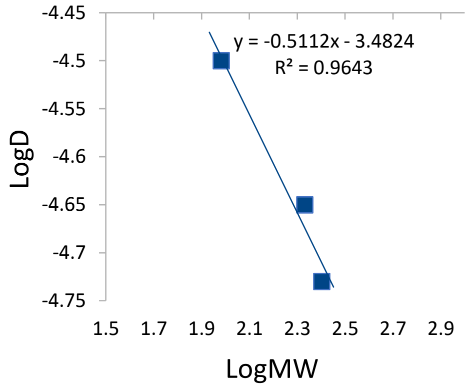

Figure S4. Logarithmic plot of the molecular weight–diffusion coefficient dependence.

From the equation of the plotted curve the molecular mass of the desired complex were determined (Figure S4). The experimental molecular mass (MW_{exp} = 851.8, 2.3% error) of the complex suggests formation of the ligand+Cu+2 diol complex ($MW_{theoretical}$ = 832.5).

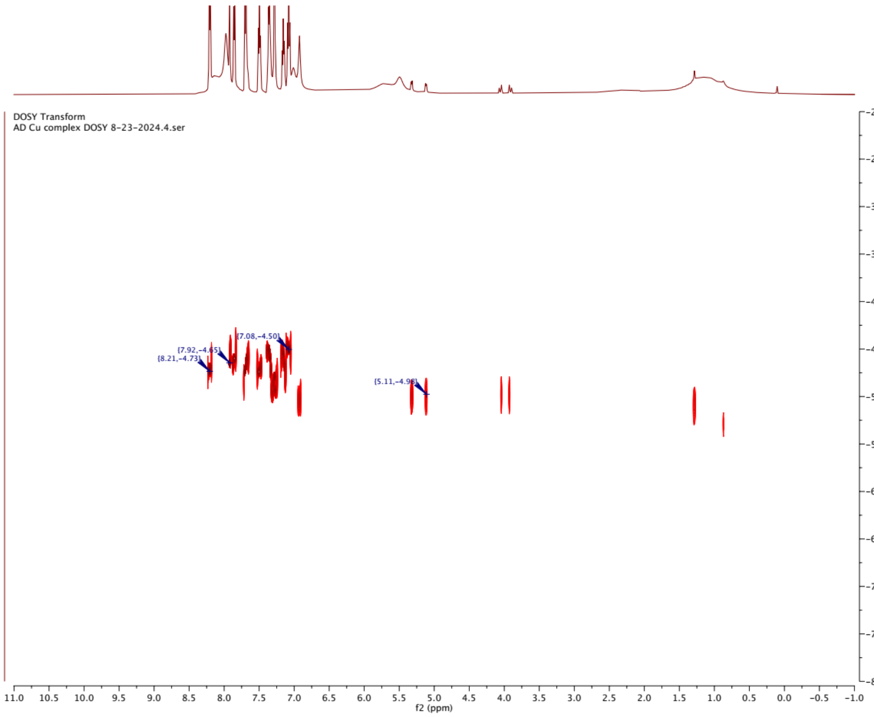

Figure S5. DOSY NMR spectrum of the reaction mixture.

Diffusion-ordered ¹H NMR spectroscopy (DOSY) studies of the mixture of silver carbonate and diol 2

The DOSY experiment was run using the dstebpgp3s pulse sequence with LED and convection compensation using the following: d20 = 0.06 s, p30 = 550 ms, d1=3 s, ns = 16, gpz6 = 5–95% using a squared (q) ramp.

The data was processed using MestreNova (version 14.3) by Whittaker Smoother baseline correction of the first spectrum in the array which was then applied to all other spectra. The DOSY was transformed using a Bayesian transform (0.1 resolution factor, 0 repetitions) to give a 2D DOSY spectrum with logD values between –2 and –8 cm^2/s.

Construction of an internal calibration curve

1,2-diiodobenzene, 1-bromo- 3-(trifluoromethyl)benzene and 1-fluoro-4-methylbenzene were taken as the known reference for the molecular weight determination of the desired complex (Figure S6). Equimolar solutions (0.1 M) of each component were measured and dissolved in 0.5 mL $CDCl_3$ to prepare the DOSY NMR sample inside a glovebox.

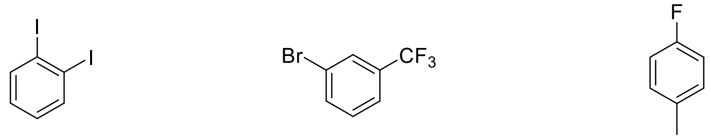

1,2-diiodobenzene 1-bromo-3-(trifluoromethyl)benzene 1-fluoro-4-methylbenzene

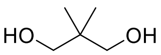

Ag_2CO_3

2,2-dimethylpropane-1,3-diol

Figure S6. Reference compounds used in the DOSY NMR study of the diol **2** – silver carbonate mixture speciation.

After the experiment, from the NMR data the logD (D = diffusion coefficient) values for 1,2-diiodobenzene, 1-bromo-3-(trifluoromethyl)benzene, 1-fluoro-4-methylbenzene, and the mixture of 2,2-dimethylpropane-1,3-diol (**2**) and silver carbonate were obtained. These values were plotted (series 1) against the logarithmic value of molecular masses of the known references (1,2-diiodobenzene, 1-bromo- 3-(trifluoromethyl)benzene, 1-fluoro-4-methylbenzene).

Table S2. Results of the DOSY NMR study of the diol **2** – silver carbonate mixture speciation.

	Molecular weight	Log(MW)	log D
**1-fluoro-4-methylbenzene**	110.13	2.041906	-4.52
**1-bromo- 3-(trifluoromethyl)benzene**	225.01	2.352202	-4.61
**1,2-diiodobenzene**	329.91	2.518382	-4.65
**Sample (diol+Ag_2CO_3)**	109.10	2.037818	-4.52
**Expected MW of complex**	211.00		

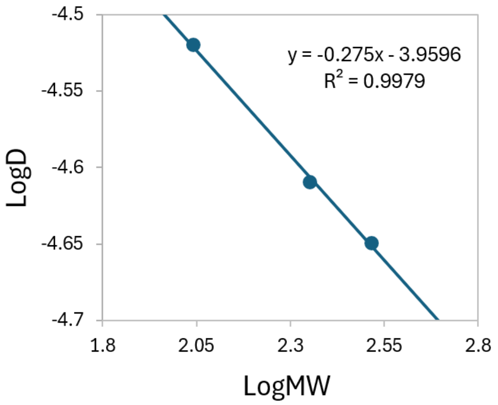

Figure S7. Logarithmic plot of the molecular weight–diffusion coefficient dependence.

From the equation of the plotted curve the molecular mass of the desired complex were determined (Figure S7). The experimental molecular mass (MW_{exp} = 109.10) suggests that there is no complexation between diol **2** and silver metal.

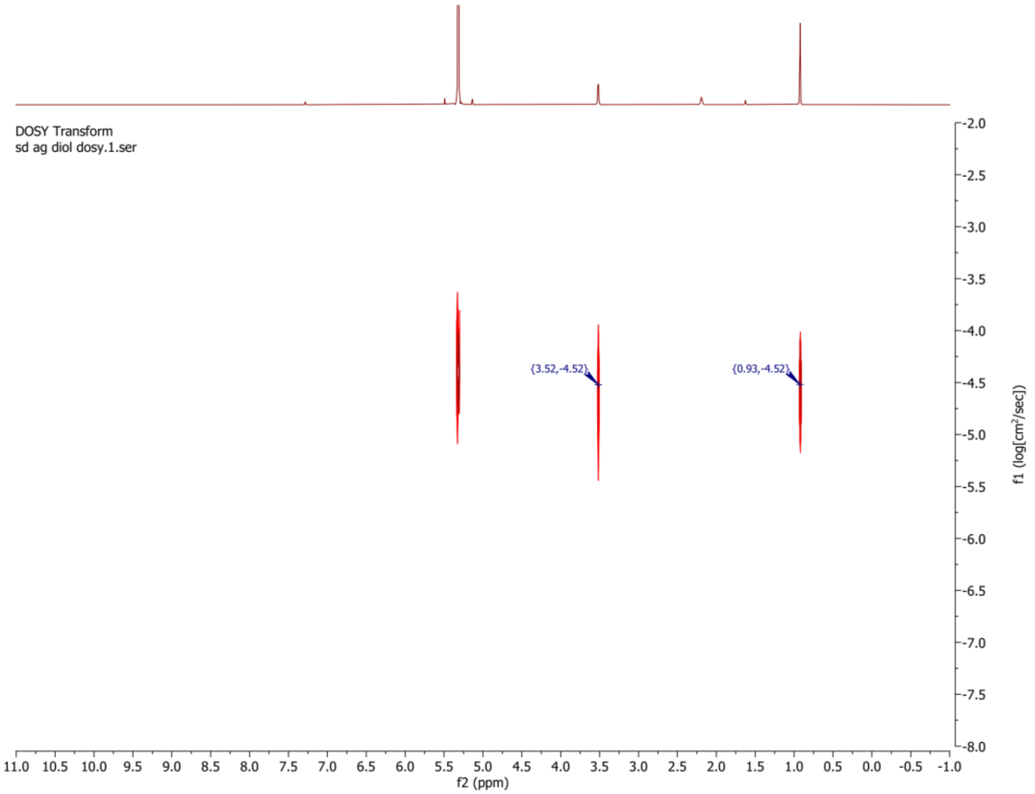

Figure S8. DOSY NMR spectrum of the diol **2**–silver carbonate mixture.

Synthesis of sulfonimidoyl chlorides

***N*-Tosylbenzenesulfonimidoyl chloride (*rac*-1a)**

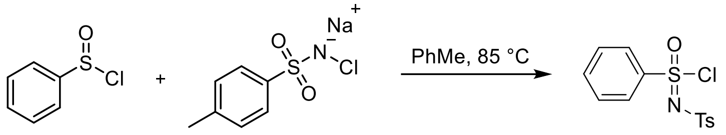

According to GP1, the reaction was carried out with benzenesulfinic chloride (320 mg, 2.5 mmol) prepared from benzenesulfonyl chloride (528 mg, 3.0 mmol), and chloramine-T (569 mg, 2.5 mmol) in toluene (12 mL). After the completion of the reaction, it was then filtered through celite with toluene. The filtrate was collected, the solvent was removed under reduced pressure, and the remaining material was purified by flash chromatography on silica gel (EtOAc/hexane, 2 : 8 v/v) to give product ***rac*-1a** (601 mg, 73%) as a white solid.

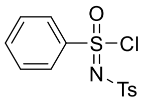

m.p.: 35–37 °C. – ^{1}H NMR (300 MHz, $CDCl_3$) δ 8.11–8.04 (m, 2H), 7.99 (d, *J* = 8.3 Hz, 2H), 7.78 (t, *J* = 7.5 Hz, 1H), 7.64 (t, *J* = 7.8 Hz, 2H), 7.37 (d, *J* = 8.0 Hz, 2H), 2.46 (s, 3H) ppm. – ^{13}C NMR (126 MHz, $CDCl_3$) *δ* 144.6, 142.6, 138.3, 135.6, 129.8, 129.7, 127.5, 126.9, 21.7 ppm. – IR: 2970, 1738, 1597, 1449, 1365, 1342, 1287, 1163, 1114, 1087, 896, 734 cm^{-1}. – HRMS: calcd for $C_{13}H_{12}ClNO_3S_2$: 328.9947, found 328.9942 [M+H$^+$].

2-Floro-*N*-tosylbenzenesulfonimidoyl chloride (*rac*-1b)

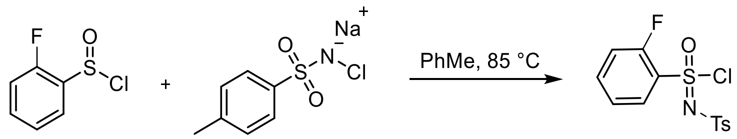

According to GP1, the reaction was carried out with 2-fluorobenzenesulfinic chloride (445 mg, 2.5 mmol) prepared from 2-fluorobenzenesulfinic chloride (582 mg, 3.0 mmol), and chloramine-T (569 mg, 2.5 mmol) in toluene (12 mL). After the completion of the reaction, it was then filtered through celite with toluene. The filtrate was collected, the solvent was removed under reduced pressure, and the remaining material was purified by flash chromatography on silica gel (EtOAc/hexane, 3 : 7 v/v) to give product ***rac*-1b** (625 mg, 72%) as a pale yellow solid.

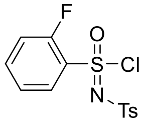

m.p.: 70–73 °C. – ^{1}H NMR (500 MHz, $CDCl_3$) *δ* 8.09–8.00 (m, 1H), 7.98 (d, *J* = 8.0 Hz, 2H), 7.83–7.74 (m, 1H), 7.41–7.32 (m, 4H), 2.47 (s, 3H) ppm. – ^{13}C NMR (126 MHz, $CDCl_3$) *δ* 158.6 (d, *J* = 265.0 Hz), 144.6, 138.2, 138.2 (d, *J* = 8.9 Hz), 130.3 (d, *J* = 11.7 Hz), 129.7, 129.3, 127.5, 126.5, 124.7 (d, *J* = 4.1 Hz), 21.7 ppm. – ^{19}F NMR (471 MHz, $CDCl_3$) *δ* –104.41 ppm. – IR: 2925, 1594, 1478, 1342, 1291, 1264, 1186, 1131, 1110, 1087, 1062, 830, 814, 734 cm^{-1}. – HRMS: calcd for $C_{13}H_{11}ClFNO_3S_2$: 347.9926, found 347.9918 [M+H$^+$].

4-Chloro-*N*-tosylbenzenesulfonimidoyl chloride (*rac*-1c)

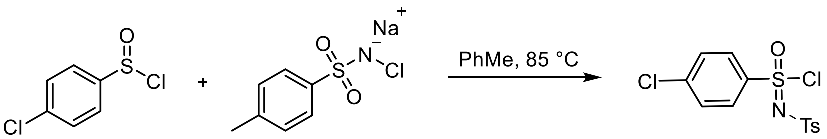

According to GP1, the reaction was carried out with 4-chlorobenzenesulfinic chloride (485 mg, 2.5 mmol) prepared from 4-chlorobenzenesulfinic chloride (630 mg, 3.0 mmol), and chloramine-T (569 mg, 2.5 mmol) in toluene (12 mL). After the completion of the reaction, it was then filtered through celite with toluene. The filtrate was collected, the solvent was removed under reduced pressure, and the remaining material was purified by flash chromatography on silica gel (EtOAc/hexane, 3 : 7 v/v) to give product ***rac*-1c** (617 mg, 68%) as a white crystalline solid.

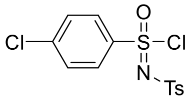

m.p.: 71–73 °C. – ^{1}H NMR (500 MHz, $CDCl_3$) *δ* 8.02 (d, *J* = 8.5 Hz, 2H), 7.98 (d, *J* = 8.0 Hz, 2H), 7.61 (d, *J* = 8.6 Hz, 2H), 7.38 (d, *J* = 7.8 Hz, 2H), 2.47 (s, 3H) ppm. – ^{13}C NMR (126 MHz, $CDCl_3$) *δ* 144.7, 142.7, 140.9, 138.2, 130.1, 129.8, 128.3, 127.5, 126.5, 21.7 ppm. – IR: 3092, 2923, 1596, 1573, 1471, 1396, 1339, 1278, 1162, 1113, 1083, 1009 cm^{-1}. – HRMS: calcd for $C_{13}H_{11}Cl_2NO_3S_2$: 401.9189, found 401.9182 [M+K$^+$].

4-Bromo-*N*-tosylbenzenesulfonimidoyl chloride (*rac*-1d)

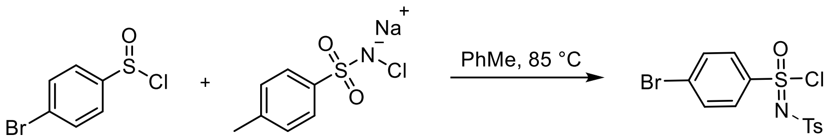

According to GP1, the reaction was carried out with 4-bromobenzenesulfinic chloride (595 mg, 2.5 mmol) prepared from 4-bromobenzenesulfinic chloride (761 mg, 3.0 mmol), and chloramine-T (569 mg, 2.5 mmol) in toluene (12 mL). After the completion of the reaction, it was then filtered through celite with toluene. The filtrate was collected, the solvent was removed under reduced pressure, and the remaining material was purified by flash chromatography on silica gel (EtOAc/hexane, 3 : 7 v/v) to give product ***rac*-1d** (560 mg, 55%) as a pale yellow solid.

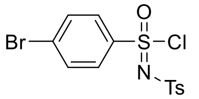

m.p.: 97–99 °C. – ^{1}H NMR (500 MHz, $CDCl_3$) δ 7.98 (d, J = 7.5 Hz, 2H), 7.93 (d, J = 7.6 Hz, 2H), 7.77 (d, J = 8.0 Hz, 2H), 7.38 (d, J = 7.8 Hz, 2H), 2.47 (s, 3H) ppm. – ^{13}C NMR (126 MHz, $CDCl_3$) δ 144.7, 141.5, 138.2, 133.2, 131.4, 129.8, 128.3, 127.5, 126.5, 21.7 ppm. – IR: 3092, 2922, 2852, 1596, 1566, 1468, 1391, 1339, 1277, 1163, 1112, 1087, 1066, 1005 cm^{-1}. – HRMS: calcd for $C_{13}H_{11}BrClNO_3S_2$: 445.8684, found 445.8674 [M+K^{+}].

4-Iodo-*N*-tosylbenzenesulfonimidoyl chloride (*rac*-1e)

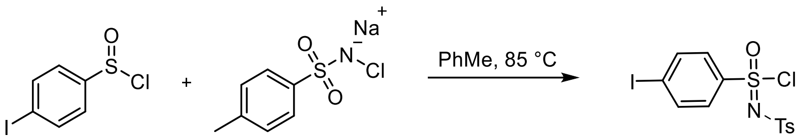

According to GP1, the reaction was carried out with 4-iodobenzenesulfinic chloride (715 mg, 2.5 mmol) prepared from 4-iodobenzenesulfonyl chloride (906 mg, 3.0 mmol), and chloramine-T (569 mg, 2.5 mmol) in toluene (12 mL). After the completion of the reaction, it was then filtered through celite with toluene. The filtrate was collected, the solvent was removed under reduced pressure, and the remaining material was purified by flash chromatography on silica gel (EtOAc/hexane, 2 : 8 v/v) to give product ***rac*-1e** (648 mg, 57%) as a pale yellow solid.

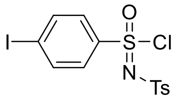

m.p.: 117–119 °C. – ^{1}H NMR (500 MHz, $CDCl_3$) δ 8.05–7.90 (m, 4H), 7.76 (d, J = 8.3 Hz, 2H), 7.37 (d, J = 8.1 Hz, 2H), 2.47 (s, 3H) ppm. – ^{13}C NMR (126 MHz, $CDCl_3$) δ 144.7, 142.2, 139.2, 138.2, 129.8, 127.9, 127.5, 104.3, 21.7 ppm. – IR: 2969, 1738, 1589, 1456, 1342, 1289, 1230, 1162, 1112, 1086, 1001, 813, 724 cm^{-1}. – HRMS: calcd for $C_{13}H_{11}ClINO_3S_2$: 477.8806, found 477.8814 [M+Na^{+}].

4-Nitro-*N*-tosylbenzenesulfonimidoyl chloride (*rac*-1f)

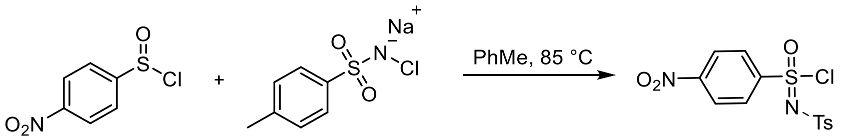

According to GP1, the reaction was carried out with 4-nitrobenzenesulfinic chloride (514 mg, 2.5 mmol) prepared from 4-nitrobenzenesulfonyl chloride (665 mg, 3.0 mmol), and chloramine-T (569 mg, 2.5 mmol) in toluene (12 mL). After the completion of the reaction, it was then filtered through celite with toluene. The filtrate was collected, the solvent was removed under reduced pressure, and the remaining material was purified by flash chromatography on silica gel (EtOAc/hexane, 3 : 7 v/v) to give product ***rac*-1f** (617 mg, 66%) as a pale yellow solid.

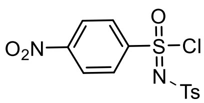

m.p.: 95–97 °C. – ^{1}H NMR (500 MHz, $CDCl_3$) δ 8.47 (d, J = 9.0 Hz, 2H), 8.30 (d, J = 9.0 Hz, 2H), 7.99 (d, J = 8.3 Hz, 2H), 7.40 (d, J = 8.2 Hz, 2H), 2.48 (s, 3H) ppm. – ^{13}C NMR (126 MHz, $CDCl_3$) δ 151.4, 147.2, 145.1, 137.9, 129.9, 128.5, 127.5, 125.0, 21.7 ppm. – IR: 2922, 1596, 1532, 1340, 1287, 1163, 1116, 1086, 1069, 1009 cm^{-1}. – HRMS: calcd for $C_{13}H_{11}ClN_2O_3S_2$: 392.0136, found 392.0133 [M+NH$_4^{+}$].

4-Methoxy-*N*-tosylbenzenesulfonimidoyl chloride (*rac*-1g)

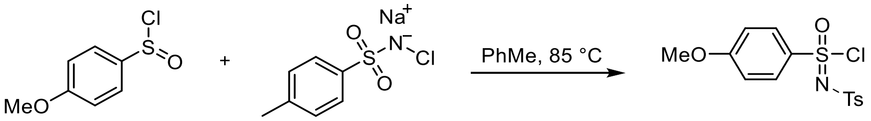

According to GP1, the reaction was carried out with 4-methoxybenzenesulfinic chloride (475 mg, 2.5 mmol) prepared from 4-methoxybenzenesulfonyl chloride (618 mg, 3.0 mmol), and chloramine-T (569 mg, 2.5 mmol) in toluene (12 mL). After the completion of the reaction, it was then filtered through celite with toluene. The filtrate was collected, the solvent was removed under reduced pressure, and the remaining material was purified by flash chromatography on silica gel (EtOAc/hexane, 3 : 7 v/v) to give product ***rac*-1g** (503 mg, 56%) as a white solid.

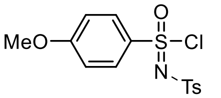

m.p.: 60–63 °C. – ^{1}H NMR (500 MHz, $CDCl_3$) δ 8.05–7.95 (m, 4H), 7.36 (d, *J* = 8.1 Hz, 2H), 7.05 (d, *J* = 9.2 Hz, 2H), 3.93 (s, 3H), 2.46 (s, 3H) ppm. – ^{13}C NMR (126 MHz, $CDCl_3$) δ 165.3, 144.4, 143.7, 139.1, 138.5, 129.6, 129.7, 127.5, 126.5, 114.9, 56.1, 21.6 ppm. – IR: 2944, 1738, 1588, 1493, 1339, 1270, 1205, 1163, 1106, 1085, 1019, 834, 815, 733 cm^{-1}. – HRMS: calcd for $C_{14}H_{14}ClNO_4S_2$: 360.0126, found 360.0129 [M+H$^+$].

4-(Difluoromethoxy)-*N*-tosylbenzenesulfonimidoyl chloride (*rac*-1h)

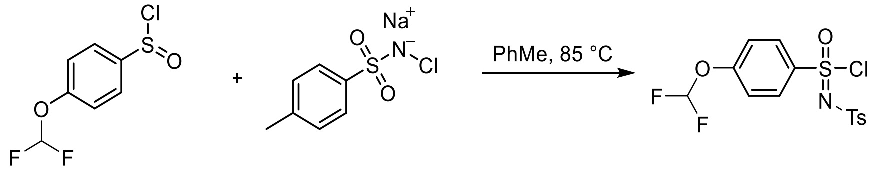

According to GP1, the reaction was carried out with 4-(difluoromethoxy)benzenesulfinic chloride (565 mg, 2.5 mmol) prepared from 4-(difluoromethoxy)benzene sulfonyl chloride (726 mg, 3.0 mmol), and chloramine-T (569 mg, 2.5 mmol) in toluene (12 mL). After the completion of the reaction, it was then filtered through celite with toluene. The filtrate was collected, the solvent was removed under reduced pressure, and the remaining material was purified by flash chromatography on silica gel (EtOAc/hexane, 3 : 7 v/v) to give product ***rac*-1h** (603 mg, 61%) as a pale yellow solid.

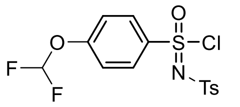

m.p.: 50–53 °C. – ^{1}H NMR (500 MHz, $CDCl_3$) δ 8.10 (d, *J* = 8.7 Hz, 2H), 7.98 (d, *J* = 7.8 Hz, 2H), 7.36 (dd, *J* = 20.5, 8.4 Hz, 4H), 6.67 (t, *J* = 71.8 Hz, 1H), 2.47 (s, 3H) ppm. – ^{13}C NMR (126 MHz, $CDCl_3$) δ 156.2, 144.7, 138.6, 138.3, 129.7, 129.5, 127.5, 119.8, 114.8 (t, *J* = 265.0 Hz), 21.7 ppm. – ^{19}F NMR (471 MHz, $CDCl_3$) δ –81.80, –81.95 ppm. – IR: 2940, 1738, 1590, 1456, 1416, 1347, 1231, 1206, 1162, 1126, 1043, 949, 814 cm^{-1}. – HRMS: calcd for $C_{14}H_{12}ClF_2NO_4S_2$: 417.9795, found 417.9751 [M+Na$^+$].

***N*-Tosyl-3-(trifluoromethoxy)benzenesulfonimidoyl chloride (*rac*-1i)**

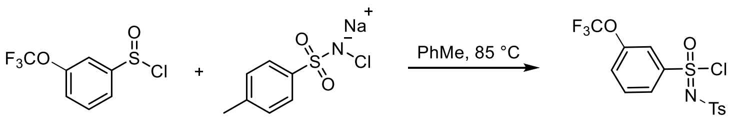

According to GP1, the reaction was carried out with 4-(trifluoromethoxy)benzenesulfinic chloride (610 mg, 2.5 mmol) prepared from 4-(trifluoromethoxy)benzenesulfonly chloride (780 mg, 3.0 mmol), and chloramine-T (569 mg, 2.5 mmol) in toluene (12 mL). After the completion of the reaction, it was then filtered through celite with toluene. The filtrate was collected, the solvent was removed under reduced pressure, and the remaining material was purified by flash chromatography on silica gel (EtOAc/hexane, 3 : 7 v/v) to give product ***rac*-1i** (510 mg, 49%) as a pale yellow liquid.

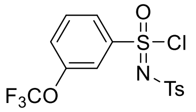

^{1}H NMR (500 MHz, $CDCl_3$) δ 8.04 (d, *J* = 8.1 Hz, 1H), 7.99 (d, *J* = 7.8 Hz, 2H), 7.91 (s, 1H), 7.71 (t, *J* = 8.2 Hz, 1H), 7.63 (d, *J* = 8.2 Hz, 1H), 7.39 (d, *J* = 7.9 Hz, 2H), 2.48 (s, 3H) ppm. – ^{13}C NMR (126 MHz, $CDCl_3$) δ 149.4, 144.8, 144.1, 138.1, 131.6, 129.8, 127.8, 127.5, 125.1, 121.2, 119.5, 119.1, 21.7 ppm. – ^{19}F NMR (471 MHz, $CDCl_3$) δ –58.0 ppm. – IR: 3086, 1596, 1476, 1344, 1288, 1255, 1207, 1161, 1108, 1084, 997, 946, 888 cm^{-1}. – HRMS: calcd for $C_{14}H_{11}ClF_3NO_4S_2$: 413.9843, found 413.9833 [M+H$^+$].

3-(Methylsulfonyl)-*N*-tosylbenzenesulfonimidoyl chloride (*rac*-1j)

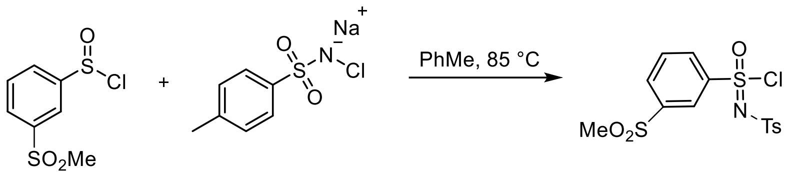

According to GP1, the reaction was carried out with 3-(methylsulfonyl)benzenesulfinic chloride (595 mg, 2.5 mmol) prepared from 3-(methylsulfonyl)benzene sulfonyl chloride (761 mg, 3.0 mmol), and chloramine-T (569 mg, 2.5 mmol) in toluene (12 mL). After the completion of the reaction, it was then filtered through celite with toluene. The filtrate was collected, the solvent was removed under reduced pressure, and the remaining material was purified by flash chromatography on silica gel (EtOAc/hexane, 4 : 6 v/v) to give product ***rac*-1j** (590 mg, 58%) as a pale yellow solid.

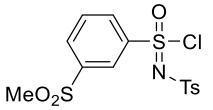

m.p.: 120–122 °C. – ^{1}H NMR (500 MHz, $CDCl_3$) *δ* 8.61 (s, 1H), 8.36 (dd, *J* = 18.1, 7.9 Hz, 2H), 7.99 (d, *J* = 8.0 Hz, 2H), 7.90 (t, *J* = 8.0 Hz, 1H), 7.40 (d, *J* = 7.9 Hz, 2H), 3.16 (s, 3H), 2.48 (s, 3H) ppm. – ^{13}C NMR (126 MHz, $CDCl_3$) *δ* 145.0, 144.1, 142.9, 137.9, 134.0, 131.6, 131.4, 129.9, 127.6, 126.0, 44.4, 21.7 ppm. – IR: 3071, 2926, 1596, 1411, 1321, 1303, 1288, 1187, 1157, 1118, 1084, 993, 962 cm^{-1}. – HRMS: calcd for $C_{14}H_{14}ClNO_3S_3$: 445.9354, found 445.9342 [M+K$^+$].

4-Isocyano-*N*-tosylbenzenesulfonimidoyl chloride (*rac*-1k)

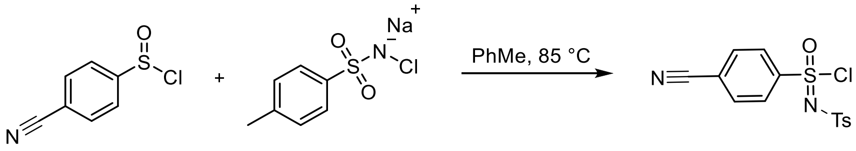

According to GP1, the reaction was carried out with 4-isocyanobenzenesulfinic chloride (463 mg, 2.5 mmol) prepared from 4-isocyanobenzenesulfonyl chloride (603 mg, 3.0 mmol), and chloramine-T (569 mg, 2.5 mmol) in toluene (12 mL). After the completion of the reaction, it was then filtered through celite with toluene. The filtrate was collected, the solvent was removed under reduced pressure, and the remaining material was purified by flash chromatography on silica gel (EtOAc/hexane, 3 : 7 v/v) to give product ***rac*-1k** (407 mg, 46%) as a pale yellow solid.

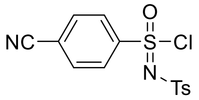

m.p.: 95–97 °C. – ^{1}H NMR (500 MHz, $CDCl_3$) *δ* 8.21 (d, *J* = 8.4 Hz, 2H), 7.96 (dd, *J* = 21.4, 8.3 Hz, 4H), 7.39 (d, *J* = 8.1 Hz, 2H), 2.48 (s, 3H) ppm. – ^{13}C NMR (126 MHz, $CDCl_3$) *δ* 146.0, 145.0, 137.9, 133.5, 129.9, 127.6, 127.5, 119.2, 116.3, 21.7 ppm. – IR: 3095, 2923, 2236, 1596, 1491, 1399, 1341, 1286, 1163, 1113, 1086, 1013 cm^{-1}. – HRMS: calcd for $C_{14}H_{11}ClN_2O_3S_2$: 372.0238, found 372.0235 [M+NH$_4^+$].

4-Methyl-*N*-tosylbenzenesulfonimidoyl chloride (*rac*-1l)

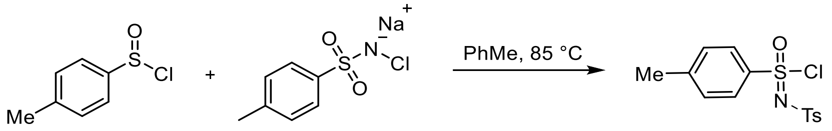

According to GP1, the reaction was carried out with 4-methylbenzenesulfinic chloride (435 mg, 2.5 mmol) prepared from 4-methylbenzenesulfonyl chloride (570 mg, 3.0 mmol), and chloramine-T (569 mg, 2.5 mmol) in toluene (12 mL). After the completion of the reaction, it was then filtered through celite with toluene. The filtrate was collected, the solvent was removed under reduced pressure, and the remaining material was purified by flash chromatography on silica gel (EtOAc/hexane, 3 : 7 v/v) to give product ***rac*-1l** (592 mg, 69%) as a pale yellow solid.

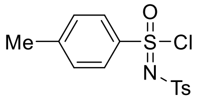

m.p.: 48–50 °C. – ^{1}H NMR (500 MHz, $CDCl_3$) *δ* 7.96 (dd, *J* = 18.4, 8.2 Hz, 4H), 7.38 (dd, *J* = 24.6, 8.2 Hz, 4H), 2.49 (s, 3H), 2.46 (s, 3H) ppm. – ^{13}C NMR (126 MHz, $CDCl_3$) *δ* 147.4, 144.5, 139.7, 138.4, 130.4, 129.7, 127.5, 126.9, 21.8, 21.7 ppm. – IR: 2924, 1743, 1596, 1478, 1456, 1342, 1285, 1205, 1151, 1111, 1087, 1013, 814, 731 cm^{-1}. – HRMS: calcd for $C_{14}H_{14}ClNO_3S_2$: 365.9996, found 365.9983 [M+Na$^+$].

4-(*tert*-butyl)-*N*-tosylbenzenesulfonimidoyl chloride (*rac*-1m)

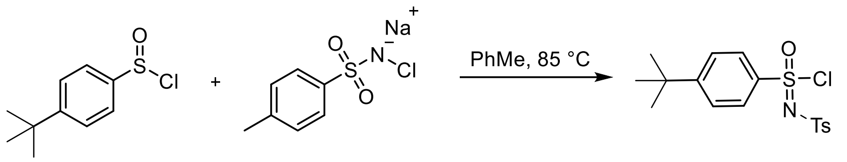

According to GP1, the reaction was carried out with 4-(*tert*-butyl) benzenesulfinic chloride (540 mg, 2.5 mmol) prepared from 4-(*tert*-butyl)benzene sulfonyl chloride (696 mg, 3.0 mmol), and chloramine-T (569 mg, 2.5 mmol) in toluene (12 mL). After the completion of the reaction, it was then filtered through celite with toluene. The filtrate was collected, the solvent was removed under reduced pressure, and the remaining material was purified by flash chromatography on silica gel (EtOAc/hexane, 3 : 7 v/v) to give product ***rac*-1m** (578 mg, 60%) as a pale yellow liquid.

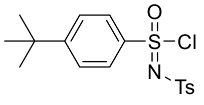

^{1}H NMR (500 MHz, $CDCl_3$) *δ* 7.99 (d, *J* = 8.2 Hz, 4H), 7.62 (d, *J* = 8.6 Hz, 2H), 7.36 (d, *J* = 8.1 Hz, 2H), 2.46 (s, 3H), 1.37 (s, 10H) ppm. – ^{13}C NMR (126 MHz, $CDCl_3$) *δ* 160.2, 144.4, 139.6, 138.5, 129.6, 127.5, 126.8, 126.8, 35.6, 30.9, 21.7 ppm. – IR: 2968, 1738, 1595, 148, 1265, 1230, 1205, 1152, 1124, 1100, 1087, 817, 735 cm^{-1}. – HRMS: calcd for $C_{17}H_{20}ClNO_3S_2$: 408.0465, found 408.0461 [M+Na$^+$].

***N*-Tosyl-[1,1'-biphenyl]-4-sulfonimidoyl chloride (*rac*-1n)**

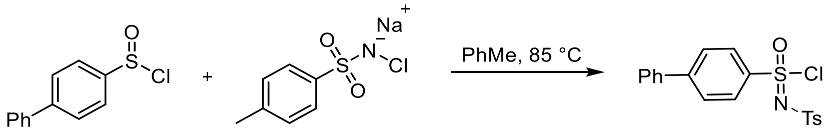

According to GP1, the reaction was carried out with [1,1'-biphenyl]-4-sulfinic chloride (590 mg, 2.5 mmol) prepared from [1,1'-biphenyl]-4-sulfonyl chloride (756 mg, 3.0 mmol), and chloramine-T (569 mg, 2.5 mmol) in toluene (12 mL). After the completion of the reaction, it was then filtered through celite with toluene. The filtrate was collected, the solvent was removed under reduced pressure, and the remaining material was purified by flash chromatography on silica gel (EtOAc/hexane, 2 : 8 v/v) to give product ***rac*-1n** (516 mg, 51%) as a white solid.

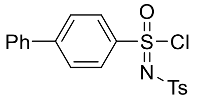

m.p.: 70–73 °C. – ^{1}H NMR (500 MHz, $CDCl_3$) *δ* 8.14 (d, *J* = 8.6 Hz, 2H), 8.01 (d, *J* = 8.3 Hz, 2H), 7.81 (d, *J* = 8.7 Hz, 2H), 7.62 (d, *J* = 6.8 Hz, 2H), 7.57 – 7.45 (m, 3H), 7.38 (d, *J* = 8.2 Hz, 2H), 2.48 (s, 3H). – ^{13}C NMR (126 MHz, $CDCl_3$) *δ* 148.8, 144.5, 140.9, 138.4, 138.2, 129.7, 129.4, 129.3, 128.3, 127.5, 127.5, 127.5, 21.7 ppm. – IR: 3063, 1587, 1477, 1399, 1338, 1284, 1185, 1163, 1109, 1085, 1004, 916 cm^{-1}. – HRMS: calcd for $C_{19}H_{16}ClNO_3S_2$: 423.0598, found 423.0595 [M+NH$_4^+$].

4'-Fluoro-*N*-tosyl-[1,1'-biphenyl]-4-sulfonimidoyl chloride (*rac*-1o)

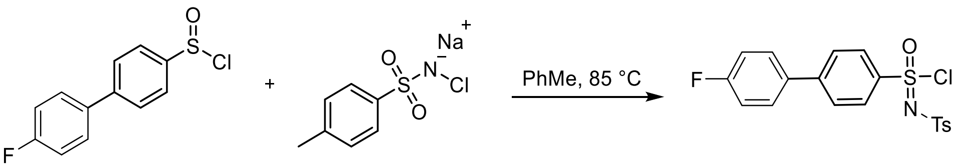

According to GP1, the reaction was carried out with 4'-fluoro-[1,1'-biphenyl]-4-sulfinic chloride (1385 mg, 2.5 mmol) prepared from 4'-fluoro-[1,1'-biphenyl]-4-sulfinoyl chloride (1710 mg, 3.0 mmol), and chloramine-T (569 mg, 2.5 mmol) in toluene (12 mL). After the completion of the reaction, it was then filtered through celite with toluene. The filtrate was collected, the solvent was removed under reduced pressure, and the remaining material was purified by flash chromatography on silica gel (EtOAc/hexane, 3 : 7 v/v) to give product ***rac*-1o** (593 mg, 56%) as a pale yellow solid.

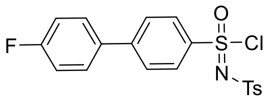

m.p.: 83–85 °C. – ^{1}H NMR (500 MHz, $CDCl_3$) *δ* 8.13 (d, *J* = 8.2 Hz, 2H), 8.01 (d, *J* = 7.9 Hz, 2H), 7.77 (d, *J* = 8.3 Hz, 2H), 7.68–7.55 (m, 2H), 7.38 (d, *J* = 8.0 Hz, 2H), 7.22 (t, *J* = 8.3 Hz, 2H), 2.47 (s, 3H) ppm. – ^{13}C NMR (126 MHz, $CDCl_3$) *δ* 163.6 (d, *J* = 250.1 Hz), 147.6, 144.5, 141.0, 138.4, 134.4 (d, *J* = 3.5 Hz), 129.7, 129.3 (d, *J* = 8.3 Hz), 128.1, 127.6 (d, *J* = 5.3 Hz), 116.4 (d, *J* = 21.8 Hz), 21.7 ppm. – ^{19}F

Go to table of contents

NMR (471 MHz, $CDCl_3$) δ –111.83, –113.64 ppm. – IR: 2969, 1738, 1595, 1365, 1216, 1205, 1161, 1127, 895, 821 cm^{-1}. – HRMS: calcd for $C_{19}H_{15}ClFNO_3S_2$: 446.0058, found 446.0054 [$M+Na^+$].

2,5-Difluoro-*N*-tosylbenzenesulfonimidoyl chloride (*rac*-1p)

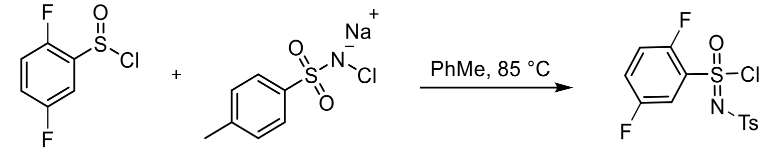

According to GP1, the reaction was carried out with 2,5-difluorobenzenesulfinic chloride (490 mg, 2.5 mmol) prepared from 2,5-difluorobenzenesulfonyl chloride (632 mg, 3.0 mmol), and chloramine-T (569 mg, 2.5 mmol) in toluene (12 mL). After the completion of the reaction, it was then filtered through celite with toluene. The filtrate was collected, the solvent was removed under reduced pressure, and the remaining material was purified by flash chromatography on silica gel (EtOAc/hexane, 3 : 7 v/v) to give product ***rac*-1p** (520 mg, 57%) as a white solid.

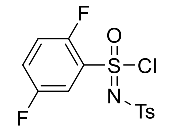

m.p.: 78–80 °C. – ^{1}H NMR (500 MHz, $CDCl_3$) δ 7.97 (d, *J* = 8.2 Hz, 2H), 7.80 – 7.71 (m, 1H), 7.53 – 7.44 (m, 1H), 7.36 (dd, *J* = 19.5, 6.0 Hz, 3H), 2.48 (s, 3H) ppm. – ^{13}C NMR (126 MHz, $CDCl_3$) δ 157.3 (d, *J* = 251.0 Hz), 154.8 (d, *J* = 261.5 Hz), 144.8, 138.1, 129.8, 127.5, 125.0 (d, *J* = 8.9 Hz), 124.8 (d, *J* = 8.8 Hz), 120.1 (d, *J* = 7.8 Hz), 119.9 (d, *J* = 7.9 Hz), 116.3 (d, *J* = 28.7 Hz), 21.7 ppm. – ^{19}F NMR (471 MHz, $CDCl_3$) δ –110.3 (dt, *J* = 13.0, 6.4 Hz), –113.1 (dd, *J* = 15.5, 8.2 Hz) ppm. – IR: 3070, 1596, 1490, 1402, 1341, 1290, 1254, 1199, 1163, 1109, 1084, 1047, 876 cm^{-1}. – HRMS: calcd for $C_{13}H_{10}ClF_2NO_3S_2$: 383.0097, found 383.0091 [$M+NH_4^+$].

3,5-Dichloro-*N*-tosylbenzenesulfonimidoyl chloride (*rac*-1q)

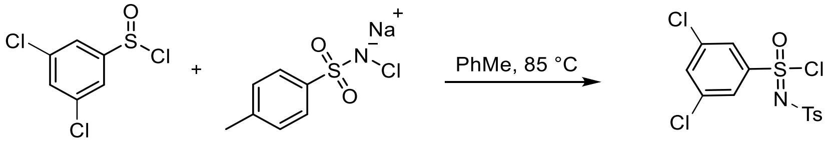

According to GP1, the reaction was carried out with 3,5-dichlorobenzenesulfinic chloride (570 mg, 2.5 mmol) prepared from 3,5-dichlorobenzenesulfonyl chloride (732 mg, 3.0 mmol), and chloramine-T (569 mg, 2.5 mmol) in toluene (12 mL). After the completion of the reaction, it was then filtered through celite with toluene. The filtrate was collected, the solvent was removed under reduced pressure, and the remaining material was purified by flash chromatography on silica gel (EtOAc/hexane, 3 : 7 v/v) to give product ***rac*-1q** (675 mg, 68%) as a pale yellow solid.

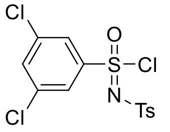

m.p.: 70–72 °C. – ^{1}H NMR (500 MHz, $CDCl_3$) δ 8.06–7.91 (m, 4H), 7.73 (s, 1H), 7.39 (d, *J* = 8.0 Hz, 2H), 2.48 (s, 3H) ppm. – ^{13}C NMR (126 MHz, $CDCl_3$) δ 145.0, 144.5, 137.9, 136.8, 135.6, 129.8, 127.5, 125.1, 21.7 ppm. – IR: 3075, 1596, 1570, 1423, 1347, 1302, 1287, 1165, 1147, 1108, 1084, 866 cm^{-1}. – HRMS: calcd for $C_{13}H_{10}Cl_3NO_3S_2$: 414.9506, found 414.9505 [$M+NH_4^+$].

3-Chloro-4-fluoro-*N*-tosylbenzenesulfonimidoyl chloride (*rac*-1r)

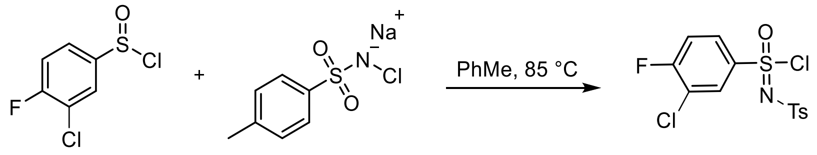

According to GP1, the reaction was carried out with 3-chloro-4-fluorobenzenesulfinic chloride (530 mg, 2.5 mmol) prepared from 3-chloro-4-fluorobenzenesulfonyl chloride (684 mg, 3.0 mmol), and chloramine-T (569 mg, 2.5 mmol) in toluene (12 mL). After the completion of the reaction, it was then filtered through celite with toluene. The filtrate was collected, the solvent was removed under reduced pressure, and the remaining material was purified by flash chromatography on silica gel (EtOAc/hexane, 3 : 7 v/v) to give product ***rac*-1r** (553 mg, 54%) as a white solid.

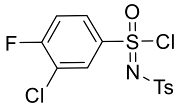

m.p.: 35–38 °C. – ^{1}H NMR (500 MHz, $CDCl_3$) δ 8.21–8.11 (m, 1H), 8.08–7.91 (m, 3H), 7.46–7.33 (m, 3H), 2.48 (s, 3H) ppm. – ^{13}C NMR (126 MHz, $CDCl_3$) δ 162.3 (d, *J* = 262.7 Hz), 144.9, 139.0 (d, *J* = 3.9 Hz), 138.0, 130.1, 129.8, 128.0 (d, *J* = 9.2 Hz), 127.5, 123.6 (d, *J* = 19.2 Hz), 118.1 (d, *J* = 23.0 Hz), 21.7 ppm. – ^{19}F NMR (471 MHz, $CDCl_3$) δ –100.8 ppm. – IR: 2926, 139, 1588, 1465, 1415, 1347, 1262, 1230, 1205, 1162, 1126, 1085, 1042, 949, 813, 735 cm^{-1}. – HRMS: calcd for $C_{13}H_{10}Cl_2FNO_3S_2$: 403.9355, found 403.9357 [M+Na$^+$].

4-Fluoro-*N*-tosyl-3-(trifluoromethyl)benzenesulfonimidoyl chloride (*rac*-1s)

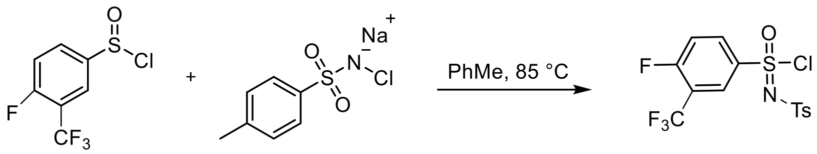

According to GP1, the reaction was carried out with 4-fluoro-3-(trifluoromethyl)benzenesulfinic chloride (615 mg, 2.5 mmol) prepared from 4-fluoro-3-(trifluoromethyl)benzenesulfonyl chloride (786 mg, 3.0 mmol), and chloramine-T (569 mg, 2.5 mmol) in toluene (12 mL). After the completion of the reaction, it was then filtered through celite with toluene. The filtrate was collected, the solvent was removed under reduced pressure, and the remaining material was purified by flash chromatography on silica gel (EtOAc/hexane, 3 : 7 v/v) to give product ***rac*-1s** (617 mg, 65%) as a white solid.

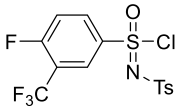

m.p.: 71–73 °C. – ^{1}H NMR (500 MHz, $CDCl_3$) δ 8.34 (d, *J* = 5.2 Hz, 2H), 7.98 (d, *J* = 7.5 Hz, 2H), 7.51 (t, *J* = 9.0 Hz, 1H), 7.39 (d, *J* = 7.9 Hz, 2H), 2.48 (s, 3H) ppm. – ^{13}C NMR (126 MHz, $CDCl_3$) δ 163.6 (d, *J* = 270.8 Hz), 145.0, 138.8 (d, *J* = 3.8 Hz), 137.9, 133.4 (d, *J* = 10.7 Hz), 129.9, 127.5, 127.2 (q, *J* = 4.4 Hz), 121.0 (d, *J* = 273.7 Hz), 120.6 (dd, *J* = 35.0, 14.1 Hz), 119.2 (d, *J* = 22.6 Hz), 21.7 ppm. – ^{19}F NMR (471 MHz, $CDCl_3$) δ –61.9 (d, *J* = 12.7 Hz), –100.0 (ddd, *J* = 18.3, 9.5, 5.2 Hz) ppm. – IR: 3074, 1617, 1586, 1489, 1422, 1345, 1321, 1289, 1250, 1146, 1111, 1084, 1055, 907 cm^{-1}. – HRMS: calcd for $C_{14}H_{10}ClF_4NO_3S_2$: 433.0065, found 433.0055 [M+NH$_4^+$].

4-Chloro-*N*-tosyl-3-(trifluoromethyl)benzenesulfonimidoyl chloride (*rac*-1t)

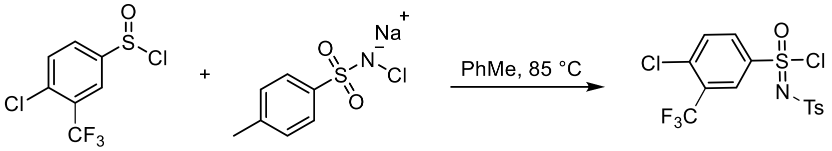

According to GP1, the reaction was carried out with 4-chloro-3-(trifluoromethyl)benzenesulfinic chloride (655 mg, 2.5 mmol) prepared from 4-chloro-3-(trifluoromethyl)benzene sulfonyl chloride (834 mg, 3.0 mmol), and chloramine-T (569 mg, 2.5 mmol) in toluene (12 mL). After the completion of the reaction, it was then filtered through celite with toluene. The filtrate was collected, the solvent was removed under reduced pressure, and the remaining material was purified by flash chromatography on silica gel (EtOAc/hexane, 3 : 7 v/v) to give product ***rac*-1t** (671 mg, 63%) as a white solid.

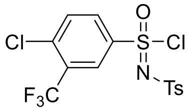

m.p.: 50–52 °C. – ^{1}H NMR (500 MHz, $CDCl_3$) δ 8.34 (s, 1H), 8.20 (d, *J* = 8.6 Hz, 1H), 7.98 (d, *J* = 7.8 Hz, 2H), 7.81 (d, *J* = 8.7 Hz, 1H), 7.39 (d, *J* = 8.0 Hz, 2H), 2.48 (s, 3H) ppm. – ^{13}C NMR (126 MHz, $CDCl_3$) δ 145.0, 141.2, 140.8, 137.9, 133.3, 131.0, 130.5 (q, *J* = 33.4 Hz), 129.9, 127.5, 126.3 (q, *J* = 5.6 Hz),121.4 (q, *J* = 274.6 Hz), 21.7 ppm. – IR: 3100, 1595, 1567, 1467, 1405, 1345, 1290, 1164, 1120, 1106, 1082, 1033, 906 cm^{-1}. – HRMS: calcd for $C_{14}H_{10}Cl_2F_3NO_3S_2$: 431.9504, found 431.9499 [M+H$^+$].

4-Bromo-3-methyl-*N*-tosylbenzenesulfonimidoyl chloride (*rac*-1u)

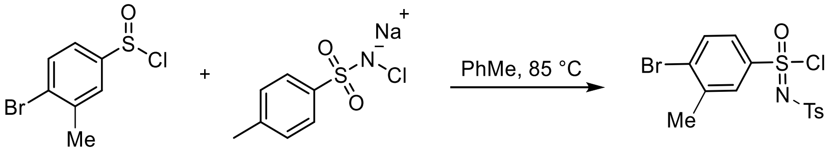

According to GP1, the reaction was carried out with 4-bromo-3-methylbenzenesulfinic chloride (630 mg, 2.5 mmol) prepared from 4-bromo-3-methylbenzenesulfonyl chloride (804 mg, 3.0 mmol), and chloramine-T (569 mg, 2.5 mmol) in toluene (12 mL). After the completion of the reaction, it was then filtered through celite with toluene. The filtrate was collected, the solvent was removed under reduced pressure, and the remaining material was purified by flash chromatography on silica gel (EtOAc/hexane, 3 : 7 v/v) to give product ***rac*-1u** (663 mg, 63%) as a pale yellow solid.

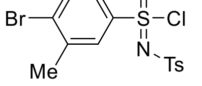

m.p.: 70–73 °C. – ^{1}H NMR (500 MHz, $CDCl_3$) δ 7.98 (d, *J* = 8.0 Hz, 2H), 7.92 (s, 1H), 7.83–7.70 (m, 2H), 7.37 (d, *J* = 8.1 Hz, 2H), 2.53 (s, 3H), 2.47 (s, 3H) ppm. – ^{13}C NMR (126 MHz, $CDCl_3$) δ 144.6, 141.3, 140.8, 138.2, 133.8, 133.8, 129.7, 128.3, 127.5, 125.3, 23.3, 21.7 ppm. – IR: 2921, 1596, 1556, 1463, 1340, 1283, 1163, 1110, 1081, 1027, 877 cm^{-1}. – HRMS: calcd for $C_{14}H_{13}BrClNO_3S_2$: 421.9282, found 421.9280 [M+H$^+$].

***N*-Tosylnaphthalene-2-sulfonimidoyl chloride (*rac*-1v)**

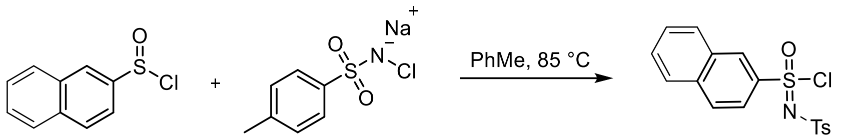

According to GP1, the reaction was carried out with naphthalene-2-sulfinic chloride (525 mg, 2.5 mmol) prepared from naphthalene-2-sulfonyl chloride (678 mg, 3.0 mmol), and chloramine-T (569 mg, 2.5 mmol) in toluene (12 mL). After the completion of the reaction, it was then filtered through celite with toluene. The filtrate was collected, the solvent was removed under reduced pressure, and the remaining material was purified by flash chromatography on silica gel (EtOAc/hexane, 3 : 7 v/v) to give product ***rac*-1v** (455 mg, 48%) as a pale yellow solid.

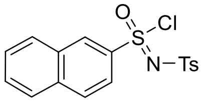

m.p.: 56–58 °C. – ^{1}H NMR (500 MHz, $CDCl_3$) δ 8.66 (d, *J* = 2.1 Hz, 1H), 8.10–7.93 (m, 6H), 7.78 (ddd, *J* = 8.2, 6.9, 1.3 Hz, 1H), 7.72 (ddd, *J* = 8.2, 6.9, 1.3 Hz, 1H), 7.39 (d, *J* = 8.1 Hz, 2H), 2.47 (s, 3H) ppm. – ^{13}C NMR (126 MHz, $CDCl_3$) δ 144.5, 139.2, 138.5, 135.9, 131.6, 130.8, 130.5, 130.0, 129.7, 129.1, 128.6, 128.2, 127.6, 120.9, 21.7 ppm. – IR: 2969, 1738, 1595, 1456, 1384, 1230, 1205, 1152, 1108, 1087, 895, 815, 733 cm^{-1}. – HRMS: calcd for $C_{17}H_{14}ClNO_3S_2$: 380.0176, found 380.0172 [M+H$^+$].

***N*-((4-Fluorophenyl)sulfonyl)benzenesulfonimidoyl chloride (*rac*-1w)**

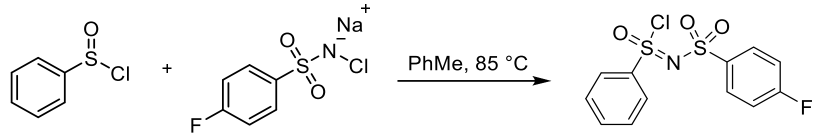

According to GP2, the reaction was carried out with benzenesulfinic chloride (359 mg, 2.24 mmol), and sodium chloro((4-fluorophenyl) sulfonyl)amide (518 mg, 2.24 mmol) prepared from (4-fluorophenyl)sulfonamide (434 mg, 2.5 mmol) in toluene (12 mL). After the completion of the reaction, it was then filtered through celite with toluene. The filtrate was collected, the solvent was removed under reduced pressure, and the remaining material was purified by flash chromatography on silica gel (EtOAc/hexane, 3 : 7 v/v) to give product ***rac*-1w** (470 mg, 63%) as a pale yellow solid.

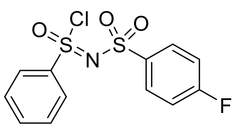

m.p.: 52–54 °C. – ^{1}H NMR (500 MHz, $CDCl_3$) δ 8.17–8.05 (m, 4H), 7.79 (t, *J* = 7.5 Hz, 1H), 7.71–7.62 (m, 2H), 7.31–7.22 (m, 2H) ppm. – ^{13}C NMR (126 MHz, $CDCl_3$) δ 165.6 (d, *J* = 256.0 Hz), 142.5, 137.3 (d, *J* = 3.3 Hz), 135.8, 130.4 (d, *J* = 9.3 Hz), 129.9, 126.8, 116.4 (d, *J* = 22.7 Hz) ppm. – ^{19}F NMR (471 MHz, $CDCl_3$) δ –103.70 ppm. – IR: 2922, 2851, 1738, 1661, 1589, 1494, 1456, 1346, 1290, 1231, 1125, 1087, 1042, 997, 838, 738 cm^{-1}. – HRMS: calcd for $C_{12}H_9ClFNO_3S_2$: 355.9589, found 355.9577 [M+Na$^+$].

N-((3-Fluorophenyl)sulfonyl)benzenesulfonimidoyl chloride (*rac*-1x)

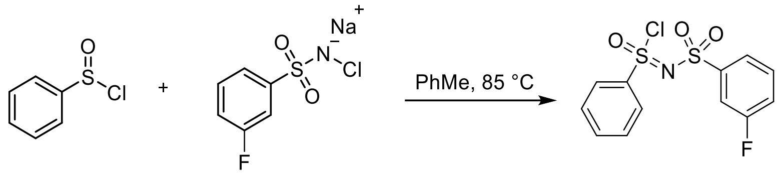

According to GP2, the reaction was carried out with benzenesulfinic chloride (359 mg, 2.24 mmol), and sodium chloro((3-fluorophenyl)sulfonyl)amide (518 mg, 2.24 mmol) prepared from (3-fluorophenyl)sulfonamide (436 mg, 2.5 mmol) in toluene (12 mL). After the completion of the reaction, it was then filtered through celite with toluene. The filtrate was collected, the solvent was removed under reduced pressure, and the remaining material was purified by flash chromatography on silica gel (EtOAc/hexane, 3 : 7 v/v) to give product ***rac*-1x** (470 mg, 63%) as a pale yellow solid.

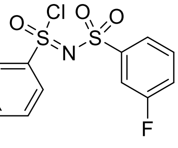

m.p.: 39–41 °C. – ^{1}H NMR (500 MHz, $CDCl_3$) δ 8.09 (d, J = 8.5 Hz, 2H), 7.91 (d, J = 7.8 Hz, 1H), 7.80 (t, J = 7.0 Hz, 2H), 7.66 (t, J = 7.8 Hz, 2H), 7.61–7.52 (m, 1H), 7.41–7.31 (m, 1H) ppm. – ^{13}C NMR (126 MHz, $CDCl_3$) δ 162.3 (d, J = 251.7 Hz), 143.1 (d, J = 7.1 Hz), 142.4, 135.8, 130.9 (d, J = 7.5 Hz), 129.9, 126.8, 123.2 (d, J = 3.5 Hz), 120.7 (d, J = 21.1 Hz), 114.9 (d, J = 24.7 Hz) ppm. – ^{19}F NMR (471 MHz, $CDCl_3$) δ –109.39 ppm. – IR: 2939, 1739, 1589, 1456, 1435, 1347, 1230, 1206, 1162, 1125, 1083, 950, 811, 749 cm^{-1}. – HRMS: calcd for $C_{12}H_9ClFNO_3S_2$: 355.9589, found 355.9594 [M+Na$^+$].

N-((3,4-Difluorophenyl)sulfonyl)benzenesulfonimidoyl chloride (*rac*-1y)

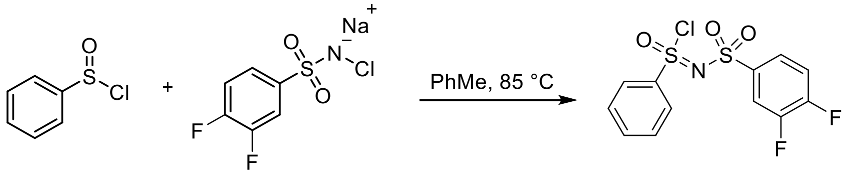

According to GP2, the reaction was carried out with benzenesulfinic chloride (359 mg, 2.24 mmol), and sodium chloro((2,3-difluorophenyl)sulfonyl)amide (558 mg, 2.24 mmol) prepared from (3,4-difluorophenyl)sulfonamide (481 mg, 2.5 mmol) in toluene (12 mL). After the completion of the reaction, it was then filtered through celite with toluene. The filtrate was collected, the solvent was removed under reduced pressure, and the remaining material was purified by flash chromatography on silica gel (EtOAc/hexane, 3 : 7 v/v) to give product ***rac*-1y** (607mg, 69%) as a pale yellow solid.

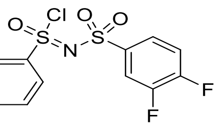

m.p.: 65–67 °C. – ^{1}H NMR (500 MHz, $CDCl_3$) δ 8.09 (d, J = 8.1 Hz, 2H), 8.00–7.87 (m, 2H), 7.81 (t, J = 7.4 Hz, 1H), 7.67 (t, J = 7.8 Hz, 2H), 7.37 (q, J = 8.7 Hz, 1H) ppm. – ^{13}C NMR (126 MHz, $CDCl_3$) δ 153.7 (d, J = 246.1 Hz), 150.1 (d, J = 241.3 Hz), 154.7 (d, J = 12.2 Hz), 152.6 (d, J = 12.5 Hz), 151.1 (d, J = 13.5 Hz), 149.0 (d, J = 13.6 Hz), 142.3, 138.0 (d, J = 4.7 Hz), 135.9, 130.0, 126.8, 124.7 (dd, J = 7.8, 4.1 Hz), 118.2 (d, J = 18.3 Hz), 117.5 (d, J = 20.2 Hz) ppm. – ^{19}F NMR (471 MHz, $CDCl_3$) δ –122.70, –130.23 ppm. – IR: 2924, 1742, 1590, 1508, 1456, 1417, 1347, 1278, 1206, 1161, 1125, 1069, 997, 914, 820, 744 cm^{-1}. – HRMS: calcd for $C_{12}H_8ClF_2NO_3S_2$: 351.9675, found 351.9665 [M+H$^+$].

N-((4-Bromophenyl)sulfonyl)benzenesulfonimidoyl chloride (*rac*-1z)

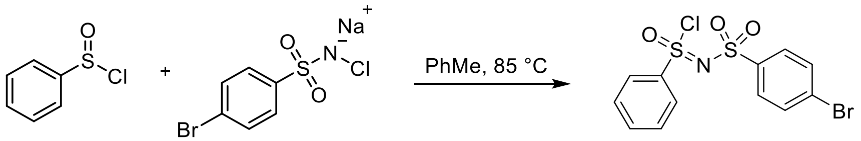

According to GP2, the reaction was carried out with benzenesulfinic chloride (359 mg, 2.24 mmol), and sodium ((4-bromophenyl)sulfonyl)chloroamide (655 mg, 2.24 mmol) prepared from 4-bromobenzenesulfonamide (590 mg, 2.5 mmol) in toluene (12 mL). After the completion of the reaction, it was then filtered through celite with toluene. The filtrate was collected, the solvent was removed under reduced pressure, and the remaining material was purified by

Go to table of contents

flash chromatography on silica gel (EtOAc/hexane, 3 : 7 v/v) to give product ***rac*-1z** (630 mg, 71%) as a pale yellow solid.

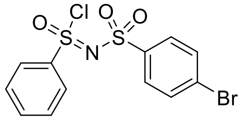

m.p.: 85–88 °C. – ^{1}H NMR (300 MHz, $CDCl_3$) δ 8.13–8.05 (m, 2H), 8.02–7.93 (m, 2H), 7.85–7.76 (m, 1H), 7.76–7.61 (m, 4H) ppm. – ^{13}C NMR (126 MHz, $CDCl_3$) δ 135.1, 134.8, 132.0, 129.5, 128.4, 127.6, 127.5, 76.9, 67.1, 36.7, 21.1 ppm. – IR: 3092, 1738, 1574, 1449, 1390, 1346, 1282, 1167, 1113, 1086, 1067, 1022, 1009, 824, 761 cm^{-1}. – HRMS: calcd for $C_{12}H_9BrClNO_3S_2$: 393.8969, found 393.8973 [M+H$^+$].

N-((3-(Trifluoromethyl)phenyl)sulfonyl)benzenesulfonimidoyl chloride (*rac*-1aa)

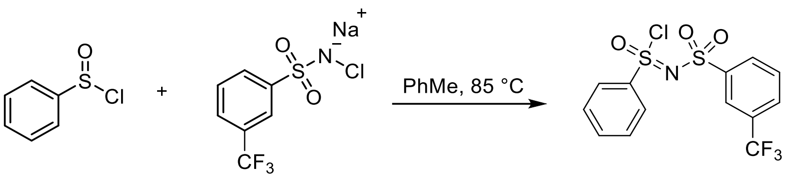

According to GP2, the reaction was carried out with benzenesulfinic chloride (359 mg, 2.24 mmol), and sodium chloro((3-(trifluoromethyl)phenyl)sulfonyl)amide (630 mg, 2.24 mmol) prepared from ((3-(trifluoromethyl)phenyl)sulfonamide (590 mg, 2.5 mmol) in toluene (12 mL). After the completion of the reaction, it was then filtered through celite with toluene. The filtrate was collected, the solvent was removed under reduced pressure, and the remaining material was purified by flash chromatography on silica gel (EtOAc/hexane, 3 : 7 v/v) to give product ***rac*-1aa** (515 mg, 60%) as a pale yellow solid.

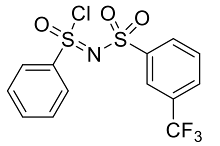

m.p.: 38–40 °C. – ^{1}H NMR (500 MHz, $CDCl_3$) *δ* 8.37 (s, 1H), 8.31 (d, *J* = 7.6 Hz, 1H), 8.09 (d, *J* = 8.0 Hz, 2H), 7.91 (d, *J* = 7.8 Hz, 1H), 7.81 (t, *J* = 7.5 Hz, 1H), 7.74 (t, *J* = 7.9 Hz, 1H), 7.67 (t, *J* = 7.8 Hz, 2H) ppm. – ^{13}C NMR (126 MHz, $CDCl_3$) *δ* 142.4 (d, *J* = 12.2 Hz), 135.9, 131.8 (q, *J* = 33.6 Hz), 130.7, 130.1 (q, *J* = 3.78 Hz), 130.0, 129.9, 126.9, 124.7 (q, *J* = 3.3 Hz), 123.1 (d, *J* = 272.9 Hz) ppm. – ^{19}F NMR (471 MHz, $CDCl_3$) δ –62.8 ppm. – IR: 2969, 1738, 1590, 1456, 1349, 1327, 1230, 1206, 1164, 1127, 895 cm^{-1}. – HRMS: calcd for $C_{13}H_9ClF_3NO_3S_2$: 383.9797, found 383.9734 [M+H$^+$].

N-((3-Chloro-4-methoxyphenyl)sulfonyl)benzenesulfonimidoyl chloride (*rac*-1ab)

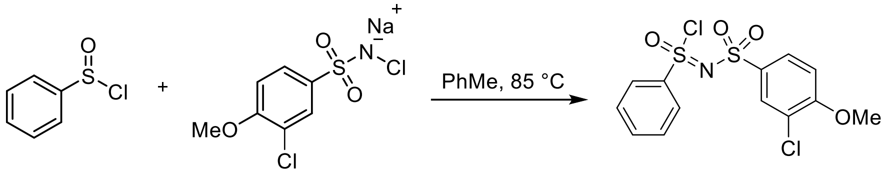

According to GP2, the reaction was carried out with benzenesulfinic chloride (359 mg, 2.24 mmol), and sodium chloro((3-chloro-4-methoxyphenyl)sulfonyl)amide (621 mg, 2.24 mmol) prepared from (3-chloro-4-methoxyphenyl)sulfonamide (494 mg, 2.5 mmol) in toluene (12 mL). After the completion of the reaction, it was then filtered through celite with toluene. The filtrate was collected, the solvent was removed under reduced pressure, and the remaining material was purified by flash chromatography on silica gel (EtOAc/hexane, 3 : 7 v/v) to give product ***rac*-1ab** (577 mg, 69%) as a pale yellow solid.

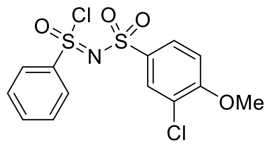

m.p.: 85–87 °C. – ^{1}H NMR (500 MHz, $CDCl_3$) *δ* 8.09 (d, *J* = 8.6 Hz, 3H), 8.01 (d, *J* = 8.7 Hz, 1H), 7.79 (t, *J* = 7.4 Hz, 1H), 7.66 (t, *J* = 7.8 Hz, 2H), 7.06 (d, *J* = 8.7 Hz, 1H), 4.01 (s, 3H) ppm. – ^{13}C NMR (126 MHz, $CDCl_3$) *δ* 159.0, 142.5, 135.7, 133.6, 129.9, 129.6, 128.0, 126.9, 123.3, 111.5, 56.6 ppm. – IR: 2923, 1738, 1584, 1490, 1449, 1343, 1276, 1166, 1096, 1062, 1018, 850, 738 cm^{-1}. – HRMS: calcd for $C_{13}H_{11}Cl_2NO_4S_2$: 401.9399, found 401.9402 [M+Na$^+$].

N-(Cyclopropylsulfonyl)benzenesulfonimidoyl chloride (*rac*-1ac)

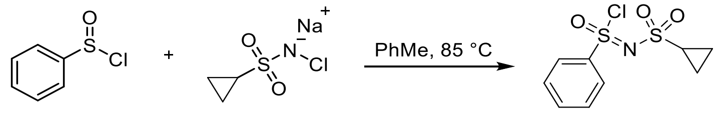

According to GP2, the reaction was carried out with benzenesulfinic chloride (359 mg, 2.24 mmol), and sodium chloro(cyclopropylsulfonyl)amide (397 mg, 2.24 mmol) prepared from (cyclopropylsulfonyl)amide (301 mg, 2.5 mmol) in toluene (12 mL). After the completion of the reaction, it was then filtered through celite with toluene. The filtrate was collected, the solvent was removed under reduced pressure, and the remaining material was purified by flash chromatography on silica gel (EtOAc/hexane, 3 : 7 v/v) to give product ***rac*-1ac** (456 mg, 73 %) as a pale yellow solid.

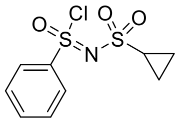

m.p.: 33–35 °C. – ^{1}H NMR (500 MHz, $CDCl_3$) δ 8.22–8.13 (m, 2H), 7.82 (t, J = 7.4 Hz, 1H), 7.69 (t, J = 7.9 Hz, 2H), 2.94–2.79 (m, 1H), 1.55–1.40 (m, 2H), 1.22–1.12 (m, 2H) ppm. – ^{13}C NMR (126 MHz, $CDCl_3$) δ 142.6, 135.7, 129.9, 127.0, 34.1, 6.8, 6.7 ppm. – IR: 2922, 2851, 1660, 1449, 1337, 1286, 1152, 1115, 1071, 1022, 886, 744 cm^{-1}. – HRMS: calcd for $C_9H_{10}ClNO_3S_2$: 279.9863, found 279.9857 [M+H$^+$].

Kinetic resolution products

3-Hydroxy-2,2-dimethylpropyl *(R)-N*-tosylbenzenesulfonimidate (3a)

***(S)-N*-Tosylbenzenesulfonimidoyl chloride (1a)**

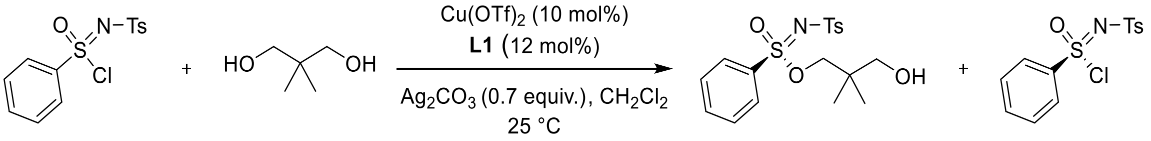

According to GP3, the reaction was carried out with sulfonimidoyl chloride ***rac*-1a** (33 mg, 0.1 mmol), and diol **2** (17.7 mg, 0.17 mmol) in anhydrous dichloromethane (1 mL). After stirring for 12.5 h, for work-up, a saturated solution of EDTA (1.5 mL) was added, followed by dichloromethane (5 mL). The organic phases were combined, dried over anhydrous Na_2SO_4, concentrated under reduced pressure, and the remaining material was purified by preparative TLC (hexane/ethyl acetate, 80 : 20 – 60 : 40) to give enantioenriched sulfonimidate product **3a** (17.9 mg, 45%, 90% ee) and sulfonimidoyl chloride **1a** (13.2 mg, 40%, 97% ee).

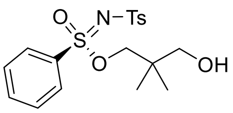

$[\alpha]_D$ = –15.0 (c 0.16, $CHCl_3$). – [Daicel Chiralpak IBN (0.46 cm × 25 cm); 90 : 10 v/v, *n*-hexane / isopropanol; v = 1.2 mL·min^{-1}, λ = 210 nm, t_R (major) = 15.98 min, t_R (minor) = 19.44 min]. – ^{1}H NMR (500 MHz, $CDCl_3$) δ 7.94 (d, J = 8.3 Hz, 2H), 7.86 (d, J = 8.1 Hz, 2H), 7.69 (t, J = 7.3 Hz, 1H), 7.56 (t, J = 7.7 Hz, 2H), 7.27 (d, J = 8.1 Hz, 2H), 4.08–3.96 (m, 2H), 3.42 (s, 2H), 2.40 (s, 3H), 0.89 (s, 6H) ppm. – ^{13}C NMR (126 MHz, $CDCl_3$) δ 143.3, 139.8, 135.3, 134.6, 129.4, 129.3, 127.6, 126.8, 76.7, 67.1, 36.7, 21.5, 21.1 ppm. – IR: 3522, 2969, 1738, 1473, 1448, 1365, 1320, 1275, 1155, 1119, 1089, 913, 852, 734 cm^{-1}. – HRMS: calcd for $C_{18}H_{23}NO_5S_2$: 397.1018, found 397.1014 [M+H$^+$].

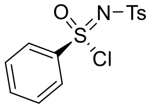

$[\alpha]_D$ = +169.8 (c 0.55, $CHCl_3$). – [Daicel Chiralpak IH (0.46 cm × 25 cm); 85 : 15 v/v, *n*-hexane / isopropanol; v = 1.2 mL·min^{-1}, λ = 254 nm, t_R (major) = 34.83 min, t_R (minor) = 28.64 min]. – m.p.: 35–37 °C. – ^{1}H NMR (300 MHz, $CDCl_3$) δ 8.11–8.04 (m, 2H), 7.99 (d, J = 8.3 Hz, 2H), 7.78 (t, J = 7.5 Hz, 1H), 7.64 (t, J = 7.8 Hz, 2H), 7.37 (d, J = 8.0 Hz, 2H), 2.46 (s, 3H) ppm. – ^{13}C NMR (126 MHz, $CDCl_3$) δ 144.6, 142.6, 138.3, 135.6, 129.8, 129.7, 127.5, 126.9, 21.7 ppm. – IR: 2970, 1738, 1597, 1449, 1365, 1342, 1287, 1163, 1114, 1087, 896, 734 cm^{-1}. – HRMS: calcd for $C_{13}H_{12}ClNO_3S_2$: 328.9947, found 328.9942 [M+H$^+$].

3-Hydroxy-2,2-dimethylpropyl *(R)*-2-fluoro-*N*-tosylbenzenesulfonimidate (3b)

***(S)*-2-Fluoro-*N*-tosylbenzenesulfonimidoyl chloride (1b)**

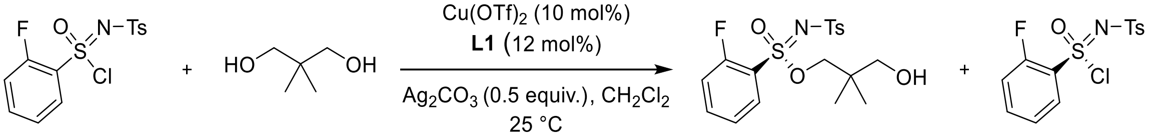

According to GP3, the reaction was carried out with sulfonimidoyl chloride ***rac*-1b** (34.7mg, 0.1 mmol), and diol **2** (17.7 mg, 0.17 mmol) in anhydrous dichloromethane (1 mL). After stirring for 12.5 h, for work-up, a saturated solution of EDTA (1.5 mL) was added, followed by dichloromethane (5 mL). The organic phases were combined, dried over anhydrous Na_2SO_4, concentrated under reduced pressure, and the remaining material was purified by preparative TLC (hexane/ethyl acetate, 80: 20 – 60: 40) to give enantioenriched sulfonimidate product **3b** (17.8 mg, 43%, 97% ee) and sulfonimidoyl chloride **1b** (13.8 mg, 40%, 99% ee).

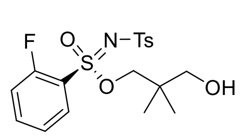

$[\alpha]_D$ = –46.8 (c .31, $CHCl_3$). – [Daicel Chiralpak IBN (0.46 cm × 25 cm); 90 : 10 v/v, *n*-hexane / isopropanol; v = 1.2 mL·min^{-1}, λ = 254 nm, t_R (major) = 20.48 min, t_R (minor) = 23.85 min]. – ^{1}H NMR (500 MHz, $CDCl_3$) δ 8.04–7.95 (m, 1H), 7.86 (d, *J* = 8.3 Hz, 2H), 7.74–7.66 (m, 1H), 7.34 (t, *J* = 7.7 Hz, 1H), 7.30–7.20 (m, 3H), 4.21 (d, *J* = 9.0 Hz, 1H), 4.14 (d, *J* = 9.0 Hz, 1H), 3.48 (d, *J* = 11.3 Hz, 1H), 3.42 (d, *J* = 11.3 Hz, 1H), 2.42 (s, 3H), 0.94 (d, *J* = 8.3 Hz, 6H) ppm. – ^{13}C NMR (126 MHz, $CDCl_3$) δ 159.1 (d, *J* = 261.1 Hz), 143.4, 139.6, 137.1 (d, *J* = 8.7 Hz), 131.0, 129.3, 126.8, 124.6 (d, *J* = 3.9 Hz), 123.9 (d, *J* = 12.8 Hz), 117.7 (d, *J* = 20.4 Hz), 77.1, 67.1, 36.7, 21.6, 21.0 ppm. – ^{19}F NMR (471 MHz, $CDCl_3$) δ –106.39. – IR: 3522, 2923, 1598, 1476, 1373, 1285, 1156, 1114, 1090, 1073, 937 cm^{-1}. – HRMS: calcd for $C_{18}H_{22}FNO_5S_2$: 416.0996, found 416.0984 [M+H$^+$].

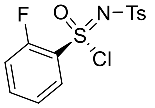

$[\alpha]_D$ = +33.9 (c 0.31, $CHCl_3$). – [Daicel Chiralpak IH (0.46 cm × 25 cm); 50 : 50 v/v, *n*-hexane/ isopropanol; v = 1.2 mL·min^{-1}, λ = 254 nm, t_R (major) = 9.62 min, t_R (minor) = 10.82 min]. – m.p.: 70–73 °C. – ^{1}H NMR (500 MHz, $CDCl_3$) δ 8.09–8.00 (m, 1H), 7.98 (d, *J* = 8.0 Hz, 2H), 7.83–7.74 (m, 1H), 7.41–7.32 (m, 4H), 2.47 (s, 3H) ppm. – ^{13}C NMR (126 MHz, $CDCl_3$) δ 158.6 (d, *J* = 265.0 Hz), 144.6, 138.2, 138.2 (d, *J* = 8.9 Hz), 130.3 (d, *J* = 11.7 Hz), 129.7, 129.3, 127.5, 126.5, 124.7 (d, *J* = 4.1 Hz), 21.7 ppm. – ^{19}F NMR (471 MHz, $CDCl_3$) δ –104.41 ppm. – IR: 2925, 1594, 1478, 1342, 1291, 1264, 1186, 1131, 1110, 1087, 1062, 830, 814, 734 cm^{-1}. – HRMS: calcd for $C_{13}H_{11}ClFNO_3S_2$: 347.9926, found 347.9918 [M+H$^+$].

3-Hydroxy-2,2-dimethylpropyl *(R)*-4-chloro-*N*-tosylbenzenesulfonimidate (3c)

***(S)*-4-Chloro-*N*-tosylbenzenesulfonimidoyl chloride (1c)**

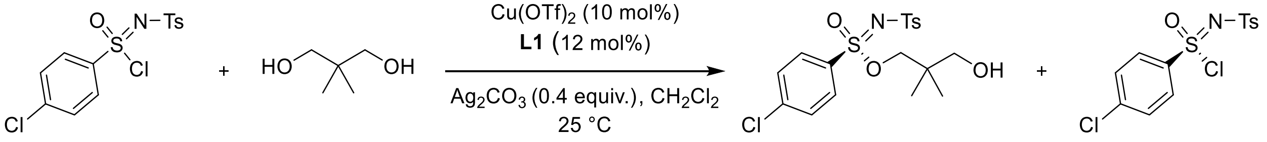

According to GP3, the reaction was carried out with sulfonimidoyl chloride ***rac*-1c** (36.4 mg, 0.1 mmol), and diol **2** (17.7 mg, 0.17 mmol) in anhydrous dichloromethane (1 mL). After stirring for 13 h, for work-up, a saturated solution of EDTA (1.5 mL) was added, followed by dichloromethane (5 mL). The organic phases were combined, dried over anhydrous Na_2SO_4, concentrated under reduced pressure, and the remaining material was purified by preparative TLC (hexane/ethyl acetate, 80 : 20 – 60 : 40) to give enantioenriched sulfonimidate product **3c** (18.9 mg, 44%, 91% ee) and sulfonimidoyl chloride **1c** (14.5 mg, 40%, 95% ee).

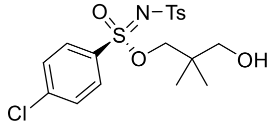

$[\alpha]_D$ = –13.2 (c 0.31, $CHCl_3$). – [Daicel Chiralpak IC (0.46 cm × 25 cm); 50 : 50 v/v, *n*-hexane / isopropanol; v = 1.2 mL·min^{-1}, λ = 254 nm, t_R (major) = 8.80 min, t_R (minor) = 11.83 min]. – ^{1}H NMR (500 MHz, $CDCl_3$) δ 7.88 (dd, *J* = 15.8, 8.1 Hz, 4H), 7.54 (d, *J* = 8.3 Hz, 2H), 7.38 (d, *J* = 1.2 Hz, 1H), 7.29 (d, *J* = 7.2 Hz, 1H), 4.06 (q, *J* = 8.9 Hz, 2H), 3.44 (s, 2H), 2.43 (s, 3H), 0.92 (d, *J* = 2.2 Hz, 6H) ppm. – ^{13}C NMR (126 MHz, $CDCl_3$) δ 143.5, 141.5, 139.6, 133.9, 129.8, 129.4, 129.1, 128.3, 126.8, 126.5,

76.9, 67.1, 36.7, 21.6, 21.1 ppm. – IR: 3521, 3091, 2963, 1738, 1597, 1474, 1319, 1273, 1155, 1121, 1084, 1011, 918 cm^{-1}. – HRMS: calcd for $C_{19}H_{22}ClNO_5S_2$: 432.0701, found 432.0698 [$M+H^+$].

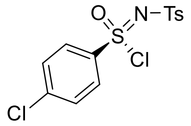

$[\alpha]_D$ = +18.2 (c 0.11, $CHCl_3$). – [Daicel Chiralpak IF (0.46 cm × 25 cm); 70 : 30 v/v, *n*-hexane / isopropanol; v = 1.2 mL·min^{-1}, λ = 254 nm, t_R (major) = 9.01 min, t_R (minor) = 6.79 min]. – m.p.: 71–73 °C. – ^1H NMR (500 MHz, $CDCl_3$) δ 8.02 (d, J = 8.5 Hz, 2H), 7.98 (d, J = 8.0 Hz, 2H), 7.61 (d, J = 8.6 Hz, 2H), 7.38 (d, J = 7.8 Hz, 2H), 2.47 (s, 3H) ppm. ^{13}C NMR (126 MHz, $CDCl_3$) δ 144.7, 142.7, 140.9, 138.2, 130.1, 129.8, 128.3, 127.5, 126.5, 21.7 ppm. – IR: 3092, 2923, 1596, 1573, 1471, 1396, 1339, 1278, 1162, 1113, 1083, 1009 cm^{-1}. – HRMS: calcd for $C_{13}H_{11}Cl_2NO_3S_2$: 401.9189, found 401.9182 [$M+K^+$].

3-Hydroxy-2,2-dimethylpropyl *(R)*-4-bromo-*N*-tosylbenzenesulfonimidate (3d)

***(S)*-4-Bromo-*N*-tosylbenzenesulfonimidoyl chloride (1d)**

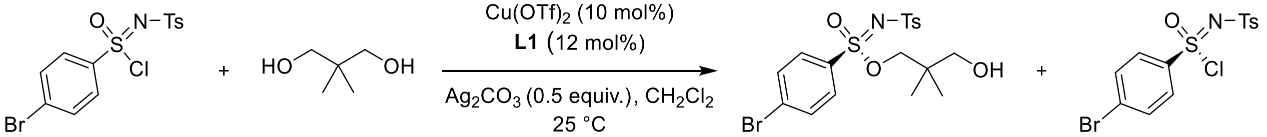

According to GP3, the reaction was carried out with sulfonimidoyl chloride ***rac*-1d** (40.8 mg, 0.1 mmol), and diol **2** (17.7 mg, 0.17 mmol) in anhydrous dichloromethane (1 mL). After stirring for 13 h, for work-up, a saturated solution of EDTA (1.5 mL) was added, followed by dichloromethane (5 mL). The organic phases were combined, dried over anhydrous Na_2SO_4, concentrated under reduced pressure, and the remaining material was purified by preparative TLC (hexane/ethyl acetate, 80 : 20 – 60 : 40) to give enantioenriched sulfonimidate product **3d** (21.4 mg, 45%, 90% ee) and sulfonimidoyl chloride **1d** (17.1 mg, 42%, 98% ee).

Gram scale synthesis and the recovery of silver(I) and *ent*-L1: To a 250 mL round bottom flask equipped with a magnetic stir bar, ligand *ent*-**L1** (429 mg, 0.77 mmol, 12 mol%), $Cu(OTf)_2$ (230 mg, 0.64 mmol, 10 mol%), 3Å molecular sieves (2.56 g) and anhydrous dichloromethane (64 mL) were added in a glovebox. After stirring for 30 min, diol **2** (1.13 g, 10.88 mmol, 1.7 equiv.), sulfonimidoyl chloride *rac*-**1d** (2.61 g, 6.40 mmol), and silver carbonate (1.24 g, 0.7 equiv.) were added sequentially. The reaction mixture was then stirred at 450 rpm for 13 h at 25 °C.

For work-up, 30 ml of water was added to quench the reaction. The reaction mixture was then extracted with dichloromethane (3 × 20 mL). The organic phases were combined, dried over anhydrous Na_2SO_4, concentrated under reduced pressure, and the remaining material was purified by column chromatography (hexane/ethyl acetate, 90 : 10, 85 : 15 and 60 : 40) to give the recovered ligand *ent*-**L1** (412 mg, 96%), enantioenriched sulfonimidate *ent*-**3d** product (1.40 g, 46%, 99% ee) and sulfonimidoyl chloride *ent*-**1d** (1.03 g, 40%, 99% ee).

The aqueous phase was first treated with concentrated (28–30%) aqueous ammonia (10 ml) followed by filtration to remove the molecular sieves. The filtrate was acidified to pH 6 with 12M hydrochloric acid and stirred for 30 minutes. The solid silver(I) chloride was recovered as a colorless solid by filtration and dried in an oven at 90 °C for 1 hour (1.19 g, 93%).

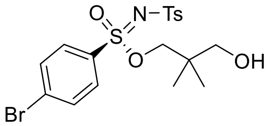

3d: $[\alpha]_D$ = –10.0 (c 3.5, $CHCl_3$). – [Daicel Chiralpak IF (0.46 cm × 25 cm); 70 : 30 v/v, *n*-hexane / isopropanol; v = 1.2 mL·min^{-1}, λ = 254 nm, t_R (major) = 9.79 min, t_R (minor) = 7.38 min]. – ^1H NMR (500 MHz, $CDCl_3$) δ 7.83 (dd, J = 23.5, 7.6 Hz, 4H), 7.71 (d, J = 7.9 Hz, 2H), 7.29 (d, J = 6.7 Hz, 2H), 4.06 (q, J = 8.9 Hz, 2H), 3.44 (s, 2H), 2.43 (s, 3H), 0.92 (s, 6H) ppm. – ^{13}C NMR (126 MHz, $CDCl_3$) δ 143.5, 139.6, 134.5, 132.8, 130.1, 129.7, 129.4, 129.1, 128.3, 126.8, 126.5, 76.9, 67.1, 36.8, 21.6, 21.1 ppm. – IR: 3526, 3090, 2962, 1738, 1572, 1472, 1320, 1289, 115, 1119, 1089,1068, 1008, 918 cm^{-1}. – HRMS: calcd for $C_{18}H_{22}BrNO_5S_2$: 476.0196, found 476.0193 [$M+H^+$].

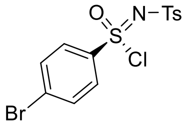

1d: $[\alpha]_D$ = +9.1 (c 2.3, $CHCl_3$). – [Daicel Chiralpak IH (0.46 cm × 25 cm); 50 : 50 v/v, *n*-hexane / isopropanol; v = 1.2 mL·min^{-1}, λ = 254 nm, t_R (major) = 8.73 min, t_R (minor) = 7.2 min]. – m.p.: 97–99 °C. – ^1H NMR (500 MHz, $CDCl_3$) δ 7.98 (d, J = 7.5 Hz, 2H), 7.93 (d, J = 7.6 Hz, 2H), 7.77 (d, J = 8.0 Hz, 2H), 7.38 (d, J = 7.8 Hz, 2H), 2.47 (s, 3H) ppm. – ^{13}C NMR (126 MHz, $CDCl_3$) δ 144.7, 141.5, 138.2,

133.2, 131.4, 129.8, 128.3, 127.5, 126.5, 21.7 ppm. – IR: 3092, 2922, 2852, 1596, 1566, 1468, 1391, 1339, 1277, 1163, 1112, 1087, 1066, 1005 cm^{-1}. – HRMS: calcd for $C_{13}H_{11}BrClNO_3S_2$: 445.8684, found 445.8674 [M+K$^+$].

3-Hydroxy-2,2-dimethylpropyl *(R)*-4-iodo-*N*-tosylbenzenesulfonimidate (3e)

(*S*)-4-Iodo-*N*-tosylbenzenesulfonimidoyl chloride (1e)

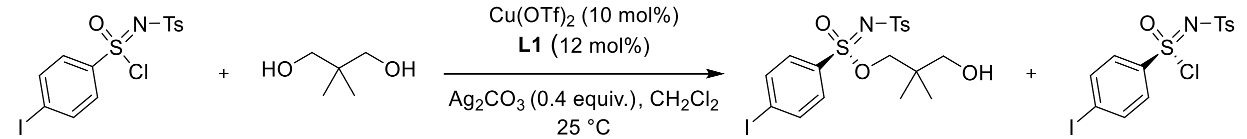

According to GP3, the reaction was carried out with sulfonimidoyl chloride ***rac*-1e** (45.5 mg, 0.1 mmol), and diol **2** (17.7 mg, 0.17 mmol) in anhydrous dichloromethane (1 mL). After stirring for 12 h, for work-up, a saturated solution of EDTA (1.5 mL) was added, followed by dichloromethane (5 mL). The organic phases were combined, dried over anhydrous Na_2SO_4, concentrated under reduced pressure, and the remaining material was purified by preparative TLC (hexane/ethyl acetate, (80 : 20 – 60 : 40) to give enantioenriched sulfonimidate product **3e** (21.4 mg, 41%, 91% ee) and sulfonimidoyl chloride **1e** (18.7 mg, 41%, 99% ee).

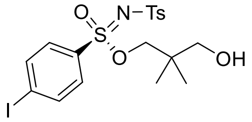

$[\alpha]_D$ = –27.1 (c 0.23, $CHCl_3$). – [Daicel Chiralpak IF (0.46 cm × 25 cm); 70 : 30 v/v, *n*-hexane / isopropanol; v = 1.2 mL·min^{-1}, λ = 254 nm, t_R (major) = 11.49 min, t_R (minor) = 8.45 min]. – ^{1}H NMR (500 MHz, $CDCl_3$) δ 7.92 (d, *J* = 8.4 Hz, 2H), 7.85 (d, *J* = 8.1 Hz, 2H), 7.64 (d, *J* = 8.6 Hz, 2H), 7.32–7.24 (m, 2H), 4.06 (q, *J* = 9.0 Hz, 2H), 3.44 (s, 2H), 2.43 (s, 3H), 0.92 (d, *J* = 2.5 Hz, 6H) ppm. – ^{13}C NMR (126 MHz, $CDCl_3$) *δ* 143.5, 139.5, 138.7, 135.1, 129.7, 129.4, 128.8, 126.8, 126.4, 102.8, 76.9, 67.1, 36.7, 21.6, 21.1 ppm. – IR: 3521, 2962, 1738, 1566, 1471, 1386, 1319, 1269, 1155, 1118, 1087, 1054, 1004, 918 cm^{-1}. – HRMS: calcd for $C_{18}H_{22}INO_5S_2$: 524.0057, found 524.0051 [M+H$^+$].

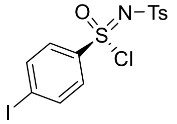

$[\alpha]_D$ = +15.4 (c 0.13, $CHCl_3$). – [Daicel Chiralpak IH (0.46 cm × 25 cm); 50 : 50 v/v, *n*-hexane / isopropanol; v = 1.2 mL·min^{-1}, λ = 254 nm, t_R (major) = 11.23 min, t_R (minor) = 8.04 min]. – m.p.: 117–119 °C. – ^{1}H NMR (500 MHz, $CDCl_3$) *δ* 8.05–7.90 (m, 4H), 7.76 (d, *J* = 8.3 Hz, 2H), 7.37 (d, *J* = 8.1 Hz, 2H), 2.47 (s, 3H) ppm. – ^{13}C NMR (126 MHz, $CDCl_3$) *δ* 144.7, 142.2, 139.2, 138.2, 129.8, 127.9, 127.5, 104.3, 21.7 ppm. – IR: 2969, 1738, 1589, 1456, 1342, 1289, 1230, 1162, 1112, 1086, 1001, 813, 724 cm^{-1}. – HRMS: calcd for $C_{13}H_{11}ClINO_3S_2$: 477.8806, found 477.8814 [M+Na$^+$].

3-Hydroxy-2,2-dimethylpropyl *(R)*-4-nitro-*N*-tosylbenzenesulfonimidate (3f)

(*S*)-4-Nitro-*N*-tosylbenzenesulfonimidoyl chloride (1f)

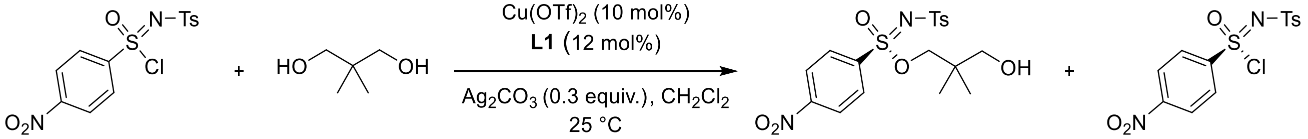

According to GP3, the reaction was carried out with sulfonimidoyl chloride ***rac*-1f** (37.4 mg, 0.1 mmol), and diol **2** (17.7 mg, 0.17 mmol) in anhydrous dichloromethane (1 mL). After stirring for 12 h, for work-up, a saturated solution of EDTA (1.5 mL) was added, followed by dichloromethane (5 mL). The organic phases were combined, dried over anhydrous Na_2SO_4, concentrated under reduced pressure, and the remaining material was purified by preparative TLC (hexane/ethyl acetate, 80 : 20 – 60 : 40) to give enantioenriched sulfonimidate product **3f** (17.7 mg, 41%, 90% ee) and sulfonimidoyl chloride **1f** (13.1 mg, 40%, 98% ee).

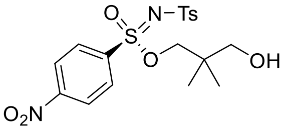

$[\alpha]_D$ = –26.9 (c 0.13, $CHCl_3$). – [Daicel Chiralpak IF (0.46 cm × 25 cm); 70 : 30 v/v, *n*-hexane / isopropanol; v = 1.2 mL·min^{-1}, λ = 254 nm, t_R (major) = 19.75 min, t_R (minor) = 10.37 min]. – ^{1}H NMR (500 MHz, $CDCl_3$) *δ* 8.40 (d, *J* = 8.3 Hz, 2H), 8.17 (d, *J* = 8.3 Hz, 2H), 7.86 (d, *J* = 7.6 Hz, 2H), 7.30 (d, *J* = 7.9 Hz, 2H), 4.20–4.11 (m, 2H), 3.49–3.40 (m, 2H), 2.44 (s, 3H), 0.94

(d, J = 5.3 Hz, 6H) ppm. – ^{13}C NMR (126 MHz, $CDCl_3$) δ 151.1, 143.8, 141.4, 139.3, 129.5, 129.2, 128.3, 126.8, 126.5, 124.6, 77.5, 67.0, 36.8, 21.6, 21.0 ppm. – IR: 3516, 3105, 2967, 1738, 1606, 1530, 1347, 1287, 1157, 1120, 1085, 1011, 918 cm^{-1}. – HRMS: calcd for $C_{18}H_{22}N_2O_7S_2$: 443.0941, found 443.0941 [M+H$^+$].

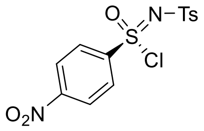

$[\alpha]_D$ = +7.7 (c 0.26, $CHCl_3$). – [Daicel Chiralpak IH (0.46 cm × 25 cm); 50 : 50 v/v, *n*-hexane / isopropanol; v = 1.2 mL·min^{-1}, λ = 254 nm, t_R (major) = 16.28 min, t_R (minor) = 13.78 min]. – m.p.: 95–97 °C. – ^{1}H NMR (500 MHz, $CDCl_3$) δ 8.47 (d, J = 9.0 Hz, 2H), 8.30 (d, J = 9.0 Hz, 2H), 7.99 (d, J = 8.3 Hz, 2H), 7.40 (d, J = 8.2 Hz, 2H), 2.48 (s, 3H) ppm. – ^{13}C NMR (126 MHz, $CDCl_3$) δ 151.4, 147.2, 145.1, 137.9, 129.9, 128.5, 127.5, 125.0, 21.7 ppm. – IR: 2922, 1596, 1532, 1340, 1287, 1163, 1116, 1086, 1069, 1009 cm^{-1}. – HRMS: calcd for $C_{13}H_{11}ClN_2O_3S_2$: 392.0136, found 392.0133 [M+NH$_4^+$].

3-Hydroxy-2,2-dimethylpropyl (*R*)-4-methoxy-*N*-tosylbenzenesulfonimidate (3g)

(*S*)-4-Methoxy-*N*-tosylbenzenesulfonimidoyl chloride (1g)

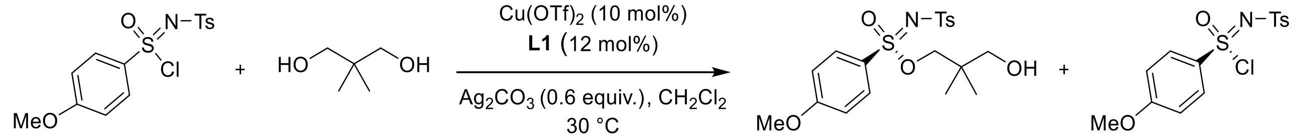

According to GP3, the reaction was carried out with sulfonimidoyl chloride ***rac*-1g** (35.9 mg, 0.1 mmol), and diol **2** (17.7 mg, 0.17 mmol) in anhydrous dichloromethane (1 mL). After stirring for 23 h, for work-up, a saturated solution of EDTA (1.5 mL) was added, followed by dichloromethane (5 mL). The organic phases were combined, dried over anhydrous Na_2SO_4, concentrated under reduced pressure, and the remaining material was purified by preparative TLC (hexane/ethyl acetate, 80: 20 – 60: 40) to give enantioenriched sulfonimidate product **3g** (18.8 mg, 43%, 93% ee) and sulfonimidoyl chloride **1g** (14.3 mg, 40%, 93% ee).

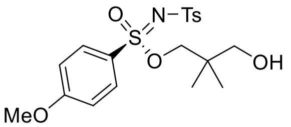

$[\alpha]_D$ = –12.6 (c 0.27, $CHCl_3$). – [Daicel Chiralpak IBN (0.46 cm × 25 cm); 70 : 30 v/v, *n*-hexane / isopropanol; v = 1.2 mL·min^{-1}, λ = 254 nm, t_R (major) = 5.15 min, t_R (minor) = 6.32 min]. – ^{1}H NMR (500 MHz, $CDCl_3$) δ 7.91–7.85 (m, 4H), 7.28 (d, J = 7.2 Hz, 2H), 7.01 (d, J = 9.1 Hz, 2H), 4.03 (d, J = 9.0 Hz, 1H), 3.94 (d, J = 9.0 Hz, 1H), 3.90 (s, 3H), 3.48–3.38 (m, 2H), 2.42 (s, 3H), 0.90 (s, 6H) ppm. – ^{13}C NMR (126 MHz, $CDCl_3$) δ 164.5, 143.2, 139.9, 130.1, 129.3, 126.8, 126.2, 114.7, 76.3, 67.2, 55.9, 36.7, 21.6, 21.1 ppm. – IR: 3522, 2969, 1738, 1593, 1497, 1318, 1266, 1156, 1112, 1089, 1020, 939, 836, 742 cm^{-1}. – HRMS: calcd for $C_{19}H_{25}NO_6S_2$: 428.1196, found 428.1199 [M+H$^+$].

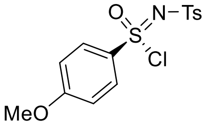

$[\alpha]_D$ = +18.0 (c 0.3, $CHCl_3$). – [Daicel Chiralpak IH (0.46 cm × 25 cm); 50 : 50 v/v, *n*-hexane / isopropanol; v = 1.2 mL·min^{-1}, λ = 254 nm, t_R (major) = 14.41 min, t_R (minor) = 11.0 min]. – m.p.: 60–63 °C. – ^{1}H NMR (500 MHz, $CDCl_3$) δ 8.05–7.95 (m, 4H), 7.36 (d, J = 8.1 Hz, 2H), 7.05 (d, J = 9.2 Hz, 2H), 3.93 (s, 3H), 2.46 (s, 3H) ppm. – ^{13}C NMR (126 MHz, $CDCl_3$) δ 165.3, 144.4, 143.7, 139.1, 138.5, 129.7, 129.6, 127.5, 126.5, 114.9, 56.1, 21.6 ppm. – IR: 2944, 1738, 1588, 1493, 1339, 1270, 1205, 1163, 1106, 1085, 1019, 834, 815, 733 cm^{-1}. – HRMS: calcd for $C_{14}H_{14}ClNO_4S_2$: 360.0126, found 360.0129 [M+H$^+$].

3-Hydroxy-2,2-dimethylpropyl (*R*)-4-(difluoromethoxy)-*N*-tosylbenzenesulfonimidate (3h)

(*S*)-4-(Difluoromethoxy)-*N*-tosylbenzenesulfonimidoyl chloride (1h)

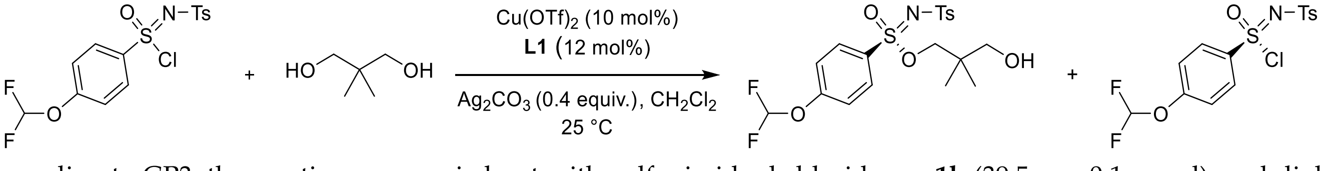

According to GP3, the reaction was carried out with sulfonimidoyl chloride ***rac*-1h** (39.5 mg, 0.1 mmol), and diol **2** (17.7 mg, 0.17 mmol) in anhydrous dichloromethane (1 mL). After stirring for 13 h, for work-up, a saturated solution of EDTA (1.5 mL) was added, followed by dichloromethane (5 mL). The organic phases were combined, dried over

anhydrous Na_2SO_4, concentrated under reduced pressure, and the remaining material was purified by preparative TLC (hexane/ethyl acetate, 80: 20 – 60: 40) to give enantioenriched sulfonimidate product **3h** (18.9 mg, 41%, 99% ee) and sulfonimidoyl chloride **1h** (15.8 mg, 40%, 97% ee).

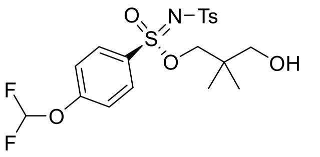

$[\alpha]_D$ = –60.0 (c 0.32, $CHCl_3$). – [Daicel Chiralpak IBN (0.46 cm × 25 cm); 90 : 10 v/v, *n*-hexane / isopropanol; v = 1.2 mL·min^{-1}, λ = 254 nm, t_R (major) = 31.04 min, t_R (minor) = 27.86 min]. – ^{1}H NMR (500 MHz, $CDCl_3$) δ 7.97 (d, *J* = 8.5 Hz, 2H), 7.86 (d, *J* = 8.0 Hz, 2H), 7.32–7.24 (m, 4H), 6.65 (t, *J* = 72.1 Hz, 1H), 4.12–4.01 (m, 2H), 3.44 (s, 2H), 2.42 (s, 3H), 0.92 (s, 6H) ppm. – ^{13}C NMR (126 MHz, $CDCl_3$) δ 155.5, 143.4, 139.6, 131.7, 130.1, 129.4, 126.8, 119.5, 114.9 (t, *J* = 264.1 Hz), 76.7, 67.1, 36.7, 21.5, 21.1 ppm. – ^{19}F NMR (471 MHz, $CDCl_3$) δ –82.6, –82.7 ppm. – IR: 3480, 2923, 1738, 1596,1494, 1370, 1319, 1277, 1156, 1112, 1086, 1013, 920, 837, 814 cm^{-1}. – HRMS: calcd for $C_{19}H_{23}F_2NO_6S_2$: 486.0827, found 486.0825 [M+H$^+$].

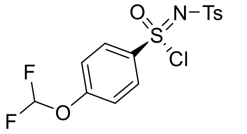

$[\alpha]_D$ = +13.3 (c 0.2, $CHCl_3$). – [Daicel Chiralpak IC (0.46 cm × 25 cm); 70 : 30 v/v, *n*-hexane / isopropanol; v = 1.2 mL·min^{-1}, λ = 254 nm, t_R (major) = 12.67 min, t_R (minor) = 11.32 min]. – m.p.: 50–53 °C. – ^{1}H NMR (500 MHz, $CDCl_3$) δ 8.10 (d, *J* = 8.7 Hz, 2H), 7.98 (d, *J* = 7.8 Hz, 2H), 7.36 (dd, *J* = 20.5, 8.4 Hz, 4H), 6.67 (t, *J* = 71.8 Hz, 1H), 2.47 (s, 3H) ppm. – ^{13}C NMR (126 MHz, $CDCl_3$) δ 156.2, 144.6, 138.4 (d, *J* = 47.1 Hz), 129.6 (d, *J* = 36.7 Hz), 127.5, 119.8, 114.8 (t, *J* = 265.1 Hz), 21.7 ppm. – ^{19}F NMR (471 MHz, $CDCl_3$) δ –81.80, –81.95 ppm. – IR: 2940, 1738, 1590, 1456, 1416, 1347, 1231, 1206, 1162, 1126, 1043, 949, 814 cm^{-1}. – HRMS: calcd for $C_{14}H_{12}ClF_2NO_4S_2$: 417.9795, found 417.9751 [M+Na$^+$].

3-Hydroxy-2,2-dimethylpropyl *(R)-N*-tosyl-3-(trifluoromethoxy)benzenesulfonimidate (3i)

***(S)-N*-Tosyl-3-(trifluoromethoxy)benzenesulfonimidoyl chloride (1i)**

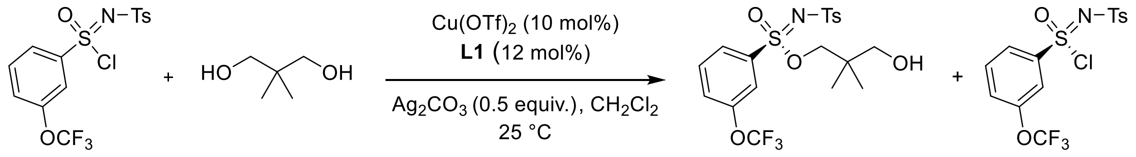

According to GP3, the reaction was carried out with sulfonimidoyl chloride ***rac*-1i** (41.3 mg, 0.1 mmol), and diol **2** (17.7 mg, 0.17 mmol) in anhydrous dichloromethane (1 mL). After stirring for 13 h, for work-up, a saturated solution of EDTA (1.5 mL) was added, followed by dichloromethane (5 mL). The organic phases were combined, dried over anhydrous Na_2SO_4, concentrated under reduced pressure, and the remaining material was purified by preparative TLC (hexane/ethyl acetate, 80: 20 – 60: 40) to give enantioenriched sulfonimidate product **3i** (20.2 mg, 42%, 93% ee) and sulfonimidoyl chloride **1i** (16.5 mg, 40%, 97% ee).

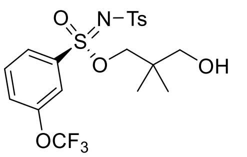

$[\alpha]_D$ = –15.5 (c 0.11, $CHCl_3$). – [Daicel Chiralpak IC (0.46 cm × 25 cm); 50 : 50 v/v, *n*-hexane / isopropanol; v = 1.2 mL·min^{-1}, λ = 254 nm, t_R (major) = 4.46 min, t_R (minor) = 6.18 min]. – ^{1}H NMR (500 MHz, $CDCl_3$) δ 7.90 (d, *J* = 7.9 Hz, 1H), 7.85 (d, *J* = 8.2 Hz, 2H), 7.76 (s, 1H), 7.63 (t, *J* = 8.1 Hz, 1H), 7.54 (d, *J* = 8.2 Hz, 1H), 7.28 (d, *J* = 8.1 Hz, 2H), 4.10 (s, 2H), 3.43 (d, *J* = 2.6 Hz, 2H), 2.42 (s, 3H), 0.92 (d, *J* = 4.1 Hz, 6H) ppm. – ^{13}C NMR (126 MHz, $CDCl_3$) δ 149.3, 143.6, 139.4, 137.5, 131.2, 129.4, 126.8, 126.0, 120.2, 77.2, 67.1, 36.7, 21.5, 21.0 ppm. – ^{19}F NMR (471 MHz, $CDCl_3$) δ –58.0 ppm. – IR: 3527, 2968, 1738, 1597, 1475, 1322, 1256, 1208, 1156, 1113, 1088, 999, 911 cm^{-1}. – HRMS: calcd for $C_{19}H_{22}F_3NO_6S_2$: 482.0913, found 482.0913 [M+H$^+$].

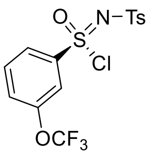

$[\alpha]_D$ = +29.2 (c 0.12, $CHCl_3$). – [Daicel Chiralpak IC (0.46 cm × 25 cm); 50 : 50 v/v, *n*-hexane / isopropanol; v = 1.2 mL·min^{-1}, λ = 254 nm, t_R (major) = 5.24 min, t_R (minor) = 4.83 min]. – ^{1}H NMR (500 MHz, $CDCl_3$) δ 8.04 (d, *J* = 8.1 Hz, 1H), 7.99 (d, *J* = 7.8 Hz, 2H), 7.91 (s, 1H), 7.71 (t, *J* = 8.2 Hz, 1H), 7.63 (d, *J* = 8.2 Hz, 1H), 7.39 (d, *J* = 7.9 Hz, 2H), 2.48 (s, 3H) ppm. – ^{13}C NMR (126 MHz, $CDCl_3$) δ 149.4, 144.8, 144.1, 138.1, 131.6, 129.8, 127.8, 127.5, 125.1, 121.2, 119.5, 119.1, 21.7 ppm. – ^{19}F NMR (471 MHz, $CDCl_3$) δ –58.0 ppm. – IR: 3086, 1596, 1476, 1344, 1288, 1255, 1207, 1161, 1108, 1084, 997, 946, 888 cm^{-1}. – HRMS: calcd for $C_{14}H_{11}ClF_3NO_4S_2$: 413.9843, found 413.9833 [M+H$^+$].

3-Hydroxy-2,2-dimethylpropyl *(R)-3-*(methylsulfonyl)-*N*-tosylbenzenesulfonimidate (3j)

(S)-3-(Methylsulfonyl)-*N*-tosylbenzenesulfonimidoyl chloride (1j)

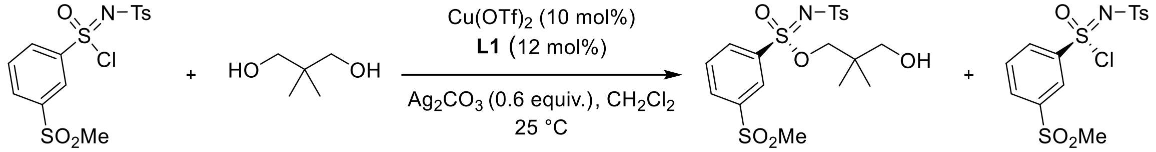

According to GP3, the reaction was carried out with sulfonimidoyl chloride ***rac*-1j** (40.7 mg, 0.1 mmol), and diol **2** (17.7 mg, 0.17 mmol) in anhydrous dichloromethane (1 mL). After stirring for 13 h, for work-up, a saturated solution of EDTA (1.5 mL) was added, followed by dichloromethane (5 mL). The organic phases were combined, dried over anhydrous Na_2SO_4, concentrated under reduced pressure, and the remaining material was purified by preparative TLC (hexane/ethyl acetate, 80 : 20 – 60 : 40) to give enantioenriched sulfonimidate product **3j** (20.0 mg, 42%, 94% ee) and sulfonimidoyl chloride **1j** (16.3 mg, 40%, 96% ee).

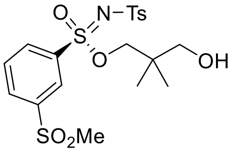

$[\alpha]_D$ = –5.1 (c 0.5, $CHCl_3$). – [Daicel Chiralpak IH (0.46 cm × 25 cm); 50 : 50 v/v, *n*-hexane / isopropanol; v = 1.2 mL·min^{-1}, λ = 254 nm, t_R (major) = 21.09 min, t_R (minor) = 14.87 min]. – ^{1}H NMR (500 MHz, $CDCl_3$) δ 8.50 (s, 1H), 8.26 (d, *J* = 7.9 Hz, 2H), 7.92–7.79 (m, 3H), 7.31 (d, *J* = 8.1 Hz, 3H), 4.19–4.07 (m, 2H), 3.42 (d, *J* = 5.7 Hz, 2H), 3.13 (s, 3H), 2.44 (s, 3H), 0.93 (d, *J* = 4.9 Hz, 6H) ppm. – ^{13}C NMR (126 MHz, $CDCl_3$) δ 143.7, 142.3, 139.4, 137.9, 133.0, 132.6, 130.8, 129.5, 126.9, 126.8, 77.4, 66.9, 44.3, 36.7, 21.6, 21.1 ppm. – IR: 3539, 3071, 2925, 1738, 1491, 1320, 1303, 1158, 1122, 1088, 995, 917 cm^{-1}. – HRMS: calcd for $C_{19}H_{25}NO_7S_3$: 493.1131, found 493.1133 [$M+NH_4^+$].

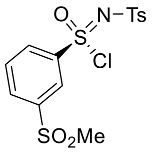

$[\alpha]_D$ = +71.6 (c 0.3, $CHCl_3$). – [Daicel Chiralpak IC (0.46 cm × 25 cm); 50 : 50 v/v, *n*-hexane / isopropanol; v = 1.2 mL·min^{-1}, λ = 254 nm, t_R (major) = 24.3 min, t_R (minor) = 20.01 min]. – m.p.: 120–122 °C. – ^{1}H NMR (500 MHz, $CDCl_3$) δ 8.61 (s, 1H), 8.36 (dd, *J* = 18.1, 7.9 Hz, 2H), 7.99 (d, *J* = 8.0 Hz, 2H), 7.90 (t, *J* = 8.0 Hz, 1H), 7.40 (d, *J* = 7.9 Hz, 2H), 3.16 (s, 3H), 2.48 (s, 3H) ppm. – ^{13}C NMR (126 MHz, $CDCl_3$) δ 145.0, 144.1, 142.9, 137.9, 134.0, 131.6, 131.4, 129.9, 127.6, 126.0, 44.4, 21.7 ppm. – IR: 3071, 2926, 1596, 1411, 1321, 1303, 1288, 1187, 1157, 1118, 1084, 993, 962 cm^{-1}. – HRMS: calcd for $C_{14}H_{14}ClNO_3S_3$: 445.9354, found 445.9342 [$M+K^+$].

3-Hydroxy-2,2-dimethylpropyl (*R*)-4-cyano-*N*-tosylbenzenesulfonimidate (3k)

(*S*)-4-Cyano-*N*-tosylbenzenesulfonimidoyl chloride (1k)

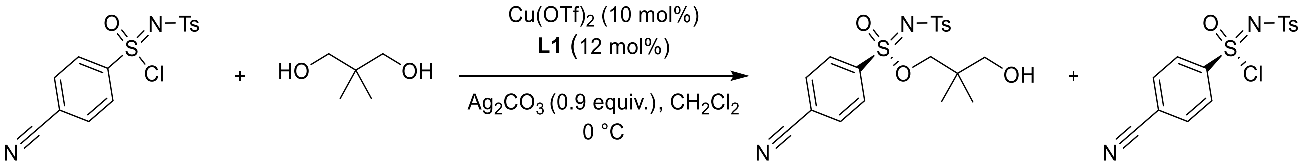

According to GP3, the reaction was carried out with sulfonimidoyl chloride ***rac*-1k** (35.4 mg, 0.1 mmol), and diol **2** (17.7 mg, 0.17 mmol) in anhydrous dichloromethane (1 mL). After stirring for 9 h, for work-up, a saturated solution of EDTA (1.5 mL) was added, followed by dichloromethane (5 mL). The organic phases were combined, dried over anhydrous Na_2SO_4, concentrated under reduced pressure, and the remaining material was purified by preparative TLC (hexane/ethyl acetate, 80: 20 – 60: 40) to give enantioenriched sulfonimidate product **3k** (17.9 mg, 42 %, 92% ee) and sulfonimidoyl chloride **1k** (14.2 mg, 40 %, 99% ee).

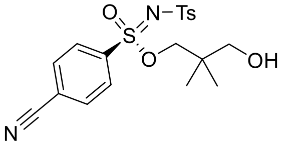

$[\alpha]_D$ = –30.0 (c 0.1, $CHCl_3$). – [Daicel Chiralpak IF (0.46 cm × 25 cm); 70 : 30 v/v, *n*-hexane / isopropanol; v = 1.2 mL·min^{-1}, λ = 254 nm, t_R (major) = 5.29 min, t_R (minor) = 4.16 min]. – ^{1}H NMR (500 MHz, $CDCl_3$) δ 8.09 (d, *J* = 8.6 Hz, 2H), 7.86 (dd, *J* = 8.4, 4.6 Hz, 4H), 7.30 (d, *J* = 8.1 Hz, 2H), 4.12 (d, *J* = 1.7 Hz, 2H), 3.44 (d, *J* = 4.9 Hz, 2H), 2.44 (s, 3H), 0.93 (d, *J* = 4.7 Hz, 6H) ppm. – ^{13}C NMR (126 MHz, $CDCl_3$) δ 143.8, 140.0, 139.3, 133.1, 132.3, 129.5, 128.4, 126.8, 118.3, 116.7, 77.4, 67.0, 36.8, 21.6, 21.0 ppm. – IR: 3454, 3094, 2923, 2234, 1737, 1597, 1456, 1322, 1279, 1157, 1117, 1088, 1016, 919 cm^{-1}. – HRMS: calcd for $C_{19}H_{22}N_2O_5S_2$: 423.1043, found 423.1041 [$M+H^+$].

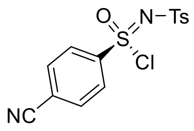

$[\alpha]_D$ = +15.6 (c 0.16, $CHCl_3$). – [Daicel Chiralpak IF (0.46 cm × 25 cm); 70 : 30 v/v, *n*-hexane / isopropanol; v = 1.2 mL·min^{-1}, λ = 254 nm, t_R (major) = 24.81 min, t_R (minor) = 14.06 min]. m.p.: 95–97 °C. – ^{1}H NMR (500 MHz, $CDCl_3$) δ 8.21 (d, *J* = 8.4 Hz, 2H), 7.96 (dd, *J* = 21.4, 8.3 Hz, 4H), 7.39 (d, *J* = 8.1 Hz, 2H), 2.48 (s, 3H) ppm. – ^{13}C NMR (126 MHz, $CDCl_3$) δ 146.0, 145.0, 137.9, 133.5, 129.9, 127.6, 127.5, 119.2, 116.3, 21.7 ppm. – IR: 3095, 2923, 2236, 1596, 1491, 1399, 1341, 1286, 1163, 1113, 1086, 1013 cm^{-1}. – HRMS: calcd for $C_{14}H_{11}ClN_2O_3S_2$: 372.0238, found 372.0235 [M+NH_4^+].

3-Hydroxy-2,2-dimethylpropyl *(R)*-4-methyl-*N*-tosylbenzenesulfonimidate (3l)

***(S)*-4-Methyl-*N*-tosylbenzenesulfonimidoyl chloride (1l)**

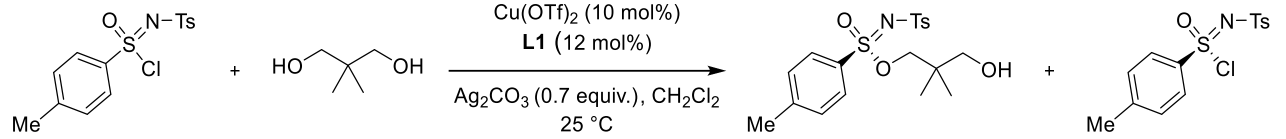

According to GP3, the reaction was carried out with sulfonimidoyl chloride ***rac*-1l** (34.4 mg, 0.1 mmol), and diol **2** (17.7 mg, 0.17 mmol) in anhydrous dichloromethane (1 mL). After stirring for 13.5 h, for work-up, a saturated solution of EDTA (1.5 mL) was added, followed by dichloromethane (5 mL). The organic phases were combined, dried over anhydrous Na_2SO_4, concentrated under reduced pressure, and the remaining material was purified by preparative TLC (hexane/ethyl acetate, 80: 20 – 60: 40) to give enantioenriched sulfonimidate product **3l** (17.8 mg, 43%, 92% ee) and sulfonimidoyl chloride **1l** (13.9 mg, 40 %, 90% ee).

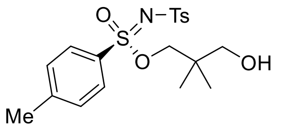

$[\alpha]_D$ = –7.9 (c 0.4, $CHCl_3$). – [Daicel Chiralpak IBN (0.46 cm × 25 cm); 90 : 10 v/v, *n*-hexane / isopropanol; v = 1.2 mL·min^{-1}, λ = 254 nm, t_R (major) = 14.56 min, t_R (minor) = 19.68 min]. – ^{1}H NMR (500 MHz, $CDCl_3$) δ 7.88 (d, *J* = 8.1 Hz, 2H), 7.84 (d, *J* = 8.2 Hz, 2H), 7.36 (d, *J* = 8.1 Hz, 2H), 7.30–7.26 (m, 2H), 4.05 (d, *J* = 9.0 Hz, 1H), 3.96 (d, *J* = 9.0 Hz, 1H), 3.49–3.40 (m, 2H), 2.47 (s, 3H), 2.42 (s, 3H), 0.90 (s, 6H) ppm. – ^{13}C NMR (126 MHz, $CDCl_3$) δ 146.0, 143.2, 139.9, 132.2, 130.0, 129.3, 127.7, 126.8, 76.4, 67.1, 36.7, 21.7, 21.6, 21.1 ppm. – IR: 3523, 2963, 1746, 1596, 1453, 1320, 1275, 1156, 1115, 1089, 938, 813, 738 cm^{-1}. – HRMS: calcd for $C_{19}H_{25}NO_5S_2$: 412.1247, found 412.1238 [M+H$^+$].

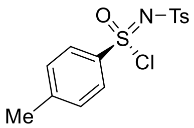

$[\alpha]_D$ = +52.9 (c 0.57, $CHCl_3$). – [Daicel Chiralpak IG (0.46 cm × 25 cm); 70 : 30 v/v, *n*-hexane / isopropanol; v = 1.2 mL·min^{-1}, λ = 254 nm, t_R (major) = 22.23 min, t_R (minor) = 18.73 min]. – m.p.: 48–50 °C. – ^{1}H NMR (500 MHz, $CDCl_3$) δ 7.96 (dd, *J* = 18.4, 8.2 Hz, 4H), 7.38 (dd, *J* = 24.6, 8.2 Hz, 4H), 2.49 (s, 3H), 2.46 (s, 3H) ppm. – ^{13}C NMR (126 MHz, $CDCl_3$) δ 147.4, 144.5, 139.7, 138.4, 130.4, 129.7, 127.5, 126.9, 21.8, 21.7 ppm. – IR: 2924, 1743, 1596, 1478, 1456, 1342, 1285, 1205, 1151, 1111, 1087, 1013, 814, 731 cm^{-1}. – HRMS: calcd for $C_{14}H_{14}ClNO_3S_2$: 365.9996, found 365.9983 [M+Na$^+$].

3-Hydroxy-2,2-dimethylpropyl (*R*)-4-(*tert*-butyl)-*N*-tosylbenzenesulfonimidate (3m)

(*S*)-4-(*tert*-butyl)-*N*-tosylbenzenesulfonimidoyl chloride (1m)

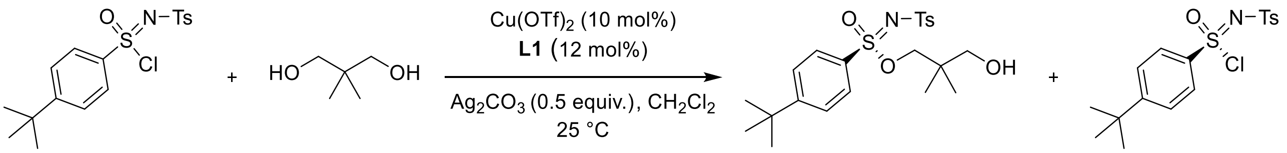

According to GP3, the reaction was carried out with sulfonimidoyl chloride ***rac*-1m** (38.6 mg, 0.1 mmol), and diol **2** (17.7 mg, 0.17 mmol) in anhydrous dichloromethane (1 mL). After stirring for 17 h, for work-up, a saturated solution of EDTA (1.5 mL) was added, followed by dichloromethane (5 mL). The organic phases were combined, dried over anhydrous Na_2SO_4, concentrated under reduced pressure, and the remaining material was purified by preparative TLC (hexane/ethyl acetate, 80: 20 – 60: 40) to give enantioenriched sulfonimidate product **3m** (20.3 mg, 45%, 92% ee) and sulfonimidoyl chloride **1m** (15.8 mg, 41%, 96% ee).

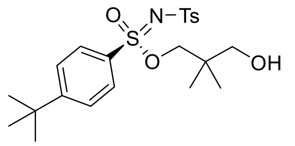

[α]D = –23.8 (c 0.37, $CHCl_3$). – [Daicel Chiralpak IBN (0.46 cm × 25 cm); 70 : 30 v/v, *n*-hexane / isopropanol; v = 1.2 mL·min^{-1}, λ = 254 nm, t_R (major) = 3.09 min, t_R (minor) = 3.87 min]. – ^{1}H NMR (500 MHz, $CDCl_3$) δ 7.93–7.78 (m, 4H), 7.55 (d, *J* = 8.3 Hz, 2H), 7.26 (d, *J* = 7.9 Hz, 2H), 4.12–3.95 (m, 2H), 3.45 (s, 2H), 2.41 (s, 3H), 1.35 (s, 9H), 0.92 (s, 6H) ppm. – ^{13}C NMR (126 MHz, $CDCl_3$) δ 158.8, 143.1, 139.9, 132.2, 129.3, 127.5, 126.8, 126.4, 76.4, 67.2, 36.8, 35.4, 31.0, 21.5, 21.1 ppm. – IR: 3416, 2962, 1738, 1593, 1474, 1400, 1320, 1271, 1156, 1128, 1088, 1012, 938, 835, 814, 735 cm^{-1}. – HRMS: calcd for $C_{22}H_{31}NO_5S_2$: 454.1716, found 454.1714 [M+H$^+$].

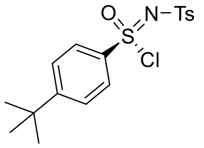

[α]D = +47.5 (c 0.4, $CHCl_3$). – [Daicel Chiralpak IG (0.46 cm × 25 cm); 70 : 30 v/v, *n*-hexane / isopropanol; v = 1.2 mL·min^{-1}, λ = 254 nm, t_R (major) = 13.46 min, t_R (minor) = 11.69 min]. – ^{1}H NMR (500 MHz, $CDCl_3$) δ 7.99 (d, *J* = 8.2 Hz, 4H), 7.62 (d, *J* = 8.6 Hz, 2H), 7.36 (d, *J* = 8.1 Hz, 2H), 2.46 (s, 3H), 1.37 (s, 10H) ppm. – ^{13}C NMR (126 MHz, $CDCl_3$) δ 160.2, 144.4, 139.6, 138.5, 129.6, 127.5, 126.8, 126.8, 35.6, 30.9, 21.7 ppm. – IR: 2968, 1738, 1595, 148, 1265, 1230, 1205, 1152, 1124, 1100, 1087, 817, 735 cm^{-1}. – HRMS: calcd for $C_{17}H_{20}ClNO_3S_2$: 408.0465, found 408.0461 [M+Na$^+$].

3-Hydroxy-2,2-dimethylpropyl (*R*)-*N*-tosyl-[1,1'-biphenyl]-4-sulfonimidate (3n)

(*S*)-*N*-Tosyl-[1,1'-biphenyl]-4-sulfonimidoyl chloride (1n)

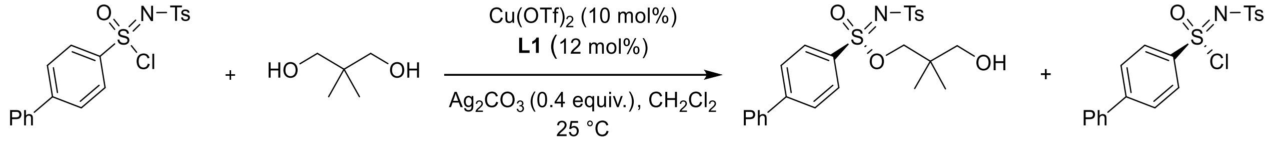

According to GP3, the reaction was carried out with sulfonimidoyl chloride ***rac*-1n** (40.5 mg, 0.1 mmol), and diol **2** (17.7 mg, 0.17 mmol) in anhydrous dichloromethane (1 mL). After stirring for 13 h, for work-up, a saturated solution of EDTA (1.5 mL) was added, followed by dichloromethane (5 mL). The organic phases were combined, dried over anhydrous Na_2SO_4, concentrated under reduced pressure, and the remaining material was purified by preparative TLC (hexane/ethyl acetate, 80 : 20 – 60 : 40) to give enantioenriched sulfonimidate product **3n** (20.3 mg, 41%, 95% ee) and sulfonimidoyl chloride **1n** (16.6 mg, 43%, 98% ee).

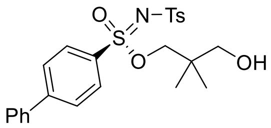

[α]D = –15.2 (c 0.33, $CHCl_3$). – [Daicel Chiralpak IF (0.46 cm × 25 cm); 70 : 30 v/v, *n*-hexane / isopropanol; v = 1.2 mL·min^{-1}, λ = 254 nm, t_R (major) = 12.99 min, t_R (minor) = 12.0 min]. – ^{1}H NMR (500 MHz, $CDCl_3$) δ 8.01 (d, *J* = 8.4 Hz, 2H), 7.89 (d, *J* = 8.2 Hz, 2H), 7.76 (d, *J* = 8.4 Hz, 2H), 7.61 (d, *J* = 7.5 Hz, 2H), 7.55–7.43 (m, 3H), 7.31–7.23 (m, 2H), 4.16–4.01 (m, 2H), 3.47 (s, 2H), 2.41 (s, 3H), 0.94 (d, *J* = 2.0 Hz, 6H) ppm. – ^{13}C NMR (126 MHz, $CDCl_3$) δ 147.6, 143.3, 139.8, 138.6, 133.7, 129.3, 129.2, 129.0, 128.2, 127.9, 127.4, 126.8, 116.4, 76.7, 67.2, 36.8, 21.5, 21.1 ppm. – IR: 3501, 2961, 1738, 1593, 1478, 1319, 1279, 1155, 1116, 1088, 1017, 937 cm^{-1}. – HRMS: calcd for $C_{24}H_{27}NO_5S_2$: 474.1403, found 474.1393 [M+H$^+$].

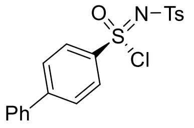

[α]D = +11.2 (c 0.13, $CHCl_3$). – [Daicel Chiralpak IH (0.46 cm × 25 cm); 50 : 50 v/v, *n*-hexane / isopropanol; v = 1.2 mL·min^{-1}, λ = 254 nm, t_R (major) = 11.25 min, t_R (minor) = 8.16 min]. – m.p.: 70–73 °C. – ^{1}H NMR (500 MHz, $CDCl_3$) δ 8.14 (d, *J* = 8.6 Hz, 2H), 8.01 (d, *J* = 8.3 Hz, 2H), 7.81 (d, *J* = 8.7 Hz, 2H), 7.62 (d, *J* = 6.8 Hz, 2H), 7.57–7.45 (m, 3H), 7.38 (d, *J* = 8.2 Hz, 2H), 2.48 (s, 3H) ppm. – ^{13}C NMR (126 MHz, $CDCl_3$) δ 148.8, 144.5, 140.9, 138.4, 138.2, 129.7, 129.4, 129.3, 128.3, 127.5, 127.5, 127.5, 21.7 ppm. – IR: 3063, 1587, 1477, 1399, 1338, 1284, 1185, 1163, 1109, 1085, 1004, 916 cm^{-1}. – HRMS: calcd for $C_{19}H_{16}ClNO_3S_2$: 423.0598, found 423.0595 [M+NH$_4^+$].

3-Hydroxy-2,2-dimethylpropyl *(R)*-4'-fluoro-*N*-tosyl-[1,1'-biphenyl]-4-sulfonimidate (3o)

_(S)_-4'-Fluoro-*N*-tosyl-[1,1'-biphenyl]-4-sulfonimidoyl chloride (1o)

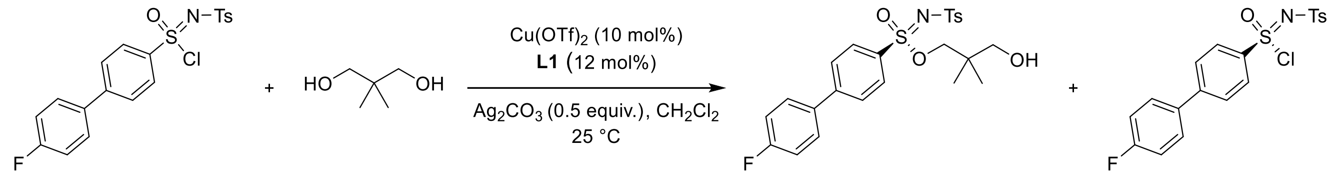

According to GP3, the reaction was carried out with sulfonimidoyl chloride ***rac*-1o** (42.3 mg, 0.1 mmol), and diol **2** (17.7 mg, 0.17 mmol) in anhydrous dichloromethane (1 mL). After stirring for 13 h, for work-up, a saturated solution of EDTA (1.5 mL) was added, followed by dichloromethane (5 mL). The organic phases were combined, dried over anhydrous Na_2SO_4, concentrated under reduced pressure, and the remaining material was purified by preparative TLC (hexane/ethyl acetate, 80: 20 – 60: 40) to give enantioenriched sulfonimidate product **3o** (19.6 mg, 40%, 92% ee) and sulfonimidoyl chloride **1o** (16.9 mg, 40%, 96% ee).

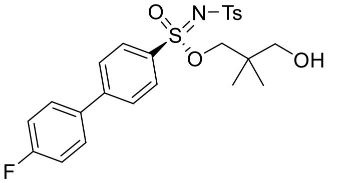

$[\alpha]_D$ = –69.2 (c 0.25, $CHCl_3$). – [Daicel Chiralpak IBN (0.46 cm × 25 cm); 70 : 30 v/v, *n*-hexane / isopropanol; v = 1.2 mL·min^{-1}, λ = 254 nm, t_R (major) = 6.05 min, t_R (minor) = 7.75 min]. – ^{1}H NMR (500 MHz, $CDCl_3$) δ 8.01 (d, *J* = 7.9 Hz, 2H), 7.90 (d, *J* = 7.7 Hz, 2H), 7.72 (d, *J* = 8.0 Hz, 2H), 7.64–7.54 (m, 2H), 7.29 (d, *J* = 8.0 Hz, 3H), 7.21 (t, *J* = 8.2 Hz, 2H), 4.16–4.01 (m, 2H), 3.47 (s, 2H), 2.42 (s, 3H), 0.94 (s, 6H) ppm. – ^{13}C NMR (126 MHz, $CDCl_3$) δ 163.4 (d, *J* = 249.9 Hz), 146.6, 143.3, 139.8, 134.7 (d, *J* = 3.0 Hz), 133.8, 129.4, 129.2 (d, *J* = 8.3 Hz), 128.3, 127.8, 126.8, 116.3 (d, *J* = 21.8 Hz), 76.6, 67.2, 36.8, 21.6, 21.1 ppm. – ^{19}F NMR (471 MHz, $CDCl_3$) δ –112.48 ppm. – IR: 3501, 2924, 1738, 1596, 1518, 1486, 1320, 1278, 1156, 1117, 1089, 938 cm^{-1}. – HRMS: calcd for $C_{24}H_{26}FNO_5S_2$: 492.1309, found 492.1312 [M+H$^+$].

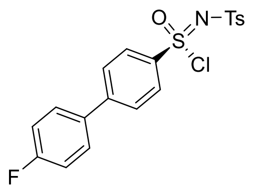

$[\alpha]_D$ = +39.0 (c 0.3, $CHCl_3$). – [Daicel Chiralpak IH (0.46 cm × 25 cm); 50 : 50 v/v, *n*-hexane / isopropanol; v = 1.2 mL·min^{-1}, λ = 254 nm, t_R (major) = 14.97 min, t_R (minor) = 9.59 min]. – m.p.: 83–85 °C. – ^{1}H NMR (500 MHz, $CDCl_3$) δ 8.13 (d, *J* = 8.2 Hz, 2H), 8.01 (d, *J* = 7.9 Hz, 2H), 7.77 (d, *J* = 8.3 Hz, 2H), 7.68–7.55 (m, 2H), 7.38 (d, *J* = 8.0 Hz, 2H), 7.22 (t, *J* = 8.3 Hz, 2H), 2.47 (s, 3H) ppm. – ^{13}C NMR (126 MHz, $CDCl_3$) δ 163.6 (d, *J* = 250.1 Hz), 147.6, 144.5, 141.0, 138.4, 134.4 (d, *J* = 3.5 Hz), 129.7, 129.3 (d, *J* = 8.3 Hz), 128.1, 127.6 (d, *J* = 5.3 Hz), 116.4 (d, *J* = 21.8 Hz), 21.7 ppm. – ^{19}F NMR (471 MHz, $CDCl_3$) δ –111.83, –113.64 ppm. – IR: 2969, 1738, 1595, 1365, 1216, 1205, 1161, 1127, 895, 821 cm^{-1}. – HRMS: calcd for $C_{19}H_{15}ClFNO_3S_2$: 446.0058, found 446.0054 [M+Na$^+$].

3-Hydroxy-2,2-dimethylpropyl *(R)*-2,5-difluoro-*N*-tosylbenzenesulfonimidate (3p)

_(S)_-2,5-Difluoro-*N*-tosylbenzenesulfonimidoyl chloride (1p)

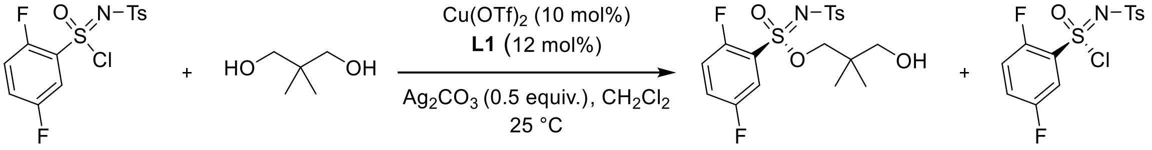

According to GP3, the reaction was carried out with sulfonimidoyl chloride ***rac*-1p** (36.5 mg, 0.1 mmol), and diol **2** (17.7 mg, 0.17 mmol) in anhydrous dichloromethane (1 mL). After stirring for 13 h, for work-up, a saturated solution of EDTA (1.5 mL) was added, followed by dichloromethane (5 mL). The organic phases were combined, dried over anhydrous Na_2SO_4, concentrated under reduced pressure, and the remaining material was purified by preparative TLC (hexane/ethyl acetate, 80: 20 – 60: 40) to give enantioenriched sulfonimidate product **3p** (17.7 mg, 41%, 97% ee) and sulfonimidoyl chloride **1p** (14.5 mg, 40%, 96% ee).

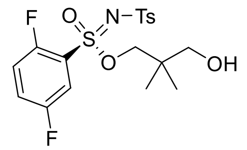

[α]D = –13.6 (c 0.11, $CHCl_3$). – [Daicel Chiralpak IG (0.46 cm × 25 cm); 70 : 30 v/v, *n*-hexane / isopropanol; v = 1.2 mL·min^{-1}, λ = 254 nm, t_R (major) = 13.02 min, t_R (minor) = 14.27 min]. – ^{1}H NMR (500 MHz, $CDCl_3$) δ 7.85 (d, *J* = 8.1 Hz, 2H), 7.74–7.61 (m, 1H), 7.43–7.33 (m, 1H), 7.26 (dd, *J* = 29.0, 5.6 Hz, 3H), 4.31–4.10 (m, 2H), 3.55–3.35 (m, 2H), 2.43 (s, 3H), 0.95 (d, *J* = 8.2 Hz, 6H) ppm. – ^{13}C NMR (126 MHz, $CDCl_3$) δ 157.6 (d, *J* = 249.2 Hz), 155.1 (d, *J* = 256.2 Hz), 143.6, 139.3, 129.4, 126.8, 123.8 (d, *J* = 8.5 Hz), 123.6 (d, *J* = 8.4 Hz), 119.3 (d, *J* = 8.0 Hz), 119.1 (d, *J* = 7.9 Hz), 117.8 (d, *J* = 28.0 Hz), 77.7, 67.0, 36.7, 21.6, 21.0 ppm. – ^{19}F NMR (471 MHz, $CDCl_3$) δ –112.3 (d, *J* = 17.2 Hz), –114.3 (d, *J* = 18.4 Hz), –114.5 (d, *J* = 54.6 Hz), –117.3 ppm. – IR: 3527, 3071, 2924, 1738, 1597, 1489, 1323, 1287, 1158, 1119, 1088, 1055, 936 cm^{-1}.– HRMS: calcd for $C_{18}H_{21}F_2NO_5S_2$: 434.0902, found 434.0902 [M+H$^+$].

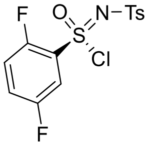

[α]D = +25.0 (c 0.1, $CHCl_3$). – [Daicel Chiralpak IG (0.46 cm × 25 cm); 70 : 30 v/v, *n*-hexane / isopropanol; v = 1.2 mL·min^{-1}, λ = 254 nm, t_R (major) = 12.28 min, t_R (minor) = 14.15 min]. – m.p.: 78–80 °C. – ^{1}H NMR (500 MHz, $CDCl_3$) δ 7.97 (d, *J* = 8.2 Hz, 2H), 7.80–7.71 (m, 1H), 7.53–7.44 (m, 1H), 7.36 (dd, *J* = 19.5, 6.0 Hz, 3H), 2.48 (s, 3H) ppm. – ^{13}C NMR (126 MHz, $CDCl_3$) δ 157.3 (d, *J* = 251.0 Hz), 154.8 (d, *J* = 261.5 Hz), 144.8, 138.1, 129.8, 127.5, 125.0 (d, *J* = 8.9 Hz), 124.8 (d, *J* = 8.8 Hz), 120.1 (d, *J* = 7.8 Hz), 119.9 (d, *J* = 7.9 Hz), 116.3 (d, *J* = 28.7 Hz), 21.7 ppm. – ^{19}F NMR (471 MHz, $CDCl_3$) δ –110.3 (dt, *J* = 13.0, 6.4 Hz), –113.1 (dd, *J* = 15.5, 8.2 Hz) ppm. – IR: 3070, 1596, 1490, 1402, 1341, 1290, 1254, 1199, 1163, 1109, 1084, 1047, 876 cm^{-1}. – HRMS: calcd for $C_{13}H_{10}ClF_2NO_3S_2$: 383.0097, found 383.0091 [M+NH$_4^+$].

3-Hydroxy-2,2-dimethylpropyl *(R)*-3,5-dichloro-*N*-tosylbenzenesulfonimidate (3q)

***(S)*-3,5-Dichloro-*N*-tosylbenzenesulfonimidoyl chloride (1q)**

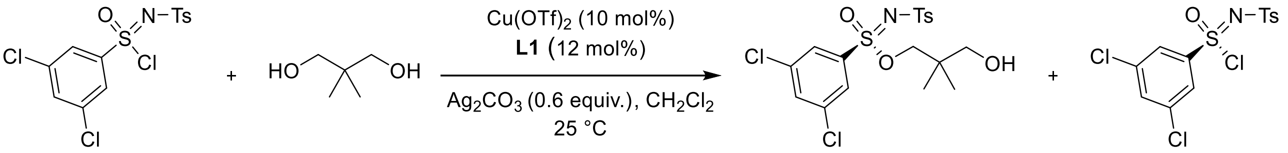

According to GP3, the reaction was carried out with sulfonimidoyl chloride ***rac*-1q** (39.8 mg, 0.1 mmol), and diol **2** (17.7 mg, 0.17 mmol) in anhydrous dichloromethane (1 mL). After stirring for 13 h, for work-up, a saturated solution of EDTA (1.5 mL) was added, followed by dichloromethane (5 mL). The organic phases were combined, dried over anhydrous Na_2SO_4, concentrated under reduced pressure, and the remaining material was purified by preparative TLC (hexane/ethyl acetate, 80 : 20 – 60 : 40) to give enantioenriched sulfonimidate product **3q** (19.1 mg, 41 %, 92% ee) and sulfonimidoyl chloride **1q** (15.9 mg, 40%, 99% ee).

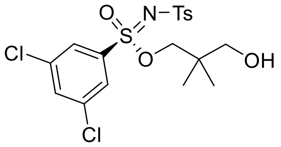

[α]D = –25.0 (c 0.2, $CHCl_3$). – [Daicel Chiralpak IG (0.46 cm × 25 cm); 70 : 30 v/v, *n*-hexane / isopropanol; v = 1.2 mL·min^{-1}, λ = 254 nm, t_R (major) = 11.82 min, t_R (minor) = 10.03 min]. – ^{1}H NMR (500 MHz, $CDCl_3$) δ 7.85 (d, *J* = 7.8 Hz, 2H), 7.79 (s, 2H), 7.65 (s, 1H), 7.30 (d, *J* = 10.7 Hz, 3H), 4.19–4.08 (m, 2H), 3.52–3.39 (m, 2H), 2.44 (s, 3H), 0.95 (d, *J* = 3.1 Hz, 6H) ppm. – ^{13}C NMR (126 MHz, $CDCl_3$) δ 143.7, 139.2, 138.3, 136.5, 134.5, 129.4, 126.8, 125.9, 77.5, 67.0, 36.8, 21.6, 21.1 ppm. – IR: 3521, 3075, 2923, 1738, 1570, 1417, 1324, 1282, 1150, 1112, 1087, 993, 918 cm^{-1}. – HRMS: calcd for $C_{18}H_{21}Cl_2NO_5S_2$: 466.0311, found 466.0301 [M+H$^+$].

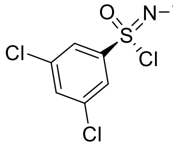

[α]D = +8.9 (c 0.8, $CHCl_3$). – [Daicel Chiralpak IG (0.46 cm × 25 cm); 70 : 30 v/v, *n*-hexane / isopropanol; v = 1.2 mL·min^{-1}, λ = 254 nm, t_R (major) = 11.18 min, t_R (minor) = 12.59 min]. – m.p.: 70–72 °C. – ^{1}H NMR (500 MHz, $CDCl_3$) δ 8.06–7.91 (m, 4H), 7.73 (s, 1H), 7.39 (d, *J* = 8.0 Hz, 2H), 2.48 (s, 3H) ppm. – ^{13}C NMR (126 MHz, $CDCl_3$) δ 145.0, 144.5, 137.9, 136.8, 135.6, 129.8, 127.5, 125.1, 21.7 ppm. – IR: 3091, 2924, 1586, 1578, 1461, 1396, 1339, 1275, 1161, 1110, 1067, 1006 cm^{-1}. – HRMS: calcd for $C_{13}H_{10}Cl_3NO_3S_2$: 414.9506, found 414.9505 [M+NH$_4^+$].

3-Hydroxy-2,2-dimethylpropyl *(R)*-3-chloro-4-fluoro-*N*-tosylbenzenesulfonimidate (3r)

***(S)*-3-Chloro-4-fluoro-*N*-tosylbenzenesulfonimidoyl chloride (1r)**

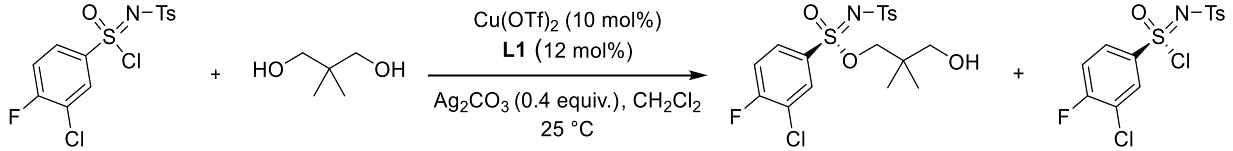

According to GP3, the reaction was carried out with sulfonimidoyl chloride ***rac*-1r** (38.2 mg, 0.1 mmol), and diol **2** (17.7 mg, 0.17 mmol) in anhydrous dichloromethane (1 mL). After stirring for 13 h, for work-up, a saturated solution of EDTA (1.5 mL) was added, followed by dichloromethane (5 mL). The organic phases were combined, dried over anhydrous Na_2SO_4, concentrated under reduced pressure, and the remaining material was purified by preparative TLC (hexane/ethyl acetate, 80: 20 – 60: 40) to give enantioenriched sulfonimidate product **3r** (19.4 mg, 43%, 93% ee) and sulfonimidoyl chloride **1r** (15.7 mg, 41%, 97% ee).

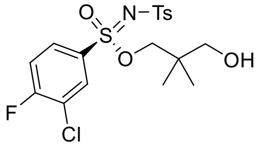

$[\alpha]_D$ = –11.6 (c 0.36, $CHCl_3$). – [Daicel Chiralpak IBN (0.46 cm × 25 cm); 70 : 30 v/v, *n*-hexane / isopropanol; v = 1.2 mL·min^{-1}, λ = 254 nm, t_R (major) = 3.66 min, t_R (minor) = 4.02 min]. – ^{1}H NMR (500 MHz, $CDCl_3$) δ 8.00 (dd, *J* = 6.4, 2.1 Hz, 1H), 7.87 (dd, *J* = 14.3, 6.5 Hz, 3H), 7.41–7.21 (m, 3H), 4.10 (s, 2H), 3.44 (d, *J* = 2.6 Hz, 2H), 2.43 (s, 3H), 0.93 (d, *J* = 3.0 Hz, 6H) ppm. – ^{13}C NMR (126 MHz, $CDCl_3$) δ 161.8 (d, *J* = 260.2 Hz), 143.6, 139.4, 132.4 (d, *J* = 4.3 Hz), 130.7, 129.4, 128.4 (d, *J* = 8.8 Hz), 126.8, 123.1 (d, *J* = 19.0 Hz), 117.8 (d, *J* = 22.7 Hz), 77.1, 67.1, 36.8, 21.6, 21.1 ppm. – ^{19}F NMR (471 MHz, $CDCl_3$) *δ* –103.37 ppm. – IR: 3355, 2922, 2853, 1738, 1484, 1456, 1375, 1265, 1157, 1125, 1086, 1058, 914, 815, 739 cm^{-1}. – HRMS: calcd for $C_{18}H_{21}ClFNO_5S_2$: 472.0426, found 472.0433 [M+Na$^+$].

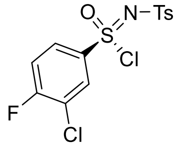

$[\alpha]_D$ = +14.1 (c 0.23, $CHCl_3$). – [Daicel Chiralpak IG (0.46 cm × 25 cm); 90 : 10 v/v, *n*-hexane / isopropanol; v = 1.2 mL·min^{-1}, λ = 254 nm, t_R (major) = 26.67 min, t_R (minor) = 28.22 min]. – m.p.: 35–38 °C. – ^{1}H NMR (500 MHz, $CDCl_3$) *δ* 8.21–8.11 (m, 1H), 8.08–7.91 (m, 3H), 7.46–7.33 (m, 3H), 2.48 (s, 3H) ppm. – ^{13}C NMR (126 MHz, $CDCl_3$) *δ* 162.3 (d, *J* = 262.7 Hz), 144.9, 139.0 (d, *J* = 3.9 Hz), 138.0, 130.1, 129.8, 128.0 (d, *J* = 9.2 Hz), 127.5, 123.6 (d, *J* = 19.2 Hz), 118.1 (d, *J* = 23.0 Hz), 21.7 ppm. – ^{19}F NMR (471 MHz, $CDCl_3$) δ –100.8 ppm. – IR: 2926, 139, 1588, 1465, 1415, 1347, 1262, 1230, 1205, 1162, 1126, 1085, 1042, 949, 813, 735 cm^{-1}. – HRMS: calcd for $C_{13}H_{10}Cl_2FNO_3S_2$: 403.9355, found 403.9357 [M+Na$^+$].

3-Hydroxy-2,2-dimethylpropyl *(R)*-4-fluoro-*N*-tosyl-3-(trifluoromethyl)benzenesulfonimidate (3s)

***(S)*-4-Fluoro-*N*-tosyl-3-(trifluoromethyl)benzenesulfonimidoyl chloride (1s)**

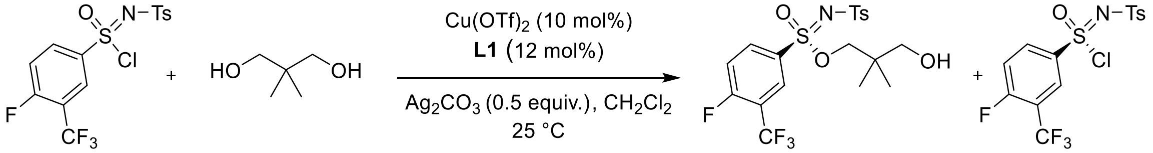

According to GP3, the reaction was carried out with sulfonimidoyl chloride ***rac*-1s** (41.5 mg, 0.1 mmol), and diol **2** (17.7 mg, 0.17 mmol) in anhydrous dichloromethane (1 mL). After stirring for 12 h, for work-up, a saturated solution of EDTA (1.5 mL) was added, followed by dichloromethane (5 mL). The organic phases were combined, dried over anhydrous Na_2SO_4, concentrated under reduced pressure, and the remaining material was purified by preparative TLC (hexane/ethyl acetate, 80 : 20 – 60 : 40) to give enantioenriched sulfonimidate product **3s** (19.8 mg, 41%, 92% ee) and sulfonimidoyl chloride **1s** (16.6 mg, 40%, 95% ee).

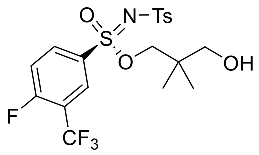

$[\alpha]_D$ = –30.0 (c 0.5, $CHCl_3$). – [Daicel Chiralpak IC (0.46 cm × 25 cm); 50 : 50 v/v, *n*-hexane / isopropanol; v = 1.2 mL·min^{-1}, λ = 254 nm, t_R (major) = 4.12 min, t_R (minor) = 5.33 min]. – ^{1}H NMR (500 MHz, $CDCl_3$) *δ* 8.25–8.10 (m, 2H), 7.84 (d, *J* = 8.1 Hz, 2H), 7.42 (t, *J* = 9.0 Hz, 1H), 7.29 (d, *J* = 7.4 Hz, 2H), 4.20–4.07 (m, 2H), 3.50–3.38 (m, 2H), 2.43 (s, 3H), 0.94 (d, *J* = 5.3 Hz, 6H) ppm. – ^{13}C NMR (126 MHz, $CDCl_3$) *δ* 163.1 (d, *J* = 268.2 Hz), 143.8, 139.2, 134.1 (d, *J* = 10.5

Hz), 132.4 (d, *J* = 3.7 Hz), 129.5, 128.0 – 127.5 (m), 126.8, 122.3, 120.6 – 119.7 (m), 118.7 (d, *J* = 22.4 Hz), 77.3, 67.0, 36.8, 21.5, 21.0 ppm. – ^{19}F NMR (471 MHz, $CDCl_3$) δ –61.8 (d, *J* = 12.5 Hz), –102.6 (dq, *J* = 14.7, 5.4 Hz) ppm. – IR: 3526, 2968, 1738, 1618, 1590, 1493, 1320, 1288, 1150, 1117, 1088, 1056, 912 cm^{-1}. – HRMS: calcd for $C_{19}H_{21}F_4NO_5S_2$: 484.0870, found 484.0869 [M+H$^+$].

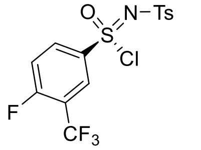

$[\alpha]_D$ = +40.0 (c 0.5, $CHCl_3$). – [Daicel Chiralpak IC (0.46 cm × 25 cm); 50 : 50 v/v, *n*-hexane / isopropanol; v = 1.2 mL·min^{-1}, λ = 254 nm, t_R (major) = 5.02 min, t_R (minor) = 4.35 min]. – m.p.: 71–73 °C. – ^{1}H NMR (500 MHz, $CDCl_3$) *δ* 8.34 (d, *J* = 5.2 Hz, 2H), 7.98 (d, *J* = 7.5 Hz, 2H), 7.51 (t, *J* = 9.0 Hz, 1H), 7.39 (d, *J* = 7.9 Hz, 2H), 2.48 (s, 3H) ppm. – ^{13}C NMR (126 MHz, $CDCl_3$) *δ* 163.6 (d, *J* = 270.8 Hz), 145.0, 138.8 (d, *J* = 3.8 Hz), 137.9, 133.4 (d, *J* = 10.7 Hz), 129.9, 127.5, 127.2 (q, *J* = 4.4 Hz), 121.0 (d, *J* = 273.7 Hz), 120.6 (dd, *J* = 35.0, 14.1 Hz), 119.2 (d, *J* = 22.6 Hz), 21.7 ppm. – ^{19}F NMR (471 MHz, $CDCl_3$) δ –61.9 (d, *J* = 12.7 Hz), –100.0 (ddd, *J* = 18.3, 9.5, 5.2 Hz) ppm. – IR: 3074, 1617, 1586, 1489, 1422, 1345, 1321, 1289, 1250, 1146, 1111, 1084, 1055, 907 cm^{-1}. – HRMS: calcd for $C_{14}H_{10}ClF_4NO_3S_2$: 433.0065, found 433.0055 [M+NH_4^+].

3-Hydroxy-2,2-dimethylpropyl *(R)*-4-chloro-*N*-tosyl-3-(trifluoromethyl)benzenesulfonimidate (3t)

***(S)*-4-Chloro-*N*-tosyl-3-(trifluoromethyl)benzenesulfonimidoyl chloride (1t)**

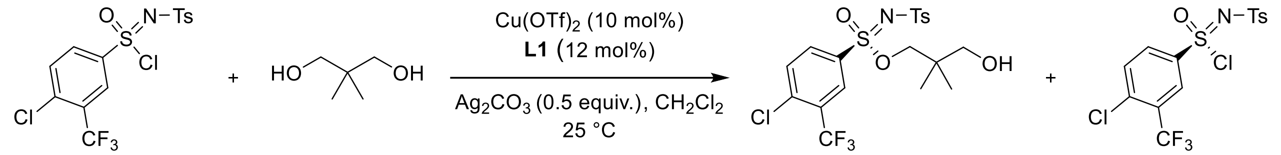

According to GP3, the reaction was carried out with sulfonimidoyl chloride ***rac*-1t** (43.2 mg, 0.1 mmol), and diol **2** (17.7 mg, 0.17 mmol) in anhydrous dichloromethane (1 mL). After stirring for 13 h, for work-up, a saturated solution of EDTA (1.5 mL) was added, followed by dichloromethane (5 mL). The organic phases were combined, dried over anhydrous Na_2SO_4, concentrated under reduced pressure, and the remaining material was purified by preparative TLC (hexane/ethyl acetate, 80 : 20 – 60 : 40) to give enantioenriched sulfonimidate product **3t** (21.5 mg, 43%, 91% ee) and sulfonimidoyl chloride **1t** (17.2 mg, 40%, 97% ee).

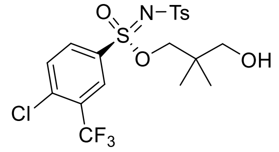

$[\alpha]_D$ = –11.9 (c 0.8, $CHCl_3$). – [Daicel Chiralpak IC (0.46 cm × 25 cm); 70 : 30 v/v, *n*-hexane / isopropanol; v = 1.2 mL·min^{-1}, λ = 254 nm, t_R (major) = 9.25 min, t_R (minor) = 13.97 min]. – ^{1}H NMR (500 MHz, $CDCl_3$) *δ* 8.17 (s, 1H), 8.07 (d, *J* = 8.5 Hz, 1H), 7.83 (d, *J* = 7.8 Hz, 2H), 7.72 (d, *J* = 8.5 Hz, 1H), 7.35–7.26 (m, 3H), 4.22 – 4.09 (m, 2H), 3.51–3.39 (m, 2H), 2.43 (s, 3H), 0.95 (d, *J* = 4.9 Hz, 6H) ppm. – ^{13}C NMR (126 MHz, $CDCl_3$) *δ* 143.8, 139.5, 139.2, 134.9, 132.8, 131.9, 129.5, 128.3, 127.0 (q, *J* = 5.5 Hz), 126.8, 122.7, 120.5, 77.0, 67.0, 36.8, 21.5, 21.0 ppm. – IR: 3521, 3075, 2923, 1738, 1570, 1417, 1324, 1282, 1150, 1112, 1087, 993, 918 cm^{-1}. – HRMS: calcd for $C_{19}H_{21}ClF_3NO_5S_2$: 500.0575, found 500.0563 [M+H$^+$].

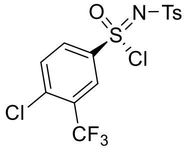

$[\alpha]_D$ = +19.7 (c 0.8, $CHCl_3$). – [Daicel Chiralpak IC (0.46 cm × 25 cm); 70 : 30 v/v, *n*-hexane / isopropanol; v = 1.2 mL·min^{-1}, λ = 254 nm, t_R (major) = 9.50 min, t_R (minor) = 8.03 min]. – m.p.: 50–52 °C. – ^{1}H NMR (500 MHz, $CDCl_3$) *δ* 8.34 (s, 1H), 8.20 (d, *J* = 8.6 Hz, 1H), 7.98 (d, *J* = 7.8 Hz, 2H), 7.81 (d, *J* = 8.7 Hz, 1H), 7.39 (d, *J* = 8.0 Hz, 2H), 2.48 (s, 3H) ppm. – ^{13}C NMR (126 MHz, $CDCl_3$) δ 145.0, 141.2, 140.8, 137.9, 133.3, 131.0, 130.5 (q, *J* = 33.4 Hz), 129.9, 127.5, 126.3 (q, *J* = 5.6 Hz),121.4 (q, *J* = 274.6 Hz), 21.7 ppm. – IR: 3100, 1595, 1567, 1467, 1405, 1345, 1290, 1164, 1106, 1082, 1033, 906 cm^{-1}. – HRMS: calcd for $C_{14}H_{10}Cl_2F_3NO_3S_2$: 431.9504, found 431.9499 [M+H$^+$].

3-Hydroxy-2,2-dimethylpropyl (*R*)-4-bromo-3-methyl-*N*-tosylbenzenesulfonimidate (3u)

(*S*)-4-Bromo-3-methyl-*N*-tosylbenzenesulfonimidoyl chloride (1u)

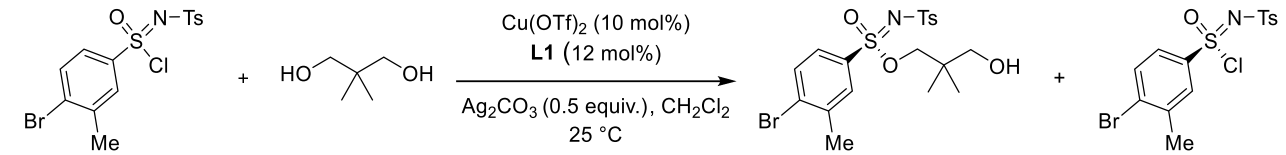

According to GP3, the reaction was carried out with sulfonimidoyl chloride ***rac*-1u** (42.2 mg, 0.1 mmol), and diol **2** (17.7 mg, 0.17 mmol) in anhydrous dichloromethane (1 mL). After stirring for 13 h, for work-up, a saturated solution of EDTA (1.5 mL) was added, followed by dichloromethane (5 mL). The organic phases were combined, dried over anhydrous Na_2SO_4, concentrated under reduced pressure, and the remaining material was purified by preparative TLC (hexane/ethyl acetate, 80 : 20 – 60 : 40) to give enantioenriched sulfonimidate product **3u** (20.7 mg, 42%, 93% ee) and sulfonimidoyl chloride **1u** (16.8 mg, 40%, 96% ee).

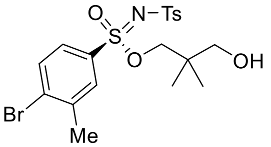

$[\alpha]_D$ = –17.3 (c .26, $CHCl_3$). – [Daicel Chiralpak IC (0.46 cm × 25 cm); 50 : 50 v/v, *n*-hexane / isopropanol; v = 1.2 mL·min^{-1}, λ = 254 nm, t_R (major) = 11.76 min, t_R (minor) = 17.27 min]. – ^{1}H NMR (500 MHz, $CDCl_3$) δ 7.85 (d, *J* = 8.0 Hz, 2H), 7.76 (d, *J* = 2.4 Hz, 1H), 7.72 (d, *J* = 8.4 Hz, 1H), 7.60 (dd, *J* = 8.5, 2.4 Hz, 1H), 7.33–7.23 (m, 2H), 4.05 (q, *J* = 9.0 Hz, 2H), 3.44 (s, 2H), 2.47 (s, 3H), 2.42 (s, 3H), 0.92 (d, *J* = 1.9 Hz, 6H) ppm. – ^{13}C NMR (126 MHz, $CDCl_3$) *δ* 143.4, 140.1, 139.6, 134.2, 133.5, 132.5, 129.3, 129.3, 126.8, 126.2, 116.4, 76.8, 67.1, 36.7, 23.1, 21.6, 21.1 ppm. – IR: 3521, 2961, 1738, 1465, 1319, 1278, 1156, 1118, 1088, 1028, 920 cm^{-1}. – HRMS: calcd for $C_{19}H_{24}BrNO_5S_2$: 490.0352, found 490.0344 [M+H$^+$].

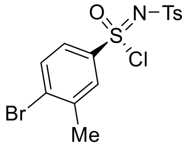

$[\alpha]_D$ = +11.5 (c 0.13, $CHCl_3$). – [Daicel Chiralpak IH (0.46 cm × 25 cm); 50 : 50 v/v, *n*-hexane / isopropanol; v = 1.2 mL·min^{-1}, λ = 254 nm, t_R (major) = 9.73 min, t_R (minor) = 7.75 min]. – m.p.: 70–73 °C. – ^{1}H NMR (500 MHz, $CDCl_3$) *δ* 7.98 (d, *J* = 8.0 Hz, 2H), 7.92 (s, 1H), 7.83–7.70 (m, 2H), 7.37 (d, *J* = 8.1 Hz, 2H), 2.53 (s, 3H), 2.47 (s, 3H) ppm. – ^{13}C NMR (126 MHz, $CDCl_3$) *δ* 144.6, 141.3, 140.8, 138.2, 133.8, 133.8, 129.7, 128.3, 127.5, 125.3, 23.3, 21.7 ppm. – IR: 2921, 1596, 1556, 1463, 1340, 1283, 1163, 1110, 1081, 1027, 877 cm^{-1}. – HRMS: calcd for $C_{14}H_{13}BrClNO_3S_2$: 421.9282, found 421.9280 [M+H$^+$].

3-Hydroxy-2,2-dimethylpropyl (*R*)-*N*-tosylnaphthalene-2-sulfonimidate (3v)

(*S*)-*N*-Tosylnaphthalene-2-sulfonimidoyl chloride (1v)

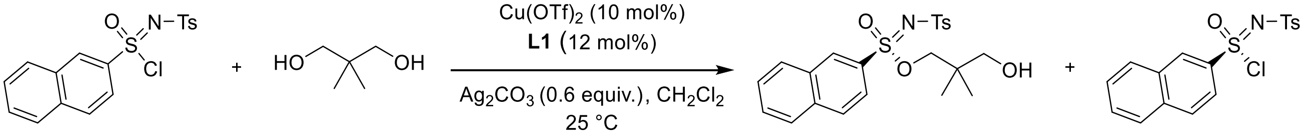

According to GP3, the reaction was carried out with sulfonimidoyl chloride ***rac*-1v** (38.0 mg, 0.1 mmol), and diol **2** (17.7 mg, 0.17 mmol) in anhydrous dichloromethane (1 mL). After stirring for 17 h, for work-up, a saturated solution of EDTA (1.5 mL) was added, followed by dichloromethane (5 mL). The organic phases were combined, dried over anhydrous Na_2SO_4, concentrated under reduced pressure, and the remaining material was purified by preparative TLC (hexane/ethyl acetate, 80: 20 – 60: 40) to give enantioenriched sulfonimidate product **3v** (19.7 mg, 44%, 90% ee) and sulfonimidoyl chloride **1v** (15.6 mg, 41%, 97% ee).

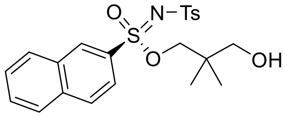

$[\alpha]_D$ = –43.5 (c 0.4, $CHCl_3$). – [Daicel Chiralpak IBN (0.46 cm × 25 cm); 70 : 30 v/v, *n*-hexane / isopropanol; v = 1.2 mL·min^{-1}, λ = 254 nm, t_R (major) = 4.53 min, t_R (minor) = 5.18 min]. – ^{1}H NMR (500 MHz, $CDCl_3$) δ 8.52 (s, 1H), 8.05–7.82 (m, 6H), 7.73 (t, *J* = 7.1 Hz, 1H), 7.68 (t, *J* = 7.5 Hz, 1H), 7.25 (d, *J* = 8.1 Hz, 2H), 4.19–3.99 (m, 2H), 3.47 (d, *J* = 5.4 Hz, 2H), 2.38 (s, 3H), 0.91 (d, *J* = 3.2 Hz, 6H) ppm. – ^{13}C NMR (126 MHz, $CDCl_3$) δ 143.3, 139.8, 135.5, 131.8, 131.7, 130.0, 130.0, 129.9, 129.6, 129.3, 128.1, 128.0, 126.8, 121.7, 76.7, 67.2, 36.8, 21.5, 21.1 ppm. – IR: 3522, 2969, 1738, 1597, 1320, 1285, 1157, 1108, 1088, 919, 867, 813, 736 cm^{-1}. – HRMS: calcd for $C_{22}H_{25}NO_5S_2$: 448.1247, found 448.1248 [M+H$^+$].

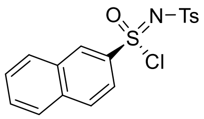

[α]D = +51.3 (c 0.45, $CHCl_3$). – [Daicel Chiralpak IC (0.46 cm × 25 cm); 70 : 30 v/v, *n*-hexane / isopropanol; v = 1.2 mL·min^{-1}, λ = 254 nm, t_R (major) = 26.84 min, t_R (minor) = 24.56 min]. m.p.: 56–58 °C. – ^{1}H NMR (500 MHz, $CDCl_3$) δ 8.66 (d, *J* = 2.1 Hz, 1H), 8.10–7.93 (m, 6H), 7.78 (ddd, *J* = 8.2, 6.9, 1.3 Hz, 1H), 7.72 (ddd, *J* = 8.2, 6.9, 1.3 Hz, 1H), 7.39 (d, *J* = 8.1 Hz, 2H), 2.47 (s, 3H) ppm. – ^{13}C NMR (126 MHz, $CDCl_3$) δ 144.5, 139.2, 138.5, 135.9, 131.6, 130.8, 130.5, 130.0, 129.7, 129.1, 128.6, 128.2, 127.6, 120.9, 21.7 ppm. – IR: 2969, 1738, 1595, 1456, 1384, 1230, 1205, 1152, 1108, 1087, 895, 815, 733 cm^{-1}. – HRMS: calcd for $C_{17}H_{14}ClNO_3S_2$: 380.0176, found 380.0172 [M+H$^+$].

3-Hydroxy-2,2-dimethylpropyl (*R*)-*N*-((4-fluorophenyl)sulfonyl)benzenesulfonimidate (3w)

(*S*)-*N*-((4-Fluorophenyl)sulfonyl)benzenesulfonimidoyl chloride (1w)

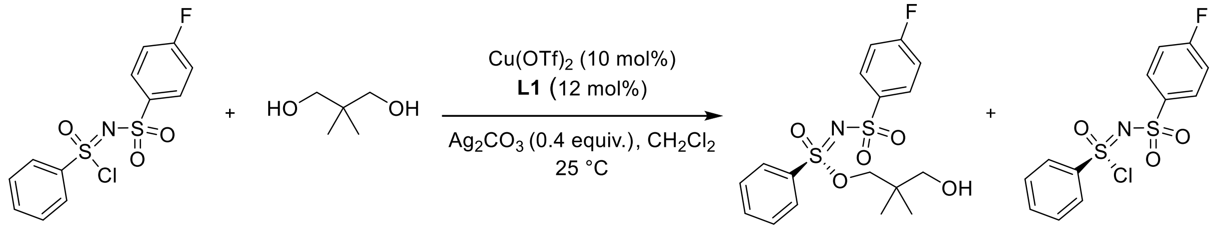

According to GP3, the reaction was carried out with sulfonimidoyl chloride ***rac*-1w** (33.3 mg, 0.1 mmol), and diol **2** (17.7 mg, 0.17 mmol) in anhydrous dichloromethane (1 mL). After stirring for 12.5 h, for work-up, a saturated solution of EDTA (1.5 mL) was added, followed by dichloromethane (5 mL). The organic phases were combined, dried over anhydrous Na_2SO_4, concentrated under reduced pressure, and the remaining material was purified by preparative TLC (hexane/ethyl acetate, 80 : 20 – 60 : 40) to give the enantioenriched sulfonimidate product **3w** (17.2 mg, 43%, 92% ee) and sulfonimidoyl chloride **1w** (13.3 mg, 40%, 96% ee).

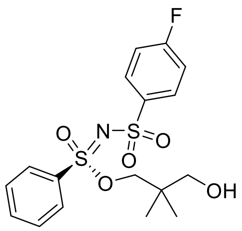

[α]D = –56.1 (c 0.63, $CHCl_3$). – [Daicel Chiralpak IBN (0.46 cm × 25 cm); 90 : 10 v/v, *n*-hexane / isopropanol; v = 1.2 mL·min^{-1}, λ = 254 nm, t_R (major) = 13.41 min, t_R (minor) = 15.96 min]. – ^{1}H NMR (500 MHz $CDCl_3$) *δ* 8.07–7.88 (m, 4H), 7.72 (t, *J* = 7.5 Hz, 1H), 7.59 (t, *J* = 7.8 Hz, 2H), 7.16 (t, *J* = 8.6 Hz, 2H), 4.11–4.01 (m, 2H), 3.45 (s, 2H), 0.92 (d, *J* = 1.9 Hz, 6H) ppm. – ^{13}C NMR (126 MHz, $CDCl_3$) *δ* 165.0 (d, *J* = 254.3 Hz), 138.7 (d, *J* = 3.6 Hz), 135.2, 134.8, 129.6, 129.5, 127.6, 115.9 (d, *J* = 22.7 Hz), 76.8, 67.1, 36.7, 21.1 ppm. – ^{19}F NMR (471 MHz, $CDCl_3$) *δ* –105.63. – IR: 3522, 2926, 1591, 1494, 1448, 1325, 1289, 1166, 1152, 1118, 1089, 937, 836, 755 cm^{-1}. – HRMS: calcd for $C_{17}H_{20}FNO_5S_2$: 402.0840, found 402.0832 [M+H$^+$].

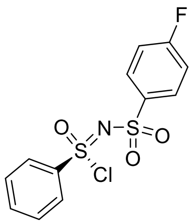

[α]D = +112.0 (c 0.4, $CHCl_3$). – [Daicel Chiralpak IG (0.46 cm × 25 cm); 70 : 30 v/v, *n*-hexane / isopropanol; v = 1.2 mL·min^{-1}, λ = 254 nm, t_R (major) = 15.28 min, t_R (minor) = 11.46 min]. m.p.: 52–54 °C. – ^{1}H NMR (500 MHz, $CDCl_3$) *δ* 8.17–8.05 (m, 4H), 7.79 (t, *J* = 7.5 Hz, 1H), 7.71–7.62 (m, 2H), 7.31–7.22 (m, 2H) ppm. – ^{13}C NMR (126 MHz, $CDCl_3$) *δ* 165.6 (d, *J* = 256.0 Hz), 142.5, 137.3 (d, *J* = 3.3 Hz), 135.8, 130.4 (d, *J* = 9.3 Hz), 129.9, 126.8, 116.4 (d, *J* = 22.7 Hz) ppm. – ^{19}F NMR (471 MHz, $CDCl_3$) δ –103.70 ppm. – IR: 2922, 2851, 1738, 1661, 1589, 1494, 1456, 1346, 1290, 1231, 1125, 1087, 1042, 997, 838, 738 cm^{-1} – HRMS: calcd for $C_{12}H_9ClFNO_3S_2$: 355.9589, found 355.9577 [M+Na$^+$].

3-Hydroxy-2,2-dimethylpropyl (*R*)-*N*-((3-fluorophenyl)sulfonyl)benzenesulfonimidate (3x)

(*S*)-*N*-((3-Fluorophenyl)sulfonyl)benzenesulfonimidoyl chloride (1x)

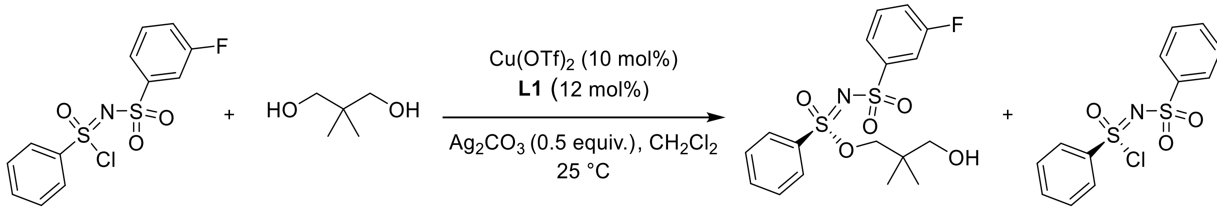

According to GP3, the reaction was carried out with sulfonimidoyl chloride ***rac*-1x** (33.3 mg, 0.1 mmol), and diol **2** (17.7 mg, 0.17 mmol) in anhydrous dichloromethane (1 mL). After stirring for 12.5 h, for work-up, a saturated solution of EDTA (1.5 mL) was added, followed by dichloromethane (5 mL). The organic phases were combined, dried over anhydrous Na_2SO_4, concentrated under reduced pressure, and the remaining material was purified by preparative TLC (hexane/ethyl acetate, 80 : 20 – 60 : 40) to give the enantioenriched sulfonimidate product **3x** (16.4 mg, 41%, 91% ee) and sulfonimidoyl chloride **1x** (13.3 mg, 40%, 99% ee).

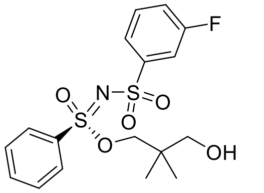

$[\alpha]_D$ = –25.1 (c 0.42, $CHCl_3$). – [Daicel Chiralpak IBN (0.46 cm × 25 cm); 90 : 10 v/v, *n*-hexane / isopropanol; v = 1.2 mL·min^{-1}, λ = 254 nm, t_R (major) = 11.6 min, t_R (minor) = 13.44 min]. – ^{1}H NMR (500 MHz, $CDCl_3$) δ 7.96 (d, *J* = 8.2 Hz, 2H), 7.79 (d, *J* = 7.8 Hz, 1H), 7.71 (dt, *J* = 16.4, 7.8 Hz, 2H), 7.60 (t, *J* = 7.7 Hz, 2H), 7.50 (s, 1H), 7.26 (t, *J* = 9.4 Hz, 1H), 4.07 (s, 2H), 3.45 (s, 2H), 0.92 (d, *J* = 2.3 Hz, 6H) ppm. – ^{13}C NMR (126 MHz $CDCl_3$) δ 162.1 (d, *J* = 250.8 Hz), 144.5 (d, *J* = 6.8 Hz), 135.1, 134.8, 130.5 (d, *J* = 7.5 Hz), 129.5, 127.7, 122.6 (d, *J* = 3.6 Hz), 119.7 (d, *J* = 21.2 Hz), 114.3 (d, *J* = 24.8 Hz), 76.9, 67.2, 36.8, 21.1 ppm. – ^{19}F NMR (471 MHz, $CDCl_3$) *δ* –110.26 ppm. – IR: 3522, 2969, 1738, 1593, 1477, 1449, 1328, 1303, 1272, 1226, 1151, 1119, 1085, 998, 938, 852, 755 cm^{-1}. – HRMS: calcd for $C_{17}H_{20}FNO_5S_2$: 402.0840, found 402.0847 [M+H$^+$].

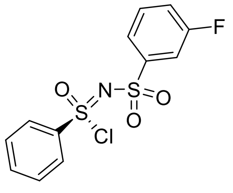

$[\alpha]_D$ = +37.0 (c 0.1, $CHCl_3$). – [Daicel Chiralpak IH (0.46 cm × 25 cm); 50 : 50 v/v, *n*-hexane / isopropanol; v = 1.2 mL·min^{-1}, λ = 254 nm, t_R (major) = 6.64 min, t_R (minor) = 5.47 min]. – m.p.: 39–41 °C. – ^{1}H NMR (500 MHz, $CDCl_3$) *δ* 8.09 (d, *J* = 8.5 Hz, 2H), 7.91 (d, *J* = 7.8 Hz, 1H), 7.80 (t, *J* = 7.0 Hz, 2H), 7.66 (t, *J* = 7.8 Hz, 2H), 7.61–7.52 (m, 1H), 7.41–7.31 (m, 1H) ppm. – ^{13}C NMR (126 MHz, $CDCl_3$) *δ* 162.3 (d, *J* = 251.7 Hz), 143.1 (d, *J* = 7.1 Hz), 142.4, 135.8, 130.9 (d, *J* = 7.5 Hz), 129.9, 126.8, 123.2 (d, *J* = 3.5 Hz), 120.7 (d, *J* = 21.1 Hz), 114.9 (d, *J* = 24.7 Hz) ppm. – ^{19}F NMR (471 MHz, $CDCl_3$) δ –109.39 ppm. – IR: 2939, 1739, 1589, 1456, 1435, 1347, 1230, 1206, 1162, 1125, 1083, 950, 811, 749 cm^{-1}. – HRMS: calcd for $C_{12}H_9ClFNO_3S_2$: 355.9589, found 355.9594 [M+Na$^+$].

3-Hydroxy-2,2-dimethylpropyl (*R*)-*N*-((3,4-difluorophenyl)sulfonyl)benzenesulfonimidate (3y)

(*S*)-*N*-((3,4-Difluorophenyl)sulfonyl)benzenesulfonimidoyl chloride (1y)

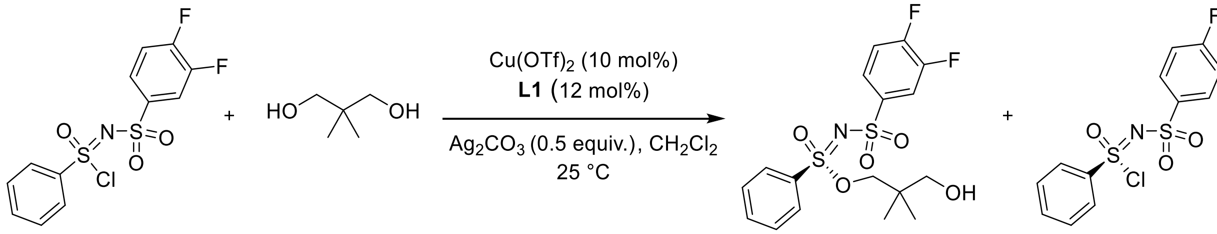

According to GP3, the reaction was carried out with sulfonimidoyl chloride ***rac*-1y** (35.1 mg, 0.1 mmol), and diol **2** (17.7 mg, 0.17 mmol) in anhydrous dichloromethane (1 mL). After stirring for 13 h, for work-up, a saturated solution of EDTA (1.5 mL) was added, followed by dichloromethane (5 mL). The organic phases were combined, dried over anhydrous Na_2SO_4, concentrated under reduced pressure, and the remaining material was purified by preparative TLC (hexane/ethyl acetate, 80: 20 – 60: 40) to give the enantioenriched sulfonimidate product **3y** (17.6 mg, 42%, 90% ee) and sulfonimidoyl chloride **1y** (14.3 mg, 40%, 98% ee).

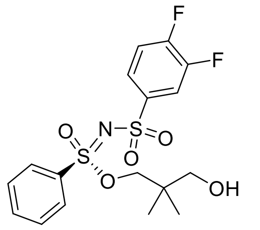

[α]D = –5.9 (c 0.7, $CHCl_3$). – [Daicel Chiralpak IBN (0.46 cm × 25 cm); 90 : 10 v/v, *n*-hexane / isopropanol; v = 1.2 mL·min^{-1}, λ = 254 nm, t_R (major) = 11.54 min, t_R (minor) = 13.71 min]. – ^{1}H NMR (500 MHz, $CDCl_3$) δ 7.96 (d, *J* = 8.1 Hz, 2H), 7.87–7.68 (m, 3H), 7.61 (t, *J* = 7.8 Hz, 2H), 7.33 – 7.22 (m, 1H), 4.06 (s, 2H), 3.44 (s, 2H), 0.93 (s, 6H) ppm. – ^{13}C NMR (126 MHz, $CDCl_3$) δ 154.0 (d, *J* = 12.6 Hz), 151.9 (d, *J* = 12.6 Hz), 150.8 (d, *J* = 13.5 Hz), 148.8 (d, *J* = 13.1 Hz), 135.0 (d, *J* = 6.5 Hz), 129.6, 127.6, 123.9 (dd, *J* = 7.5, 3.9 Hz), 117.8 (d, *J* = 18.3 Hz), 116.9 (d, *J* = 20.0 Hz), 77.0, 67.1, 36.7, 21.0 ppm. – ^{19}F NMR (471 MHz, $CDCl_3$) δ –130.0 (dt, *J* = 18.4, 8.4 Hz), –134.3 (dt, *J* = 18.3, 8.6 Hz) ppm. – IR: 3525, 3062, 2964, 1611, 1508, 1449, 1417, 1330, 1277, 1153, 1116, 1087, 913, 856, 755 cm^{-1}. – HRMS: calcd for $C_{17}H_{19}F2NO_5S_2$: 420.0745, found 420.0739 [M+H$^+$].

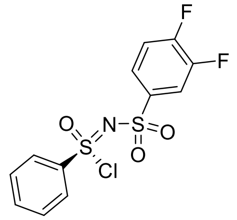

[α]D = +19.7 (c 0.33, $CHCl_3$). – [Daicel Chiralpak IH (0.46 cm × 25 cm); 50 : 50 v/v, *n*-hexane / isopropanol; v = 1.2 mL·min^{-1}, λ = 254 nm, t_R (major) = 6.41 min, t_R (minor) = 5.11 min]. – m.p.: 65–67 °C. – ^{1}H NMR (500 MHz, $CDCl_3$) δ 8.09 (d, *J* = 8.1 Hz, 2H), 8.00–7.87 (m, 2H), 7.81 (t, *J* = 7.4 Hz, 1H), 7.67 (t, *J* = 7.8 Hz, 2H), 7.37 (q, *J* = 8.7 Hz, 1H) ppm. – ^{13}C NMR (126 MHz, $CDCl_3$) δ 153.7 (d, *J* = 246.1 Hz), 150.1 (d, *J* = 241.3 Hz), 154.7 (d, *J* = 12.2 Hz), 152.6 (d, *J* = 12.5 Hz), 151.1 (d, *J* = 13.5 Hz), 149.0 (d, *J* = 13.6 Hz), 142.3, 138.0 (d, *J* = 4.7 Hz), 135.9, 130.0, 126.8, 124.7 (dd, *J* = 7.8, 4.1 Hz), 118.2 (d, *J* = 18.3 Hz), 117.5 (d, *J* = 20.2 Hz) ppm. – ^{19}F NMR (471 MHz, $CDCl_3$) δ –122.70, –130.23 ppm. – IR: 2924, 1742, 1590, 1508, 1456, 1417, 1347, 1278, 1206, 1161, 1125, 1069, 997, 914, 820, 744 cm^{-1}. – HRMS: calcd for $C_{12}H_8ClF_2NO_3S_2$: 351.9675, found 351.9665 [M+H$^+$].

3-Hydroxy-2,2-dimethylpropyl (*R*)-*N*-((4-bromophenyl)sulfonyl)benzenesulfonimidate (3z)

(*S*)-*N*-((4-Bromophenyl)sulfonyl)benzenesulfonimidoyl chloride (1z)

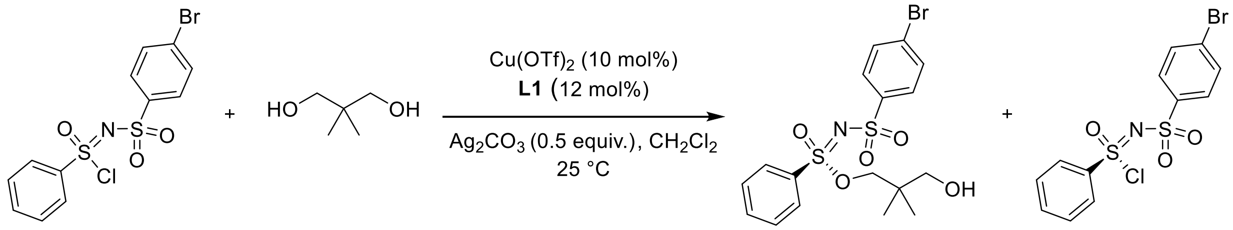

According to GP3, the reaction was carried out with sulfonimidoyl chloride ***rac*-1z** (39.5 mg, 0.1 mmol), and diol **2** (17.7 mg, 0.17 mmol) in anhydrous dichloromethane (1 mL). After stirring for 12 h, for work-up, a saturated solution of EDTA (1.5 mL) was added, followed by dichloromethane (5 mL). The organic phases were combined, dried over anhydrous Na_2SO_4, concentrated under reduced pressure, and the remaining material was purified by preparative TLC (hexane/ethyl acetate, 80: 20 – 60: 40) to give the enantioenriched sulfonimidate product **3z** (18.5 mg, 40%, 92% ee) and sulfonimidoyl chloride **1z** (17.0 mg, 43%, 98% ee).

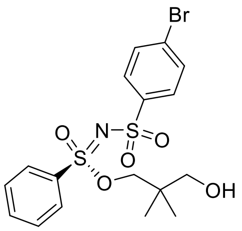

[α]D = –10.7 (c 0.14, $CHCl_3$). – [Daicel Chiralpak IH (0.46 cm × 25 cm); 50 : 50 v/v, *n*-hexane / isopropanol; v = 1.2 mL·min^{-1}, λ = 210 nm, t_R (major) = 7.18 min, t_R (minor) = 5.34 min]. – ^{1}H NMR (500 MHz, $CDCl_3$) δ 7.95 (d, *J* = 8.3 Hz, 2H), 7.85 (d, *J* = 8.4 Hz, 2H), 7.73 (t, *J* = 7.4 Hz, 1H), 7.66–7.54 (m, 4H), 4.05 (d, *J* = 1.6 Hz, 2H), 3.44 (s, 2H), 0.92 (d, *J* = 2.0 Hz, 6H) ppm. – ^{13}C NMR (126 MHz, $CDCl_3$) δ 135.1, 134.8, 132.0, 129.5, 128.4, 127.6, 127.5, 76.9, 67.1, 36.7, 21.1 ppm. – IR: 3526, 2963, 1574, 1471, 1448, 1389, 1323, 1273, 1157, 1115, 1087, 1067, 1009, 998, 918 cm^{-1}. – HRMS: calcd for $C_{17}H_{20}BrNO_5S_2$: 462.0039, found 462.0038 [M+H$^+$].

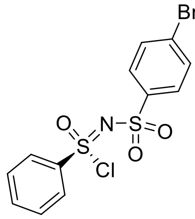

[α]D = +33.0 (c 0.9, $CHCl_3$). – [Daicel Chiralpak IH (0.46 cm × 25 cm); 50 : 50 v/v, *n*-hexane / isopropanol; v = 1.2 mL·min^{-1}, λ = 254 nm, t_R (major) = 11.71 min, t_R (minor) = 8.09 min]. – m.p.: 85–88 °C. – ^{1}H NMR (300 MHz, $CDCl_3$) δ 8.13–8.05 (m, 2H), 8.02–7.93 (m, 2H), 7.85–7.76 (m, 1H), 7.76–7.61 (m, 4H) ppm. – ^{13}C NMR (125 MHz, $CDCl_3$): 141.5, 129.4, 122.8, 118.1, 55.9, 31.8, 29.3, 29.24, 29.20, 28.6, 23.4, 22.7, 14.1 ppm. – IR: 3092, 1738, 1574, 1449, 1390, 1346, 1282, 1167, 1113,

1086, 1067, 1022, 1009, 824, 761 cm-1. – HRMS: calcd for $C_{12}H_9BrClNO_3S_2$: 393.8969, found 393.8973 [M+H+].

3-Hydroxy-2,2-dimethylpropyl (*R*)-*N*-((3-(trifluoromethyl)phenyl)sulfonyl)benzenesulfonimidate (3aa)

(*S*)-*N*-((3-(Trifluoromethyl)phenyl)sulfonyl)benzenesulfonimidoyl chloride (1aa)

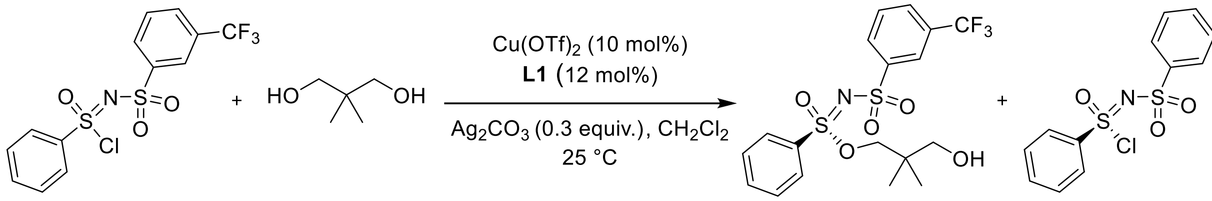

According to GP3, the reaction was carried out with sulfonimidoyl chloride ***rac*-1aa** (38.4 mg, 0.1 mmol), and diol **2** (17.7 mg, 0.17 mmol) in anhydrous dichloromethane (1 mL). After stirring for 12.5 h, for work-up, a saturated solution of EDTA (1.5 mL) was added, followed by dichloromethane (5 mL). The organic phases were combined, dried over anhydrous Na_2SO_4, concentrated under reduced pressure, and the remaining material was purified by preparative TLC (hexane/ethyl acetate, 80: 20 – 60: 40) to give the enantioenriched sulfonimidate product **3aa** (19.8 mg, 44%, 95% ee) and sulfonimidoyl chloride **1aa** (15.7 mg, 41%, 94% ee).

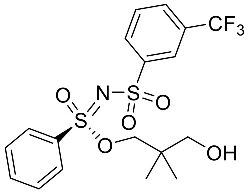

$[\alpha]_D$ = –9.1 (c 0.18, $CHCl_3$). – [Daicel Chiralpak IBN (0.46 cm × 25 cm); 90 : 10 v/v, *n*-hexane / isopropanol; v = 1.2 mL·min-1, λ = 254 nm, t_R (major) = 9.35 min, t_R (minor) = 10.97 min]. – ^{1}H NMR (500 MHz, $CDCl_3$) δ 8.28–8.15 (m, 2H), 7.94 (d, *J* = 8.0 Hz, 2H), 7.80 (d, *J* = 7.7 Hz, 1H), 7.72 (t, *J* = 7.4 Hz, 1H), 7.61 (dt, *J* = 25.5, 7.8 Hz, 3H), 4.07 (q, *J* = 8.9 Hz, 2H), 3.44 (d, *J* = 2.6 Hz, 2H), 0.95–0.82 (m, 6H) ppm. – ^{13}C NMR (126 MHz, $CDCl_3$) δ 143.7, 134.9, 131.3 (q, *J* = 33.4 Hz), 130.1, 129.6, 129.1 (d, *J* = 3.7 Hz), 129.0, 127.6, 124.3, 124.0 (d, *J* = 3.8 Hz), 122.2, 77.0, 67.2, 36.7, 21.0 ppm. – ^{19}F NMR (471 MHz, $CDCl_3$) δ –62.7 ppm. – IR: 3566, 2969, 1738, 1449, 1327, 1279, 1159, 1128, 1102, 998, 913, 824 cm-1. – HRMS: calcd for $C_{18}H_{20}F_3NO_5S_2$: 452.0808, found 452.0806 [M+H+].

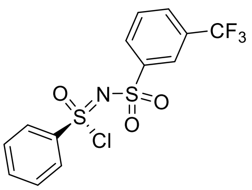

$[\alpha]_D$ = +37.3 (c 0.33, $CHCl_3$). – [Daicel Chiralpak IH (0.46 cm × 25 cm); 50 : 50 v/v, *n*-hexane / isopropanol; v = 1.2 mL·min-1, λ = 254 nm, t_R (major) = 4.07 min, t_R (minor) = 3.65 min]. – m.p.: 38–40 °C. – ^{1}H NMR (500 MHz, $CDCl_3$) δ 8.37 (s, 1H), 8.31 (d, *J* = 7.6 Hz, 1H), 8.09 (d, *J* = 8.0 Hz, 2H), 7.91 (d, *J* = 7.8 Hz, 1H), 7.81 (t, *J* = 7.5 Hz, 1H), 7.74 (t, *J* = 7.9 Hz, 1H), 7.67 (t, *J* = 7.8 Hz, 2H) ppm. – ^{13}C NMR (126 MHz, $CDCl_3$) δ 142.4 (d, *J* = 12.2 Hz), 135.9, 131.8 (q, *J* = 33.6 Hz), 130.7, 130.1 (q, *J* = 3.78 Hz), 130.0, 129.9, 126.9, 124.7 (q, *J* = 3.3 Hz), 123.1 (d, *J* = 272.9 Hz) ppm. – ^{19}F NMR (471 MHz, $CDCl_3$) δ –62.8 ppm. – IR: 2969, 1738, 1590, 1456, 1349, 1327, 1230, 1206, 1164, 1127, 895 cm-1. – HRMS: calcd for $C_{13}H_9ClF_3NO_3S_2$: 383.9797, found 383.9734 [M+H+].

3-Hydroxy-2,2-dimethylpropyl (*R*)-*N*-((3-chloro-4-methoxyphenyl)sulfonyl)benzenesulfonimidate (3ab)

(*S*)-*N*-((3-Chloro-4-methoxyphenyl)sulfonyl)benzenesulfonimidoyl chloride (1ab)

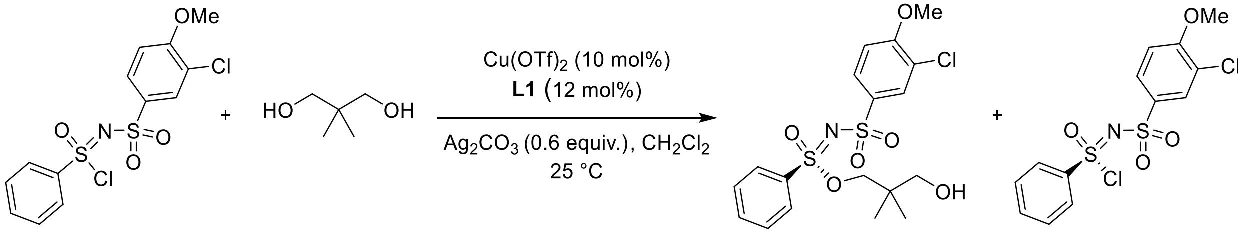

According to GP3, the reaction was carried out with sulfonimidoyl chloride ***rac*-1ab** (38.0 mg, 0.1 mmol), and diol **2** (17.7 mg, 0.17 mmol) in anhydrous dichloromethane (1 mL). After stirring for 12 h, for work-up, a saturated solution of EDTA (1.5 mL) was added, followed by dichloromethane (5 mL). The organic phases were combined, dried over anhydrous Na_2SO_4, concentrated under reduced pressure, and the remaining material was purified by preparative

TLC (hexane/ethyl acetate, 80: 20 – 60: 40) to give the enantioenriched sulfonimidate product **3ab** (18.9 mg, 42%, 90% ee) and sulfonimidoyl chloride **1ab** (15.1 mg, 40%, 97% ee).

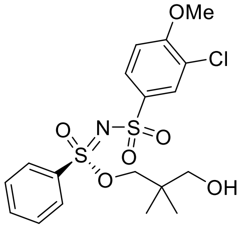

[α]D = –29.1 (c 0.47, CHCl3). – [Daicel Chiralpak IBN (0.46 cm × 25 cm); 70 : 30 v/v, *n*-hexane / isopropanol; v = 1.2 mL·min^{-1}, *λ* = 210 nm, t_R (major) = 5.42 min, t_R (minor) = 6.12 min]. – ^{1}H NMR (500 MHz, CDCl3) *δ* 8.01–7.93 (m, 3H), 7.88 (d, *J* = 8.7 Hz, 1H), 7.72 (t, *J* = 7.4 Hz, 1H), 7.59 (t, *J* = 7.7 Hz, 2H), 6.98 (d, *J* = 8.7 Hz, 1H), 4.06 (q, *J* = 9.0 Hz, 2H), 3.97 (s, 3H), 3.45 (s, 2H), 0.92 (s, 6H) ppm. – ^{13}C NMR (126 MHz, CDCl3) *δ* 158.2, 135.2, 135.2, 134.8, 129.5, 129.0, 127.7, 127.2, 122.8, 111.3, 76.8, 67.2, 56.5, 36.8, 21.1 ppm. – IR: 3522, 2924, 1738, 1586, 1491, 1448, 1323, 1274, 1157, 1119, 1100, 1062, 937, 851, 755 cm^{-1}. – HRMS: calcd for $C_{18}H_{22}ClNO_6S_2$: 448.0650, found 448.0654 [M+H$^+$].

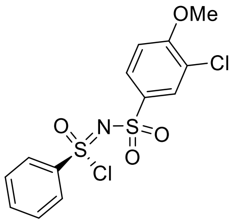

[α]D = +110.1 (c 0.49, CHCl3). – [Daicel Chiralpak IH (0.46 cm × 25 cm); 50 : 50 v/v, *n*-hexane / isopropanol; v = 1.2 mL·min^{-1}, *λ* = 254 nm, t_R (major) = 12.25 min, t_R (minor) = 10.98 min]. – m.p.: 85–87 °C. – ^{1}H NMR (500 MHz, CDCl3) *δ* 8.09 (d, *J* = 8.6 Hz, 3H), 8.01 (d, *J* = 8.7 Hz, 1H), 7.79 (t, *J* = 7.4 Hz, 1H), 7.66 (t, *J* = 7.8 Hz, 2H), 7.06 (d, *J* = 8.7 Hz, 1H), 4.01 (s, 3H) ppm. – ^{13}C NMR (126 MHz, CDCl3) *δ* 159.0, 142.5, 135.7, 133.6, 129.9, 129.6, 128.0, 126.9, 123.3, 111.5, 56.6 ppm. – IR: 2923, 1738, 1584, 1490, 1449, 1343, 1276, 1166, 1096, 1062, 1018, 850, 738 cm^{-1}. – HRMS: calcd for $C_{13}H_{11}Cl_2NO_4S_2$: 401.9399, found 401.9402 [M+Na$^+$].

3-Hydroxy-2,2-dimethylpropyl (*R*)-*N*-(cyclopropylsulfonyl)benzenesulfonimidate (3ac)

(*S*)-*N*-(Cyclopropylsulfonyl)benzenesulfonimidoyl chloride (1ac)

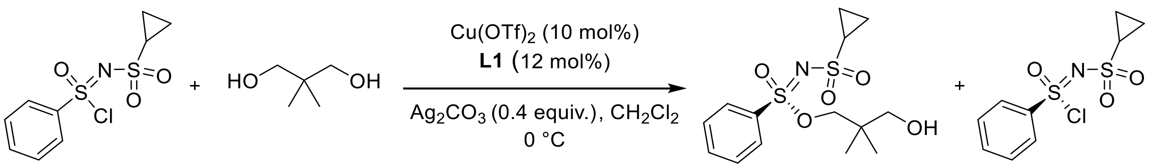

According to GP3, the reaction was carried out with sulfonimidoyl chloride ***rac*-1ac** (27.9 mg, 0.1 mmol), and diol **2** (17.7 mg, 0.17 mmol) in anhydrous dichloromethane (1 mL). After stirring for 13 h, for work-up, a saturated solution of EDTA (1.5 mL) was added, followed by dichloromethane (5 mL). The organic phases were combined, dried over anhydrous Na_2SO_4, concentrated under reduced pressure, and the remaining material was purified by preparative TLC (hexane/ethyl acetate, 80: 20 – 60: 40) to give the enantioenriched sulfonimidate product **3ac** (14.9 mg, 43%, 92% ee) and sulfonimidoyl chloride **1ac** (11.4 mg, 41%, 95% ee).

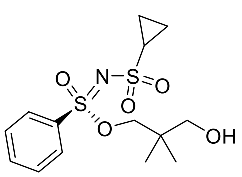

[α]D = –15.9 (c 0.33, CHCl3). – [Daicel Chiralpak IH (0.46 cm × 25 cm); 50 : 50 v/v, *n*-hexane / isopropanol; v = 1.2 mL·min^{-1}, *λ* = 254 nm, t_R (major) = 5.09 min, t_R (minor) = 3.83 min]. – ^{1}H NMR (500 MHz, CDCl3) *δ* 8.05 (d, *J* = 8.3 Hz, 2H), 7.75 (t, *J* = 7.4 Hz, 1H), 7.64 (t, *J* = 7.8 Hz, 2H), 4.10 (d, *J* = 8.9 Hz, 1H), 3.96 (d, *J* = 8.9 Hz, 1H), 3.48–3.41 (m, 2H), 2.77 (tt, *J* = 8.3, 4.8 Hz, 1H), 1.42–1.25 (m, 2H), 1.05 (d, *J* = 9.4 Hz, 2H), 0.92 (s, 6H) ppm. – ^{13}C NMR (126 MHz, CDCl3) *δ* 135.4, 134.7, 129.5, 127.7, 127.7, 76.8, 67.1, 36.8, 33.9, 21.1, 6.2, 6.2 ppm. – IR: 3501, 2925, 1449,1324, 1295, 1122, 1089, 939, 888, 851 cm^{-1}. – HRMS: calcd for $C_{14}H_{21}NO_5S_2$: 348.0934, found 348.0928 [M+H$^+$].

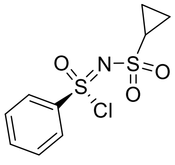

[α]D = +131.0 (c 0.6, CHCl3). – [Daicel Chiralpak IH (0.46 cm × 25 cm); 50 : 50 v/v, *n*-hexane / isopropanol; v = 1.2 mL·min^{-1}, *λ* = 254 nm, t_R (major) = 7.62 min, t_R (minor) = 6.50 min]. – m.p.: 33–35 °C. – ^{1}H NMR (500 MHz, CDCl3) *δ* 8.22–8.13 (m, 2H), 7.82 (t, *J* = 7.4 Hz, 1H), 7.69 (t, *J* = 7.9 Hz, 2H), 2.94–2.79 (m, 1H), 1.55–1.40 (m, 2H), 1.22–1.12 (m, 2H) ppm. – ^{13}C NMR (126 MHz, CDCl3) *δ* 142.6, 135.7, 129.9, 127.0, 34.1, 6.8, 6.7 ppm. – IR: 2922, 2851, 1660, 1449, 1337, 1286, 1152, 1115, 1071, 1022, 886, 744 cm^{-1}. – HRMS: calcd for $C_9H_{10}ClNO_3S_2$: 279.9863, found 279.9857 [M+H$^+$].

(*R*)-*N*-Tosylbenzenesulfonimidoyl fluoride (4)

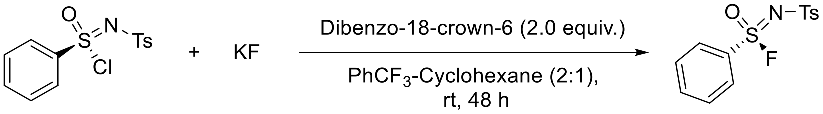

According to GP4, the reaction was carried out with **1a** (97% ee) (33.0 mg, 0.1 mmol), potassium fluoride (5.8 mg, 0.1 mmol) and dibenzo-18-crown-6 (72.0 mg, 0.2 mmol) in anhydrous $PhCF_3$-cyclohexane (2:1) (1 mL). After the completion of the reaction, for work-up, a saturated solution of brine (1.5 mL) was added, followed by ethyl acetate (5 mL). The organic phases were combined, dried over anhydrous Na_2SO_4, concentrated under reduced pressure, and the remaining material was purified by preparative TLC (hexane/ethyl acetate, 80 : 20 – 60 : 40) to give enantioenriched product **4** (22.0 mg, 70%, 95% ee).

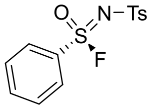

$[\alpha]_D$ = +50 (c 0.4, $CHCl_3$). – [Daicel Chiralpak IH (0.46 cm × 25 cm); 80 : 20 v/v, *n*-hexane / isopropanol; v = 1.2 mL·min^{-1}, λ = 254 nm, t_R (major) = 25.71 min, t_R (minor) = 21.17 min]. – ^{1}H NMR (500 MHz, $CDCl_3$) δ 8.05 (d, *J* = 8.0 Hz, 2H), 7.96 (d, *J* = 8.1 Hz, 2H), 7.81 (t, *J* = 7.5 Hz, 1H), 7.64 (t, *J* = 7.8 Hz, 2H), 7.35 (d, *J* = 8.0 Hz, 2H), 2.45 (s, 3H) ppm. – ^{13}C NMR (126 MHz, $CDCl_3$) δ 144.4, 138.8, 136.2, 133.1 (d, *J* = 20.3 Hz), 129.8 (d, *J* = 14.0 Hz), 128.0, 127.1, 21.7 ppm. – ^{19}F NMR (471 MHz, $CDCl_3$) δ 73.69 ppm. – IR: 2969, 1738, 1598, 1450, 1343, 1187, 1166, 1142, 1089, 816, 751 cm^{-1}. – HRMS: calcd for $C_{13}H_{12}FNO_3S_2$: 336.0135, found 336.0142 [M+Na$^+$].

(*R*)-*N*'-Tosylbenzenesulfonimidoyl azide (5)

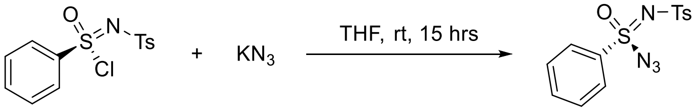

According to GP4, the reaction was carried out with **1a** (33.0 mg, 0.1 mmol), potassium azide (12.0 mg, 0.15 mmol) in anhydrous tetrahydrofuran (1 mL). After the completion of the reaction, for work-up, a saturated solution of brine (1.5 mL) was added, followed by ethyl acetate (5 mL). The organic phases were combined, dried over anhydrous Na_2SO_4, concentrated under reduced pressure, and the remaining material was purified by preparative TLC (hexane/ethyl acetate, 80 : 20 – 60 : 40) to give enantioenriched azide product **5** (30.0 mg, 96%).

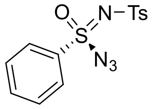

$[\alpha]_D$ = +23 (c 0.4, $CHCl_3$). – [Daicel Chiralpak IH (0.46 cm × 25 cm); 80 : 20 v/v, *n*-hexane / isopropanol; v = 1.2 mL·min^{-1}, λ = 254 nm, t_R (major) = 18.37 min, t_R (minor) = 23.72 min]. – ^{1}H NMR (500 MHz, $CDCl_3$) δ 8.02 (d, *J* = 8.5 Hz, 2H), 7.96 (d, *J* = 8.2 Hz, 2H), 7.76 (t, *J* = 7.5 Hz, 1H), 7.63 (t, *J* = 7.8 Hz, 2H), 7.34 (d, *J* = 8.1 Hz, 2H), 2.45 (s, 3H) ppm. – ^{13}C NMR (126 MHz, $CDCl_3$) δ 143.9, 139.3, 137.0, 135.3, 129.8, 129.6, 127.4, 127.0, 21.6 ppm. – IR: 2969, 2132, 1738, 1448, 1331, 1286, 1158, 1110, 1088, 1070, 997, 734 cm^{-1}. – HRMS: calcd for $C_{13}H_{12}N_4O_3S_2$: 337.0424, found 337.0434 [M+H$^+$].

Phenyl (*S*)-*N*-tosylbenzenesulfonimidate (6)

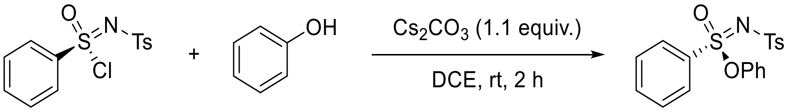

According to GP4, the reaction was carried out with **1a** (97% ee) (33.0 mg, 0.1 mmol), phenol (10.3 mg, 0.11 mmol) and cesium carbonate (35.8 mg, 0.11 mmol) in anhydrous 1,2-Dichloroethane (1 mL). After the completion of the reaction, for work-up, a saturated solution of brine (1.5 mL) was added, followed by dichloromethane (5 mL). The organic phases were combined, dried over anhydrous Na_2SO_4, concentrated under reduced pressure, and the remaining material was purified by preparative TLC (hexane/ethyl acetate, 80 : 20 – 60 : 40) to give enantioenriched sulfonimidate product **6** (29.0 mg, 75%, 96% ee).

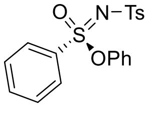

$[\alpha]_D$ = +6.5 (c 0.4, $CHCl_3$). – [Daicel Chiralpak IF (0.46 cm × 25 cm); 70 : 30 v/v, *n*-hexane / isopropanol; v = 1.2 mL·min^{-1}, λ = 254 nm, t_R (major) = 15.28 min, t_R (minor) = 16.47 min]. – ^{1}H NMR (500 MHz, $CDCl_3$) δ 7.92 (dd, *J* = 19.8, 7.9 Hz, 4H), 7.71 (t, *J* = 7.5 Hz, 1H), 7.55 (t, *J* = 7.9 Hz, 2H), 7.32–7.24 (m, 5H),

7.09–6.98 (m, 2H), 2.42 (s, 3H) ppm. – ^{13}C NMR (126 MHz, $CDCl_3$) δ 149.1, 143.3, 140.0, 135.3, 134.9, 129.8, 129.3, 129.3, 128.3, 127.7, 126.9, 122.7, 115.3, 21.6 ppm. – IR: 2969, 1738, 1596, 1486, 1448, 1328, 1286, 1156, 1115, 1080, 1022, 855, 740 cm^{-1}. – HRMS: calcd for $C_{19}H_{17}NO_4S_2$: 388.0672, found 388.0684 [M+H$^+$].

(*R*)-4-Methyl-*N*-(oxo(phenyl)(piperidin-1-yl)-λ^6-sulfaneylidene)benzenesulfonamide (7)

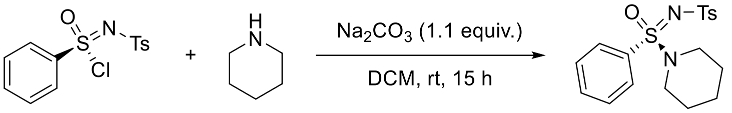

According to GP4, the reaction was carried out with **1a** (97% ee) (33.0 mg, 0.1 mmol), piperidine (9.3 mg, 0.11 mmol) and sodium carbonate (11.66 mg, 0.11 mmol) in anhydrous dichloromethane (1 mL) at rt. After the completion of the reaction, for work-up, a saturated solution of brine (1.5 mL) was added, followed by dichloromethane (5 mL). The organic phases were combined, dried over anhydrous Na_2SO_4, concentrated under reduced pressure, and the remaining material was purified by preparative TLC (hexane/ethyl acetate, 80 : 20 – 60 : 40) to give enantioenriched sulfonimidate product **7** (28.0 mg, 75%, 96% ee).

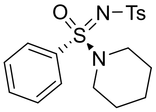

$[\alpha]_D$ = +93 (c 0.7, $CHCl_3$). – [Daicel Chiralpak IBN (0.46 cm × 25 cm); 90 : 10 v/v, *n*-hexane / isopropanol; v = 1.2 mL·min^{-1}, λ = 254 nm, t_R (major) = 21.85 min, t_R (minor) = 19.15 min]. – ^{1}H NMR (500 MHz, $CDCl_3$) δ 7.89 (dd, *J* = 14.3, 7.7 Hz, 4H), 7.63 (t, *J* = 7.5 Hz, 1H), 7.54 (t, *J* = 7.8 Hz, 2H), 7.28 (d, *J* = 8.6 Hz, 2H), 3.23 (ddd, *J* = 11.5, 7.1, 3.9 Hz, 2H), 3.10 (ddd, *J* = 11.5, 7.0, 4.0 Hz, 2H), 2.41 (s, 3H), 1.79–1.56 (m, 5H), 1.49 (p, *J* = 5.9 Hz, 2H) ppm. – ^{13}C NMR (126 MHz, $CDCl_3$) δ 142.7, 140.9, 136.1, 133.4, 129.2, 129.1, 127.6, 126.8, 47.1, 25.0, 23.4, 21.5 ppm. – IR: 2940, 2852, 1738, 1598, 1446, 1315, 1258, 1154, 1109, 1087, 1022, 925, 815, 736 cm^{-1}. – HRMS: calcd for $C_{18}H_{22}N_2O_3S_2$: 379.1145, found 379.1144 [M+H$^+$].

(*S*)-*N*-((4-(8-Chloro-5,6-dihydro-11*H*-benzo[5,6]cyclohepta[1,2-b]pyridin-11-ylidene)piperidin-1-yl)(oxo)(phenyl)-λ^6-sulfaneylidene)-4-methylbenzenesulfonamide (8)

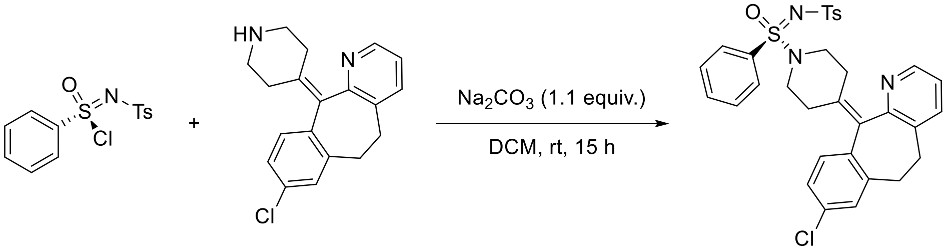

According to GP4, the reaction was carried out with *ent*-**1a** (96% ee) (33.0 mg, 0.1 mmol), 8-chloro-11-(piperidin-4-ylidene)-6,11-dihydro-5*H*-benzo[5,6]cyclohepta[1,2-b]pyridine (34.0 mg, 0.11 mmol) and sodium carbonate (11.66 mg, 0.11 mmol) in anhydrous dichloromethane (1 mL). After the completion of the reaction, for work-up, a saturated solution of brine (1.5 mL) was added, followed by dichloromethane (5 mL). The organic phases were combined, dried over anhydrous Na_2SO_4, concentrated under reduced pressure, and the remaining material was purified by preparative TLC (hexane/ethyl acetate, 80 : 20 – 60 : 40) to give enantioenriched sulfonimidate product **8** (39.0 mg, 65%, 94% ee).

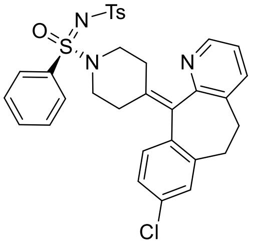

$[\alpha]_D$ = +40.0 (c 0.5, $CHCl_3$). – [Daicel Chiralpak IBN (0.46 cm × 25 cm); 85 : 15 v/v, *n*-hexane / isopropanol; v = 1.2 mL·min^{-1}, λ = 210 nm, t_R (major) = 46.78 min, t_R (minor) = 33.65 min]. – ^{1}H NMR (500 MHz, $CDCl_3$) δ 8.37 (t, *J* = 4.8 Hz, 1H), 7.89 (t, *J* = 7.1 Hz, 4H), 7.64 (t, *J* = 7.5 Hz, 1H), 7.54 (t, *J* = 6.9 Hz, 2H), 7.43 (d, *J* = 7.6 Hz, 1H), 7.27 (dd, *J* = 9.1, 3.3 Hz, 2H), 7.19–7.07 (m, 3H), 7.07 – 6.98 (m, 1H), 3.49 (ddd, *J* = 18.4, 9.1, 5.4 Hz, 1H), 3.39 – 3.06 (m, 5H), 2.89 – 2.73 (m, 2H), 2.71 – 2.48 (m, 2H), 2.47 – 2.36 (m, 5H) ppm. – ^{13}C NMR (126 MHz, $CDCl_3$) δ 156.5 (d, *J* = 8.5 Hz), 146.7 (d, *J* = 2.4 Hz), 142.8 (d, *J* = 4.1 Hz), 140.8 (d, *J* = 2.7 Hz), 139.6 (d, *J* = 3.5 Hz), 137.7 (d, *J* = 5.9 Hz), 137.3 (d, *J* = 8.7 Hz), 136.2 (d, *J* = 3.7 Hz), 135.3 (d, *J* = 3.7 Hz), 133.6, 133.4, 133.2, 130.3 (d, *J* = 11.2 Hz), 129.3 (d, *J* = 10.1 Hz), 129.1 (d, *J* = 5.4 Hz), 127.5 (d, *J* = 5.9 Hz), 126.8 (d, *J* = 4.0 Hz), 126.2, 122.5, 47.6, 47.2 (d,

J = 3.6 Hz), 31.6 (d, J = 6.7 Hz), 31.4 (d, J = 6.8 Hz), 30.0 (d, J = 26.8 Hz), 29.8 (d, J = 25.8 Hz), 21.6 ppm.– IR: 2922, 2853, 1738, 1592, 1445, 1370, 1315, 1262, 1153, 1108, 1086, 996, 928, 815, 734 cm^{-1}. – HRMS: calcd for $C_{32}H_{30}ClN_3O_3S_2$: 604.1490, found 603.1489 [M+H$^+$].

(*S*)-*N*-((4-(6-Fluorobenzo[d]isoxazol-3-yl)piperidin-1-yl)(oxo)(phenyl)- λ^6-sulfaneylidene)-4-methylbenzenesulfonamide (9)

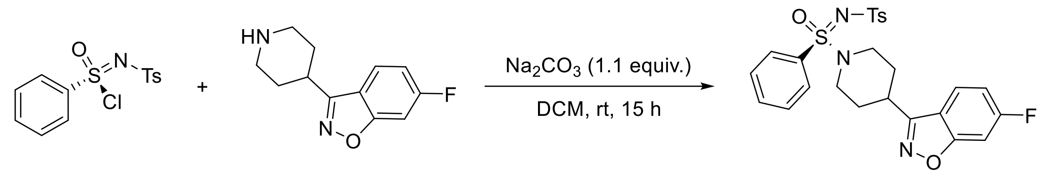

According to GP4, the reaction was carried out with *ent*-**1a** (96% ee) (33.0 mg, 0.1 mmol), 6-fluoro-3-(piperidin-4-yl)benzo[*d*]isoxazole (24.2 mg, 0.11 mmol) and sodium carbonate (11.66 mg, 0.11 mmol) in anhydrous dichloromethane (1 mL). After the completion of the reaction, for work-up, a saturated solution of brine (1.5 mL) was added, followed by dichloromethane (5 mL). The organic phases were combined, dried over anhydrous Na_2SO_4, concentrated under reduced pressure, and the remaining material was purified by preparative TLC (hexane/ethyl acetate, 80 : 20 – 60 : 40) to give enantioenriched sulfonimidate product **9** (27.7 mg, 54%, 96% ee).

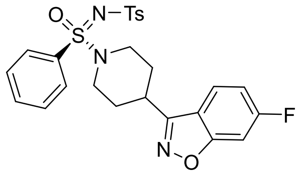

$[\alpha]_D$ = +45.0 (c 0.4, $CHCl_3$). – [Daicel Chiralpak IBN (0.46 cm × 25 cm); 50 : 50 v/v, *n*-hexane / isopropanol; v = 1.2 mL·min^{-1}, λ = 254 nm, t_R (major) = 8.94 min, t_R (minor) = 11.29 min]. – ^{1}H NMR (500 MHz, $CDCl_3$) δ 7.92 (t, J = 7.1 Hz, 4H), 7.74–7.48 (m, 4H), 7.27 (dd, J = 22.8, 9.4 Hz, 3H), 7.08 (t, J = 8.9 Hz, 1H), 4.07 (d, J = 13.7 Hz, 1H), 3.82 (d, J = 12.2 Hz, 1H), 3.27–3.09 (m, 1H), 3.00–2.85 (m, 2H), 2.41 (s, 3H), 2.19–2.03 (m, 3H) ppm. – ^{13}C NMR (126 MHz, CDCl3) δ 165.2, 163.9 (d, J = 13.6 Hz), 163.2, 159.9, 142.9, 140.7, 136.2, 133.8, 129.3 (d, J = 11.5 Hz), 127.4, 126.8, 122.4 (d, J = 11.0 Hz), 116.9, 112.8 (d, J = 25.3 Hz), 97.5 (d, J = 26.8 Hz), 45.9 (d, J = 50.2 Hz), 33.3, 29.4 (d, J = 2.7 Hz), 21.6 ppm. – ^{19}F NMR (471 MHz, $CDCl_3$) δ –108.92 ppm. – IR: 3065, 2927, 2852, 1738, 1610, 1447, 1314, 1259, 1152, 1110, 1084, 1049, 1018, 998, 922, 847, 814, 733 cm^{-1}. – HRMS: calcd for $C_{25}H_{24}FN_3O_4S_2$: 514.1265, found 514.1264 [M+H$^+$].

Methyl (*R*)-2-((*tert*-butoxycarbonyl)amino)-3-(4-(((*R*)-*N*-tosylphenylsulfonimidoyl)oxy)phenyl)propanoate (10)

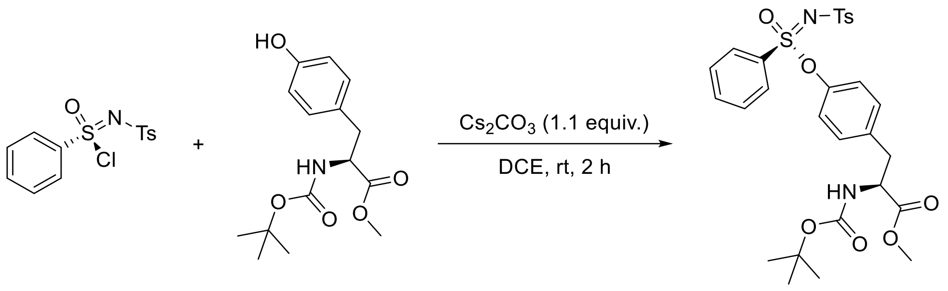

According to GP4, the reaction was carried out with *ent*-**1a** (97% ee) (33.0 mg, 0.1 mmol), methyl (*tert*-butoxycarbonyl)-L-tyrosinate (35.5 mg, 0.11 mmol) and cesium carbonate (35.8 mg, 0.11 mmol) in anhydrous 1,2-Dichloroethane (1 mL). After the completion of the reaction, for work-up, a saturated solution of brine (1.5 mL) was added, followed by dichloromethane (5 mL). The organic phases were combined, dried over anhydrous Na_2SO_4, concentrated under reduced pressure, and the remaining material was purified by preparative TLC (hexane/ethyl acetate, 80 : 20 – 60 : 40) to give enantioenriched sulfonimidate product **10** (36.0 mg, 61%, 97% de).

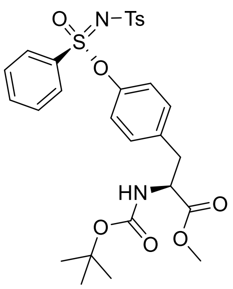

$[\alpha]_D$ = +46 (c 0.6, $CHCl_3$). – [Daicel Chiralpak IJ (0.46 cm × 25 cm); 60 : 40 v/v, *n*-hexane / isopropanol; v = 1.2 mL·min^{-1}, λ = 210 nm, t_R (major) = 19.97 min, t_R (minor) = 25.05 min]. – ^{1}H NMR (500 MHz, Chloroform-*d*) δ 7.90 (t, *J* = 6.8 Hz, 4H), 7.71 (t, *J* = 7.4 Hz, 1H), 7.55 (t, *J* = 7.7 Hz, 2H), 7.28 (d, *J* = 7.9 Hz, 2H), 7.06 (d, *J* = 8.2 Hz, 2H), 6.96 (d, *J* = 8.3 Hz, 2H), 4.99 (d, *J* = 7.6 Hz, 1H), 4.62–4.47 (m, 1H), 3.70 (s, 3H), 3.18–2.88 (m, 2H), 2.42 (s, 3H), 1.43 (s, 9H) ppm. – ^{13}C NMR (126 MHz, $CDCl_3$) δ 171.9, 155.0, 148.1, 143.3, 140.0, 136.1, 135.2, 134.9, 130.6, 129.7, 129.3, 128.3, 126.9, 126.5, 122.8, 80.1, 54.3, 52.3, 37.8, 28.3, 21.6 ppm. – IR: 3367, 2970, 1739, 1713, 1499, 1448, 1365, 1328, 1285, 1156, 1116, 1080, 1018, 861, 735 cm^{-1}. – HRMS: calcd for $C_{28}H_{32}N_2O_8S_2$: 589.1673, found 589.1672 [M+Na$^+$].

4-((2*S*,3*R*)-1-(4-fluorophenyl)-3-((*R*)-3-(4-fluorophenyl)-3-hydroxypropyl)-4-oxoazetidin-2-yl)phenyl (*S*)-N-tosylbenzenesulfonimidate (11)

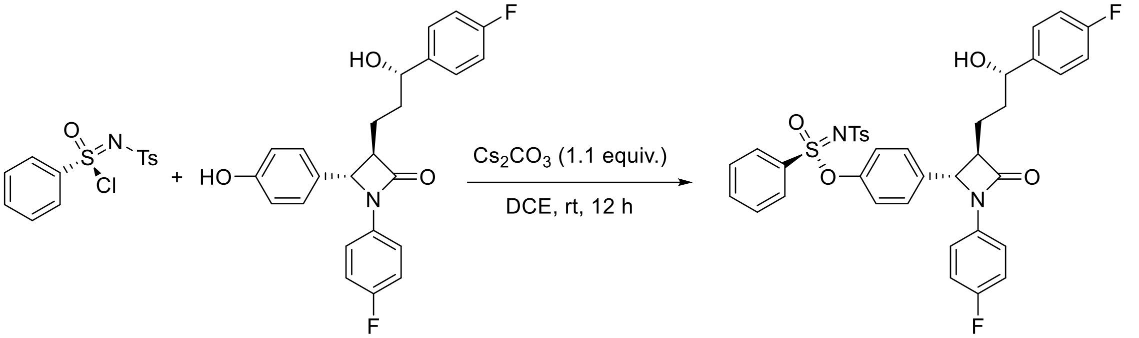

According to GP4, the reaction was carried out with sulfonimidoyl chloride *ent*-**1a** (96% ee) (33.0 mg, 0.1 mmol), (3*R*,4*S*)-1-(4-fluorophenyl)-3-((*S*)-3-(4-fluorophenyl)-3-hydroxypropyl)-4-(4-hydroxyphenyl)azetidin-2-one (45.0 mg, 0.11 mmol) and cesium carbonate (35.8 mg, 0.11 mmol) in anhydrous dichloroethane (1 mL). After the completion of the reaction, for work-up, a saturated solution of brine (1.5 mL) was added, followed by dichloromethane (5 mL). The organic phases were combined, dried over anhydrous Na_2SO_4, concentrated under reduced pressure, and the remaining material was purified by preparative TLC (hexane/ethyl acetate, 50 : 50) to give enantioenriched sulfonimidate product **11** as a dense liquid (52.5 mg, 75%, 96% de).

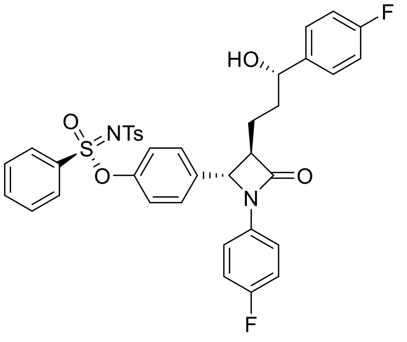

$[\alpha]_D$ = –9 (c 1.0, $CHCl_3$). – [Daicel Chiralpak IC (0.46 cm × 25 cm); 40 : 60 v/v, *n*-hexane / isopropanol; v = 1.2 mL·min^{-1}, λ = 210 nm, t_R (major) = 21.06 min, t_R (minor) = 24.05 min]. – ^{1}H NMR (500 MHz, $CDCl_3$) δ 7.89 (dd, *J* = 13.2, 7.9 Hz, 4H), 7.70 (t, *J* = 7.4 Hz, 1H), 7.53 (t, *J* = 7.9 Hz, 2H), 7.35 – 7.22 (m, 7H), 7.18 (dd, *J* = 9.0, 4.6 Hz, 2H), 7.09 – 6.98 (m, 4H), 6.94 (t, *J* = 8.7 Hz, 1H), 4.72 (t, *J* = 6.0 Hz, 1H), 4.64 (d, *J* = 2.3 Hz, 1H), 3.04 (td, *J* = 7.5, 2.4 Hz, 1H), 2.41 (s, 3H), 2.06 – 1.88 (m, 4H) ppm. – ^{13}C NMR (126 MHz, $CDCl_3$) δ 167.1,162.2 (d, *J* = 245.5 Hz), 159.1 (d, *J* = 243.9 Hz), 149.1, 143.6, 140.1 (d, *J* = 3.2 Hz), 139.8, 137.4, 135.1, 133.6, 133.5, 129.4, 128.2, 127.4 (d, *J* = 8.2 Hz), 127.3, 126.9, 123.6, 118.4 (d, *J* = 7.8 Hz), 116.1, 115.9, 115.5, 115.3, 73.1, 60.5, 60.4, 36.6, 25.1, 21.6 ppm. – ^{19}F NMR (471 MHz, $CDCl_3$) δ -114.86 (d, *J* = 11.6 Hz), -117.54 (q, *J* = 7.3 Hz) ppm. – IR: 292, 2361, 2337, 1743, 1509, 1280, 1160, 754, 686, 537 cm^{-1}. – HRMS: calcd for $C_{37}H_{32}F_2N_2O_6S_2$: 725.1562, found 725.1558 [M+Na$^+$].

2,2-Dimethyl-3-(((*R*)-*N*-tosylphenylsulfonimidoyl)oxy)propyl (3^5*S*,9^1*R*,9^2*R*,5*S*)-5-(*tert*-butyl)-17-methoxy-4,7-dioxo-2,8-dioxa-6-aza-1(2,3)-quinoxalina-3(3,1)-pyrrolidina-9(1,2)-cyclopropanacyclotetradecaphane-3^5-carboxylate (12)

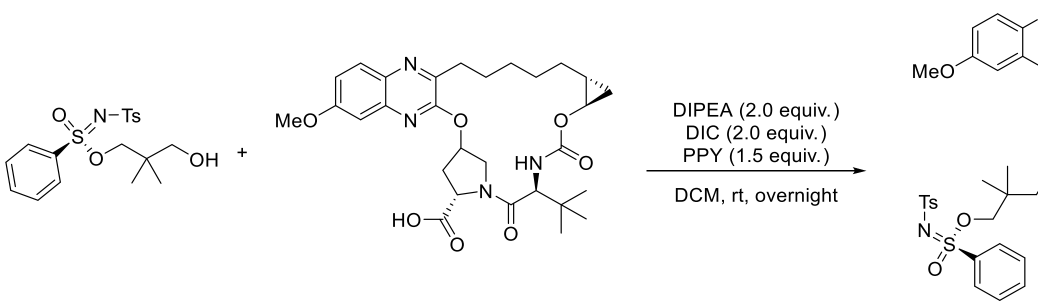

According to GP5, the reaction was carried out with (3^5*S*,9^1*R*,9^2*R*,5*S*)-5-(*tert*-butyl)-17-methoxy-4,7-dioxo-2,8-dioxa-6-aza-1(2,3)-quinoxalina-3(3,1)-pyrrolidina-9(1,2)-cyclopropanacyclotetradecaphane-3^5-carboxylic acid (67 mg, 0.15 mmol), *N*,*N*-diisopropylethylamine (DIPEA) (24 mg, 0.20 mmol), *N*,*N*'-diisopropylcarbodiimide (DIC) (26 mg, 0.20 mmol), 4-pyrrolidinopyridine (PPY) (1.5 mg, 0.01 mmol), and sulfonimidate **3a** (97% ee, 40 mg, 0.1 mmol) in anhydrous dichloromethane (1 mL). After the completion of the reaction, for work-up, a saturated solution of brine (1.5 mL) was added, followed by dichloromethane (5 mL). The organic phases were combined, dried over anhydrous Na_2SO_4, concentrated under reduced pressure, and the remaining material was purified by preparative TLC (hexane/ethyl acetate, 80 : 20 – 60 : 40) to give the enantioenriched product **12** (52 mg, 56%, 97% ee, 100% es).

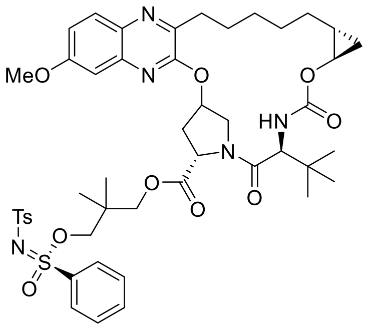

$[\alpha]_D$ = –21.6 (c 0.18, $CHCl_3$). – [Daicel Chiralpak IBN (0.46 cm × 25 cm); 80 : 20 v/v, *n*-hexane / isopropanol; v = 1.2 mL·min^{-1}, λ = 254 nm, t_R (major) = 9.11 min, t_R (minor) = 8.07 min]. – ^{1}H NMR (500 MHz, $CDCl_3$) δ 7.91 (d, *J* = 7.9 Hz, 2H), 7.85 (d, *J* = 9.0 Hz, 1H), 7.79 (d, *J* = 8.2 Hz, 2H), 7.54 (t, *J* = 7.4 Hz, 1H), 7.46 (t, *J* = 7.8 Hz, 2H), 7.24–7.13 (m, 4H), 5.99 (t, *J* = 3.9 Hz, 1H), 5.27 (d, *J* = 9.9 Hz, 1H), 4.56–4.39 (m, 3H), 4.12 (d, *J* = 9.2 Hz, 1H), 4.06–3.98 (m, 2H), 3.95–3.88 (m, 5H), 3.82 (dt, *J* = 6.5, 2.9 Hz, 1H), 2.95 (td, *J* = 13.1, 4.5 Hz, 1H), 2.81 (td, *J* = 13.0, 4.8 Hz, 1H), 2.68 (dd, *J* = 14.4, 7.2 Hz, 1H), 2.37 (s, 3H), 2.28 (ddd, *J* = 14.6, 10.8, 4.2 Hz, 1H), 1.89–1.46 (m, 11H), 1.16 (d, *J* = 6.4 Hz, 1H), 1.09 (s, 9H), 0.98 (s, 4H), 0.94 (s, 4H), 0.76–0.65 (m, 1H), 0.49 (q, *J* = 6.4 Hz, 1H) ppm. – ^{13}C NMR (126 MHz, $CDCl_3$) δ 171.7, 171.2, 160.3, 157.3, 154.9, 148.4, 143.1, 140.9, 140.0, 135.6, 134.4, 129.3, 129.2, 129.2, 127.8, 126.8, 118.6, 105.9, 75.8, 74.3, 68.6, 59.2, 57.9, 55.7, 55.3, 54.3, 35.6, 35.5, 35.0, 33.9, 30.7, 29.4, 28.4, 28.1, 26.3, 23.5, 21.5, 21.3, 21.2, 18.7, 11.0 ppm. – IR: 2968, 1718, 1648, 1620, 1505, 1434, 1264, 1160, 1090, 936, 730 cm^{-1}. – HRMS: calcd for $C_{47}H_{59}N_5O_{11}S_2$: 934.3725, found 934.3721 [M+H$^+$].

(*R*)-2-(2,2-dimethyl-3-((*N*-tosylphenylsulfonimidoyl)oxy)propoxy)-2-oxoethyl 2-(1-(4-chlorobenzoyl)-5-methoxy-2-methyl-1*H*-indol-3-yl)acetate (13)

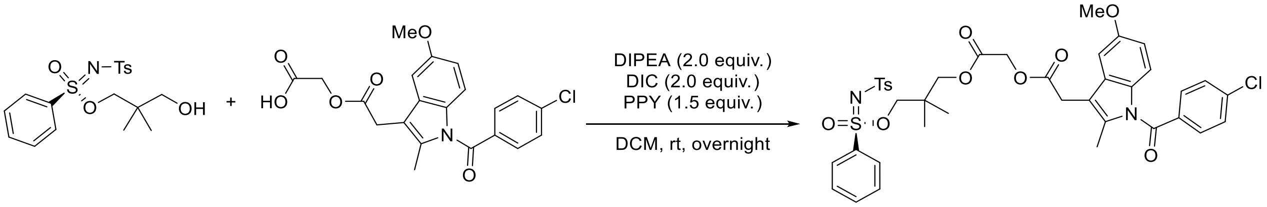

According to GP5, the reaction was carried out with 2-(2-(1-(4-chlorobenzoyl)-5-methoxy-2-methyl-1*H*-indol-3-yl)acetoxy)acetic acid (50 mg, 0.15 mmol), *N*,*N*-diisopropylethylamine (DIPEA) (24 mg, 0.20 mmol), *N*,*N*'-diisopropylcarbodiimide (DIC) (26 mg, 0.20 mmol), 4-pyrrolidinopyridine (PPY) (1.5 mg, 0.01 mmol), and sulfonimidate **3a** (97% ee, 40 mg, 0.1 mmol) in anhydrous dichloromethane (1 mL). After the completion of the reaction, for work-up, a saturated solution of brine (1.5 mL) was added, followed by dichloromethane (5 mL). The organic phases were combined, dried over anhydrous Na_2SO_4, concentrated under reduced pressure, and the

remaining material was purified by preparative TLC (hexane/ethyl acetate, 80 : 20 – 60 : 40) to give enantioenriched product **13** (51.6 mg, 65%, 96% ee, 99% es).

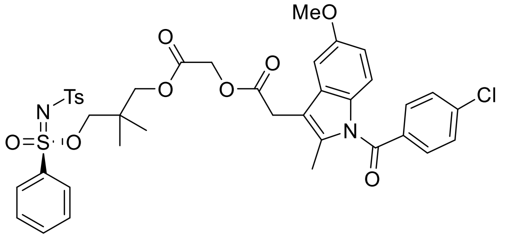

[α]$_D$ = +2.5 (c 0.18, $CHCl_3$). – [Daicel Chiralpak IBN (0.46 cm × 25 cm); 50 : 50 v/v, *n*-hexane / isopropanol; v = 1.2 mL·min^{-1}, λ = 254 nm, t_R (major) = 9.36 min, t_R (minor) = 8.39 min]. – ^{1}H NMR (500 MHz, $CDCl_3$) δ 7.92 (d, *J* = 7.3 Hz, 2H), 7.85 (d, *J* = 8.4 Hz, 2H), 7.71–7.62 (m, 3H), 7.59–7.45 (m, 4H), 7.25 (d, *J* = 8.2 Hz, 2H), 7.02 (d, *J* = 2.6 Hz, 1H), 6.91 (d, *J* = 9.0 Hz, 1H), 6.70 (dd, *J* = 9.0, 2.6 Hz, 1H), 4.61 (s, 2H), 4.02 (q, *J* = 9.2 Hz, 2H), 3.92 (s, 2H), 3.86 (s, 3H), 3.81 (s, 2H), 2.40 (d, *J* = 5.5 Hz, 6H), 0.94 (d, *J* = 2.1 Hz, 6H) ppm. – ^{13}C NMR (126 MHz, $CDCl_3$) δ 170.3, 168.3, 167.3, 156.1, 143.2, 140.0, 139.3, 136.1, 135.5, 134.6, 133.9, 131.2, 130.8, 130.6, 129.4, 129.3, 129.2, 128.4, 127.7, 126.8, 114.9, 111.9, 111.8, 101.3, 75.4, 68.8, 60.9, 55.7, 35.2, 29.8, 23.5, 21.5, 21.3 13.4 ppm. – IR: 2968, 1748, 1679, 1476, 1358, 1264, 1224, 1156, 1088, 1014, 925, 734 cm^{-1}. – HRMS: calcd for $C_{39}H_{39}ClN_2O_{10}S_2$: 833.1366, found 833.1362 [M+K$^+$].

2,2-Dimethyl-3-(((*R*)-*N*-tosylphenylsulfonimidoyl)oxy)propyl 6-(3-((3r,5r,7r)-adamantan-1-yl)-4-methoxyphenyl)-2-naphthoate (14)

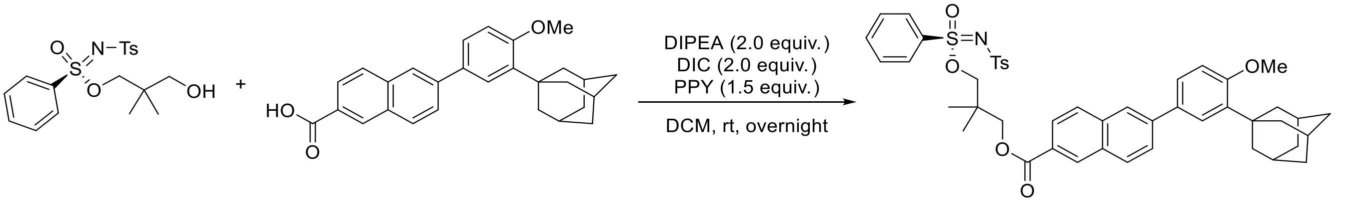

According to GP5, the reaction was carried out with adapalene (49.5 mg, 0.15 mmol), *N*,*N*-diisopropylethylamine (DIPEA) (24 mg, 0.20 mmol), *N*,*N*'-diisopropylcarbodiimide (DIC) (26 mg, 0.20 mmol), 4-pyrrolidinopyridine (PPY) (1.5 mg, 0.01 mmol), and sulfonimidate **3a** (97% ee, 40 mg, 0.1 mmol) in anhydrous dichloromethane (1 mL). After the completion of the reaction, for work-up, a saturated solution of brine (1.5 mL) was added, followed by dichloromethane (5 mL). The organic phases were combined, dried over anhydrous Na_2SO_4, concentrated under reduced pressure, and the remaining material was purified by preparative TLC (hexane/ethyl acetate, 80 : 20 – 60 : 40) to give enantioenriched product **14** (61.8 mg, 78%, 96% ee, 99% es).

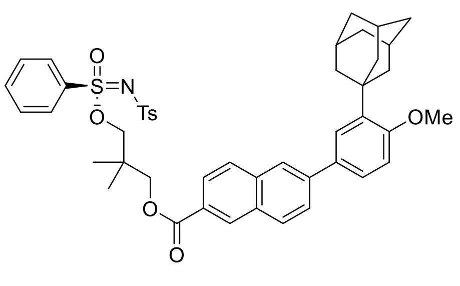

[α]$_D$ = –1.2 (c 0.35, $CHCl_3$). – [Daicel Chiralpak IBN (0.46 cm × 25 cm); 90 : 10 v/v, *n*-hexane / isopropanol; v = 1.2 mL·min^{-1}, λ = 254 nm, t_R (major) = 24.55 min, t_R (minor) = 20.9 min]. – ^{1}H NMR (500 MHz, $CDCl_3$) δ 8.47 (s, 1H), 8.08–7.97 (m, 2H), 7.97–7.80 (m, 7H), 7.68–7.55 (m, 2H), 7.49–7.36 (m, 3H), 7.23 (d, *J* = 8.2 Hz, 2H), 7.04 (d, *J* = 8.4 Hz, 1H), 4.26–4.16 (m, 2H), 4.10 (s, 2H), 3.94 (s, 3H), 2.38 (s, 3H), 2.22 (s, 7H), 2.14 (s, 3H), 1.84 (s, 7H), 1.10 (d, *J* = 7.0 Hz, 6H) ppm. – ^{13}C NMR (126 MHz, $CDCl_3$) δ 166.2, 159.0, 143.2, 141.6, 140.1, 139.1, 136.0, 135.4, 134.5, 132.5, 131.2, 130.9, 129.8, 129.3, 128.3, 127.7, 126.9, 126.6, 126.5, 125.9, 125.8, 125.4, 124.7, 112.2, 76.1, 68.7, 55.2, 40.6, 37.3, 37.1, 35.5, 29.1, 21.6, 21.5 ppm. – IR: 2903, 1717, 1474, 1237, 1216, 1158, 1119, 1090, 1024, 940, 810, 733 cm^{-1}. – HRMS: calcd for $C_{46}H_{49}NNaO_7S_2$: 814.2848, found 814.2836 [M+Na$^+$].

(*R*)-2,2-dimethyl-3-((*N*-tosylphenylsulfonimidoyl)oxy)propyl 2-(3-cyano-4-isobutoxyphenyl)-4-methylthiazole-5-carboxylate (15)

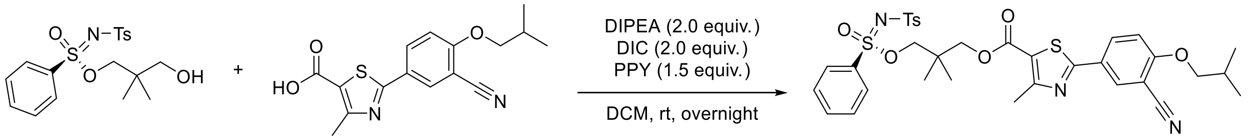

According to GP5, the reaction was carried out with 2-(3-cyano-4-isobutoxyphenyl)-4-methylthiazole-5-carboxylic acid (38 mg, 0.15 mmol), *N*,*N*-diisopropylethylamine (DIPEA) (24 mg, 0.20 mmol), *N*,*N*'-diisopropylcarbodiimide (DIC) (26 mg, 0.20 mmol), 4-pyrrolidinopyridine (PPY) (1.5 mg, 0.01 mmol), and sulfonimidate **3a** (97% ee, 40 mg, 0.1 mmol) in anhydrous dichloromethane (1 mL). After the completion of the reaction, for work-up, a saturated solution of brine (1.5 mL) was added, followed by dichloromethane (5 mL). The organic phases were combined, dried over anhydrous Na_2SO_4, concentrated under reduced pressure, and the remaining material was purified by preparative TLC (hexane/ethyl acetate, 80 : 20 – 60 : 40) to give enantioenriched product **15** (29.2 mg, 42%, 96% ee, 99% es).

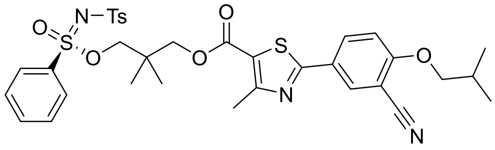

$[\alpha]_D$ = +1.4 (c 0.25, $CHCl_3$). – [Daicel Chiralpak IBN (0.46 cm × 25 cm); 50 : 50 v/v, *n*-hexane / isopropanol; v = 1.2 mL·min^{-1}, λ = 254 nm, t_R (major) = 6.0 min, t_R (minor) = 5.47 min]. – ^{1}H NMR (500 MHz, $CDCl_3$) δ 8.19 (d, *J* = 2.3 Hz, 1H), 8.12 (dd, *J* = 8.9, 2.4 Hz, 1H), 7.93 (d, *J* = 7.8 Hz, 2H), 7.85 (d, *J* = 8.4 Hz, 2H), 7.58 (t, *J* = 7.5 Hz, 1H), 7.48 (t, *J* = 7.9 Hz, 2H), 7.24 (d, *J* = 8.2 Hz, 2H), 7.05 (d, *J* = 9.0 Hz, 1H), 4.11 (d, *J* = 4.6 Hz, 2H), 4.02 (s, 2H), 3.93 (d, *J* = 6.6 Hz, 2H), 2.72 (s, 3H), 2.39 (s, 3H), 2.23 (hept, *J* = 6.6 Hz, 1H), 1.11 (d, *J* = 6.7 Hz, 6H), 1.04 (s, 6H) ppm. – ^{13}C NMR (126 MHz, $CDCl_3$) δ 167.5, 162.6, 161.4, 161.4, 143.2, 140.0, 135.5, 134.5, 132.7, 132.1, 129.3, 127.7, 126.8, 125.8, 121.2, 115.4, 112.7, 103.0, 75.8, 75.7, 68.9, 35.3, 28.2, 21.5, 21.5, 18.9, 17.5 ppm. – IR: 2970, 1715, 1606, 1507, 1449, 1431, 1375, 1326, 1263, 1158, 1118, 1089, 1013, 943, 815, 731 cm^{-1}. – HRMS: calcd for $C_{34}H_{37}N_3O_7S_3$: 696.1866, found 696.1864 [M+H$^+$].

(*R*)-*N*-(Butyl(oxo)(phenyl)-λ^6-sulfaneylidene)-4-methylbenzenesulfonamide (16)

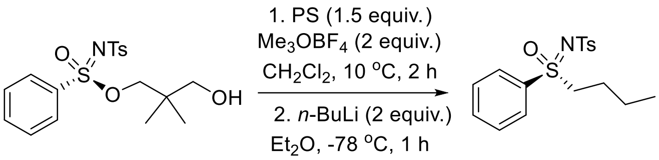

According to GP6, the reaction was conducted with sulfonimidate *ent*-**1a** (91% ee, 119 mg, 0.3 mmol), proton sponge (PS) (96 mg, 0.45 mmol, 1.5 equiv.), trimethyloxonium tetrafluoroborate (89 mg, 0.6 mmol, 2 equiv.), and dichloromethane (3 mL), and the reaction mixture was stirred at 10 °C for the 2 h. After the completion of reaction (monitored by thin layer chromatography), the reaction mixture was filtered through small pad of celite, and filtrate was concentrated under reduced pressure. The crude product was purified by column chromatography on silica gel with a mixture of hexane and ethyl acetate (7 : 3) as an eluent to give the desired methylated sulfonimidate *ent*-**3a-Me** (115 mg, 93%) as a colorless liquid.

According to GP7, the reaction was conducted with sulfonimidate *ent*-**3a-Me** (41 mg, 0.1 mmol) and diethyl ether (1 mL), and the reaction mixture was cooled to –78 °C and then *n*-BuLi (0.8 mL, 0.2 mmol, 2 equiv.) were slowly added. After the completion of reaction (monitored by thin layer chromatography), the reaction mixture was quenched with mixture of EDTA and ammonium hydroxide (pH = 9) and the organic layer was separated, and the aqueous layer was extracted with ethyl acetate (3 × 2 mL). The combined organic layers were dried over sodium sulfate and concentrated under reduced pressure. The crude mixture was purified by preparative TLC to with a mixture of hexane and ethyl acetate (7 : 3) as an eluent to give sulfoximine **16** (30 mg, 85%, 91% ee) as a colorless liquid.

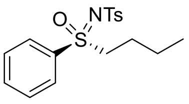

$[\alpha]_D$ = –120 (*c* 0.4, $CHCl_3$). – Daicel Chiralpak IH (0.46 cm × 25 cm); 50 : 50 v/v, n-hexane / isopropanol; v = 1.0 mL·min-1, λ = 254 nm, t_R (major) = 13.05 min., t_R (minor) = 11.2 min. – ^{1}H NMR (500 MHz, $CDCl_3$): 8.02 – 7.95 (2 H, m), 7.86 (2 H, d, *J* = 8.1 Hz), 7.71 (1 H, t, *J* = 7.4 Hz), 7.61 (2 H, t, *J* = 7.7 Hz), 7.26 (2 H, d, *J* = 8.0 Hz), 3.50 (2 H, dddd, *J* = 39.4, 14.1, 10.3, 5.8 Hz), 2.41 (3 H, s), 1.71 – 1.57 (2 H, m), 1.36

Go to table of contents

(2 H, h, *J* = 7.4 Hz), 0.88 (3 H, t, *J* = 7.3 Hz) ppm. – ^{13}C NMR (125 MHz, $CDCl_3$): 142.8, 141.0, 136.7, 134.4, 129.7, 129.3, 128.4, 126.8, 58.0, 24.6, 21.6, 21.3, 13.5 ppm. – IR: 2926, 2361-2338, 1451, 1309, 1149, 1086, 811, 749 cm^{-1}. – HRMS: calcd for $C_{17}H_{21}NO_3S_2$: 374.0855, found 374.0847 [M+Na$^+$].

(*R*)-4-methyl-*N*-(methyl(oxo)(phenyl)- λ^6--sulfaneylidene)benzenesulfonamide (17)

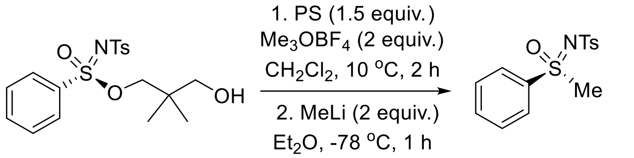

According to GP7, the reaction was conducted with sulfonimidate **3a-Me** (41 mg, 0.1 mmol) and diethyl ether (1 mL), and the reaction mixture was cooled to –78 °C and then MeLi (1.25 mL, 0.2 mmol, 2 equiv.) were slowly added. After the completion of reaction (monitored by thin layer chromatography), the reaction mixture was quenched with mixture of EDTA and ammonium hydroxide (pH = 9) and the organic layer was separated, and the aqueous layer was extracted with ethyl acetate (3 × 2 mL). The combined organic layers were dried over sodium sulfate and concentrated under reduced pressure. The crude mixture was purified by preparative TLC with a mixture of hexane and ethyl acetate (7 : 3) as an eluent to give sulfoximine **17** (19 mg, 62%, 92% ee) as a colorless liquid.

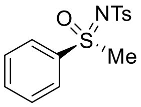

[α]$_D$ = –9 (*c* 0.2, $CHCl_3$). – Daicel Chiralpak IH (0.46 cm × 25 cm); 50 : 50 v/v, n-hexane / isopropanol; v = 1.2 mL·min-1, λ = 254 nm, , t$_R$ (major) = 13.7 min, t$_R$ (minor) = 15.5 min. – ^{1}H NMR (500 MHz, $CDCl_3$): 8.02 (2 H, dd, *J* = 7.8, 1.6 Hz), 7.90 – 7.82 (2 H, m), 7.70 (1 H, t, *J* = 7.5 Hz), 7.61 (2 H, t, *J* = 7.8 Hz), 7.26 (2 H, d, *J* = 8.3 Hz), 3.43 (3 H, s), 2.40 (3 H, s) ppm. – ^{13}C NMR (125 MHz, $CDCl_3$): 143.1, 140.8, 138.6, 134.5, 129.9, 129.4, 127.7, 126.8, 46.8, 21.7 ppm. – IR: 2924, 1746, 1508, 1324, 1218, 1156, 1118, 1089, 756, 633 cm^{-1}.

(R)-4-methyl-*N*-(oxo(phenethyl)(phenyl)- λ^6-sulfaneylidene)benzenesulfonamide (18)

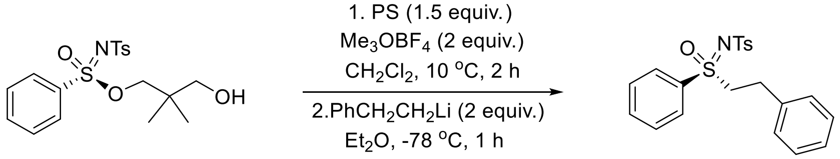

According to GP7, the reaction was conducted with (2-bromoethyl)benzene (37 mg, 0.2 mmol, 2 equiv.) and diethyl ether (1 mL), and the reaction mixture was cooled to –78 °C and then ^{t}BuLi (2.4 mL (1.7 M in pentane), 0.4 mmol, 4 equiv.) were slowly added. The reaction mixture was stirred for 1 h at same temperature and finally sulfonimidate *ent*-**3a-Me** (41 mg, 0.1 mmol, dissolved 0.5 mL diethyl ether) added slowly in 10 minutes. After the completion of reaction (monitored by thin layer chromatography), the reaction mixture was quenched with mixture of EDTA and ammonium hydroxide (pH = 9) and the organic layer was separated, and the aqueous layer was extracted with ethyl acetate (3 × 2 mL). The combined organic layers were dried over sodium sulfate and concentrated under reduced pressure. The crude mixture was purified by preparative TLC with a mixture of hexane and ethyl acetate (8 : 2) as an eluent to give sulfoximine **18** (29 mg, 73%, 92% ee) as a colorless liquid.

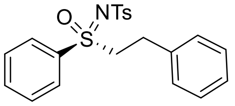

[α]$_D$ = –37 (*c* 0.13, $CHCl_3$). – Daicel Chiralpak IH (0.46 cm × 25 cm); 50 : 50 v/v, n-hexane / isopropanol; v = 1.0 mL·min-1, λ = 254 nm, , t$_R$ (major) = 14.99 min, t$_R$ (minor) = 19.40 min. – ^{1}H NMR (500 MHz, $CDCl_3$): 8.03 (2 H, dd, *J* = 7.6, 1.7 Hz), 7.89 (2 H, d, *J* = 8.3 Hz), 7.73 (1 H, d, *J* = 8.3 Hz), 7.65 – 7.59 (2 H, m), 7.30 – 7.19 (5 H, m), 7.13 – 7.03 (2 H, m), 3.87 – 3.66 (2 H, m), 3.11 – 2.93 (2 H, m), 2.42 (3 H, s) ppm. – ^{13}C NMR (125 MHz, $CDCl_3$): 143.0, 141.0, 136.8, 136.4, 134.6, 129.8, 129.4, 129.0, 128.5, 128.4, 127.3, 126.8, 59.3, 28.9, 21.7 ppm. – IR: 2922, 2339, 1665, 1580,1448, 1317, 1230, 1152, 1088, 1064, 815, 745, 685 cm^{-1}.

(*R*)-*N*-((4-Bromophenyl)(oxo)(phenyl)-λ^6-sulfaneylidene)-4-methylbenzenesulfonamide (19)

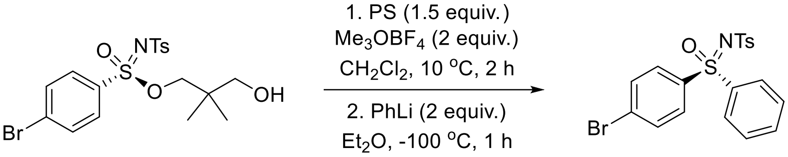

According to GP6, the reaction was conducted with sulfonimidate *ent*-**3d** (99% ee, 142.8 mg, 0.3 mmol), proton sponge (PS) (96 mg, 0.45 mmol, 1.5 equiv.), trimethyloxonium tetrafluoroborate (89 mg, 0.6 mmol, 2 equiv.), and dichloromethane (3 mL), and the reaction mixture was stirred at 10 °C for the 2 h. After the completion of reaction (monitored by thin layer chromatography), the reaction mixture was filtered through small pad of celite, and filtrate was concentrated under reduced pressure. The crude product was purified by column chromatography on silica gel with a mixture of hexane and ethyl acetate (7 : 3) as an eluent to give the desired methylated sulfonimidate *ent*-**3d-Me** (128 mg, 88%) as a colorless liquid.

According to GP7, the reaction was conducted with sulfonimidate *ent*-**3d-Me** (49 mg, 0.1 mmol) and diethyl ether (1 mL), and the reaction mixture was cooled to –78 °C and then PhLi (1.5 mL, 0.15 mmol, 1.5 equiv.) were slowly added. After the completion of reaction (monitored by thin layer chromatography), the reaction mixture was quenched with mixture of EDTA and ammonium hydroxide (pH = 9) and the organic layer was separated, and the aqueous layer was extracted with ethyl acetate (3 × 2 mL). The combined organic layers were dried over sodium sulfate and concentrated under reduced pressure. The crude mixture was purified by preparative TLC with a mixture of hexane and ethyl acetate (7 : 3) as an eluent to give sulfoximine **19** (25 mg, 56%, 97 % ee) as a colorless dense liquid.

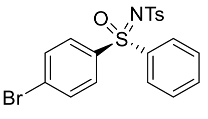

$[\alpha]_D$ = –20 (*c* 0.2, $CHCl_3$). – Daicel Chiralpak IBN (0.46 cm × 25 cm); 85 : 15 v/v, n-hexane / isopropanol; v = 1.2 mL·min-1, λ = 254 nm, t_R (major) = 20.16 min, t_R (minor) = 18.65 min. – ^1H NMR (500 MHz, $CDCl_3$): 8.04 – 7.98 (2 H, m), 7.86 (4 H, dd, *J* = 8.3, 4.8 Hz), 7.68 – 7.62 (3 H, m), 7.55 (2 H, t, *J* = 7.7 Hz), 7.26 (2 H, d, *J* = 8.1 Hz), 2.42 (3 H, s) ppm. – ^{13}C NMR (125 MHz, $CDCl_3$): 143.1, 140.8, 139.5, 139.2, 134.2, 133.0, 129.8, 129.4, 127.9, 126.9, 21.7 ppm. – IR: 2921, 2858, 2360, 1458, 1383, 1240, 1154, 1092, 1057, 749, 537 cm^{-1}. – HRMS: calcd for $C_{19}H_{16}BrNO_3S_2$: 471.9647, found 471.9648 [M+Na$^+$].

(S)-4-Methyl-*N*-(oxo(phenyl)(piperidin-1-yl)- λ^6-sulfaneylidene)benzenesulfonamide (*ent*-7)

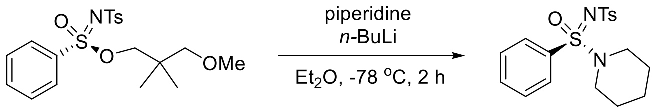

A solution of piperidine (8.5 mg, 0.10 mmol, 1.0 equiv.) in diethyl ether (1 mL) was cooled to –78 °C, and a 2.5M solution of *n*-butyllithium in hexane (0.044 mL 0.11 mmol, 1.1 equiv.) was added. The reaction mixture was stirred for 30 minutes at –78 °C, and a solution of sulfonimidate *ent*-**3a-Me** (41 mg, 0.1 mmol) in diethyl ether (0.5 mL) was added dropwise in 5 minutes. After the completion of reaction (monitored by thin layer chromatography), the reaction mixture was quenched with a mixture of a saturated aqueous solution of EDTA and aqueous ammonium hydroxide (pH = 9). The organic layer was separated, and the aqueous layer was extracted with ethyl acetate (3 × 2 mL). The combined organic layers were dried over sodium sulfate and concentrated under reduced pressure. The crude mixture was purified by column chromatography on silica gel with a mixture of hexane and ethyl acetate (7: 3) as an eluent to give sulfonimidate ***ent*-7** (19 mg, 50%, 90% ee, 100% es) as a colorless liquid.

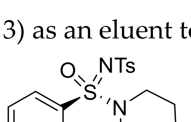

$[\alpha]_D$ = -40 (c 0.4, $CHCl_3$). – [Daicel Chiralpak IBN (0.46 cm × 25 cm); 90 : 10 v/v, *n*-hexane / isopropanol; v = 1.2 mL·min^{-1}, λ = 254 nm, t_R (major) = 15.66 min, t_R (minor) = 18.35 min]. – ^1H NMR (500 MHz, $CDCl_3$): 7.92 – 7.83 (4 H, m), 7.61 (1 H, t, *J* = 7.4 Hz), 7.52 (2 H, t, *J* = 7.7 Hz), 7.25 (2 H, d, *J* = 7.9 Hz), 3.20 (2 H, ddd, *J* = 11.4, 7.1, 3.9 Hz), 3.07 (2 H, ddd, *J* = 11.5, 7.0, 3.9 Hz), 2.39 (3 H, s), 1.63 (2 H, dt, *J* = 13.4, 5.0 Hz), 1.47 (2 H, q, *J* = 5.9 Hz) ppm. – ^{13}C NMR (126 MHz, $CDCl_3$): 142.7, 140.9, 136.1, 133.4, 129.2, 129.1, 127.6, 126.8, 47.1, 25.0, 23.4, 21.6 ppm. – IR: 2940, 2852, 1738, 1598, 1446, 1315, 1258, 1154, 1109, 1087, 1022, 925, 815, 736 cm^{-1}. – HRMS: calcd for $C_{18}H_{22}N_2O_3S_2$: 379.1145, found 379.1145 [M+H$^+$].

(*S*)-4-Methyl-*N*-(morpholino(oxo)(phenyl)- λ⁶-sulfaneylidene)benzenesulfonamide (20)

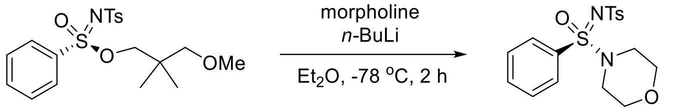

A solution of morpholine (13 mg, 0.15 mmol, 1.5 equiv.) in diethyl ether (1 mL) was cooled to –78 °C, and a 2.5M solution of *n*-butyllithium in hexane (0.068 mL, 0.16 mmol, 1.6 equiv.) was added. The reaction mixture was then stirred for 30 minutes at –78 °C, and a solution of sulfonimidate *ent*-**3a-Me** (41 mg, 0.1 mmol) in diethyl ether (0.5 mL) was added dropwise in 5 minutes. After the completion of reaction (monitored by thin layer chromatography), the reaction mixture was quenched with a mixture of a saturated aqueous solution of EDTA and aqueous ammonium hydroxide (pH = 9), and the organic layer was separated. The aqueous layer was extracted with ethyl acetate (3 × 2 mL), and the combined organic layers were dried over sodium sulfate and concentrated under reduced pressure. The crude product was purified by column chromatography on silica gel with a mixture of hexane and ethyl acetate (7: 3) as an eluent to give sulfonimidate **20** (22 mg, 58%, 90% ee, 100% es) as a colorless liquid.

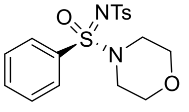

$[\alpha]_D$ = +26 (*c* 0.08, $CHCl_3$). – Daicel Chiralpak IBN (0.46 cm × 25 cm); 90 : 10 v/v, n-hexane / isopropanol; v = 1.2 mL·min-1, λ = 254 nm, , t_R (major) = 29.36 min, t_R (minor) = 34.67 min. – ^1H NMR (500 MHz, $CDCl_3$): 7.90 (4 H, dd, *J* = 21.4, 7.9 Hz), 7.68 (1 H, t, *J* = 7.5 Hz), 7.58 (2 H, t, *J* = 7.7 Hz), 7.30 (2 H, d, *J* = 9.8 Hz), 3.77 (4 H, h, *J* = 8.3 Hz), 3.26 – 3.18 (2 H, m), 3.09 (2 H, dt, *J* = 11.1, 4.2 Hz), 2.43 (3 H, s) ppm. – ^{13}C NMR (125 MHz, $CDCl_3$): 143.0, 140.6, 134.6, 133.9, 129.4, 129.3, 127.7, 126.8, 66.0, 46.3, 21.6 ppm. – IR: 2921, 2858, 2360, 2338, 1452, 1320, 1258, 1155, 1108, 1080, 771, 737, 686 cm^{-1}. – HRMS: calcd for $C_{17}H_{20}N_2O_4S_2$: 381.0937, found 381.0935 $[M+H^+]$.

Computational data

DFT optimization, vibrational analysis, and IRC calculations were conducted with Gaussian 16 (rA.03).[3] Visualizations and monitoring of calculations performed using Chemcraft.[4] Energy decomposition analysis was performed for the diastereomeric transition state structures using the 2nd generation of Absolutely Localized Molecular Orbital Energy Decomposition Analysis (ALMO-EDA2) and Complementary Occupied-Virtual orbital Pairs (COVP)[5] methods as implemented in Q-Chem 5.3.1.[6] Images were rendered using CYLview2.0[7] and VMD 1.9.3.[8] PASDI script[9] was used to automate the distortion/interaction activation strain model (ASM)[10] analysis using IRC from the transition states.

Details of Computational Methods

Ground state minima and transition states were optimized without constraints at the PW6B95-D3BJ / def2-SVP level of theory in dichloromethane (DCM) with the SMD continuum solvation model. Optimizations were performed with a convergence criteria of "tight" and an ultrafine grid. Frequency calculations performed at the same level of theory were used to classify each stationary point. Geometries with no imaginary frequencies were deemed minima, whereas those with exactly one imaginary frequency along the chemical path of interest were deemed transition states. An IRC calculation was performed for each transition state to further corroborate that the transition state connected reactants and products. A cut-off frequency of 50 cm^{-1} was selected for all structures to correct for potential errors associated with low magnitude vibrational frequencies via GoodVibes.[11] Single point calculations were performed at the PW6B95-D3BJ / def2-TZVP / SMD(DCM) level of theory.

Benchmarking studies were carried out to ascertain the suitability of the selected method for the computational mechanistic investigation of the Cu-catalyzed kinetic resolution reaction. An evaluation of the optimized structures of Cu complex **21** obtained with an array of functional and basis set combinations revealed that generally good performance was observed with a number of methods (Table S3), with the lowest RMSD observed for the selected optimization method (entry 4). This result indicates that the selected level of theory can be suitable for the computational study of the Cu-catalyzed kinetic resolution reaction.

Table S3. Evaluation of computational methods for complex **21**.[a]

Entry	Method	RMSD, Å	Entry	Method	RMSD, Å
1	B3LYP-D3BJ / def2-svp	0.81	6	PBE0-D3BJ / def2-SVP	0.67
2	M06-D3 / def2-svp	0.75	7	MN15 / def2-SVP	0.78
3	M06-2X / def2-SVP	0.83	8	M06-L / def2-SVP	0.74
**4**	**PW6B95-D3BJ / def2-SVP**	**0.62**	9	M06-2X-D3 / 6-31+g*	0.84
5	ωB97X-D / def2-SVP	0.91	10	MN15 / 6-31+g*	0.81

[a] Root mean square deviation (RMSD) values were obtained in VMD, using the X-ray structure of complex **21** as a reference structure.

Formation of intermediate complex 22

The computational studies point to a facile dissociation of the apical diol **2** ligand (Figure S9). The following sequence of deprotonation steps of the intermediate complexes **Cu1** and **Cu2** is highly thermodynamically favorable. Collectively, these results suggest that complex **22** is readily formed under the reaction conditions, in line with the experimental kinetic studies.

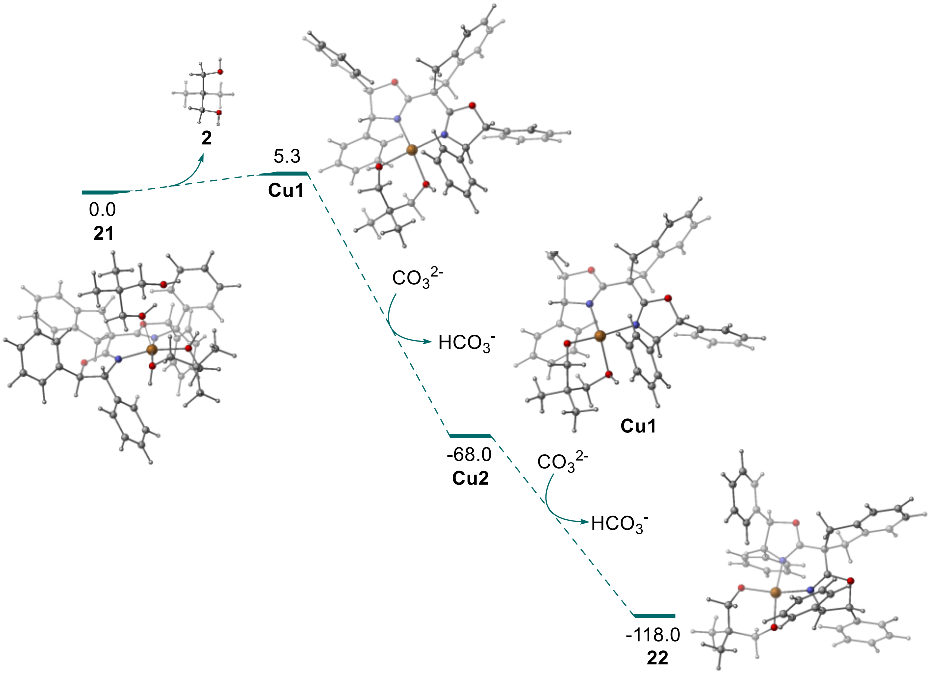

Figure S9. Computed energy profile for the formation of complex **22**, Δ*G*, kcal/mol.

Distortion/Interaction-Activation Strain Analysis

A distortion/interaction-activation strain analysis[10] was performed on transition state structures **TS-R** and **TS-S** at the PW6B95-D3BJ / def2-SVP / SMD (DCM) level of theory as previously described.[9] Fragment definitions were created for each transition state (Figure S10), with the green fragment representing the remaining structure (**F1**) and the red fragment representing the sulfonimidoyl chloride substrate (**F2**).

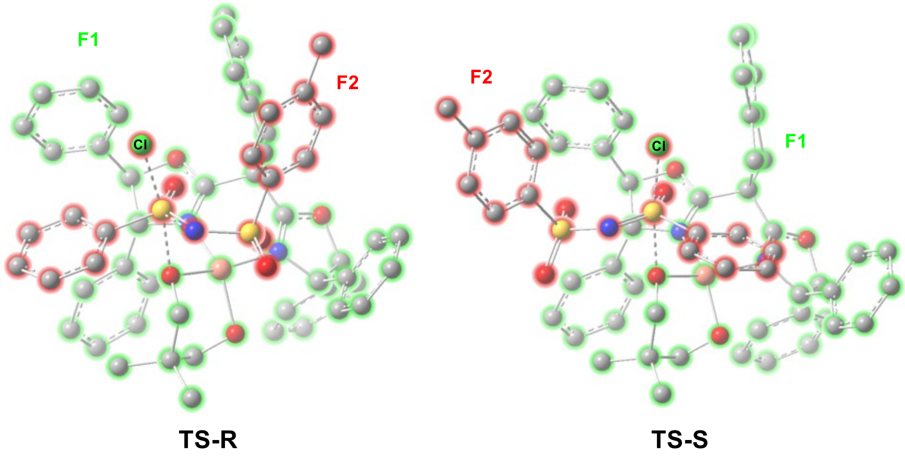

Figure S10. Fragment definition for distortion/interaction-activation strain analysis. Hydrogen atoms removed for clarity.

Distortion–interaction analysis of the substitution pathway

Distortion–interaction analysis of the substitution pathway was carried out using pASDI[9] (a bash script that extracts, creates submission script files, and runs jobs for intermediate geometries obtained along the IRCs of transition states) to investigate the effects of the $\boldsymbol{E}^{\zeta}_{dist}$ and $\boldsymbol{E}^{\zeta}_{int}$ over the course of the reaction for **TS-R** and **TS-S** as previously discussed.[12] The results of analysis are shown in Figure S11. The same fragment definition and level of theory were used as for distortion/interaction-activation strain analysis. The data indicates that stronger interactions lead to an earlier and more energetically favorable transition state structure **TS-R**.

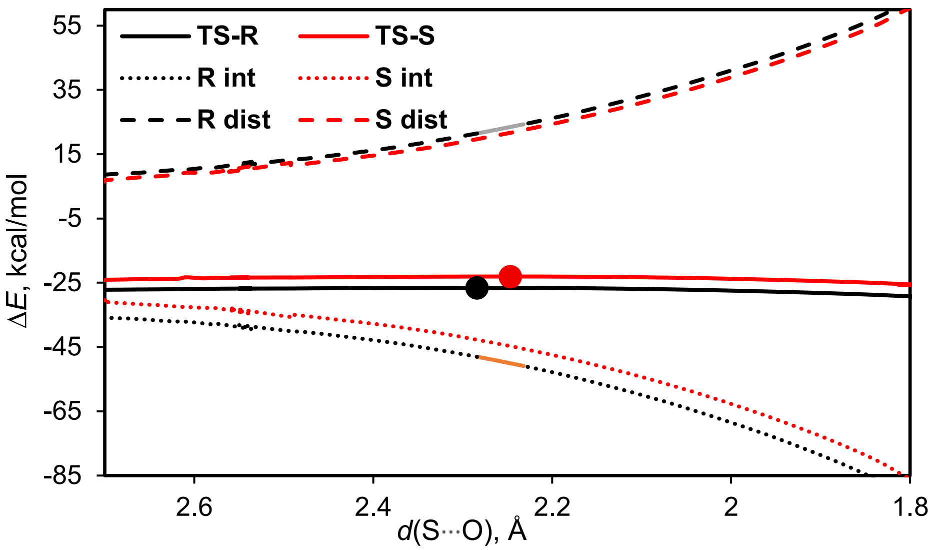

Figure S11. Distortion/interaction-activation strain analysis of **TS-R** and **TS-S** projected along the S···O bond. Large dots represent transition state structures **TS-R** and **TS-S**.

Independent Gradient Model

To identify differences in non-covalent interactions between the chiral substrate and copper catalyst, an independent gradient model based on Hershfield partition (IGMH) for **TS1** and **TS2** structure was implemented. The cube files were generated from the optimized geometries at PW6B95-D3BJ / def2-SVP / SMD(DCM) level of theory using Multiwfn[13] and exported and rendered in VMD. An isovalue of 0.05 was used to display the isosurface and are visualized in Figure S12. The color of the isosurface signifies a specific interaction. A green region represents Van der Waals (vdw) interaction, blue represents a strong attractive interaction, and red represents a strong repulsive interaction.

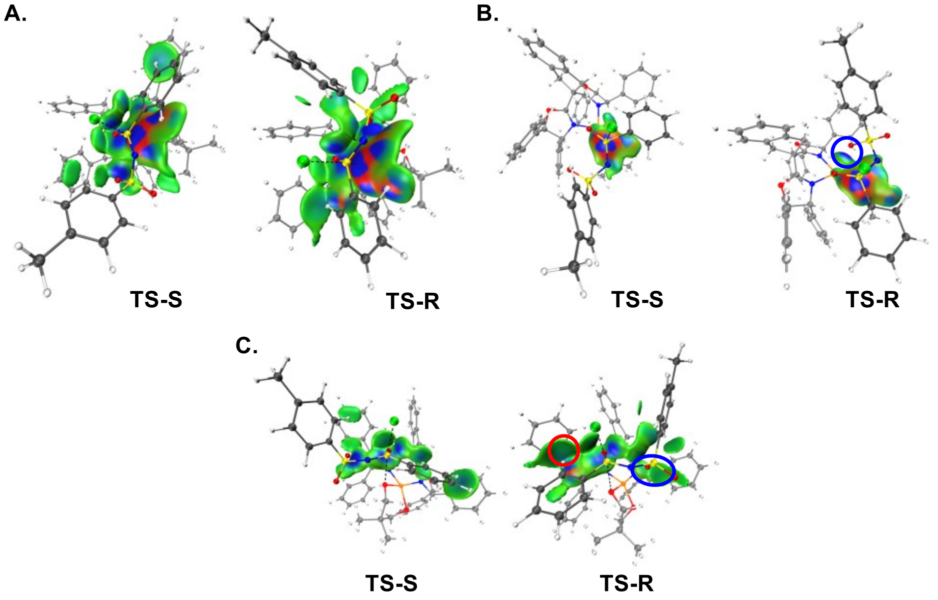

Figure S12. IGMH for **TS-R** and **TS-S**. **A.** Complete map of the interactions between the substrate and catalyst. **B.** Only interactions between diol **2** and *ent*-**1a**. **C.** Interactions between the ligand **L1** and substrate *ent*-**1a** fragments.

Energy Decomposition Analysis via ALMO-EDA2

The second generation Absolutely Localized Molecular Orbital Energy Decomposition Analysis (ALMO-EDA2) method of Head-Gordon and co-workers was employed to gain quantitative insight into the intermolecular forces governing the interaction energies of transition states **TS-R** and **TS-S**. ALMO-EDA2 calculations[14] were performed

at the PW6B95-D3BJ / def2-SVP / SMD(DCM) level of theory in Q-Chem using the optimized geometries at the same level of theory. The results of ALMO-EDA2 are tabulated in Table S4.

Table S4. Tabulated values (in kcal/mol) of energy decomposition analysis of **TS-R** and **TS-S**.

Structure	prep	ΔE_{Pauli}	ΔE_{Elec}	ΔE_{CT}	ΔE_{Disp}	ΔE_{Pol}	ΔE_{Solv}	Total $\Delta E^{\ddagger}_{int}$
**TS-R**	-0.02	103.02	-56.08	-45.88	-47.43	-10.85	7.58	-49.66
**TS-S**	-0.02	96.84	-56.33	-43.25	-40.85	-9.72	9.33	-43.99

Complementary Occupied-Virtual Orbital Pairs

To gain insight into the dominant orbital interactions contributing to the ΔE_{CT} terms of **TS-R** and **TS-S**, complementary occupied-virtual orbital pairs (COVP) analysis was performed in conjunction with the ALMO-EDA2 method. Cube files were collected at the PW6B95-D3BJ / def2-SVP / SMD(DCM) level of theory and generated in VMD using an isovalue of 0.08 for both donor and acceptor orbitals. The COVPs that contribute >0.5 kcal/mol of the stabilization energy for each transition state structure are presented in Figures S10–S13. Donor orbitals are represented as an opaque surface while acceptor orbitals are transparent.

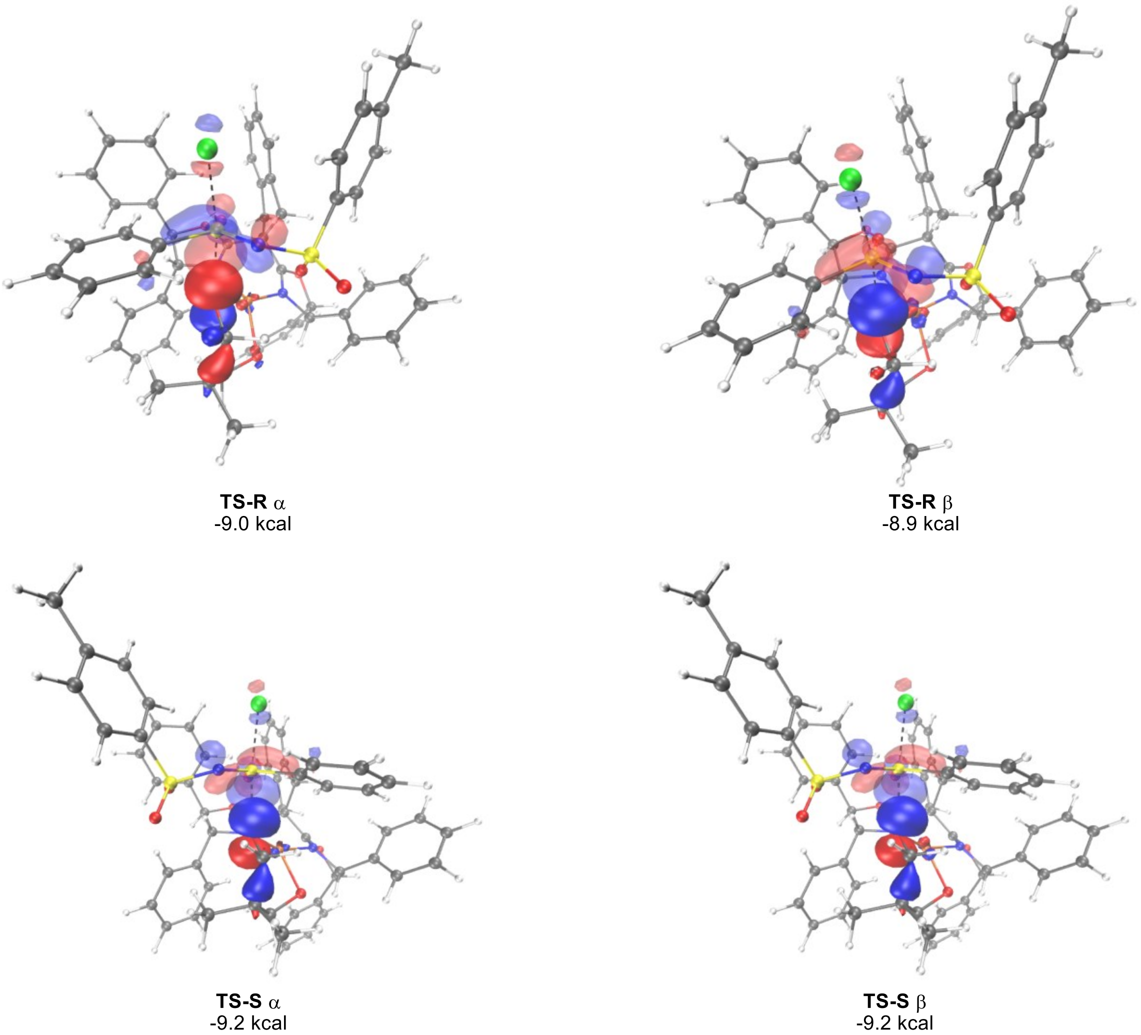

Figure S13. Dominant COVPs for **TS-R** and **TS-S**. The dominant COVPs correspond to the orbital interaction of the lone pair on diol oxygen with the σ* of S–Cl in line with proposed nucleophilic substitution mechanism.

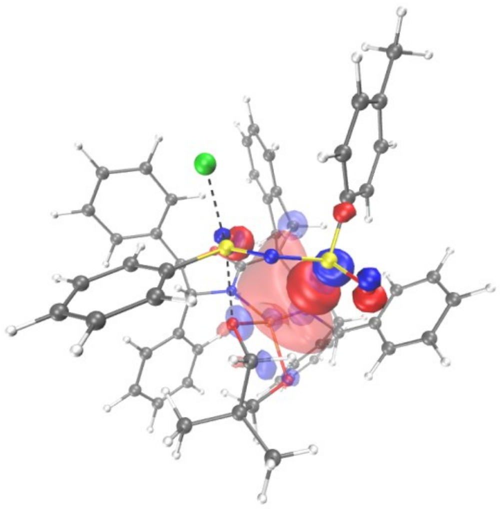

TS-R α
-1.7 kcal

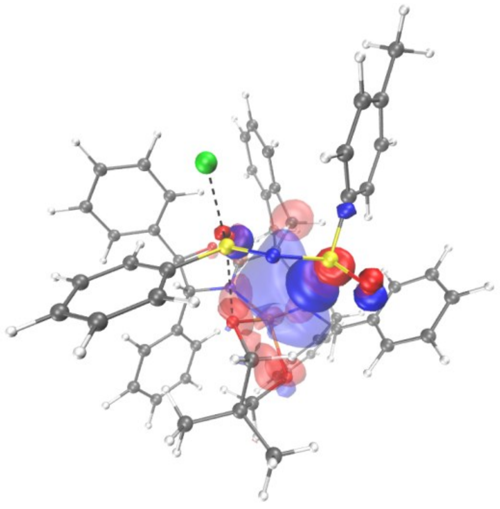

TS-R β
-1.8 kcal

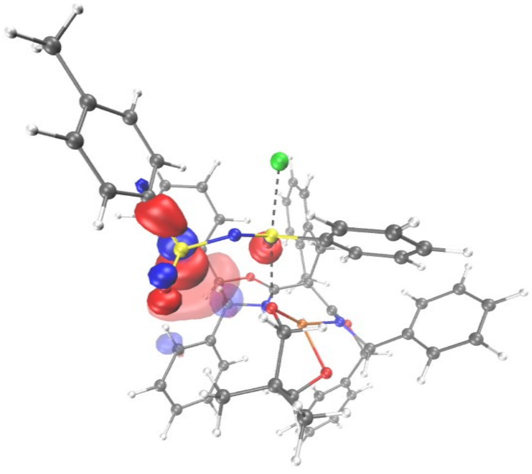

TS-S α
-0.8 kcal

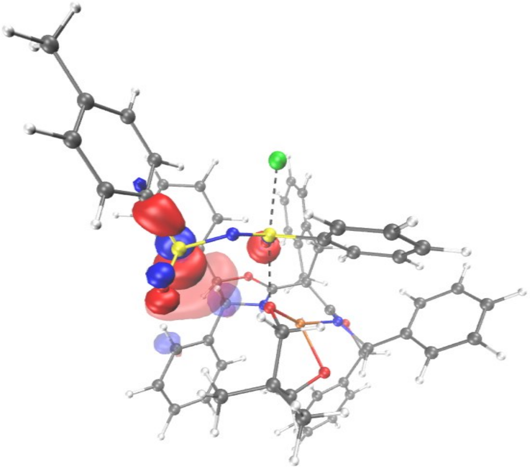

TS-S β
-1.2 kcal

Figure S14. COVPs for the secondary orbital interactions of the *N*-tosyl group in the sulfonimidoyl chloride fragment in **TS-R** and **TS-S**. The oxygen lone pair interacts with either the σ* of the *α*-C–H bond in the proximal oxazoline ring (**TS-S**) or with the σ* over Cu (**TS-R**). Isovalue for donor orbitals of **TS-R** were set to ±0.05.

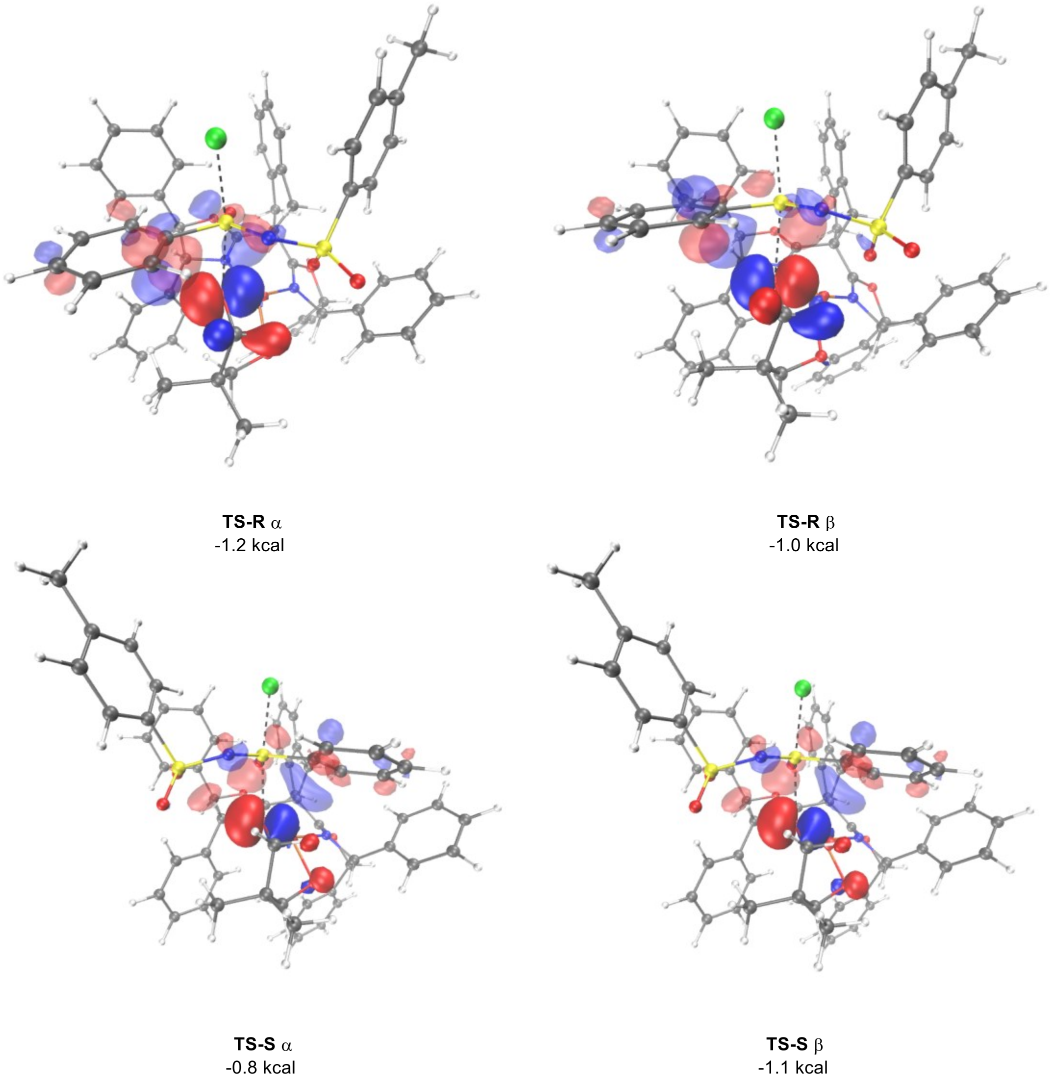

Figure S15. COVPs for the secondary orbital interactions of the diol **2** fragment in **TS-R** and **TS-S**. The lone pairs of oxygen interact with a π* of the sulfonimidoyl chloride fragment.

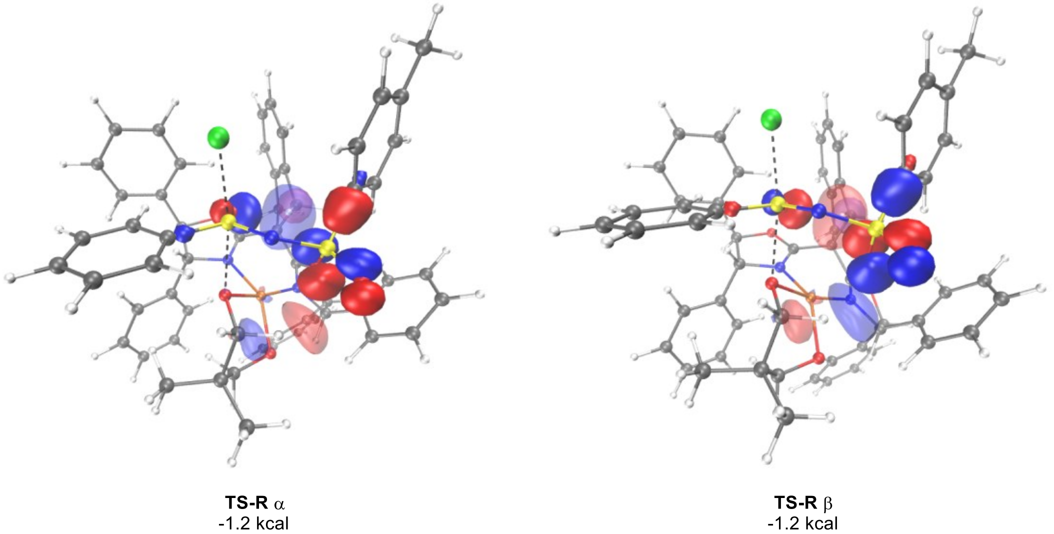

Figure S16. COVPs for the secondary orbital interactions of an oxygen lone pair of the tosyl group with σ* orbitals of the C–H bonds in the diol and indane moieties of the complex **13** fragment in **TS-R**. The interaction is not observed for **TS-S**.

To gain further insight into the orbitals involved in the σ^* shown in Figure S14 for **TS-R**, the natural atomic orbitals (NAO) were calculated via NBO7 (Figure S17). NBO analysis was carried out at the PW6B95-D3BJ / def2-SVP / DCM (SMD) level of theory. The NAO contributions of atoms involved in the COVP were isolated from MO 383: this MO was identified as the acceptor orbital from the Q-chem output file and directly related to the orbitals shown in Figure S14.

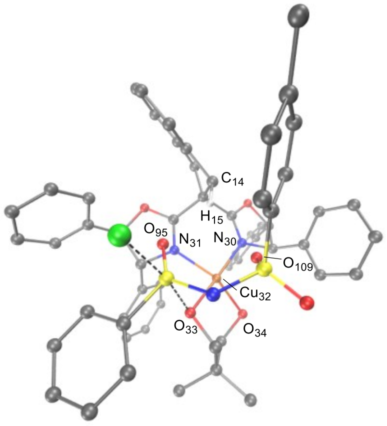

Atom	Contribution
C14	0.62
H15	0.07
N30	0.33
N31	0.26
Cu32	0.47
O33	0.18
O34	0.16
O95	0.04
O109	0.12

Figure S17. Natural atomic orbital contributions for selected atoms in MO 383 for **TS-R**. The absolute contributions of each atom is tabulated. Hydrogen atoms were removed for clarity.

Dispersion Energies

The dispersion energies between the ligand and substrate for **TS-R** and **TS-S** were calculated using HF-D4 dispersion correction and tabulated in Table S5. For the previously optimized transition states structures, a van der Waals complex was established between the sulfoximine substrate and the ligand: a total of 4 complexes were calculated for each transition state corresponding to the proximal phenyl rings, the distal phenyl rings, the indane moiety, or the bis(oxazoline) rings of the ligand with the sulfoximine substrate. A hydrogen atom was used to convert a van der Waals complex into a closed-shell molecule. The dispersion energy (ΔE_{disp}) was calculated from:

$$\Delta E_{disp} = E_{disp(lig+sub)} - \left(E_{disp(lig)} + E_{disp(sub)}\right)$$

where $E_{disp(lig+sub)}$ is the total dispersion energy of the van der Waals complex, $E_{disp(lig)}$ is the dispersion energy of the ligand substitutent, and $E_{disp(sub)}$ is the dispersion energy from the sulfoximine substrate. Dispersion energies were calculated using DFT-D4 software[15] with HF as the reference functional.

Table S5. Dispersion energies for **TS-R** and **TS-S**, kcal/mol.

Name	Complex	Ligand	Substrate	Dispersion energy
TS-S proximal	-165.18	-52.25	-106.06	-6.87
TS-S distal	-165.18	-52.25	-106.06	-6.87
TS-S indane	-156.01	-46.34	-106.06	-3.62
TS-S bis(oxazoline)	-161.24	-46.56	-106.06	-8.62
TS-R proximal	-167.46	-52.29	-107.29	-7.88
TS-R distal	-166.05	-52.23	-107.29	-6.53
TS-R indane	-161.49	-46.47	-107.29	-7.73
TS-R bis(oxazoline)	-162.90	-46.69	-107.29	-8.92

Effective oxidation state analysis using intrinsic bonding orbitals

Calculations were performed to determine the effective oxidation state (EOS) of the metal center and the ligands in complex **21** and transition state structure **TS-R**. Each structure was partitioned into fragments for fragment-level analysis of the electronic populations. The electronic population of each complex was partitioned and assigned to fragments using intrinsic bonding orbitals (IBOs). The fragment definitions used for EOS analysis for each complex

are presented sequentially, below, in addition to the partial charges and spin for each fragment. Wavefunctions for IBO analysis were imported as Molden files from Orca single point calculations performed at the UPW6B95-D3BJ / def2-TZVP / SMD(DCM) level of theory. The previously optimized Gaussian 16 geometries were used for EOS analysis.

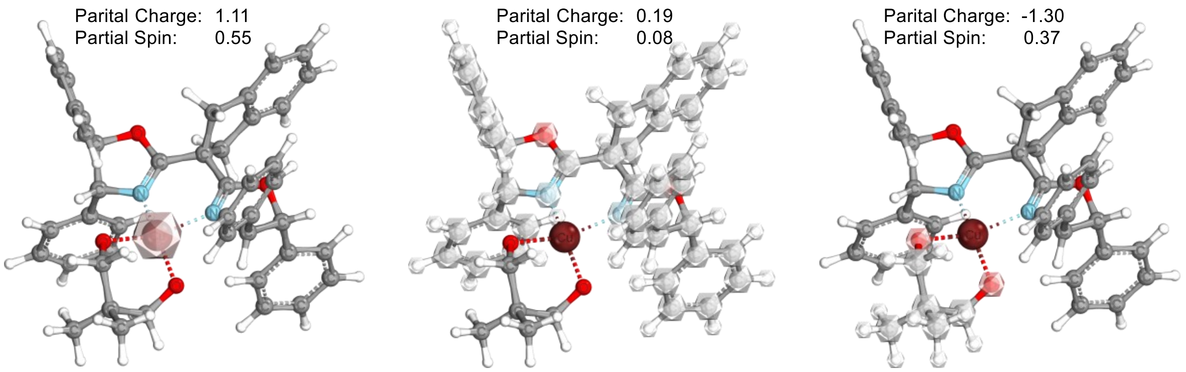

Figure S18. Fragment definitions used for complex **21** and the partial charge and partial spin for each fragment.

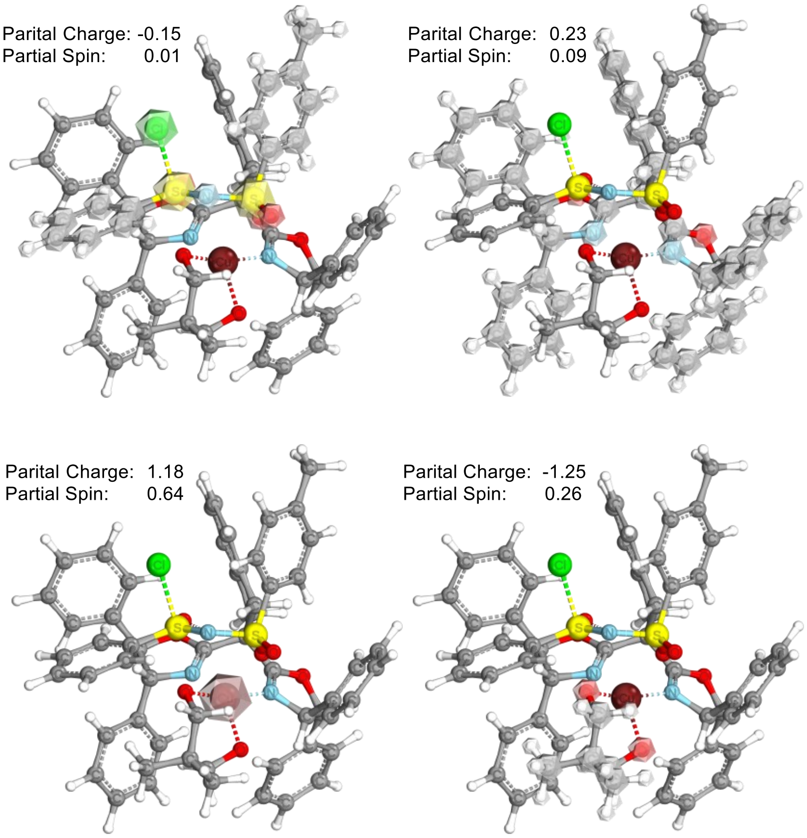

Figure S19. Fragment definitions used for **TS-R** and the partial charge and partial spin of each fragment.

Go to table of contents

Optimized geometries

2

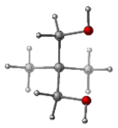

E(RPW6B95D3) = -348.356072263

Charge = 0 Multiplicity = 1

C	0.1661375475	-0.6371687256	0.0479621653
C	-1.3617987340	-0.6221006675	-0.0125123101
H	-1.7045953072	0.4267555276	0.0611814212
H	-1.6621850468	-0.9838253345	-1.0125262205
C	0.7005084953	0.1210774599	1.2636717346
H	1.7860299838	0.2688012488	1.1177742677
H	0.2429248456	1.1276710509	1.2773989703
C	0.6723405187	0.0893302677	-1.1960853631
H	1.7690815059	0.1084803450	-1.2204865975
H	0.3253795490	-0.4094371950	-2.1097067744
H	0.3179627785	1.1289021429	-1.2266483431
C	0.6716904320	-2.0750254623	0.0688794190
H	0.3257399975	-2.6235056843	-0.8174910185
H	1.7695664744	-2.1030294035	0.0775893646
H	0.3041765798	-2.5899608721	0.9611599491
O	0.4492777074	-0.5734927724	2.4555698999
H	0.8747936298	-0.0910425718	3.1715086234
O	-1.9269835311	-1.4180311859	0.9938975643
H	-2.8768041061	-1.4508637578	0.8420566178

21

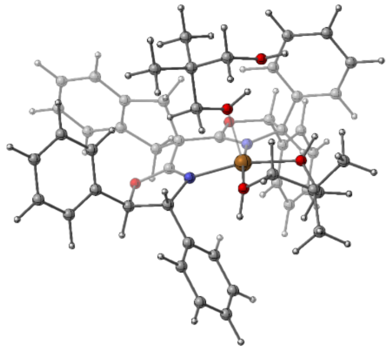

E(UPW6B95D3) = -4104.22551154

Charge = 2 Multiplicity = 2

C	0.58877	2.35935	1.25393
C	1.66992	1.47899	1.23347
C	2.96284	1.94372	1.02934
C	3.16217	3.31102	0.84838
C	2.08203	4.19136	0.8666
C	0.78578	3.72108	1.06835
H	3.80755	1.25658	1.01781
H	4.16963	3.69462	0.69819
H	2.25341	5.25755	0.72962
H	-0.05494	4.41244	1.08998
C	-0.69841	1.62192	1.48578
H	-1.38026	2.13267	2.17269
H	-1.23235	1.46609	0.54178
C	1.20958	0.06854	1.44713
H	1.83991	-0.53052	2.11384
H	1.13533	-0.47361	0.49398
C	-0.23281	0.25432	2.04314
C	-0.08408	0.31758	3.53367
C	0.17122	-0.16787	5.70343
C	0.4748	1.33955	5.45354
H	1.07378	-0.67119	6.07082
H	-0.22319	1.98401	5.9941
C	-1.06436	-0.90434	1.59197
C	-1.93453	-2.94347	1.29028
C	-2.28833	-2.03681	0.08145
H	-2.84383	-3.33671	1.75467
H	-1.88235	-2.43299	-0.85212
O	0.18631	1.49818	4.04042
O	-1.53727	-0.8198	0.37035
N	-1.27626	-2.0088	2.21
N	-0.1138	-0.66059	4.36041
Cu	-0.57134	-2.5379	3.98414
O	-0.078	-3.14186	5.89379
O	-1.42083	-4.38614	3.86894
C	1.89158	1.73581	5.73498
C	2.19112	2.42615	6.90784
C	2.92252	1.3714	4.86611
C	3.5105	2.74405	7.21527
H	1.38493	2.7183	7.57945
C	4.23819	1.70082	5.16883
H	2.70143	0.84284	3.93929
C	4.5348	2.38361	6.34624
H	3.73684	3.28486	8.13223
H	5.03571	1.42805	4.48011
H	5.56583	2.64073	6.58157
C	-1.0206	-4.09068	0.93609
C	-1.51964	-5.39398	0.90315
C	0.31871	-3.86211	0.61343
C	-0.68822	-6.45674	0.55976
H	-2.56543	-5.57397	1.15014
C	1.14651	-4.92359	0.26501
H	0.72494	-2.85157	0.65084
C	0.6456	-6.22254	0.23973
H	-1.08598	-7.46965	0.53914
H	2.18928	-4.73621	0.01815
H	1.29623	-7.05239	-0.02946
C	-0.95619	-0.44434	6.66456
C	-0.67678	-0.55312	8.02919
C	-2.26821	-0.61966	6.2199
C	-1.69704	-0.81831	8.93649
H	0.34754	-0.43175	8.37979
C	-3.28621	-0.89504	7.12907
H	-2.50068	-0.5404	5.15879
C	-3.00376	-0.99201	8.48793
H	-1.46914	-0.89686	9.99776
H	-4.30548	-1.02976	6.77186
H	-3.80074	-1.20593	9.19732
C	-3.73986	-1.7056	-0.07955
C	-4.37453	-1.95124	-1.29494
C	-4.46911	-1.1568	0.97943
C	-5.72444	-1.65239	-1.45523
H	-3.80557	-2.37865	-2.11929
C	-5.81347	-0.85007	0.81618

H	-3.98522	-0.96943	1.93865
C	-6.44336	-1.09915	-0.40187
H	-6.21332	-1.8485	-2.40757
H	-6.37547	-0.41774	1.64179
H	-7.49797	-0.86088	-0.52731
C	-2.01781	-4.88652	5.07096
H	-2.55961	-4.03393	5.49519
H	-2.75513	-5.65419	4.80869
C	-0.99238	-5.43056	6.06463
C	-0.54294	-6.83172	5.66527
H	-0.18613	-6.85624	4.62616
H	0.27967	-7.17542	6.30491
H	-1.36728	-7.54796	5.76111
C	-1.64026	-5.43926	7.4455
H	-1.84973	-4.41886	7.79578
H	-2.59199	-5.98471	7.43062
H	-0.98396	-5.92326	8.17797
C	0.24212	-4.52781	6.04739
H	0.88374	-4.76856	5.19407
H	0.83363	-4.65876	6.9616
H	-1.06221	-5.09251	3.30974
H	-0.55999	-2.80098	6.66324
O	2.84681	-5.28484	3.21345
O	1.47063	-3.07968	3.42969
H	2.57736	-1.54187	4.17685
H	2.42574	-2.93071	5.27042
C	3.90589	-3.19088	3.71822
C	2.57088	-2.63849	4.21846
C	5.01652	-2.57707	4.5626
C	4.10024	-2.84836	2.24397
C	3.93432	-4.70449	3.91987
H	4.86708	-2.77814	5.63135
H	5.99361	-2.98398	4.2755
H	5.05225	-1.4896	4.42561
H	3.31389	-3.29231	1.62295
H	4.08254	-1.76131	2.09435
H	5.0668	-3.22295	1.88514
H	3.85949	-4.93331	4.99452
H	4.88937	-5.10864	3.55248
H	1.67496	-3.99476	3.15094
H	2.83097	-6.23885	3.35273

Cu1

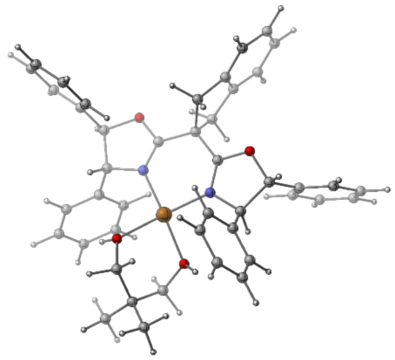

E(UPW6B95D3) = -3758.33419532

Charge = 2 Multiplicity = 2

C	-0.56534	2.68786	1.68971
C	0.72344	2.38144	1.26024
C	1.58632	3.38485	0.83756
C	1.13912	4.70404	0.85045
C	-0.15173	5.01026	1.27805
C	-1.01493	4.0023	1.70132
H	2.59645	3.14749	0.50829
H	1.80517	5.50262	0.5289
H	-0.48626	6.04597	1.28743
H	-2.021	4.24329	2.04019
C	-1.31521	1.45241	2.08661
H	-1.8456	1.55168	3.04006
H	-2.05813	1.17874	1.32689
C	0.9784	0.90544	1.31135
H	1.94473	0.64424	1.75806
H	0.95266	0.46338	0.30799
C	-0.2112	0.34601	2.16363
C	0.26213	0.22514	3.57622
C	0.87641	-0.44824	5.62415
C	1.09444	1.08414	5.48644
H	1.83425	-0.94736	5.80871
H	0.44786	1.64702	6.16429
C	-0.72992	-0.9316	1.58991
C	-1.41978	-3.02772	1.19173
C	-1.65145	-2.14895	-0.06788
H	-2.38011	-3.40598	1.55838
H	-1.06043	-2.49544	-0.91914
O	0.60521	1.35707	4.13786
O	-1.08567	-0.86426	0.32985
N	-0.89087	-2.06904	2.16386
N	0.39693	-0.83014	4.29513
Cu	-0.26684	-2.63936	3.93074
O	0.6888	-3.43086	5.5456
O	-1.17796	-4.45233	3.81905
C	2.51239	1.54885	5.60438
C	2.83848	2.56952	6.49454
C	3.51571	0.97009	4.82147
C	4.15458	3.00841	6.60582
H	2.05535	3.02261	7.1006
C	4.82647	1.41582	4.92506
H	3.27344	0.16607	4.1253
C	5.14762	2.43483	5.82
H	4.40226	3.80478	7.30487
H	5.60289	0.9658	4.30939
H	6.17612	2.78121	5.90293
C	-0.48644	-4.19699	0.99644
C	-1.01885	-5.46337	0.73981
C	0.89857	-4.04542	1.09596
C	-0.17691	-6.56045	0.58203
H	-2.09883	-5.58684	0.66846
C	1.73808	-5.14434	0.94208
H	1.3274	-3.06282	1.29121
C	1.20245	-6.40309	0.68592
H	-0.60145	-7.54229	0.38212
H	2.81581	-5.01554	1.02074
H	1.8601	-7.26199	0.56819
C	-0.09478	-0.86083	6.7026
C	0.39053	-1.21985	7.96275
C	-1.46931	-0.91898	6.45775
C	-0.48682	-1.62828	8.96291

H	1.46156	-1.18247	8.15756
C	-2.34367	-1.33514	7.4574
H	-1.8618	-0.63904	5.48053
C	-1.85456	-1.6909	8.71069
H	-0.09838	-1.90529	9.94099
H	-3.41235	-1.37817	7.25598
H	-2.53879	-2.01768	9.49114
C	-3.0854	-1.96476	-0.4565
C	-3.50622	-2.28538	-1.74488
C	-4.01172	-1.47828	0.47135
C	-4.84082	-2.1235	-2.10625
H	-2.78414	-2.66273	-2.4676
C	-5.34037	-1.30732	0.10659
H	-3.69643	-1.22751	1.48527
C	-5.75686	-1.63234	-1.1832
H	-5.16282	-2.3775	-3.11428
H	-6.05637	-0.92204	0.83002
H	-6.79943	-1.50083	-1.46676
C	-1.45297	-5.08554	5.07671
H	-1.85385	-4.28962	5.7139
H	-2.24647	-5.82775	4.93185
C	-0.21458	-5.72255	5.70816
C	0.0734	-7.08809	5.09157
H	0.15101	-7.02953	3.99665
H	1.02107	-7.49333	5.46741
H	-0.72191	-7.79963	5.34157
C	-0.47765	-5.84494	7.20594
H	-0.58309	-4.85674	7.67697
H	-1.40636	-6.39759	7.39446
H	0.34119	-6.37815	7.70235
C	0.99611	-4.82892	5.44193
H	1.3699	-4.96332	4.42098
H	1.81482	-5.06175	6.13268
H	-0.91569	-5.09328	3.1375
H	0.40803	-3.19108	6.44422

Cu2

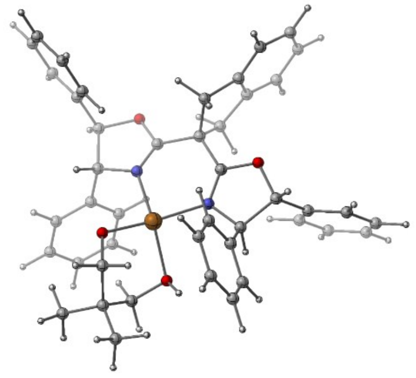

E(UPW6B95D3) = -3755.37302289

Charge = 1 Multiplicity = 2

C	-0.6973989722	2.5851005674	1.7194799299
C	0.5977576397	2.3332126391	1.2709468380
C	1.4170155615	3.3741697772	0.8525595381
C	0.9214252425	4.6758097740	0.8878971858
C	-0.3749603320	4.9275900594	1.3335125844
C	-1.1950099106	3.8817454452	1.7522984087
H	2.4314974125	3.1789457736	0.5089133674
H	1.5536597289	5.5030011609	0.5700044213
H	-0.7479090610	5.9499139472	1.3605575781
H	-2.2056530213	4.0805638754	2.1050816671
C	-1.3928464219	1.3136040280	2.1039012070
H	-1.9331746812	1.3742303425	3.0554322824
H	-2.1209898643	1.0173941968	1.3369843424
C	0.9049004857	0.8666067411	1.2987046295
H	1.8915574987	0.6342105926	1.7165531973
H	0.8704385416	0.4393955491	0.2897272504
C	-0.2436453196	0.2549440871	2.1638401089
C	0.2589994175	0.1412406442	3.5719184666
C	0.9526487479	-0.5263244349	5.5875355448
C	1.1551128420	1.0083924753	5.4480019522
H	1.9220112321	-1.0229600800	5.7134803484
H	0.5285543146	1.5659961292	6.1495465721
C	-0.7267027707	-1.0440266139	1.6005862232
C	-1.3736716547	-3.1525303003	1.2637381986
C	-1.5709057779	-2.3317627754	-0.0369342765
H	-2.3120113790	-3.6104237597	1.5885624049
H	-1.0044455630	-2.7424794227	-0.8758473662
O	0.6300381854	1.2822179534	4.1172762633
O	-0.9461867186	-1.0528737785	0.2975509621
N	-0.9872971151	-2.1257342931	2.2264593189
N	0.4016584146	-0.9082866571	4.2900186230
Cu	-0.4858238610	-2.6852853010	4.0769871705
O	0.7947580205	-3.6585408074	5.4057153235
O	-1.6504847651	-4.1525033100	4.0748546899
C	2.5757602018	1.4770223084	5.5245802715
C	2.9350478043	2.4880633108	6.4126632390
C	3.5503921054	0.9071690429	4.7001529796
C	4.2548557994	2.9255644234	6.4810491194
H	2.1749430141	2.9347174211	7.0520418151
C	4.8645571775	1.3511997835	4.7604861492
H	3.2809748284	0.1096092502	4.0066081389
C	5.2188791292	2.3606057374	5.6538481227
H	4.5282527733	3.7142585152	7.1793616223
H	5.6173585610	0.9069404672	4.1120104075
H	6.2500719232	2.7055496242	5.7034131922
C	-0.3117275552	-4.2231680678	1.2026120942
C	-0.6767155659	-5.5674377868	1.1835359403
C	1.0433654605	-3.8859668319	1.1889102808
C	0.2952366969	-6.5615191386	1.1489785789
H	-1.7318224101	-5.8342027685	1.2155116928
C	2.0169669632	-4.8790818275	1.1546637914
H	1.3490753323	-2.8405893745	1.2173454646
C	1.6446296502	-6.2199937262	1.1365604653
H	-0.0026396165	-7.6084385140	1.1426776038
H	3.0701456034	-4.6041631091	1.1467020812
H	2.4052418381	-6.9983971319	1.1160777304
C	0.0486402035	-0.9630425320	6.7133646394
C	0.6018823407	-1.4488065166	7.8995376114
C	-1.3415451512	-0.9095746694	6.5800508998
C	-0.2220938839	-1.8744432365	8.9375330744
H	1.6851411533	-1.4973582390	8.0056013365
C	-2.1637100099	-1.3376812721	7.6172788051
H	-1.7839783936	-0.5386648550	5.6556703145
C	-1.6058834102	-1.8216375065	8.7970635207
H	0.2195936172	-2.2539010441	9.8570382597
H	-3.2449342856	-1.2972573974	7.5007224835
H	-2.2497000927	-2.1609661852	9.6061999773

C	-2.9977117379	-2.0875068034	-0.4275126277
C	-3.4096427734	-2.2886779272	-1.7429426521
C	-3.9245743297	-1.6435729959	0.5212056468
C	-4.7310251543	-2.0502142563	-2.1104493019
H	-2.6898390033	-2.6354473392	-2.4829984154
C	-5.2404453052	-1.3961345574	0.1523716781
H	-3.6166110851	-1.4933182309	1.5562464102
C	-5.6460397176	-1.6011587163	-1.1648652886
H	-5.0446185768	-2.2137057359	-3.1397920042
H	-5.9550426669	-1.0474780919	0.8956049575
H	-6.6789138483	-1.4120551072	-1.4517307734
C	-1.7210767757	-4.7282138994	5.3276136557
H	-1.7520715583	-3.9672655771	6.1383536237
H	-2.6685157985	-5.2908129210	5.4325214225
C	-0.5625049781	-5.6993653246	5.6374366364
C	-0.7614507464	-7.0118399137	4.8910095769
H	-0.9779141170	-6.8102172090	3.8352094355
H	0.1323902057	-7.6485847843	4.9423279783
H	-1.6011285060	-7.5765108804	5.3154179156
C	-0.4902629231	-5.9301810821	7.1419585200
H	-0.2229066329	-5.0111003467	7.6849777685
H	-1.4627562696	-6.2593470253	7.5311797168
H	0.2514442539	-6.6984775176	7.3950305683
C	0.7257854323	-5.0659800368	5.1233931744
H	0.7731640840	-5.1392170398	4.0310988173
H	1.6121189818	-5.5580268290	5.5455777265
H	0.6498728570	-3.5023096906	6.3517683245

22

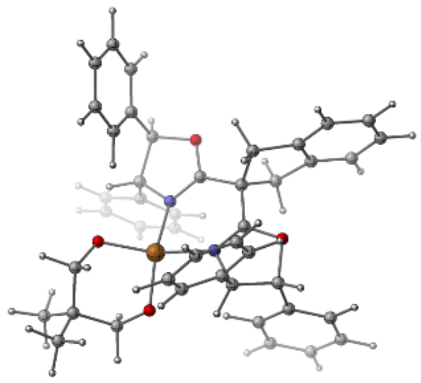

E(UPW6B95D3) = -3754.89143700

Charge = 0 Multiplicity = 2

C	-0.8691961964	2.5502190303	1.8851664554
C	0.4467537409	2.4150643883	1.4432835739
C	1.1912209700	3.5326861535	1.0880515040
C	0.6021499180	4.7923933498	1.1825369390
C	-0.7125929619	4.9275319880	1.6247188128
C	-1.4587413272	3.8046999383	1.9783937212
H	2.2209080407	3.4291386910	0.7493942547
H	1.1760002552	5.6780866519	0.9150867771
H	-1.1582675261	5.9179482656	1.7001441394
H	-2.4843625363	3.9110987266	2.3283516154
C	-1.4730255500	1.2129945209	2.1916082329
H	-2.0542450604	1.1891499071	3.1208316089
H	-2.1408076054	0.8883644983	1.3835942701
C	0.8585386154	0.9732350029	1.4178360052
H	1.8582371577	0.7943977687	1.8294115682
H	0.8499388231	0.5831070295	0.3919565182
C	-0.2410565201	0.2547986723	2.2588400724
C	0.1841471085	0.1735744673	3.6939642579
C	0.3865312304	-0.3154995029	5.8505414086
C	1.2381529952	0.9038791374	5.5184470641
H	0.9511586782	-1.1000170281	6.3594601091
H	1.0671244646	1.7592807534	6.1748540432
C	-0.5493740155	-1.0833804575	1.6631320445
C	-0.6610705523	-3.2388126073	1.1515672901
C	-1.4707327663	-2.4205386198	0.1330551042
H	-1.2559986840	-4.0381029461	1.6002280669
H	-1.1989794479	-2.6217871831	-0.9051487404
O	0.7134193792	1.2824969031	4.2000084808
O	-1.0166764661	-1.0568976491	0.4197638934
N	-0.3826517053	-2.2390341356	2.1787842491
N	0.0406446330	-0.8019457101	4.5036602203
Cu	-0.2609125896	-2.7620786602	4.1370332533
O	0.5125074341	-3.2950492027	5.8004818130
O	-1.1678788578	-4.3975805465	3.8087015307
C	2.7092929415	0.6318631701	5.3590639152
C	3.6017250030	1.7044900259	5.4060411876
C	3.1973380536	-0.6519897380	5.0936630067
C	4.9597580501	1.5044549146	5.1877205213
H	3.2242354297	2.7049936867	5.6139780940
C	4.5567574958	-0.8492386381	4.8726112594
H	2.5222372544	-1.5076421256	5.0684638733
C	5.4391753225	0.2257686897	4.9177889360
H	5.6463319021	2.3480617370	5.2313478783
H	4.9269073004	-1.8525506106	4.6693846110
H	6.5026538714	0.0660355007	4.7483816243
C	0.6108008455	-3.8424469637	0.6052162262
C	0.5757915656	-5.1440781607	0.1008111026
C	1.8115323788	-3.1330036385	0.5628790862
C	1.7149266731	-5.7218659737	-0.4474866912
H	-0.3544300016	-5.7092281503	0.1458432672
C	2.9535128586	-3.7119979819	0.0170745504
H	1.8653732853	-2.1262190199	0.9733551930
C	2.9081817902	-5.0060055685	-0.4915509241
H	1.6731679115	-6.7389172923	-0.8335425282
H	3.8847998984	-3.1485709260	-0.0054594279
H	3.8028870005	-5.4591620160	-0.9148603694
C	-0.9021743082	-0.0760510481	6.5999931200
C	-1.4931303589	-1.1689748844	7.2392848879
C	-1.5613668516	1.1543208183	6.5969253761
C	-2.7194316510	-1.0291987431	7.8789110448
H	-0.9774354046	-2.1294373170	7.1972820584
C	-2.7879884562	1.2923350496	7.2413411650
H	-1.1274680045	2.0136522499	6.0872903674
C	-3.3680914996	0.2032934874	7.8844391895
H	-3.1714019524	-1.8864765520	8.3754177472
H	-3.2928535271	2.2569126169	7.2384734233
H	-4.3269933131	0.3148999875	8.3878042435
C	-2.9614158297	-2.4623662632	0.3269641788
C	-3.7955520677	-2.1173961452	-0.7379119919
C	-3.5278063275	-2.7775261496	1.5668763414
C	-5.1751433016	-2.0789893956	-0.5705949103
H	-3.3563852368	-1.8770611053	-1.7054748182
C	-4.9083259227	-2.7342475458	1.7321527649
H	-2.8961014948	-3.0762011159	2.4049591153

C	-5.7334046002	-2.3843198890	0.6673664866
H	-5.8162006410	-1.8131100198	-1.4092484422
H	-5.3408377239	-2.9830839075	2.6996361706
H	-6.8135224696	-2.3564273734	0.8006847025
C	-1.5234479812	-4.9869842748	5.0035868555
H	-2.0297980826	-4.2714879761	5.6870740588
H	-2.2651208041	-5.7939362795	4.8251337935
C	-0.3466867871	-5.6102196871	5.7793782349
C	0.0760196331	-6.9163305755	5.1201834760
H	0.2182746841	-6.7583097298	4.0424313596
H	1.0225546456	-7.2920500466	5.5355334444
H	-0.6810763326	-7.7015393504	5.2538596297
C	-0.7869394757	-5.8421234110	7.2184405294
H	-0.9450655659	-4.8843958990	7.7333603122
H	-1.7308715447	-6.4047845879	7.2644839538
H	-0.0350340077	-6.4089220151	7.7846382025
C	0.8498047313	-4.6326142003	5.7263088130
H	1.4221848952	-4.8601288196	4.8019215260
H	1.5319680295	-4.9002444181	6.5602930407

1a

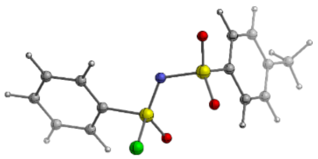

E(RPW6B95D3) = -2040.51267089

Charge = 0 Multiplicity = 1

S	0.0512952587	0.8248497938	-2.6970294767
N	-0.7881203152	1.6034796024	-3.7177592044
S	-1.1352161350	3.2376321003	-3.5237645133
O	-2.2334242986	3.4865552218	-4.4376916939
O	-1.2717543140	3.5294859674	-2.1034012529
C	0.3123241522	4.0370953422	-4.1365566874
C	0.4197494244	4.2880603174	-5.5018828879
C	1.3175033690	4.3963751918	-3.2473683588
C	1.5657227812	4.9051596167	-5.9771013521
H	-0.3892249702	4.0084954641	-6.1729055300
C	2.4568932036	5.0154180406	-3.7447114416
H	1.2060166008	4.1935094798	-2.1850654498
C	2.6014539954	5.2769929109	-5.1099633013
H	1.6599790004	5.1091787984	-7.0426910505
H	3.2503791250	5.3044282657	-3.0576612393
C	3.8304791284	5.9431210228	-5.6438696229
H	4.3403465387	5.2973613855	-6.3705687699
H	3.5753287192	6.8728357102	-6.1685423952
H	4.5373995863	6.1833780009	-4.8435722767
O	1.3367515998	1.2964122834	-2.2081369179
Cl	-1.1172880624	0.5103090720	-0.9537278152
C	0.1516048844	-0.8136037426	-3.3248255646
C	-0.9055572547	-1.3405162740	-4.0600529712
C	1.3054799270	-1.5287457717	-3.0261398257
C	-0.7854932525	-2.6438134318	-4.5202243063
H	-1.7886027690	-0.7416600887	-4.2682795577
C	1.4019775816	-2.8316805786	-3.4992718665
H	2.1048887420	-1.0747509245	-2.4457928302
C	0.3618916821	-3.3842261311	-4.2403335377
H	-1.5925571275	-3.0831586715	-5.1021661274
H	2.2958641539	-3.4139006196	-3.2871535506
H	0.4449250449	-4.4057103538	-4.6060956246

***ent*-1a**

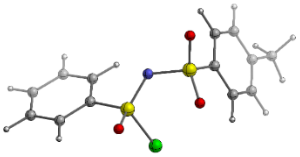

E(RPW6B95D3) = -2040.51302068

Charge = 0 Multiplicity = 1

S	-1.3912331546	-0.7463098687	0.4365262483
N	-0.2008445373	0.1758958613	0.7187974620
S	-0.3734532234	1.8327576614	0.9416803191
O	0.7410995752	2.4268988375	0.2282464185
O	-1.7434004618	2.2269390351	0.6400816002
C	-0.0957817059	2.0028773865	2.6749473380
C	1.2111900791	1.9685061993	3.1566731984
C	-1.1811962548	2.1735969088	3.5239351773
C	1.4215203394	2.0968366921	4.5195370837
H	2.0456856233	1.8502432683	2.4688333317
C	-0.9473506704	2.3050765374	4.8872406799
H	-2.1898069401	2.2036846925	3.1193031504
C	0.3491307226	2.2664351796	5.4062364470
H	2.4386814761	2.0716976968	4.9076596669
H	-1.7901234079	2.4417982949	5.5627678992
C	0.6005999172	2.4160965714	6.8736174350
H	1.2114504843	1.5881517197	7.2546665088
H	1.1545375173	3.3415680863	7.0801682583
H	-0.3345709981	2.4463061281	7.4414558091
O	-2.2628858724	-0.5917915430	-0.7123353795
Cl	-2.7012391393	-0.6988619227	2.1239461537
C	-0.7602901480	-2.3813039598	0.5562566662
C	-1.3549262105	-3.3439672724	-0.2513892758
C	0.2679589529	-2.6637654737	1.4502349417
C	-0.8848098901	-4.6482654215	-0.1604910874
H	-2.1582844071	-3.0760062422	-0.9331044300
C	0.7193584483	-3.9735311811	1.5232667793
H	0.7026206097	-1.8770770666	2.0617505549
C	0.1451806276	-4.9591159081	0.7220843059
H	-1.3260850000	-5.4219452628	-0.7847191360
H	1.5260080608	-4.2252896134	2.2080168418
H	0.5073685878	-5.9831690209	0.7872410334

TS-R

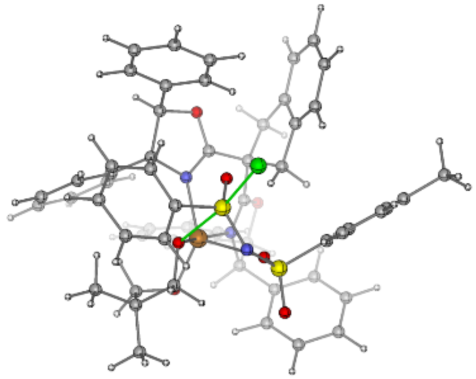

E(UPW6B95D3) = -5799.34307183

Charge = 0 Multiplicity = 2

C	2.34186	1.95524	2.14421
C	3.17283	0.97267	2.69132
C	4.41882	1.29449	3.21335
C	4.83488	2.62514	3.17072
C	4.0138	3.60547	2.6163
C	2.7592	3.27832	2.10028
H	5.03881	0.53088	3.68105
H	5.80173	2.90263	3.58738
H	4.34822	4.64143	2.59998
H	2.11459	4.05318	1.68817
C	1.03562	1.34607	1.70107
H	0.16842	1.9902	1.87412
H	1.06232	1.09577	0.63262
C	2.493	-0.35728	2.63268
H	2.67563	-1.02235	3.4789
H	2.76982	-0.89851	1.71563
C	0.99087	0.04532	2.52116
C	0.47026	0.29409	3.91415
C	-0.25141	-0.02659	5.99097
C	-0.02595	1.49622	5.73482
H	0.43508	-0.38795	6.76042
H	-0.9598	2.06095	5.81908
C	0.26607	-1.12646	1.924
C	-0.34524	-3.22502	1.52329
C	-0.88844	-2.30653	0.42304
H	-1.12868	-3.8319	1.9829
H	-0.61172	-2.6233	-0.58493
O	0.37734	1.5464	4.34822
O	-0.15705	-1.0531	0.67093
N	0.13165	-2.25137	2.5114
N	0.15142	-0.62158	4.73363
Cu	0.37586	-2.64416	4.48575
O	1.05414	-3.01411	6.29461
O	-0.77778	-4.18074	4.41787
C	1.03181	2.06239	6.6333
C	0.67707	2.4499	7.92734
C	2.37094	2.08764	6.25005
C	1.65745	2.83572	8.83496
H	-0.37056	2.43778	8.22735
C	3.34954	2.47698	7.15916
H	2.65189	1.79055	5.24321
C	2.99748	2.84364	8.45427
H	1.3742	3.13308	9.8433
H	4.39345	2.4814	6.85159
H	3.76537	3.14079	9.16619
C	0.81057	-4.12094	1.1504
C	0.97833	-5.31605	1.84807
C	1.77184	-3.73347	0.21442
C	2.08748	-6.11903	1.61015
H	0.2516	-5.57619	2.61624
C	2.88086	-4.53913	-0.02561
H	1.6658	-2.79356	-0.32744
C	3.04143	-5.73244	0.67357
H	2.21738	-7.04256	2.17129
H	3.62609	-4.2301	-0.75684
H	3.91344	-6.35808	0.49047
C	-1.64645	-0.42371	6.38783
C	-1.91914	-0.75638	7.71354
C	-2.67197	-0.48618	5.44323
C	-3.19694	-1.15751	8.09158
H	-1.11555	-0.72292	8.44845
C	-3.94861	-0.88742	5.81931
H	-2.46498	-0.235	4.40368
C	-4.21259	-1.22884	7.14375
H	-3.39523	-1.42753	9.12732
H	-4.742	-0.93752	5.07584
H	-5.21044	-1.55183	7.43523
C	-2.35956	-2.00979	0.50223
C	-3.03291	-1.60448	-0.65106
C	-3.05996	-2.08726	1.71048
C	-4.3828	-1.27552	-0.6037
H	-2.49044	-1.54955	-1.59409
C	-4.41058	-1.75866	1.75579
H	-2.55893	-2.41428	2.62119
C	-5.07383	-1.35118	0.60186
H	-4.89808	-0.96537	-1.51098
H	-4.94991	-1.83311	2.69813
H	-6.13243	-1.10048	0.64119
C	-1.37091	-4.28093	5.66081
H	-1.73155	-3.29824	6.03346
H	-2.28061	-4.9148	5.60352
C	-0.45321	-4.88494	6.74684
C	-0.44152	-6.4043	6.6371
H	-0.2387	-6.71142	5.60256
H	0.33222	-6.85064	7.278
H	-1.40904	-6.83053	6.93502
C	-0.92437	-4.436	8.12354
H	-0.6932	-3.37313	8.27206
H	-2.00992	-4.56702	8.24081
H	-0.43002	-5.00446	8.92358
C	0.97522	-4.39028	6.49785
H	1.38674	-4.94201	5.63546
H	1.58732	-4.67907	7.3692
S	3.15435	-2.15744	6.56669
O	2.66863	-1.08128	5.71883
N	3.74076	-3.52491	6.14409
C	2.6349	-2.18071	8.25077
C	2.08812	-1.00087	8.74044
C	2.82308	-3.31398	9.02798
C	1.63824	-0.99023	10.05304
H	2.035	-0.11028	8.11892
C	2.36689	-3.27632	10.34254

H	3.31572	-4.19276	8.62217
C	1.76353	-2.12922	10.84706
H	1.19759	-0.08116	10.45747
H	2.49028	-4.15394	10.97343
H	1.40684	-2.1135	11.87482
S	3.74468	-4.00028	4.56568
O	2.58259	-3.45925	3.85225
O	3.94019	-5.43927	4.60083
C	5.17055	-3.21839	3.87594
C	5.08692	-2.71753	2.58083
C	6.35751	-3.1654	4.59777
C	6.21014	-2.13558	2.0117
H	4.14985	-2.78597	2.03553
C	7.46885	-2.57415	4.01288
H	6.40336	-3.5543	5.61228
C	7.41438	-2.04615	2.71876
H	6.14994	-1.73724	0.99984
H	8.39909	-2.51491	4.57553
C	8.60547	-1.37645	2.10825
H	8.47358	-0.2855	2.10637
H	9.52184	-1.60046	2.66378
H	8.74439	-1.68381	1.06502
Cl	5.12087	-1.13587	7.13126

TS-S

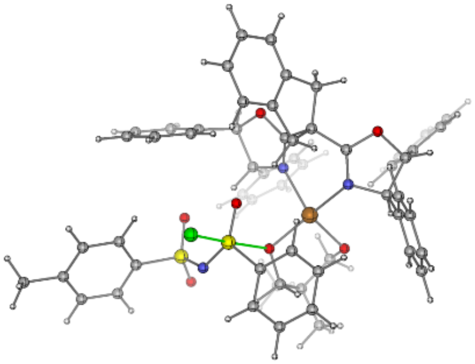

E(UPW6B95D3) = -5799.34010130

Charge = 0 Multiplicity = 2

C	2.2927	1.99434	1.85362
C	3.22495	1.20142	2.52393
C	4.45361	1.71968	2.9131
C	4.74307	3.05077	2.61825
C	3.81517	3.84295	1.94421
C	2.58223	3.31977	1.55716
H	5.16812	1.10244	3.456
H	5.69615	3.4776	2.92578
H	4.05068	4.88369	1.72815
H	1.85506	3.94602	1.0426
C	1.04788	1.20904	1.55268
H	0.12417	1.77771	1.69818
H	1.05579	0.85422	0.51511
C	2.6872	-0.17943	2.7425
H	2.89548	-0.58205	3.73921
H	3.09064	-0.88969	2.00676
C	1.1506	0.	2.50692
C	0.5413	0.31328	3.84881
C	-0.27629	0.15976	5.91274
C	0.04333	1.65043	5.57612
H	0.36197	-0.19044	6.7285
H	-0.86713	2.25828	5.56617
C	0.55997	-1.27367	1.97592
C	0.0339	-3.41889	1.6922
C	-0.60732	-2.56967	0.58307
H	-0.70865	-4.01216	2.22944
H	-0.36179	-2.9112	-0.42479
O	0.52064	1.58632	4.21146
O	0.0657	-1.27389	0.74987
N	0.53857	-2.3865	2.60654
N	0.12079	-0.53785	4.69637
Cu	0.34649	-2.56753	4.62911
O	0.88472	-2.87335	6.47876
O	-0.57591	-4.22718	4.48014
C	1.0785	2.26497	6.46816
C	0.6761	2.93023	7.62514
C	2.43771	2.10284	6.20723
C	1.6239	3.41223	8.52144
H	-0.38607	3.06059	7.83056
C	3.38506	2.59237	7.09812
H	2.75653	1.58918	5.30467
C	2.98073	3.2427	8.261
H	1.30147	3.92456	9.42629
H	4.44362	2.44823	6.88708
H	3.72249	3.61849	8.96395
C	1.14446	-4.33816	1.25074
C	1.10607	-5.68437	1.61074
C	2.21699	-3.86774	0.48762
C	2.11208	-6.55466	1.20012
H	0.28328	-6.04447	2.22612
C	3.22564	-4.73434	0.08169
H	2.26311	-2.81701	0.19961
C	3.17224	-6.082	0.43324
H	2.06919	-7.60426	1.48561
H	4.05507	-4.35828	-0.51469
H	3.96039	-6.76052	0.11153
C	-1.71103	-0.0993	6.28229
C	-2.04732	-0.29513	7.62137
C	-2.7169	-0.12761	5.31397
C	-3.37146	-0.51719	7.98896
H	-1.25696	-0.29141	8.37035
C	-4.03949	-0.34993	5.68108
H	-2.46116	0.02054	4.26495
C	-4.36923	-0.54709	7.02029
H	-3.62147	-0.67817	9.03623
H	-4.81743	-0.36797	4.91986
H	-5.40432	-0.72564	7.3065
C	-2.08531	-2.34806	0.7374
C	-2.86476	-2.08428	-0.38942
C	-2.69032	-2.36329	1.99892
C	-4.22723	-1.83502	-0.26293
H	-2.39811	-2.07785	-1.37366
C	-4.05241	-2.11445	2.12322
H	-2.10408	-2.58476	2.89133
C	-4.82286	-1.84883	0.99446
H	-4.82604	-1.63469	-1.14957
H	-4.51352	-2.13958	3.10857
H	-5.89027	-1.66007	1.09492

Go to table of contents

C	-1.43052	-4.20662	5.56944
H	-1.87521	-3.19794	5.7221
H	-2.29831	-4.87151	5.38443
C	-0.77878	-4.63931	6.90014
C	-0.78049	-6.15977	7.01235
H	-0.36701	-6.61339	6.10154
H	-0.1746	-6.5008	7.86371
H	-1.79938	-6.5452	7.15192
C	-1.54499	-4.00732	8.05238
H	-1.38931	-2.92296	8.0618
H	-2.62455	-4.19618	7.9605
H	-1.21301	-4.40111	9.02159
C	0.6881	-4.18859	6.89889
H	1.24347	-4.88961	6.25152
H	1.08563	-4.2984	7.92331
S	2.9528	-2.0717	6.84047
O	2.5077	-0.97816	5.99342
N	2.74612	-2.34245	8.33675
C	3.46928	-3.55196	6.03193
C	3.98	-4.60385	6.77786
C	3.33292	-3.59233	4.6522
C	4.36529	-5.7522	6.09528
H	4.07066	-4.52444	7.85787
C	3.72598	-4.74855	3.99181
H	2.9095	-2.75341	4.10473
C	4.23931	-5.82475	4.71061
H	4.76416	-6.59592	6.6544
H	3.62192	-4.80445	2.91159
H	4.5404	-6.72882	4.18517
S	1.79003	-1.45293	9.34445
O	0.83873	-2.35958	9.96977
O	1.29733	-0.2541	8.66883
C	2.96033	-0.93979	10.56273
C	2.8982	-1.46142	11.84601
C	3.91258	0.01122	10.20472
C	3.81455	-1.01551	12.7929
H	2.14161	-2.20145	12.09524
C	4.81741	0.44003	11.16077
H	3.94464	0.39415	9.1878
C	4.78249	-0.06258	12.46933
H	3.77502	-1.41582	13.80474
H	5.56831	1.18157	10.89143
C	5.76551	0.42081	13.48944
H	6.79637	0.25354	13.15185
H	5.6567	1.50048	13.65654
H	5.63178	-0.08785	14.44944
Cl	5.1458	-1.34013	6.95898

X-Ray crystallographic data

(*S*)-2-Floro-*N*-tosylbenzenesulfonimidoyl chloride (*ent*-1b)

CCDC 2482070

Bond precision: C–C = 0.0046 Å Wavelength = 1.54184

Cell: a = 7.5519(1) b = 14.5650(1) c = 26.8592(2)

α = 90 β = 90 γ = 90

Temperature: 100 K

	Calculated	Reported
Volume	2954.34(5)	2954.34(5)
Space group	P 21 21 21	P 21 21 21
Hall group	P 2ac 2ab	P 2ac 2ab
Moiety formula	$C_{13}H_{11}ClFNO_3S_2$	$C_{13}H_{11}ClFNO_3S_2$
Sum formula	$C_{13}H_{11}ClFNO_3S_2$	$C_{13}H_{11}ClFNO_3S_2$
M_r	347.80	347.80
D_x,g cm^{-3}	1.564	1.564
Z	8	8
Mu (mm^{-1})	5.128	5.128
F000	1424.0	1424.0
F000'	1436.08	
h,k,l_{max}	9,17,32	9,17,32
N_{ref}	5605[3198]	5544
T_{min},T_{max}	0.430,0.569	0.249,0.836
T_{min}'	0.229	

Correction method = # Reported T Limits: T_{min} = 0.249 T_{max} = 0.836 AbsCorr = GAUSSIAN

Data completeness = 1.73/0.99 Theta(max) = 69.992

R(reflections) = 0.0298(5416) wR2(reflections) = 0.0810(5544)

S = 1.036 N_{par} = 405

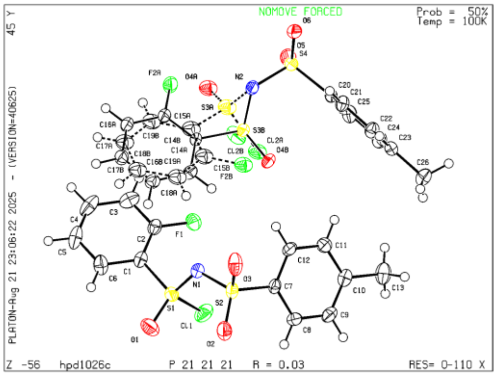

Go to table of contents

(*S*)-4-Bromo-*N*-tosylbenzenesulfonimidoyl chloride (1d)

CCDC 2365713

Bond precision: C–C = 0.0055 Å Wavelength = 1.54184

Cell:	a = 7.3649(1)	b = 9.4538(2)	c = 21.5742(4)
	α = 90	β = 90	γ = 90

Temperature: 100 K

	Calculated	Reported
Volume	1502.13(5)	1502.13(5)
Space group	P 21 21 21	P 21 21 21
Hall group	P 2ac 2ab	P 2ac 2ab
Moiety formula	$C_{13}H_{11}BrClNO_3S_2$	$C_{13}H_{11}BrClNO_3S_2$
Sum formula	$C_{13}H_{11}BrClNO_3S_2$	$C_{13}H_{11}BrClNO_3S_2$
M_r	408.70	409.72
D_x,g cm^{-3}	1.807	1.812
Z	4	4
Mu (mm^{-1})	8.053	8.053
F000	816.0	820.0
F000'	819.08	
h,k,l_{max}	9,11,27	9,11,26
N_{ref}	3202[1861]	2757
T_{min},T_{max}	0.378,0.458	0.545,1.000
T_{min}'	0.103	

Correction method = # Reported T Limits: T_{min} = 0.545 T_{max} = 1.000 AbsCorr = GAUSSIAN

Data completeness = 1.48/0.86 Theta(max) = 77.963

R(reflections) = 0.0278(2689) wR2(reflections) = 0.0716(2757)

S = 1.015 N_{par} = 192

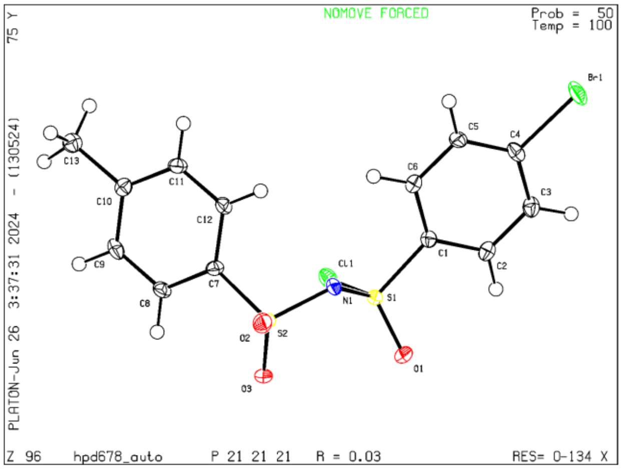

(*S*)-4-Methoxy-*N*-tosylbenzenesulfonimidoyl chloride (*ent*-1g)
CCDC 2481477

Bond precision:	C-C = 0.0043 A		Wavelength=1.54184
Cell:	a = 9.31838(8)	b = 11.02290(11)	c = 15.16584(15)
	α = 90	β = 97.1483(8)	γ = 90
Temperature: 100 K			

	Calculated	Reported
Volume	1545.66(3)	1545.66(3)
Space group	P 21/c	P 1 21/c 1
Hall group	-P 2ybc	-P 2ybc
Moiety formula	C14 H14 Cl N O4 S2	C14 H14 Cl N O4 S2
Sum formula	C14 H14 Cl N O4 S2	C14 H14 Cl N O4 S2
M_r	359.83	359.83
D_x,g cm^{-3}	1.546	1.546
Z	4	4
Mu (mm^{-1})	4.875	4.875
F000	744.0	744.0
F000'	750.01	
h,k,l_{max}	11,13,19	11,13,19
N_{ref}	3246	3038
T_{min},T_{max}	0.433,0.563	0.310,1.000
$T_{min'}$	0.261	

Correction method= # Reported T Limits: T_{min} = 0.310 T_{max} = 1.000 AbsCorr = GAUSSIAN

Data completeness= 0.936 Theta(max)= 76.368

R(reflections)= 0.0475(2936) wR2(reflections)= 0.1145(3038)

S = 1.053 N_{par}= 201

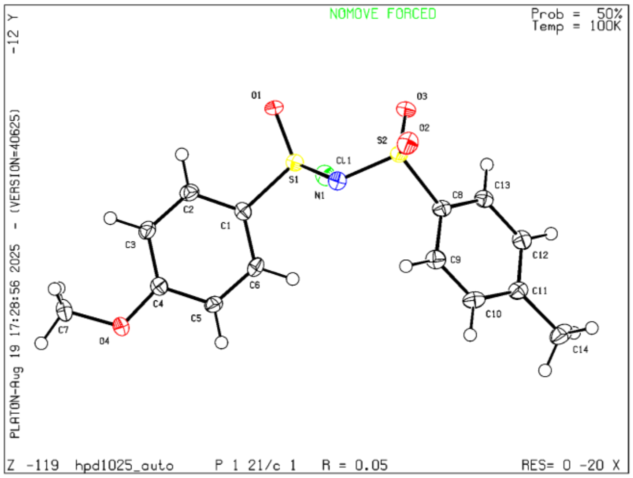

Go to table of contents

3-Hydroxy-2,2-dimethylpropyl (*R*)-*N*-((3-fluorophenyl)sulfonyl)benzenesulfonimidate (*ent*-3x)

CCDC 2365712

Bond precision:	C–C = 0.0050 Å		Wavelength = 1.54184
Cell:	a = 8.73848(7)	b = 31.7118(2)	c = 10.40203(8)
	α = 90	β =106.2808(8)	γ = 90
Temperature:	100 K		

	Calculated	Reported
Volume	2766.95(4)	2766.95(4)
Space group	P 21	P 1 21 1
Hall group	P 2yb	P 2yb
Moiety formula	$C_{17}H_{20}FNO_5S_2$	$C_{17}H_{20}FNO_5S_2$
Sum formula	$C_{17}H_{20}FNO_5S_2$	$C_{17}H_{20}FNO_5S_2$
M_r	401.46	401.46
D_x,g cm^{-3}	1.446	1.446
Z	6	6
Mu (mm^{-1})	2.965	2.965
F000	1260.0	1260.0
F000'	1267.83	
h,k,l_{max}	11,40,13	11,38,13
N_{ref}	11622[5920]	10981
T_{min},T_{max}	0.752,0.875	0.545,1.000
T_{min}'	0.440	

Correction method = # Reported T Limits: T_{min} = 0.545 T_{max} = 1.000 AbsCorr = GAUSSIAN

Data completeness = 1.85/0.94 Theta(max) = 76.572

R(reflections) = 0.0337(10649) wR2(reflections) = 0.0895(10981)

S = 1.055 N_{par} = 712

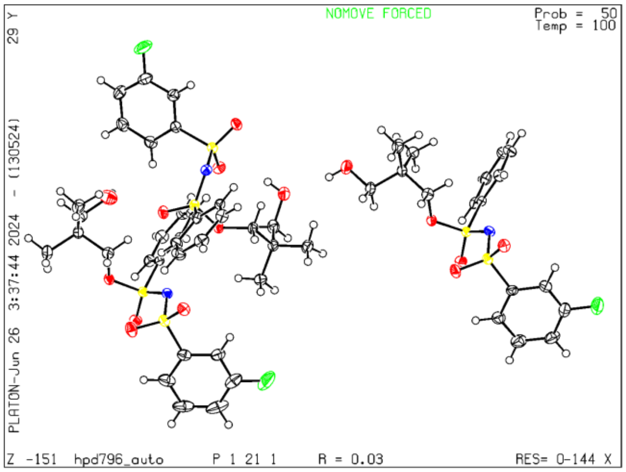

Go to table of contents

(*R*)-4-Methyl-*N*-(oxo(phenyl)(piperidin-1-yl)- λ⁶-sulfaneylidene)benzenesulfonamide (7)

CCDC 2365711

Bond precision:	C-C = 0.0043 A		Wavelength=1.54184
Cell:	a=8.89007(17)	b=10.91158(17)	c=9.92393(19)
	α =90	β =107.893(2)	γ =90
Temperature:	101 K		

	Calculated	Reported
Volume	916.11(3)	916.11(3)
Space group	P 21	P 1 21 1
Hall group	P 2yb	P 2yb
Moiety formula	$C_{18} H_{22} N_2 O_3 S_2$	$C_{18} H_{22} N_2 O_3 S_2$
Sum formula	$C_{18} H_{22} N_2 O_3 S_2$	$C_{18} H_{22} N_2 O_3 S_2$
Mr	378.50	378.49
Dx,g cm-3	1.372	1.372
Z	2	2
Mu (mm-1)	2.801	2.801
F000	400.0	400.0
F000'	402.36	
h,k,lmax	11,13,12	11,13,12
Nref	3849[2030]	2900
Tmin,Tmax	0.723,0.877	0.664,1.000
Tmin'	0.633	

Correction method= # Reported T Limits: Tmin=0.664 Tmax=1.000 AbsCorr = GAUSSIAN

Data completeness= 1.43/0.75 Theta(max)= 76.412

R(reflections)= 0.0304(2803) wR2(reflections)= 0.0789(2900)

S = 1.008 Npar= 227

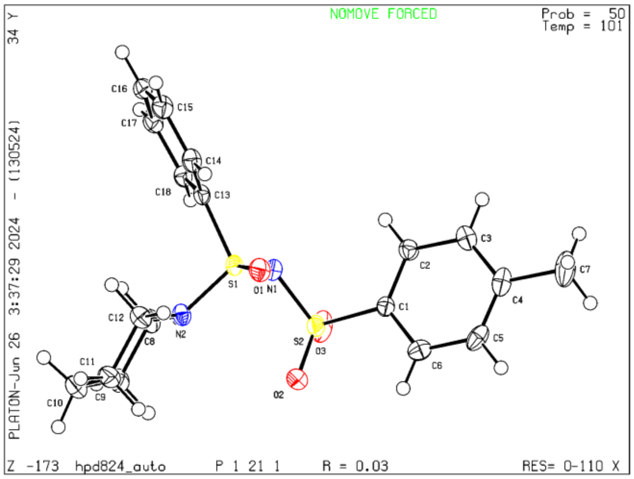

Go to table of contents

Complex of copper (II) triflate with ligand L1 and diol 2 (21)

CCDC 2375817

Bond precision:	C–C = 0.0057 Å		Wavelength = 1.54184
Cell:	a = 12.5474(1)	b = 16.1304(1)	c = 13.7264(1)
	α = 90	β = 107.210(1)	γ = 90
Temperature: 100 K			
	Calculated		Reported
Volume	2653.76(4)		2653.76(4)
Space group	P 21		P 1 21 1
Hall group	P 2yb		P 2yb
Moiety formula	$C_{49}H_{56}CuN_2O_6$, 2(CF_3O_3S)		$C_{49}H_{56}CuN_2O_6$, 2(CF_3O_3S)
Sum formula	$C_{51}H_{56}CuF_6N_2O_{12}S_2$		$C_{51}H_{56}CuF_6N_2O_{12}S_2$
M_r	1130.65		1130.63
D_x,g cm^{-3}	1.415		1.415
Z	2		2
Mu (mm^{-1})	2.036		2.036
F000	1174.0		1174.0
F000'	1175.28		
h,k,l_{max}	15,20,17		15,20,17
N_{ref}	11200[5806]		10563
T_{min},T_{max}	0.710,0.812		0.480,1.000
T_{min}'	0.594		

Correction method = # Reported T Limits: T_{min} = 0.480 T_{max} = 1.000 AbsCorr = GAUSSIAN

Data completeness = 1.82/0.94 Theta(max) = 76.550

R(reflections) = 0.0393(10295) wR2(reflections) = 0.0974(10563)

S = 1.099 N_{par} = 681

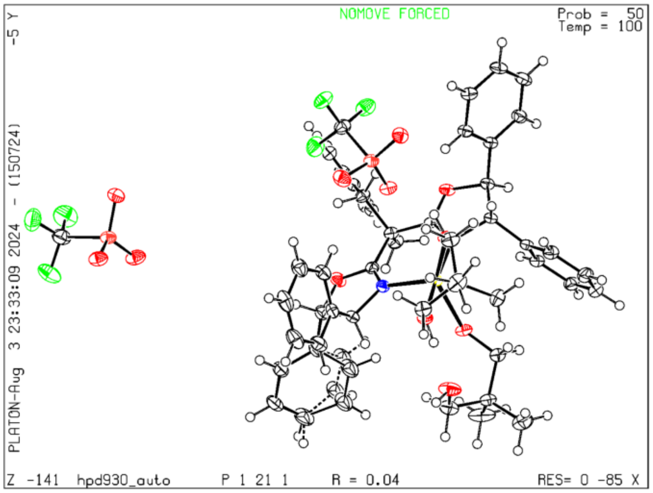

Go to table of contents

NMR Spectroscopic data

(*S*)-*N*-Tosylbenzenesulfonimidoyl chloride (1a)

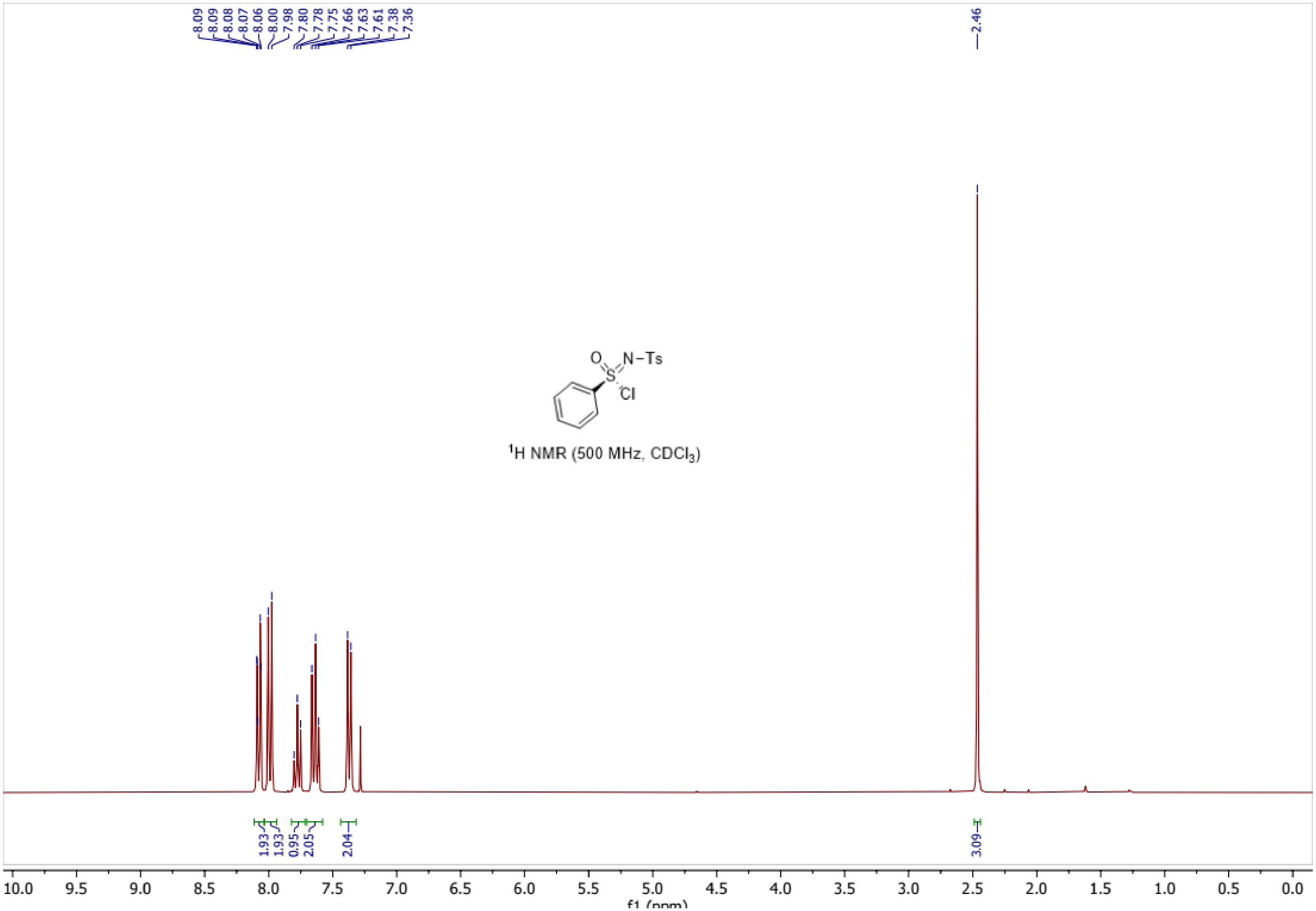

(*S*)-*N*-Tosylbenzenesulfonimidoyl chloride (1a)

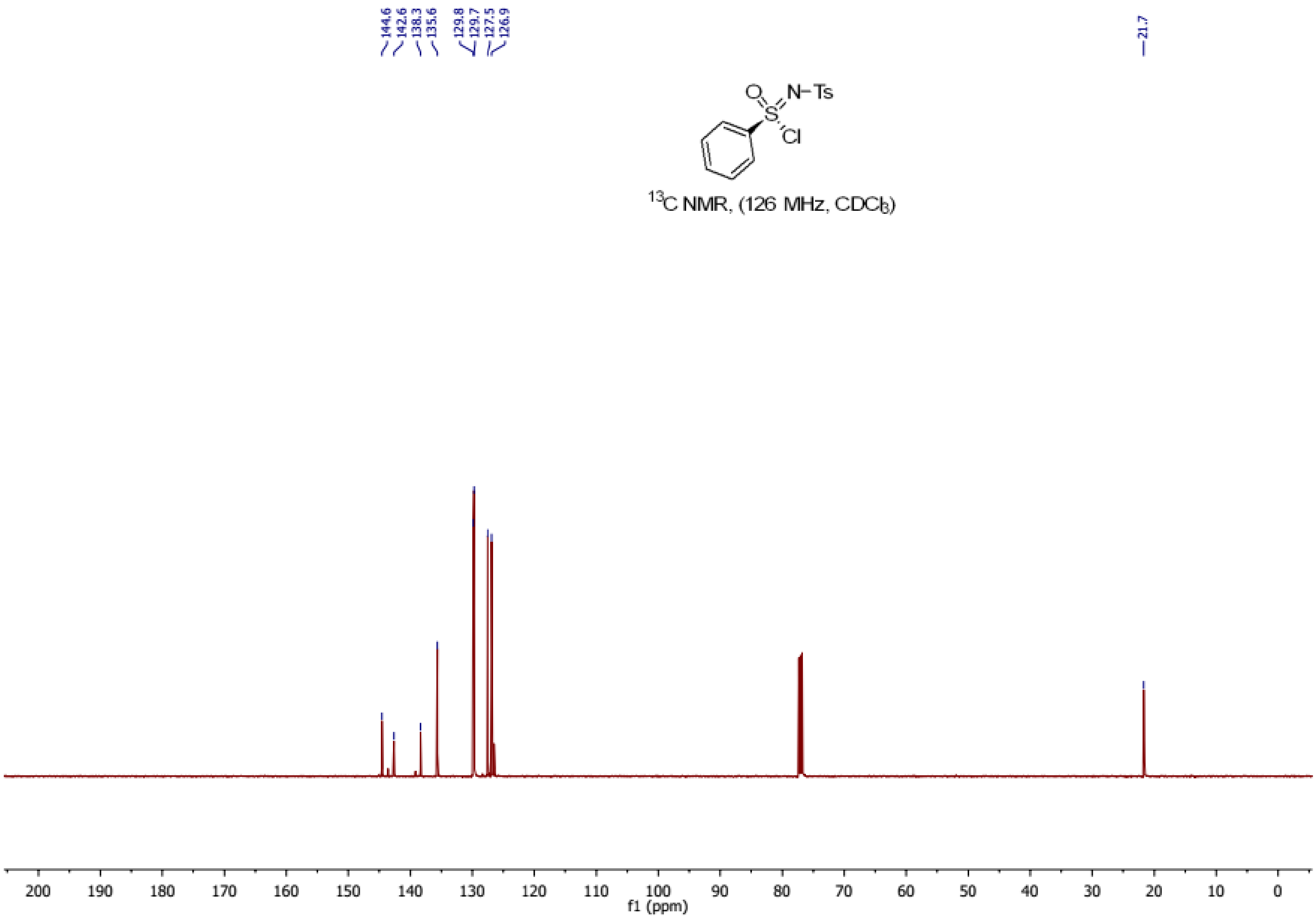

Go to table of contents

(*S*)-2-Floro-*N*-tosylbenzenesulfonimidoyl chloride (1b)

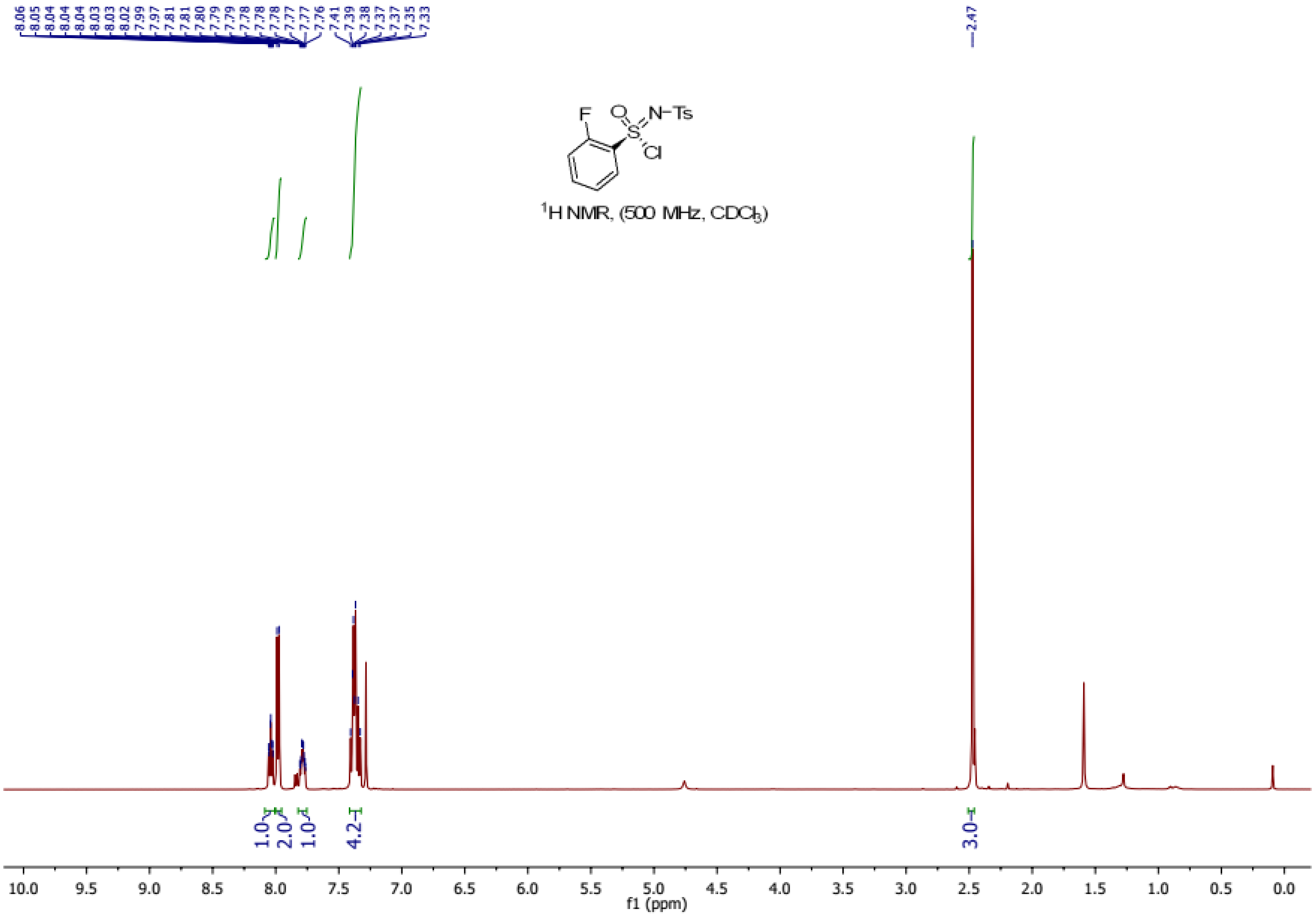

Go to table of contents

(*S*)-2-Floro-*N*-tosylbenzenesulfonimidoyl chloride (1b)

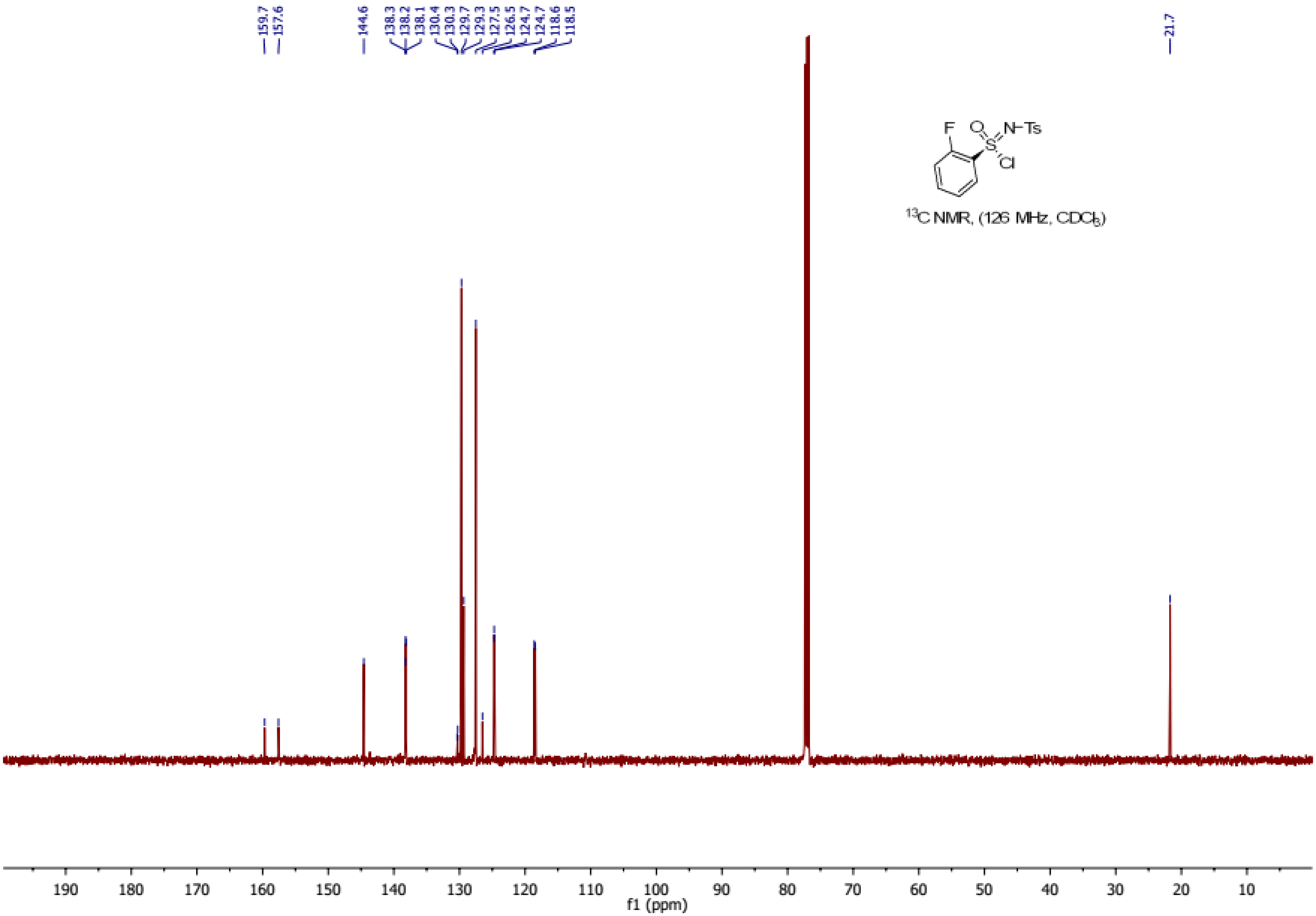

Go to table of contents

(*S*)-4-Chloro-*N*-tosylbenzenesulfonimidoyl chloride (1c)

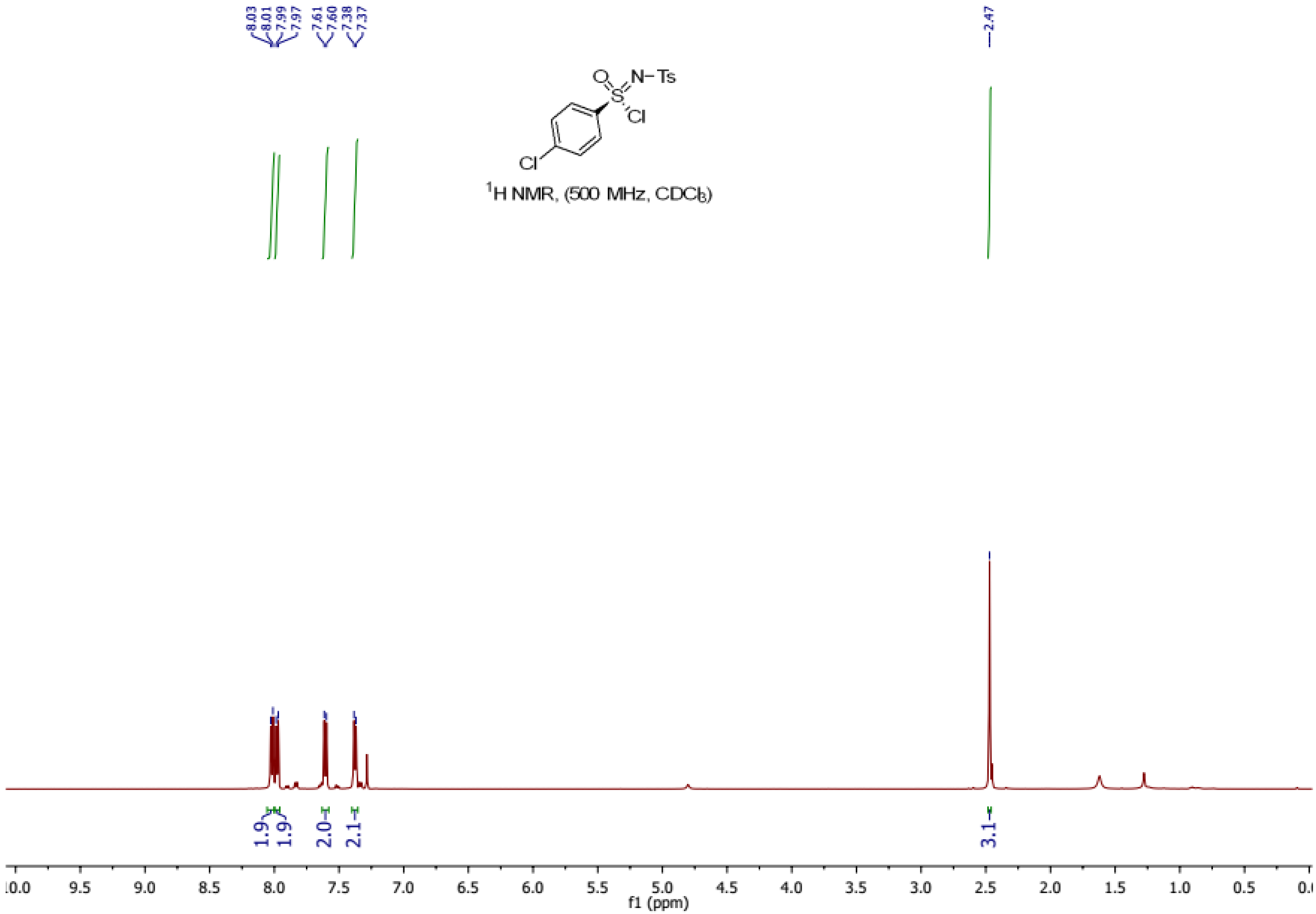

Go to table of contents

(*S*)-4-Chloro-*N*-tosylbenzenesulfonimidoyl chloride (1c)

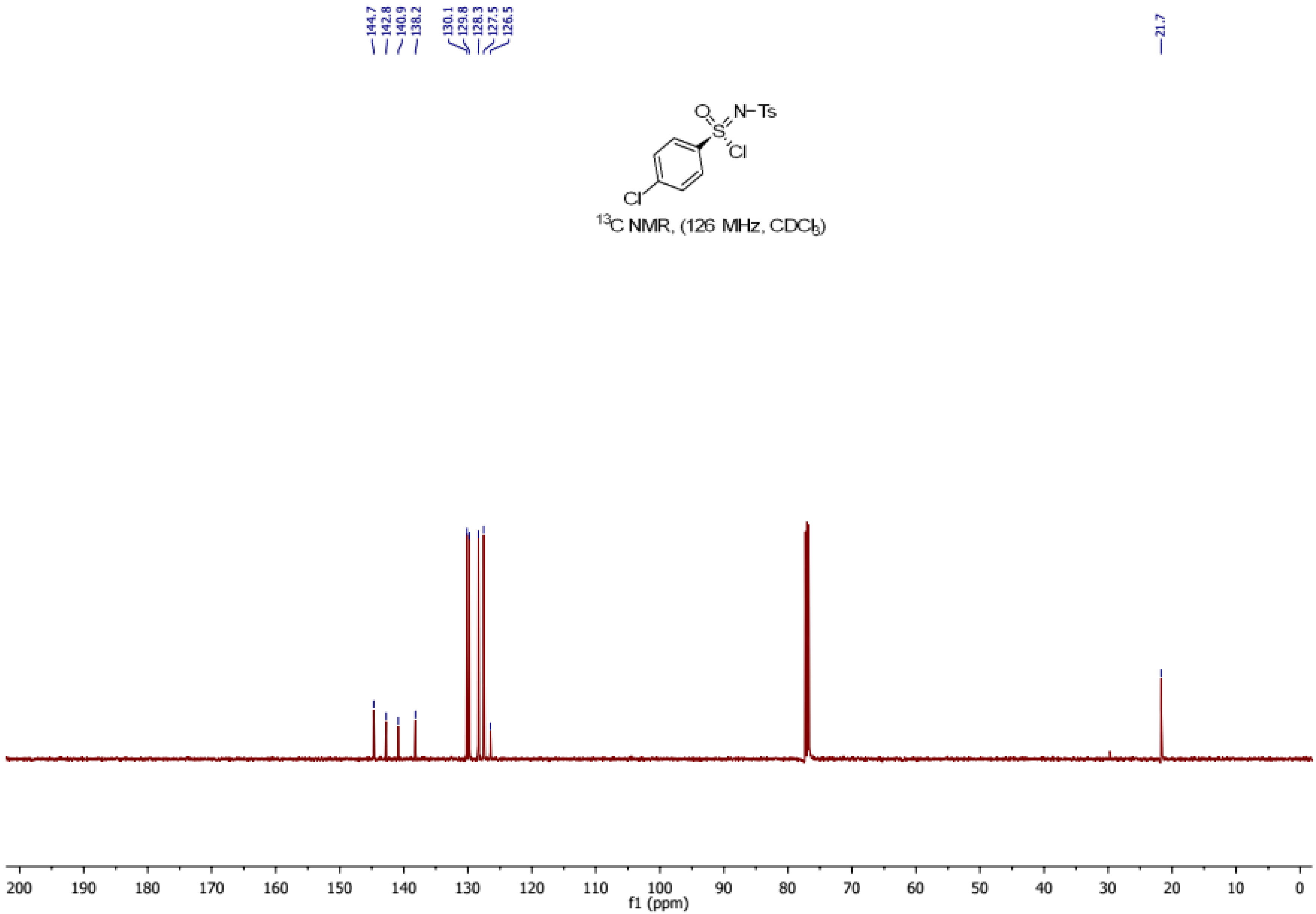

Go to table of contents

(*S*)-4-Bromo-*N*-tosylbenzenesulfonimidoyl chloride (1d)

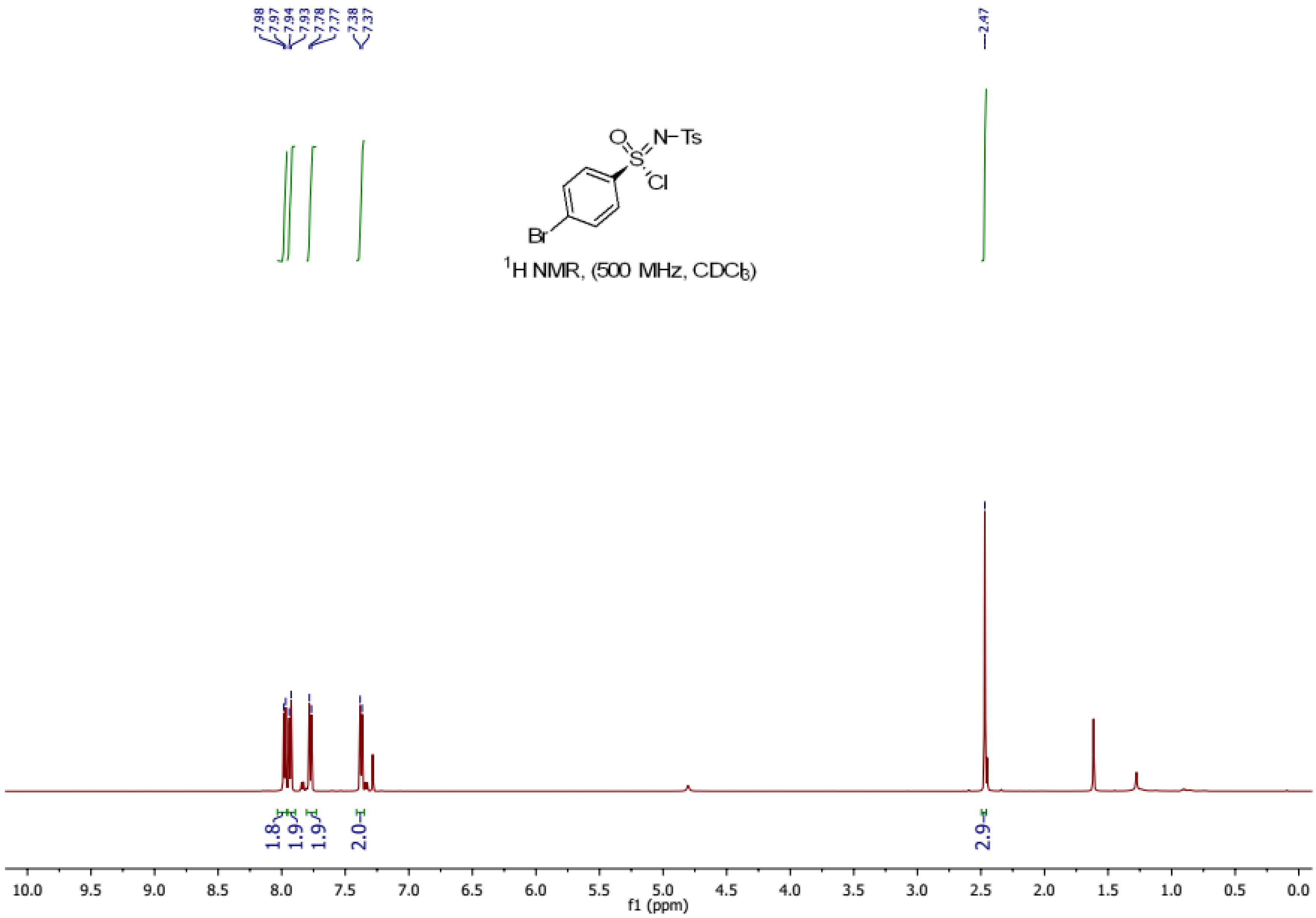

Go to table of contents

(*S*)-4-Bromo-*N*-tosylbenzenesulfonimidoyl chloride (1d)

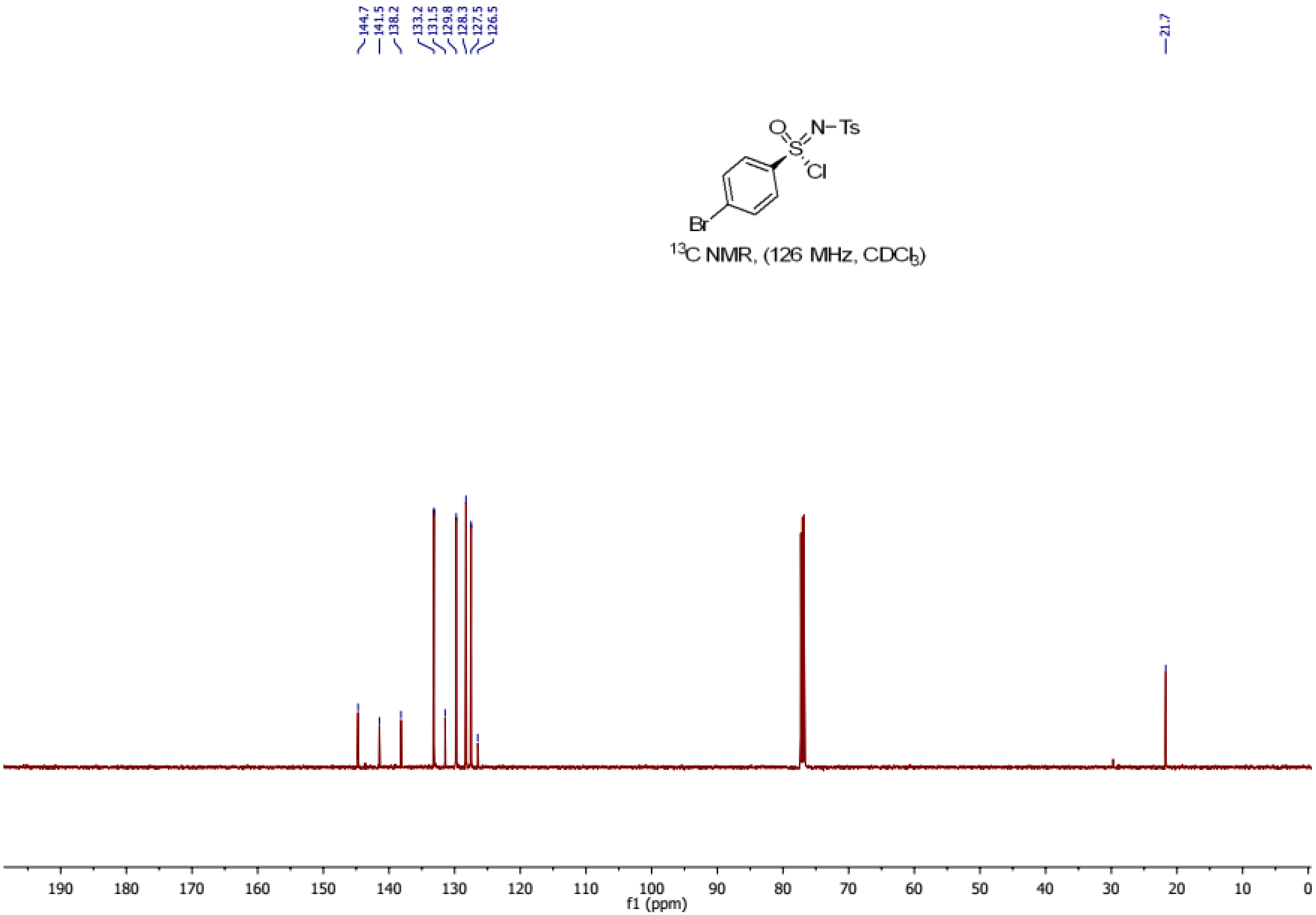

Go to table of contents

(*S*)-4-Iodo-*N*-tosylbenzenesulfonimidoyl chloride (1e)

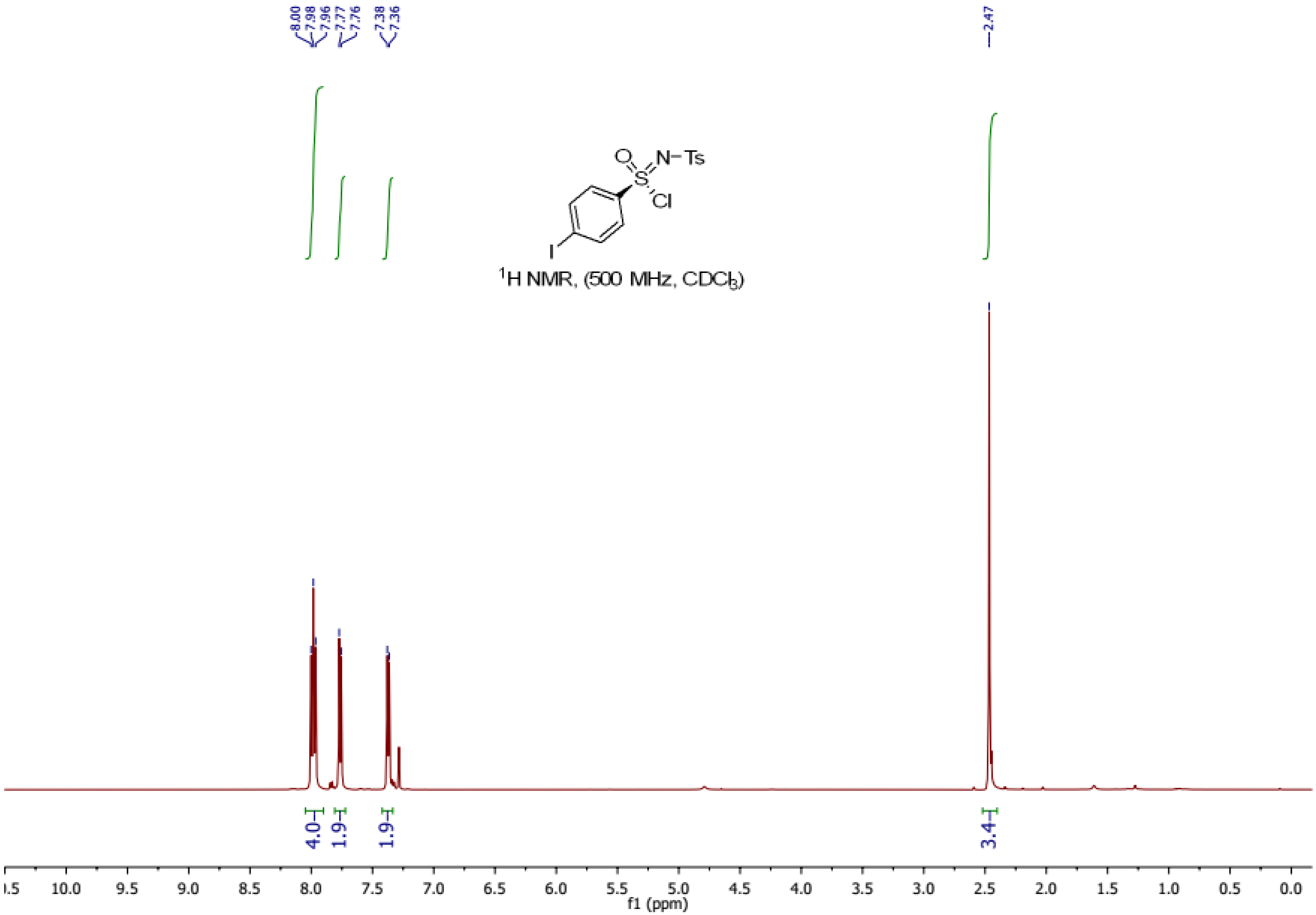

Go to table of contents

(*S*)-4-Iodo-*N*-tosylbenzenesulfonimidoyl chloride (1e)

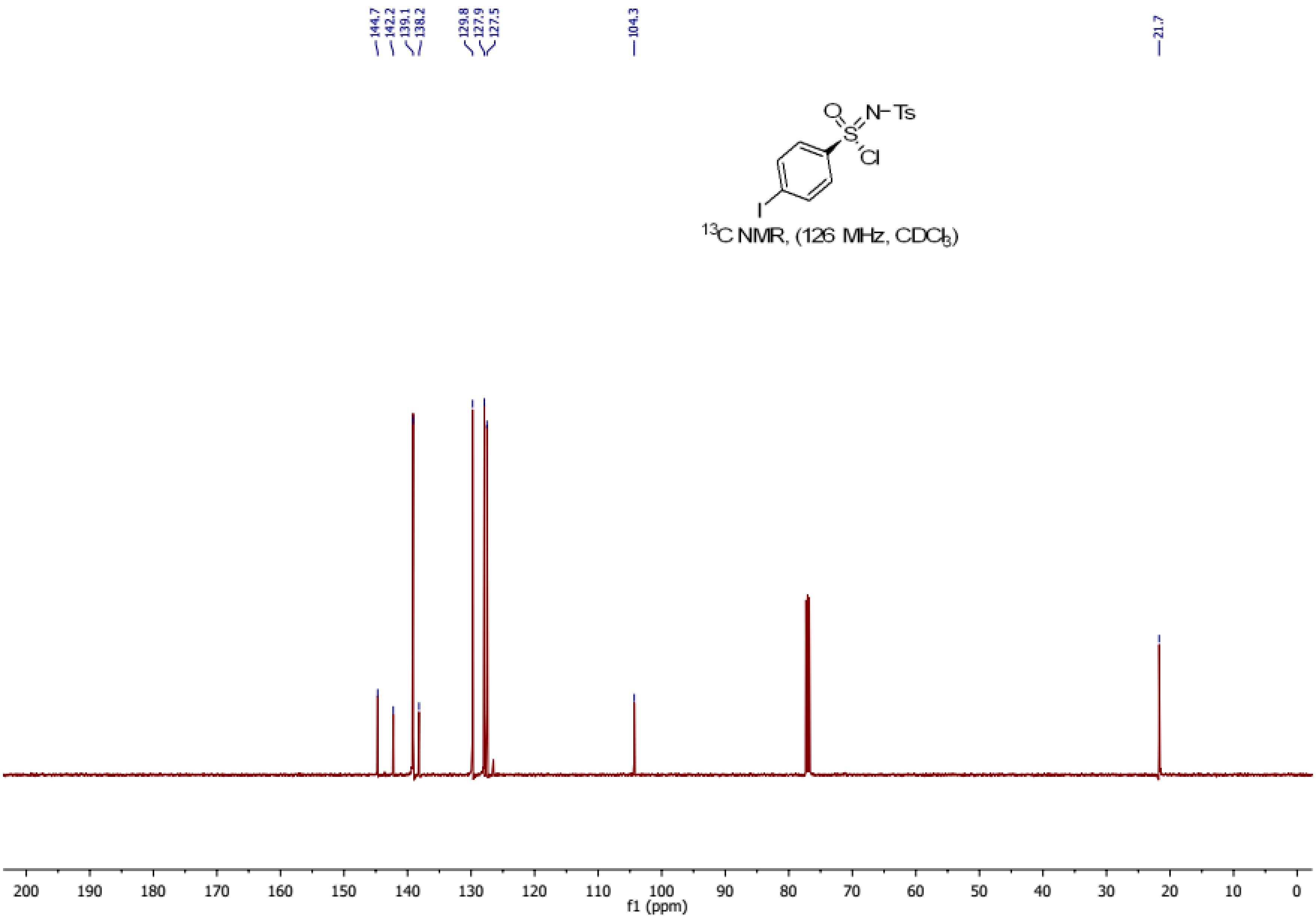

Go to table of contents

(*S*)-4-Nitro-*N*-tosylbenzenesulfonimidoyl chloride (1f)

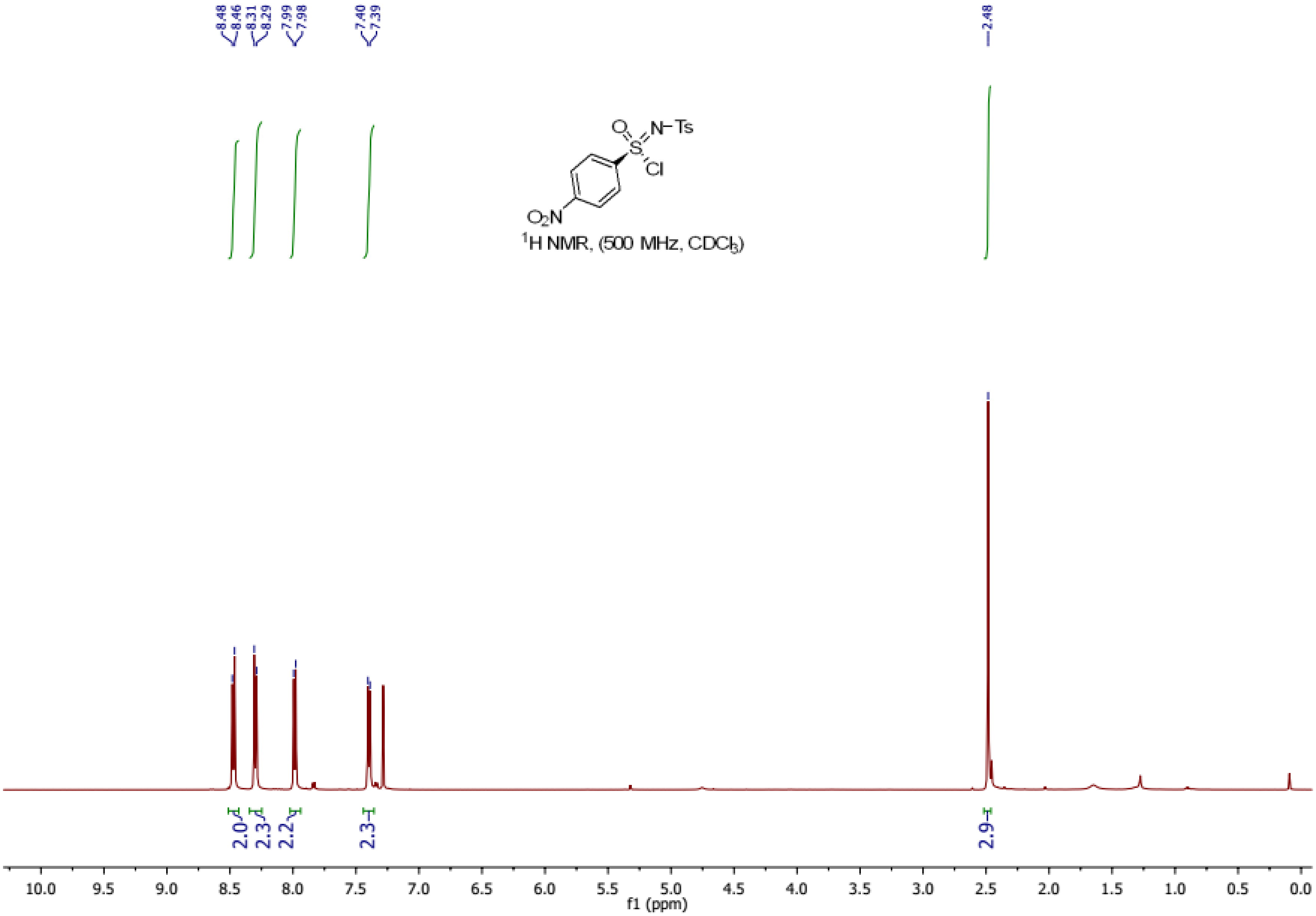

(*S*)-4-Nitro-*N*-tosylbenzenesulfonimidoyl chloride (1f)

Go to table of contents

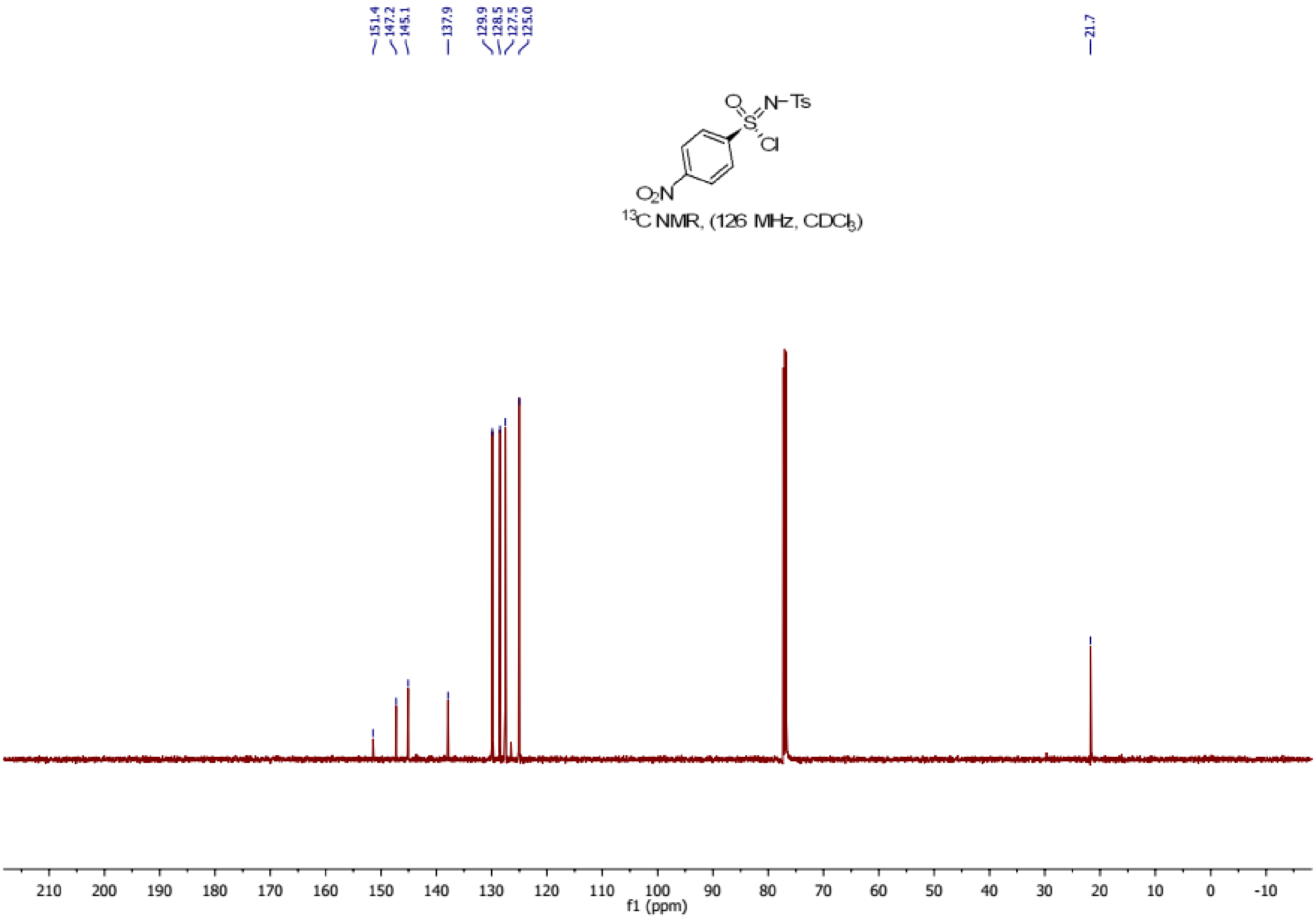

Go to table of contents

(*S*)-4-Methoxy-*N*-tosylbenzenesulfonimidoyl chloride (1g)

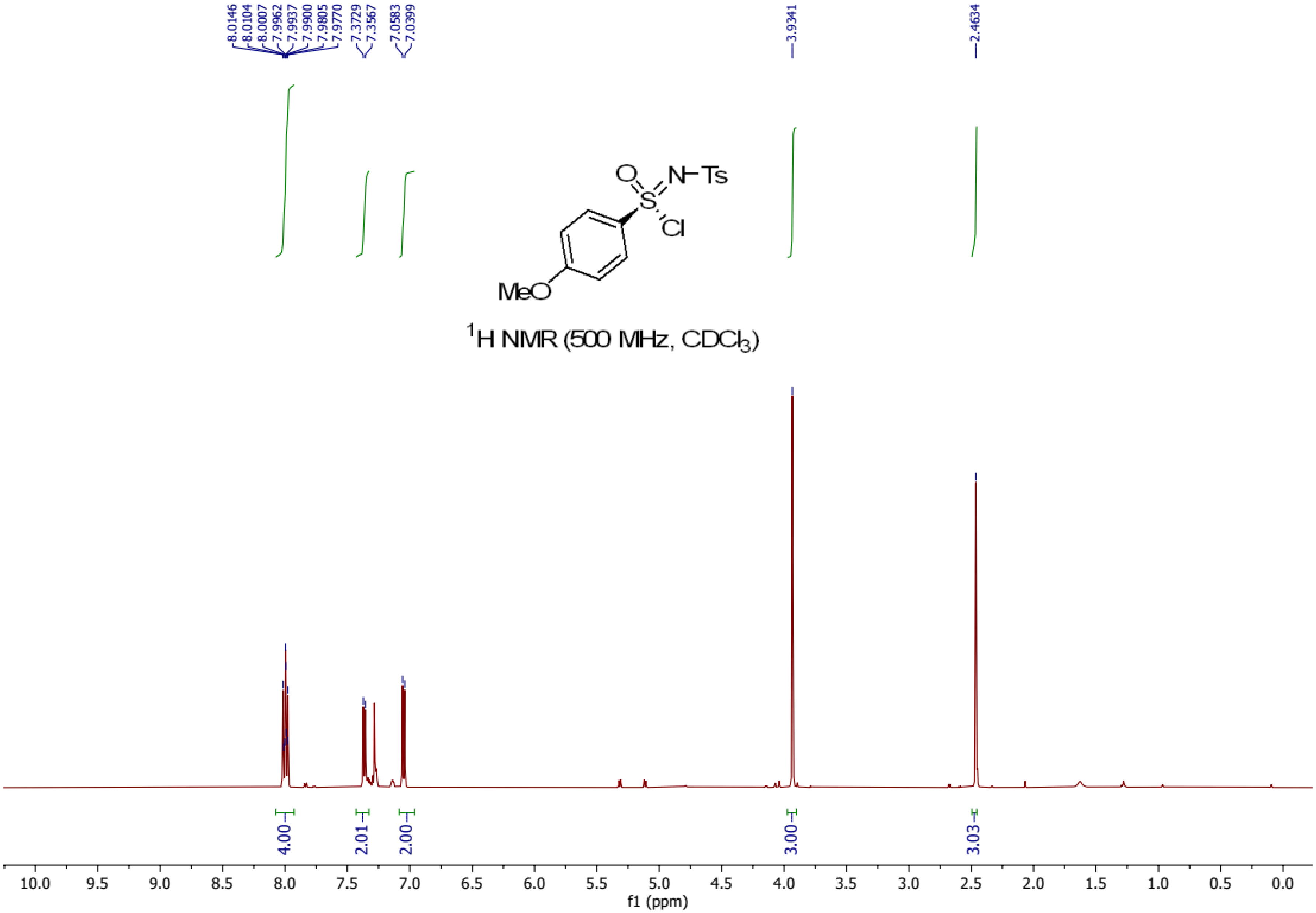

Go to table of contents

(*S*)-4-Methoxy-*N*-tosylbenzenesulfonimidoyl chloride (1g)

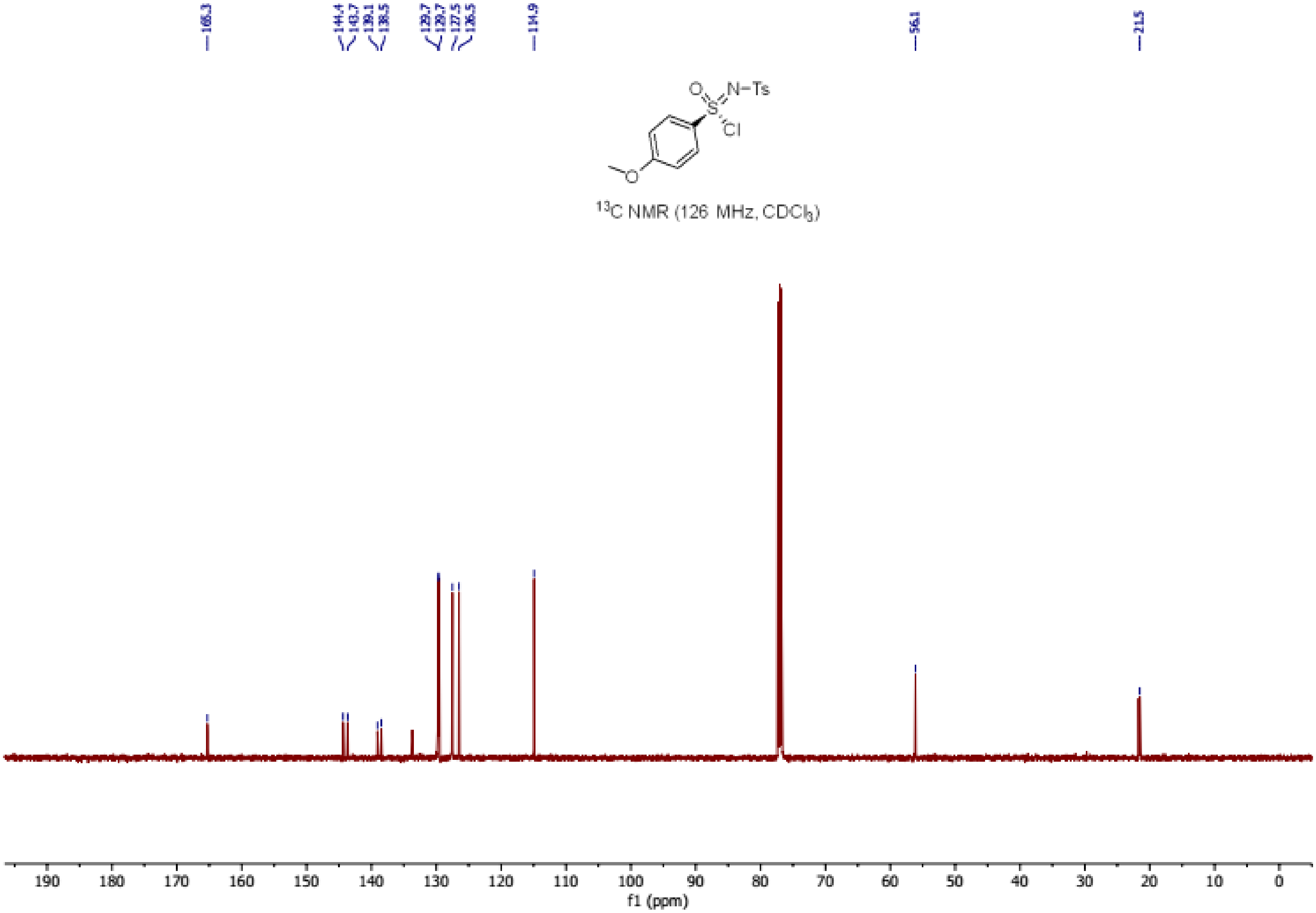

Go to table of contents

(*S*)-4-(Difluoromethoxy)-*N*-tosylbenzenesulfonimidoyl chloride (1h)

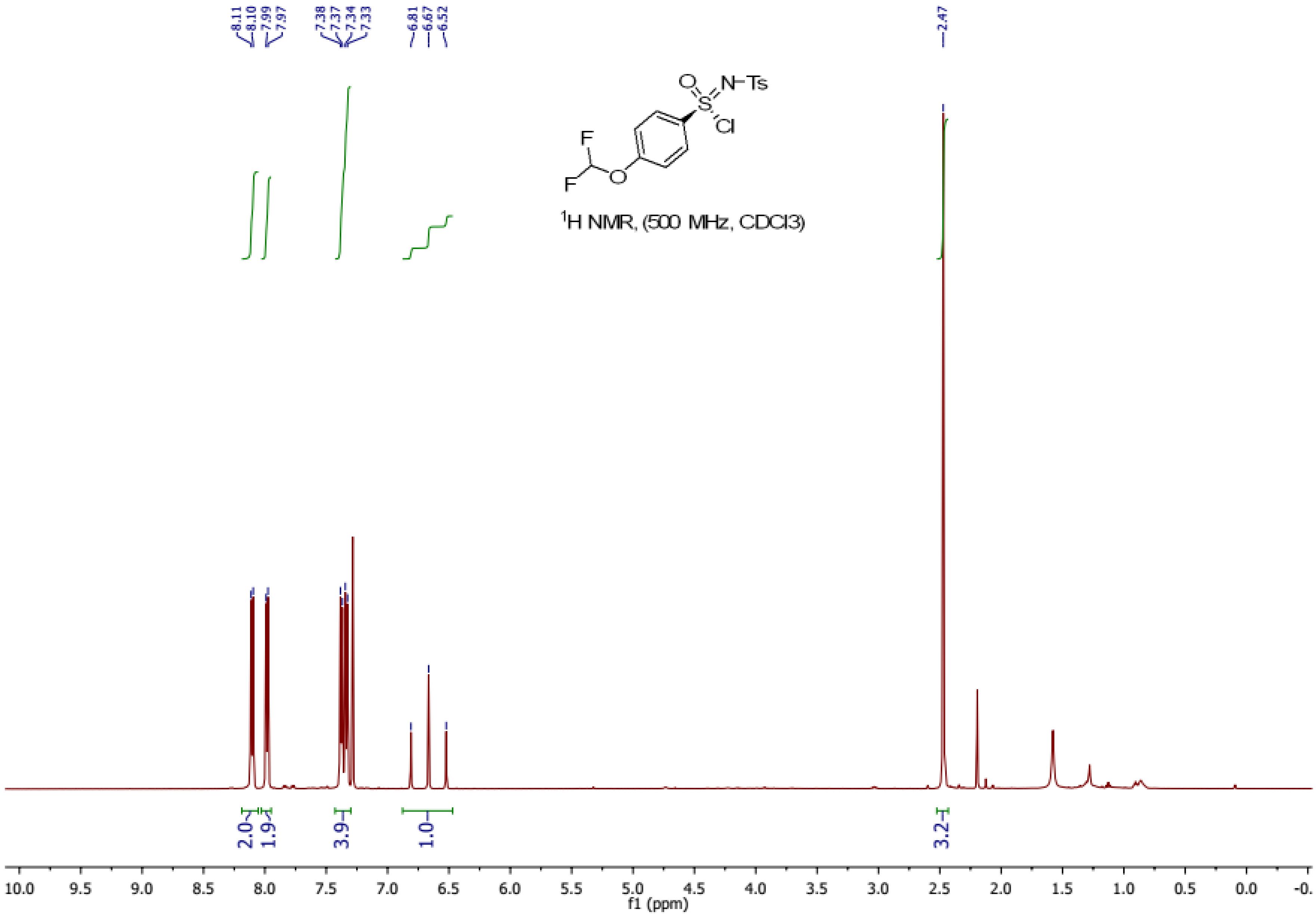

Go to table of contents

(*S*)-4-(Difluoromethoxy)-*N*-tosylbenzenesulfonimidoyl chloride (1h)

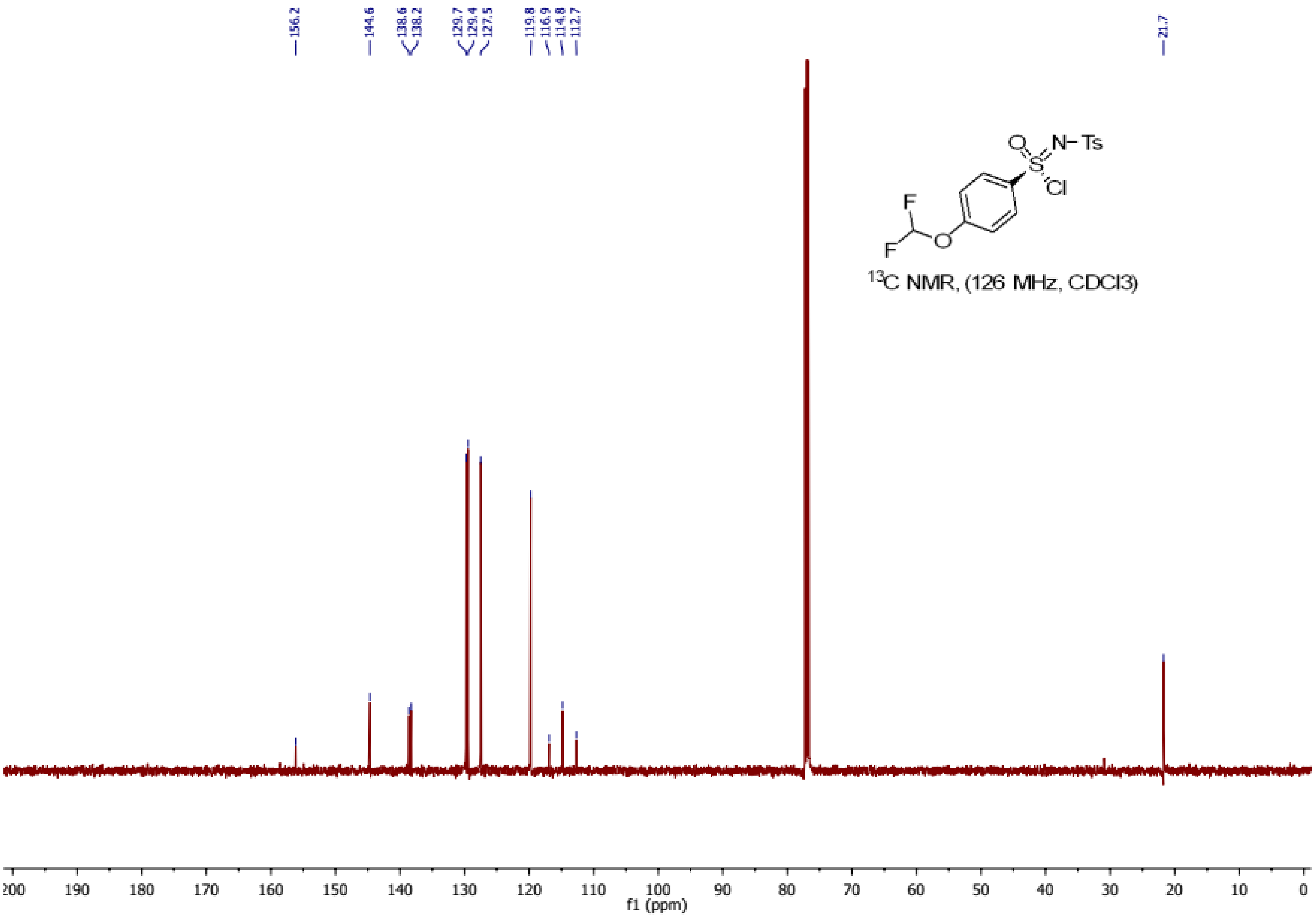

Go to table of contents

(*S*)-*N*-Tosyl-3-(trifluoromethoxy)benzenesulfonimidoyl chloride (1i)

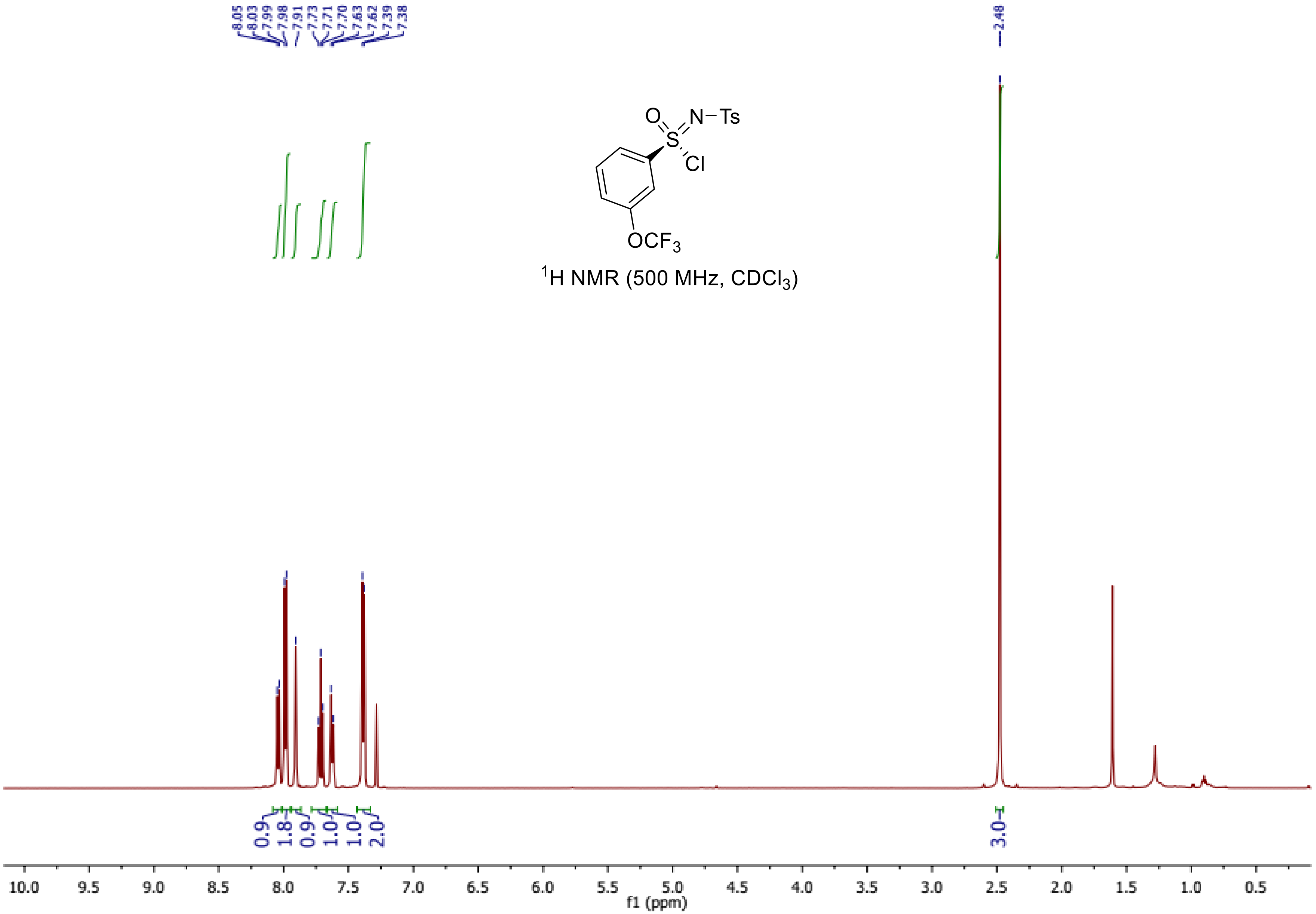

Go to table of contents

(*S*)-*N*-Tosyl-3-(trifluoromethoxy)benzenesulfonimidoyl chloride (1i)

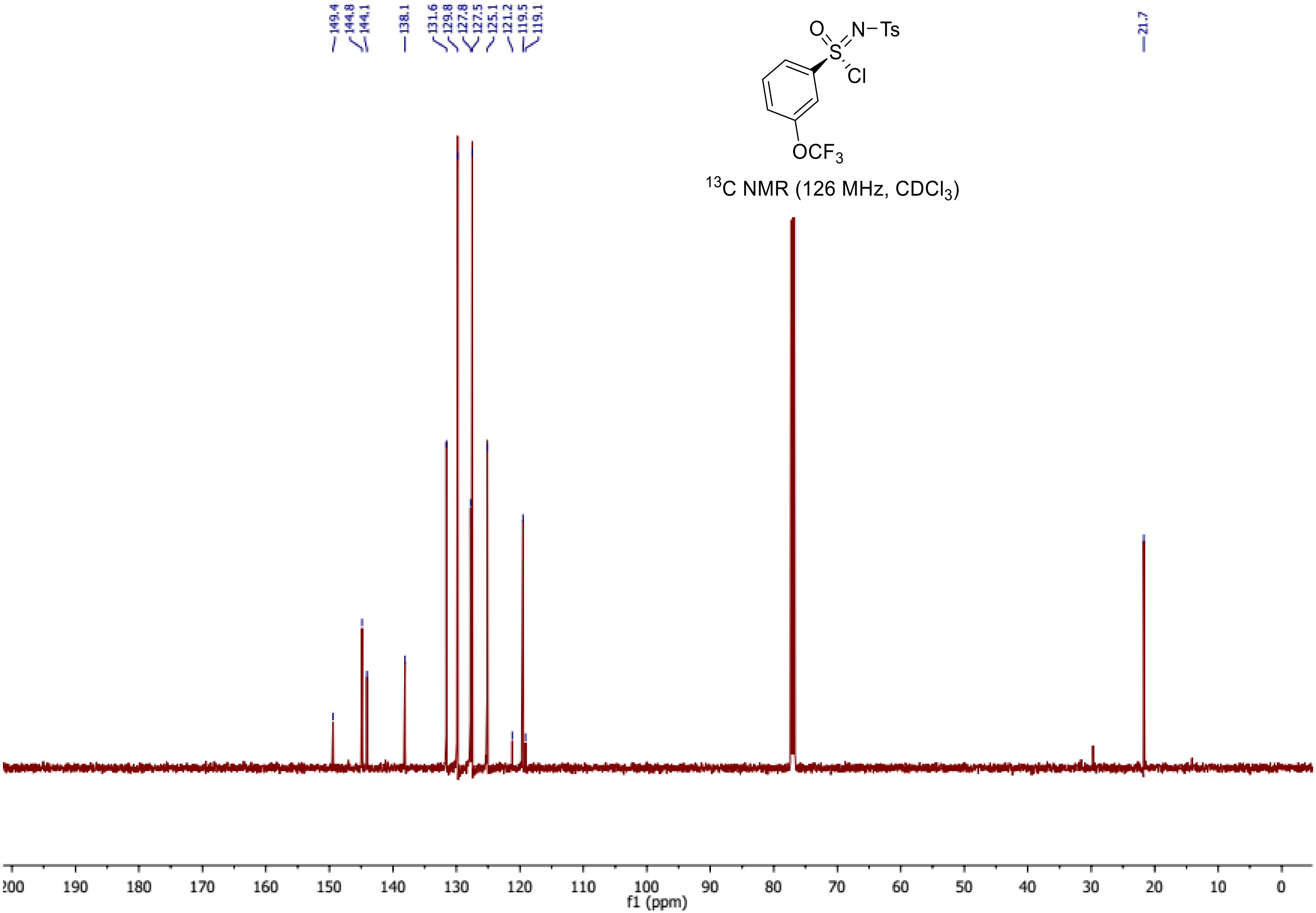

Go to table of contents

(*S*)-3-(Methylsulfonyl)-*N*-tosylbenzenesulfonimidoyl chloride (1j)

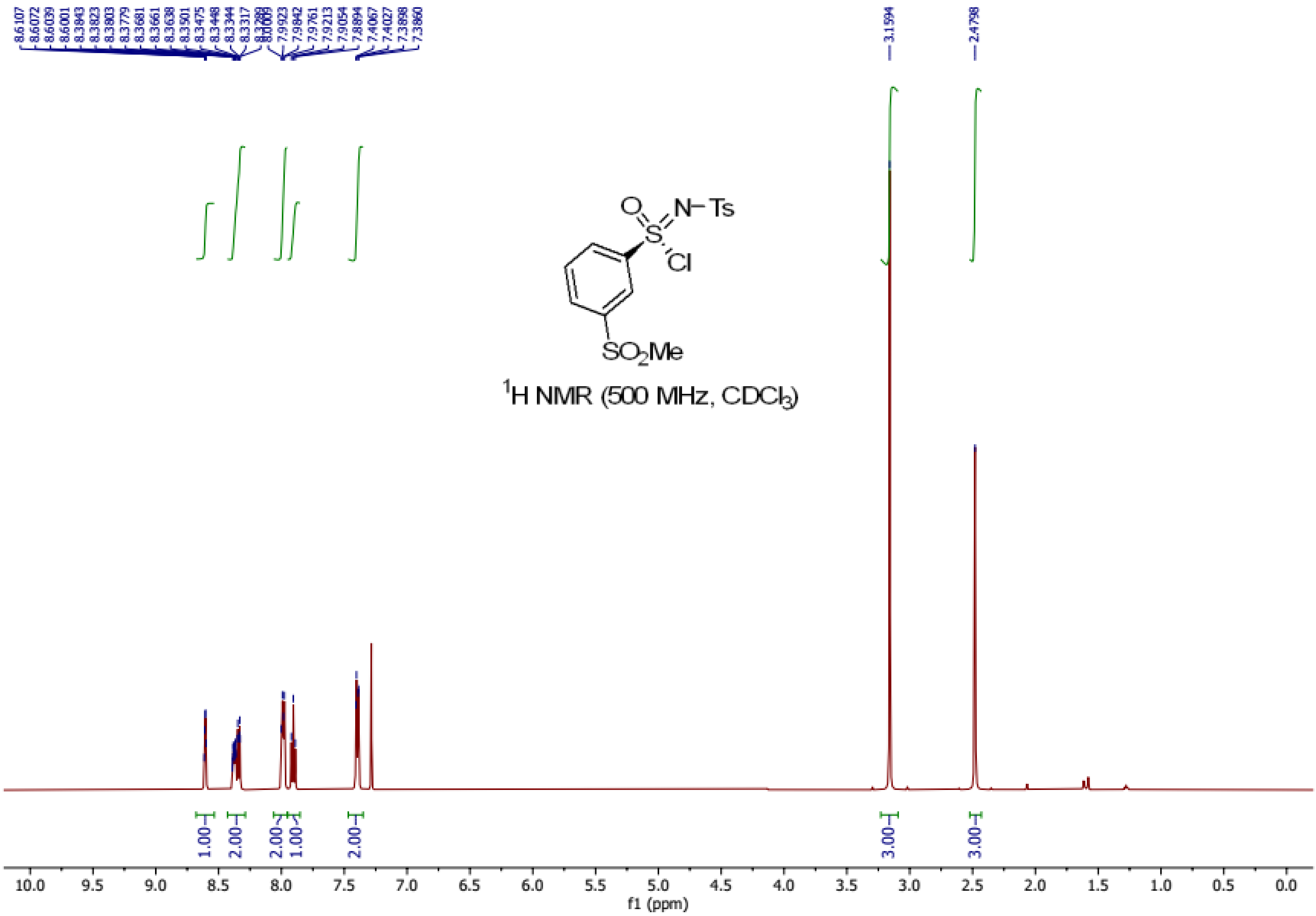

Go to table of contents

(*S*)-3-(Methylsulfonyl)-*N*-tosylbenzenesulfonimidoyl chloride (1j)

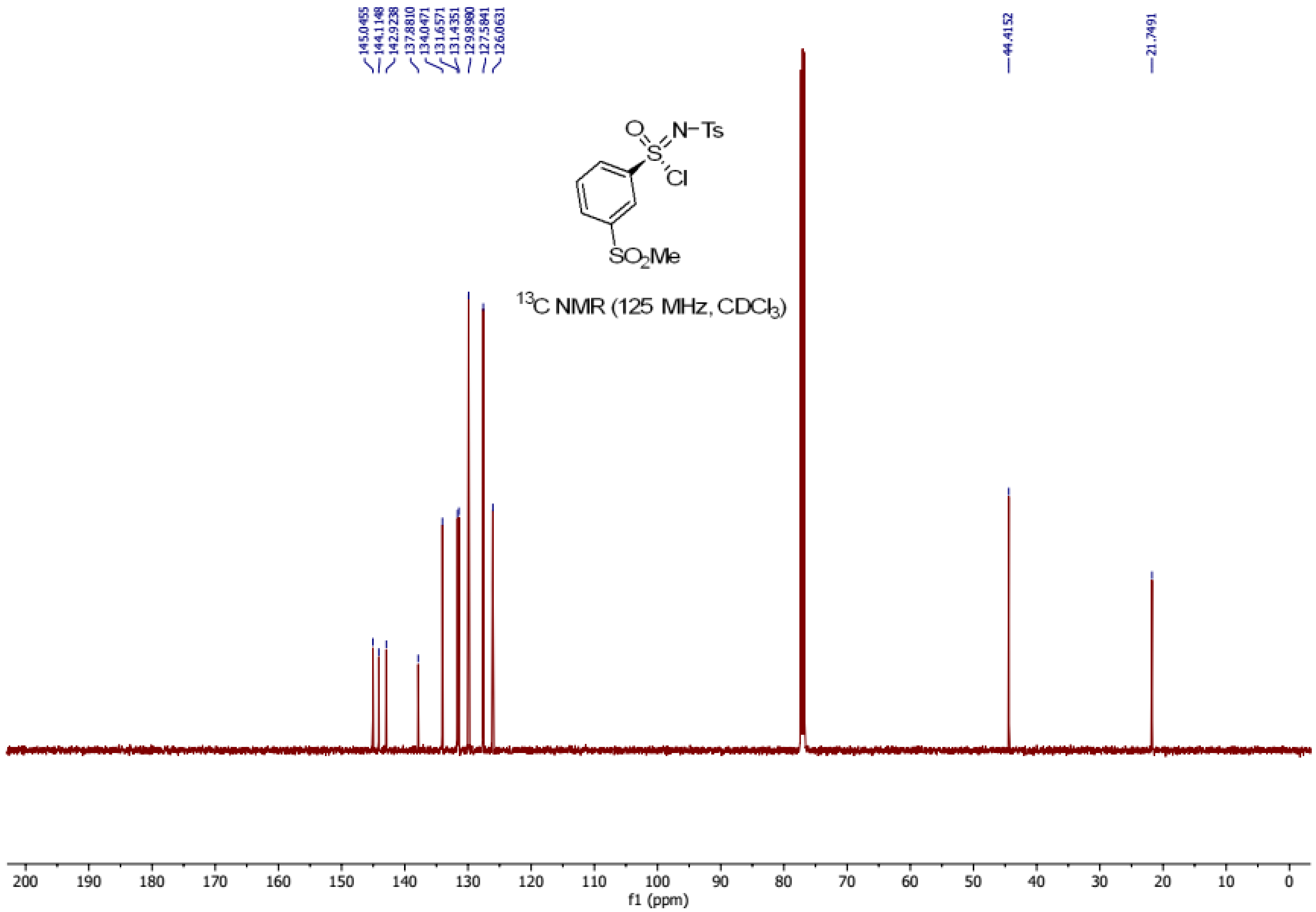

(*S*)-4-Isocyano-*N*-tosylbenzenesulfonimidoyl chloride (1k)

Go to table of contents

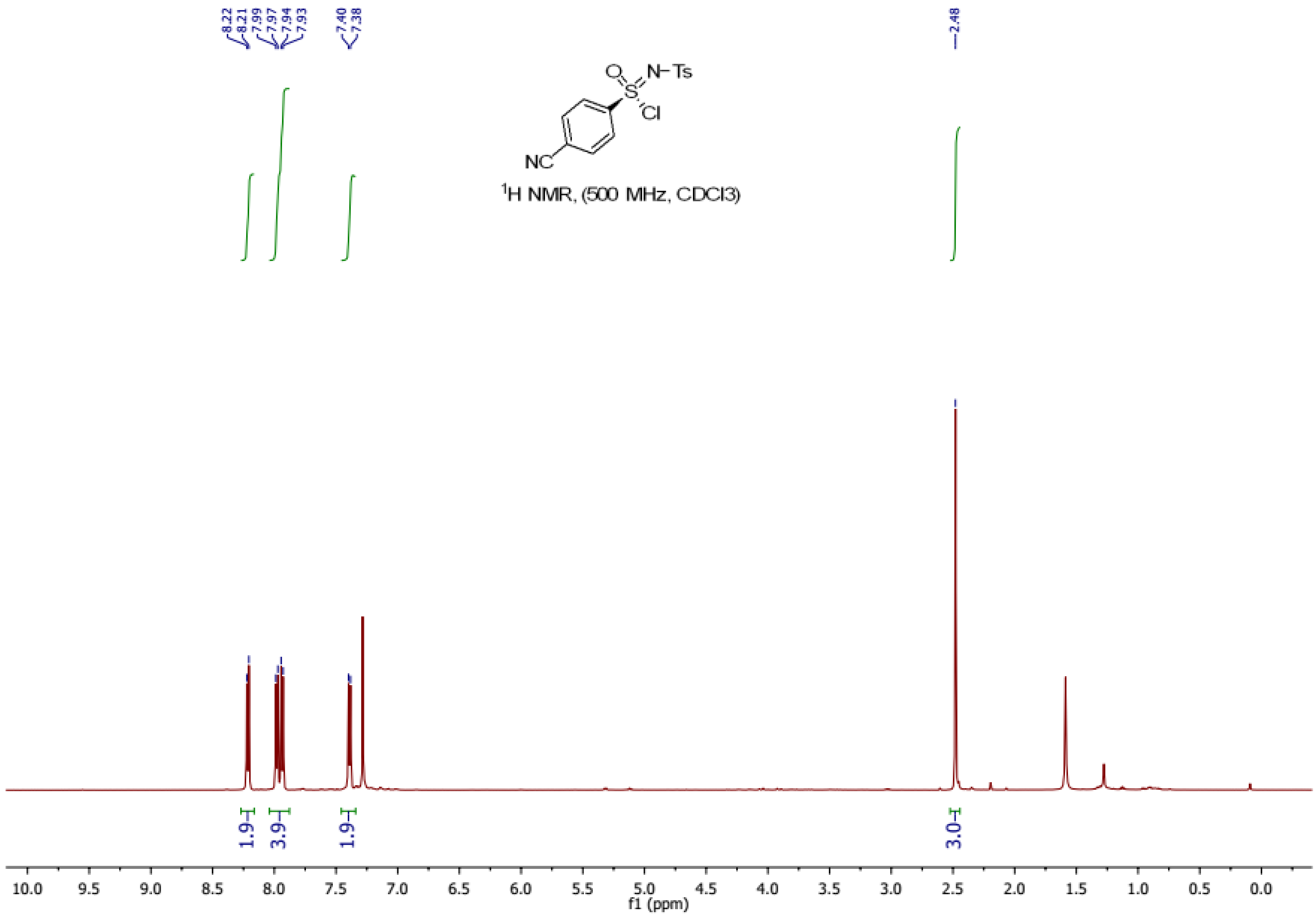

Go to table of contents

(*S*)-4-Isocyano-*N*-tosylbenzenesulfonimidoyl chloride (1k)

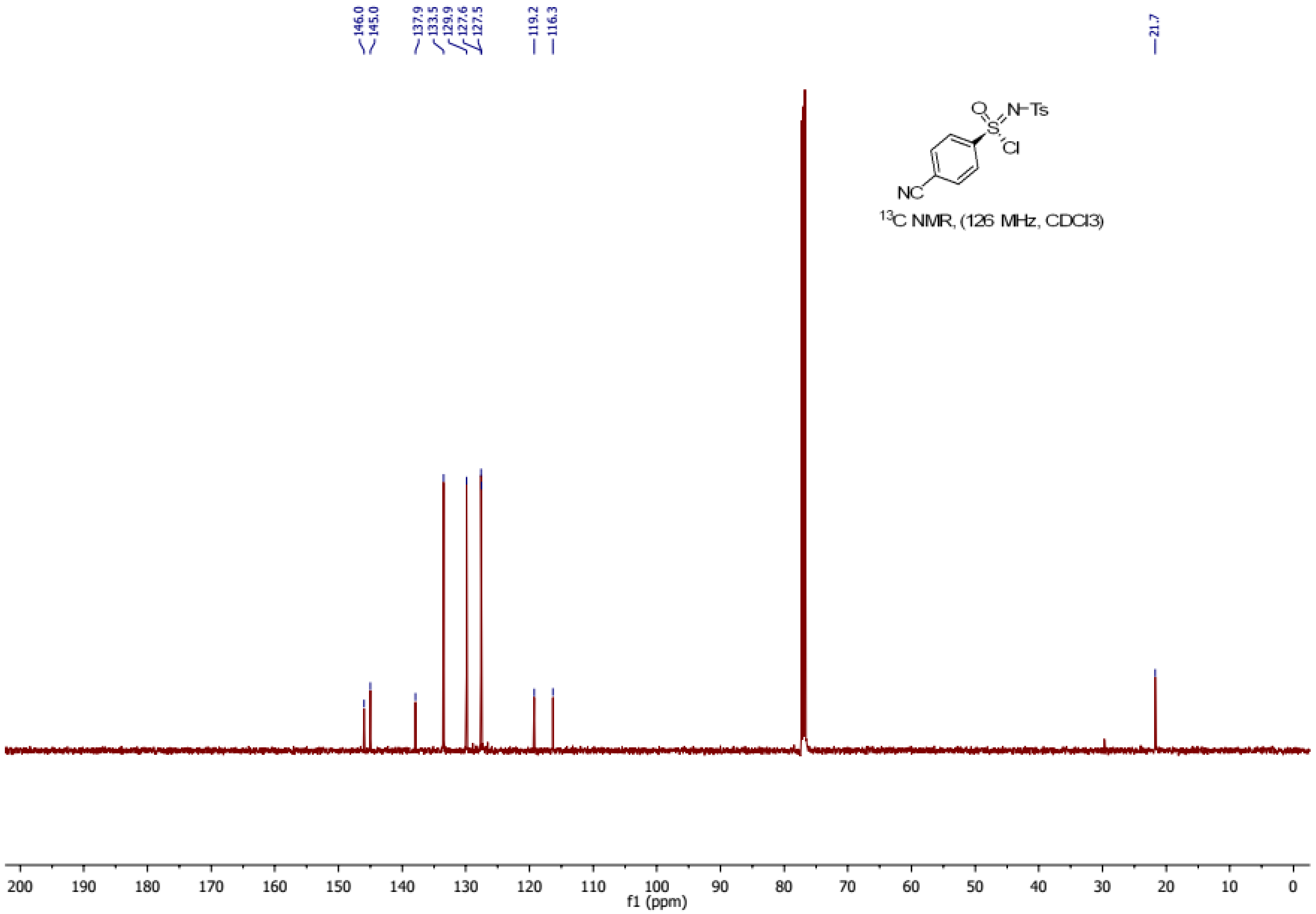

Go to table of contents

(*S*)-4-Methyl-*N*-tosylbenzenesulfonimidoyl chloride (1l)

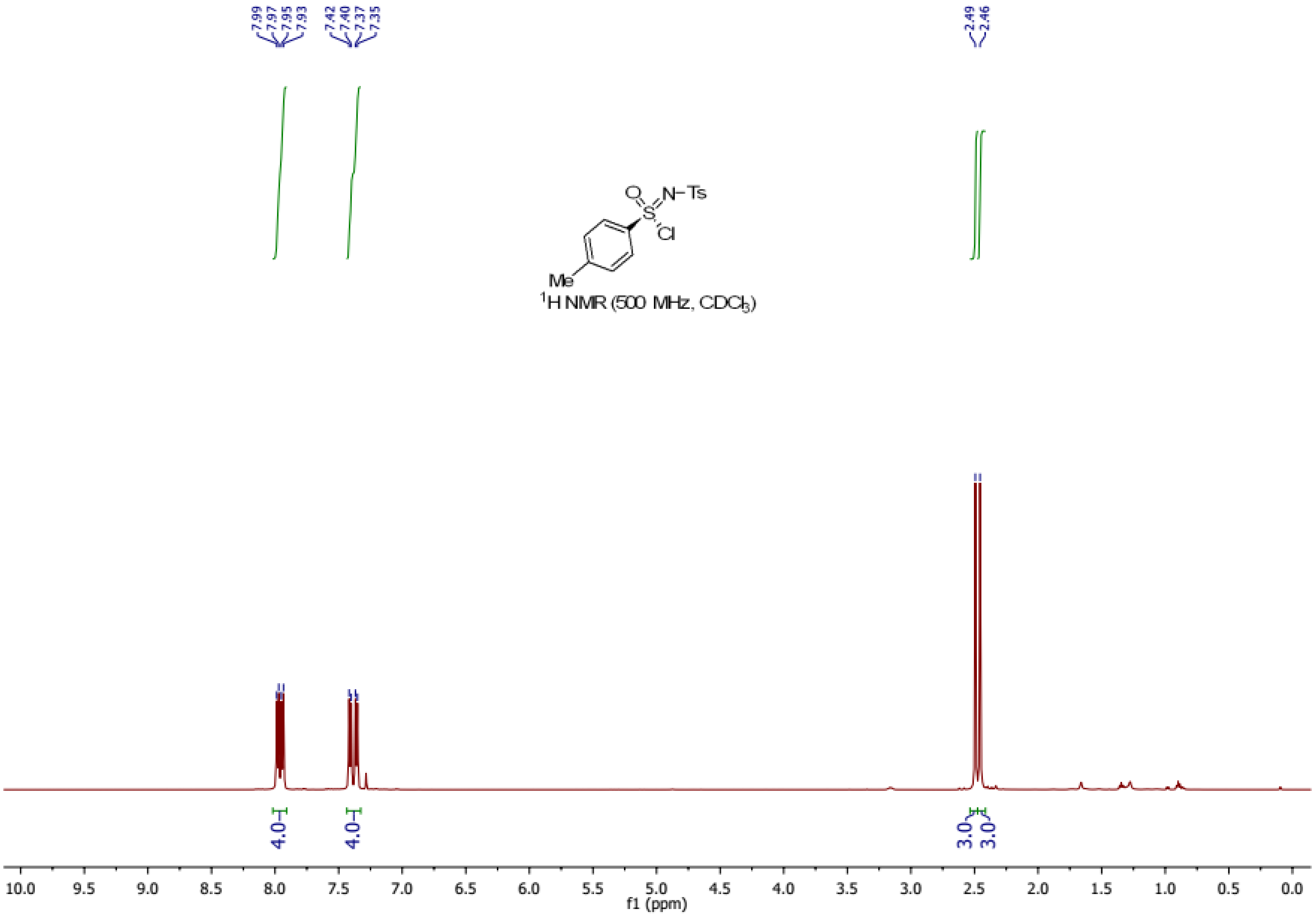

Go to table of contents

(*S*)-4-Methyl-*N*-tosylbenzenesulfonimidoyl chloride (1l)

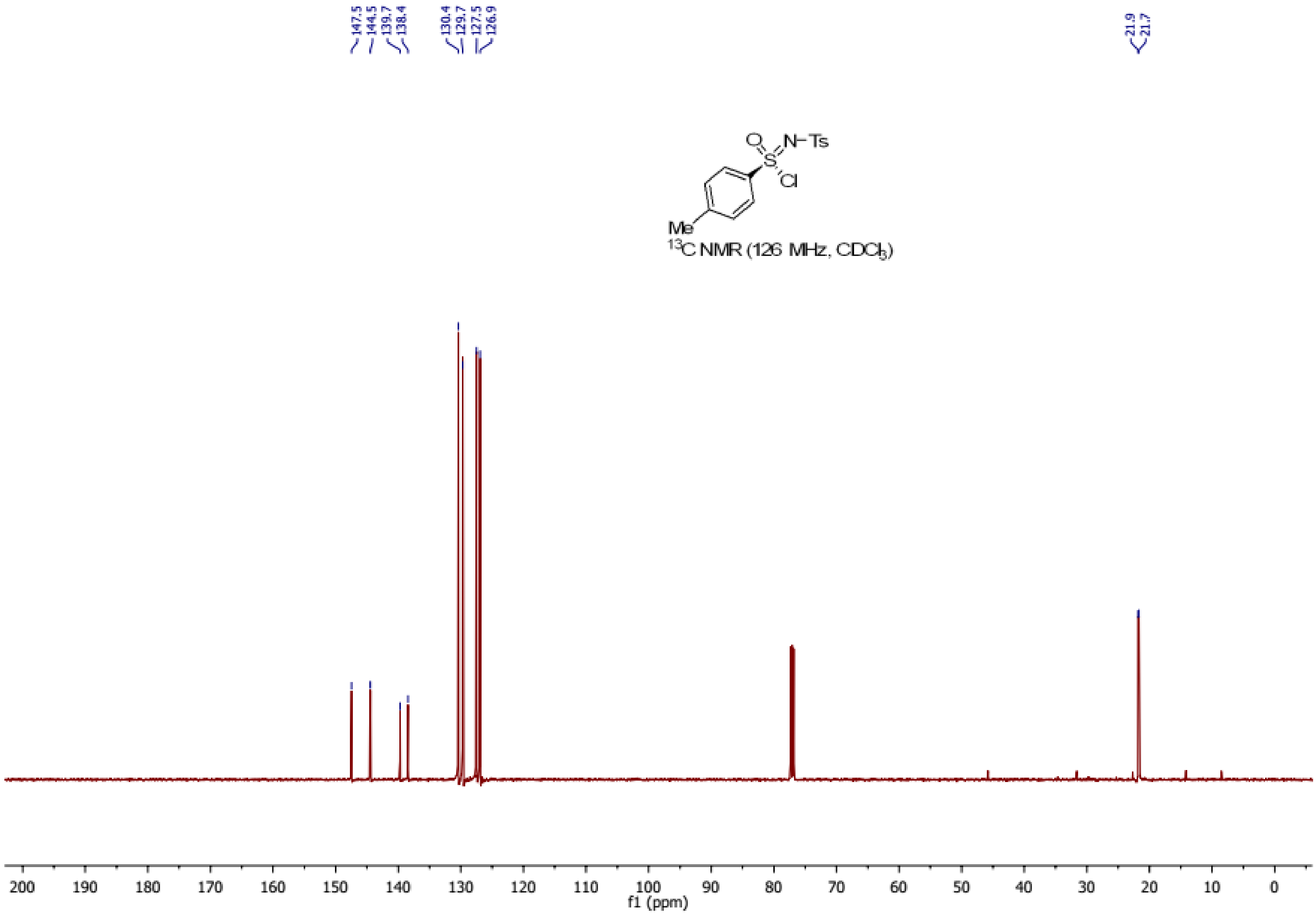

Go to table of contents

(*S*)-4-(*tert*-butyl)-*N*-tosylbenzenesulfonimidoyl chloride (1m)

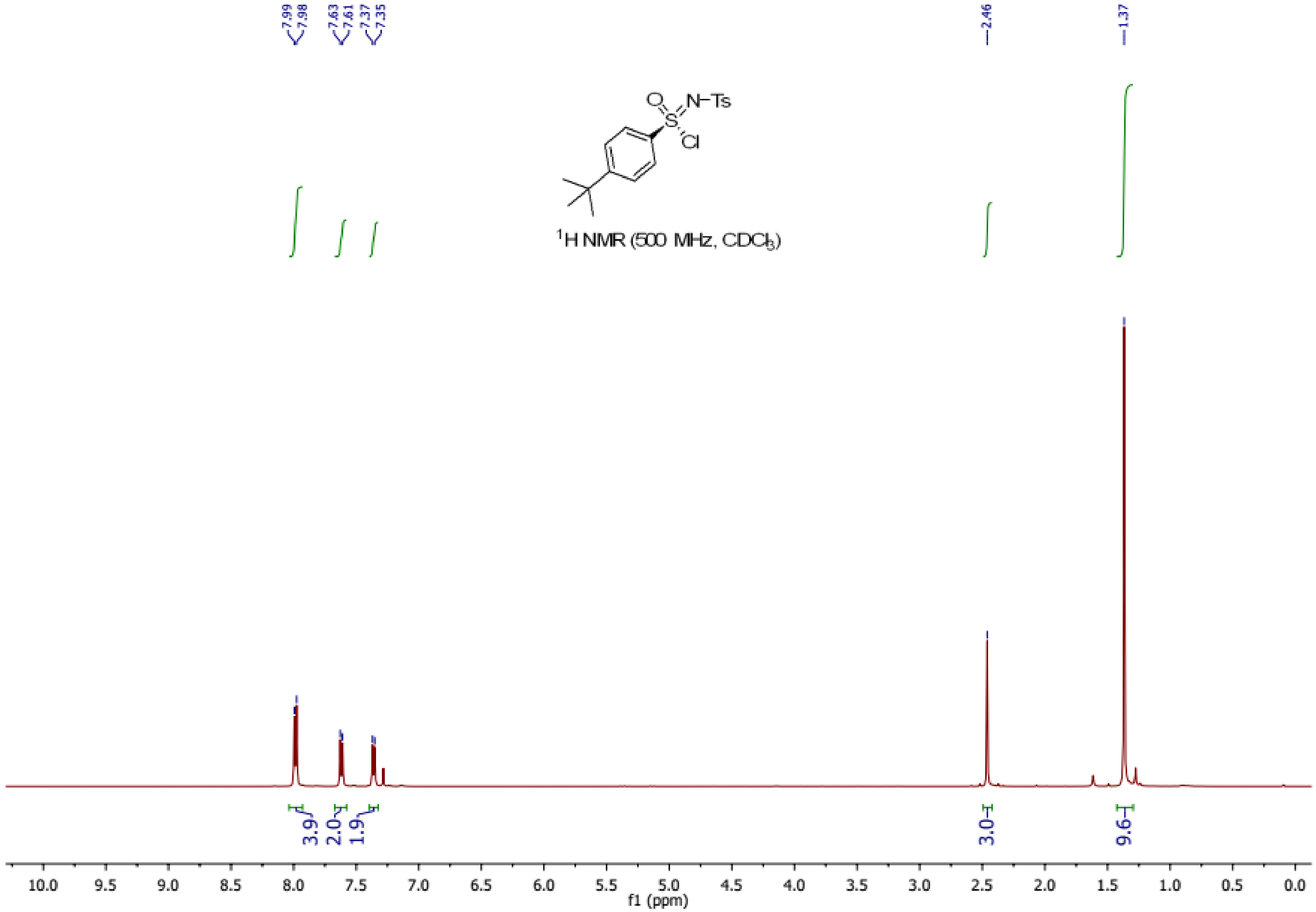

Go to table of contents

(*S*)-4-(*tert*-butyl)-*N*-tosylbenzenesulfonimidoyl chloride (1m)

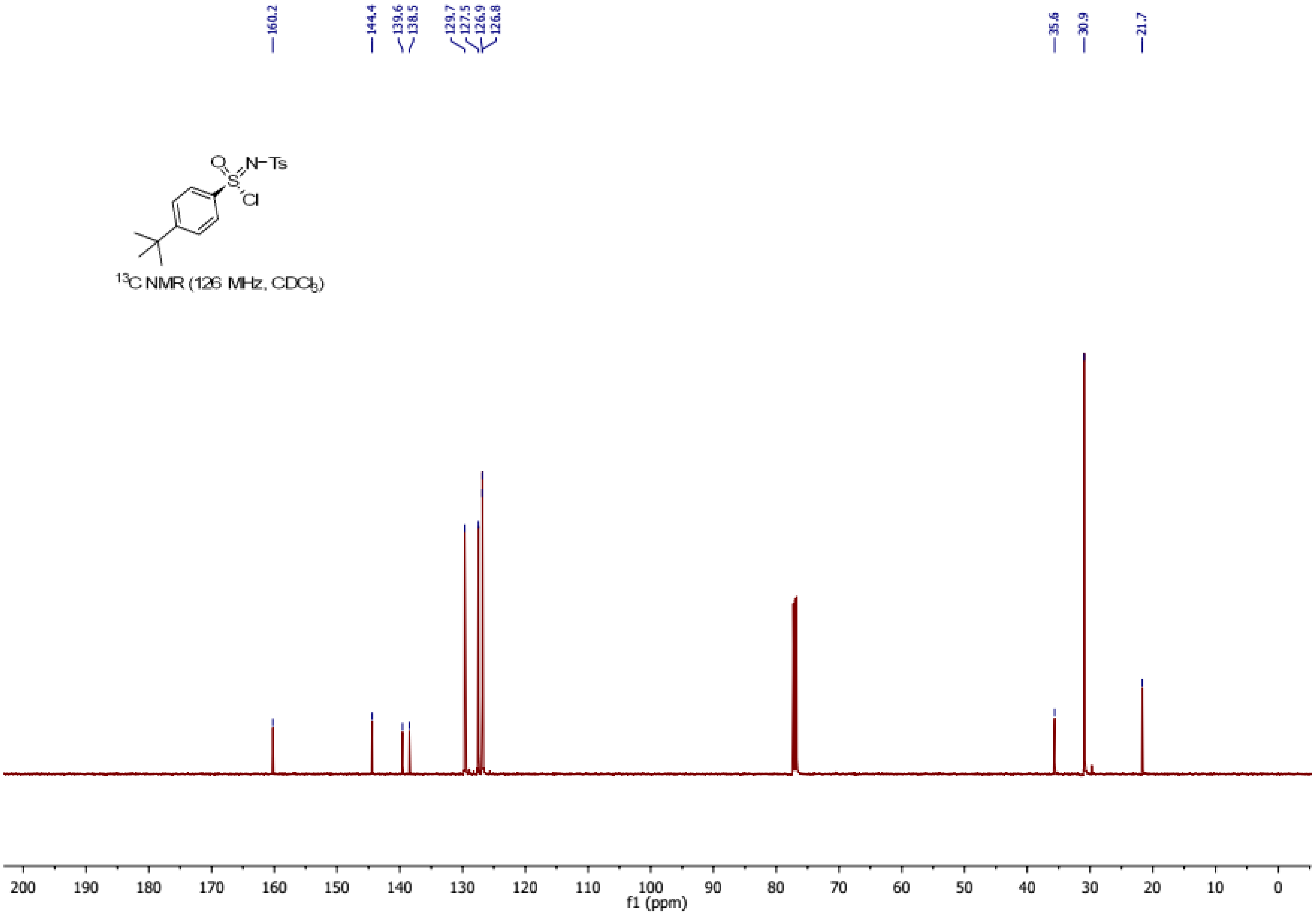

Go to table of contents

(*S*)-*N*-Tosyl-[1,1'-biphenyl]-4-sulfonimidoyl chloride (1n)

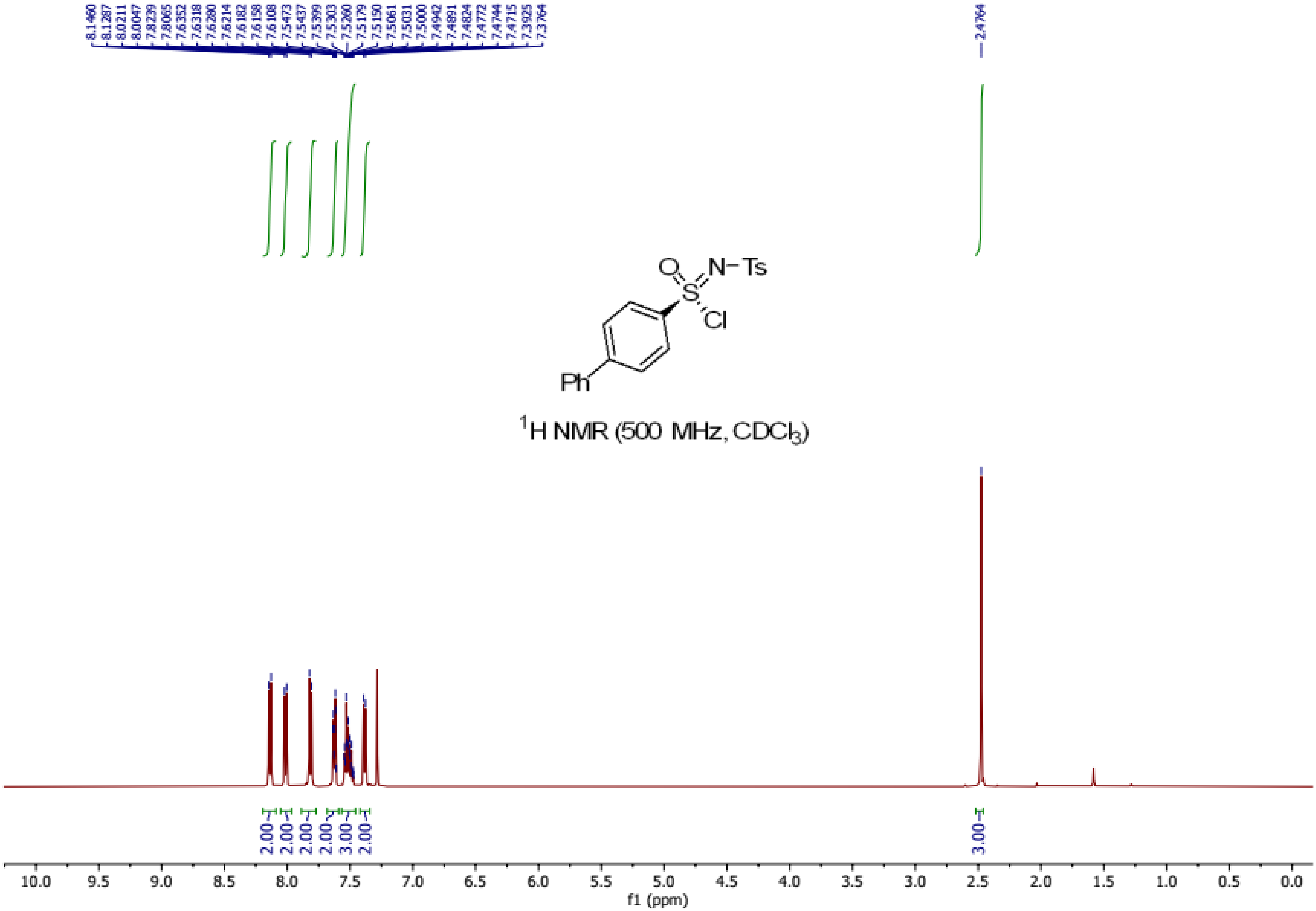

Go to table of contents

(*S*)-*N*-Tosyl-[1,1'-biphenyl]-4-sulfonimidoyl chloride (1n)

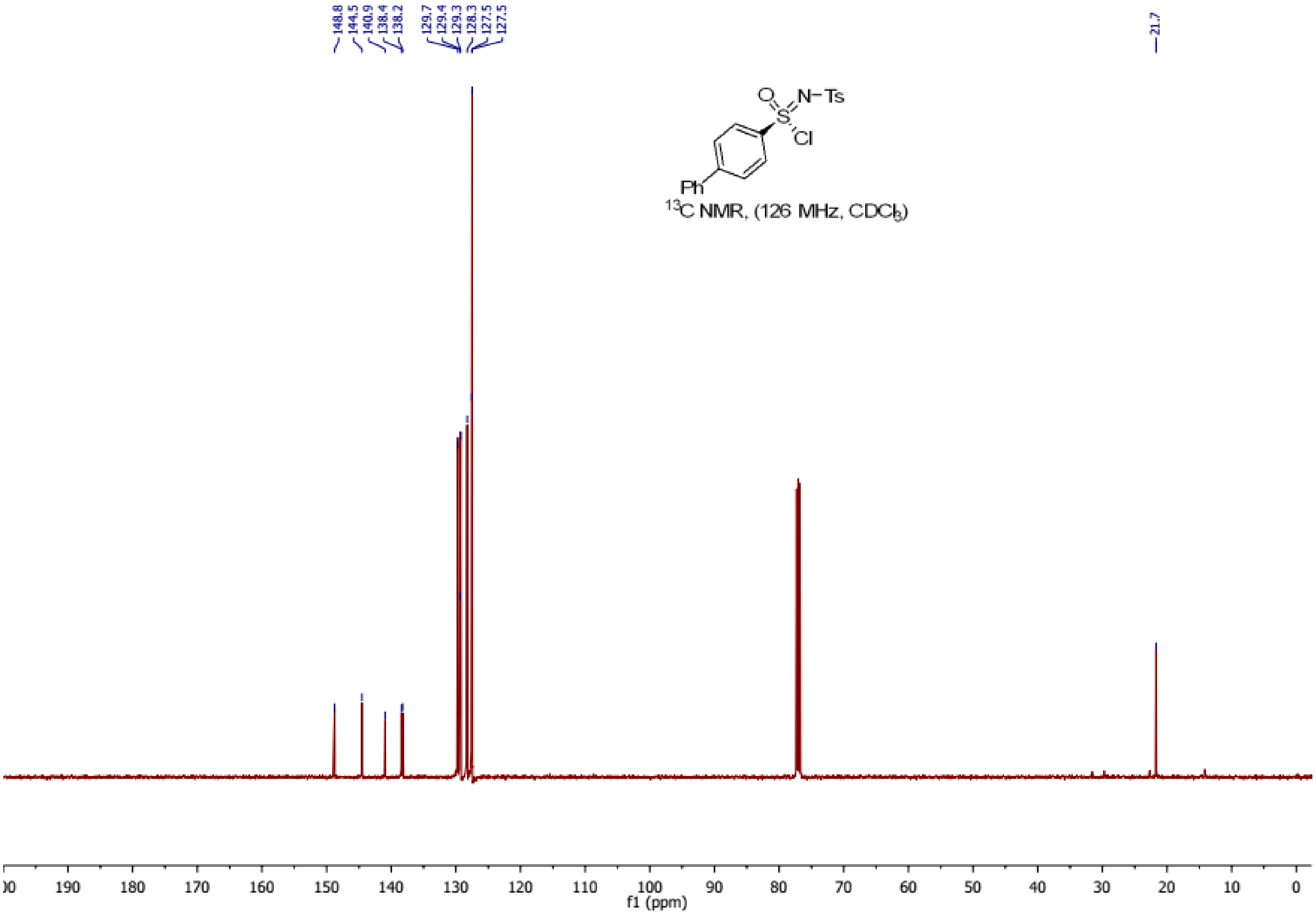

Go to table of contents

(*S*)-4'-Fluoro-*N*-tosyl-[1,1'-biphenyl]-4-sulfonimidoyl chloride (1o)

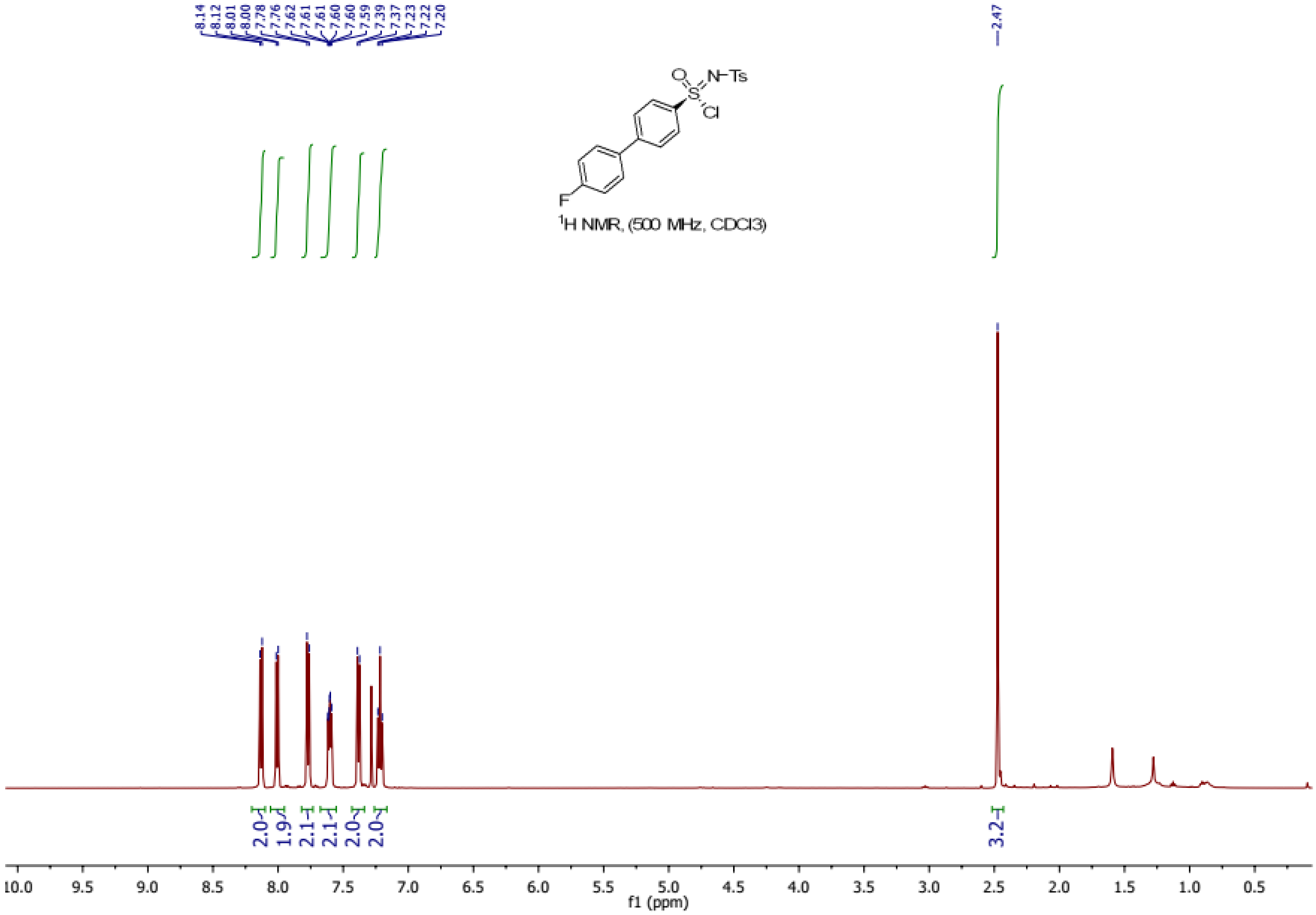

Go to table of contents

(*S*)-4'-Fluoro-*N*-tosyl-[1,1'-biphenyl]-4-sulfonimidoyl chloride (1o)

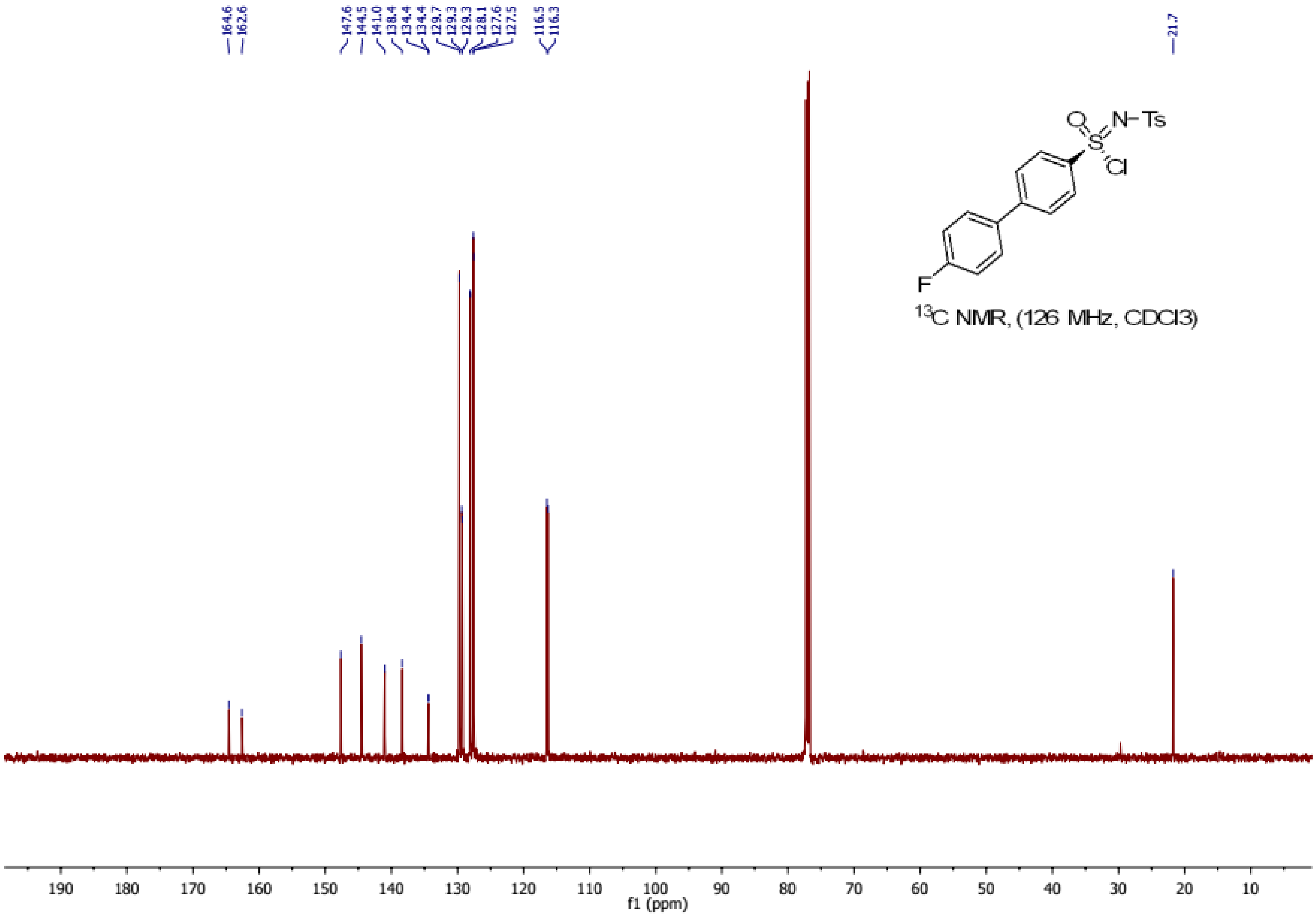

Go to table of contents

(*S*)-2,5-Difluoro-*N*-tosylbenzenesulfonimidoyl chloride (1p)

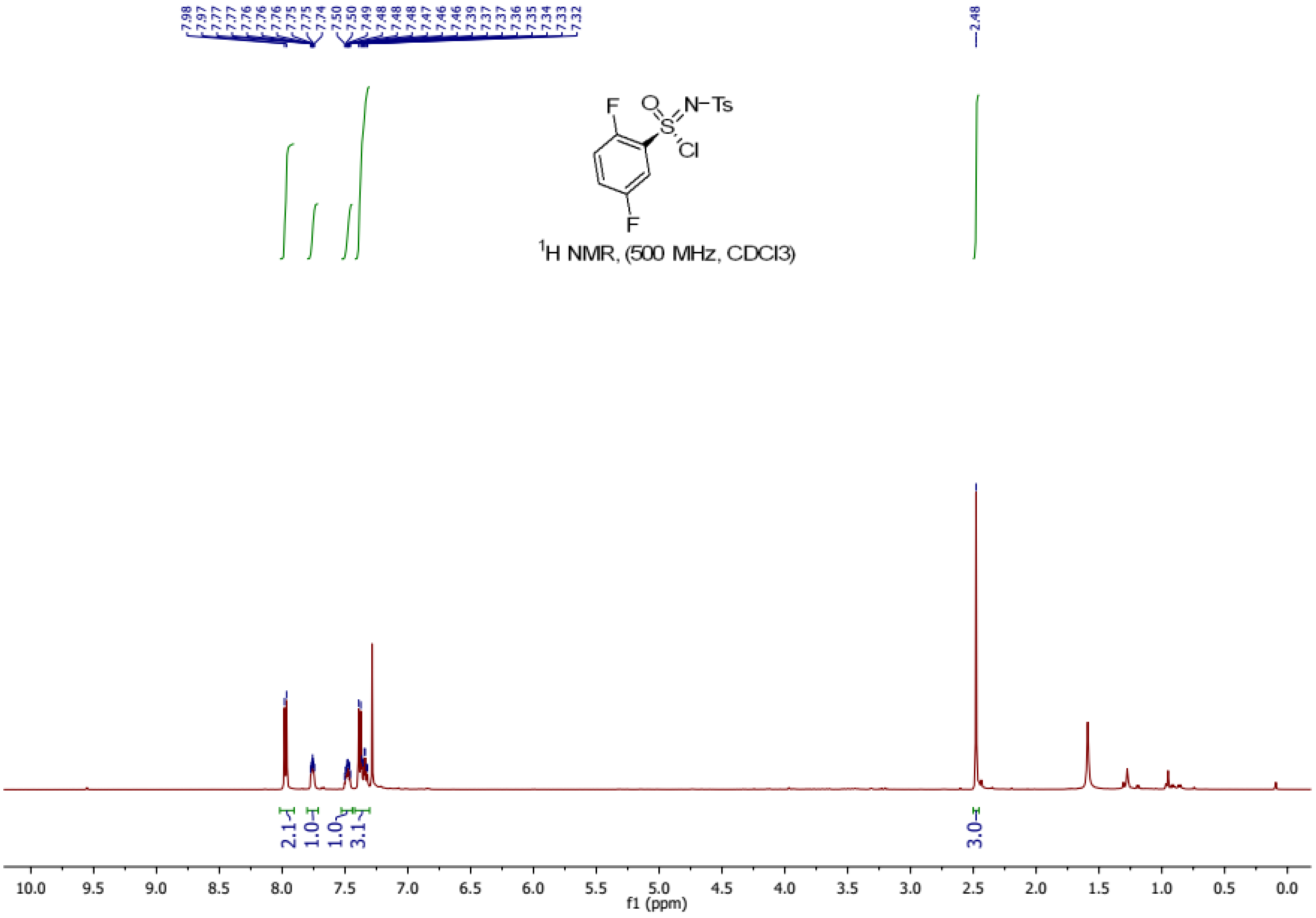

Go to table of contents

(*S*)-2,5-Difluoro-*N*-tosylbenzenesulfonimidoyl chloride (1p)

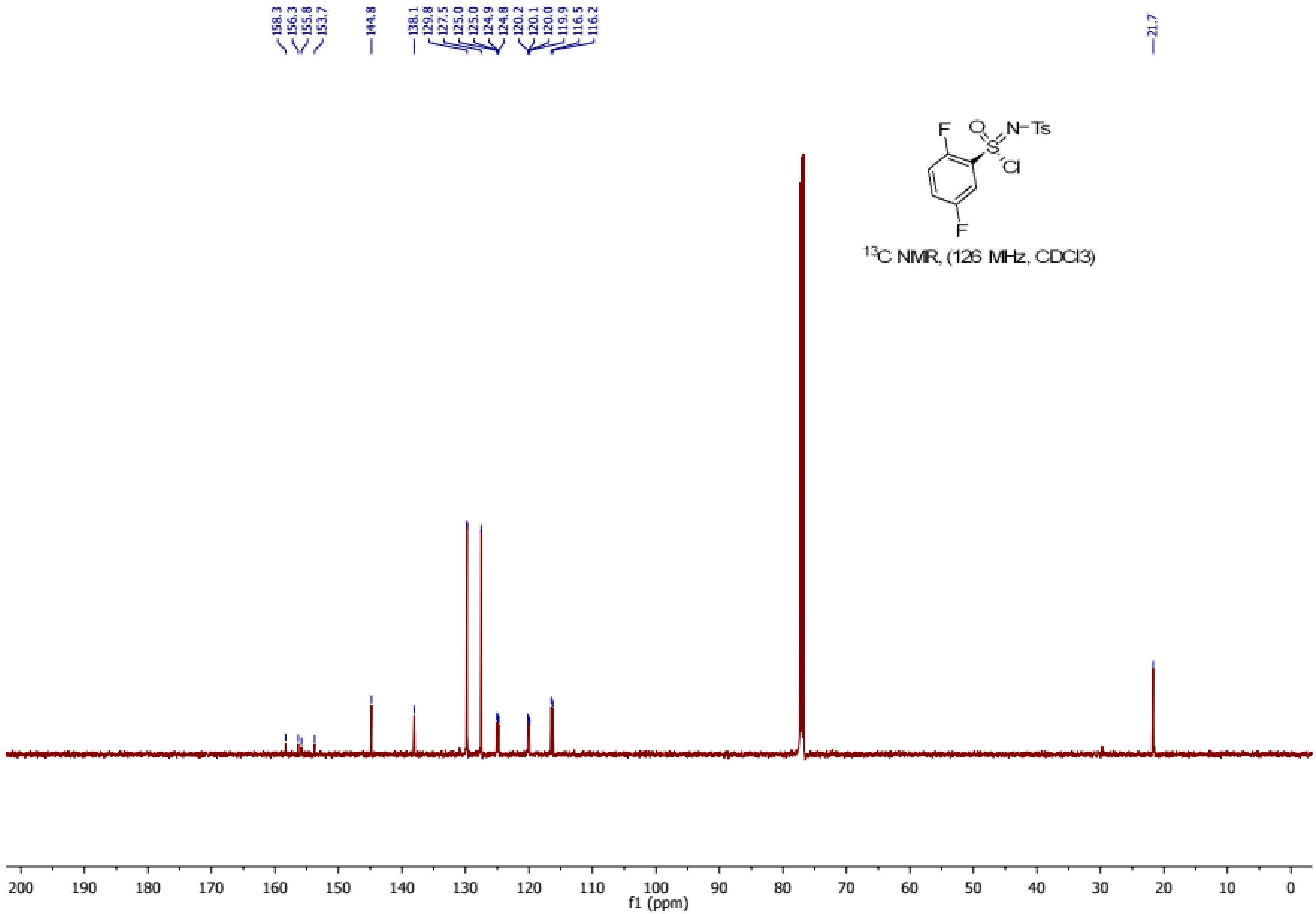

Go to table of contents

(*S*)-3,5-Dichloro-*N*-tosylbenzenesulfonimidoyl chloride (1q)

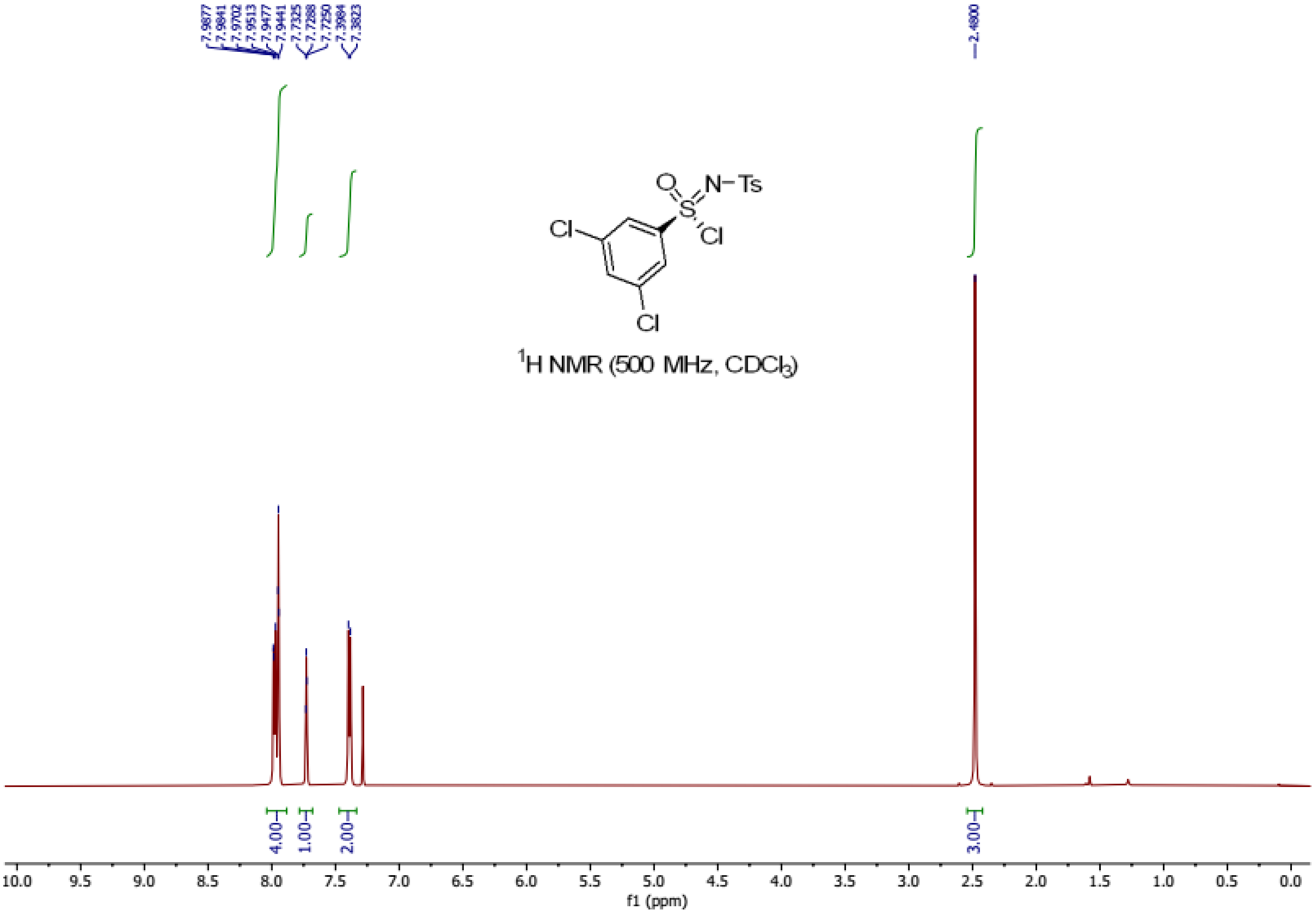

Go to table of contents

(*S*)-3,5-Dichloro-*N*-tosylbenzenesulfonimidoyl chloride (1q)

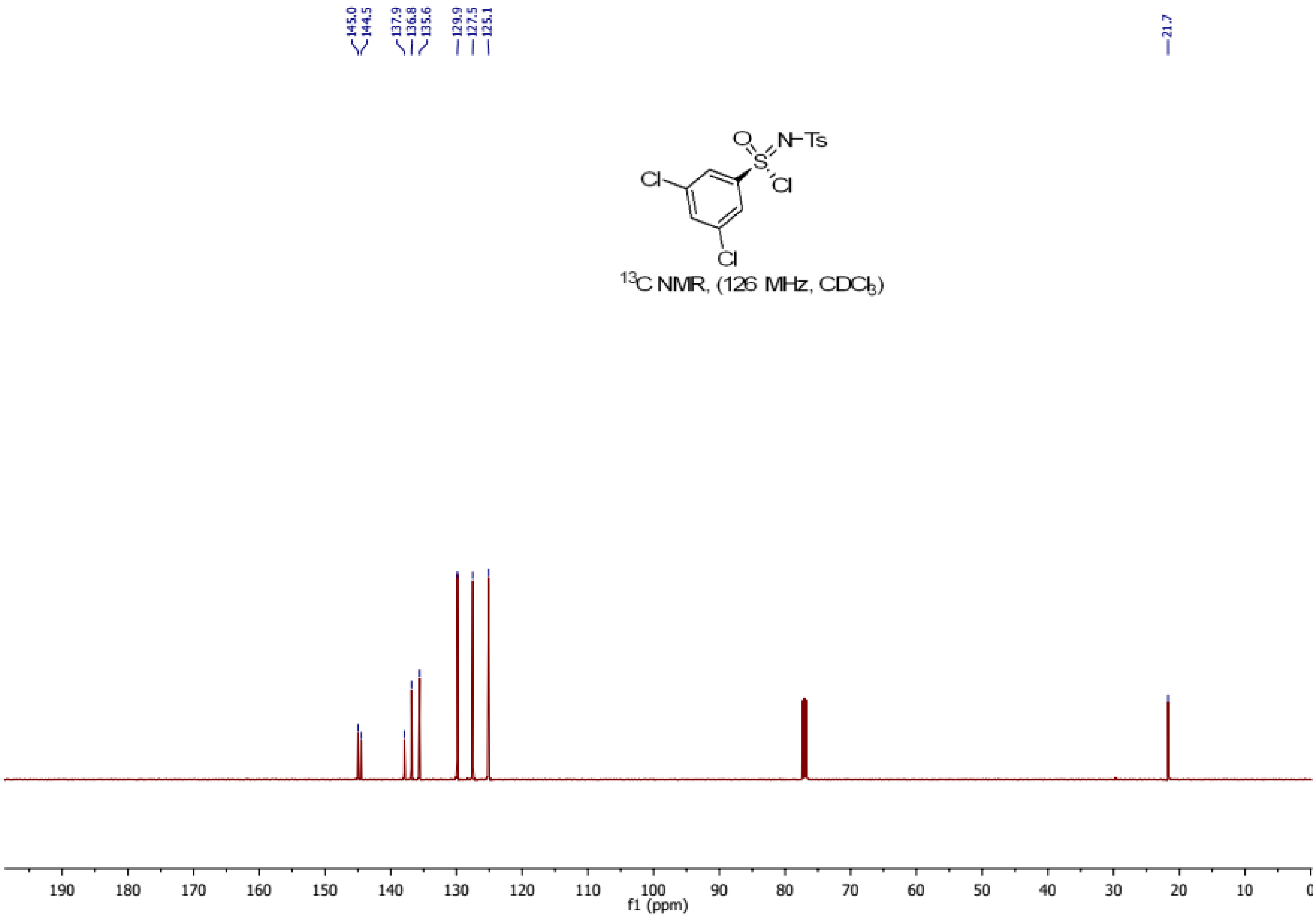

Go to table of contents

(*S*)-3-Chloro-4-fluoro-*N*-tosylbenzenesulfonimidoyl chloride (1r)

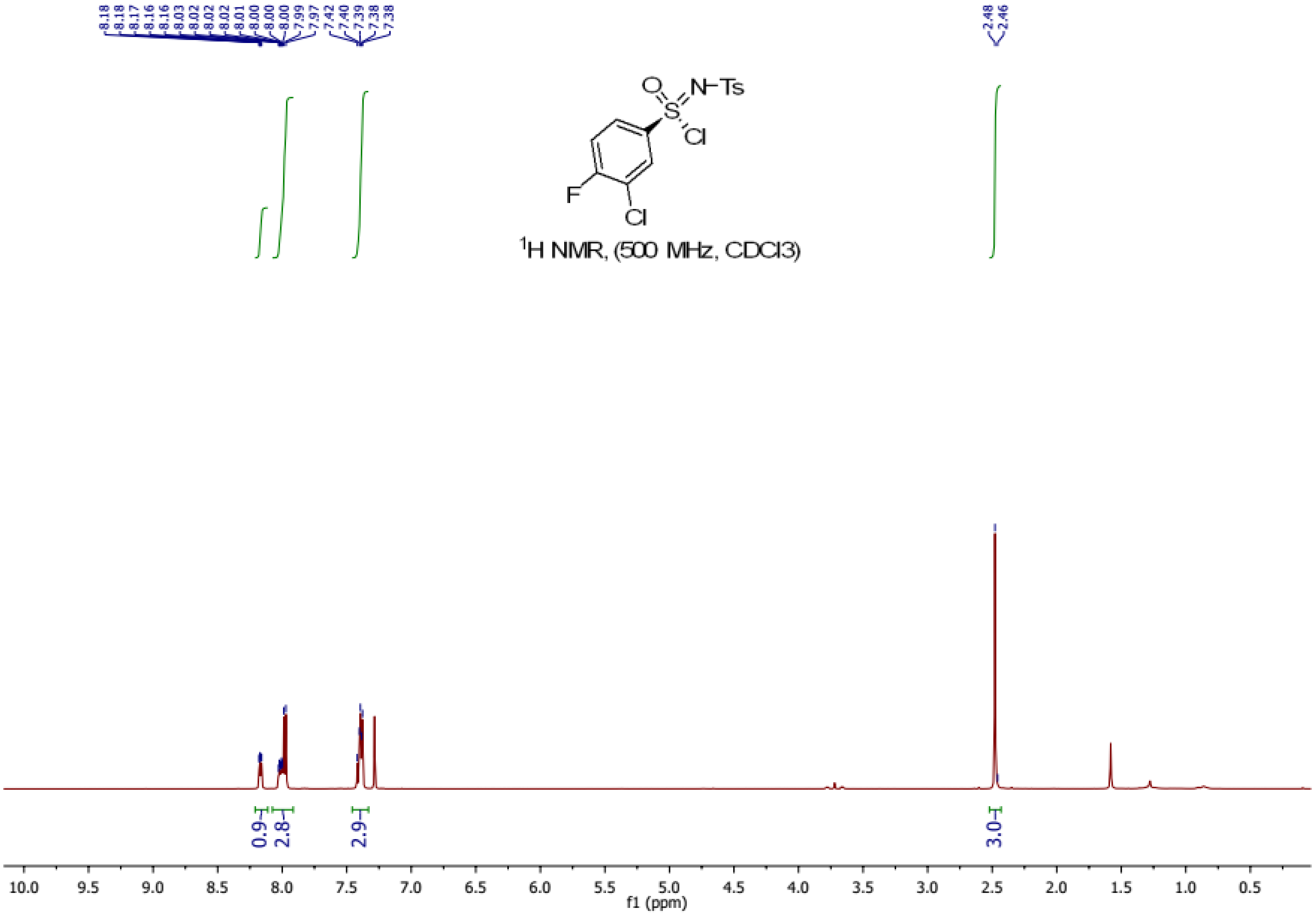

Go to table of contents

(*S*)-3-Chloro-4-fluoro-*N*-tosylbenzenesulfonimidoyl chloride (1r)

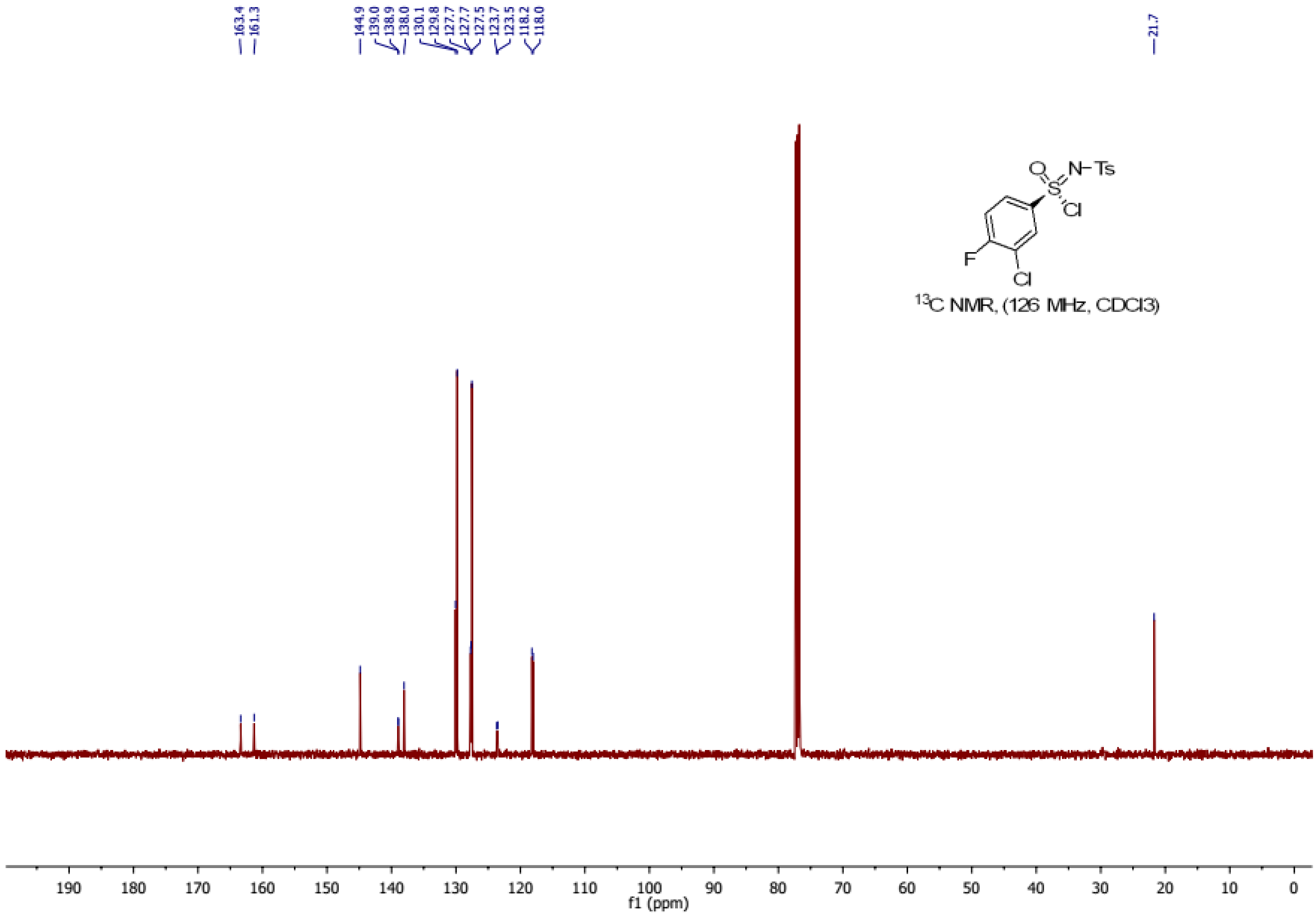

Go to table of contents

(*S*)-4-Fluoro-*N*-tosyl-3-(trifluoromethyl)benzenesulfonimidoyl chloride (1s)

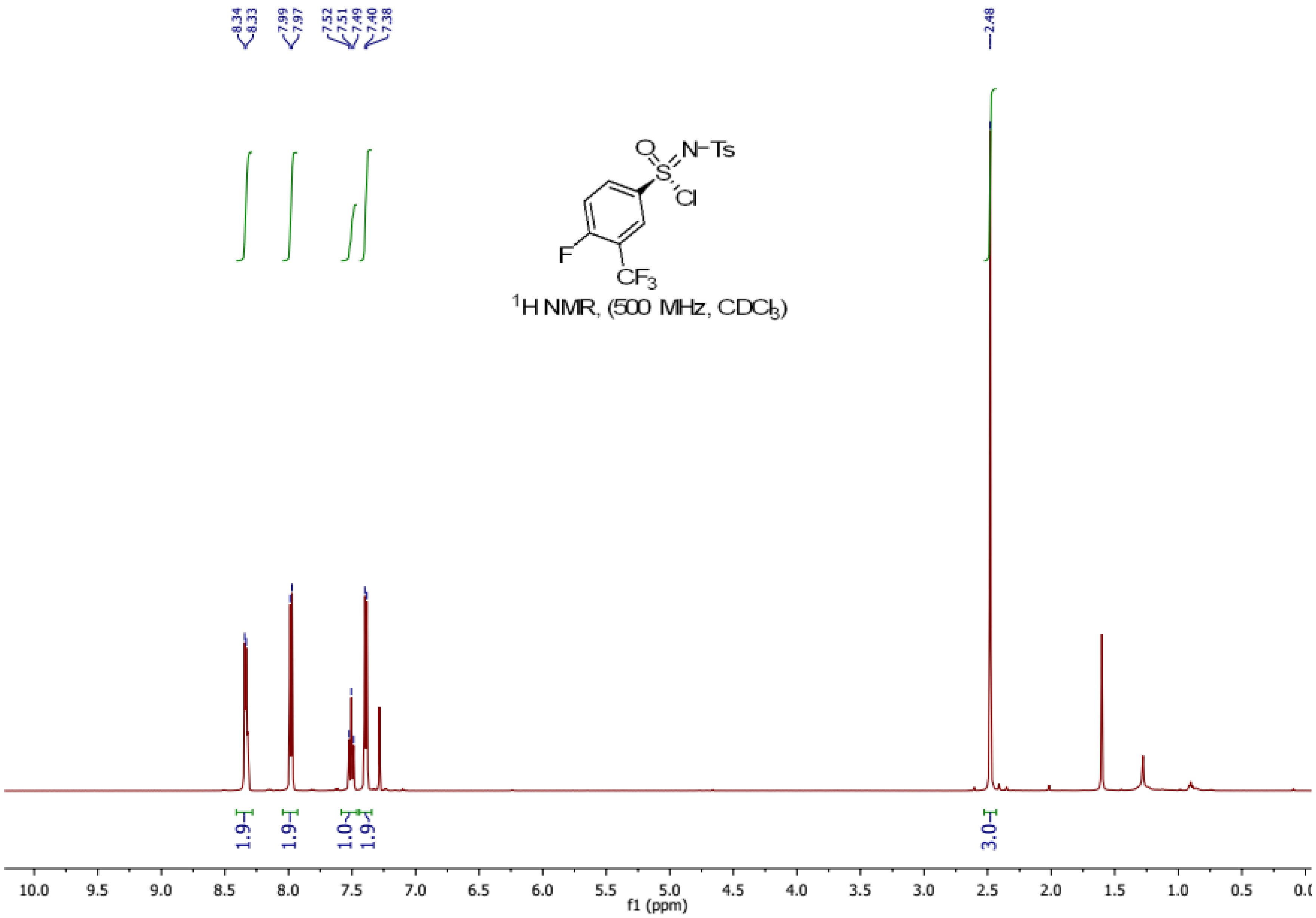

Go to table of contents

(*S*)-4-Fluoro-*N*-tosyl-3-(trifluoromethyl)benzenesulfonimidoyl chloride (1s)

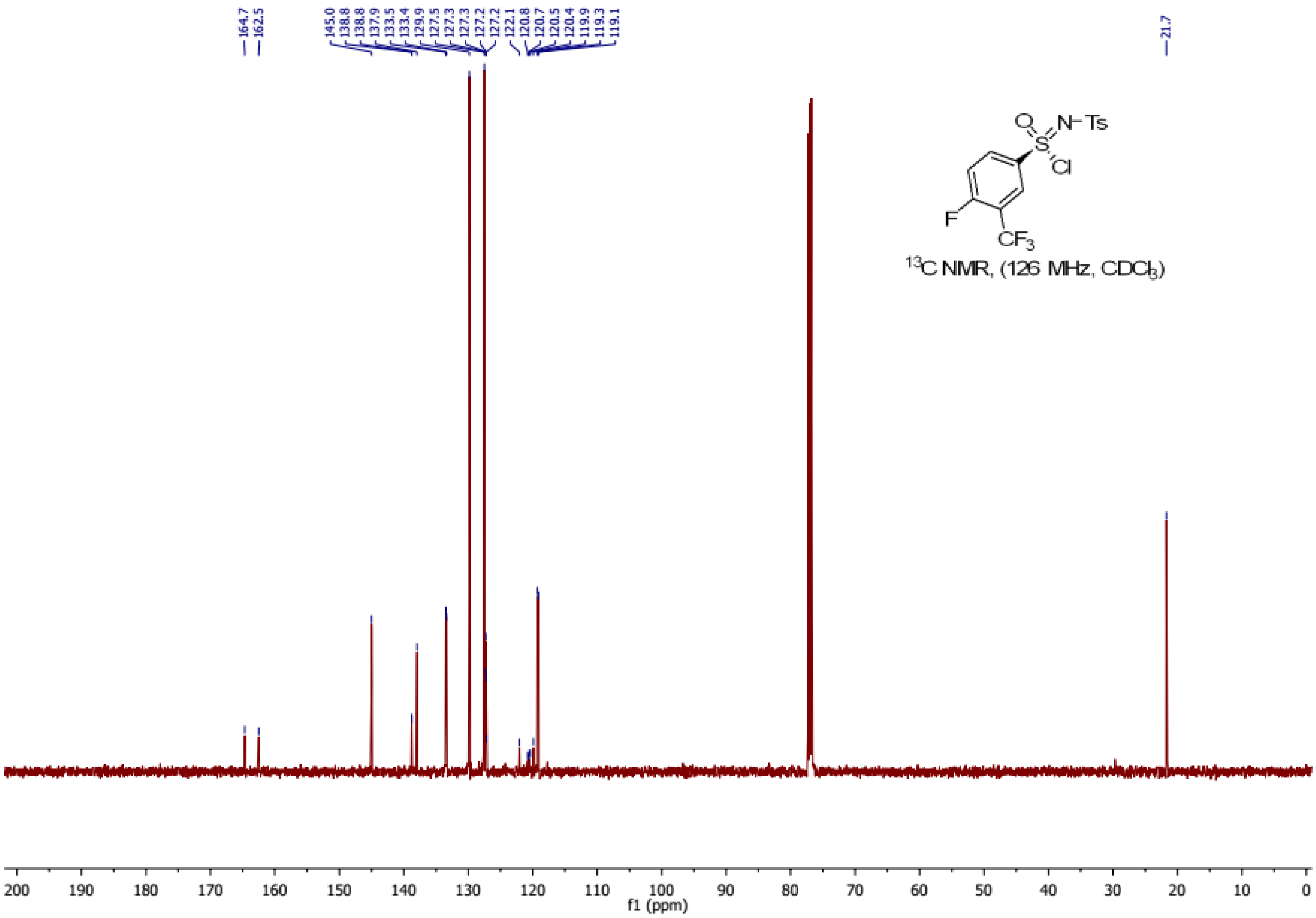

Go to table of contents

(*S*)-4-Chloro-*N*-tosyl-3-(trifluoromethyl)benzenesulfonimidoyl chloride (1t)

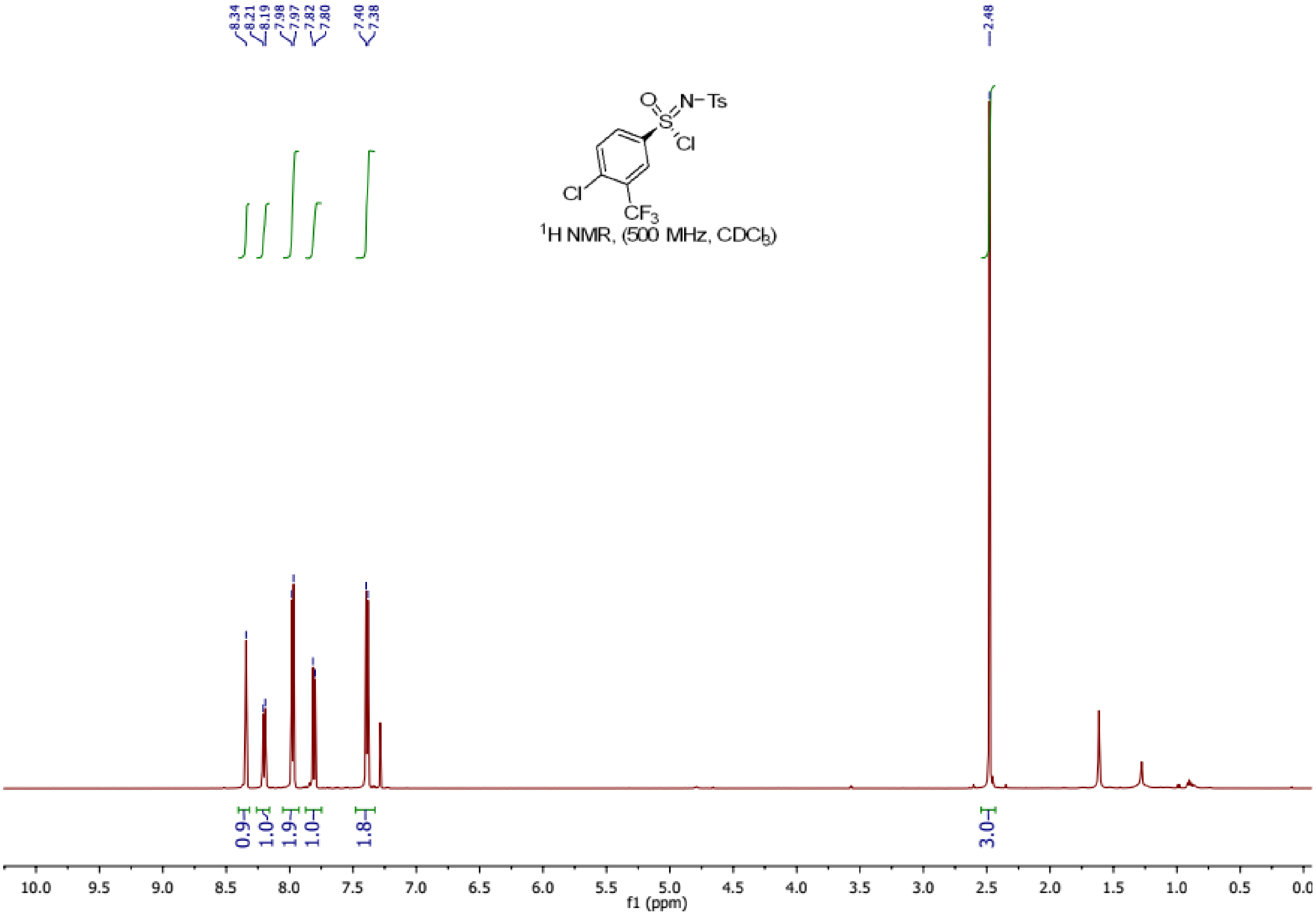

Go to table of contents

(*S*)-4-Chloro-*N*-tosyl-3-(trifluoromethyl)benzenesulfonimidoyl chloride (1t)

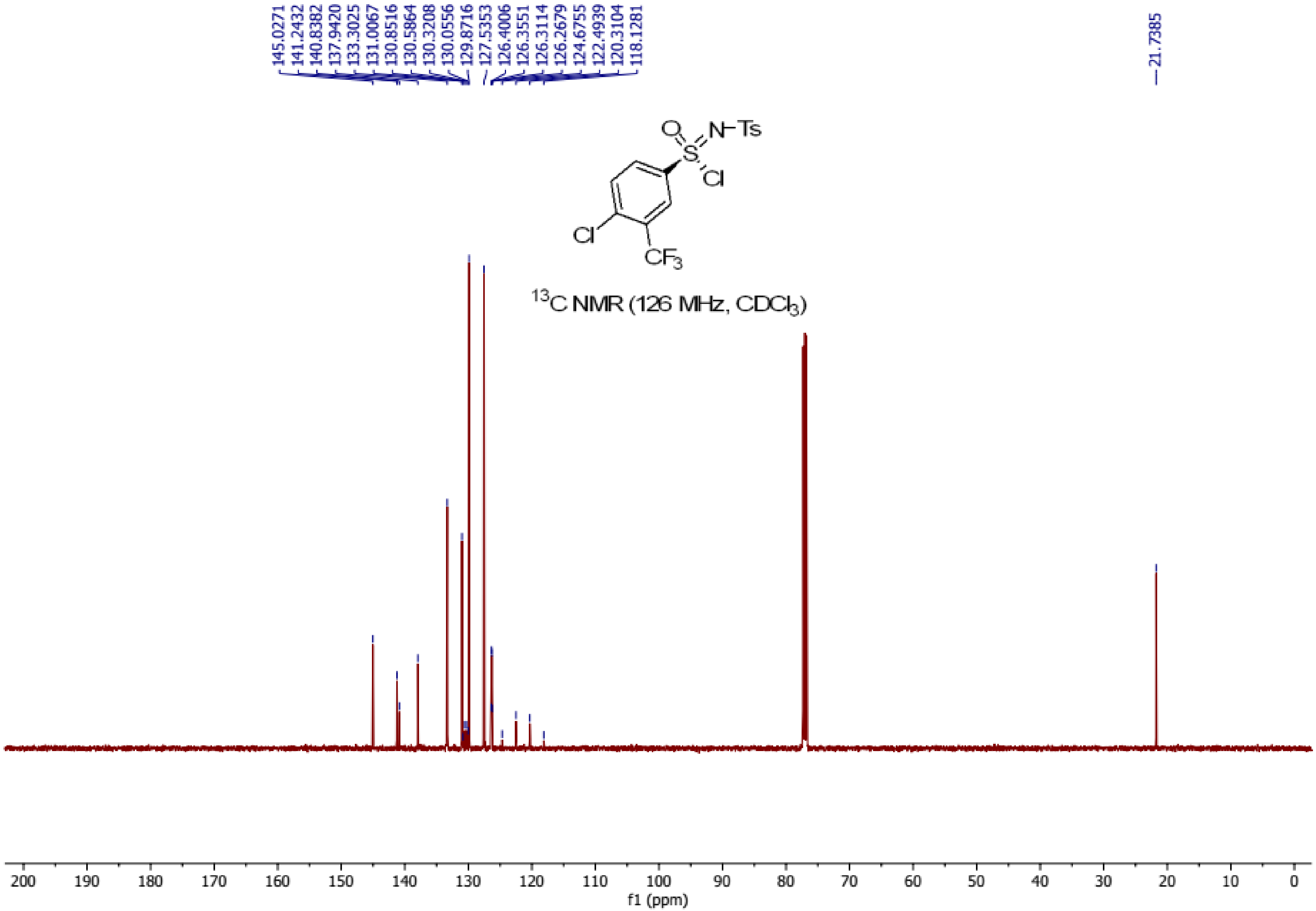

Go to table of contents

(*S*)-4-Bromo-3-methyl-*N*-tosylbenzenesulfonimidoyl chloride (1u)

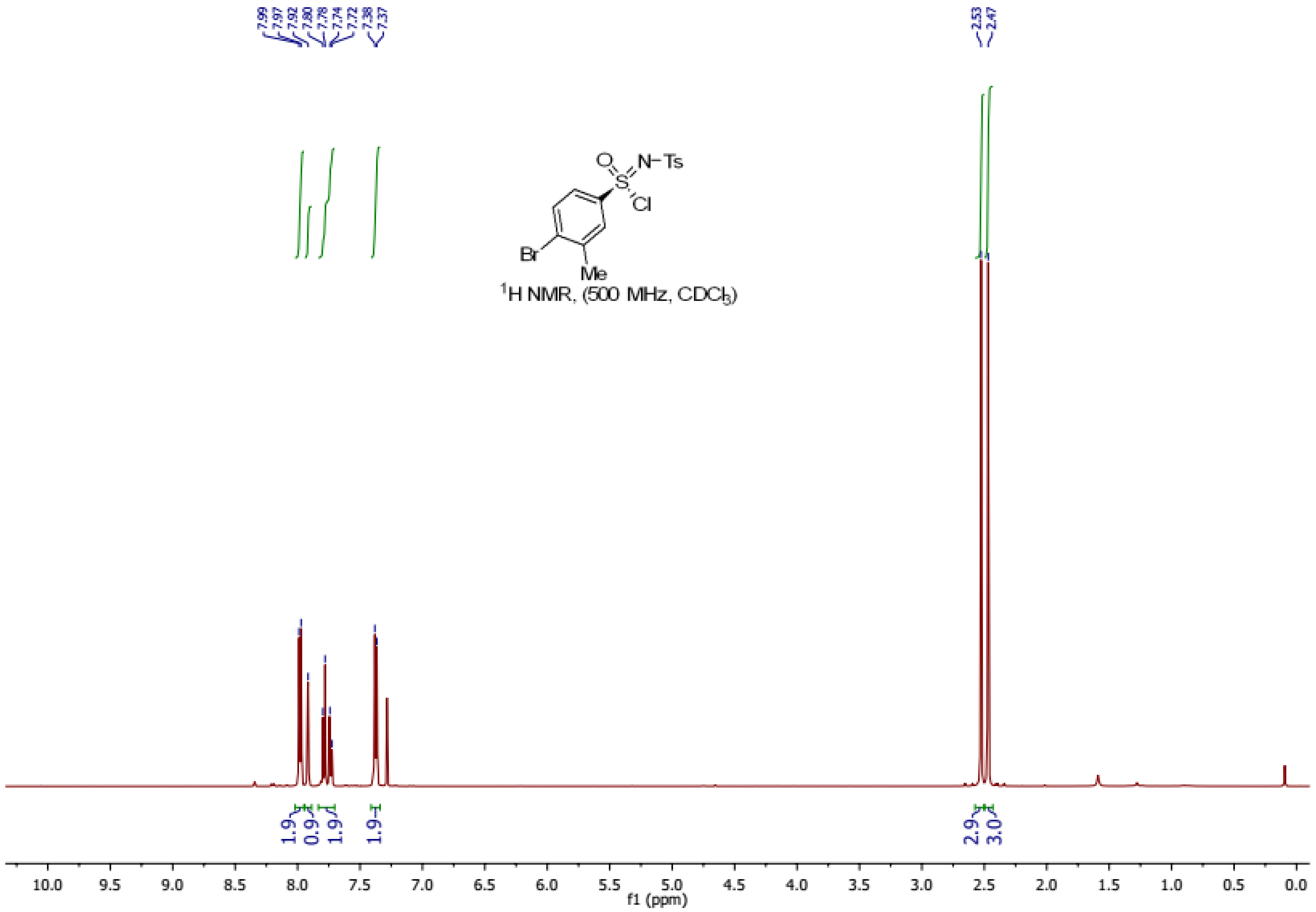

Go to table of contents

(*S*)-4-Bromo-3-methyl-*N*-tosylbenzenesulfonimidoyl chloride (1u)

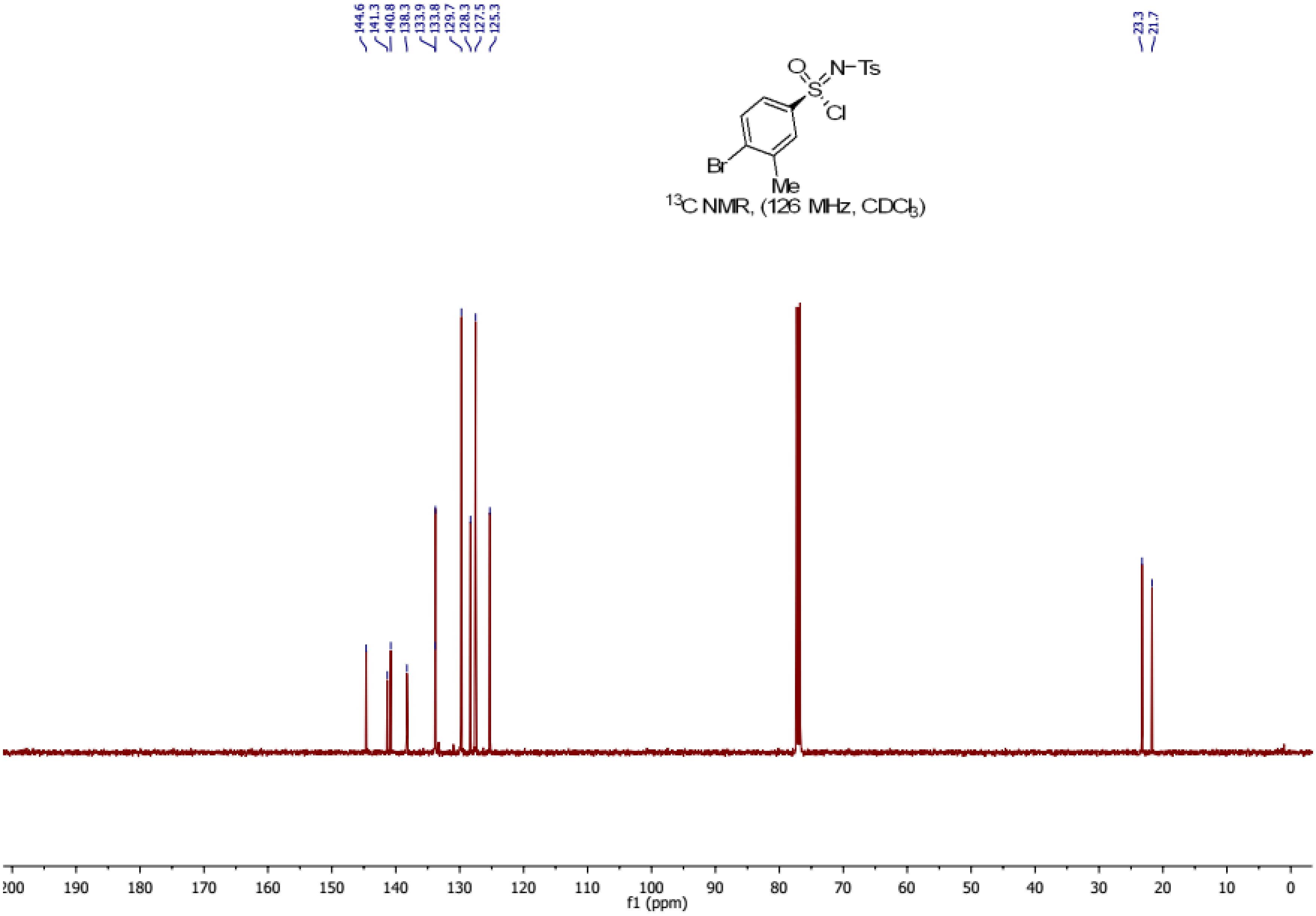

Go to table of contents

(*S*)-*N*-Tosylnaphthalene-2-sulfonimidoyl chloride (1v)

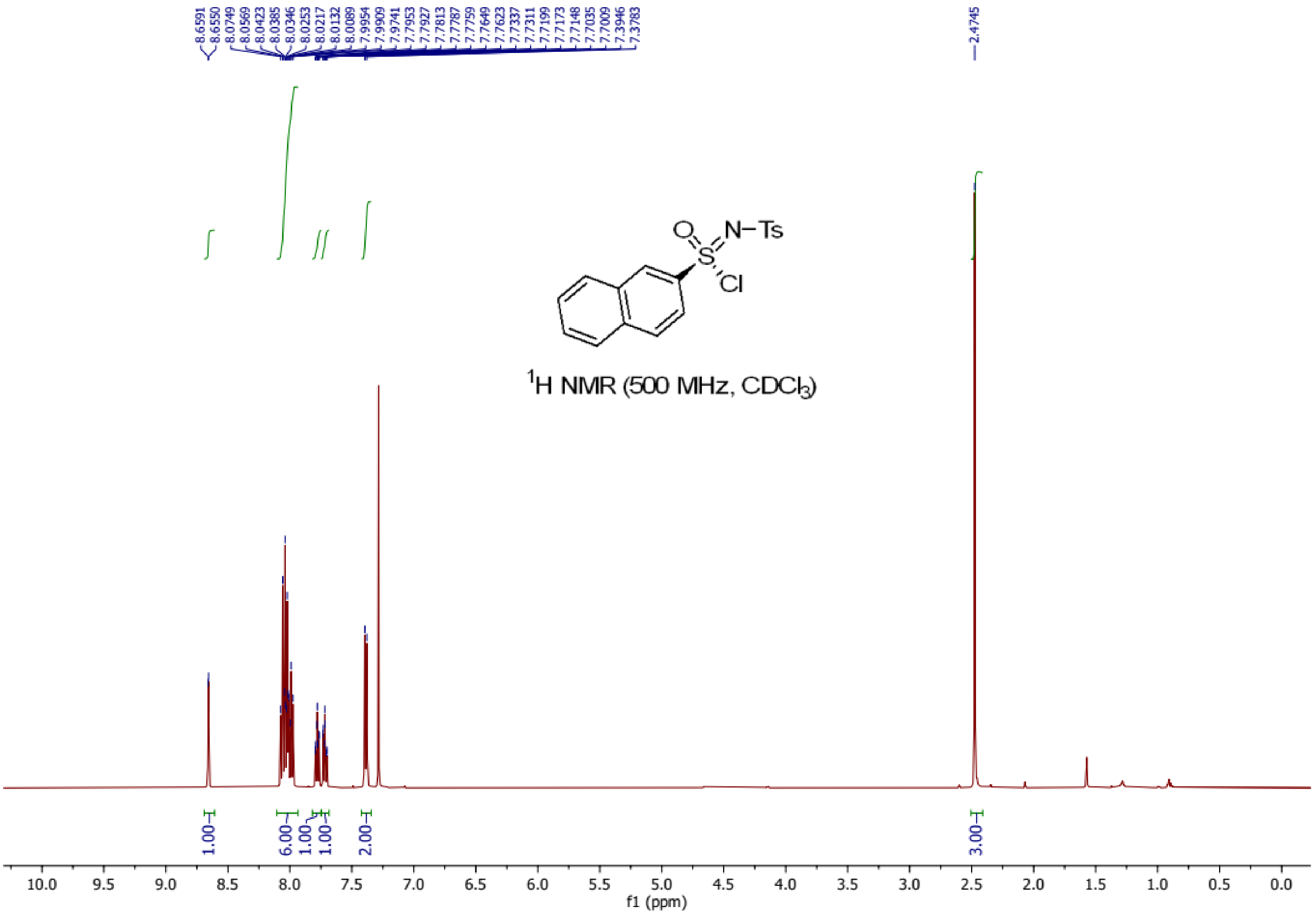

Go to table of contents

(*S*)-*N*-Tosylnaphthalene-2-sulfonimidoyl chloride (1v)

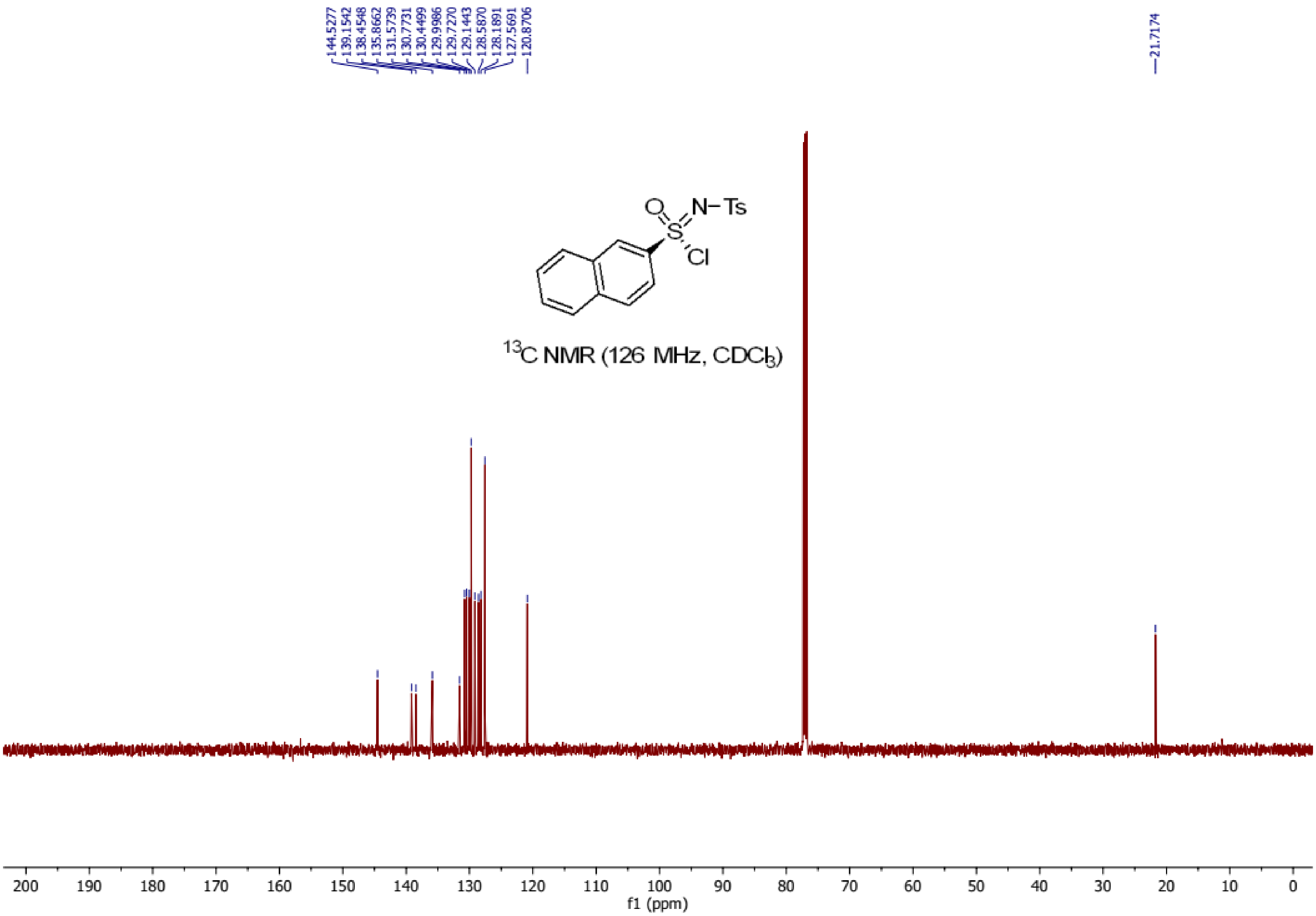

Go to table of contents

(*S*)-*N*-((4-Fluorophenyl)sulfonyl)benzenesulfonimidoyl chloride (1w)

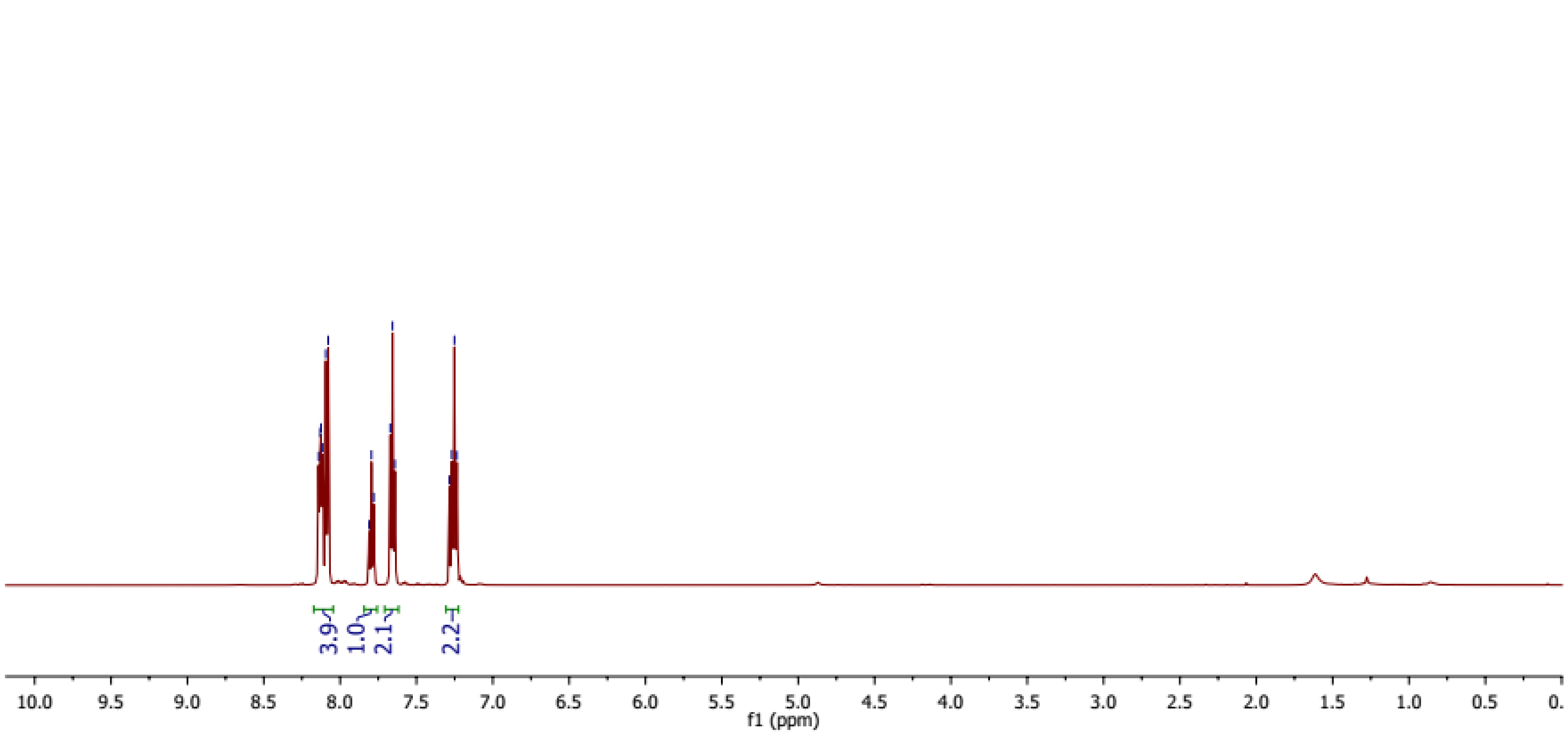

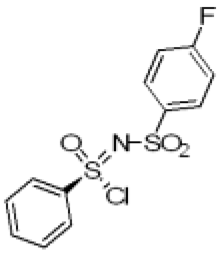

^{1}H NMR, (500 MHz, $CDCl_3$)

Go to table of contents

(*S*)-*N*-((4-Fluorophenyl)sulfonyl)benzenesulfonimidoyl chloride (1w)

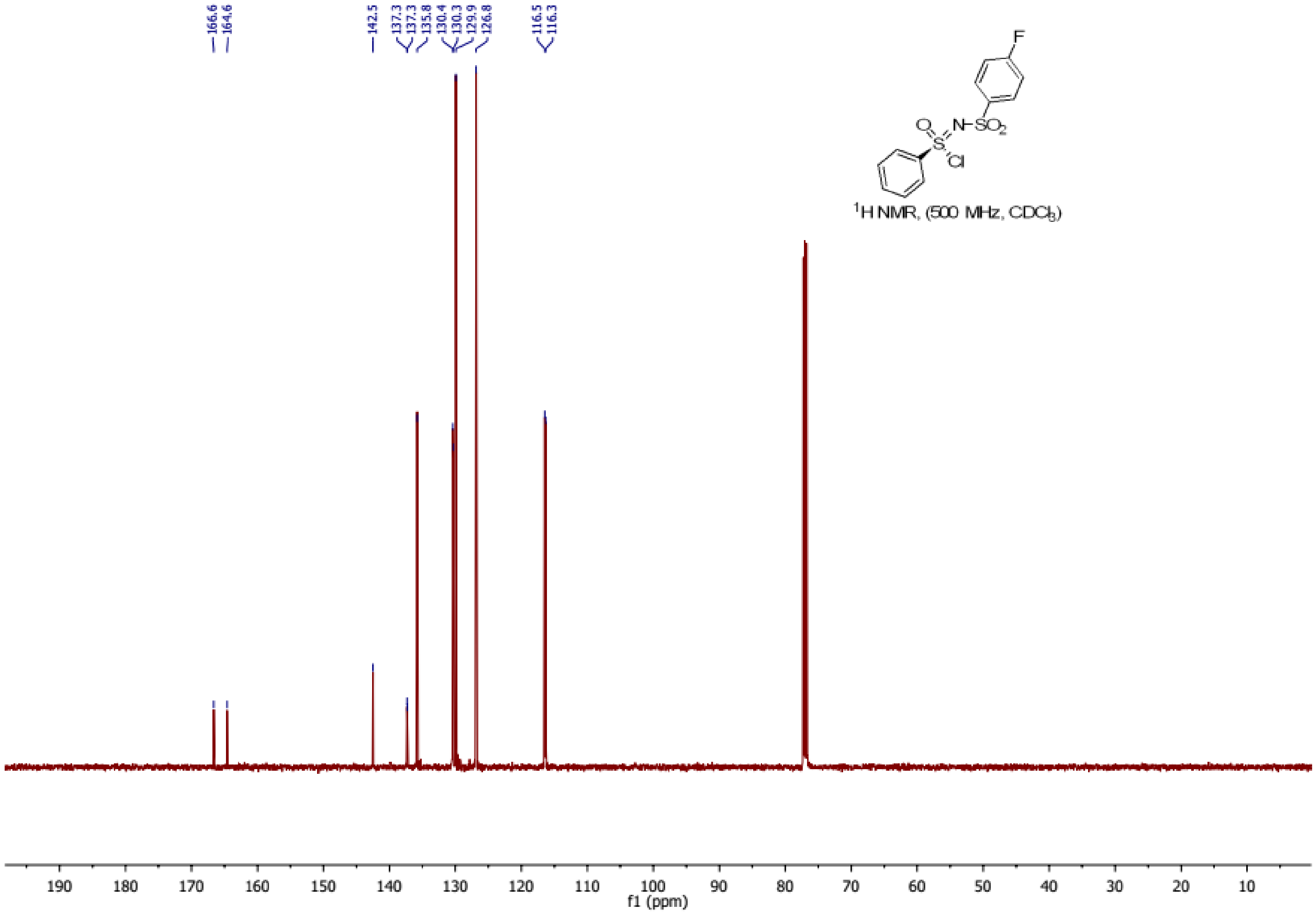

(*S*)-*N*-((3-Fluorophenyl)sulfonyl)benzenesulfonimidoyl chloride (1x)

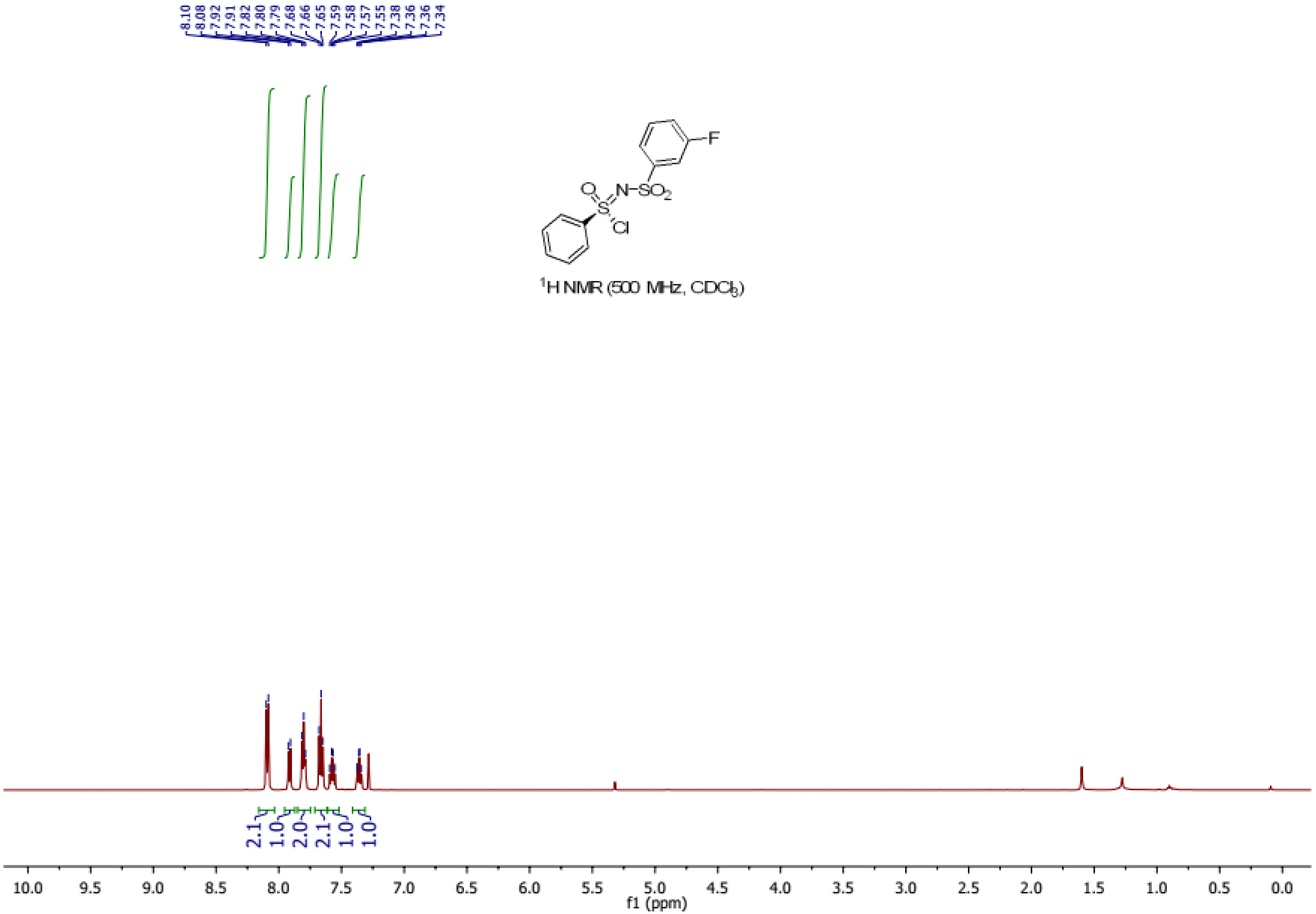

Go to table of contents

(*S*)-*N*-((3-Fluorophenyl)sulfonyl)benzenesulfonimidoyl chloride (1x)

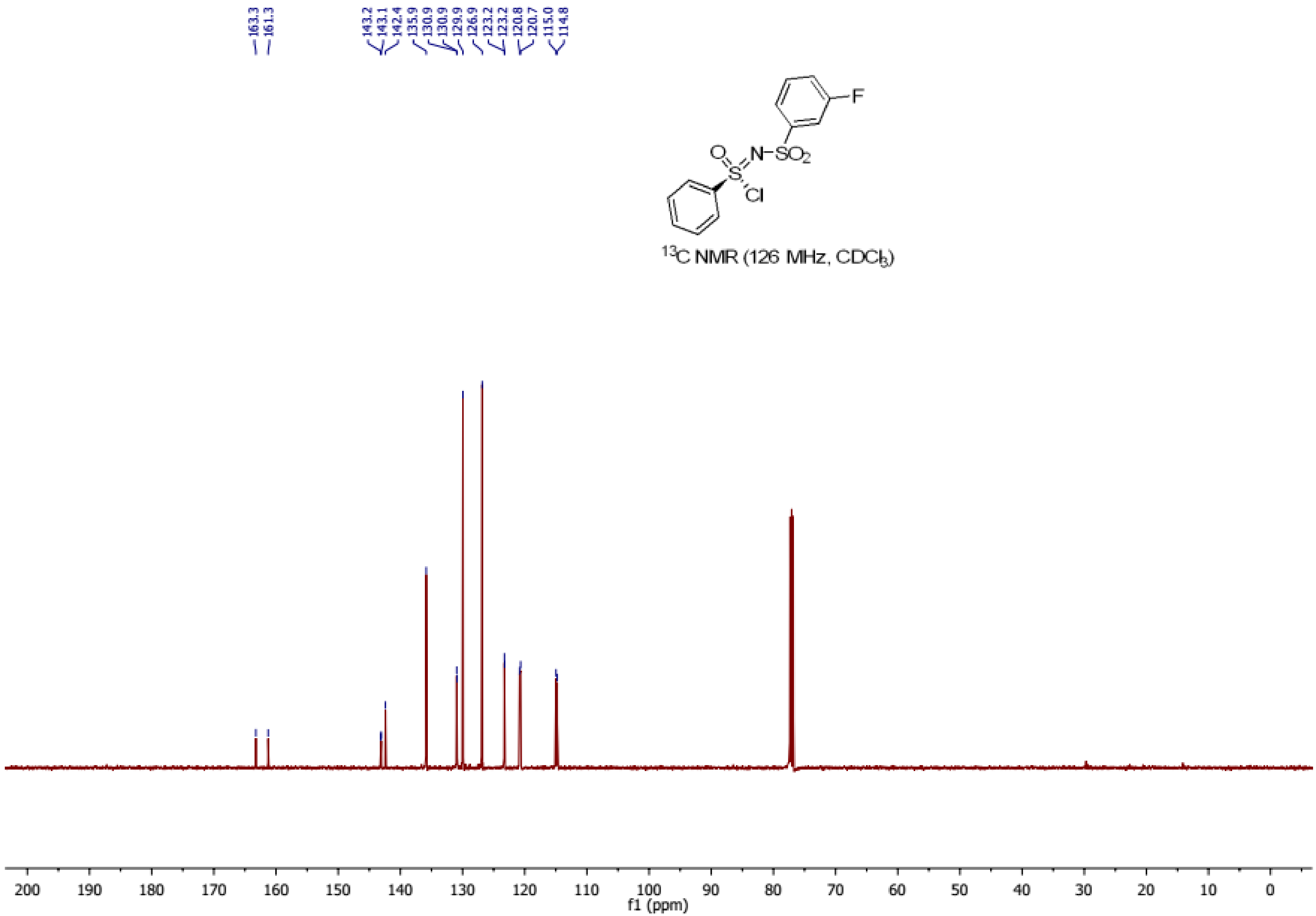

Go to table of contents

(*S*)-*N*-((3,4-Difluorophenyl)sulfonyl)benzenesulfonimidoyl chloride (1y)

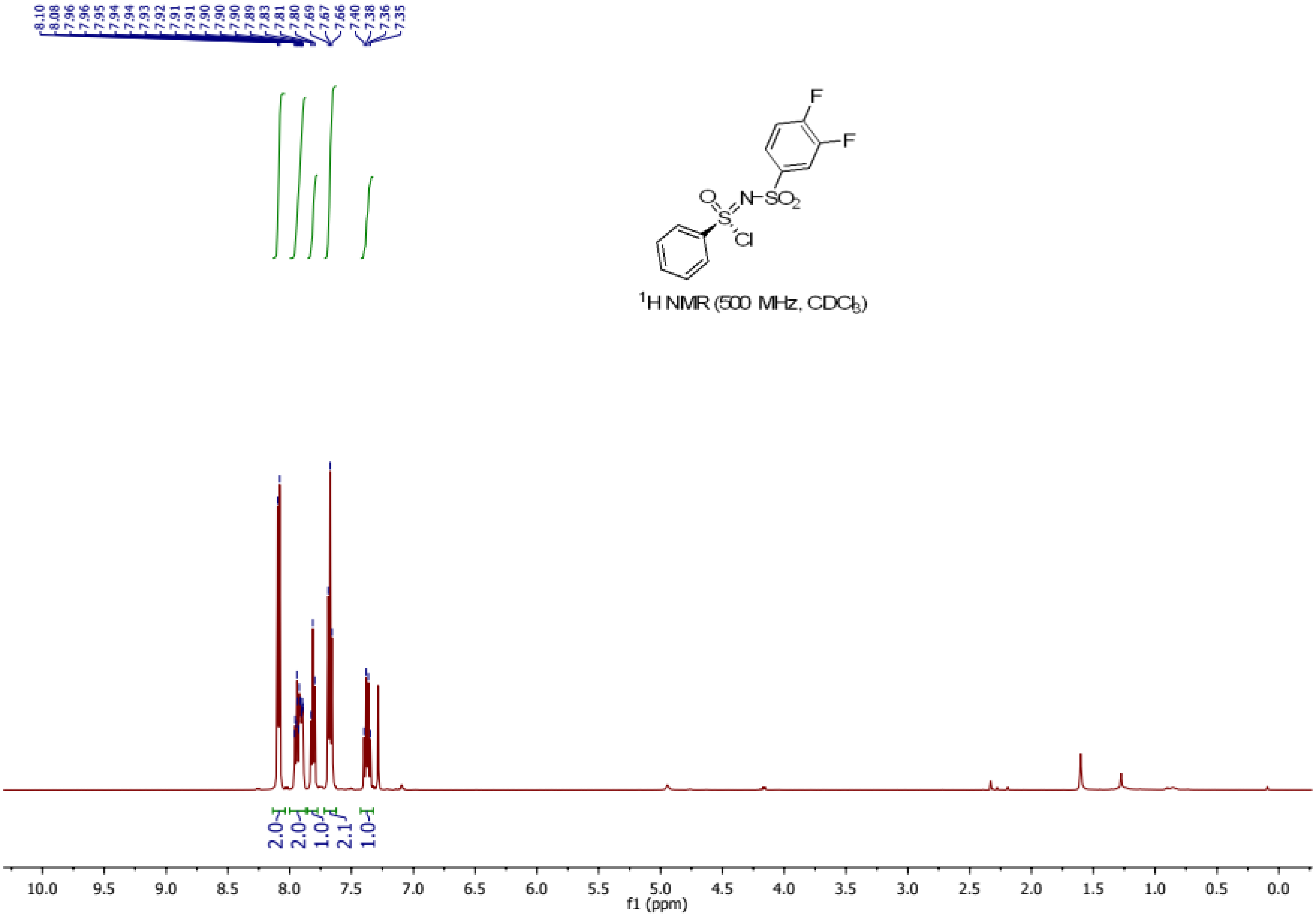

Go to table of contents

(*S*)-*N*-((3,4-Difluorophenyl)sulfonyl)benzenesulfonimidoyl chloride (1y)

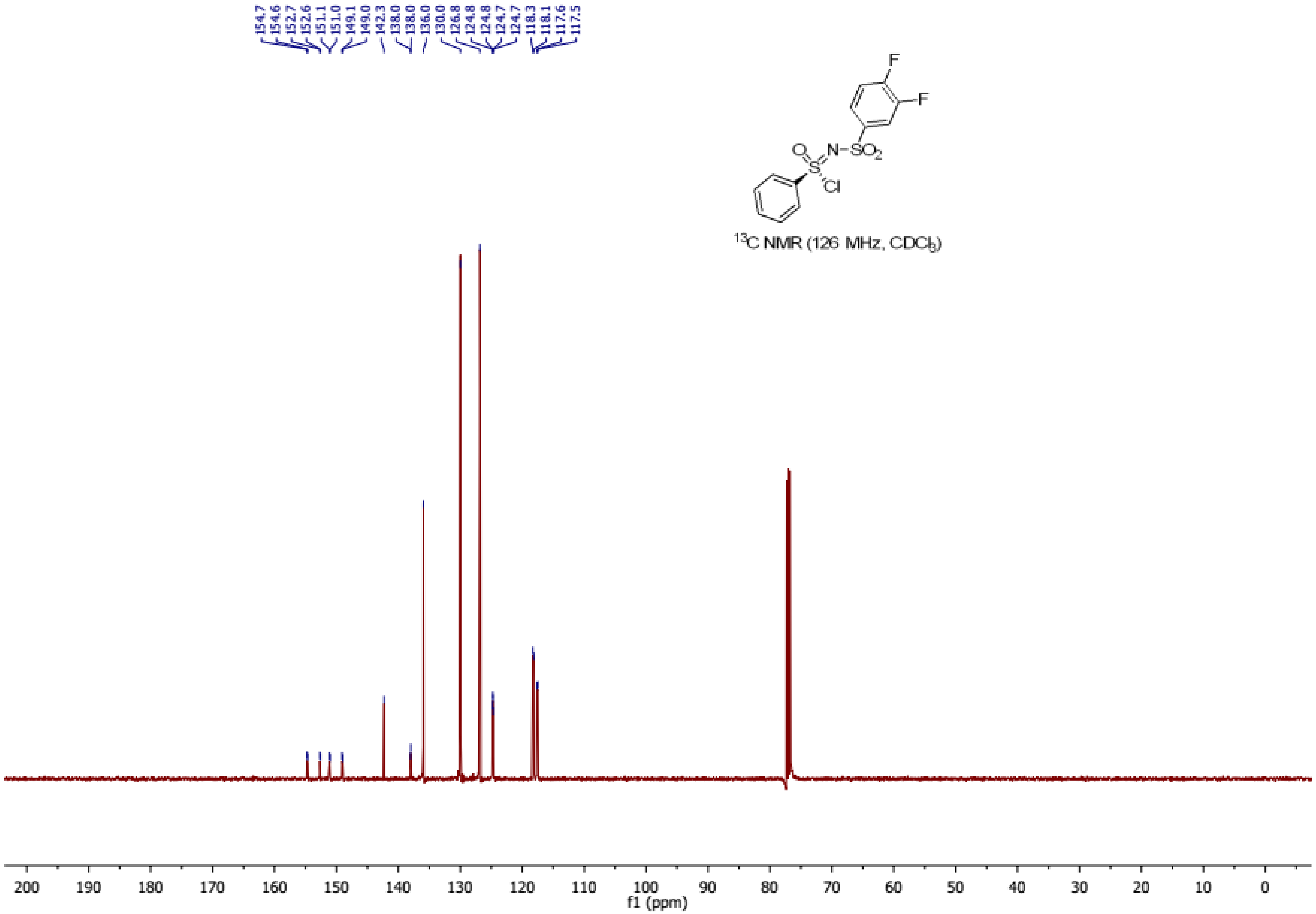

Go to table of contents

(*S*)-*N*-((4-Bromophenyl)sulfonyl)benzenesulfonimidoyl chloride (1z)

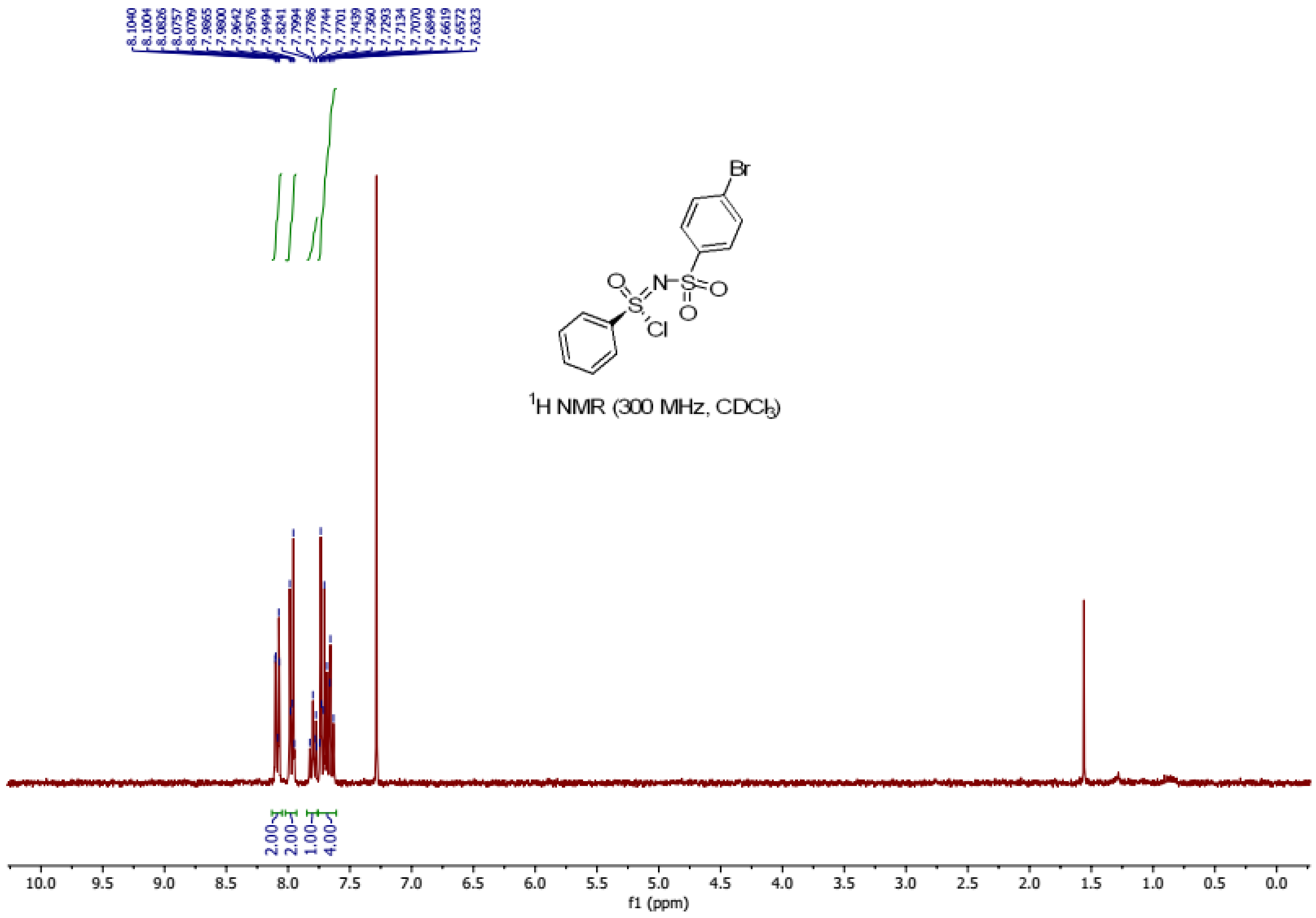

Go to table of contents

(*S*)-*N*-((4-Bromophenyl)sulfonyl)benzenesulfonimidoyl chloride (1z)

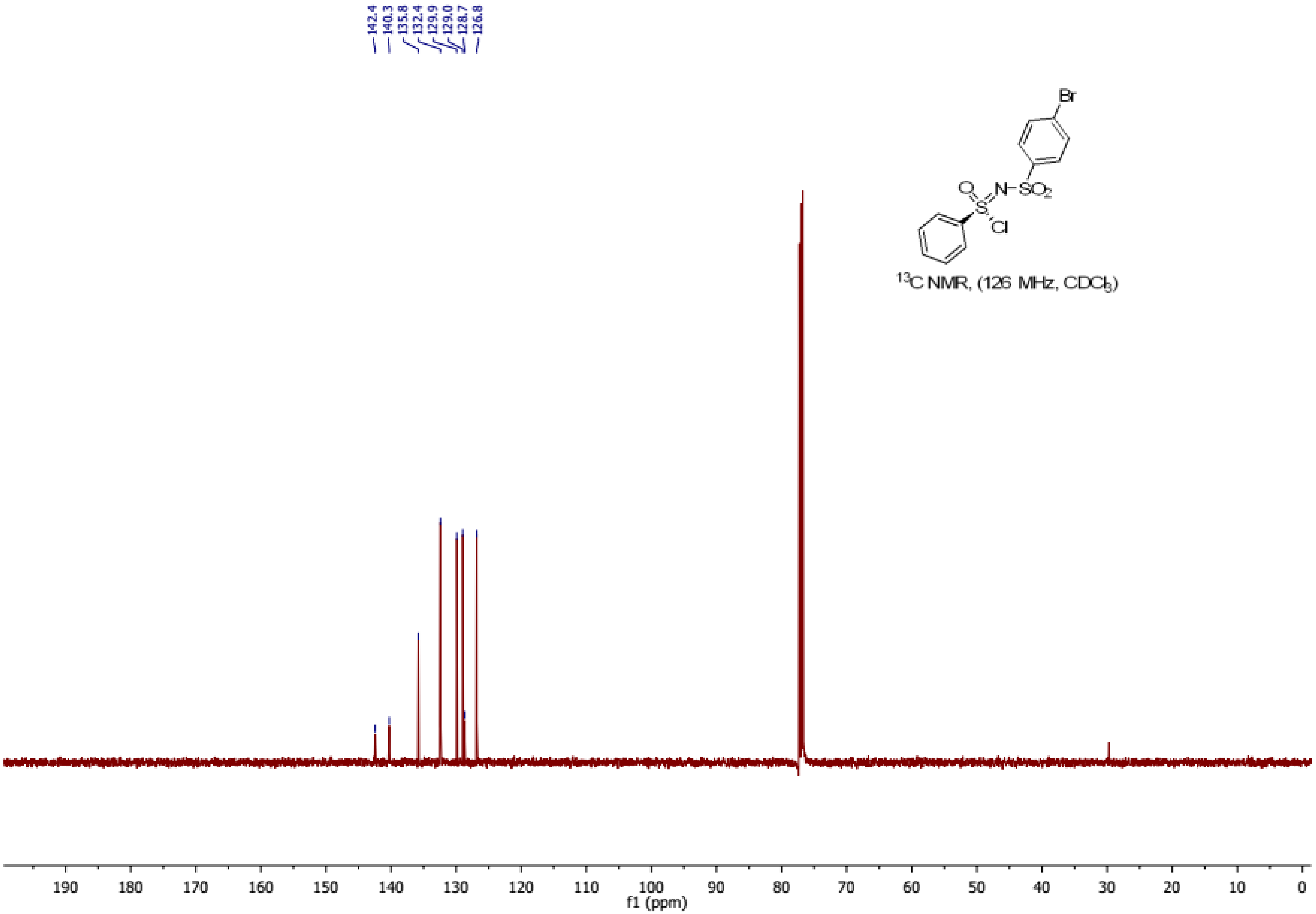

Go to table of contents

(*S*)-*N*-((3-(Trifluoromethyl)phenyl)sulfonyl)benzenesulfonimidoyl chloride (1aa)

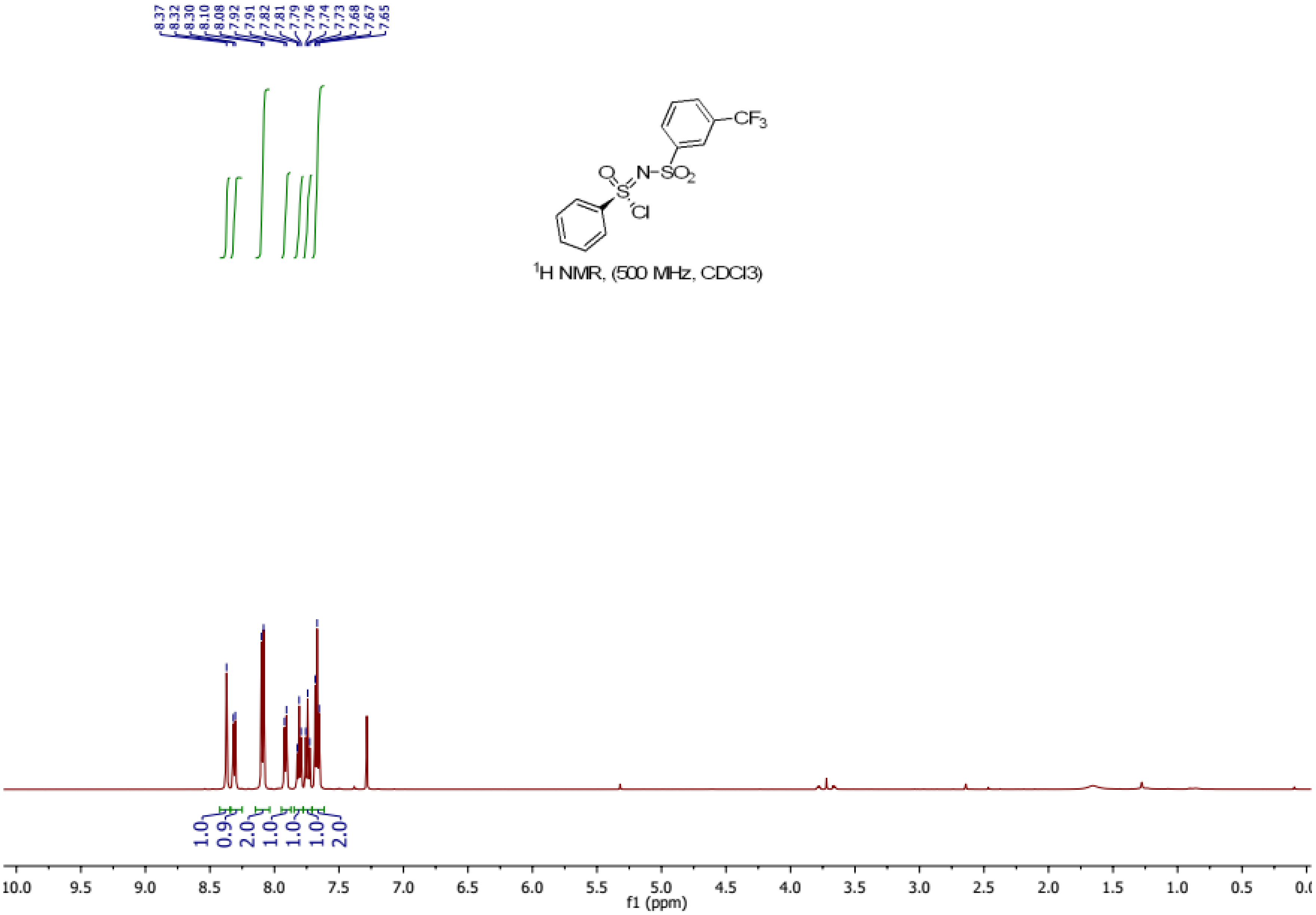

Go to table of contents

(*S*)-*N*-((3-(Trifluoromethyl)phenyl)sulfonyl)benzenesulfonimidoyl chloride (1aa)

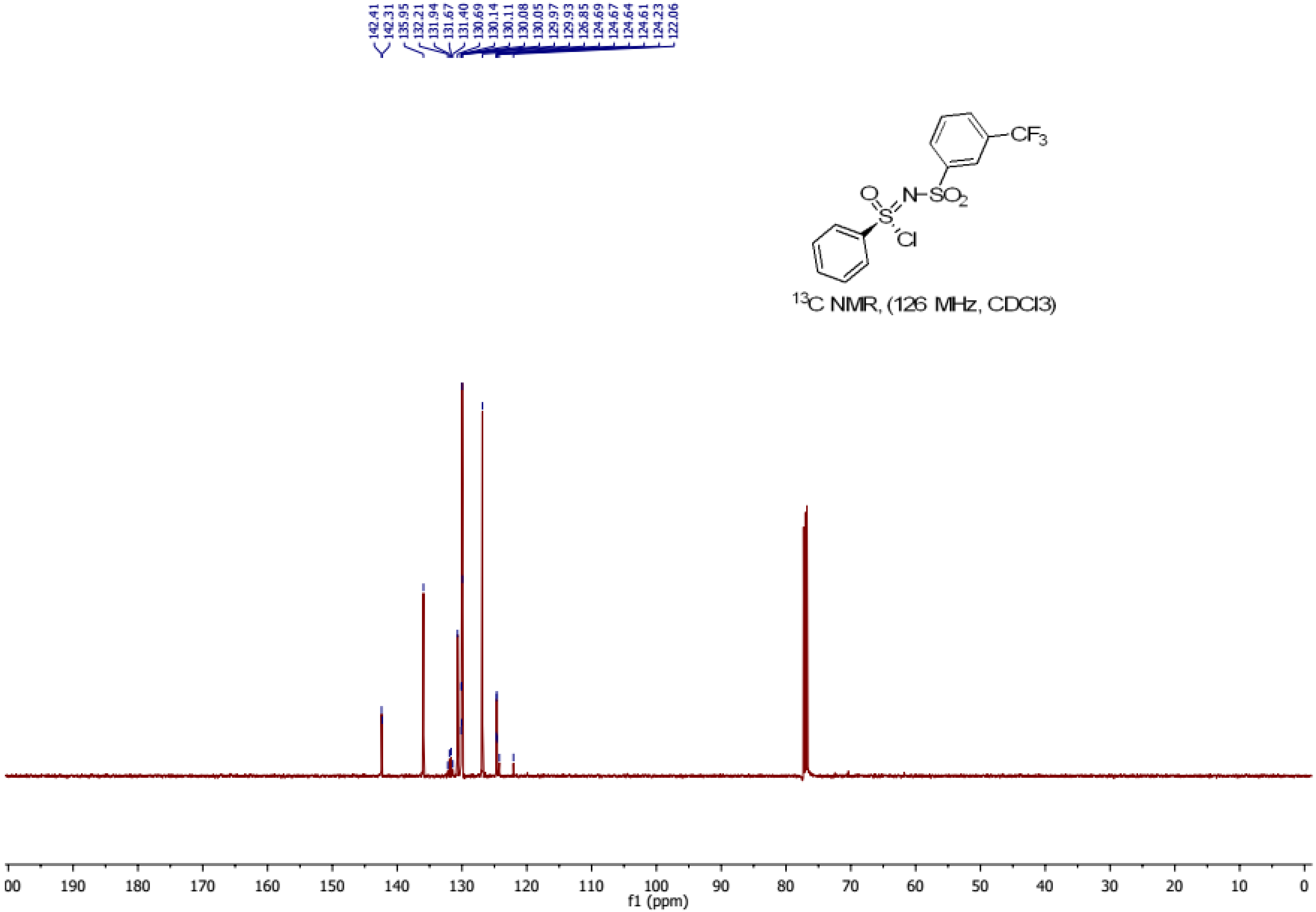

Go to table of contents

(*S*)-*N*-((3-Chloro-4-methoxyphenyl)sulfonyl)benzenesulfonimidoyl chloride (1ab)

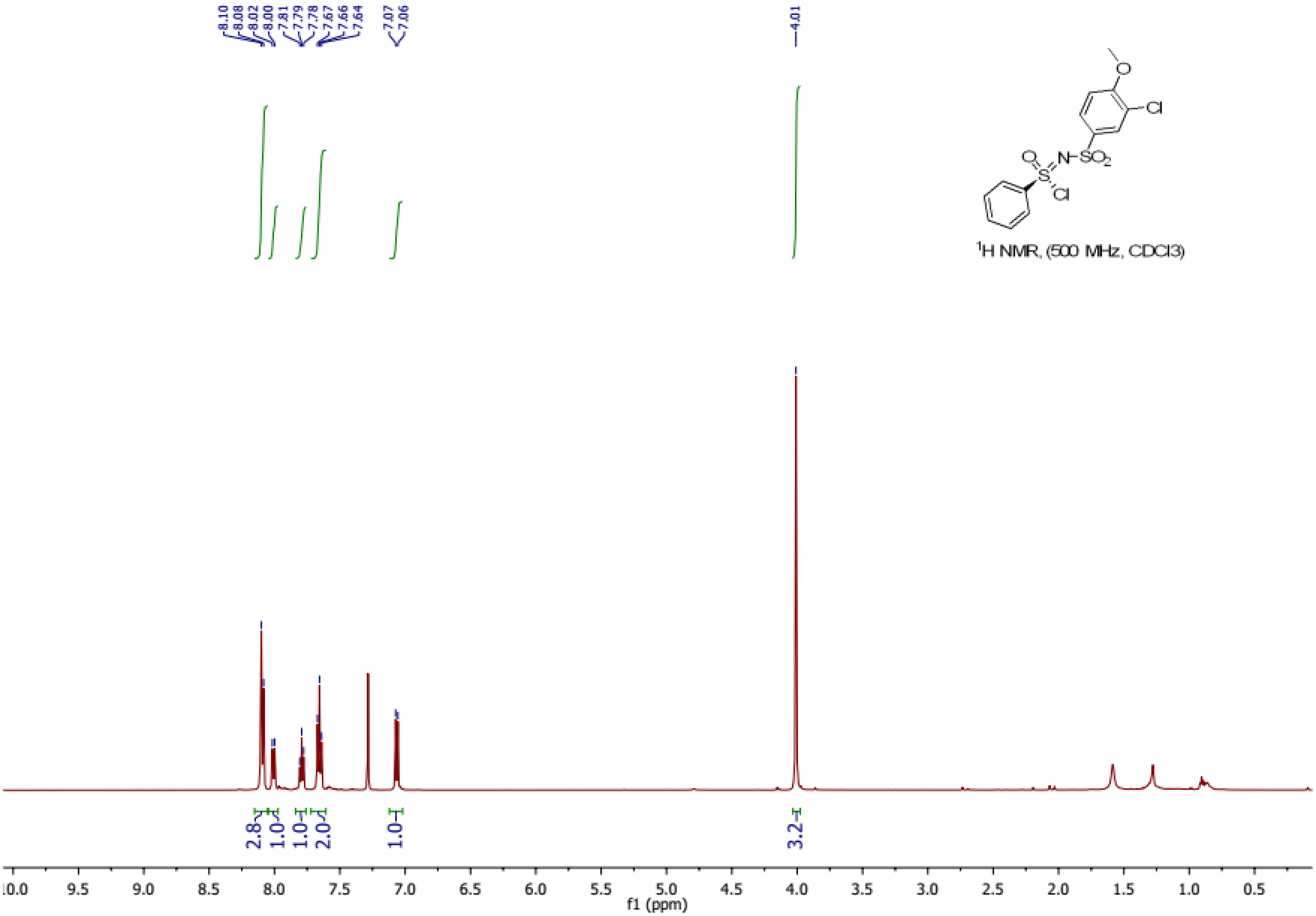

Go to table of contents

(*S*)-*N*-((3-Chloro-4-methoxyphenyl)sulfonyl)benzenesulfonimidoyl chloride (1ab)

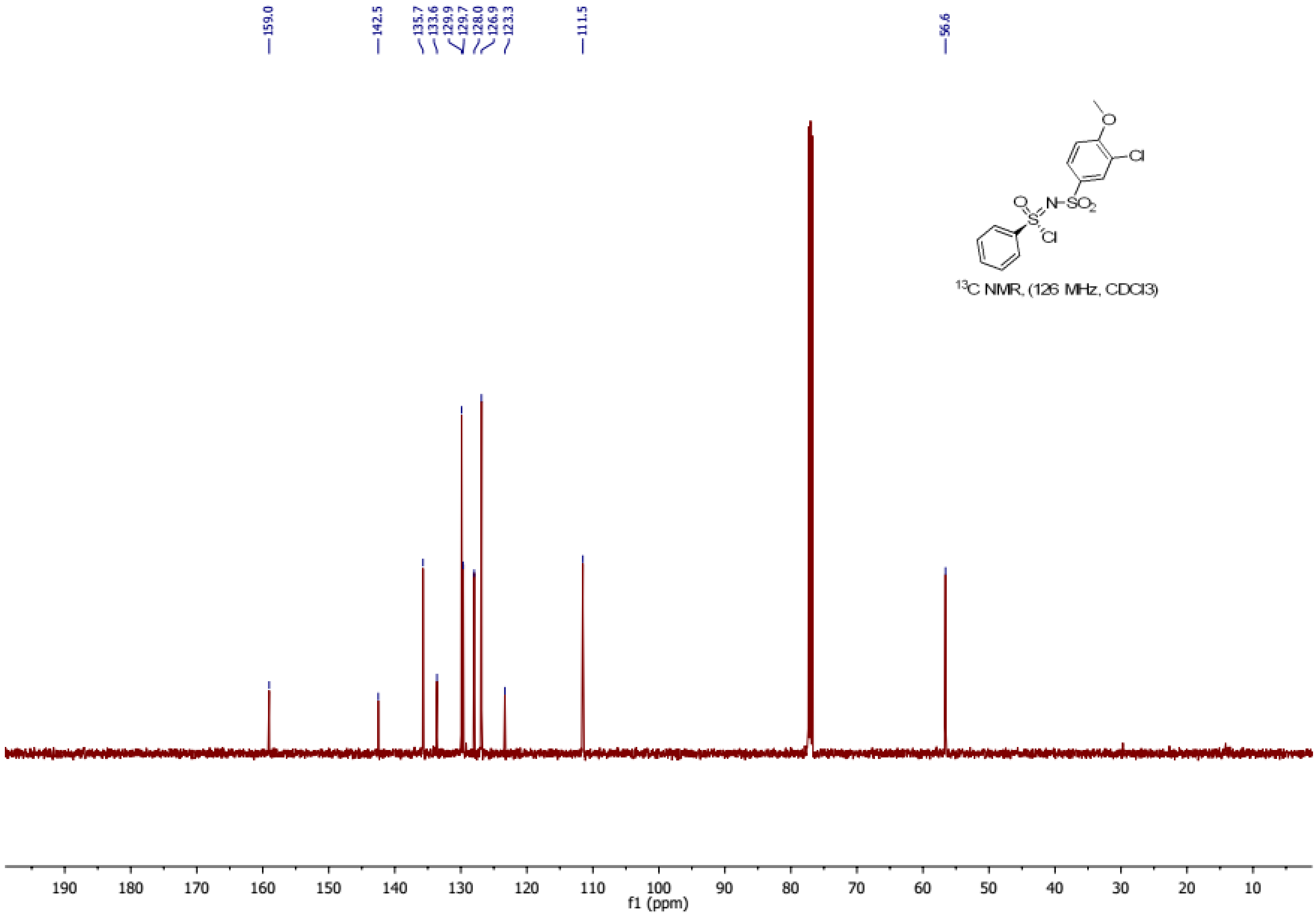

Go to table of contents

(*S*)-*N*-(Cyclopropylsulfonyl)benzenesulfonimidoyl chloride (1ac)

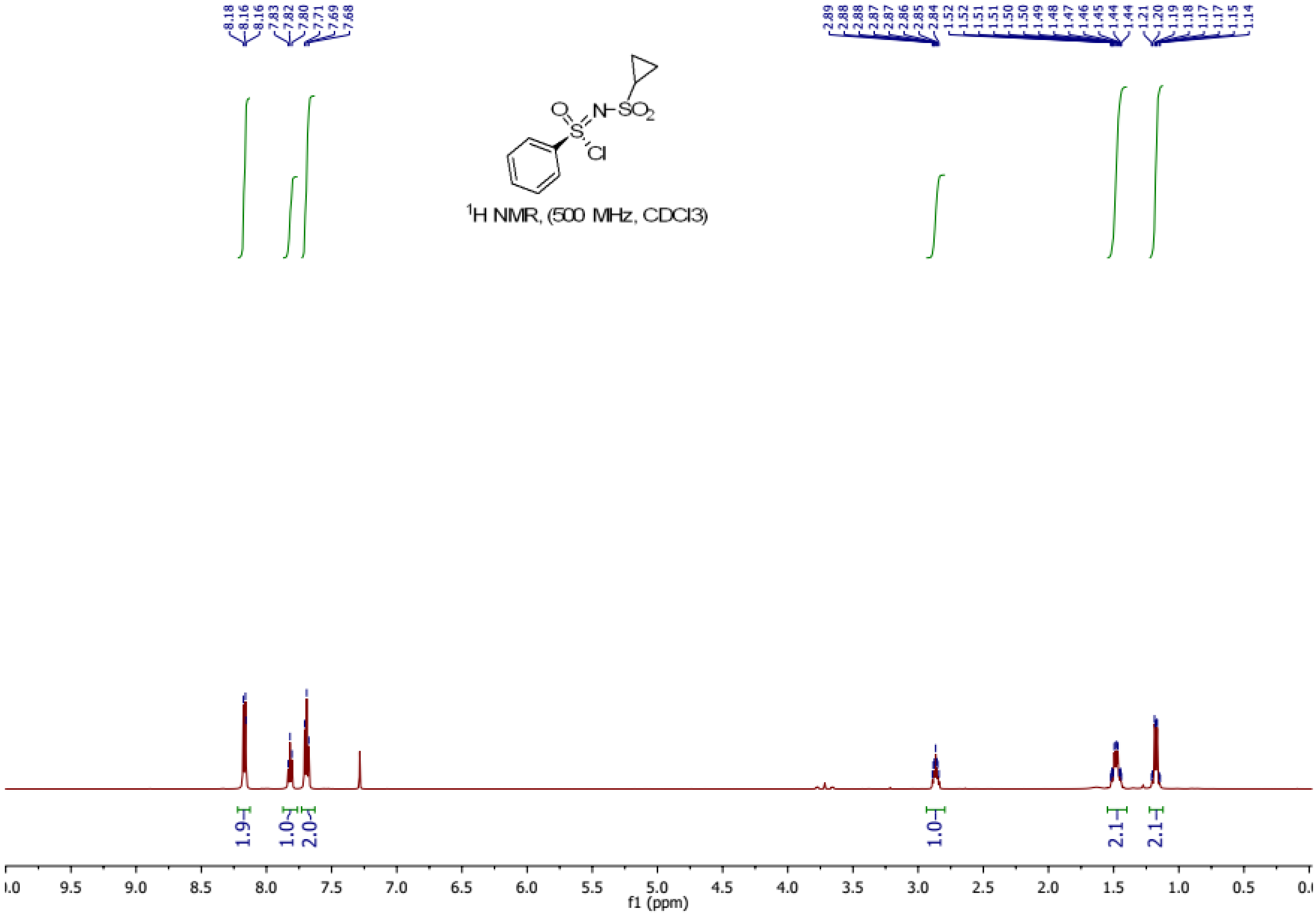

Go to table of contents

(*S*)-*N*-(Cyclopropylsulfonyl)benzenesulfonimidoyl chloride (1ac)

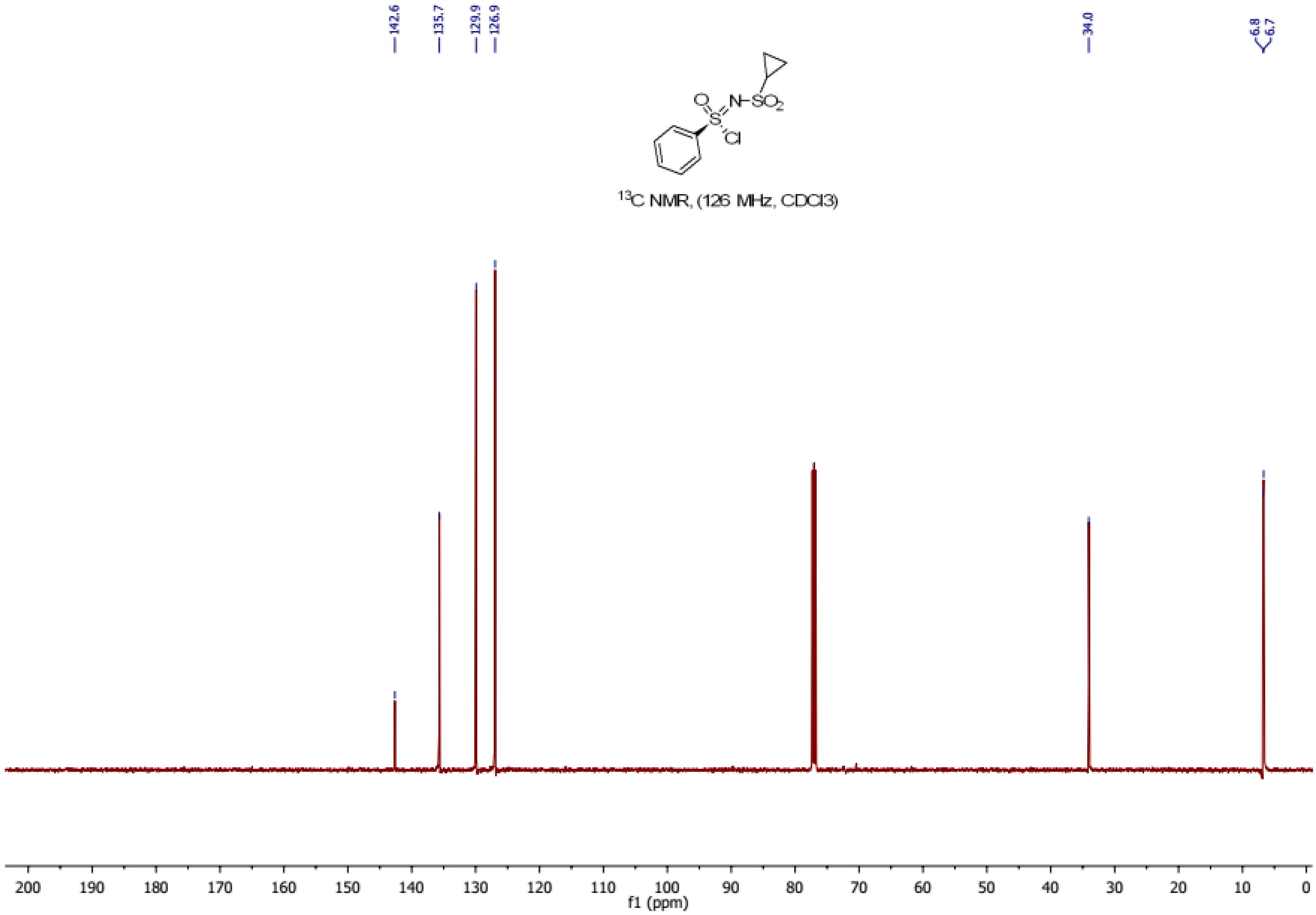

3-Hydroxy-2,2-dimethylpropyl *(R)*-*N*-tosylbenzenesulfonimidate (3a)

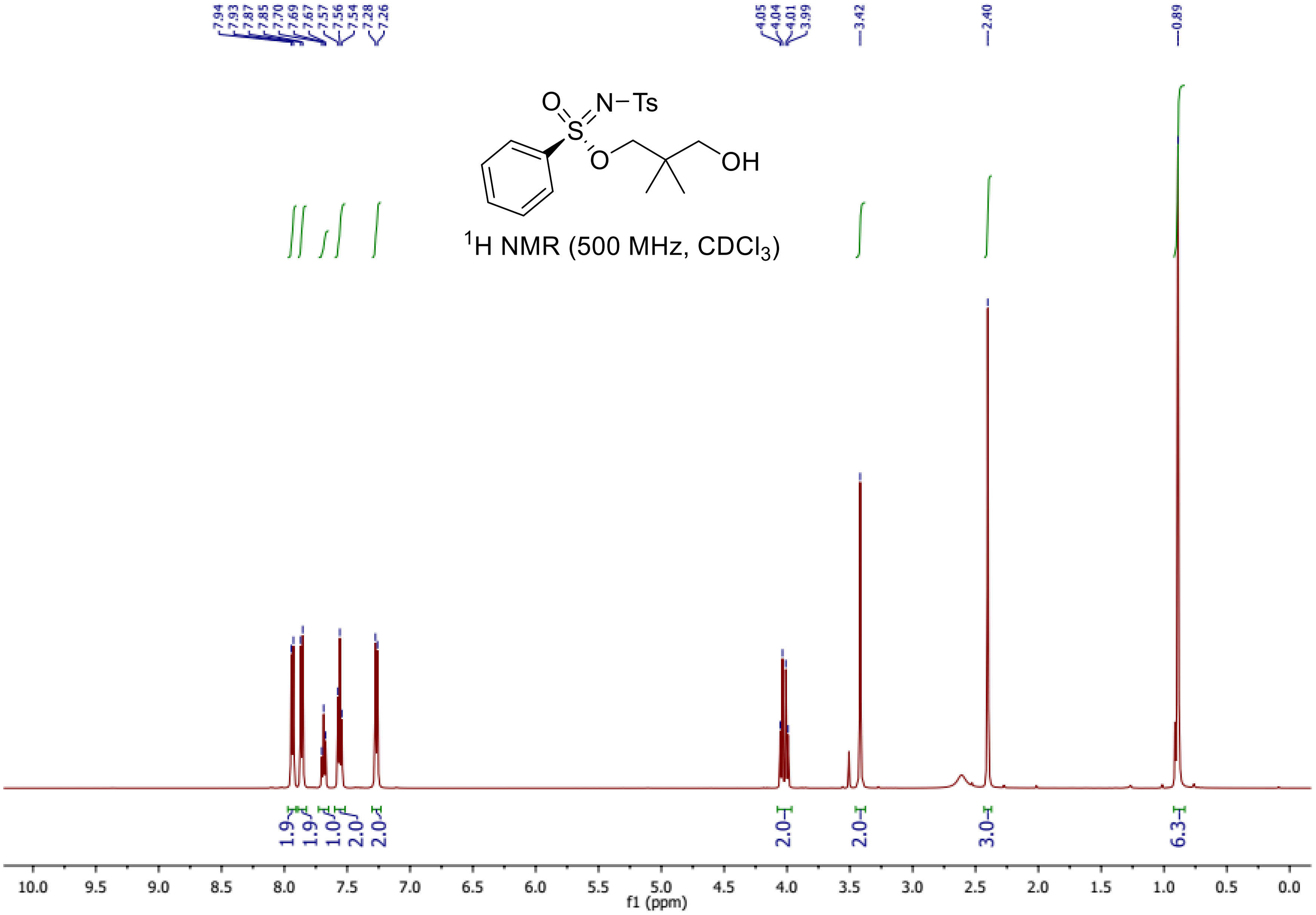

Go to table of contents

3-Hydroxy-2,2-dimethylpropyl *(R)*-*N*-tosylbenzenesulfonimidate (3a)

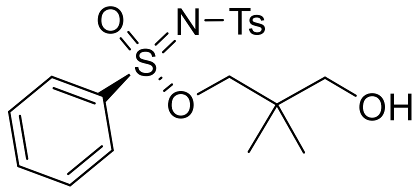

^{13}C NMR (126 MHz, $CDCl_3$)

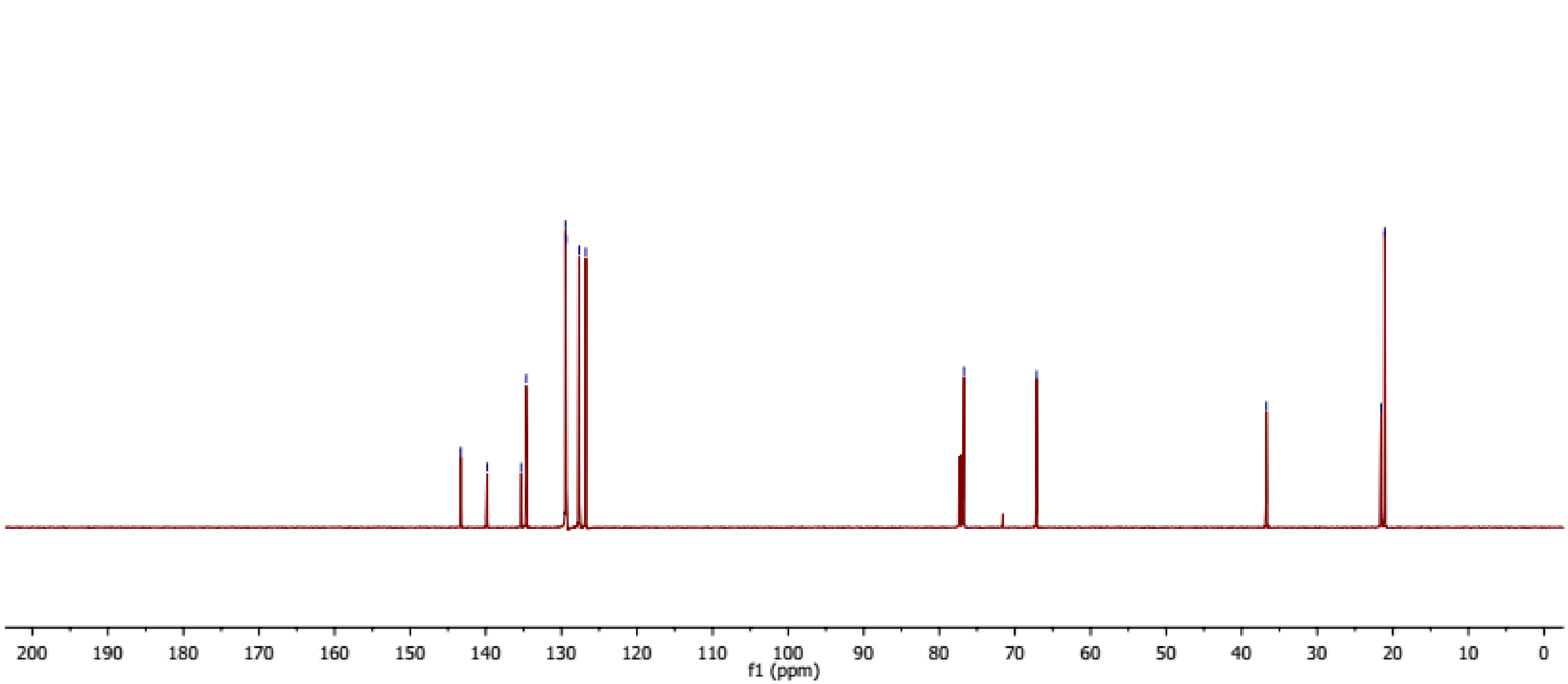

3-Hydroxy-2,2-dimethylpropyl *(R)*-2-fluoro-*N*-tosylbenzenesulfonimidate (3b)

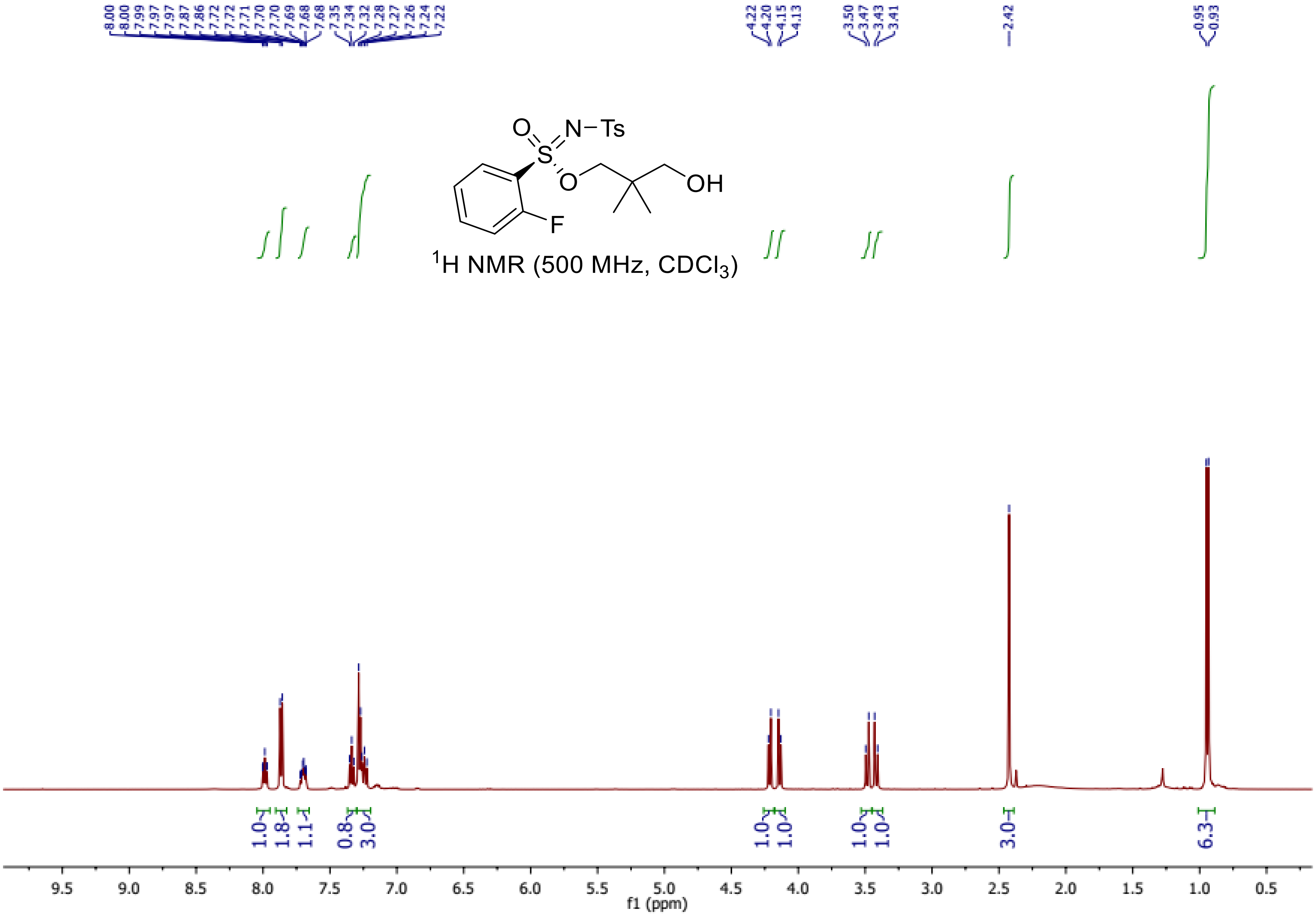

Go to table of contents

3-Hydroxy-2,2-dimethylpropyl *(R)*-2-fluoro-*N*-tosylbenzenesulfonimidate (3b)

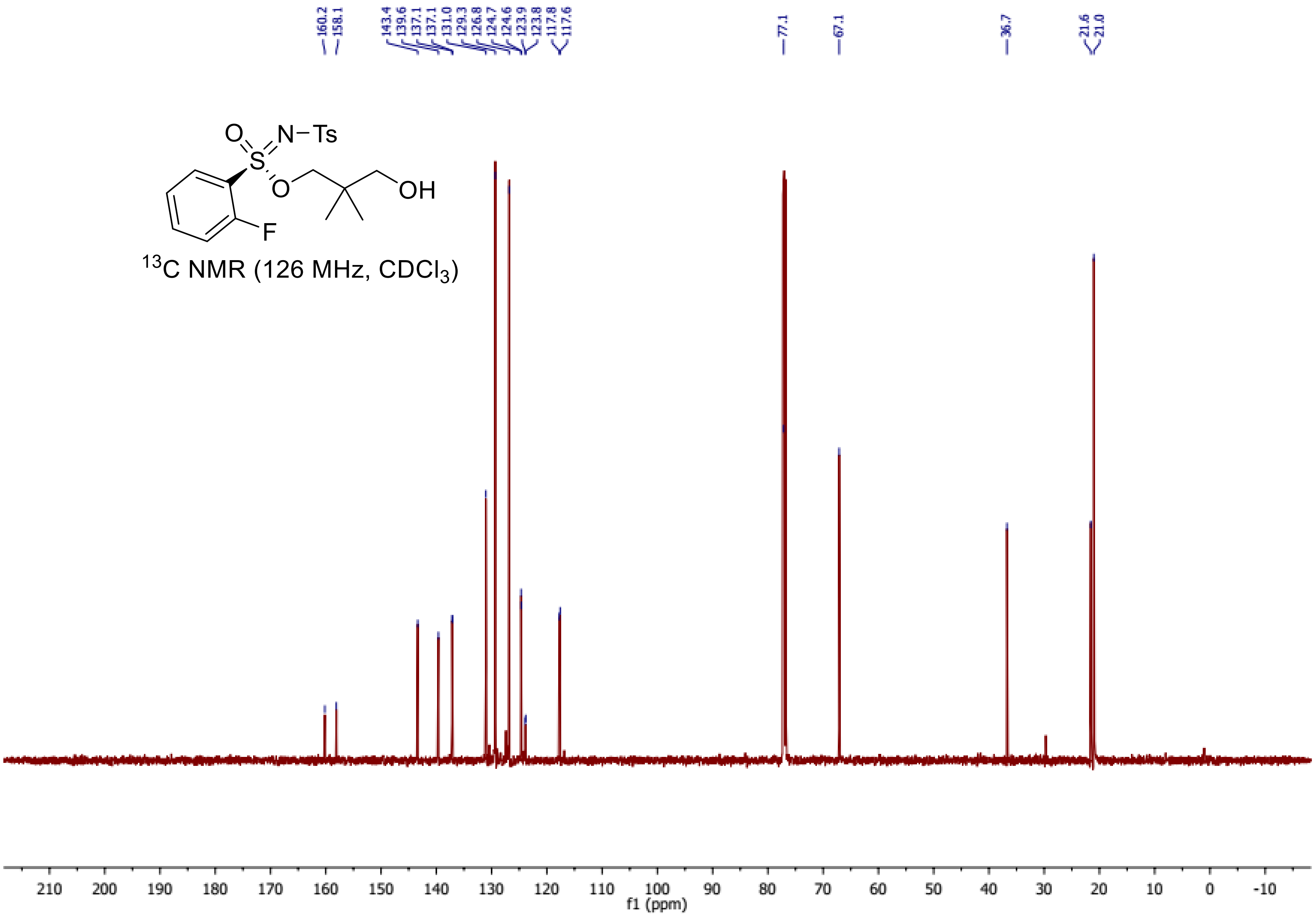

Go to table of contents

3-Hydroxy-2,2-dimethylpropyl *(R)*-4-chloro-*N*-tosylbenzenesulfonimidate (3c)

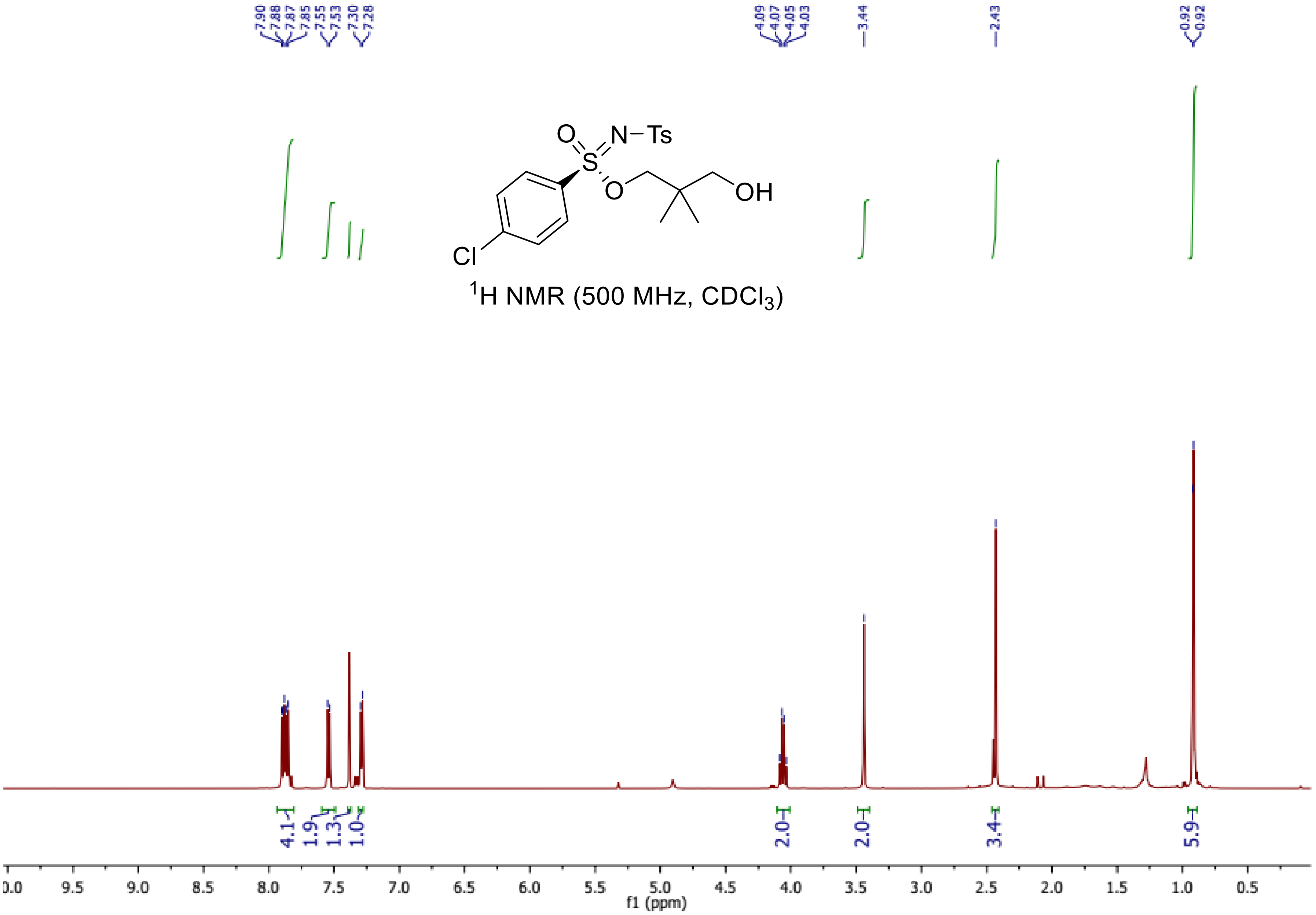

Go to table of contents

3-Hydroxy-2,2-dimethylpropyl *(R)*-4-chloro-*N*-tosylbenzenesulfonimidate (3c)

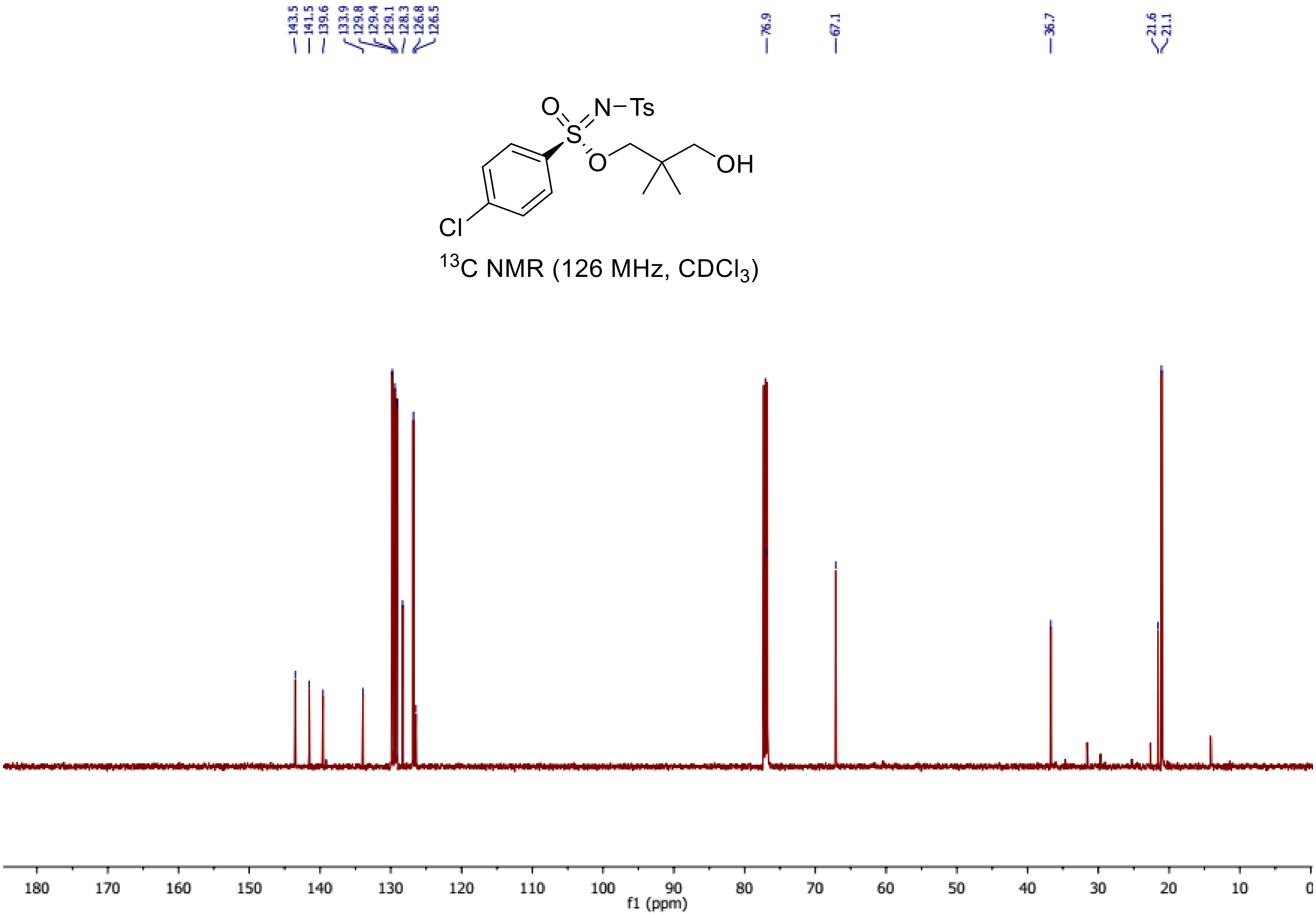

Go to table of contents

3-Hydroxy-2,2-dimethylpropyl (*R*)-4-bromo-*N*-tosylbenzenesulfonimidate (3d)

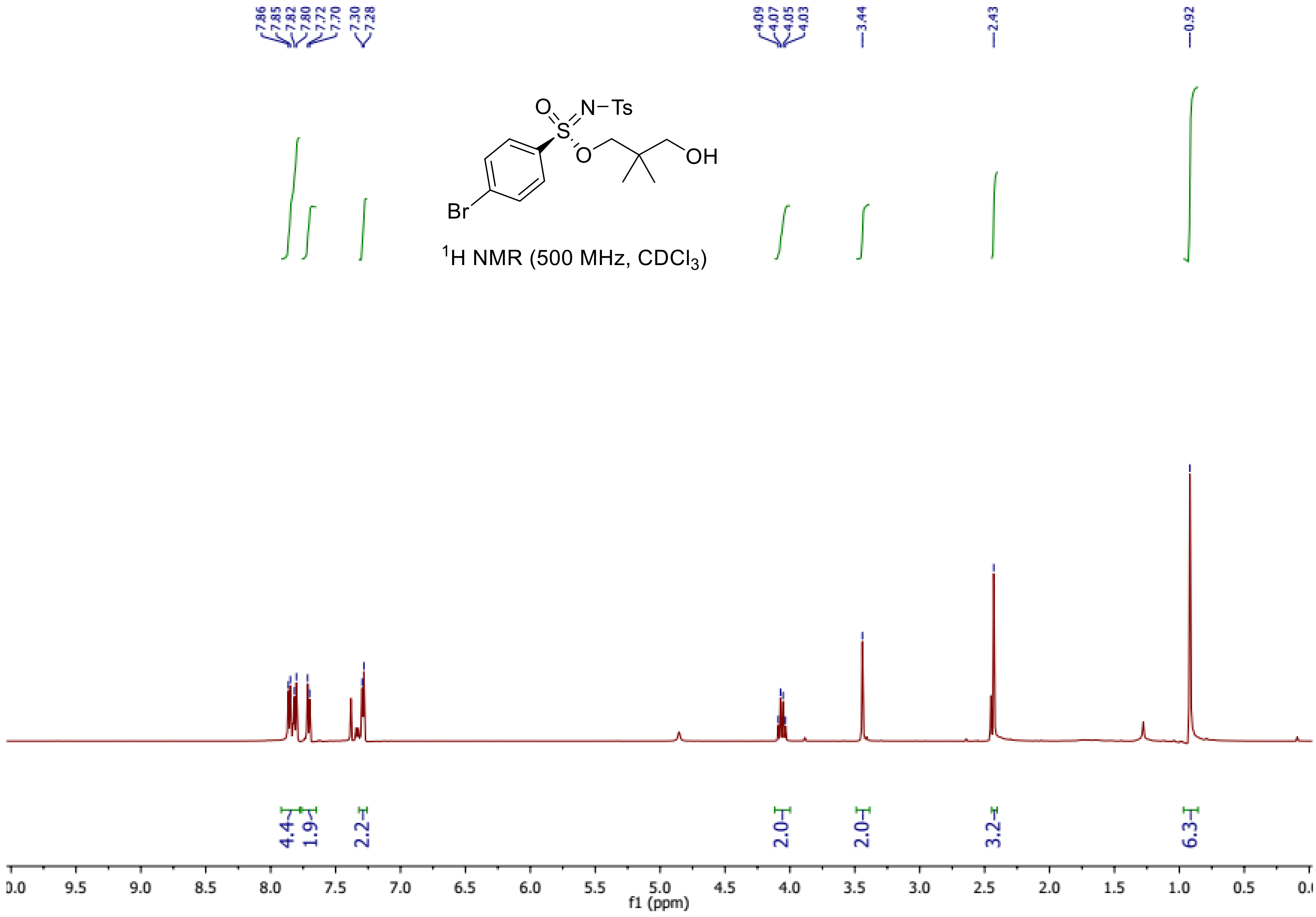

Go to table of contents

3-Hydroxy-2,2-dimethylpropyl *(R)*-4-bromo-*N*-tosylbenzenesulfonimidate (3d)

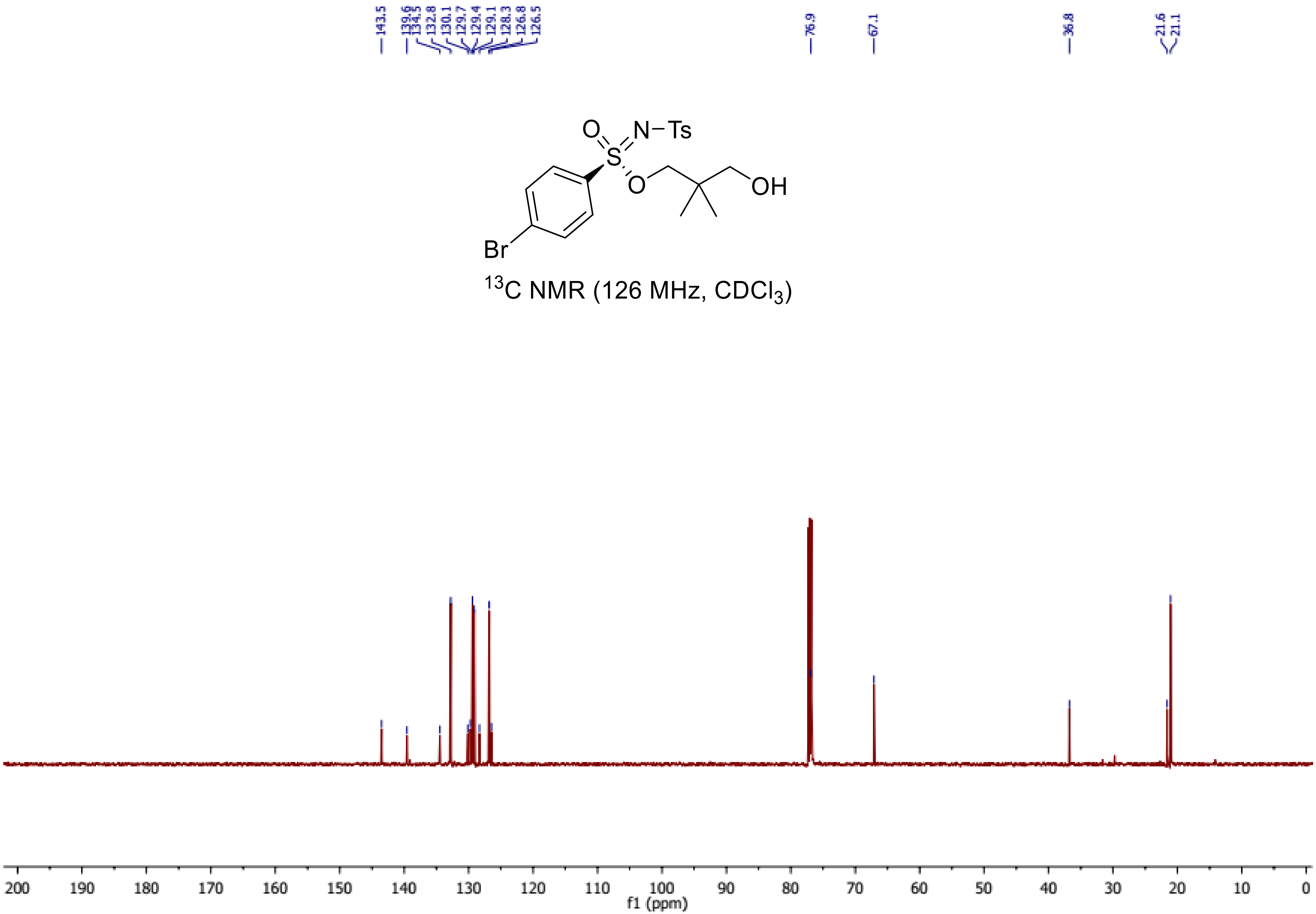

Go to table of contents

3-Hydroxy-2,2-dimethylpropyl (*R*)-4-iodo-*N*-tosylbenzenesulfonimidate (3e)

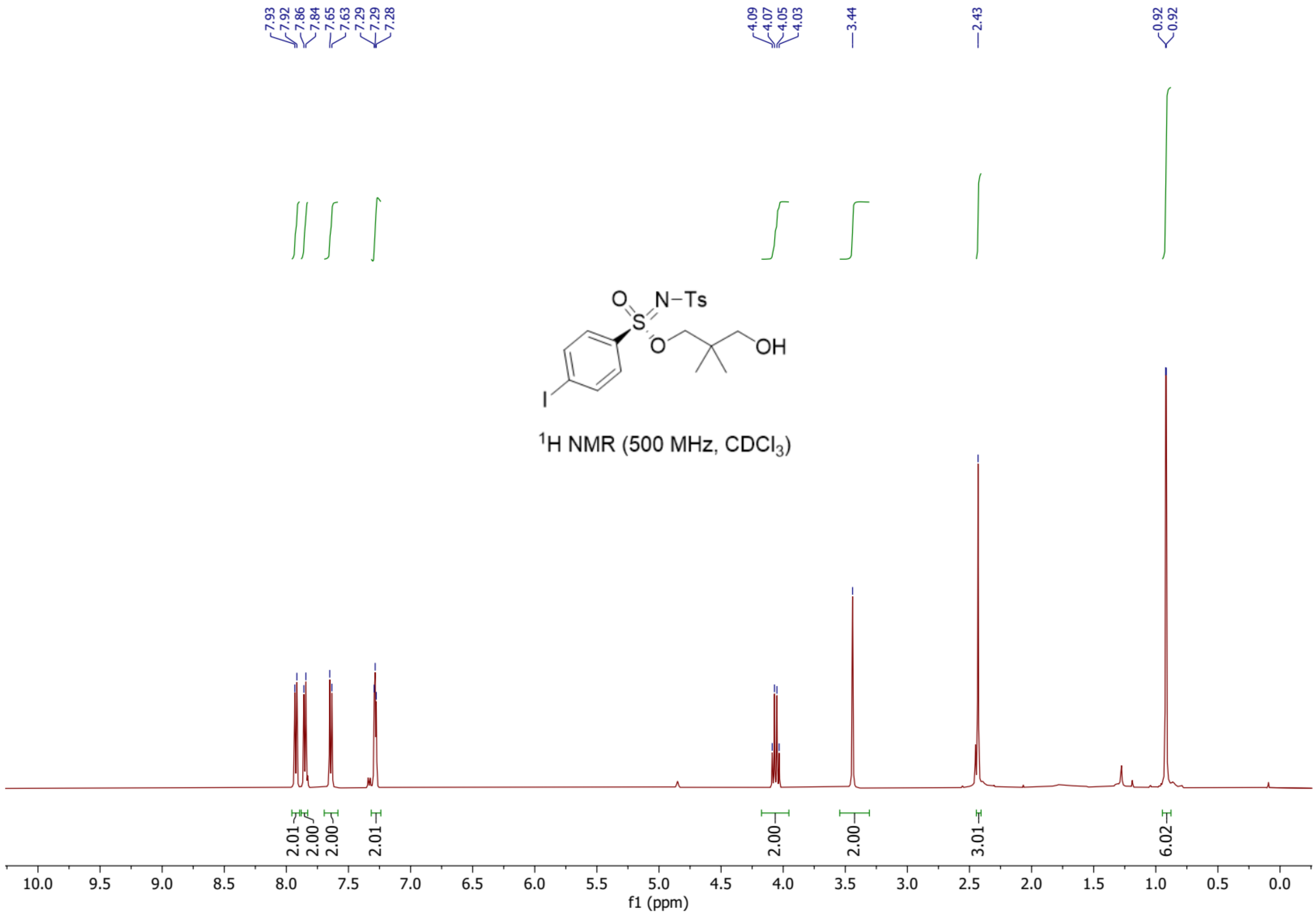

Go to table of contents

3-Hydroxy-2,2-dimethylpropyl (*R*)-4-iodo-*N*-tosylbenzenesulfonimidate (3e)

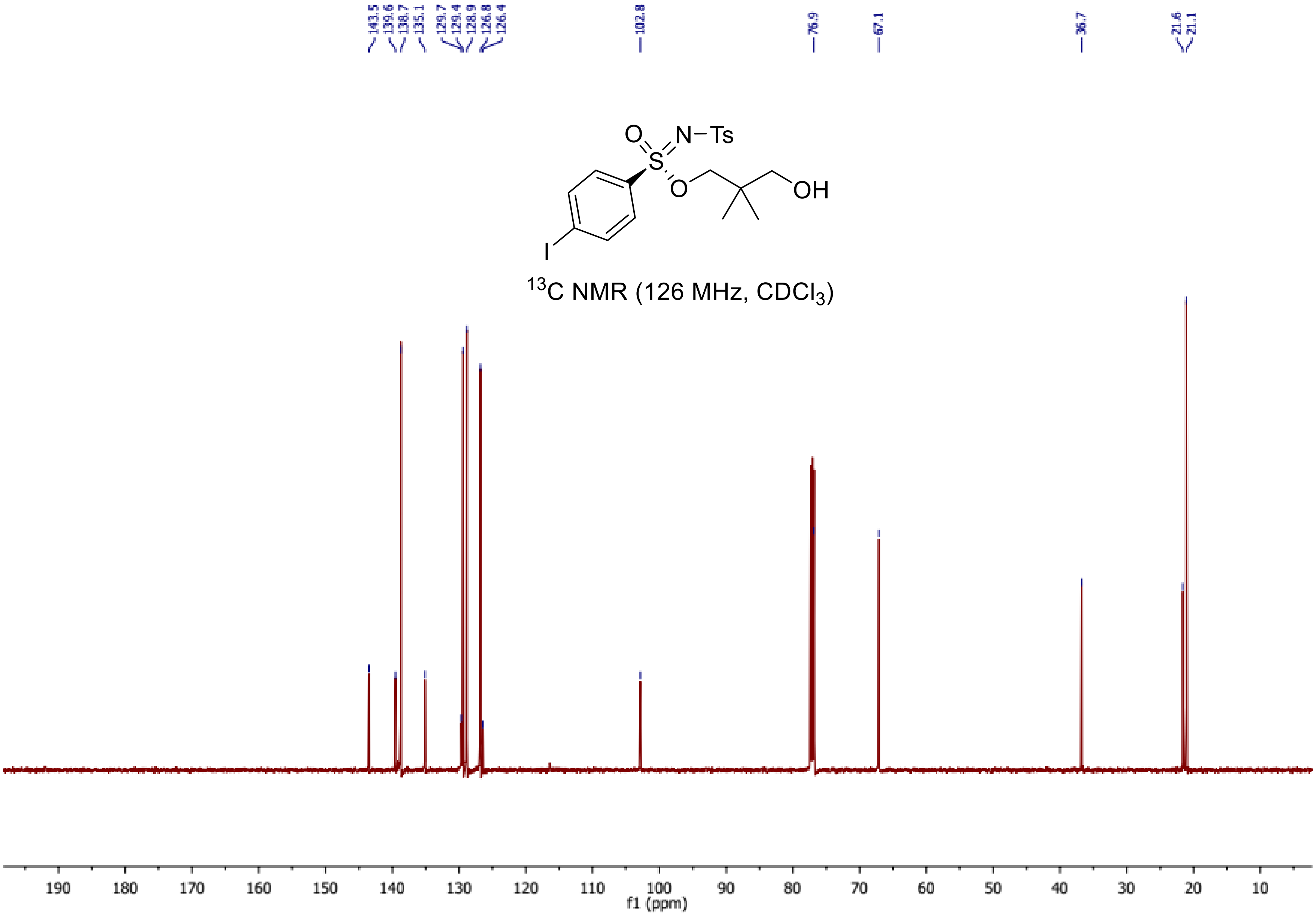

Go to table of contents

3-Hydroxy-2,2-dimethylpropyl *(R)*-4-nitro-*N*-tosylbenzenesulfonimidate (3f)

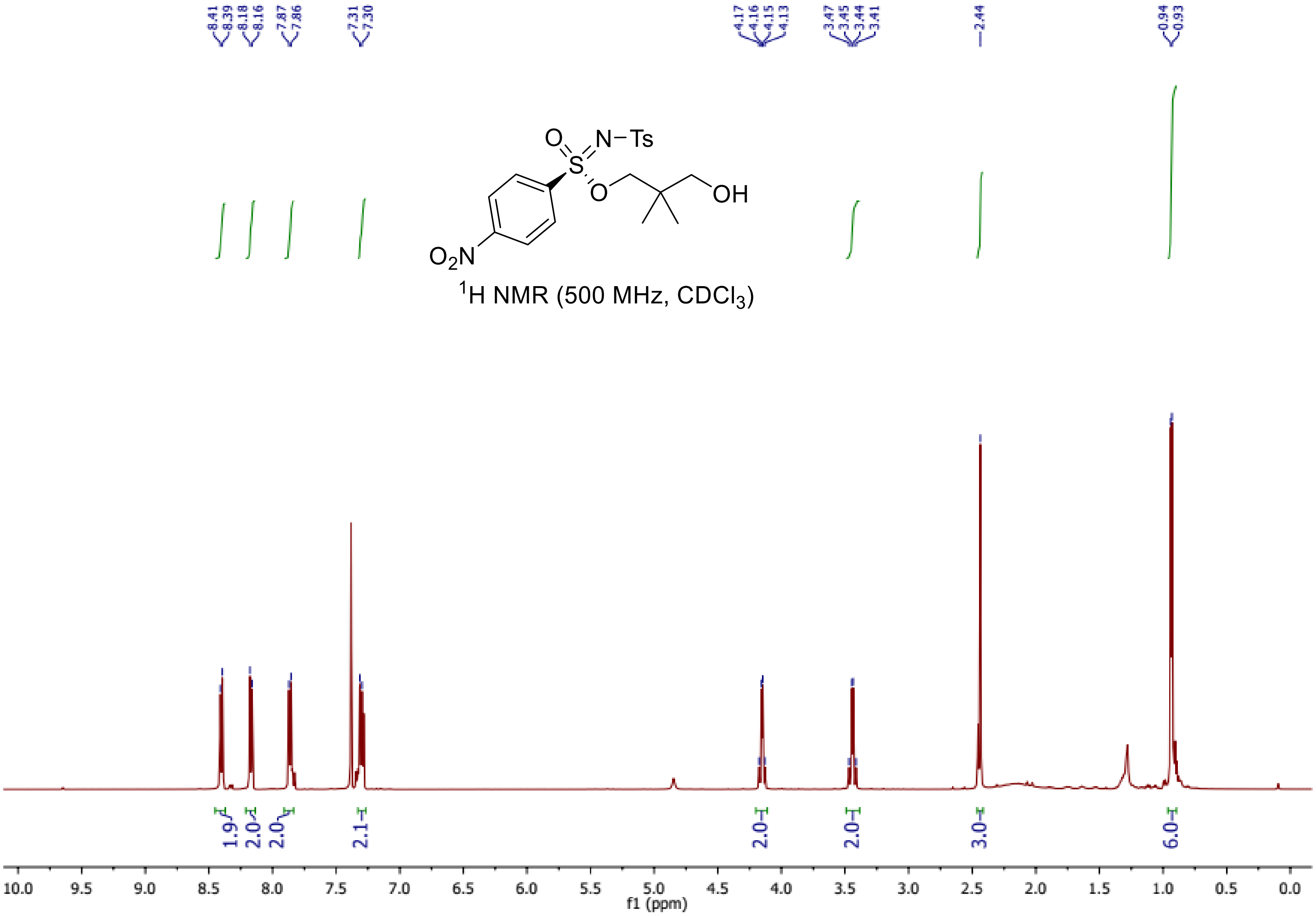

Go to table of contents

3-Hydroxy-2,2-dimethylpropyl *(R)*-4-nitro-*N*-tosylbenzenesulfonimidate (3f)

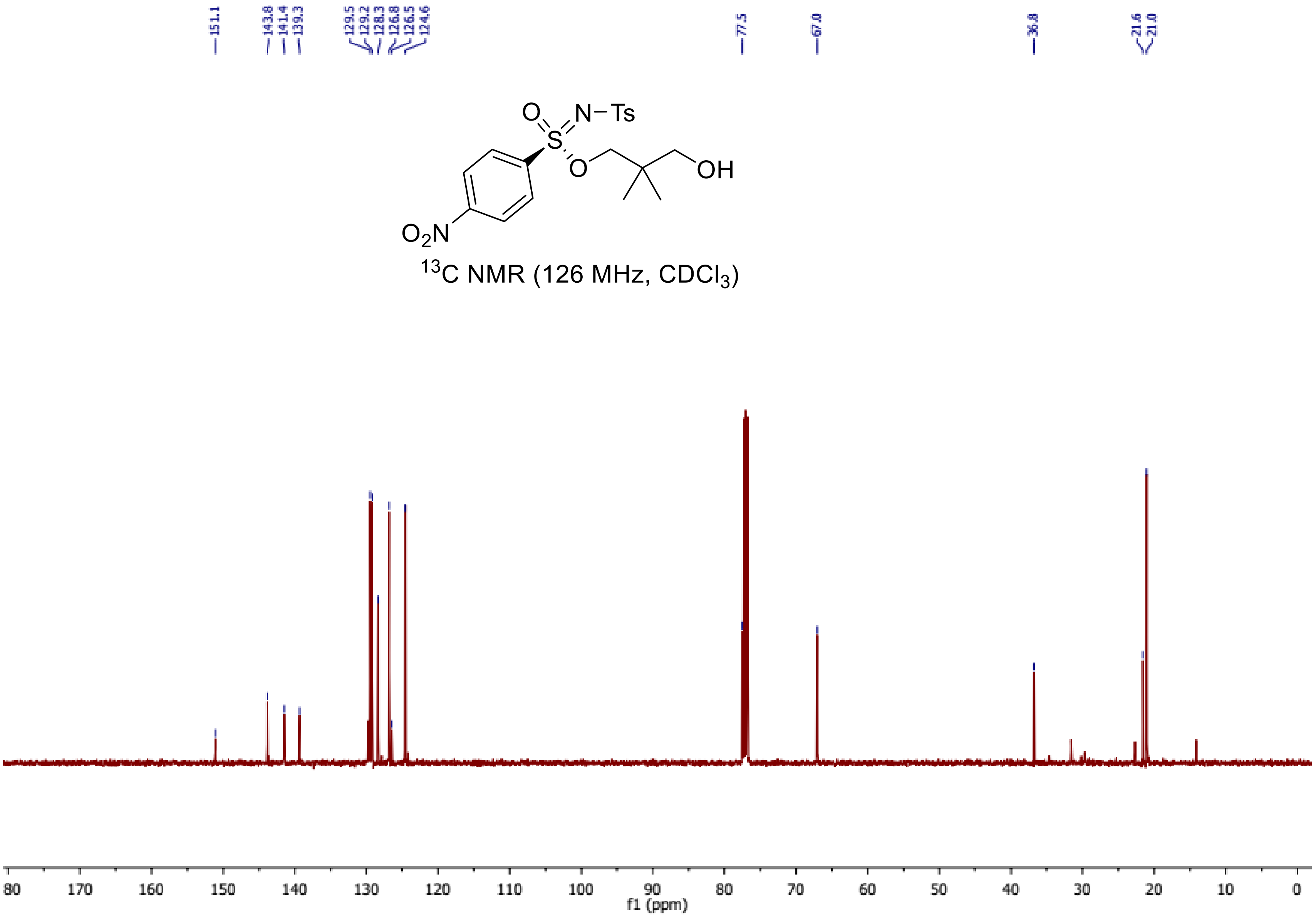

3-Hydroxy-2,2-dimethylpropyl (*R*)-4-methoxy-*N*-tosylbenzenesulfonimidate (3g)

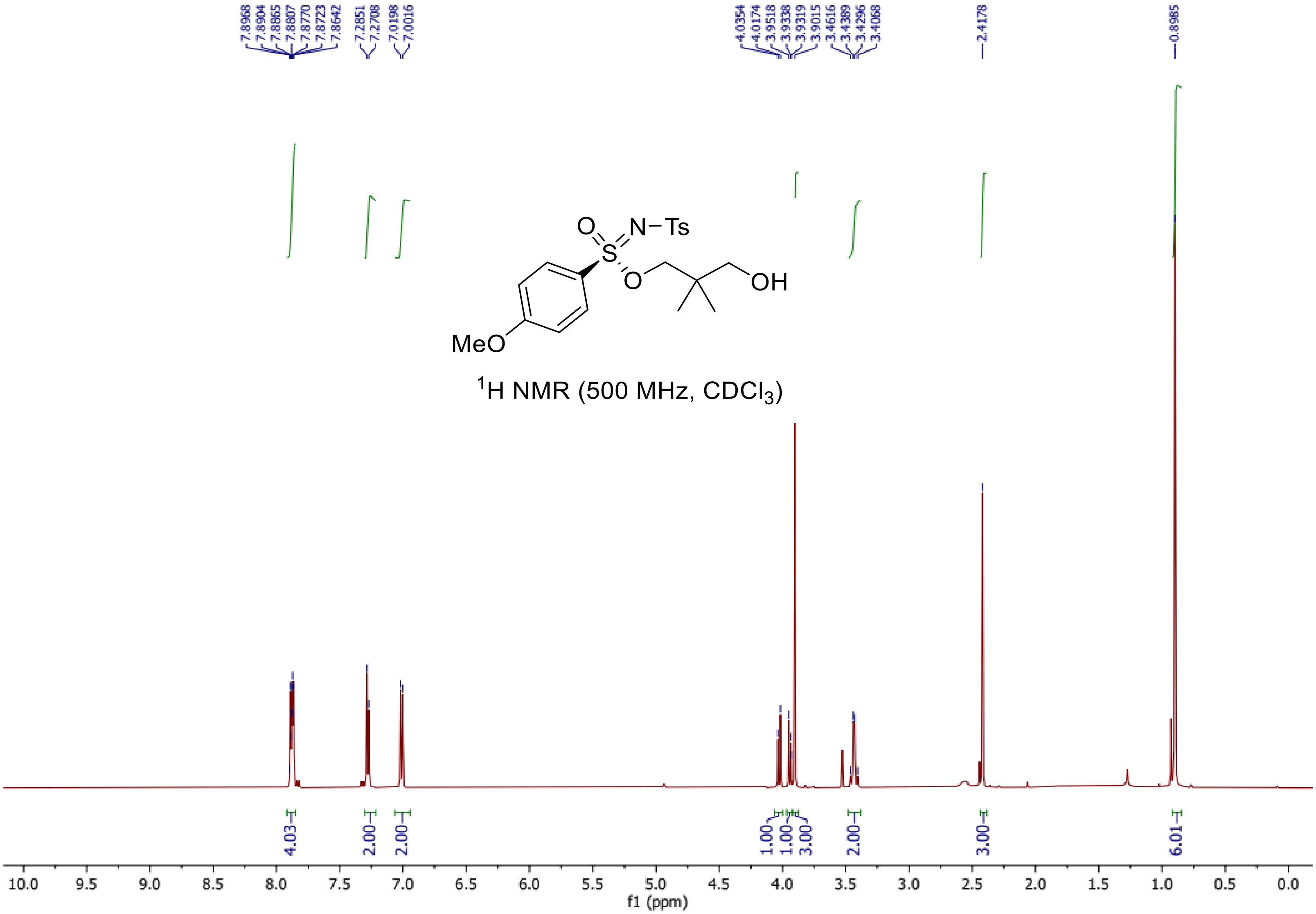

Go to table of contents

3-Hydroxy-2,2-dimethylpropyl (*R*)-4-methoxy-*N*-tosylbenzenesulfonimidate (3g)

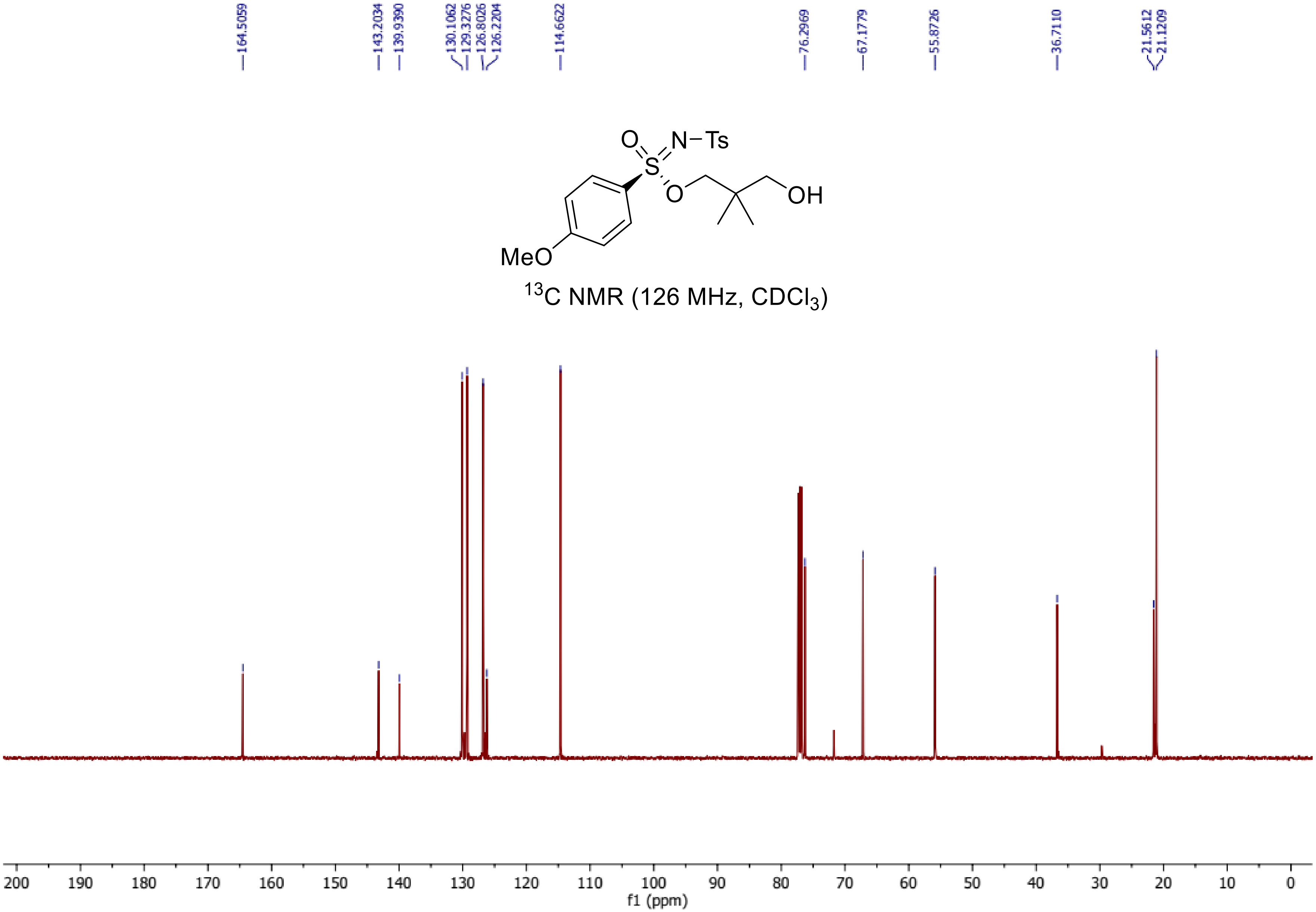

Go to table of contents

3-Hydroxy-2,2-dimethylpropyl (*R*)-4-(difluoromethoxy)-*N*-tosylbenzenesulfonimidate (3h)

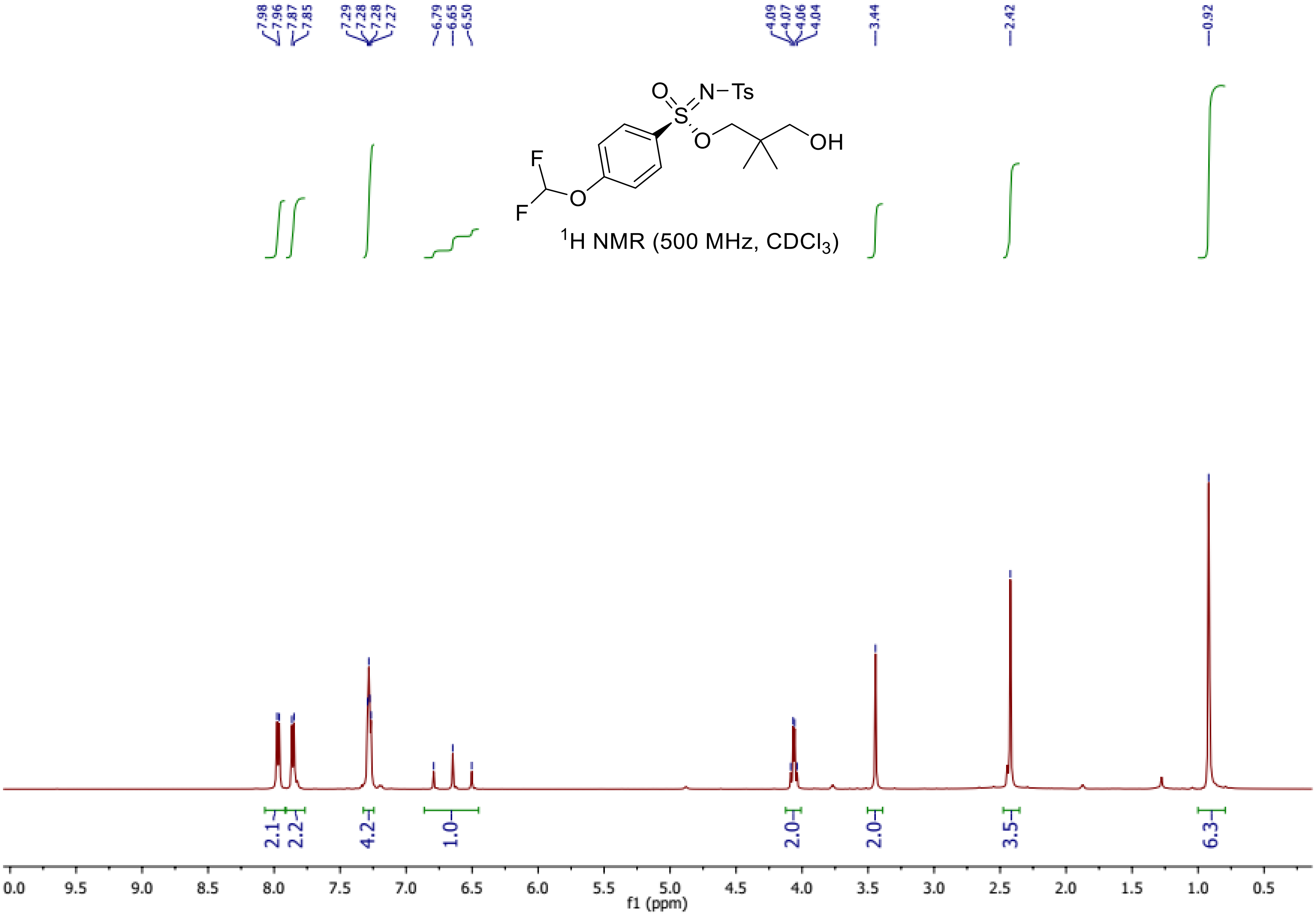

Go to table of contents

3-Hydroxy-2,2-dimethylpropyl (*R*)-4-(difluoromethoxy)-*N*-tosylbenzenesulfonimidate (3h)

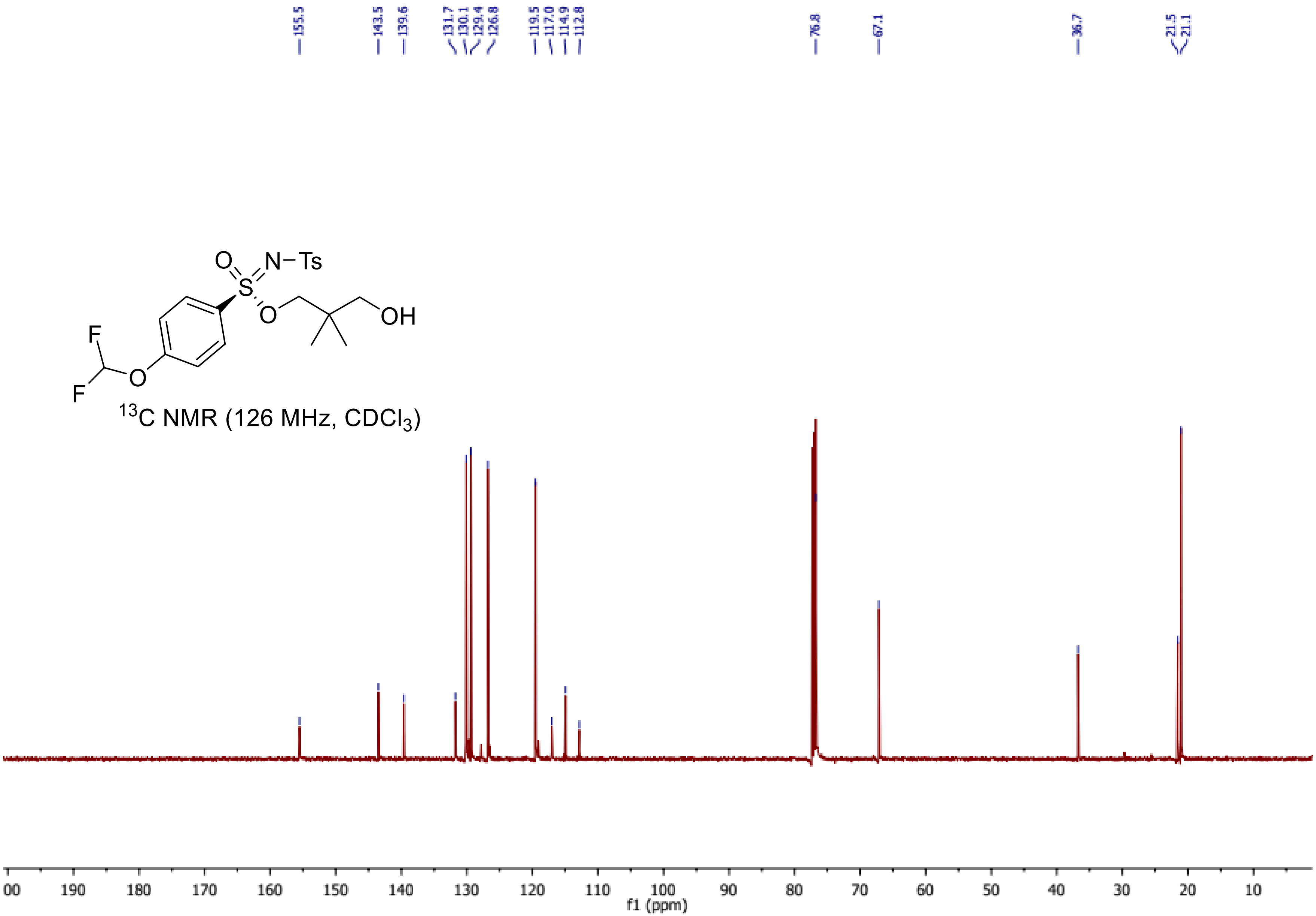

3-Hydroxy-2,2-dimethylpropyl *(R)*-*N*-tosyl-3-(trifluoromethoxy)benzenesulfonimidate (3i)

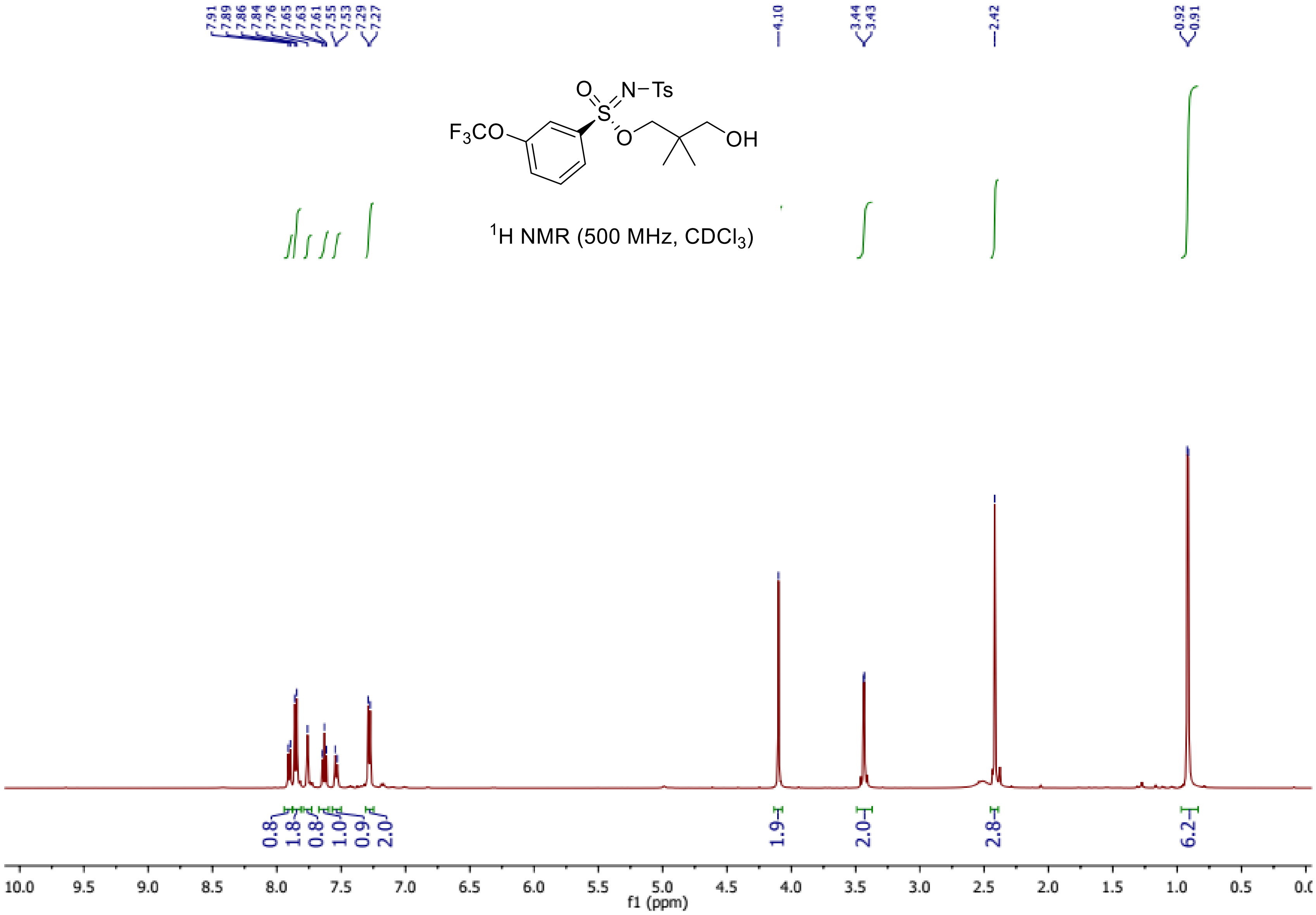

Go to table of contents

3-Hydroxy-2,2-dimethylpropyl *(R)*-*N*-tosyl-3-(trifluoromethoxy)benzenesulfonimidate (3i)

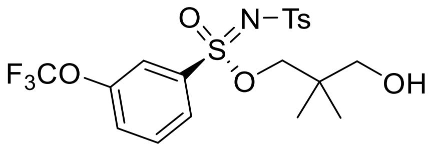

^{13}C NMR (126 MHz, $CDCl_3$)

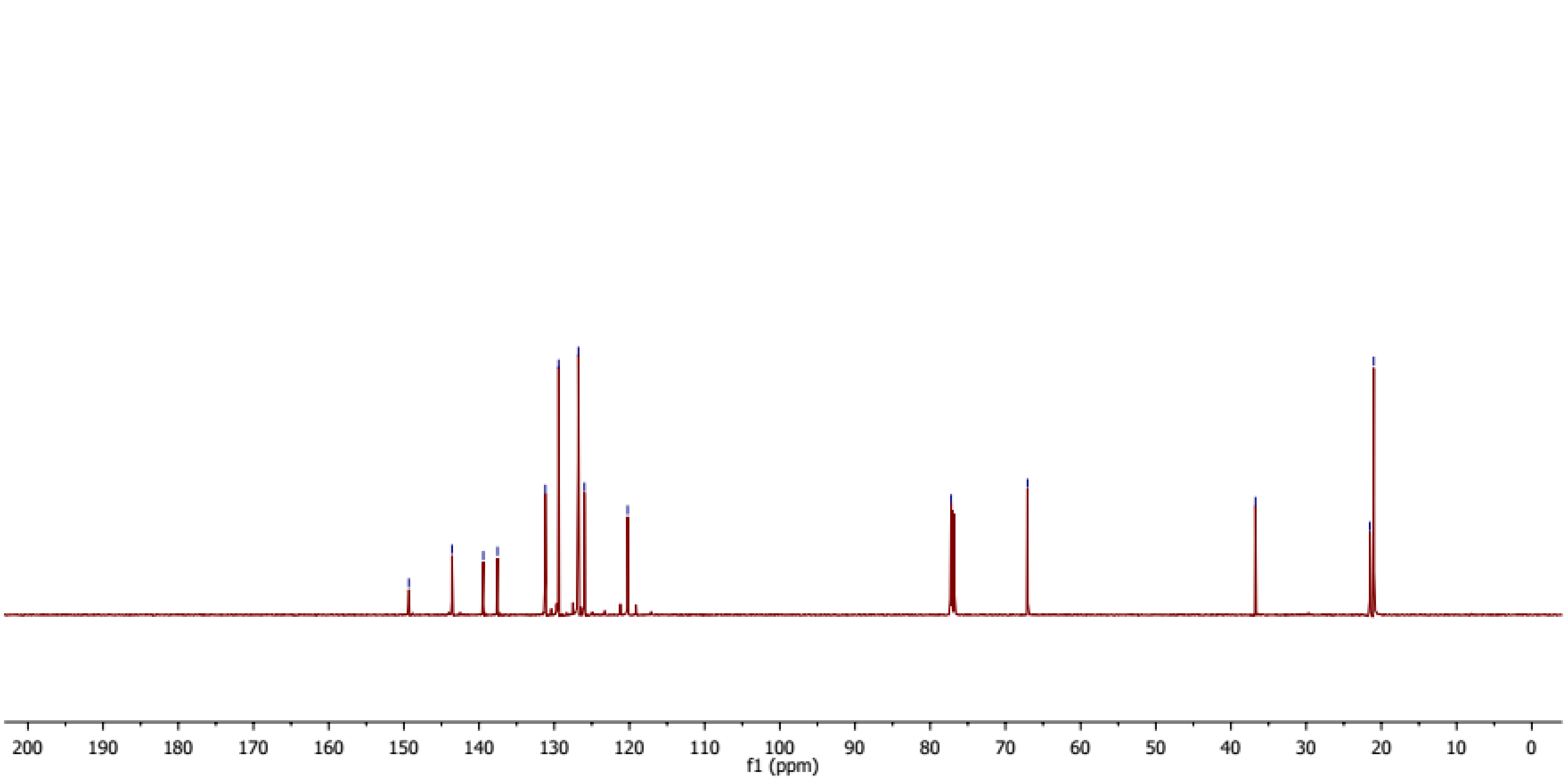

Go to table of contents

3-Hydroxy-2,2-dimethylpropyl *(R)-3-*(methylsulfonyl)-*N*-tosylbenzenesulfonimidate (3j)

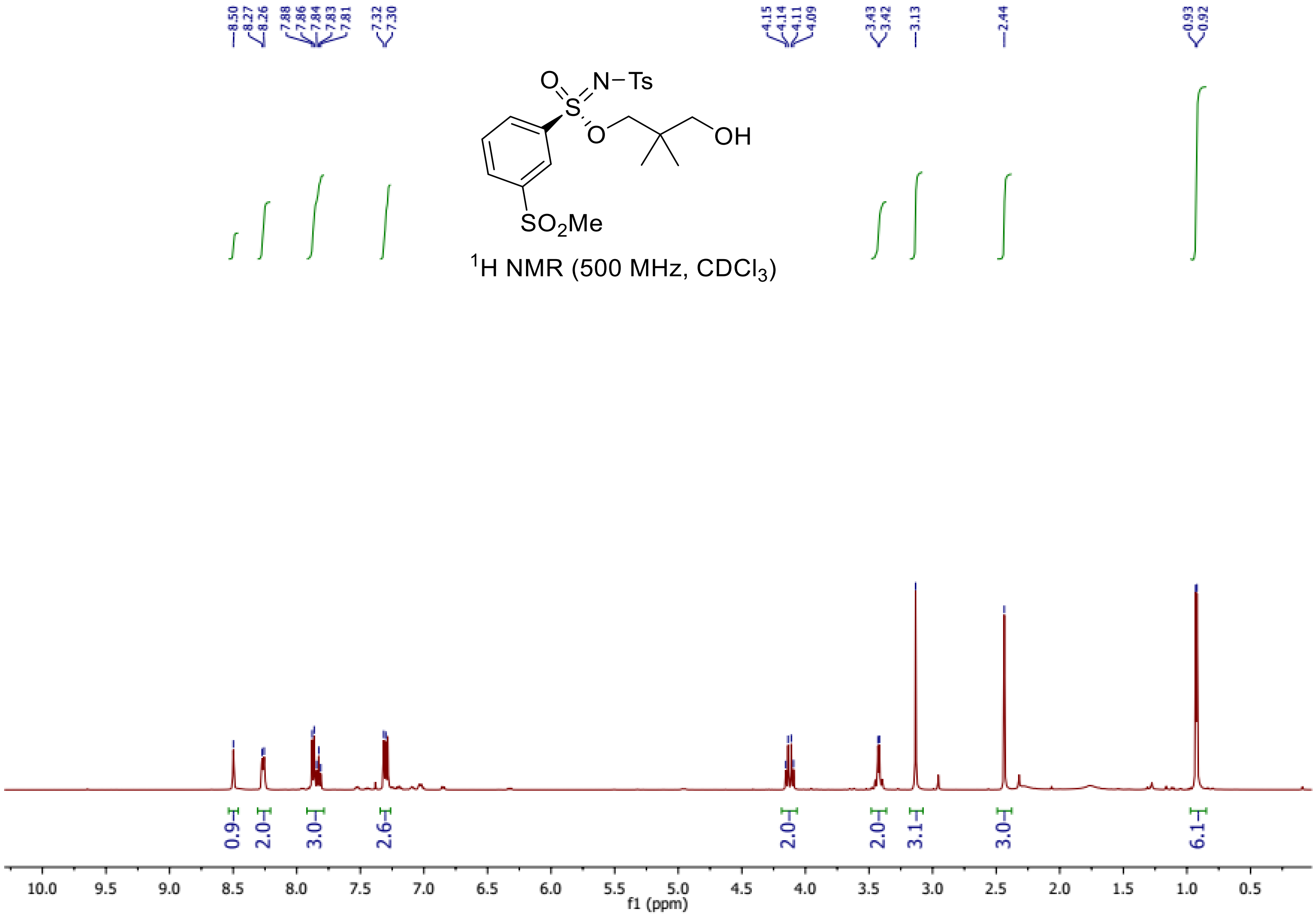

Go to table of contents

3-Hydroxy-2,2-dimethylpropyl (*R*)-3-(methylsulfonyl)-*N*-tosylbenzenesulfonimidate (3j)

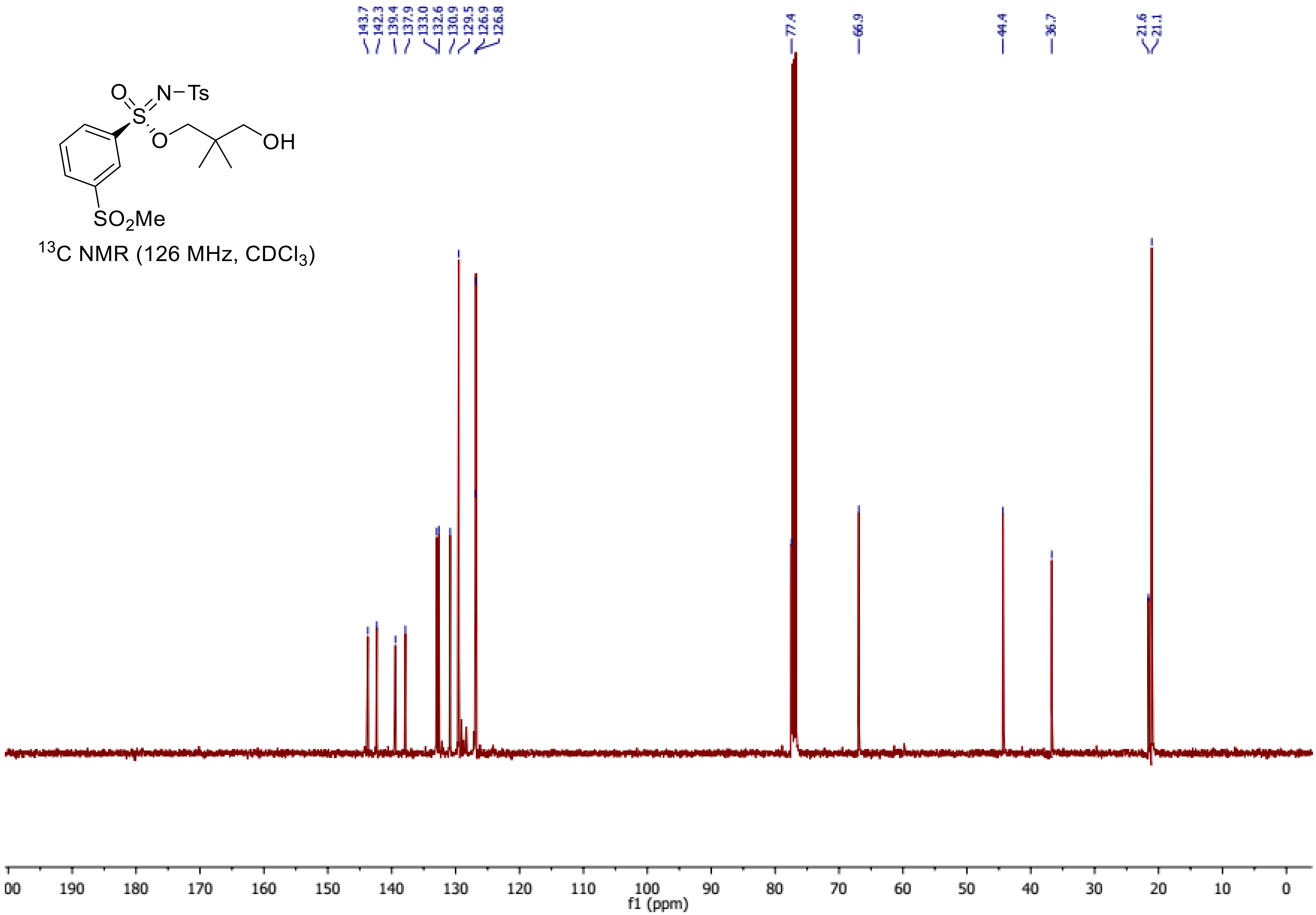

3-Hydroxy-2,2-dimethylpropyl (*R*)-4-cyano-*N*-tosylbenzenesulfonimidate (3k)

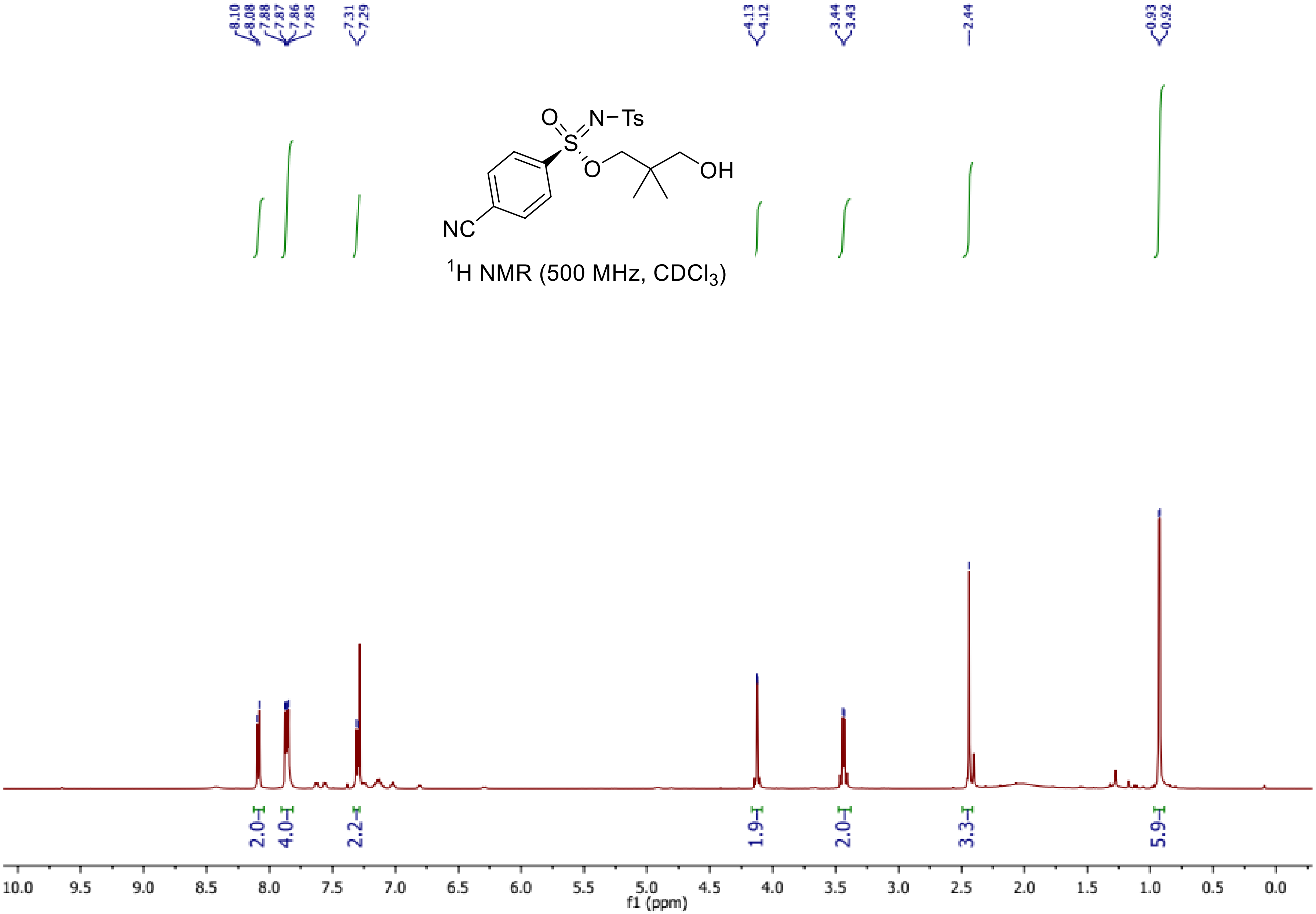

Go to table of contents

3-Hydroxy-2,2-dimethylpropyl (*R*)-4-cyano-*N*-tosylbenzenesulfonimidate (3k)

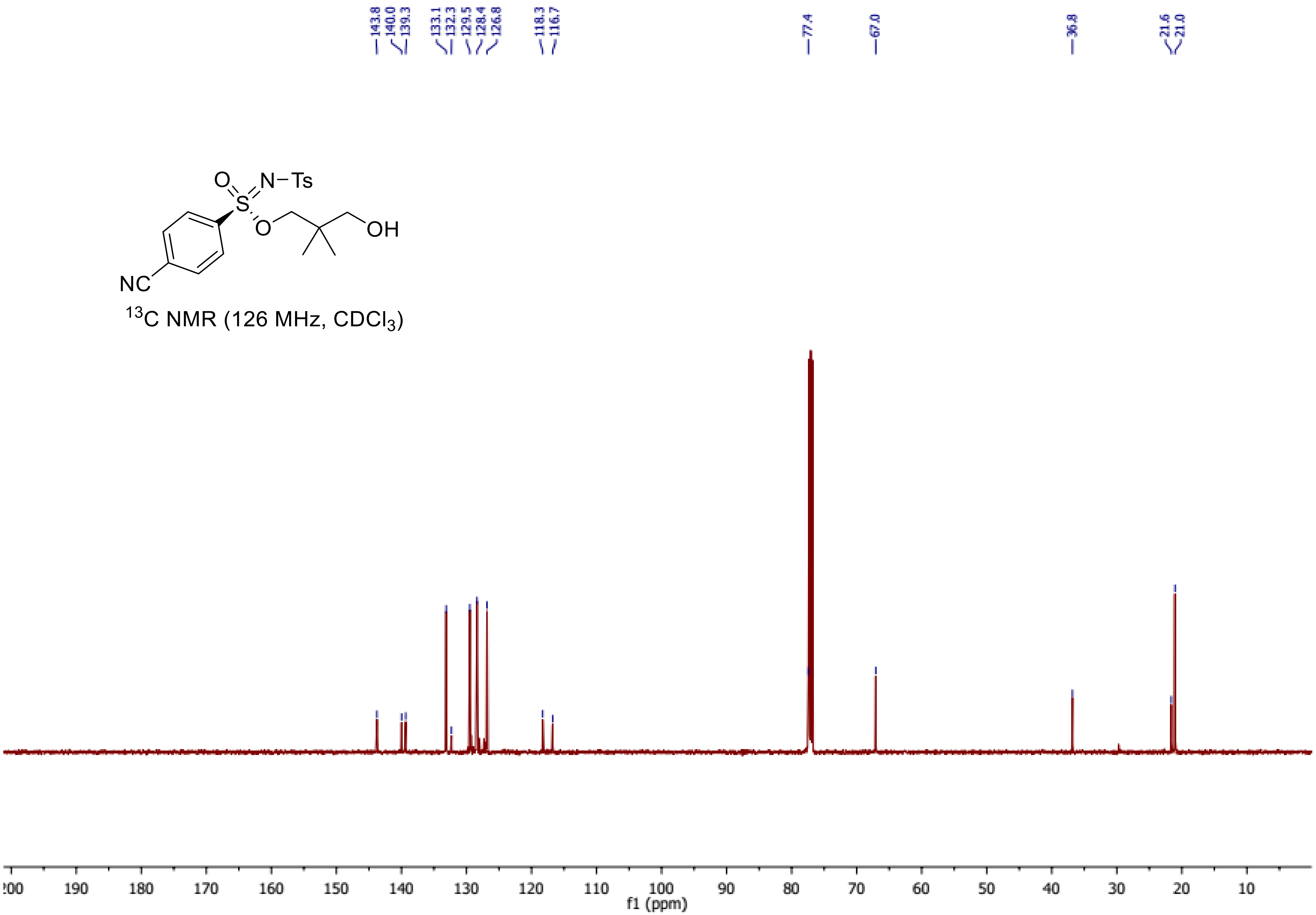

Go to table of contents

3-Hydroxy-2,2-dimethylpropyl *(R)*-4-methyl-*N*-tosylbenzenesulfonimidate (3l)

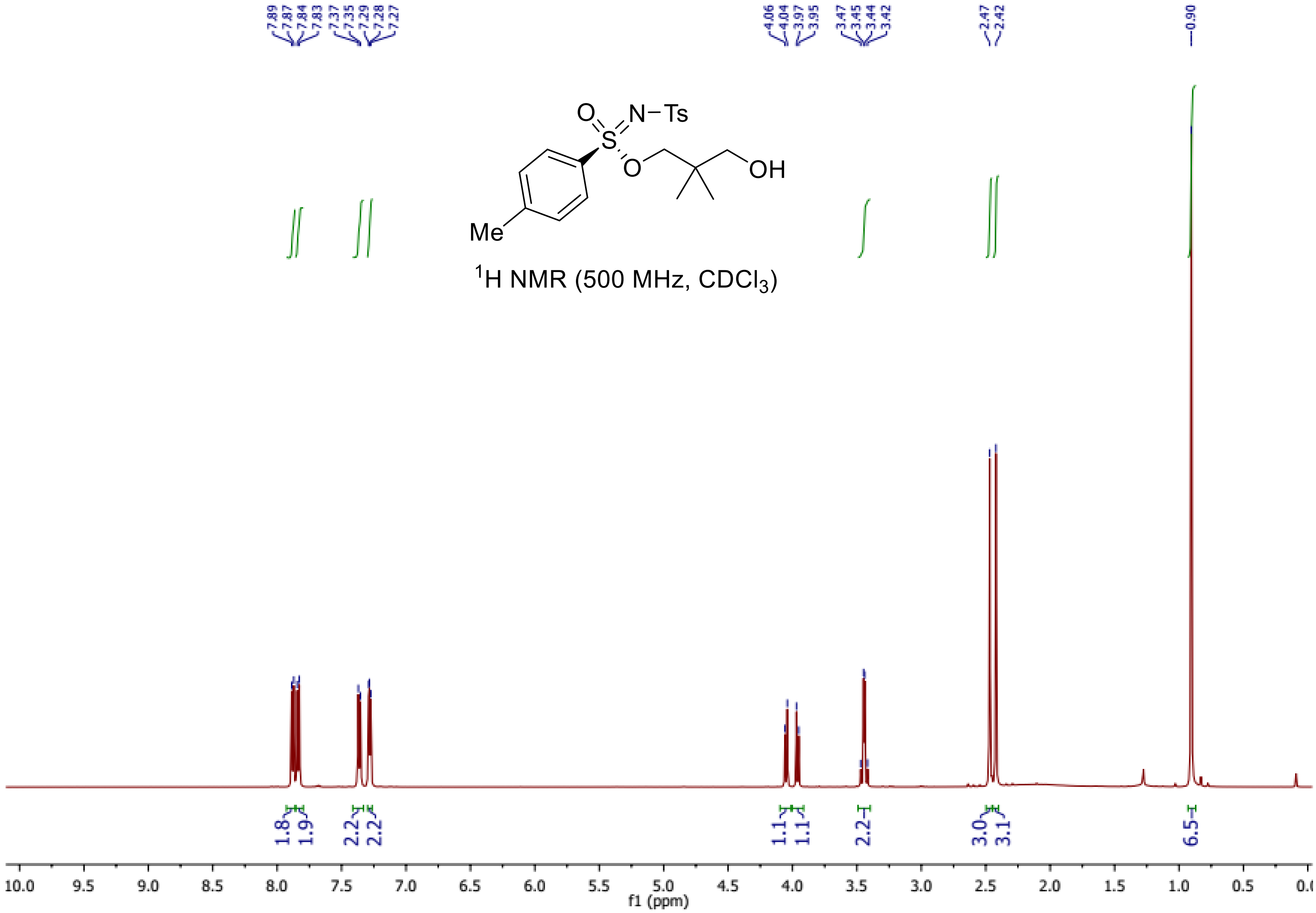

Go to table of contents

3-Hydroxy-2,2-dimethylpropyl *(R)*-4-methyl-*N*-tosylbenzenesulfonimidate (3l)

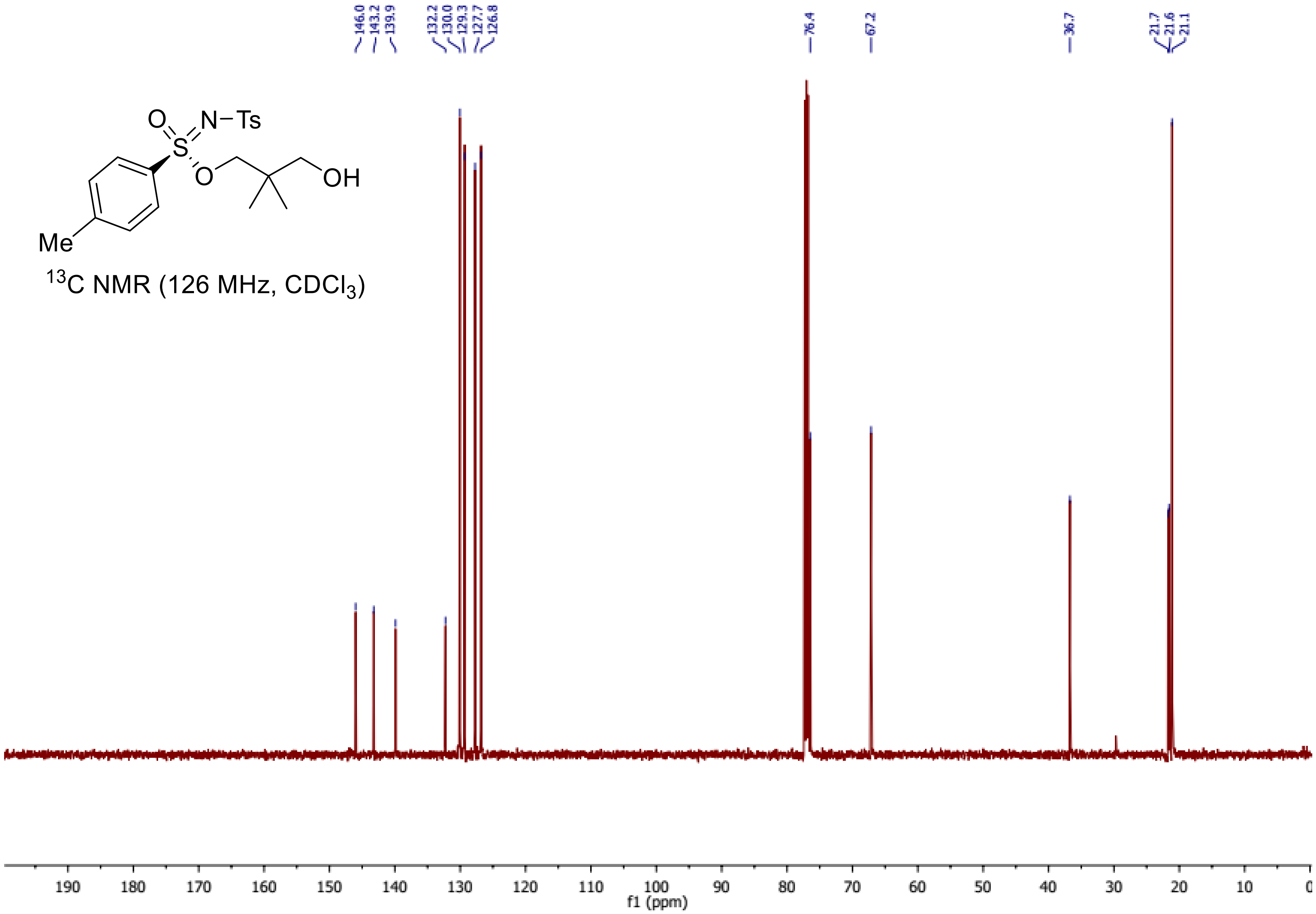

Go to table of contents

3-Hydroxy-2,2-dimethylpropyl (*R*)-4-(*tert*-butyl)-*N*-tosylbenzenesulfonimidate (3m)

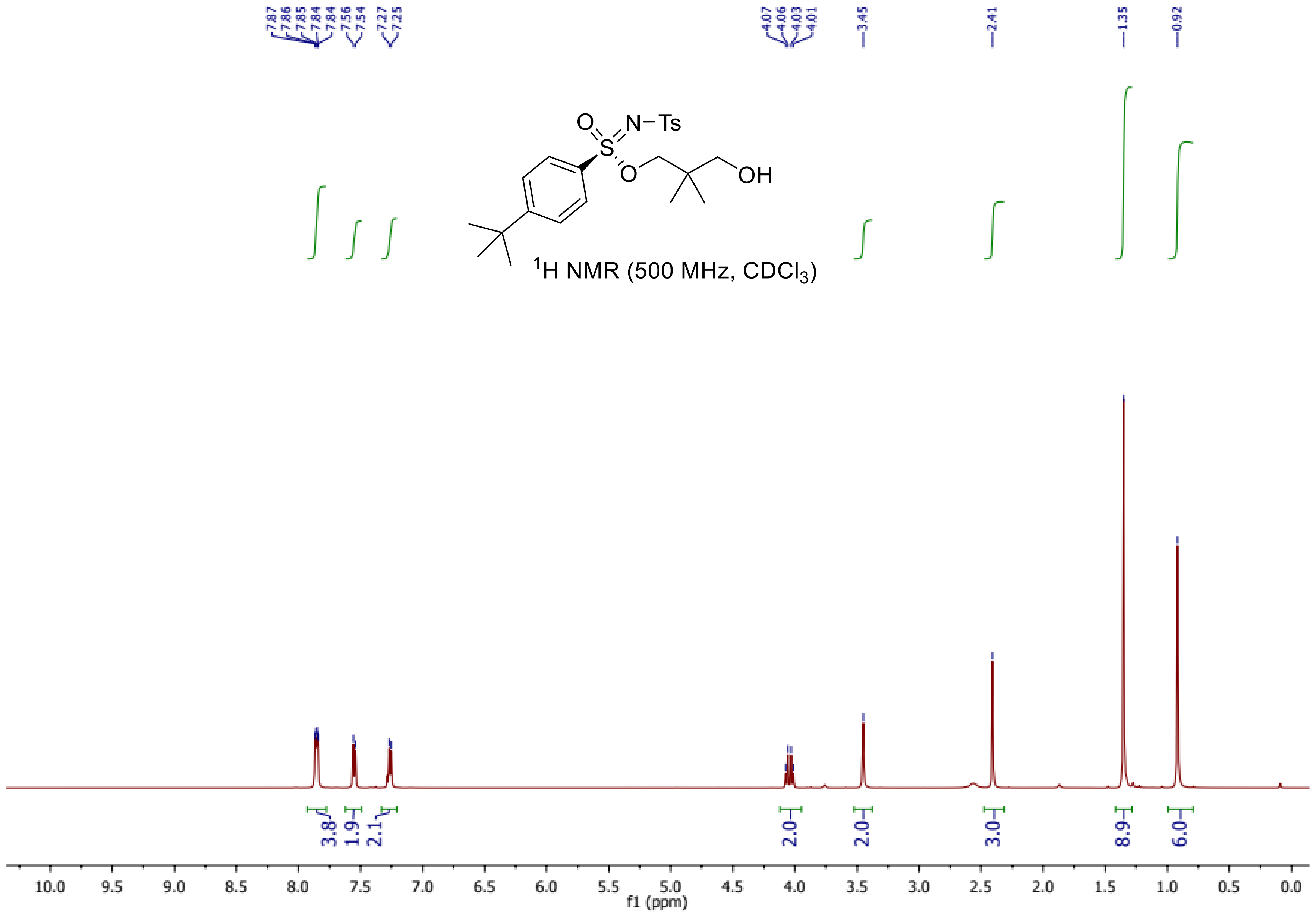

Go to table of contents

3-Hydroxy-2,2-dimethylpropyl (*R*)-4-(*tert*-butyl)-*N*-tosylbenzenesulfonimidate (3m)

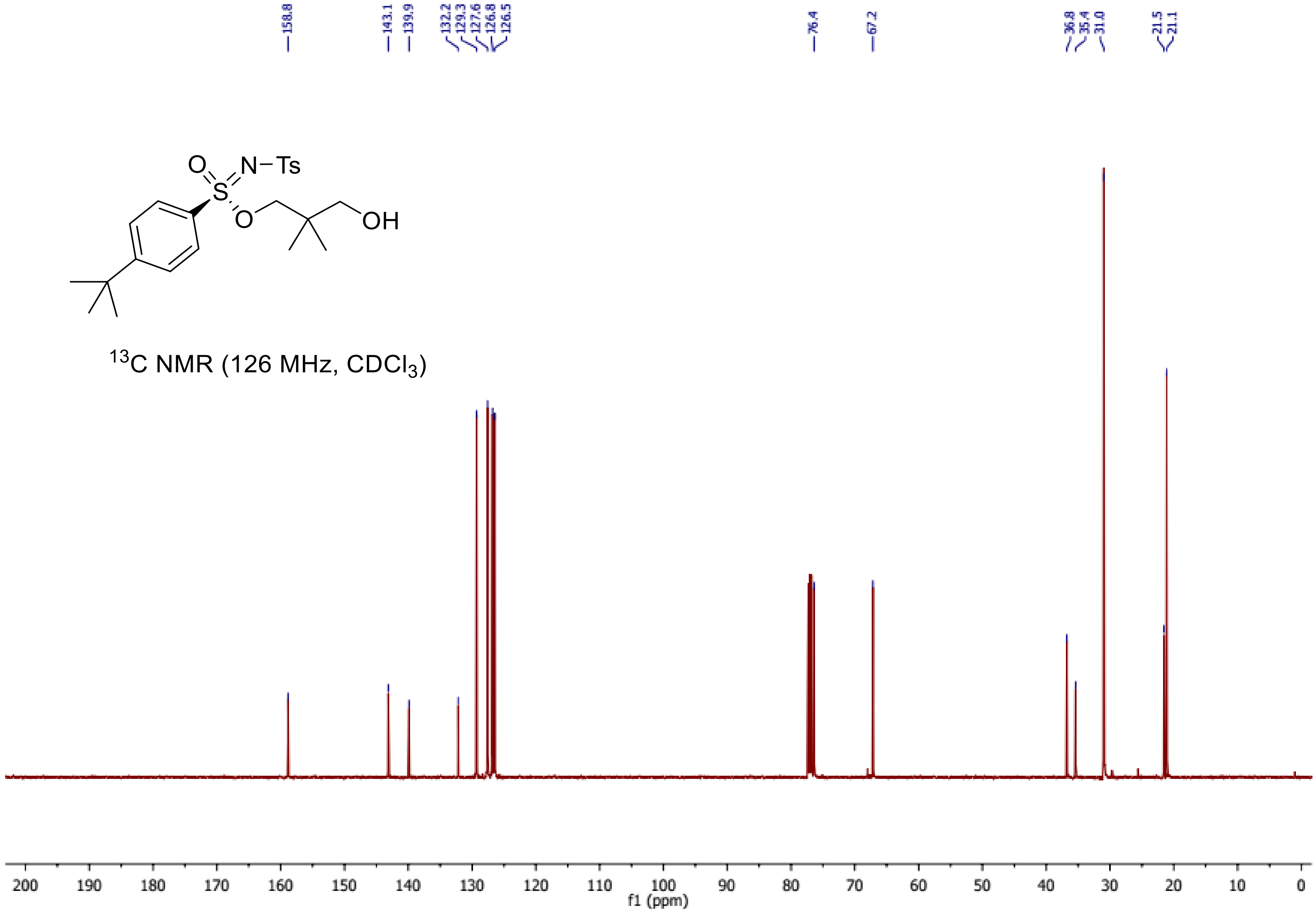

Go to table of contents

3-Hydroxy-2,2-dimethylpropyl (*R*)-*N*-tosyl-[1,1'-biphenyl]-4-sulfonimidate (3n)

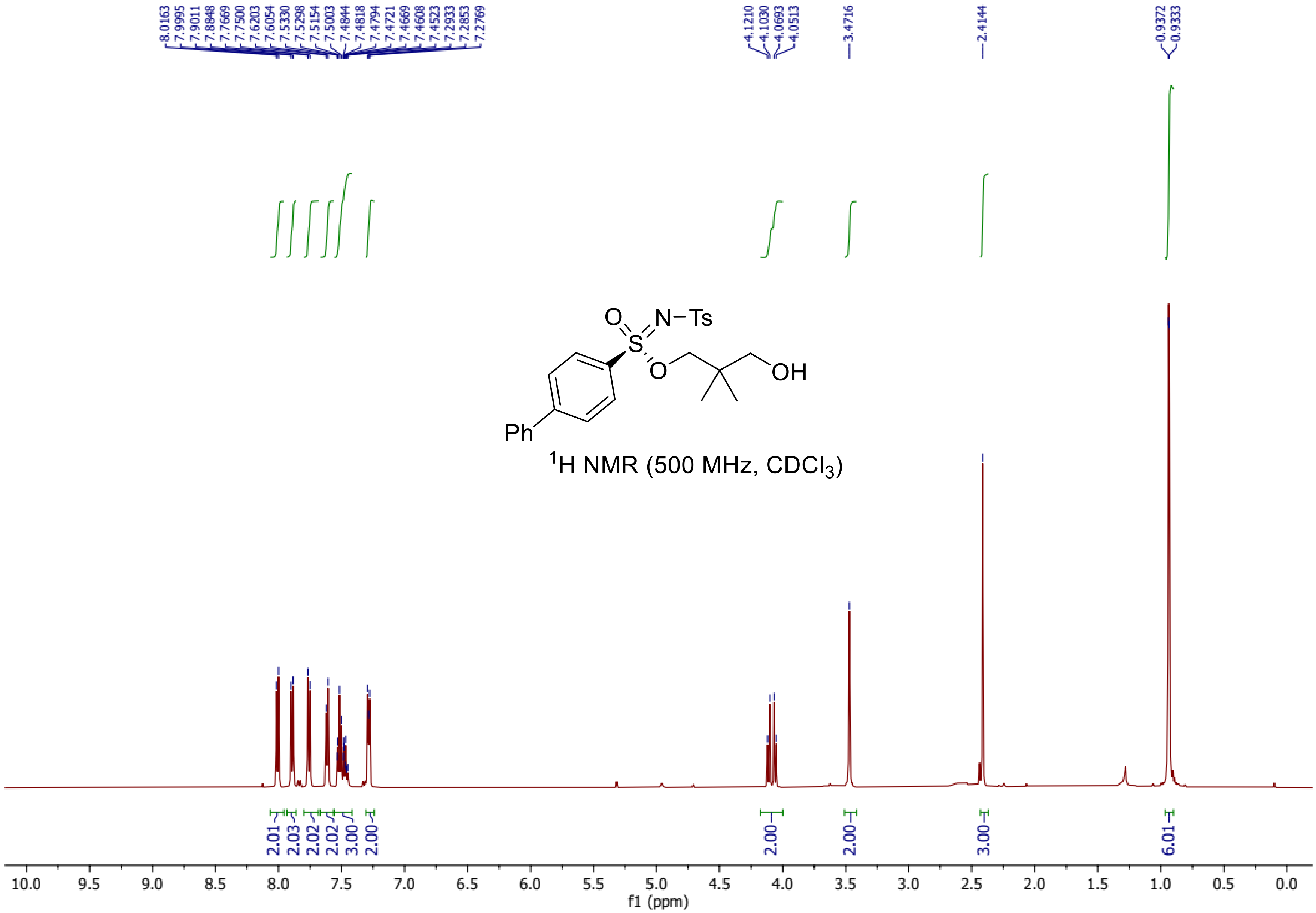

Go to table of contents

3-Hydroxy-2,2-dimethylpropyl (*R*)-*N*-tosyl-[1,1'-biphenyl]-4-sulfonimidate (3n)

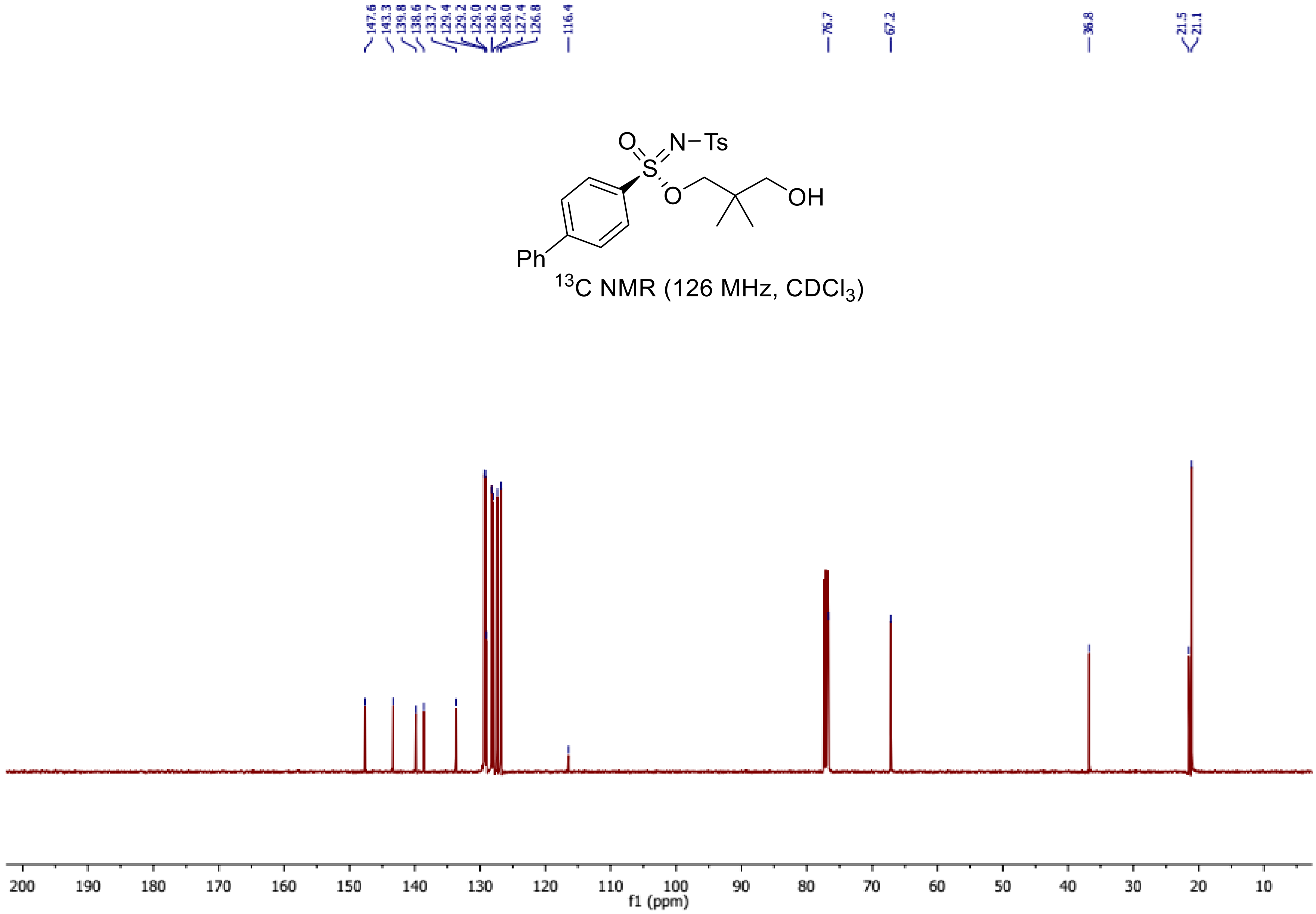

Go to table of contents

3-Hydroxy-2,2-dimethylpropyl *(R)*-4'-fluoro-*N*-tosyl-[1,1'-biphenyl]-4-sulfonimidate (3o)

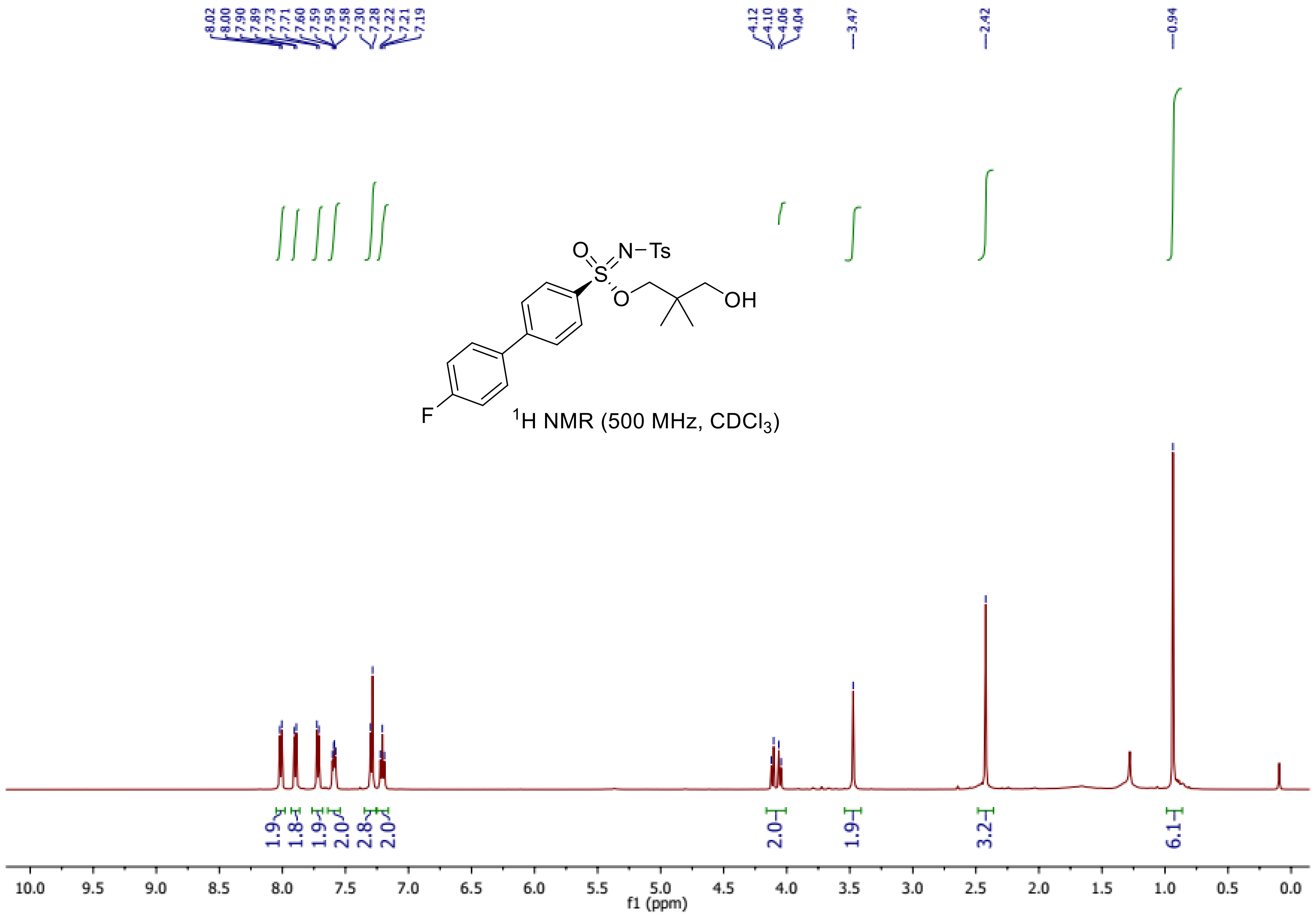

3-Hydroxy-2,2-dimethylpropyl *(R)*-4'-fluoro-*N*-tosyl-[1,1'-biphenyl]-4-sulfonimidate (3o)

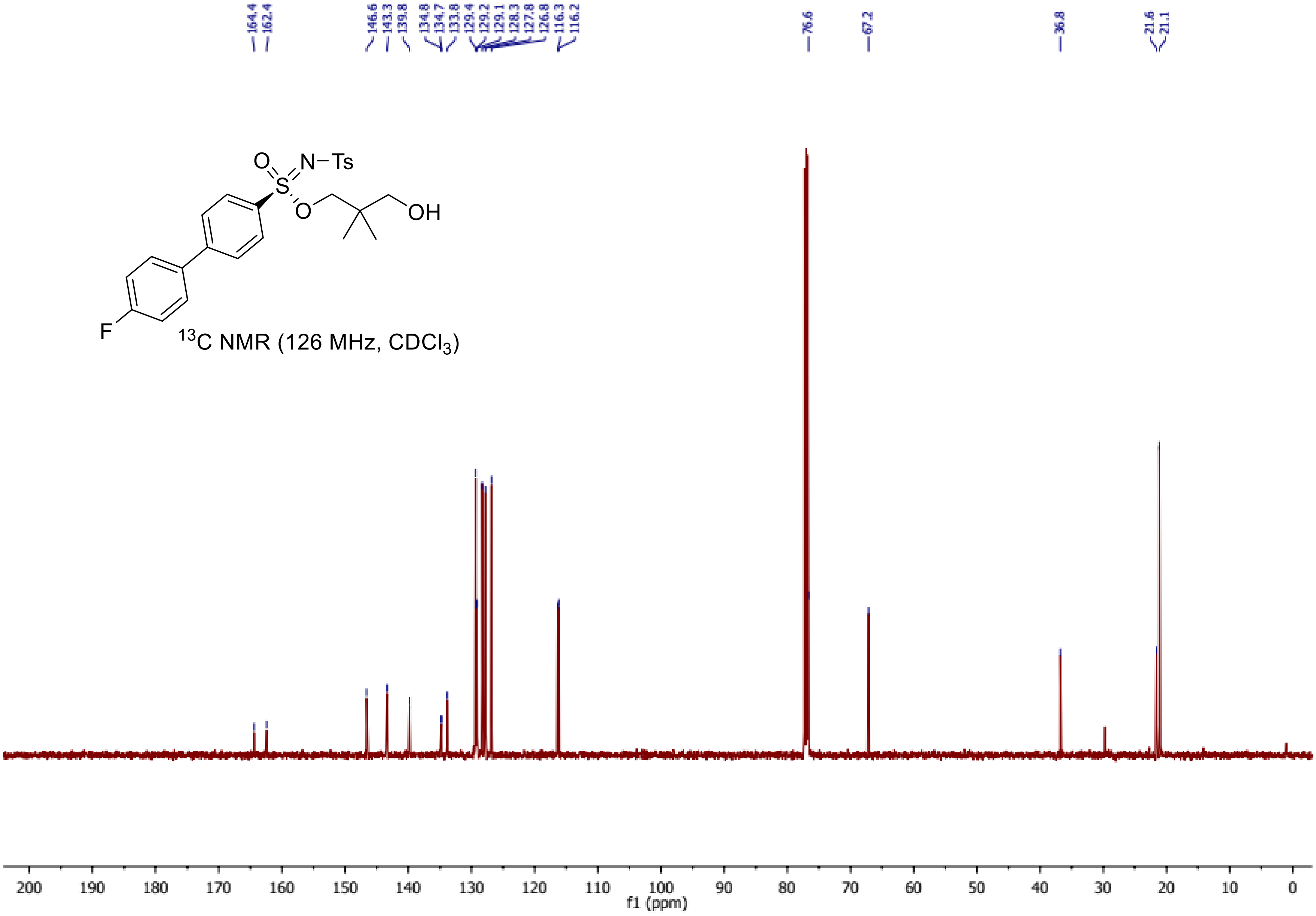

Go to table of contents

3-Hydroxy-2,2-dimethylpropyl *(R)*-2,5-difluoro-*N*-tosylbenzenesulfonimidate (3p)

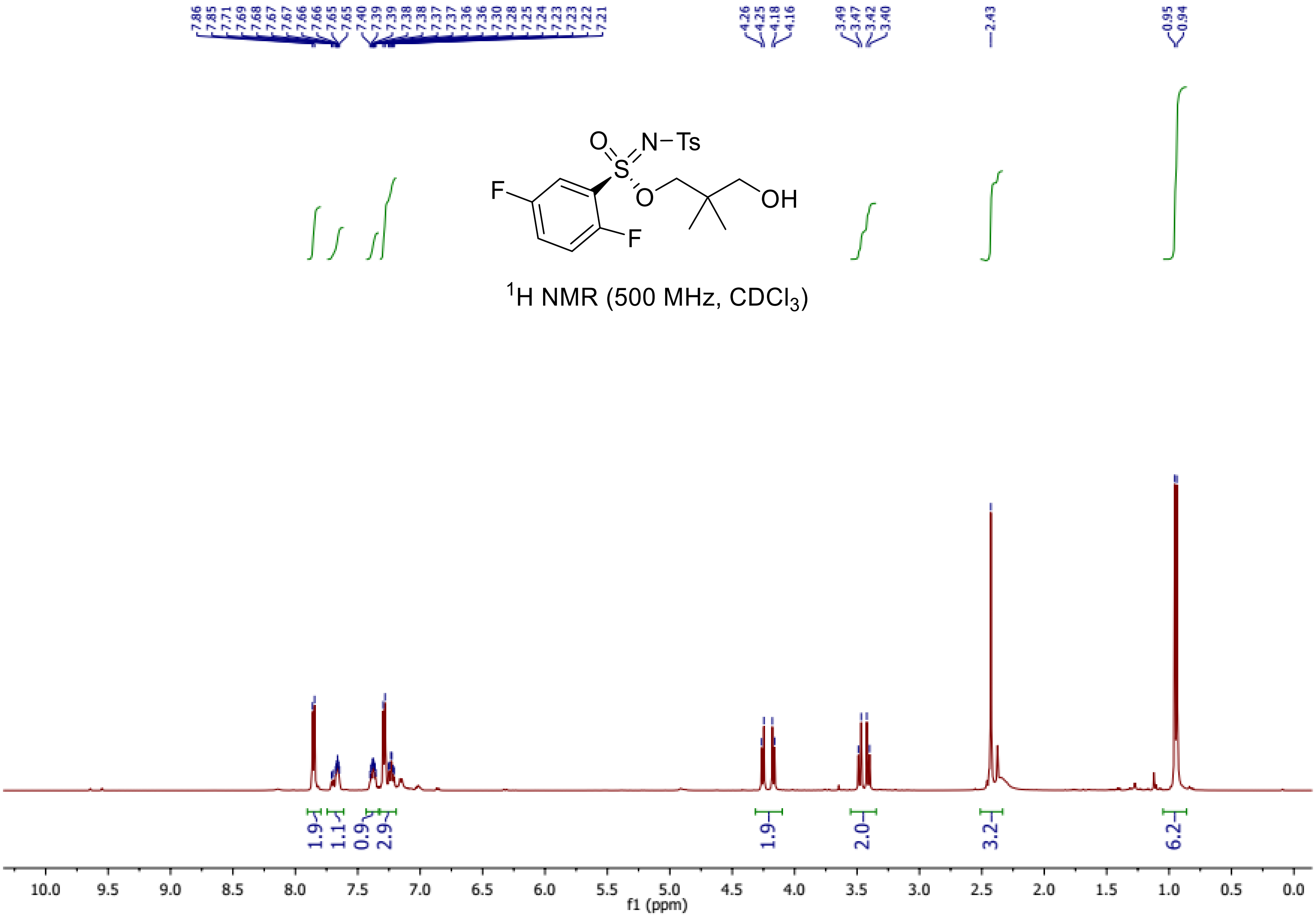

Go to table of contents

3-Hydroxy-2,2-dimethylpropyl *(R)*-2,5-difluoro-*N*-tosylbenzenesulfonimidate (3p)

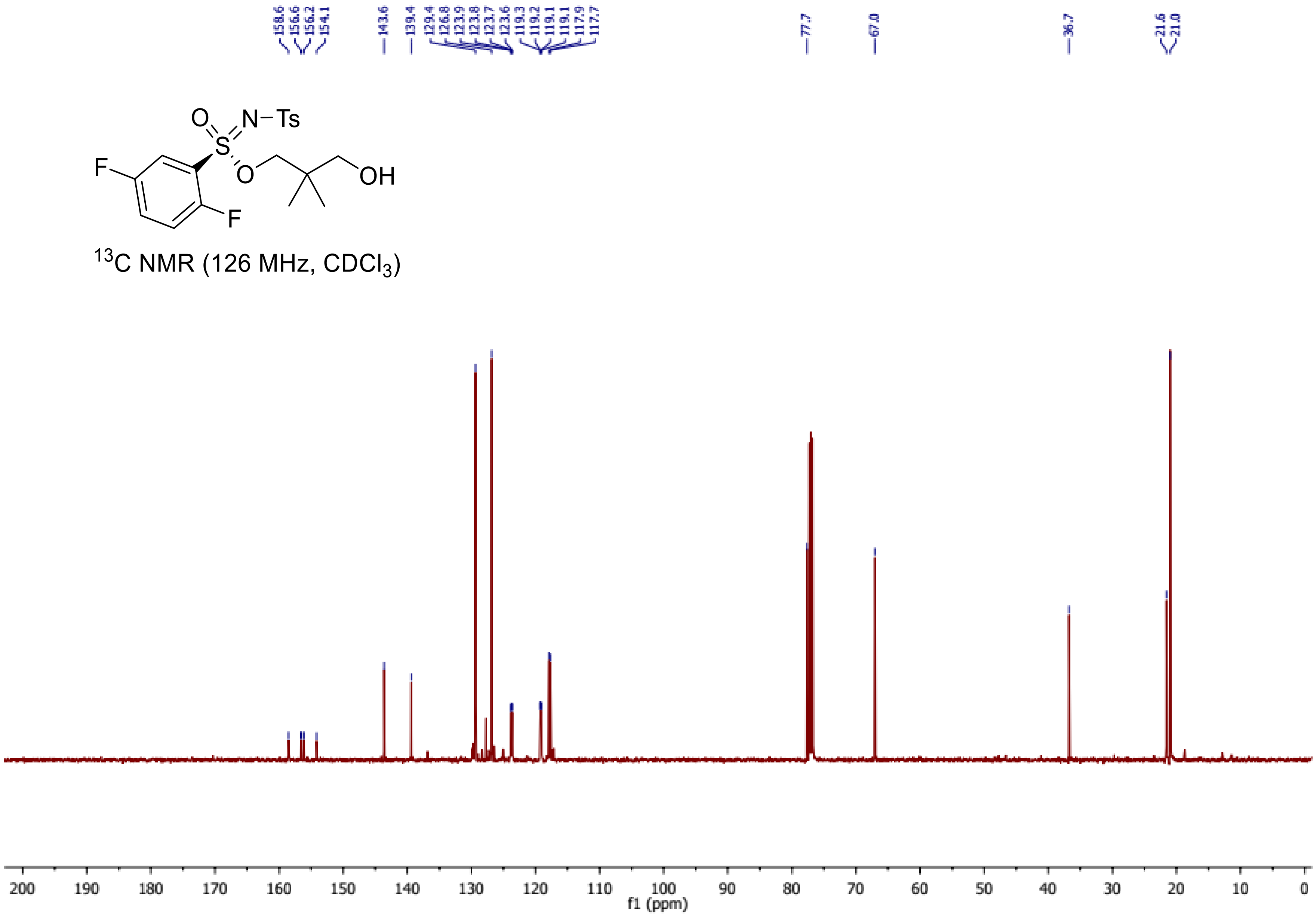

Go to table of contents

3-Hydroxy-2,2-dimethylpropyl *(R)*-3,5-dichloro-*N*-tosylbenzenesulfonimidate (3q)

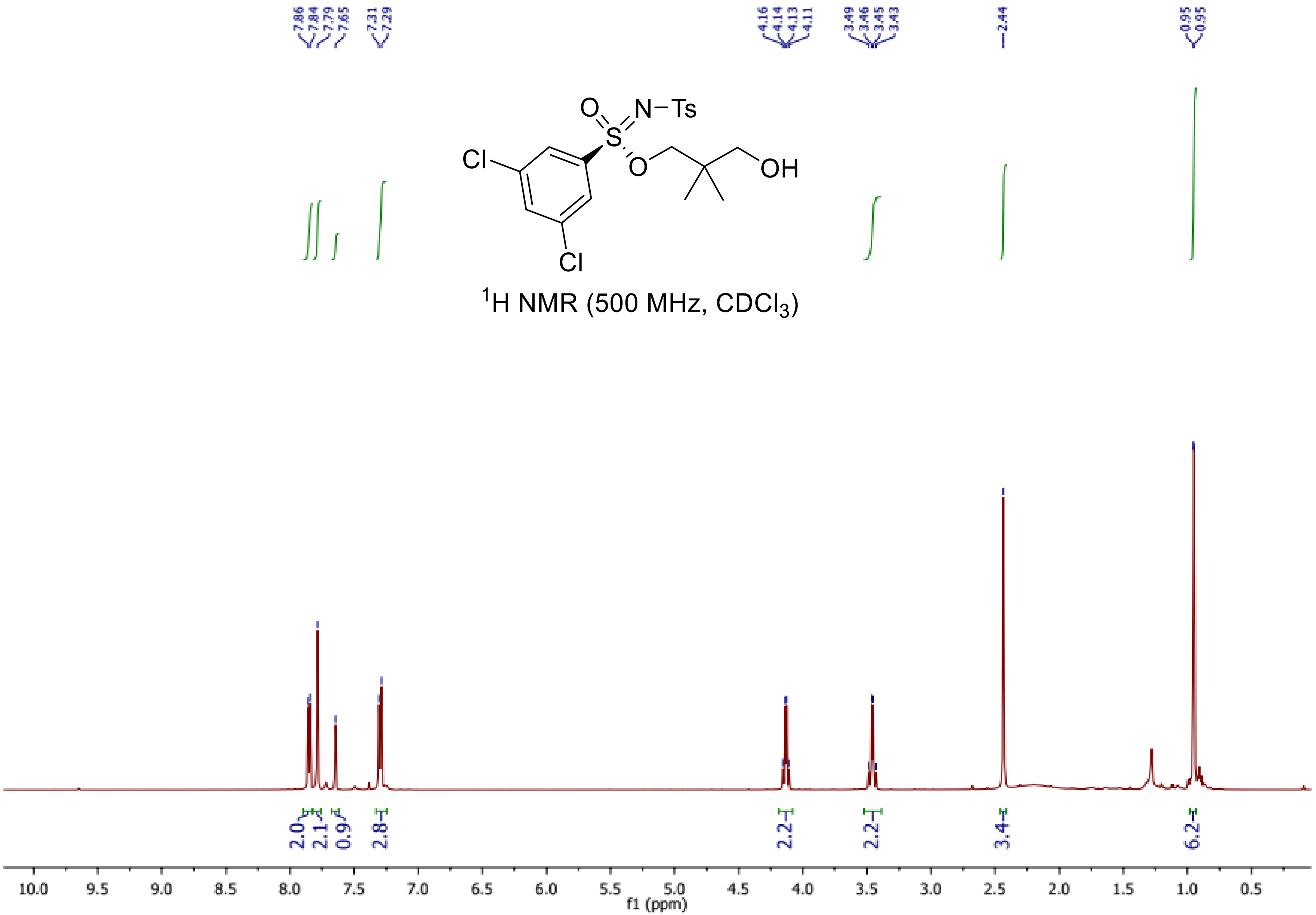

Go to table of contents

3-Hydroxy-2,2-dimethylpropyl *(R)*-3,5-dichloro-*N*-tosylbenzenesulfonimidate (3q)

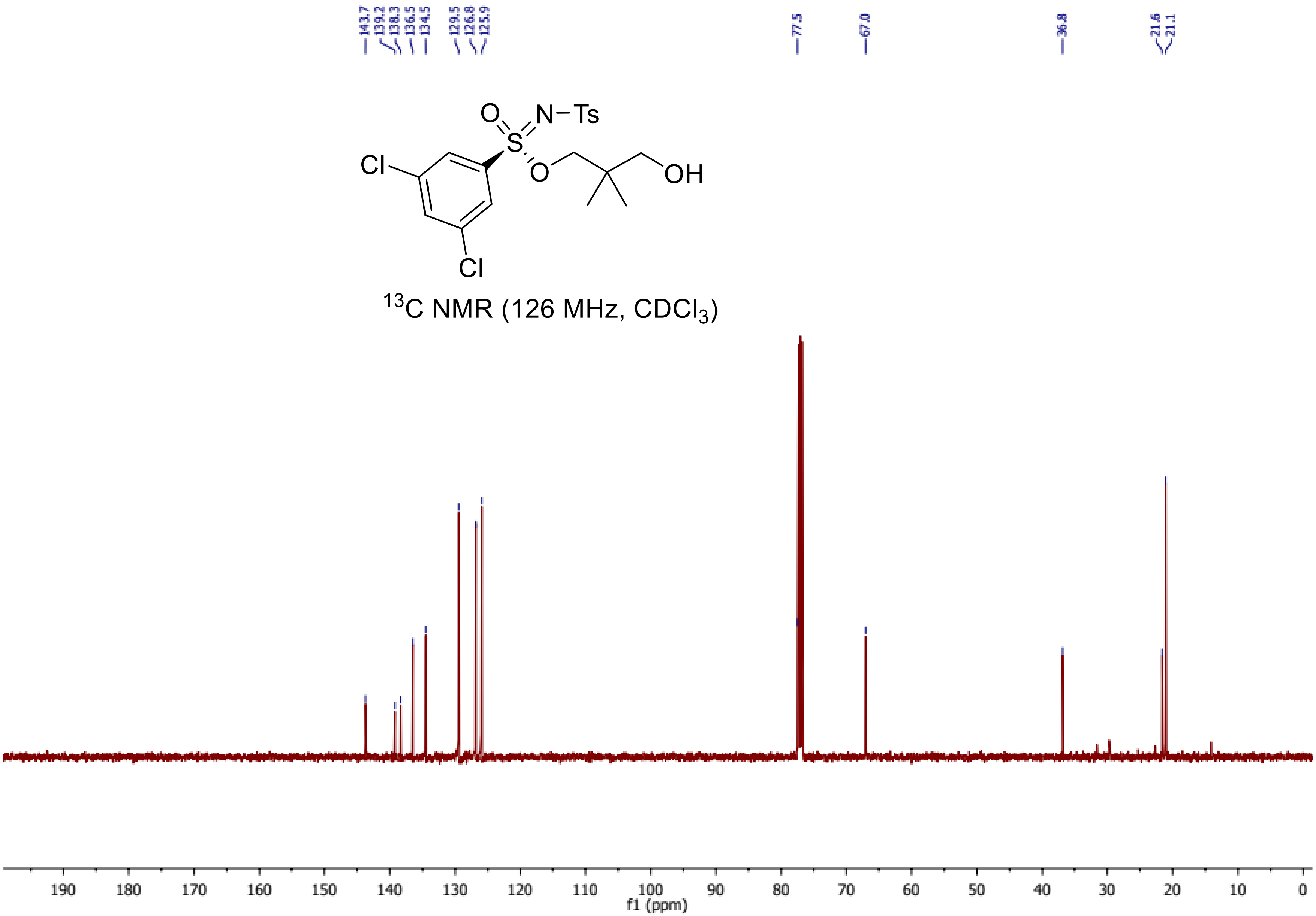

Go to table of contents

3-Hydroxy-2,2-dimethylpropyl *(R)*-3-chloro-4-fluoro-*N*-tosylbenzenesulfonimidate (3r)

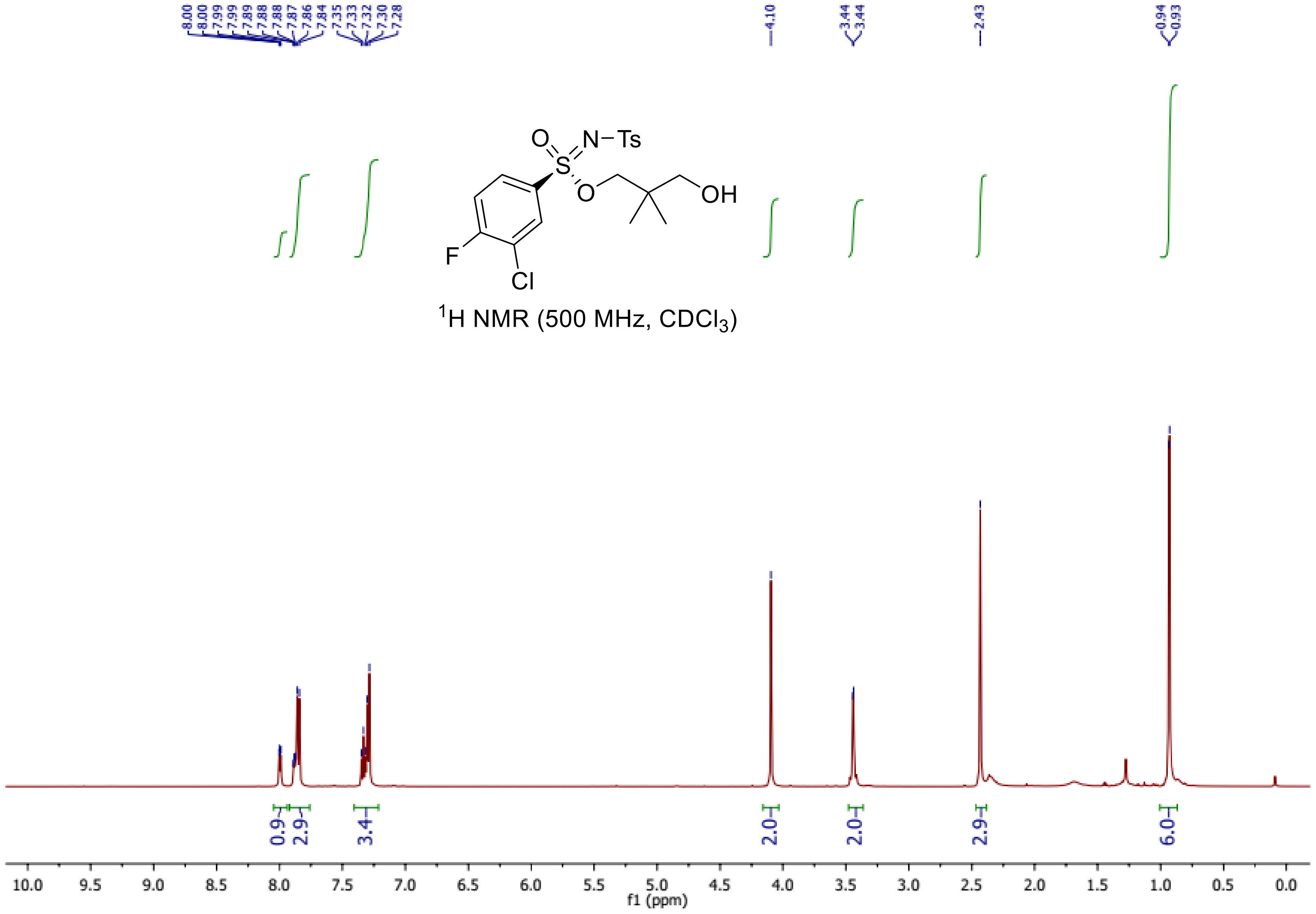

3-Hydroxy-2,2-dimethylpropyl *(R)*-3-chloro-4-fluoro-*N*-tosylbenzenesulfonimidate (3r)

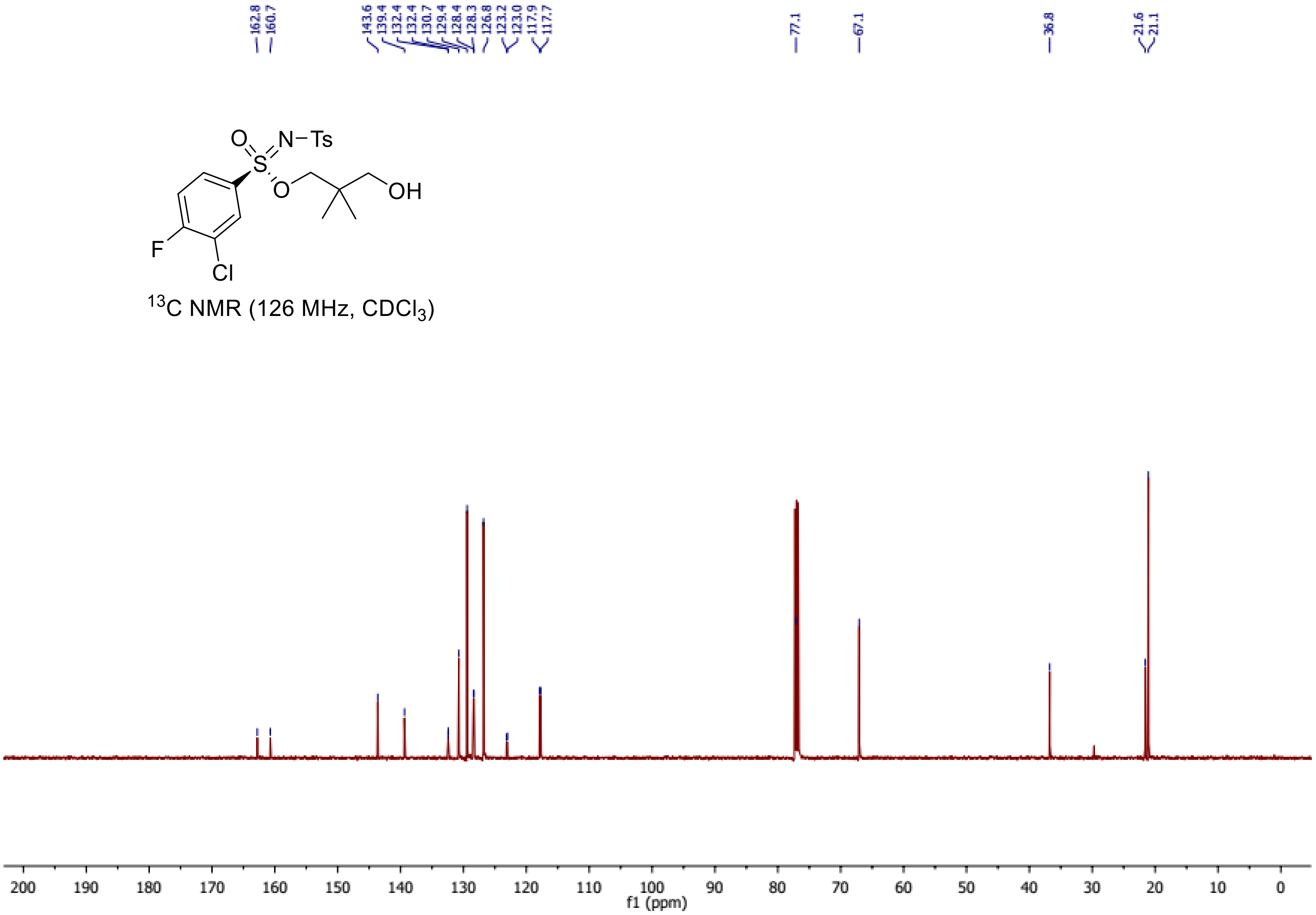

3-Hydroxy-2,2-dimethylpropyl *(R)*-4-fluoro-*N*-tosyl-3-(trifluoromethyl)benzenesulfonimidate (3s)

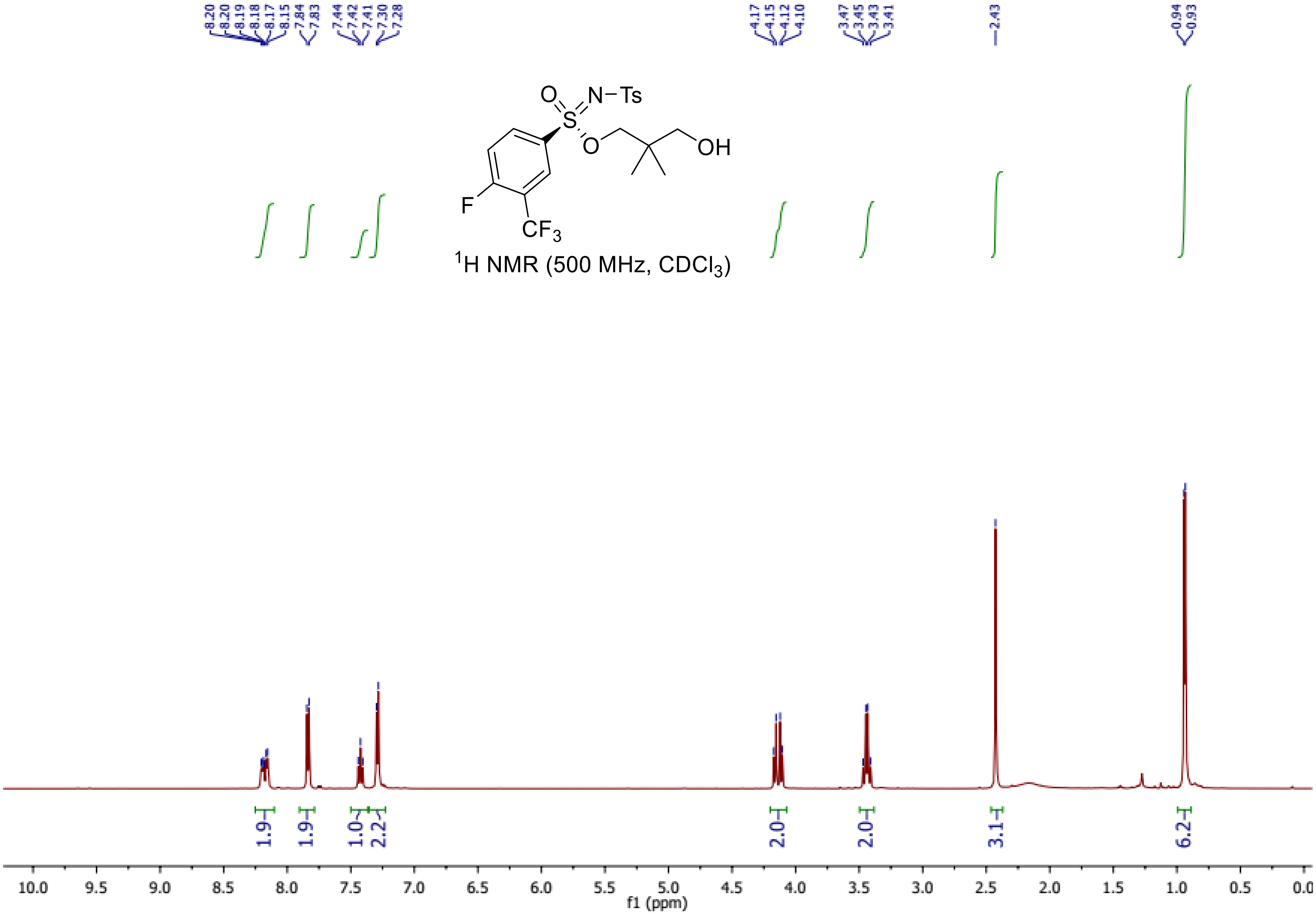

Go to table of contents

3-Hydroxy-2,2-dimethylpropyl *(R)*-4-fluoro-*N*-tosyl-3-(trifluoromethyl)benzenesulfonimidate (3s)

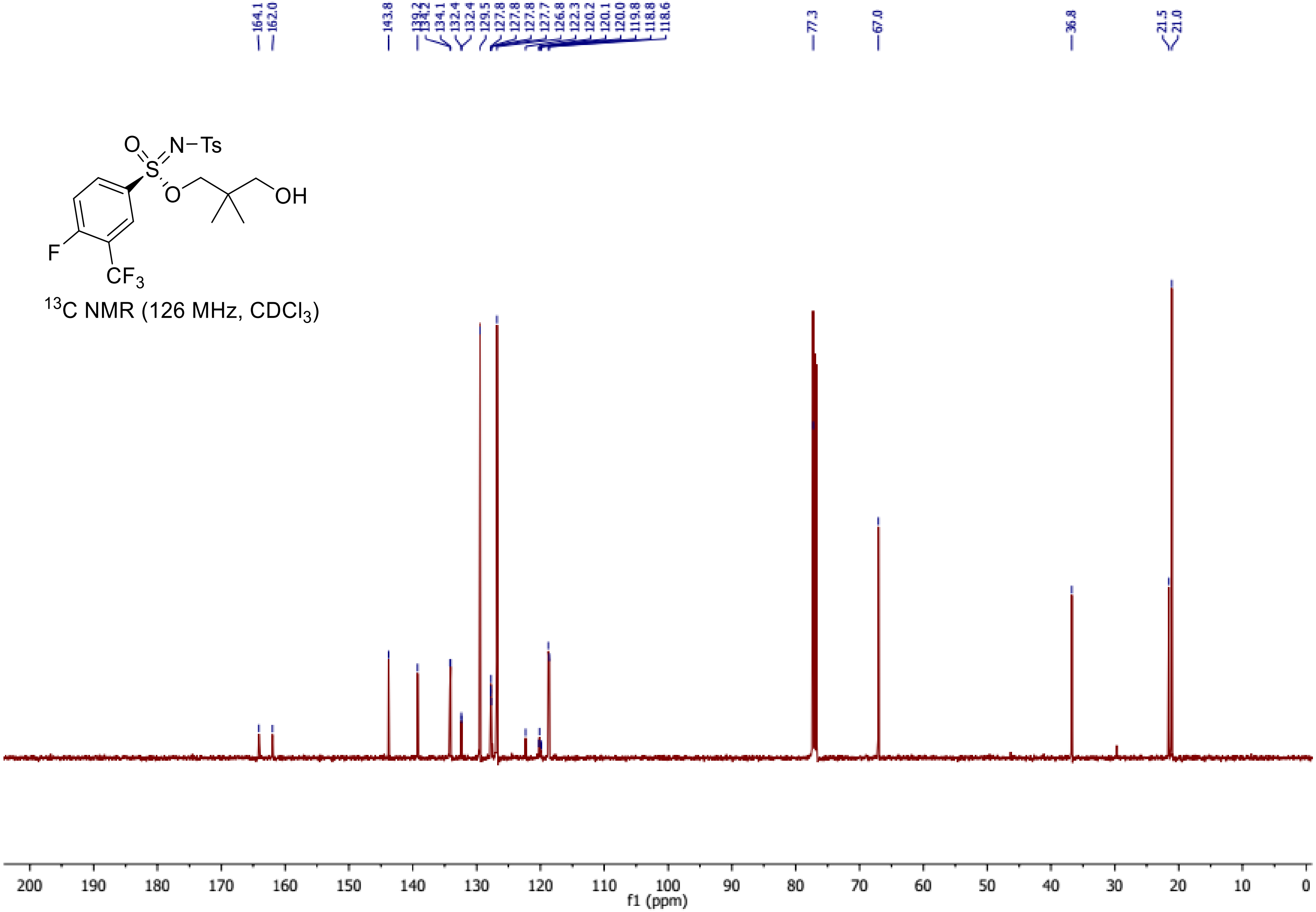

^{13}C NMR (126 MHz, $CDCl_3$)

Go to table of contents

3-Hydroxy-2,2-dimethylpropyl *(R)*-4-chloro-*N*-tosyl-3-(trifluoromethyl)benzenesulfonimidate (3t)

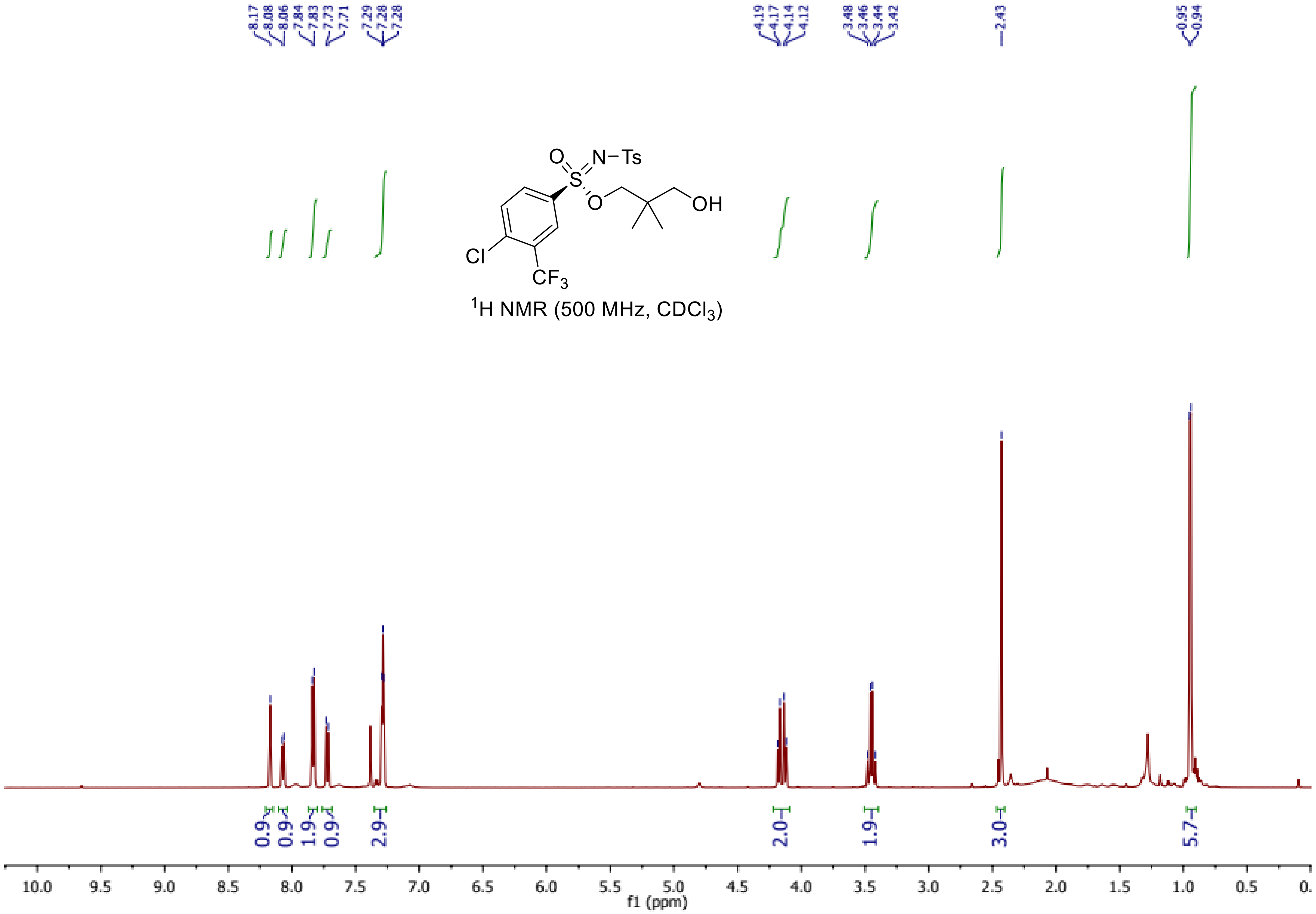

3-Hydroxy-2,2-dimethylpropyl (*R*)-4-chloro-*N*-tosyl-3-(trifluoromethyl)benzenesulfonimidate (3t)

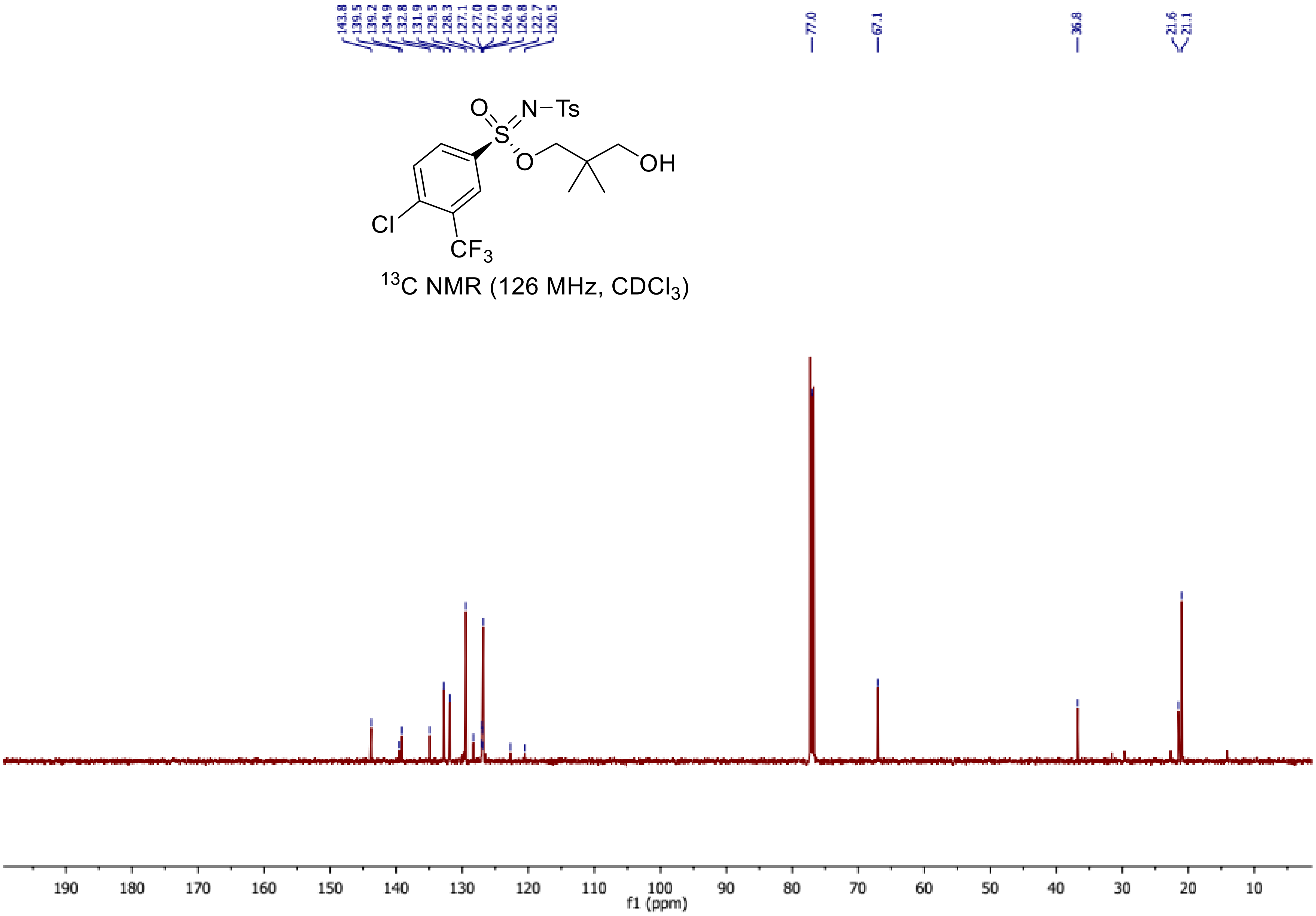

Go to table of contents

3-Hydroxy-2,2-dimethylpropyl (*R*)-4-bromo-3-methyl-*N*-tosylbenzenesulfonimidate (3u)

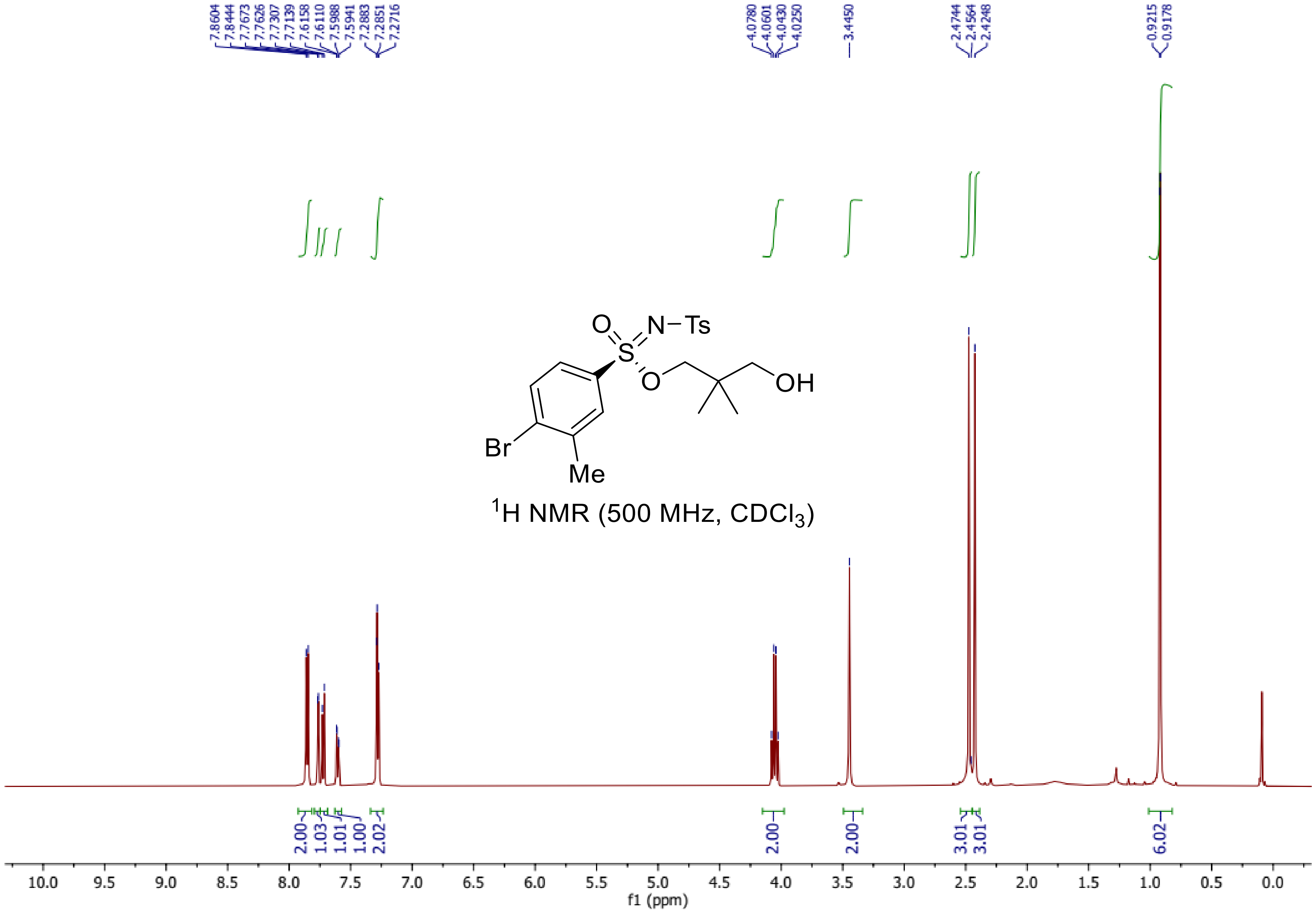

Go to table of contents

3-Hydroxy-2,2-dimethylpropyl (*R*)-4-bromo-3-methyl-*N*-tosylbenzenesulfonimidate (3u)

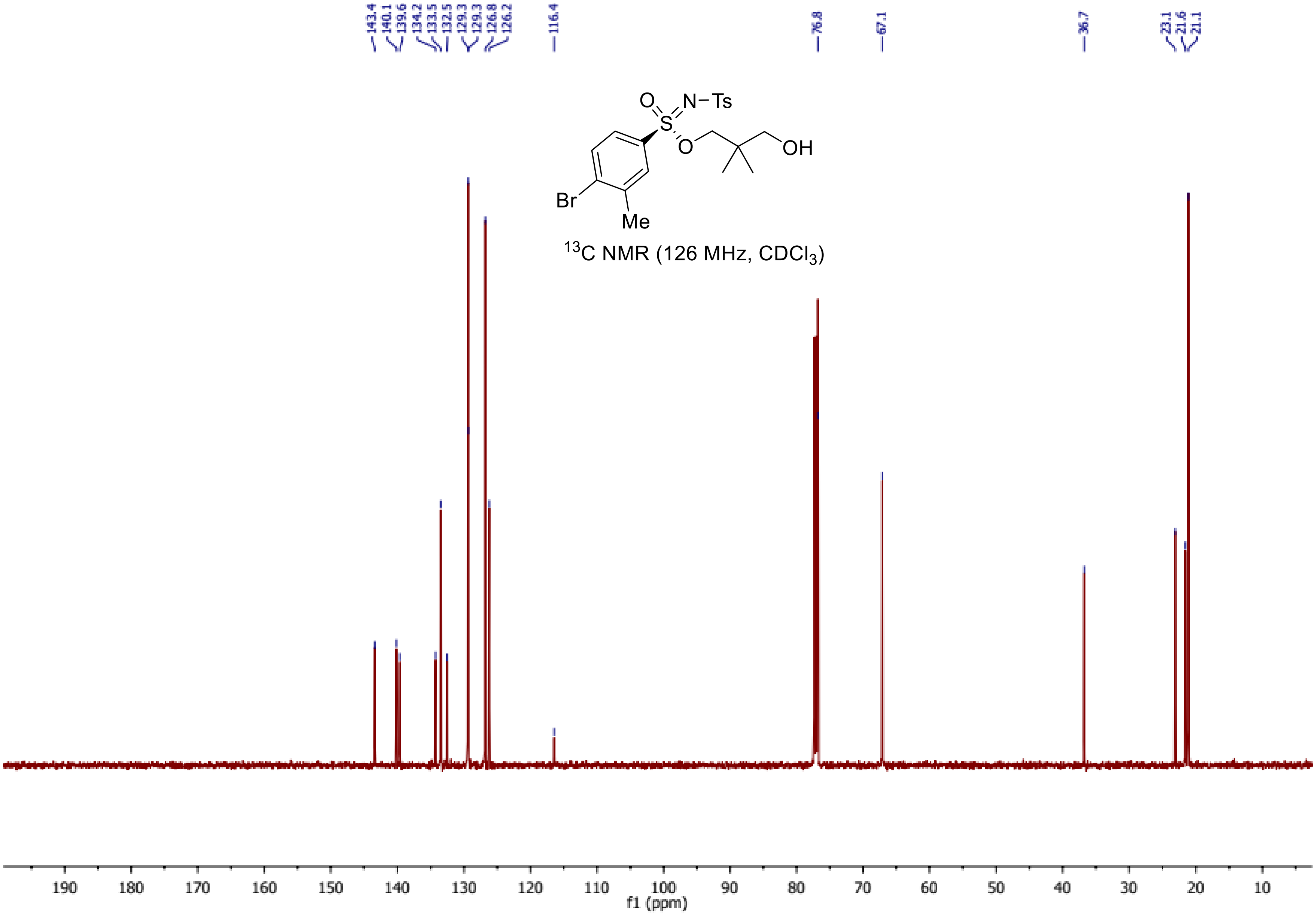

Go to table of contents

3-Hydroxy-2,2-dimethylpropyl (*R*)-*N*-tosylnaphthalene-2-sulfonimidate (3v)

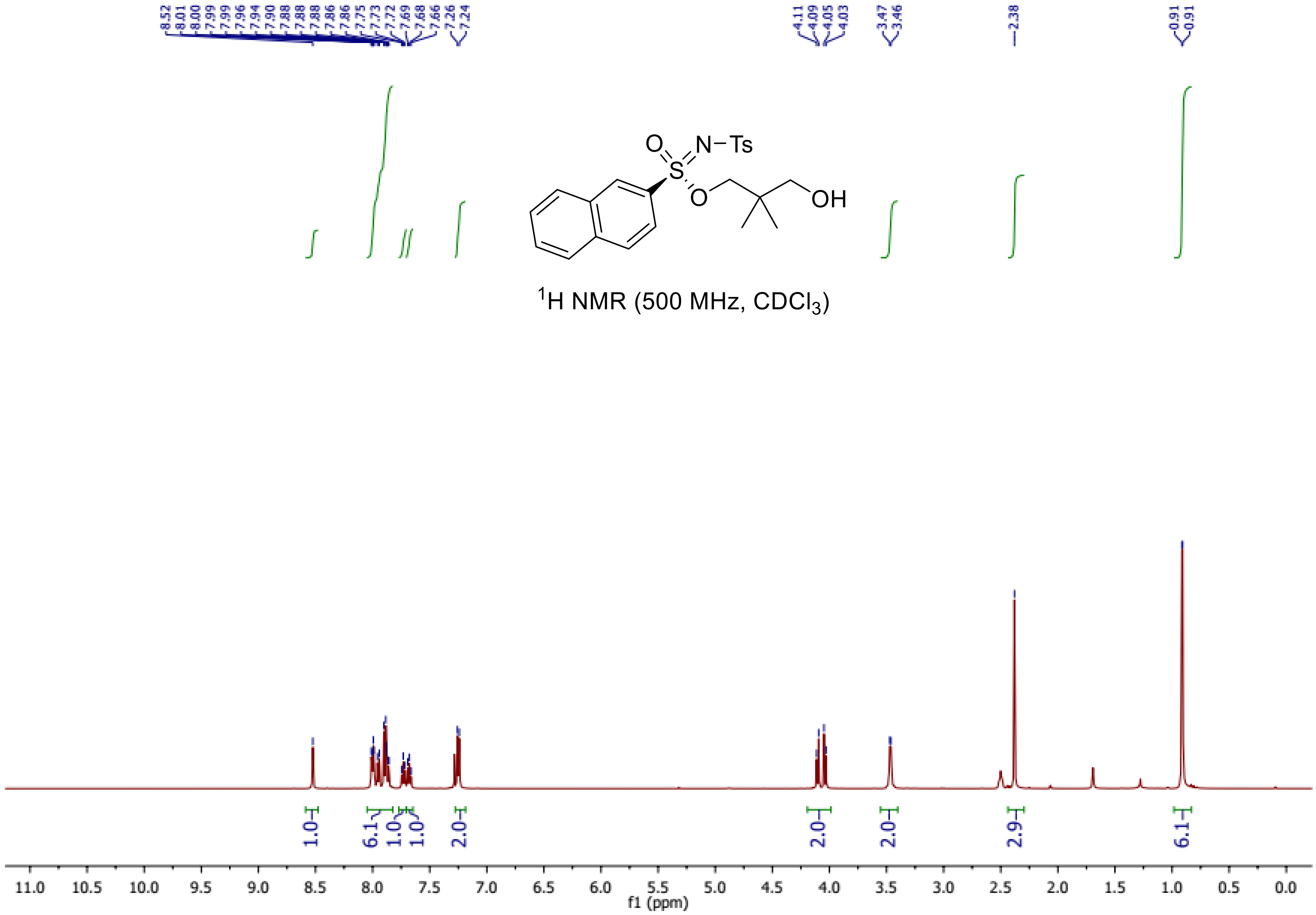

Go to table of contents

3-Hydroxy-2,2-dimethylpropyl (*R*)-*N*-tosylnaphthalene-2-sulfonimidate (3v)

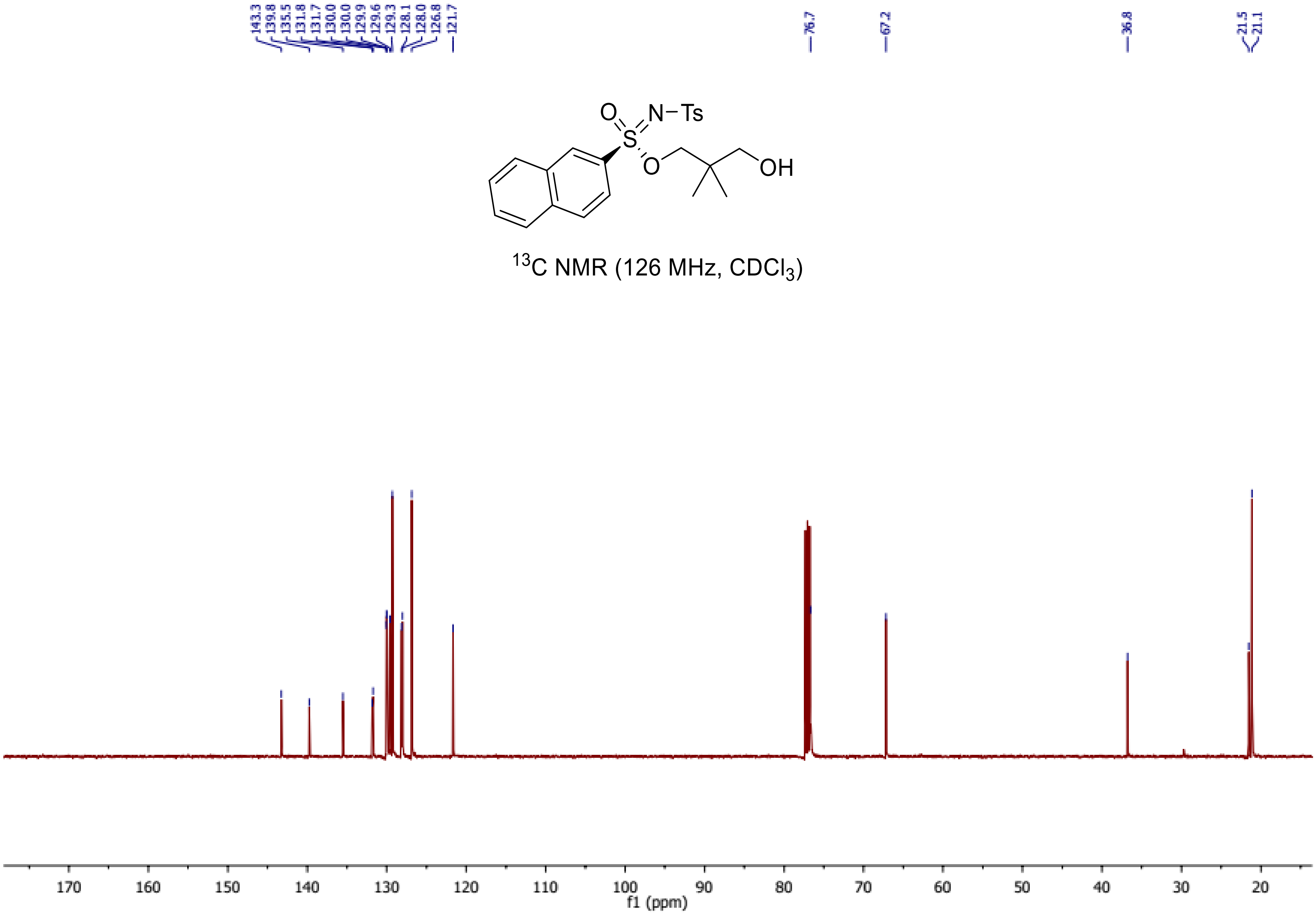

3-Hydroxy-2,2-dimethylpropyl (*R*)-*N*-((4-fluorophenyl)sulfonyl)benzenesulfonimidate (3w)

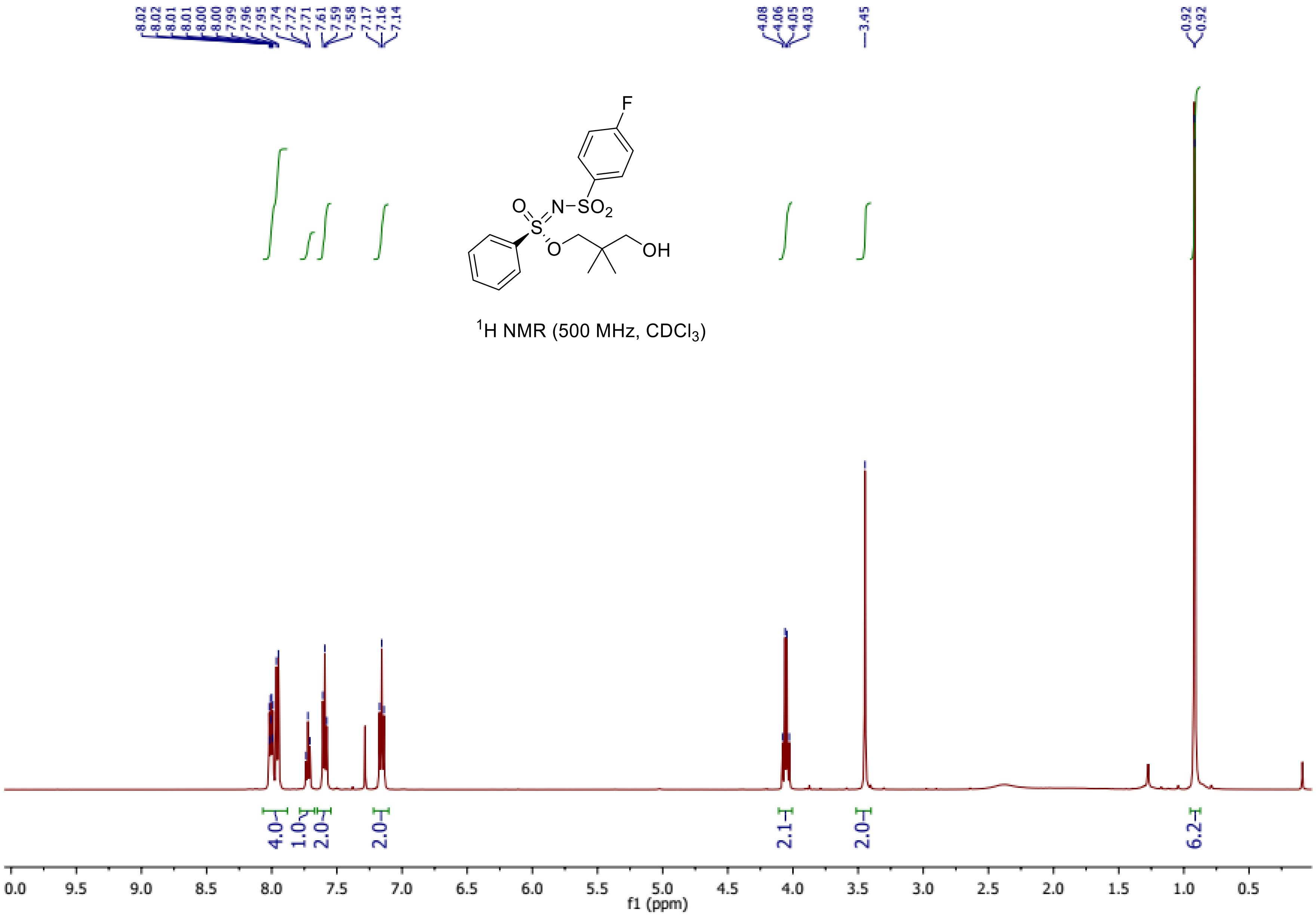

Go to table of contents

3-Hydroxy-2,2-dimethylpropyl (*R*)-*N*-((4-fluorophenyl)sulfonyl)benzenesulfonimidate (3w)

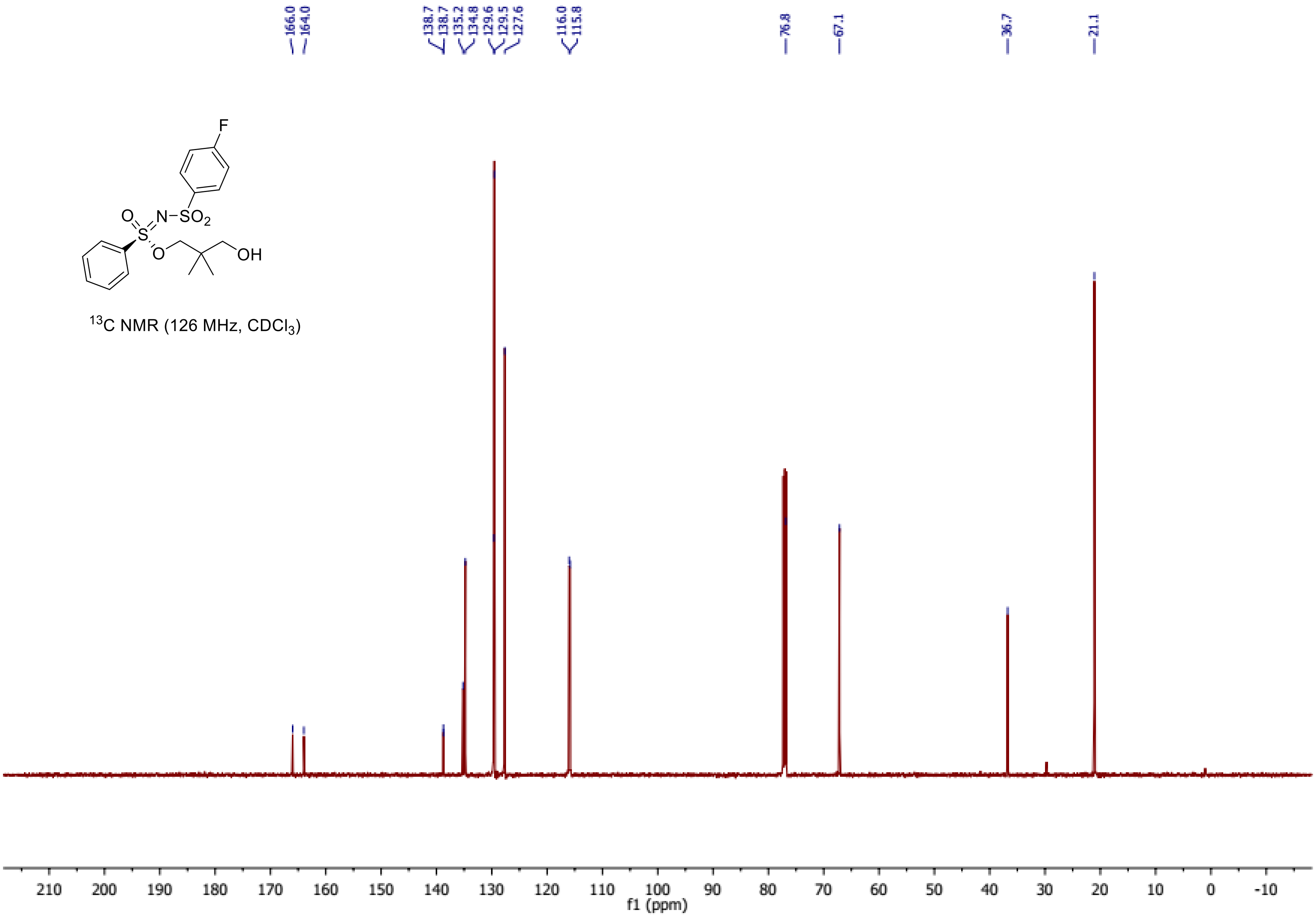

Go to table of contents

3-Hydroxy-2,2-dimethylpropyl (*R*)-*N*-((3-fluorophenyl)sulfonyl)benzenesulfonimidate (3x)

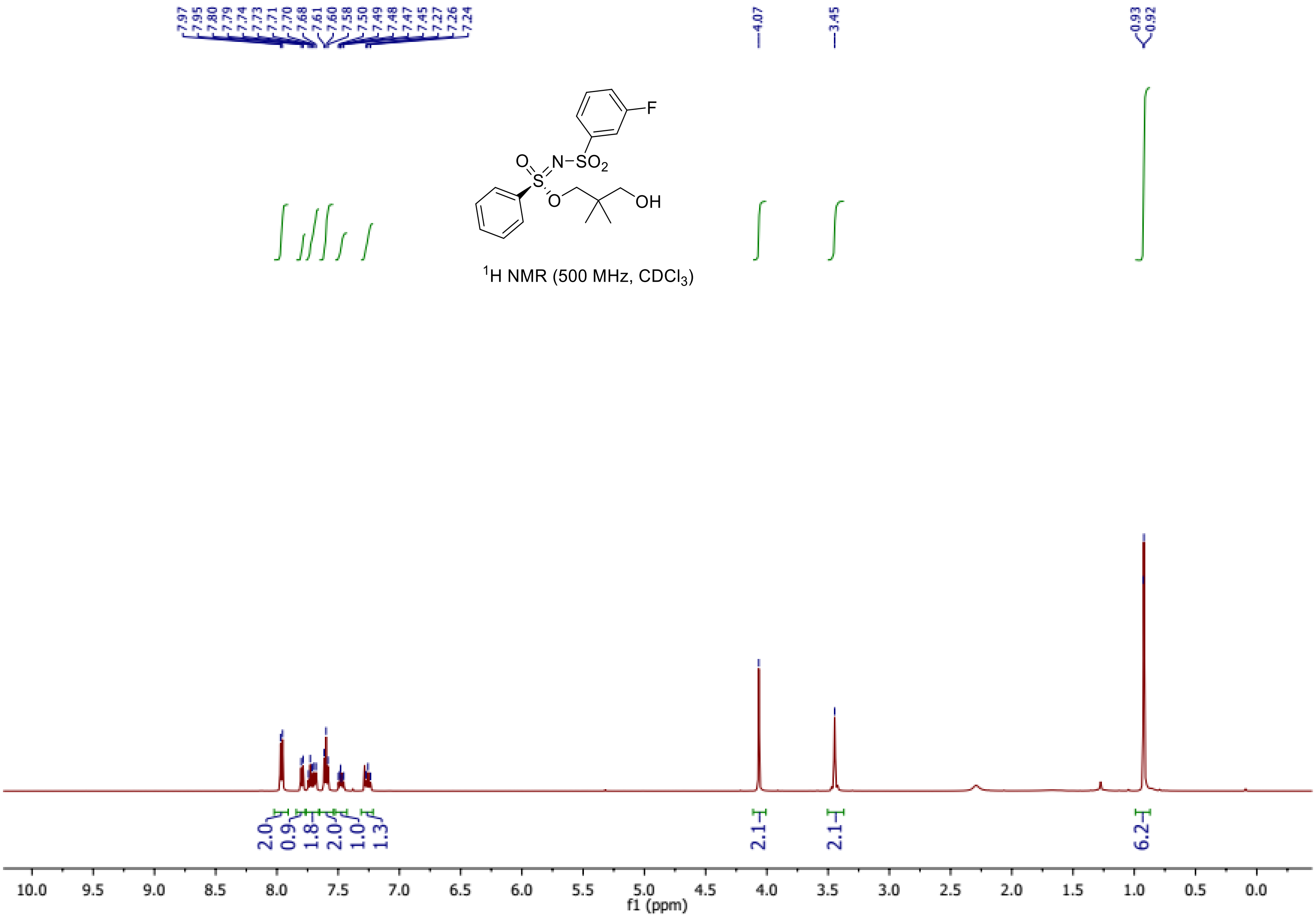

Go to table of contents

3-Hydroxy-2,2-dimethylpropyl (*R*)-*N*-((3-fluorophenyl)sulfonyl)benzenesulfonimidate (3x)

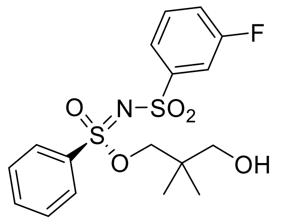

^{13}C NMR (126 MHz, $CDCl_3$)

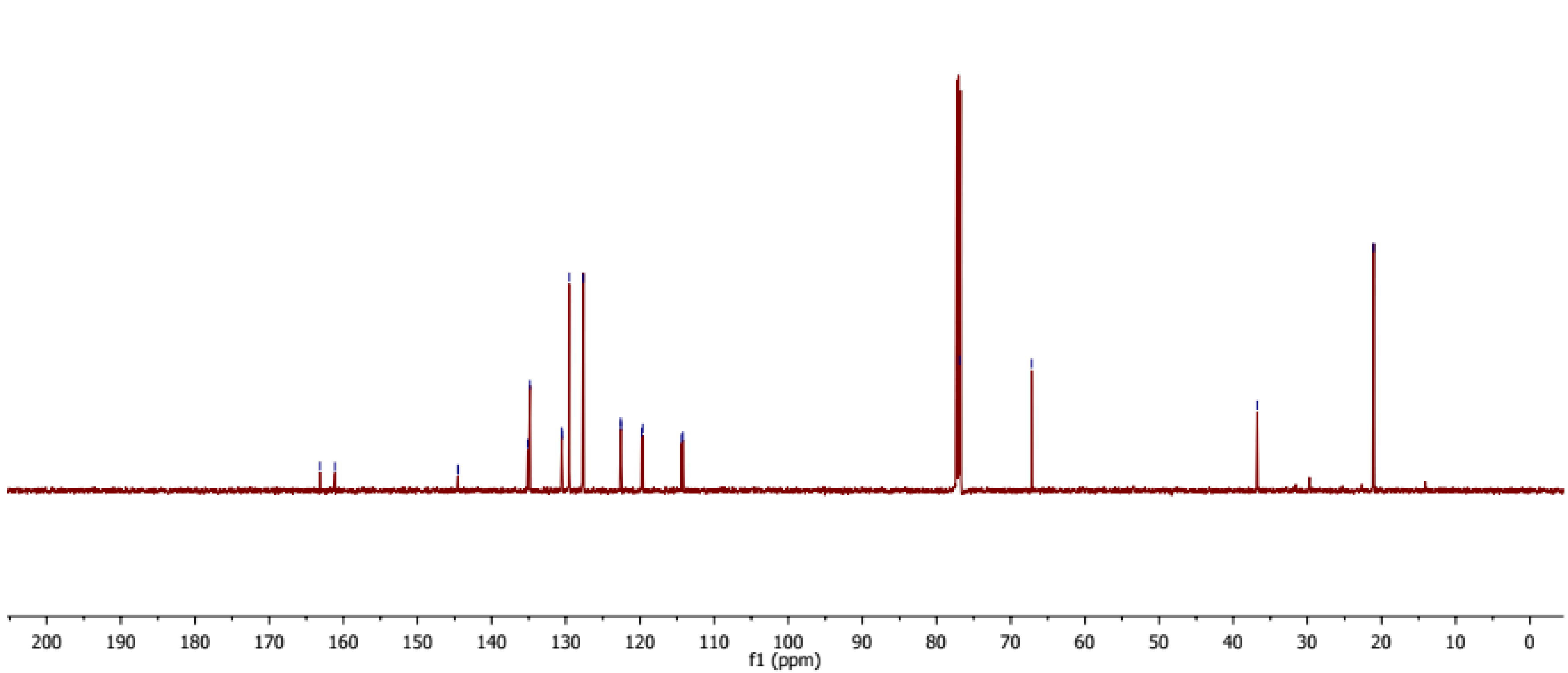

Go to table of contents

3-Hydroxy-2,2-dimethylpropyl (*R*)-*N*-((3,4-difluorophenyl)sulfonyl)benzenesulfonimidate (3y)

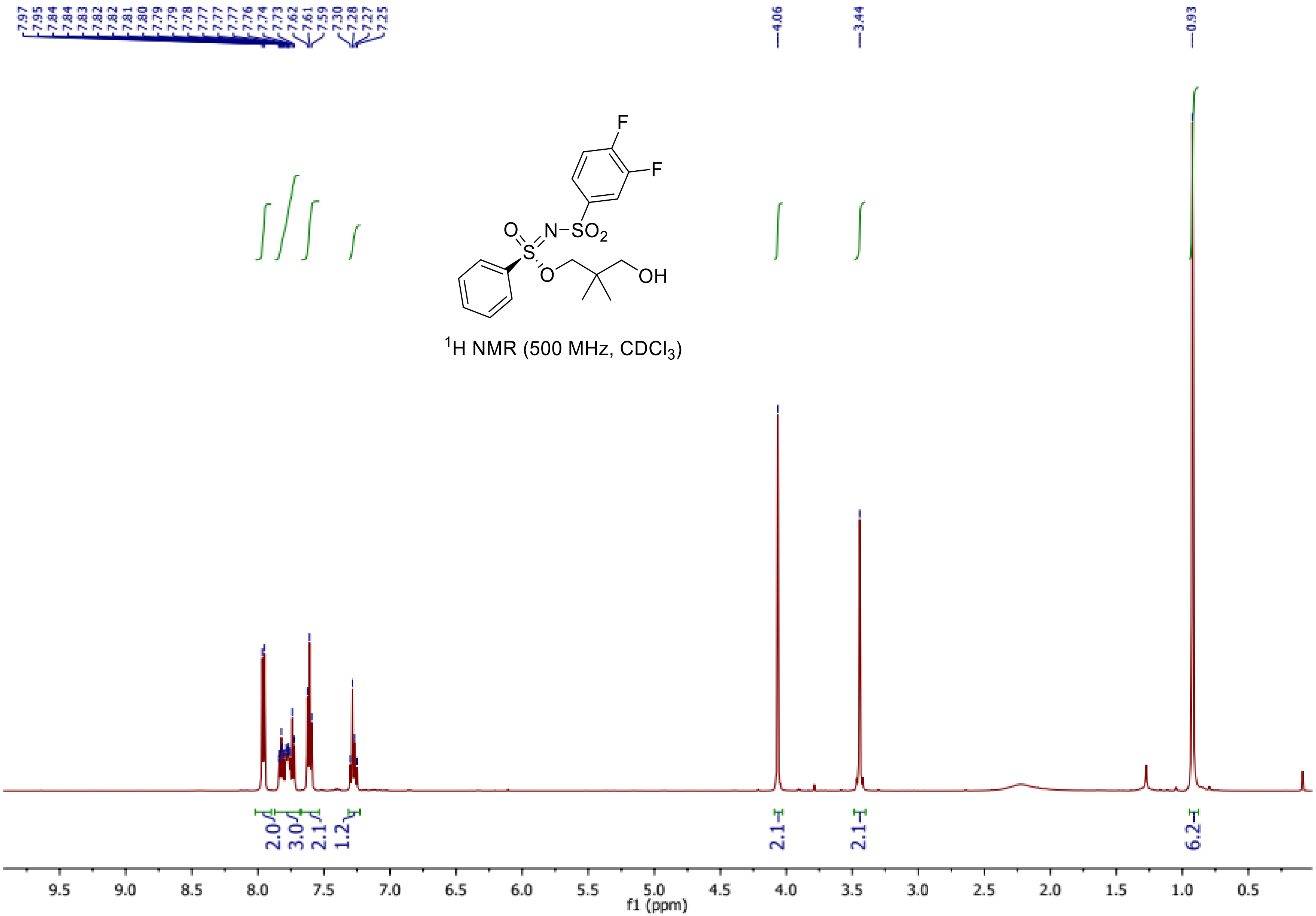

Go to table of contents

3-Hydroxy-2,2-dimethylpropyl (*R*)-*N*-((3,4-difluorophenyl)sulfonyl)benzenesulfonimidate (3y)

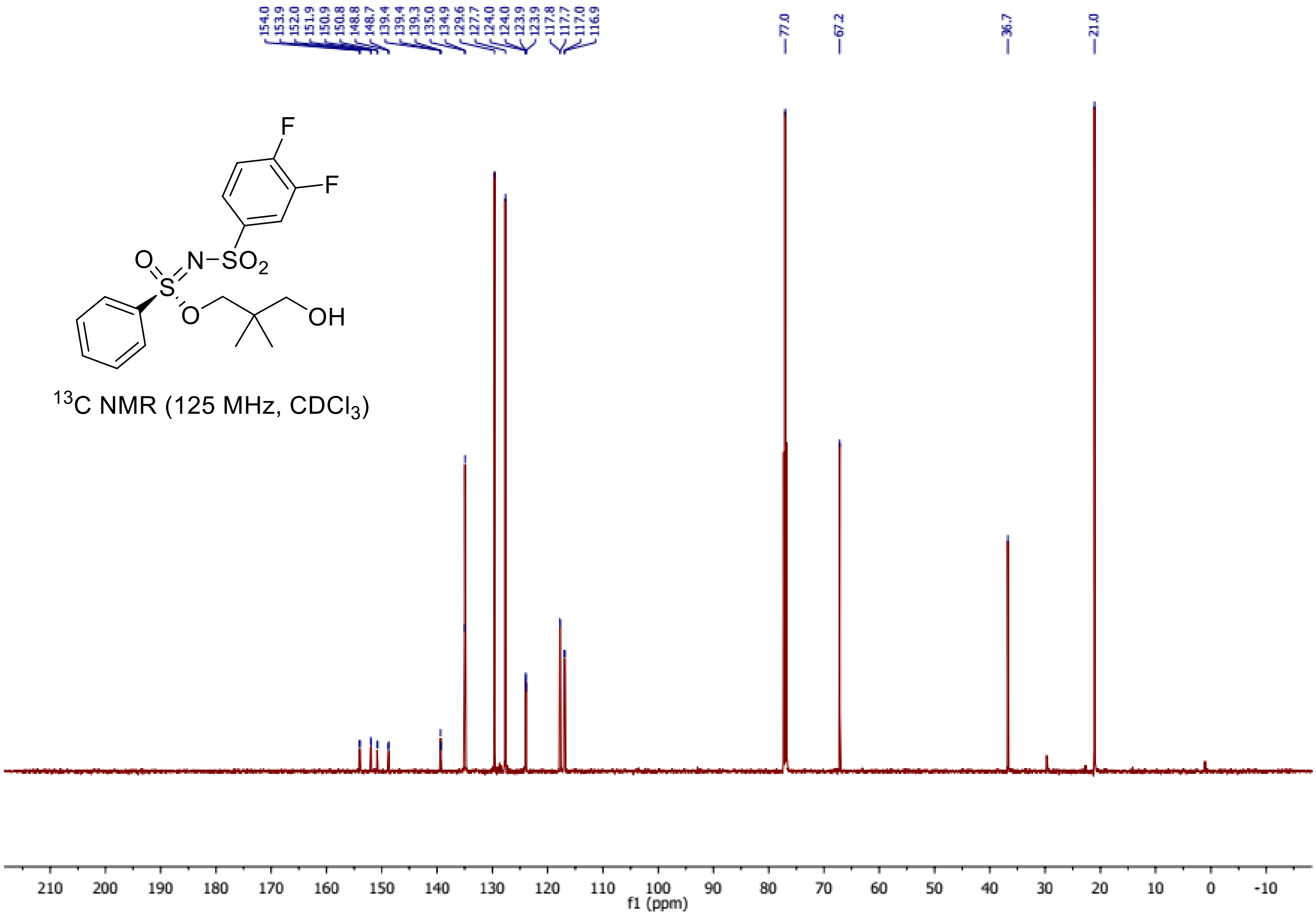

Go to table of contents

3-Hydroxy-2,2-dimethylpropyl (*R*)-*N*-((4-bromophenyl)sulfonyl)benzenesulfonimidate (3z)

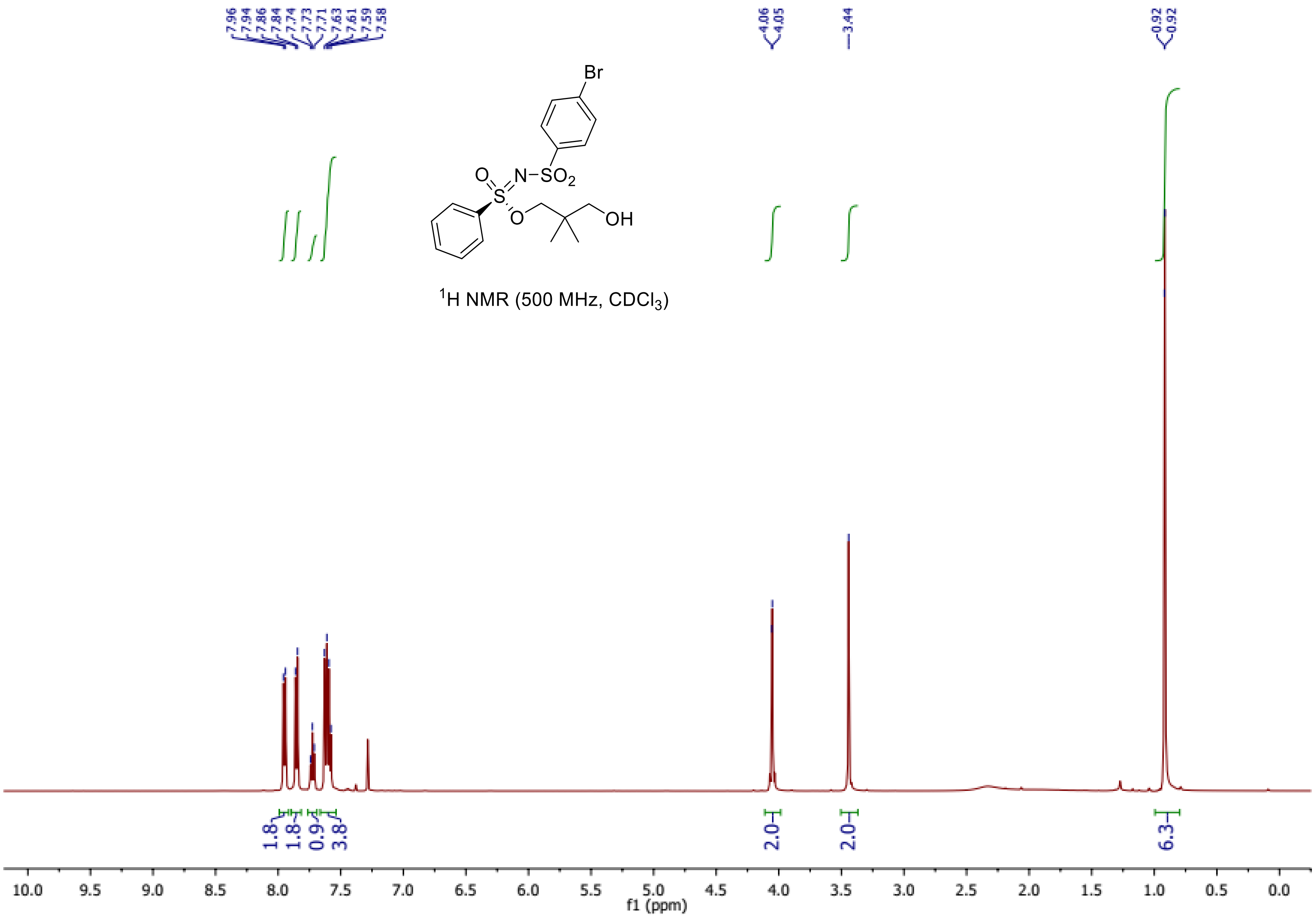

Go to table of contents

3-Hydroxy-2,2-dimethylpropyl (*R*)-*N*-((4-bromophenyl)sulfonyl)benzenesulfonimidate (3z)

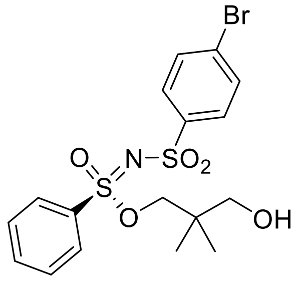

^{13}C NMR (126 MHz, $CDCl_3$)

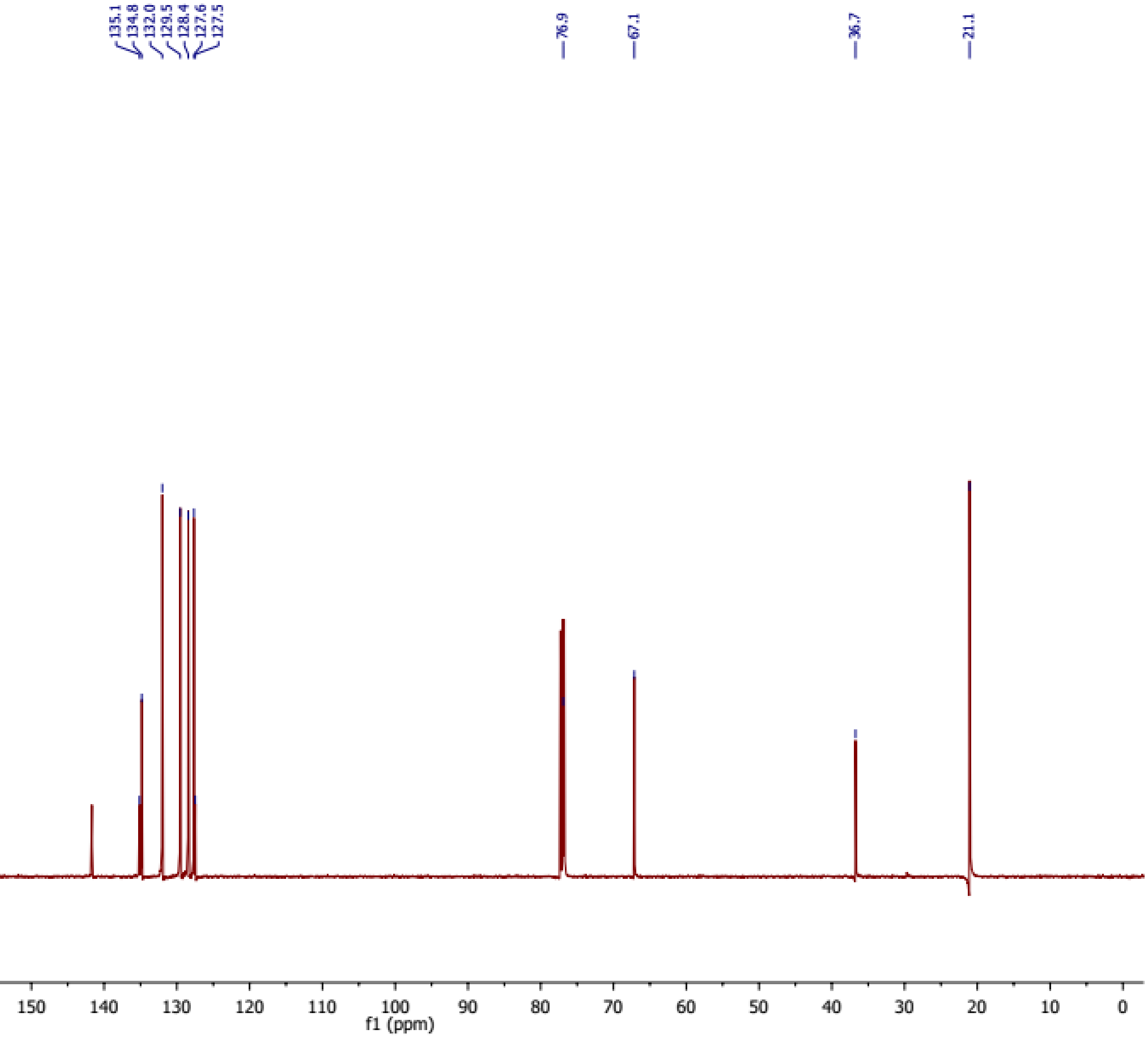

Go to table of contents

3-Hydroxy-2,2-dimethylpropyl (*R*)-*N*-((3-(trifluoromethyl)phenyl)sulfonyl)benzenesulfonimidate (3aa)

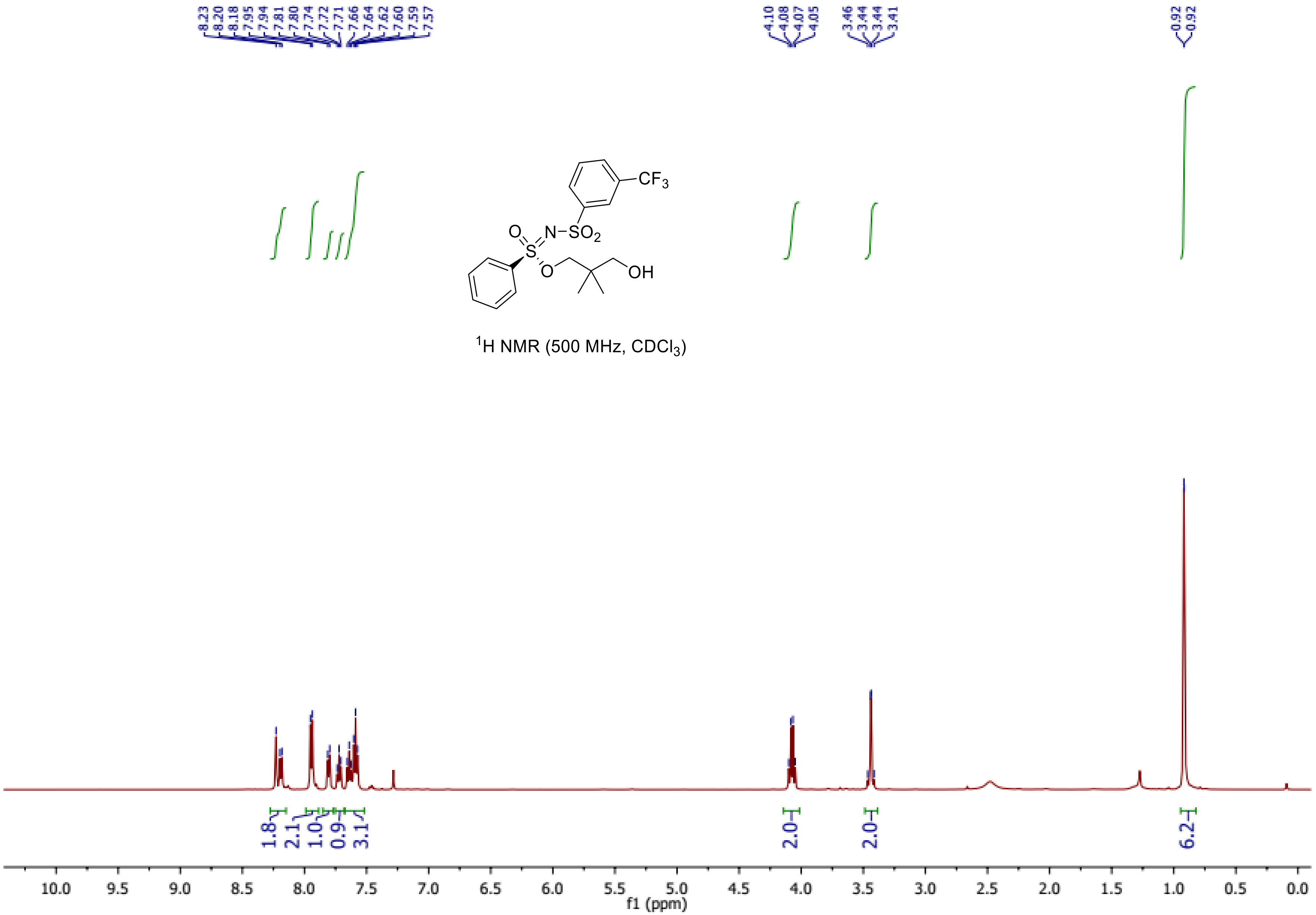

Go to table of contents

3-Hydroxy-2,2-dimethylpropyl (*R*)-*N*-((3-(trifluoromethyl)phenyl)sulfonyl)benzenesulfonimidate (3aa)

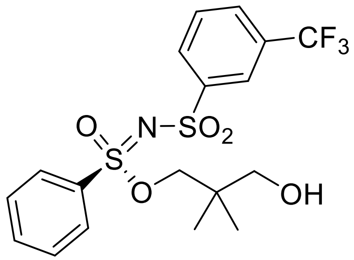

^{13}C NMR (126 MHz, $CDCl_3$)

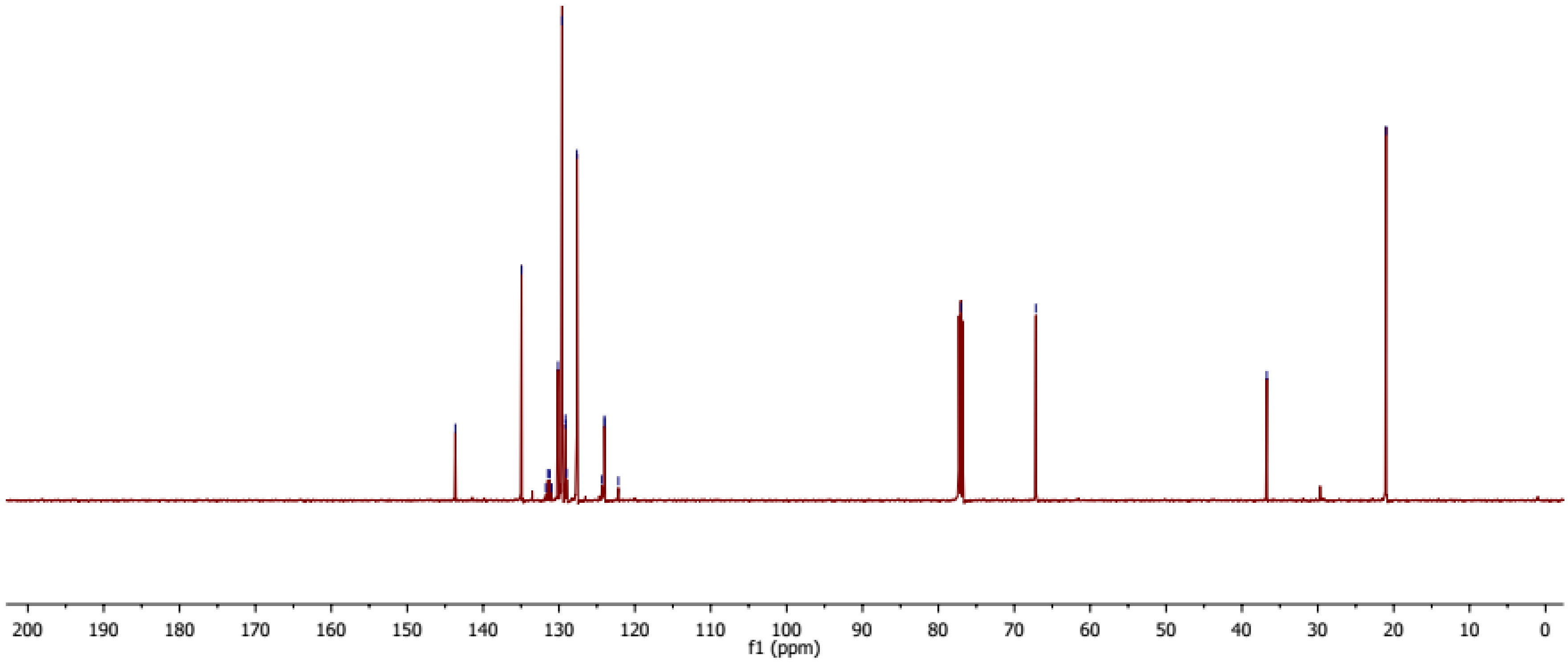

Go to table of contents

3-Hydroxy-2,2-dimethylpropyl (*R*)-*N*-((3-chloro-4-methoxyphenyl)sulfonyl)benzenesulfonimidate (3ab)

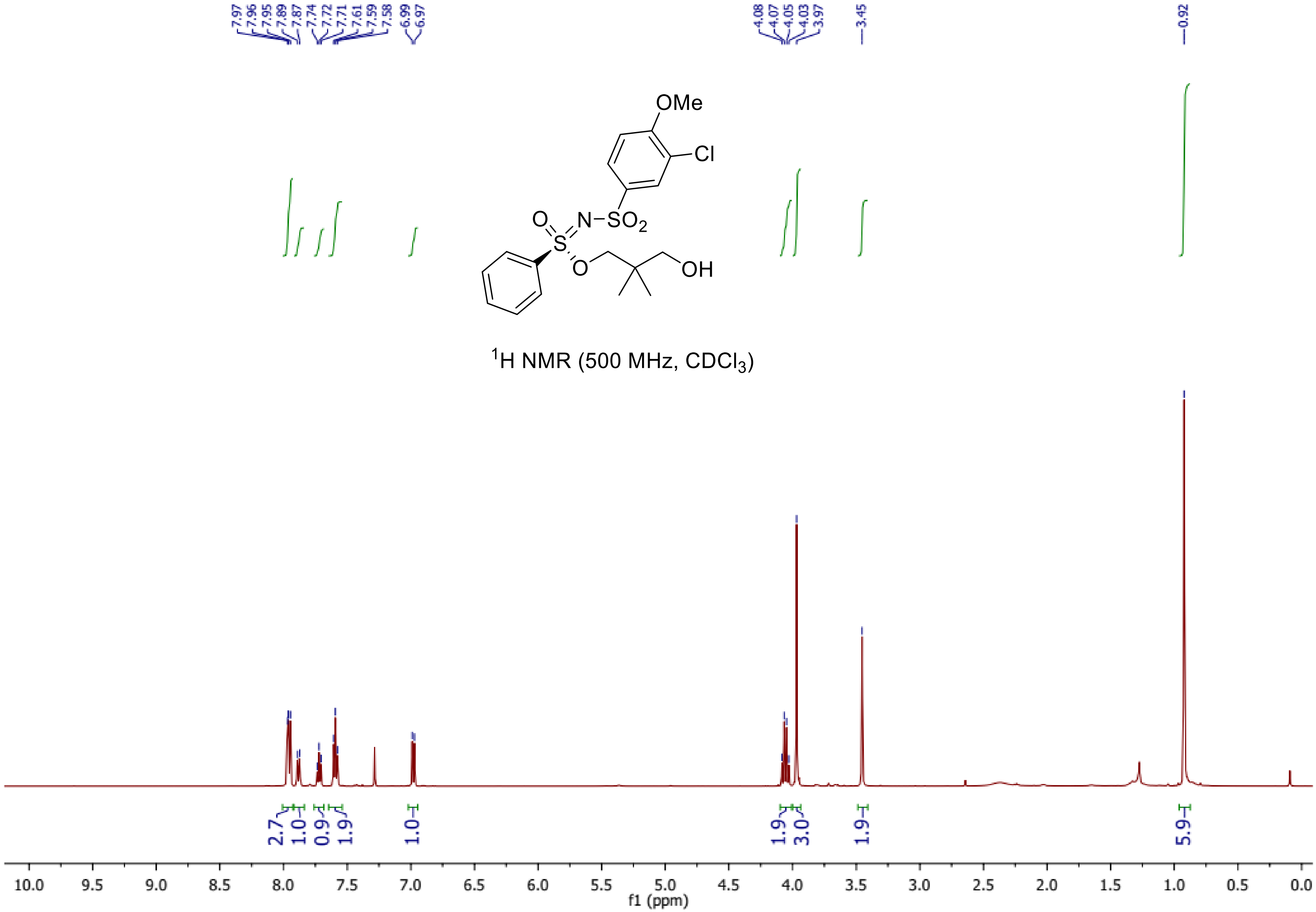

3-Hydroxy-2,2-dimethylpropyl (*R*)-*N*-((3-chloro-4-methoxyphenyl)sulfonyl)benzenesulfonimidate (3ab)

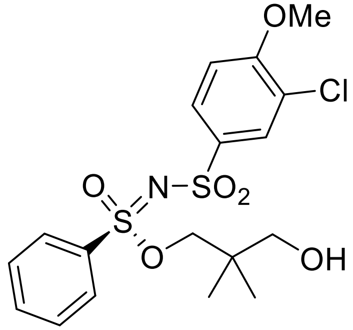

^{13}C NMR (126 MHz, $CDCl_3$)

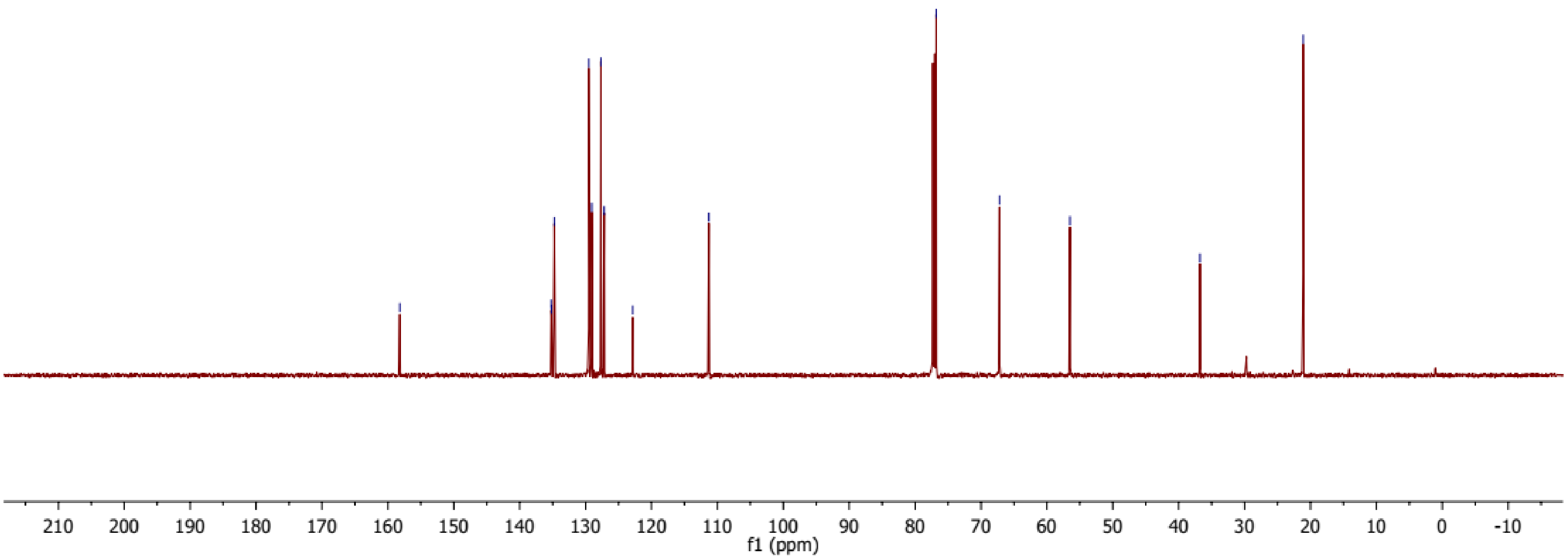

3-Hydroxy-2,2-dimethylpropyl (*R*)-*N*-(cyclopropylsulfonyl)benzenesulfonimidate (3ac)

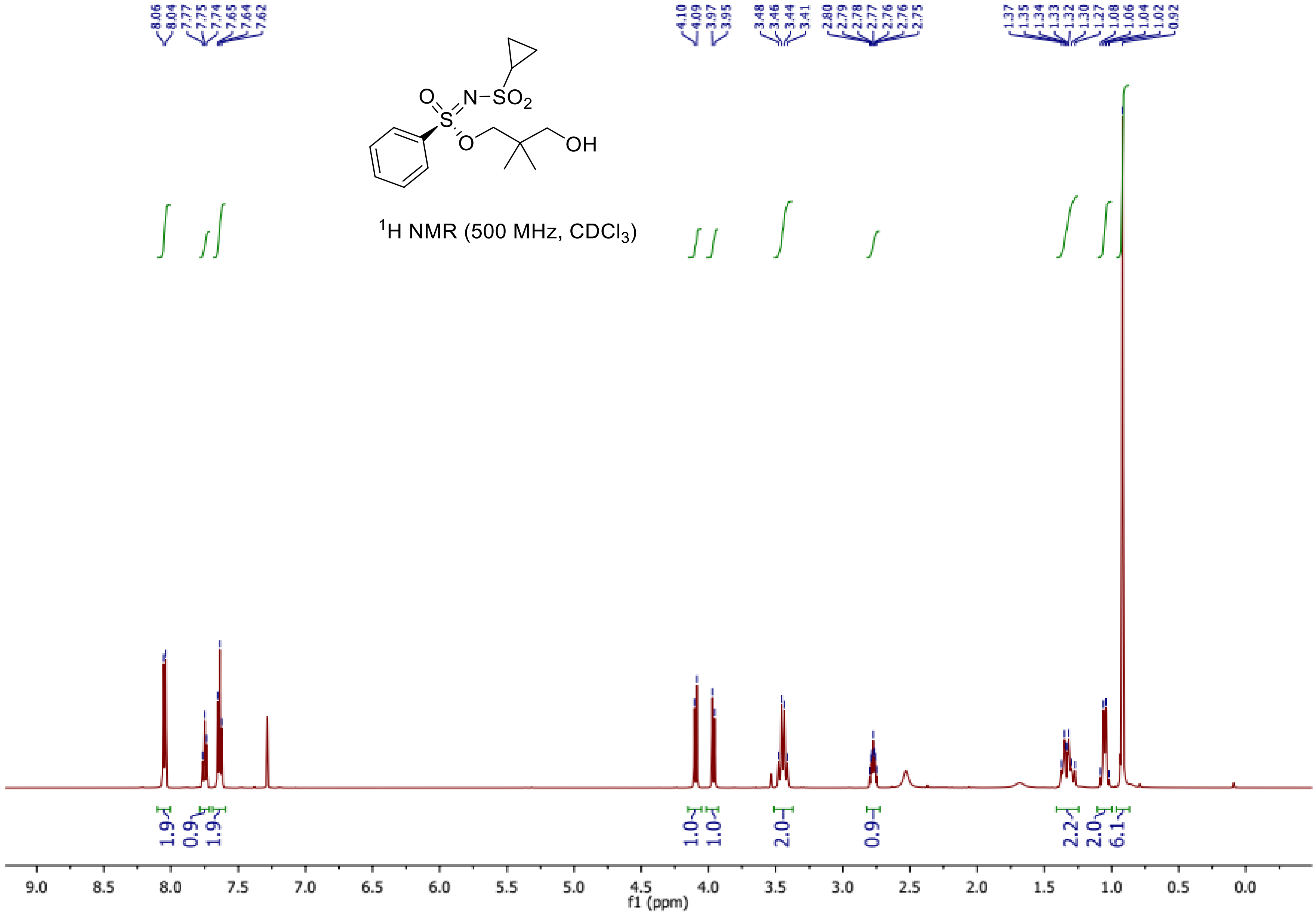

Go to table of contents

3-Hydroxy-2,2-dimethylpropyl (*R*)-*N*-(cyclopropylsulfonyl)benzenesulfonimidate (3ac)

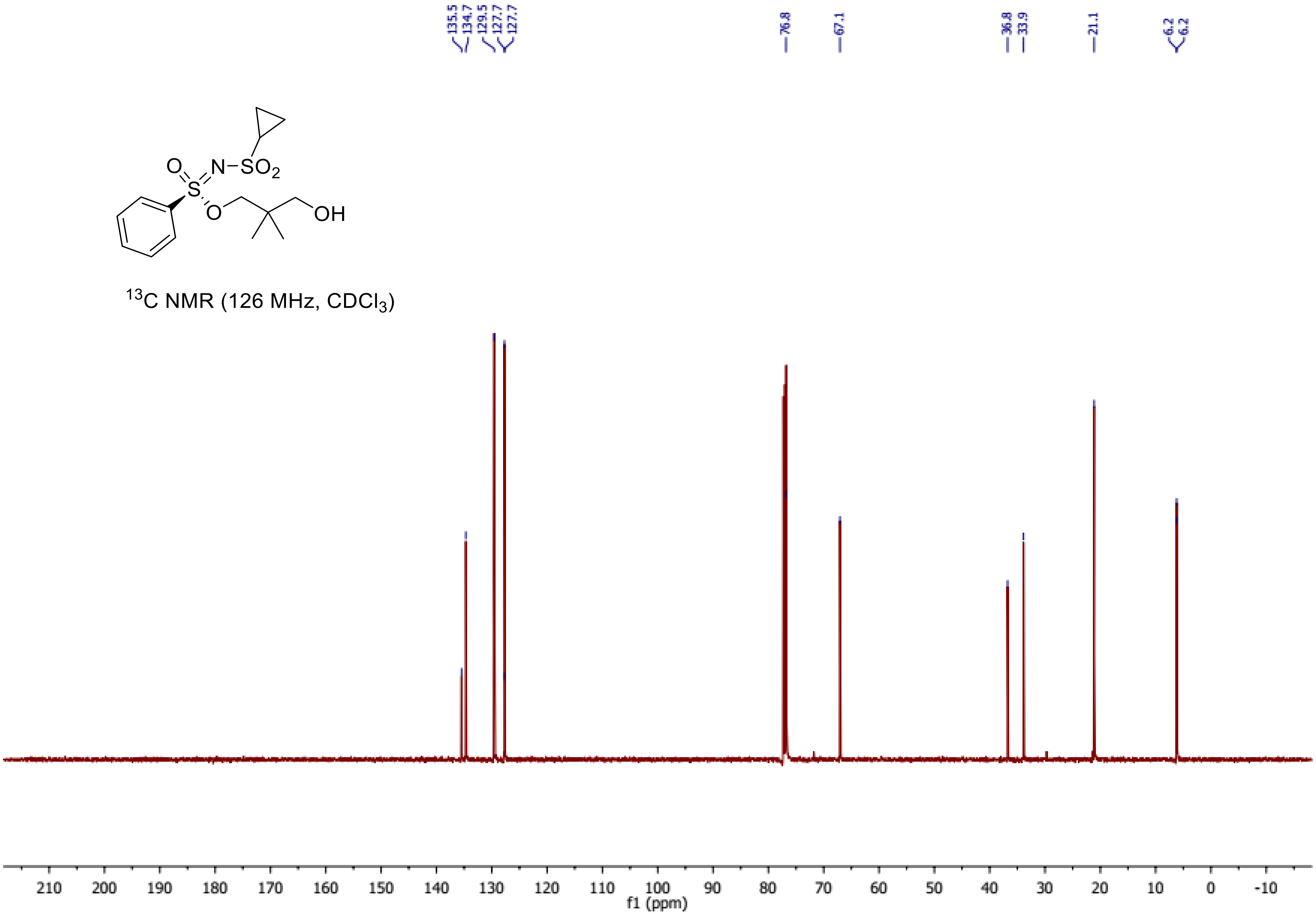

Go to table of contents

(*R*)-*N*-Tosylbenzenesulfonimidoyl fluoride (4)

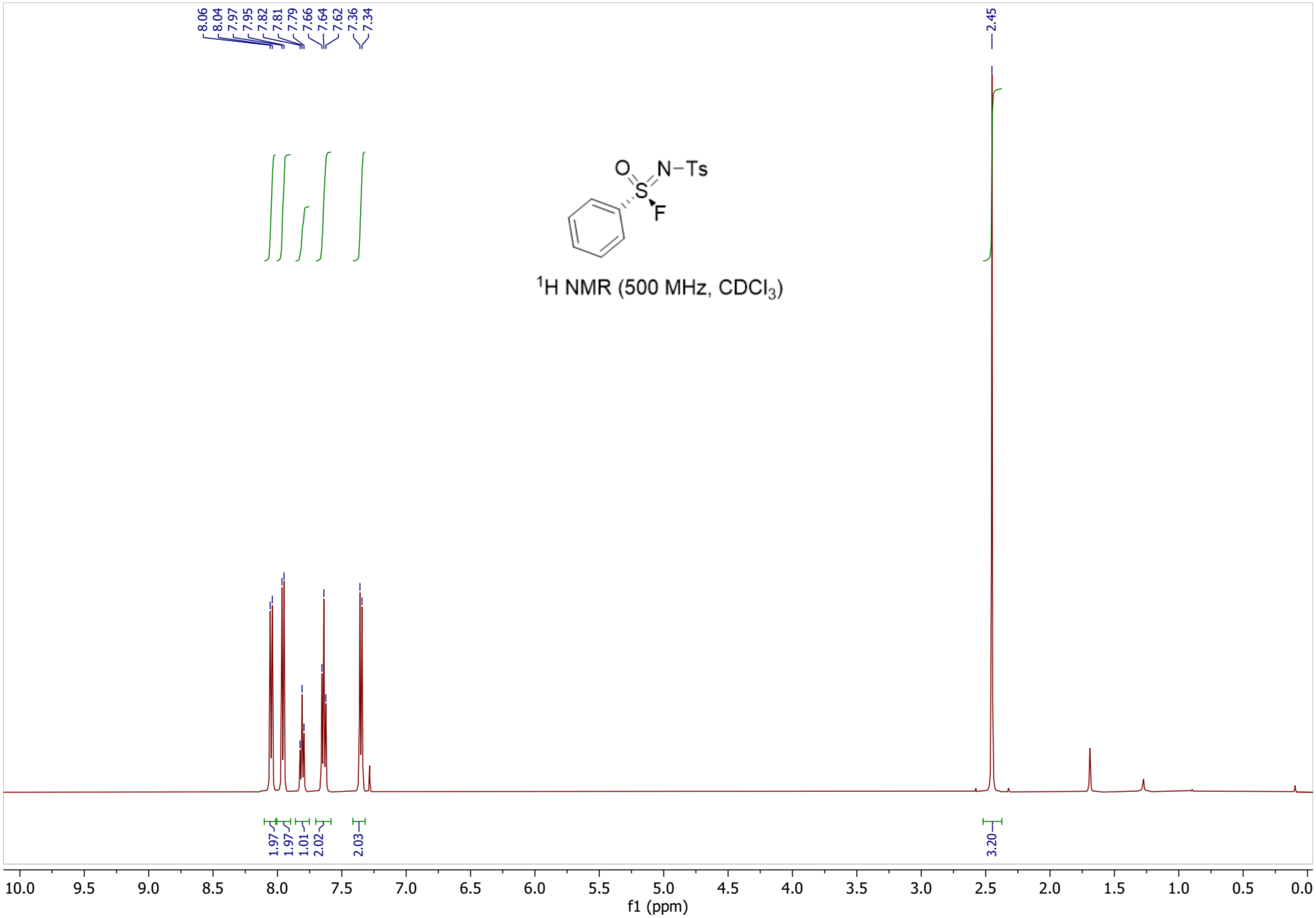

(*R*)-*N*-Tosylbenzenesulfonimidoyl fluoride (4)

Go to table of contents

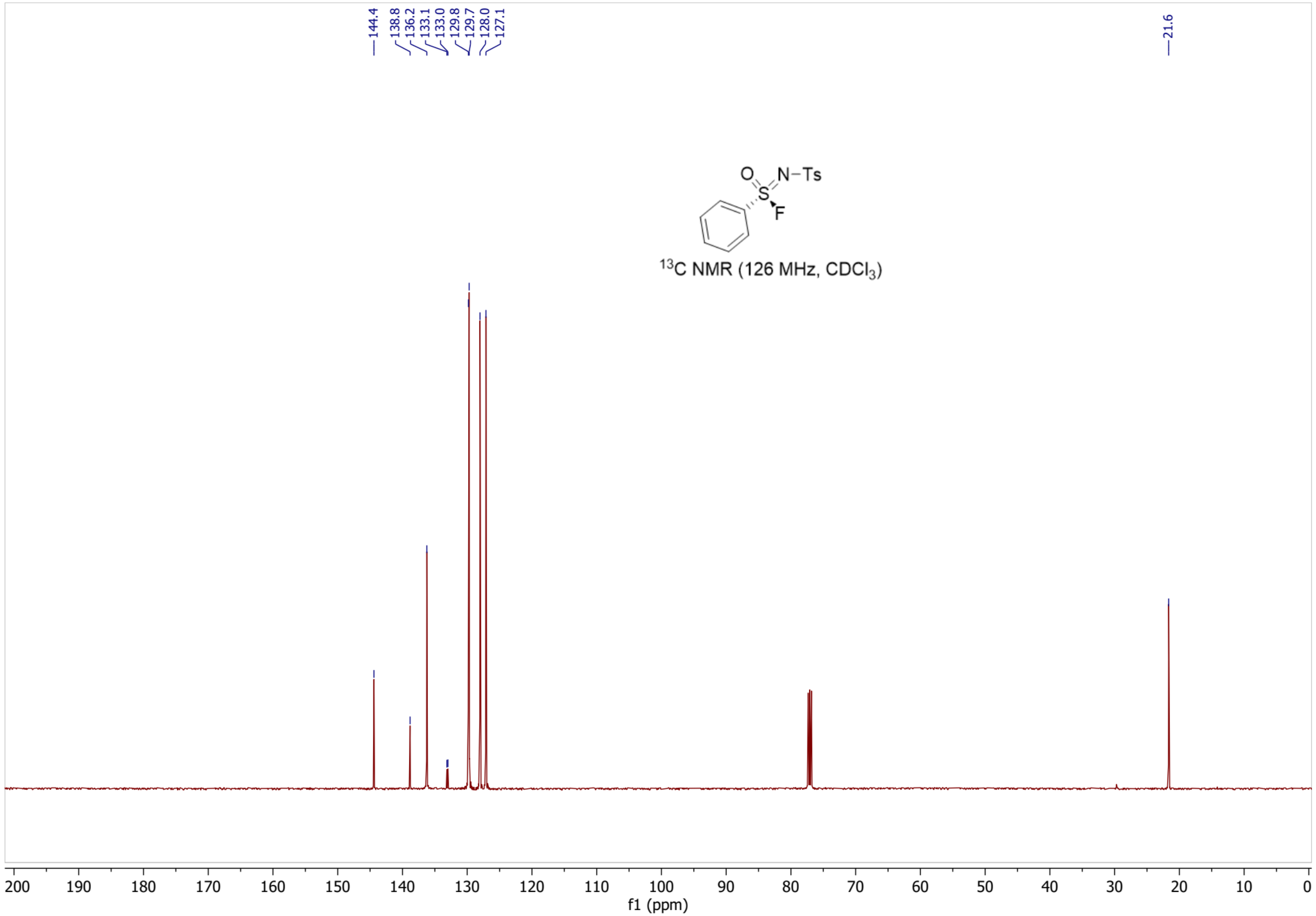

Go to table of contents

(*R*)-*N'*-Tosylbenzenesulfonimidoyl azide (5)

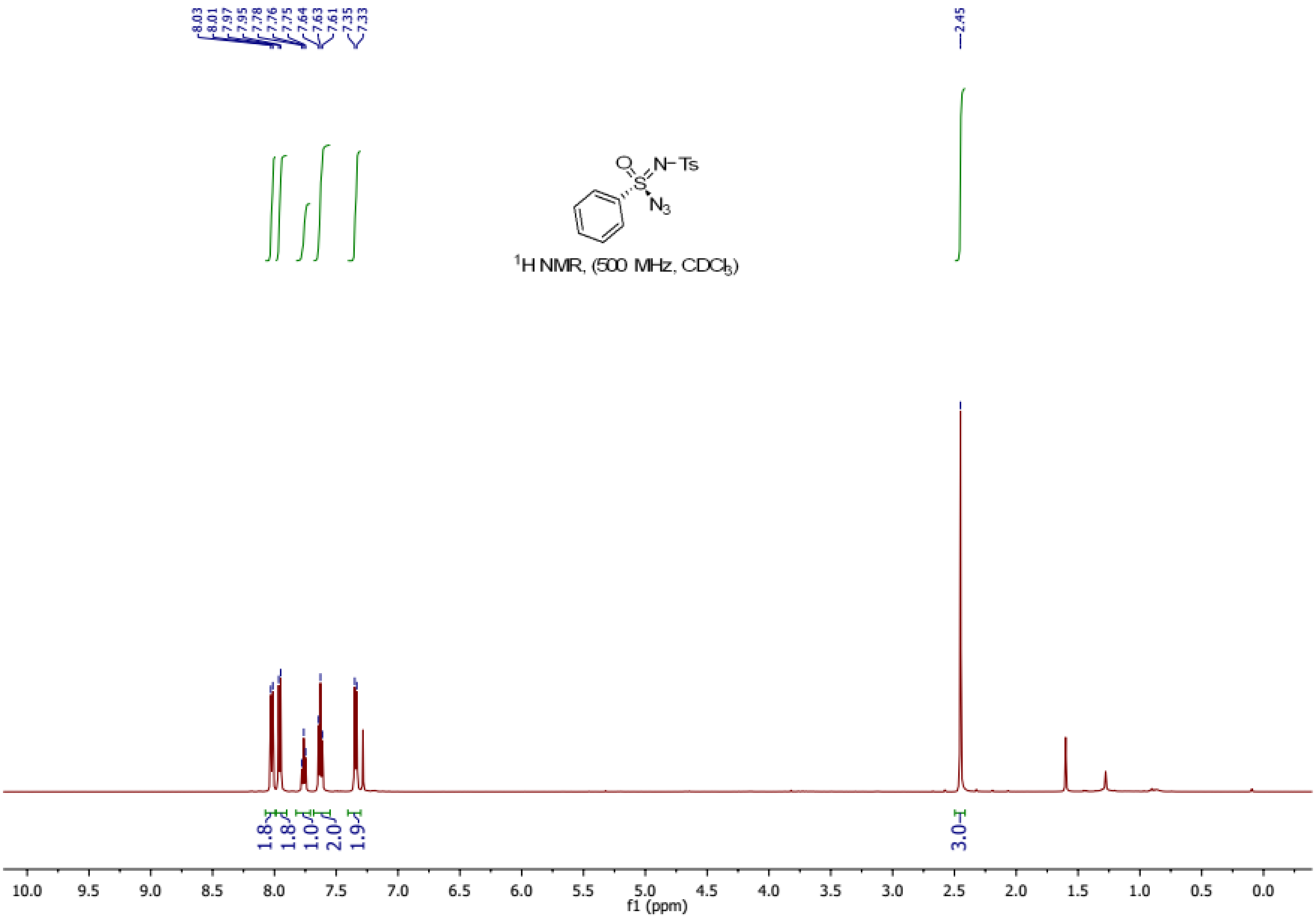

Go to table of contents

(*R*)-*N'*-Tosylbenzenesulfonimidoyl azide (5)

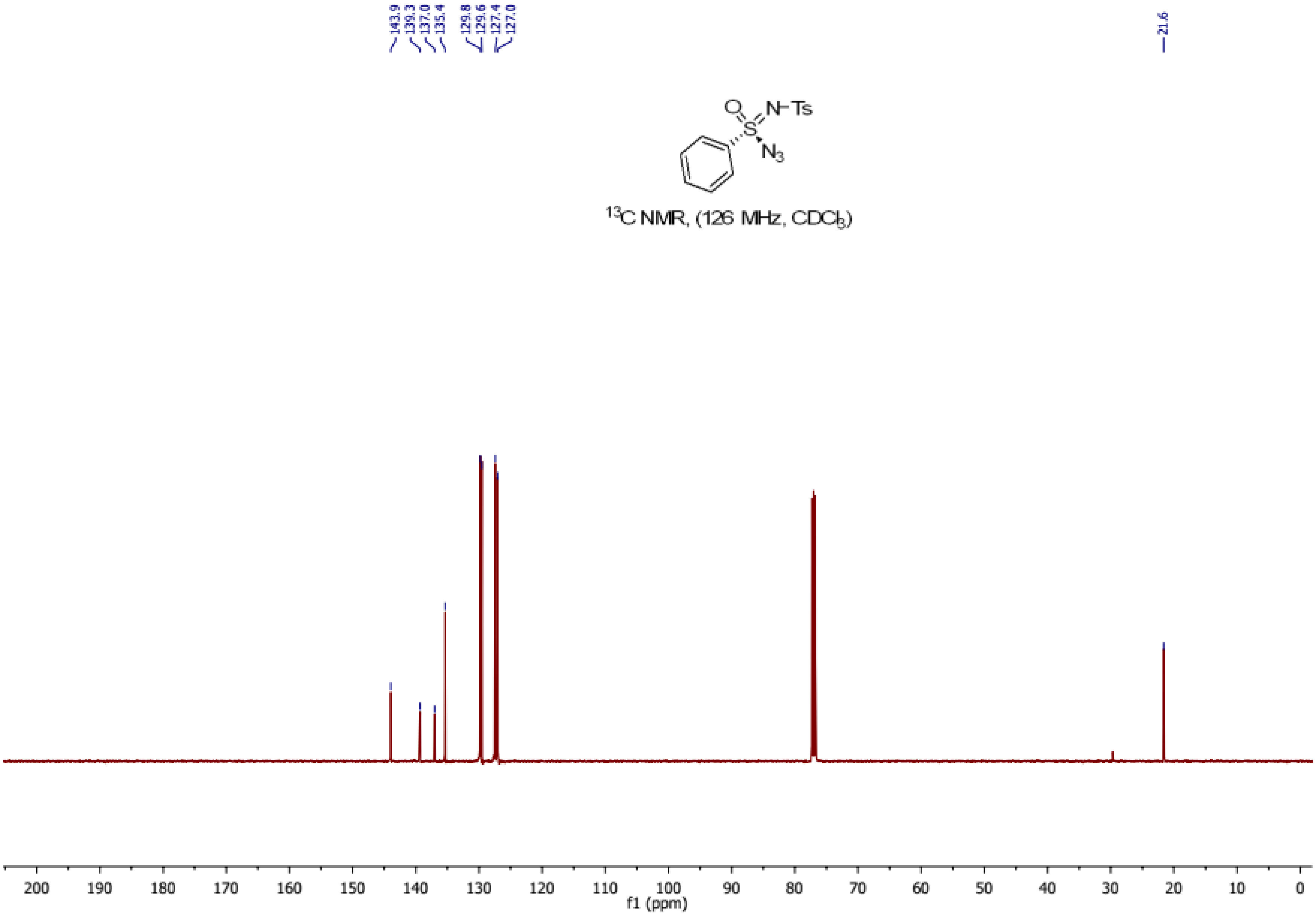

Go to table of contents

Phenyl (*S*)-*N*-tosylbenzenesulfonimidate (6)

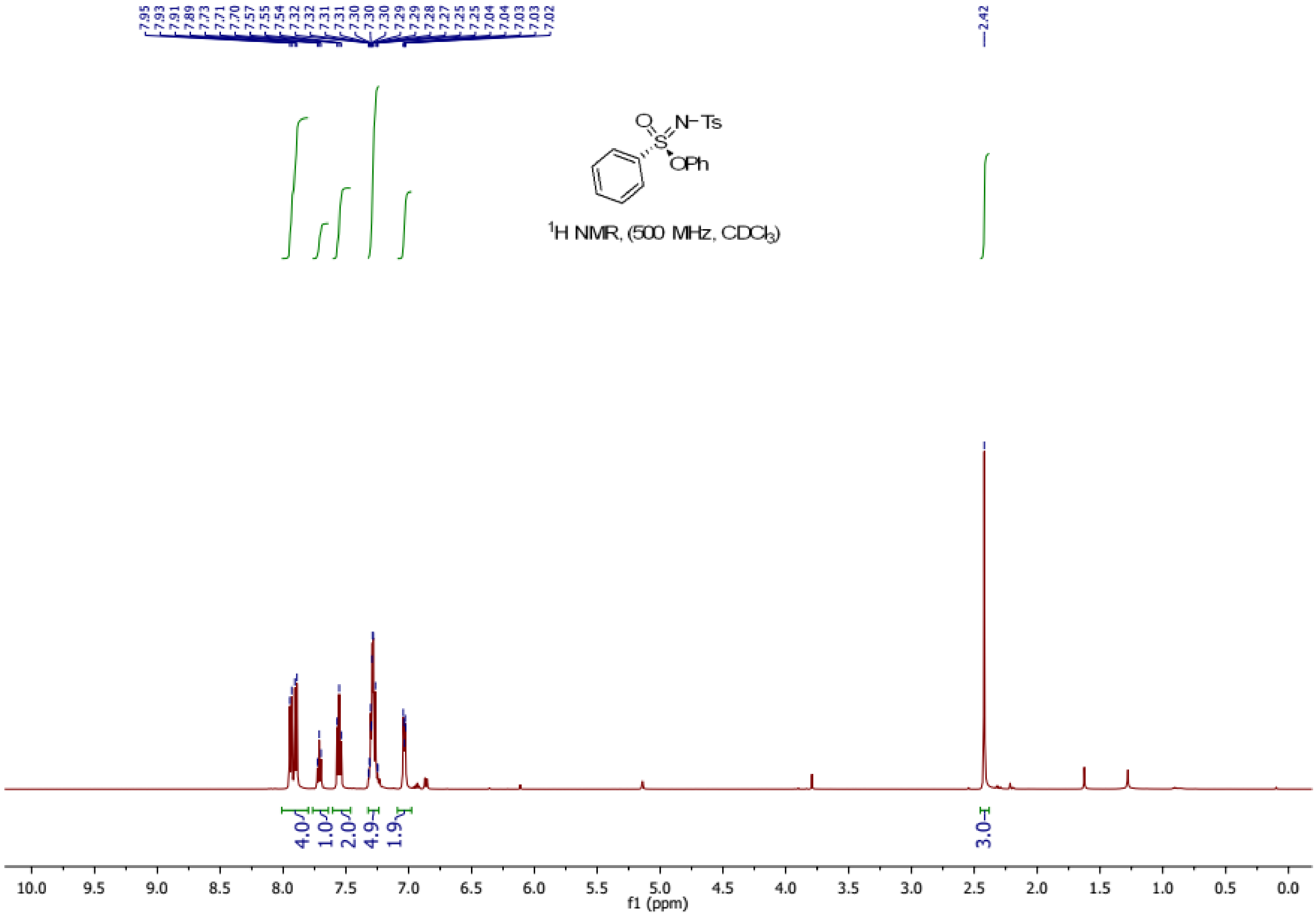

Go to table of contents

Phenyl (*S*)-*N*-tosylbenzenesulfonimidate (6)

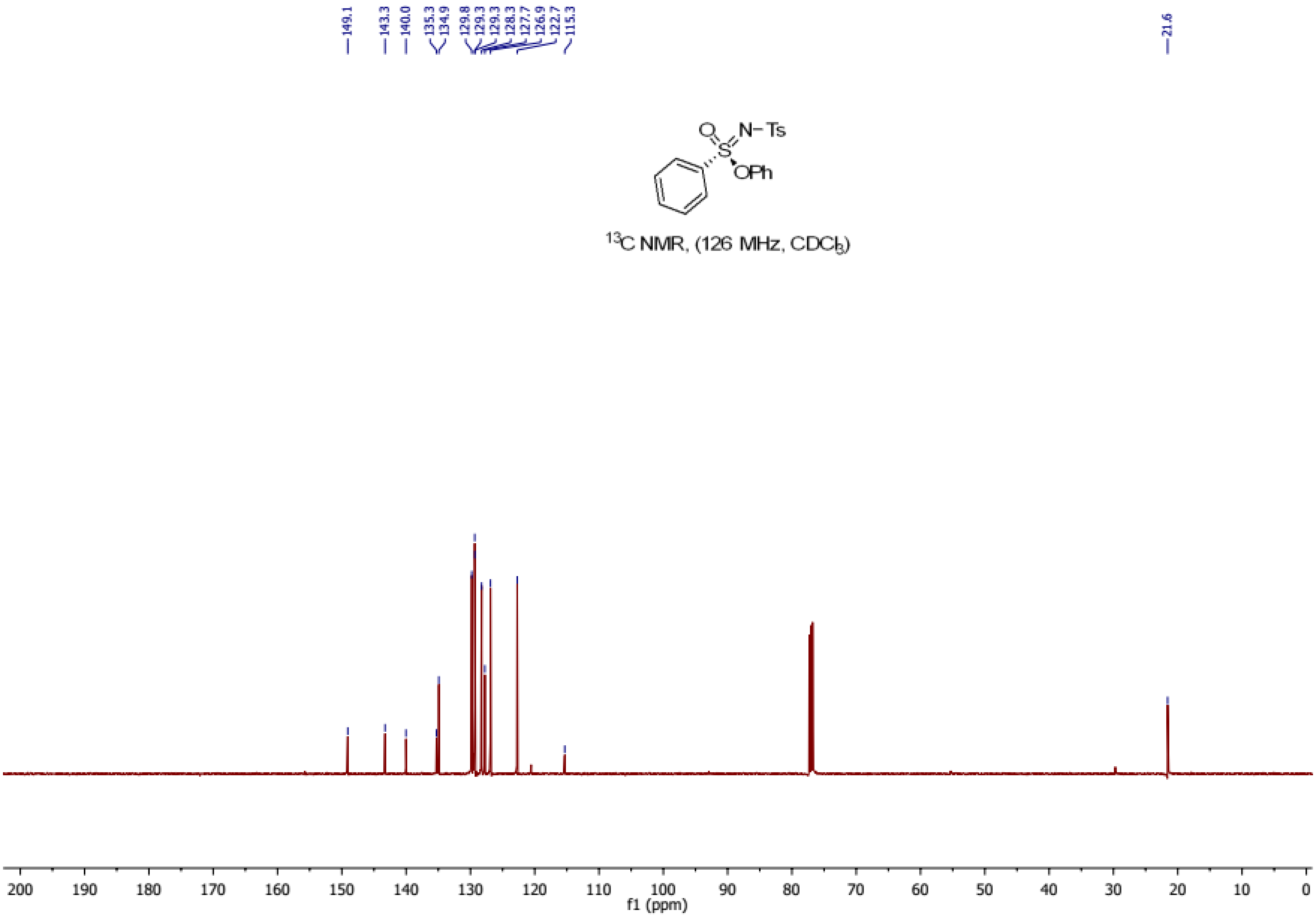

(*R*)-4-Methyl-*N*-(oxo(phenyl)(piperidin-1-yl)- λ⁶-sulfaneylidene)benzenesulfonamide (7)

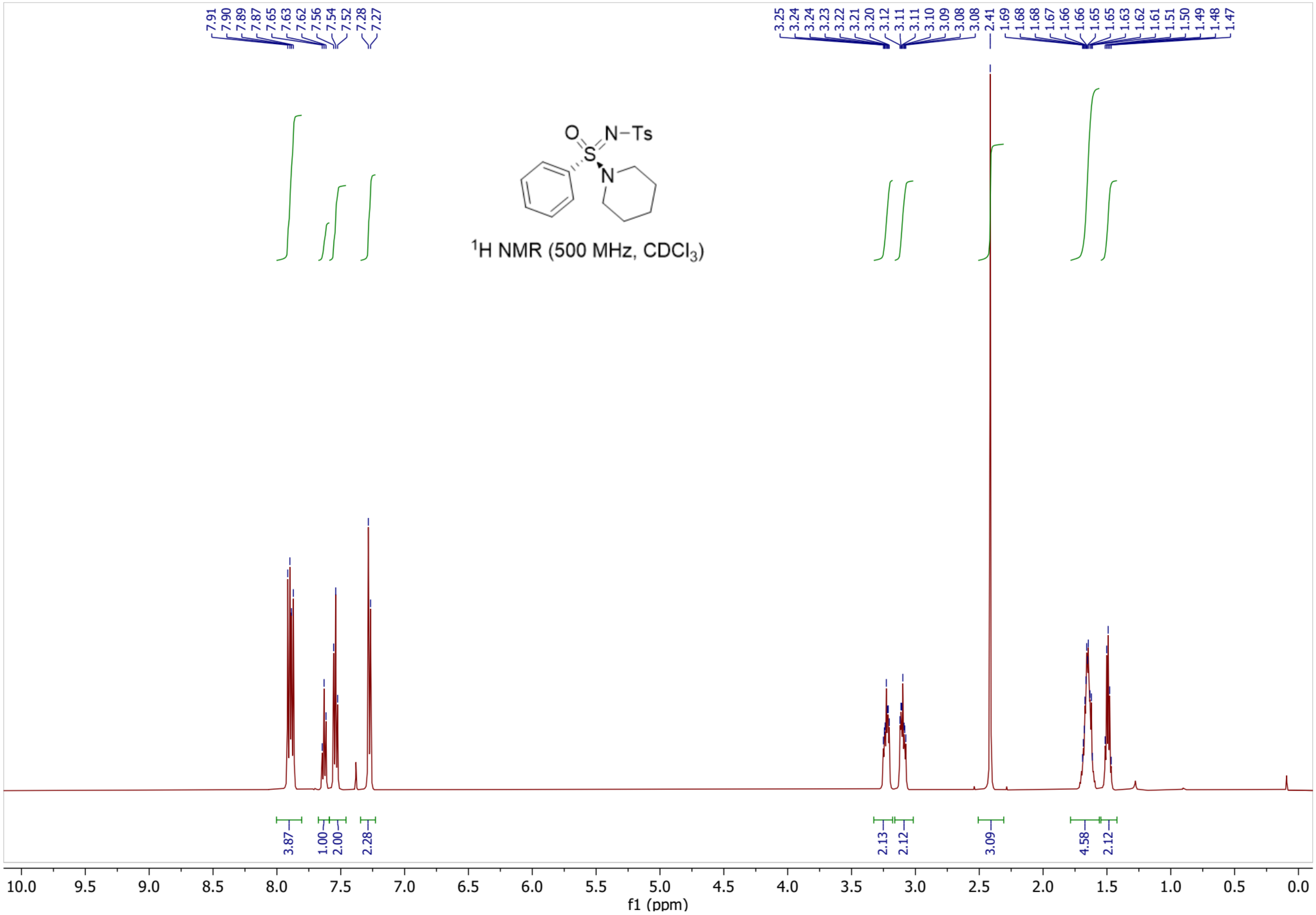

Go to table of contents

(*R*)-4-Methyl-*N*-(oxo(phenyl)(piperidin-1-yl)- λ⁶-sulfaneylidene)benzenesulfonamide (7)

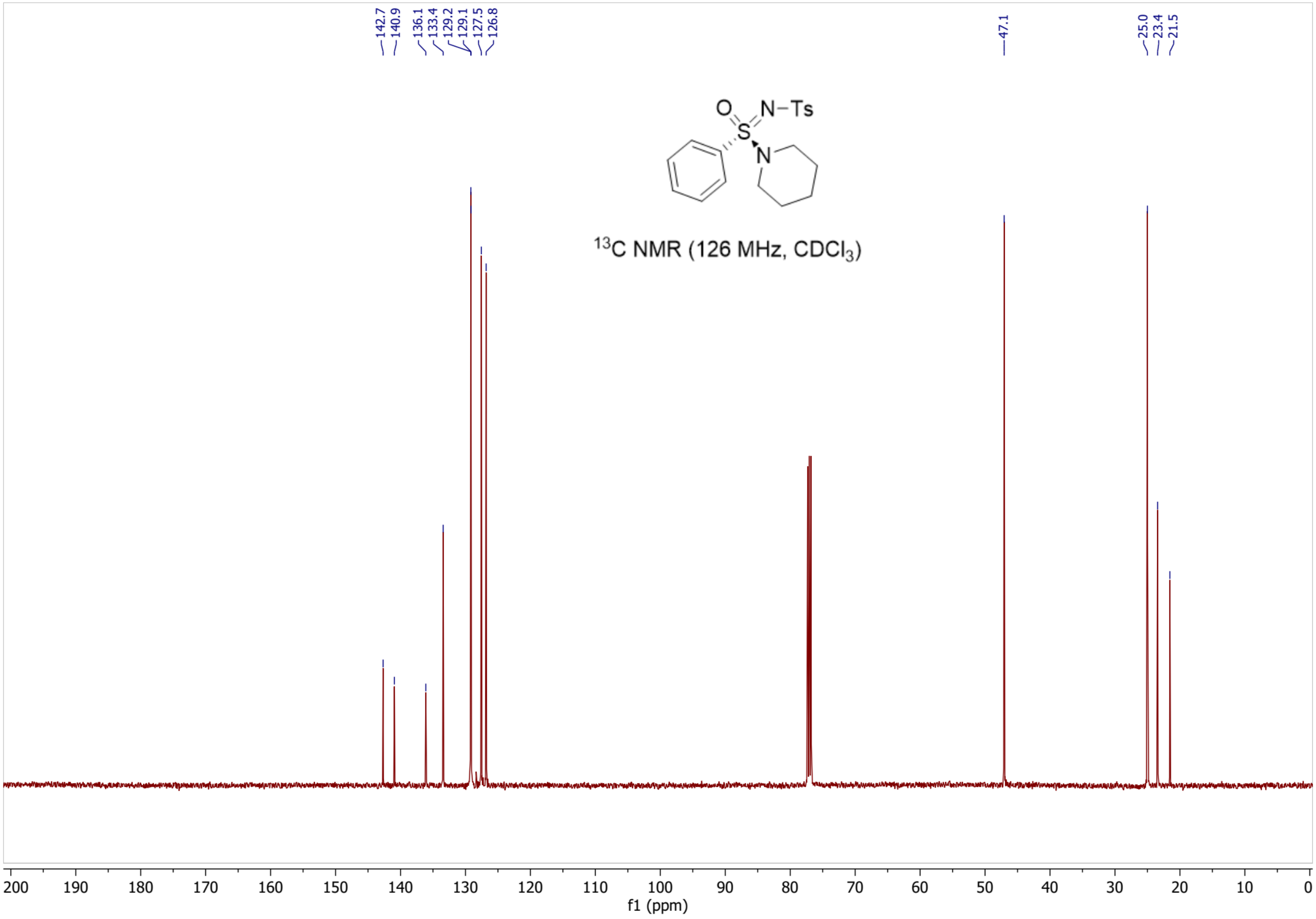

Go to table of contents

(*S*)-*N*-((4-(8-Chloro-5,6-dihydro-11*H*-benzo[5,6]cyclohepta[1,2-b]pyridin-11-ylidene)piperidin-1-yl)(oxo)(phenyl)- λ⁶-sulfaneylidene)-4-methylbenzenesulfonamide (8)

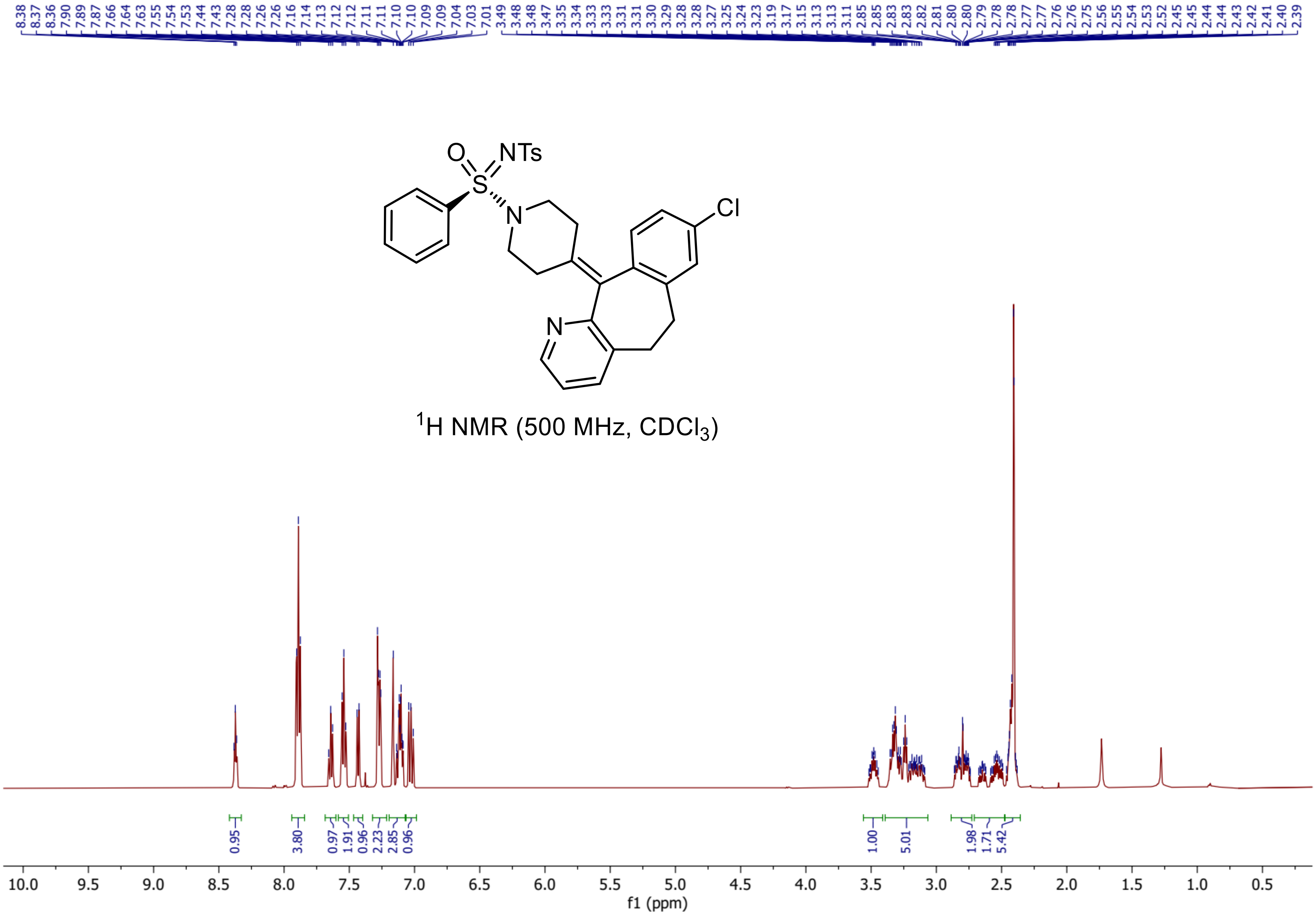

Go to table of contents

(*S*)-*N*-((4-(8-Chloro-5,6-dihydro-11*H*-benzo[5,6]cyclohepta[1,2-b]pyridin-11-ylidene)piperidin-1-yl)(oxo)(phenyl)- λ⁶-sulfaneylidene)-4-methylbenzenesulfonamide (8)

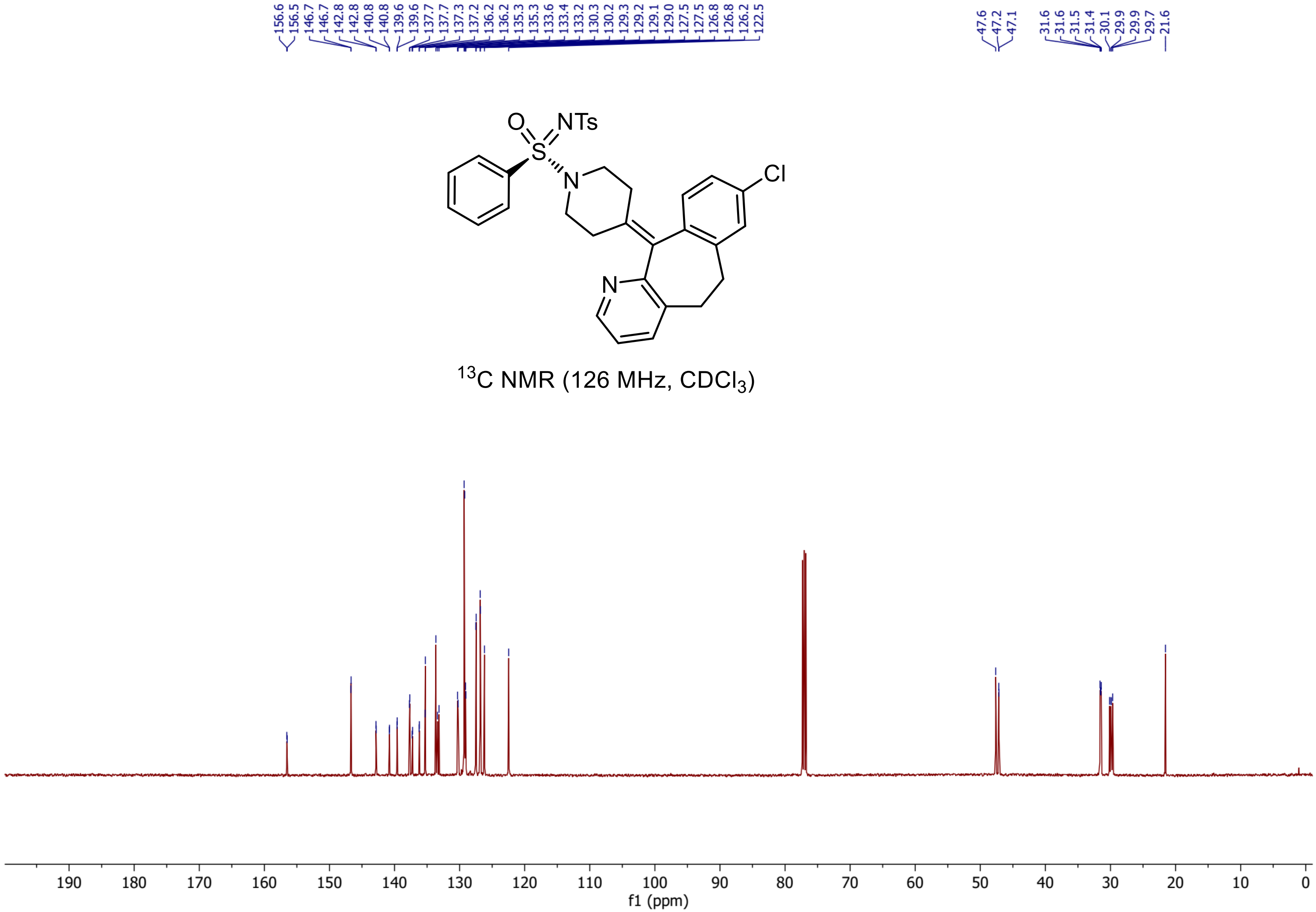

Go to table of contents

(*S*)-*N*-((4-(6-Fluorobenzo[d]isoxazol-3-yl)piperidin-1-yl)(oxo)(phenyl)-λ⁶-sulfaneylidene)-4-methylbenzenesulfonamide (9)

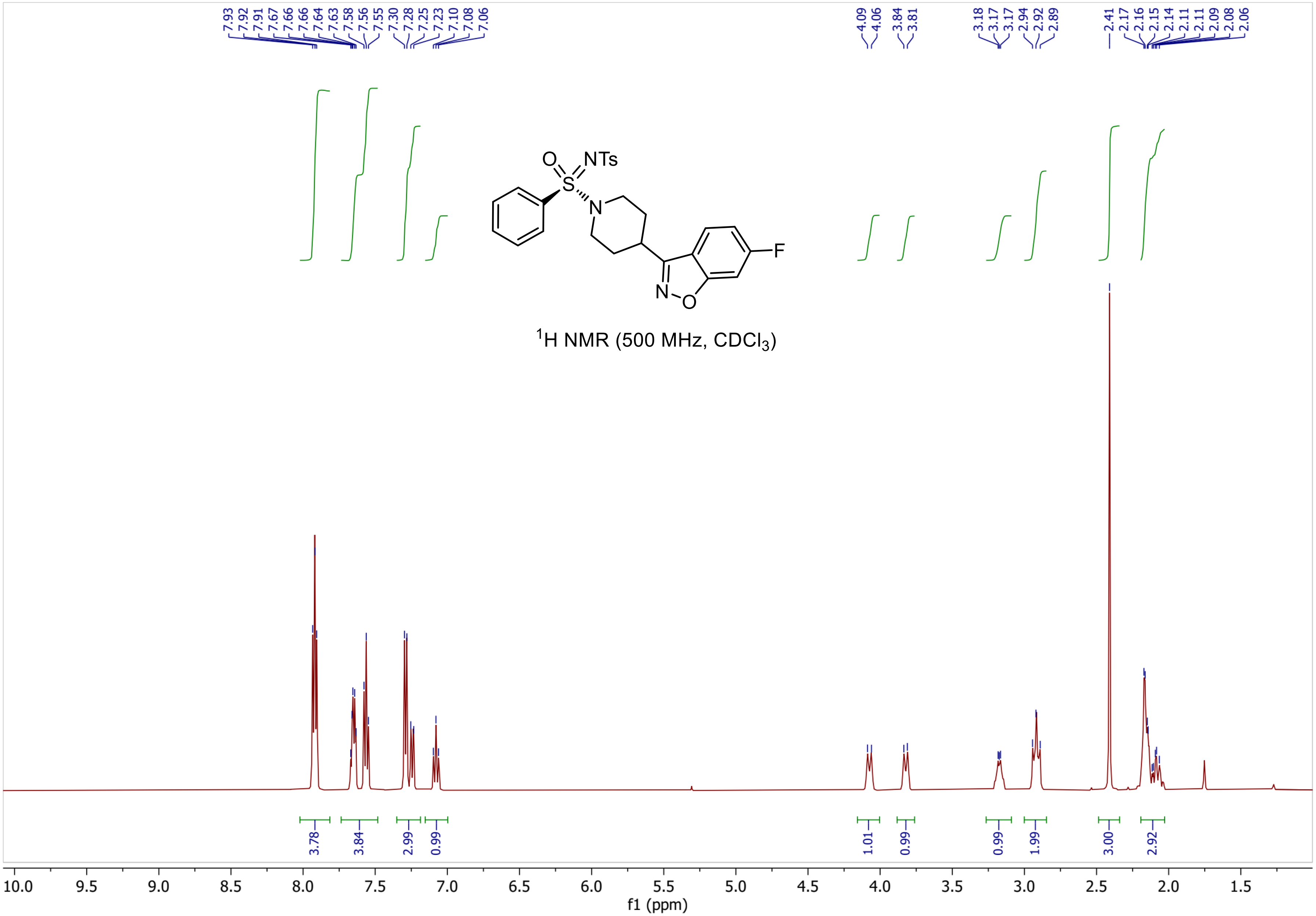

Go to table of contents

(*S*)-*N*-((4-(6-Fluorobenzo[d]isoxazol-3-yl)piperidin-1-yl)(oxo)(phenyl)-λ^6-sulfaneylidene)-4-methylbenzenesulfonamide (9)

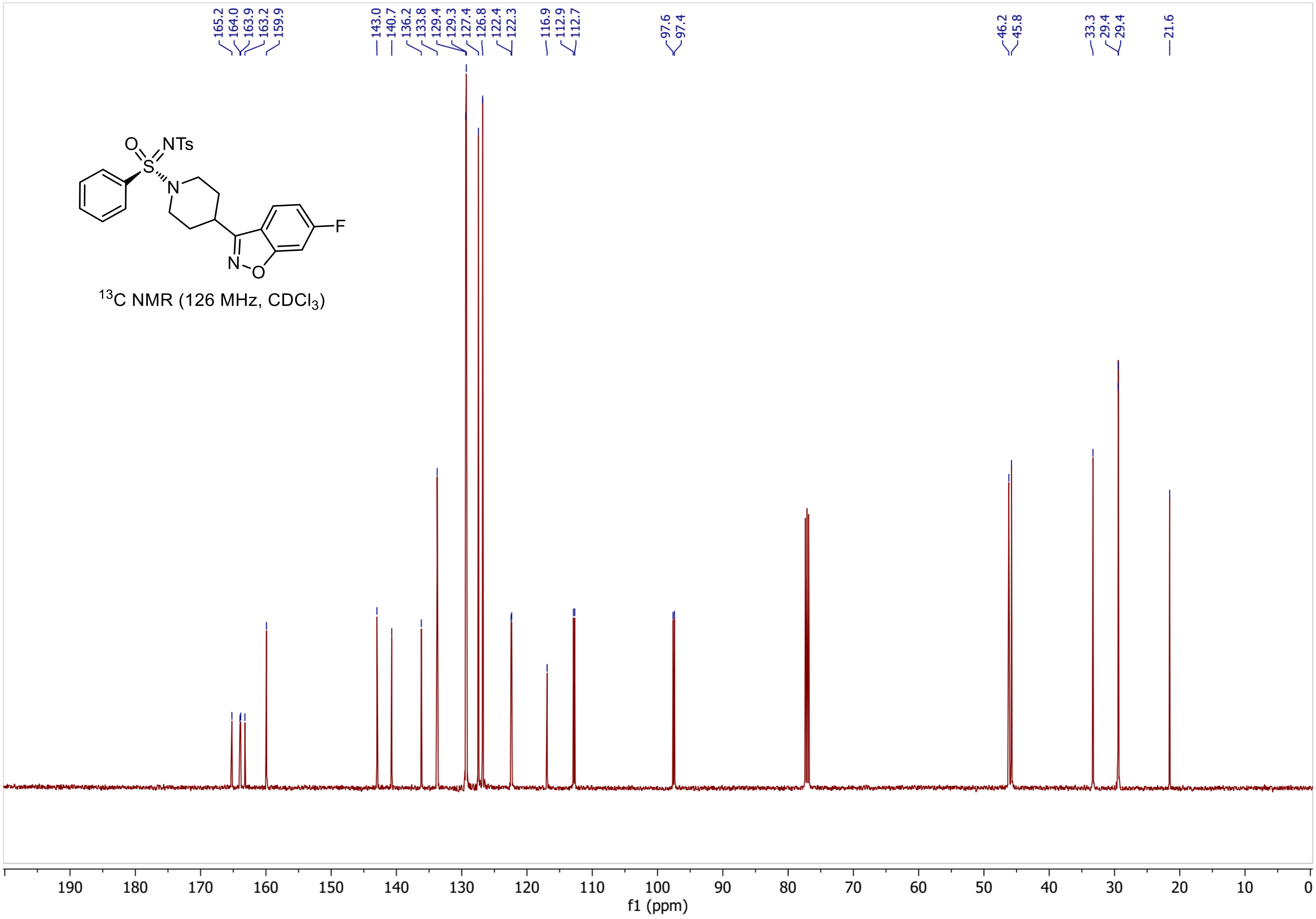

Methyl (*S*)-2-((*tert*-butoxycarbonyl)amino)-3-(4-(((*R*)-*N*-tosylphenylsulfonimidoyl)oxy)phenyl)propanoate (10)

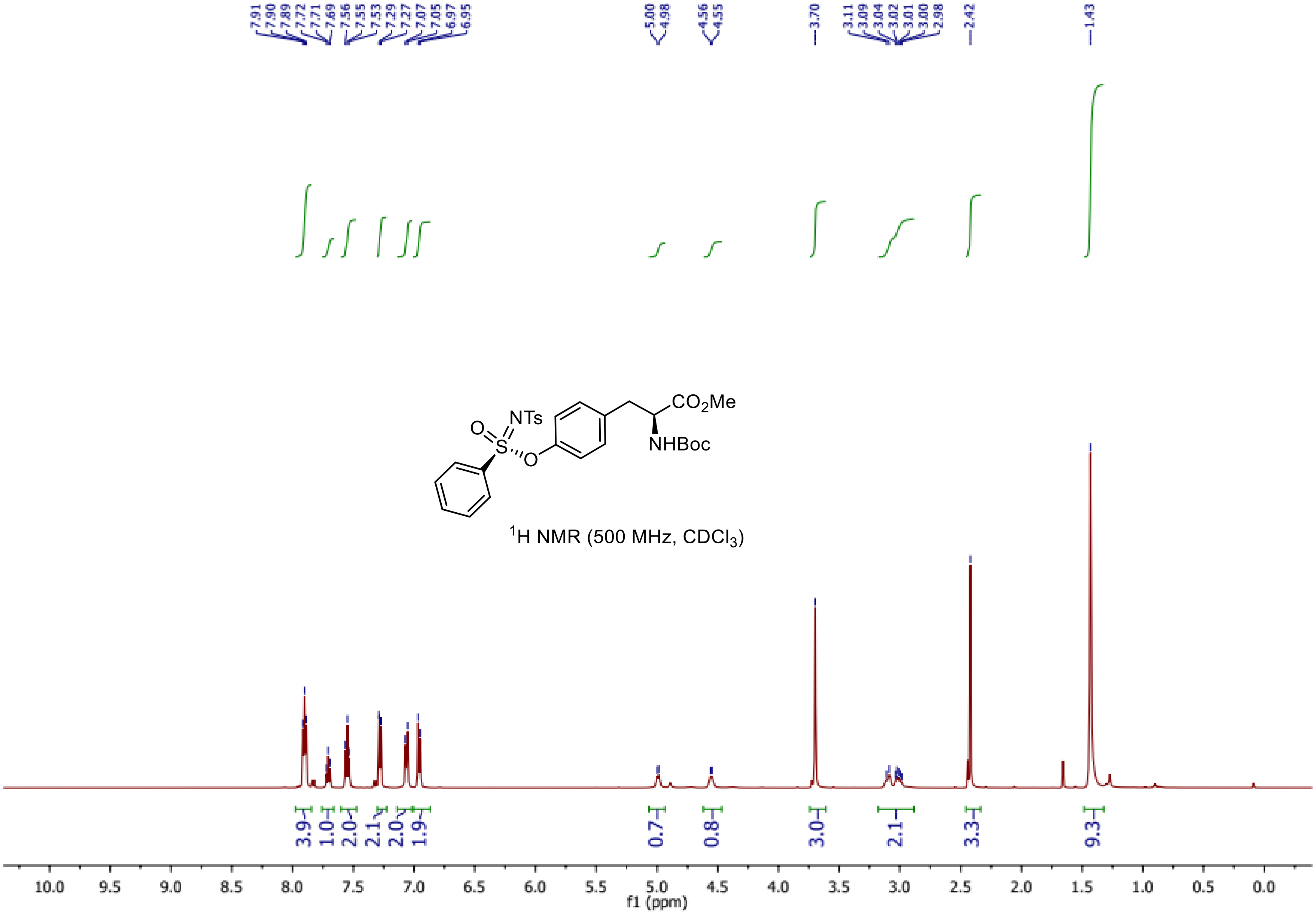

Go to table of contents

Methyl (*S*)-2-((*tert*-butoxycarbonyl)amino)-3-(4-(((*R*)-*N*-tosylphenylsulfonimidoyl)oxy)phenyl)propanoate (10)

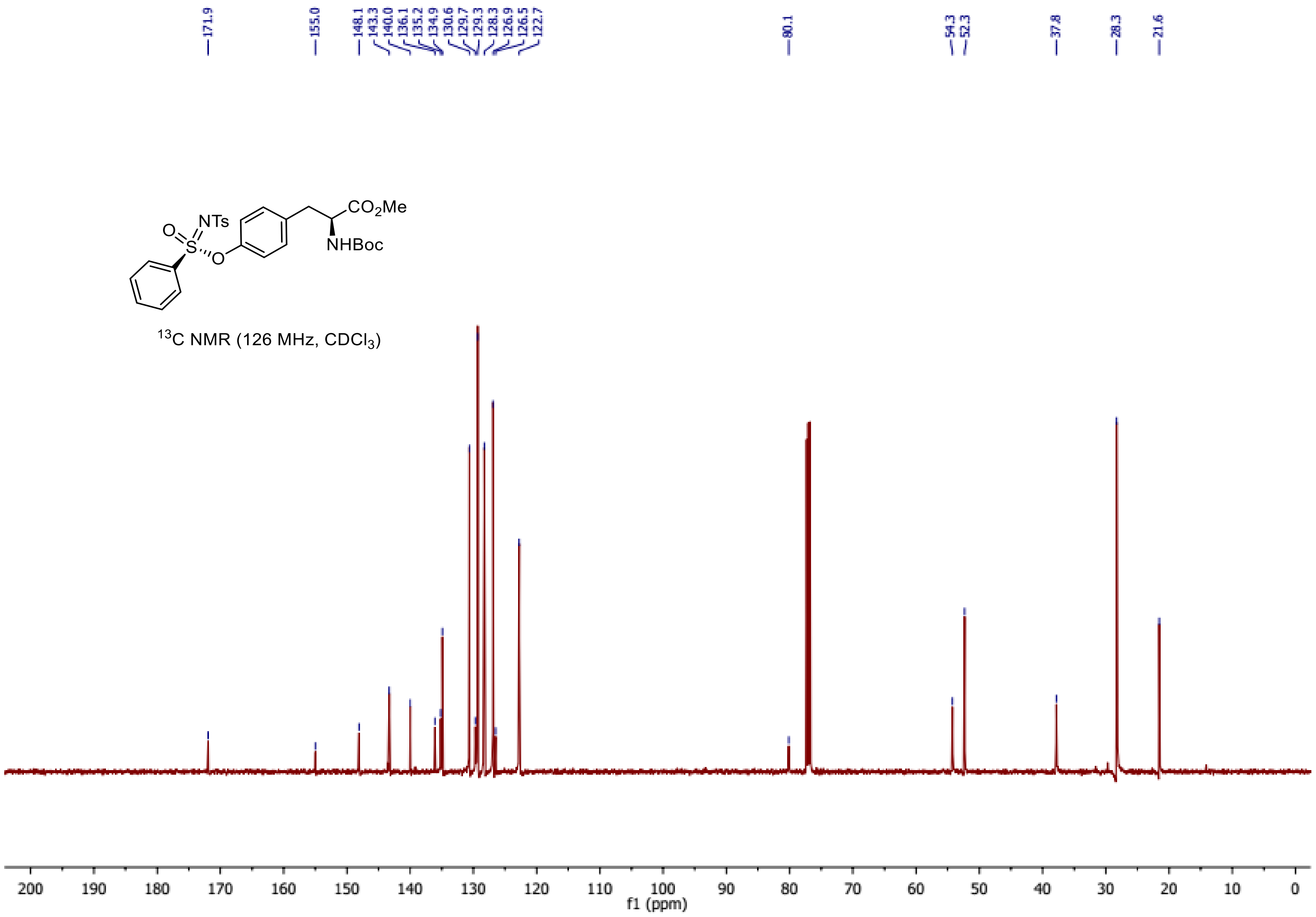

^{13}C NMR (126 MHz, $CDCl_3$)

Go to table of contents

4-((2*S*,3*R*)-1-(4-Fluorophenyl)-3-((*S*)-3-(4-fluorophenyl)-3-hydroxypropyl)-4-oxoazetidin-2-yl)phenyl (*R*)-*N*-tosylbenzenesulfonimidate (11)

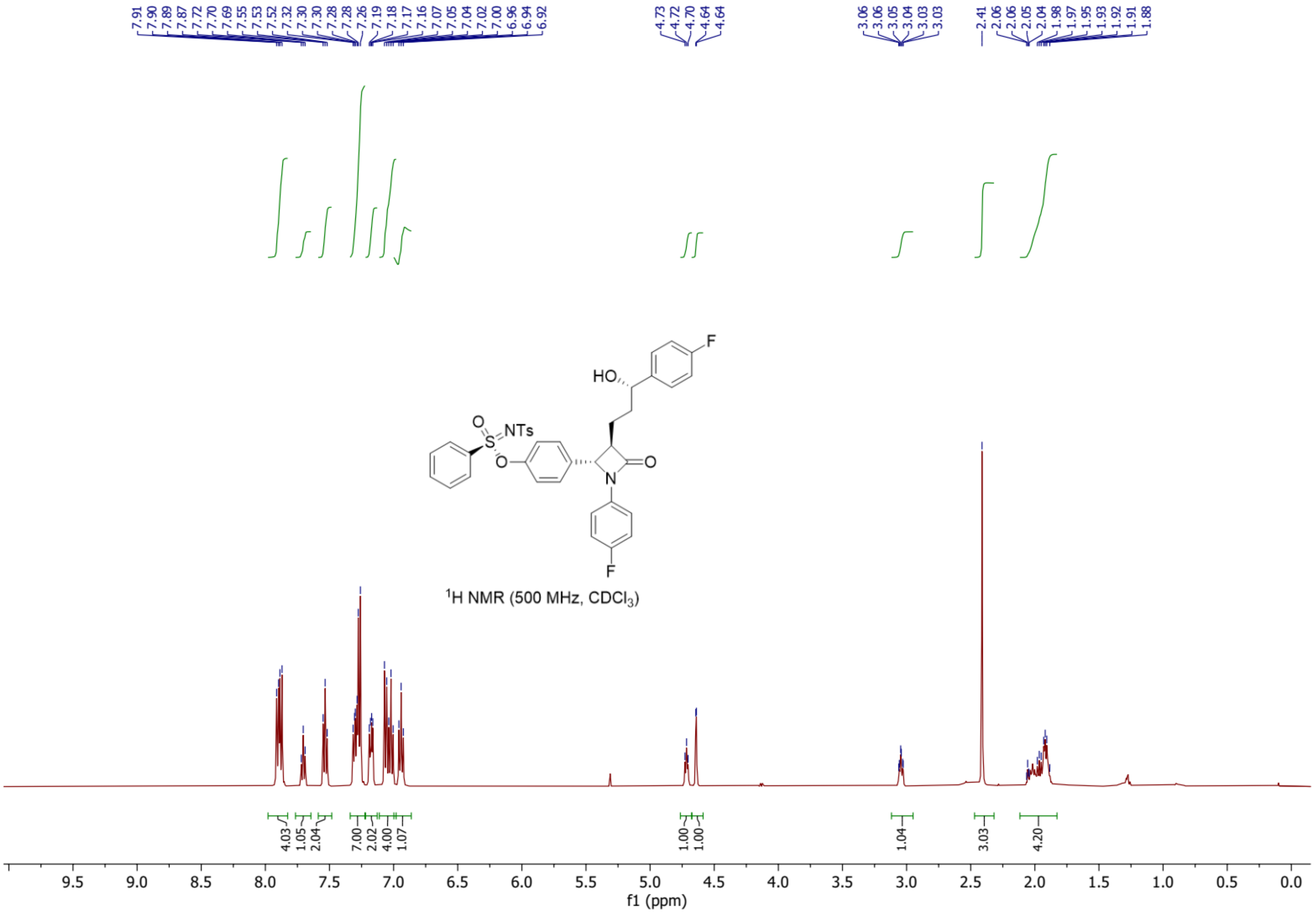

Go to table of contents

4-((2*S*,3*R*)-1-(4-Fluorophenyl)-3-((*S*)-3-(4-fluorophenyl)-3-hydroxypropyl)-4-oxoazetidin-2-yl)phenyl (*R*)-*N*-tosylbenzenesulfonimidate (11)

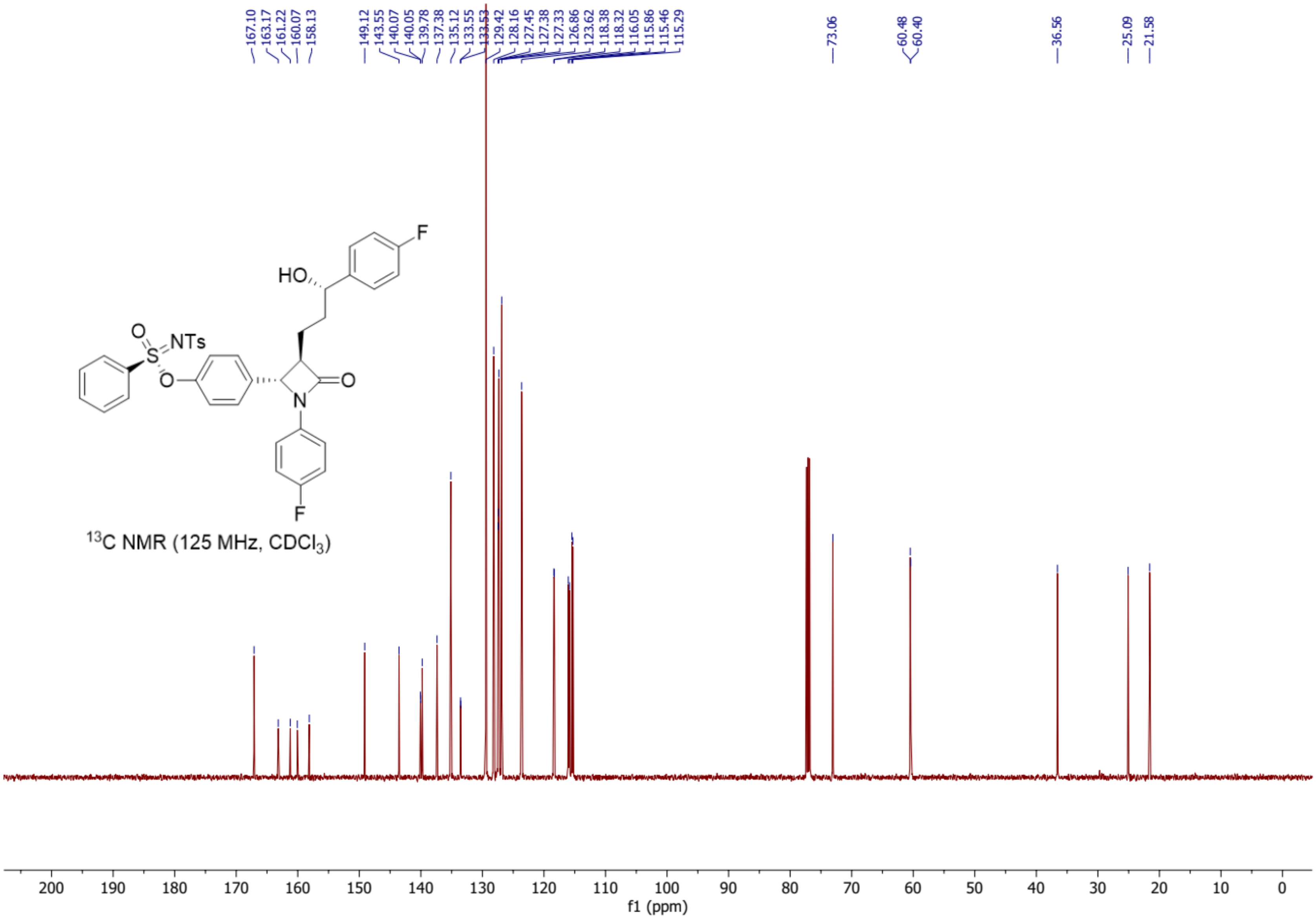

Go to table of contents

2,2-Dimethyl-3-(((R)-N-tosylphenylsulfonimidoyl)oxy)propyl (3⁵S,9¹R,9²R,5S)-5-(tert-butyl)-17-methoxy-4,7-dioxo-2,8-dioxa-6-aza-1(2,3)-quinoxalina-3(3,1)-pyrrolidina-9(1,2)-cyclopropanacyclotetradecaphane-3⁵-carboxylate (12)

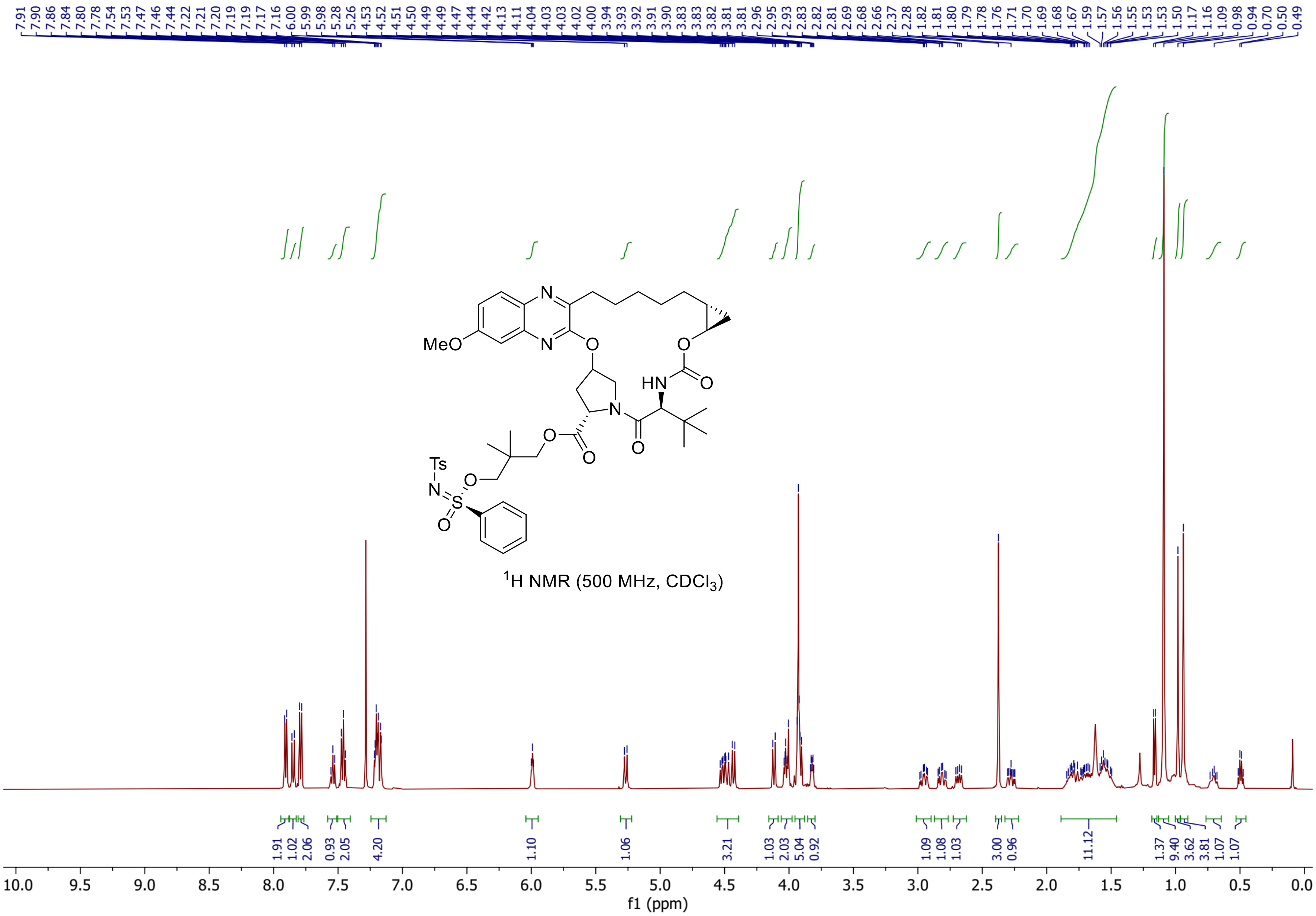

2,2-Dimethyl-3-(((*R*)-*N*-tosylphenylsulfonimidoyl)oxy)propyl (35*S*,91*R*,92*R*,5*S*)-5-(*tert*-butyl)-17-methoxy-4,7-dioxo-2,8-dioxa-6-aza-1(2,3)-quinoxalina-3(3,1)-pyrrolidina-9(1,2)-cyclopropanacyclotetradecaphane-35-carboxylate (12)

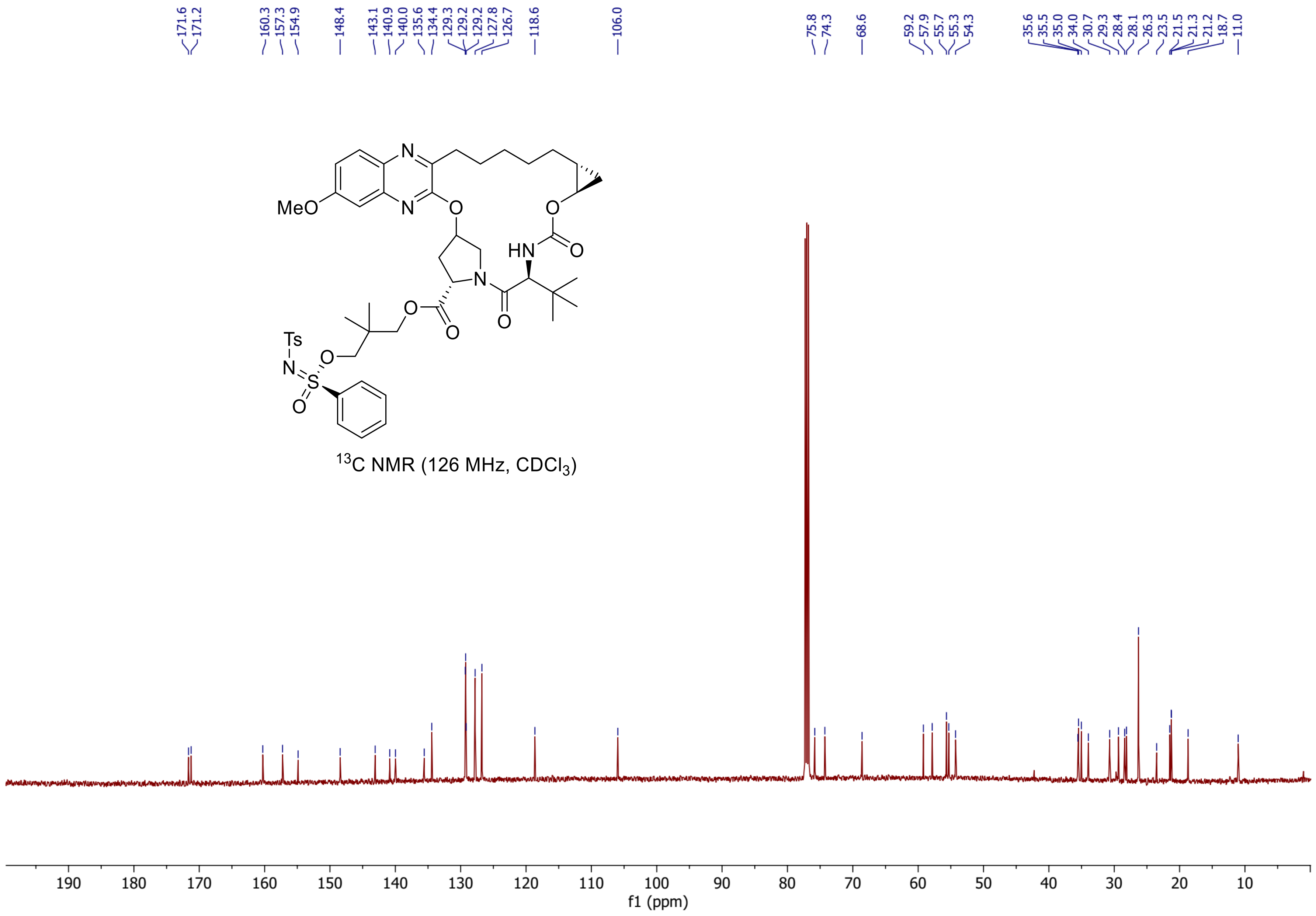

Go to table of contents

(*R*)-2-(2,2-dimethyl-3-((*N*-tosylphenylsulfonimidoyl)oxy)propoxy)-2-oxoethyl 2-(1-(4-chlorobenzoyl)-5-methoxy-2-methyl-1*H*-indol-3-yl)acetate (13)

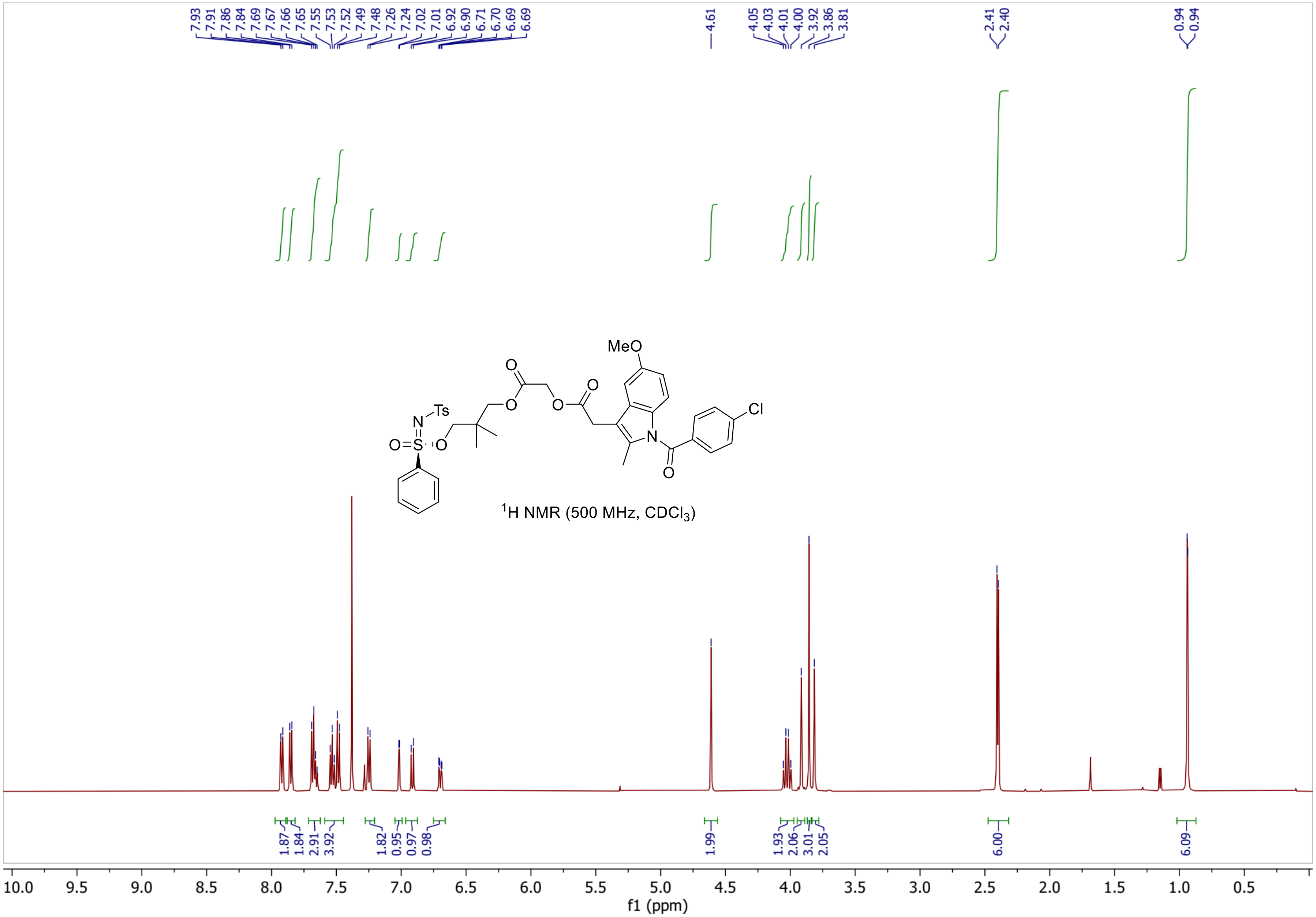

Go to table of contents

(*R*)-2-(2,2-dimethyl-3-((*N*-tosylphenylsulfonimidoyl)oxy)propoxy)-2-oxoethyl 2-(1-(4-chlorobenzoyl)-5-methoxy-2-methyl-1*H*-indol-3-yl)acetate (13)

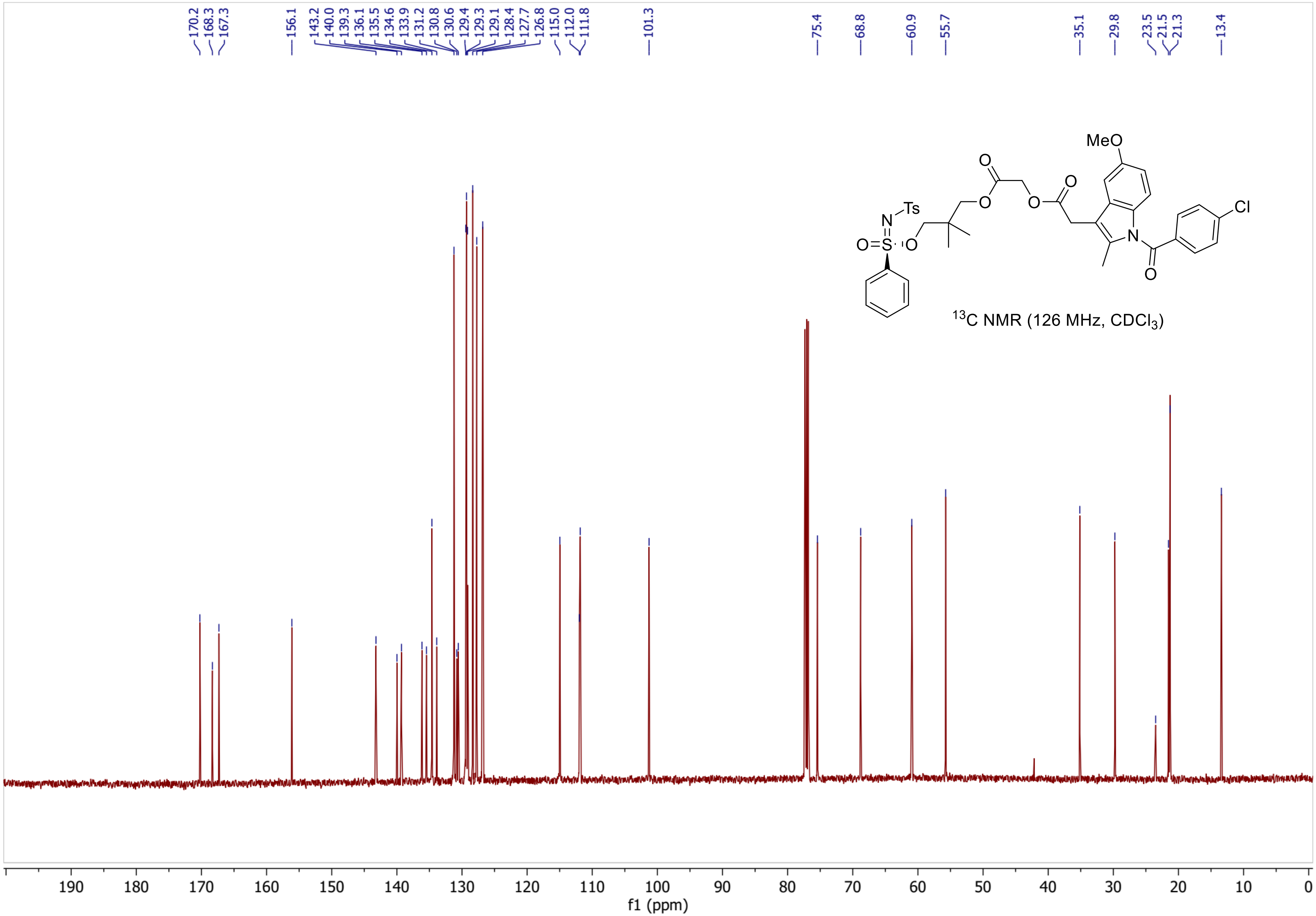

Go to table of contents

2,2-Dimethyl-3-(((*R*)-*N*-tosylphenylsulfonimidoyl)oxy)propyl 6-(3-((3r,5r,7r)-adamantan-1-yl)-4-methoxyphenyl)-2-naphthoate (14)

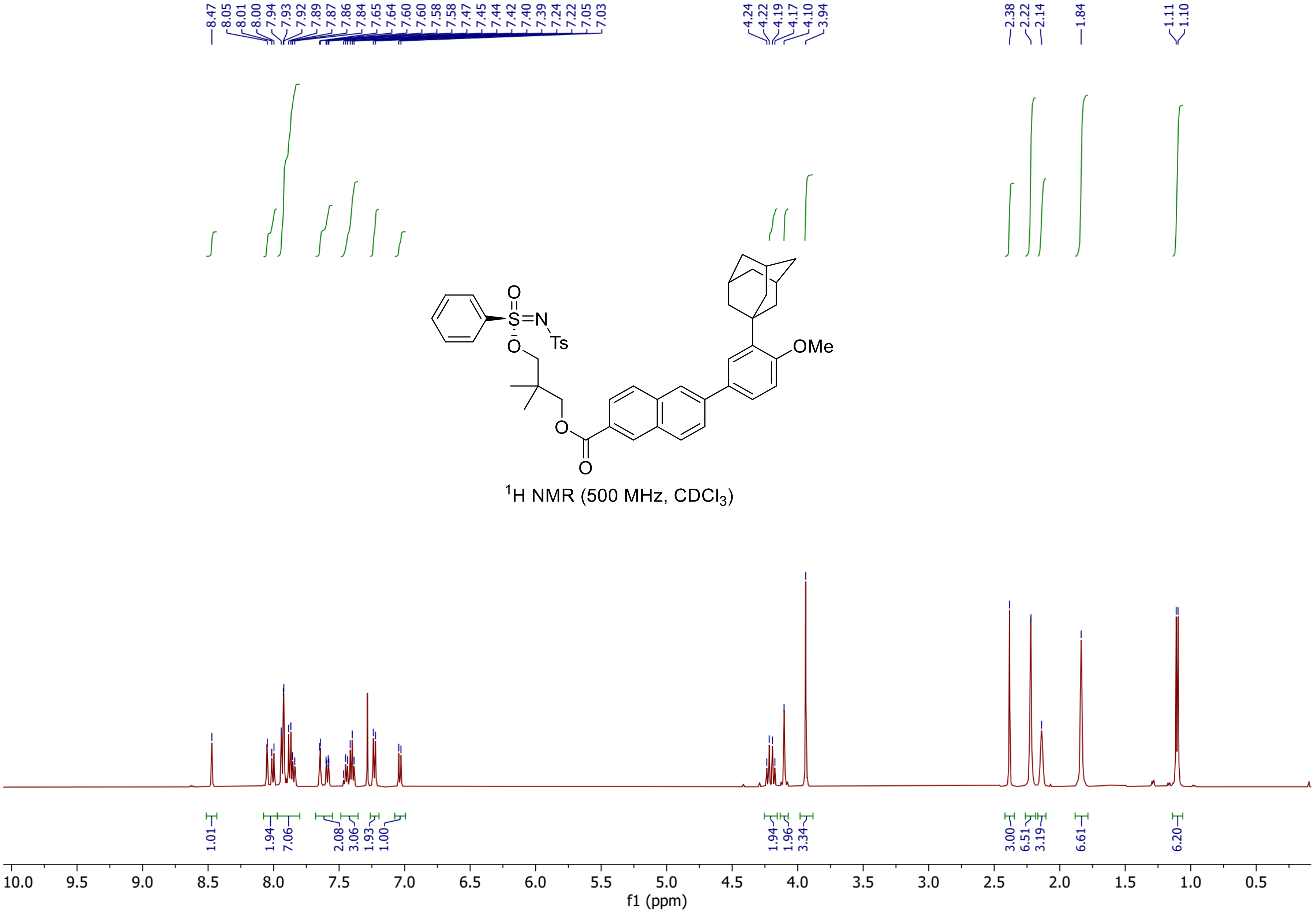

Go to table of contents

2,2-Dimethyl-3-(((*R*)-*N*-tosylphenylsulfonimidoyl)oxy)propyl 6-(3-((3r,5r,7r)-adamantan-1-yl)-4-methoxyphenyl)-2-naphthoate (14)

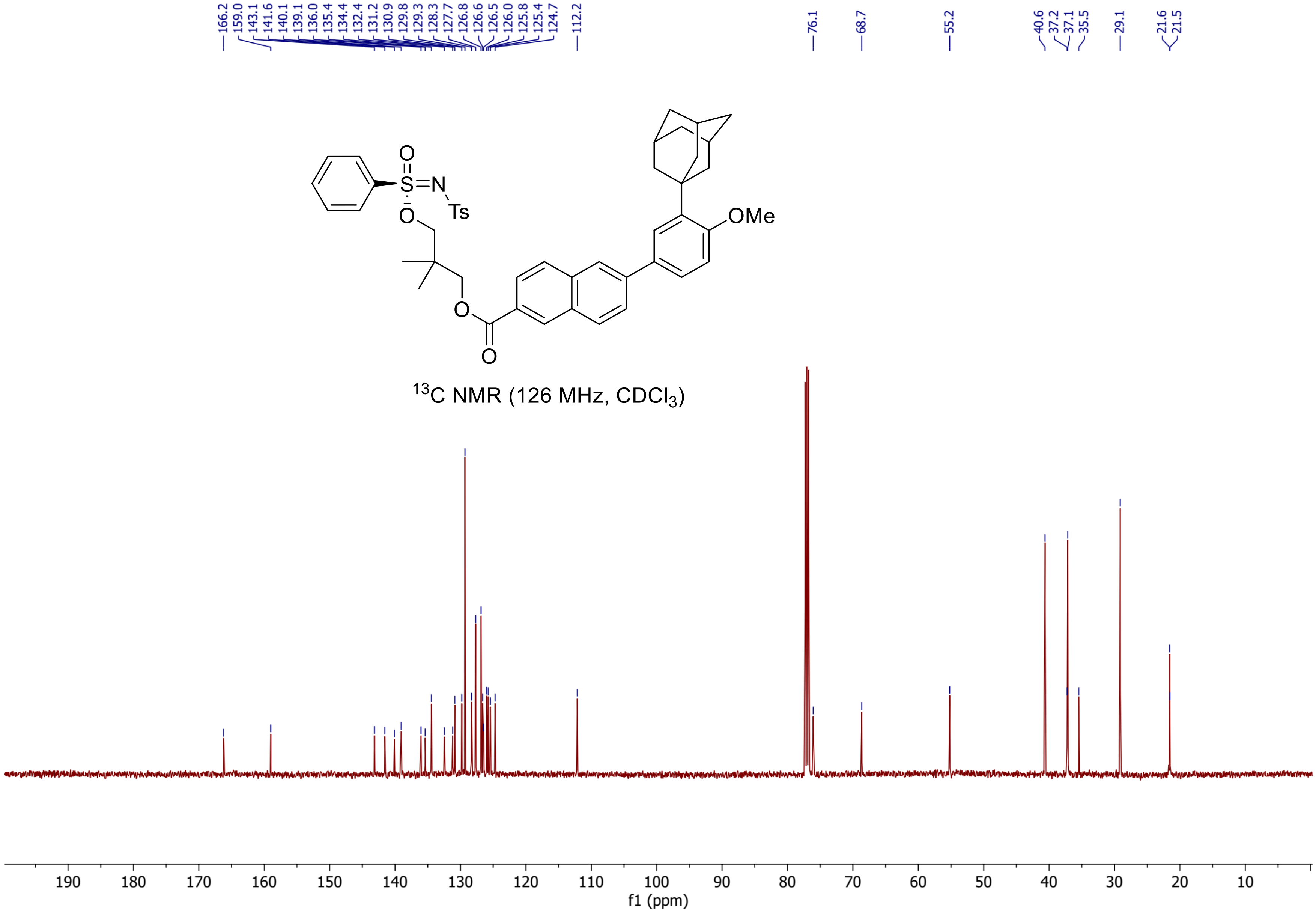

Go to table of contents

(*R*)-2,2-dimethyl-3-((*N*-tosylphenylsulfonimidoyl)oxy)propyl 2-(3-cyano-4-isobutoxyphenyl)-4-methylthiazole-5-carboxylate (15)

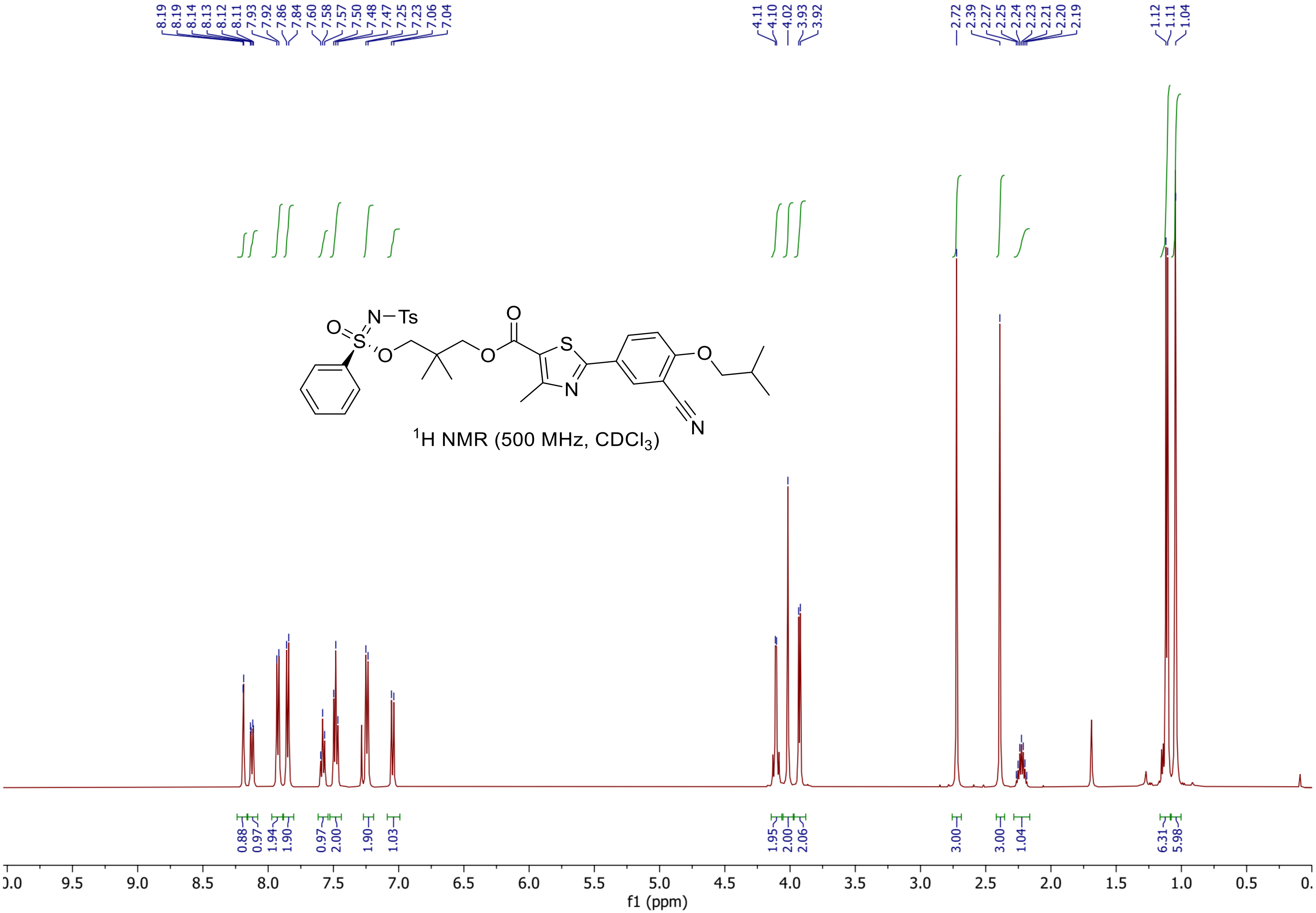

Go to table of contents

(*R*)-2,2-dimethyl-3-((*N*-tosylphenylsulfonimidoyl)oxy)propyl 2-(3-cyano-4-isobutoxyphenyl)-4-methylthiazole-5-carboxylate (15)

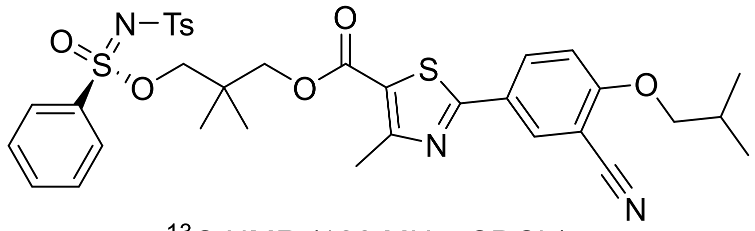

^{13}C NMR (126 MHz, $CDCl_3$)

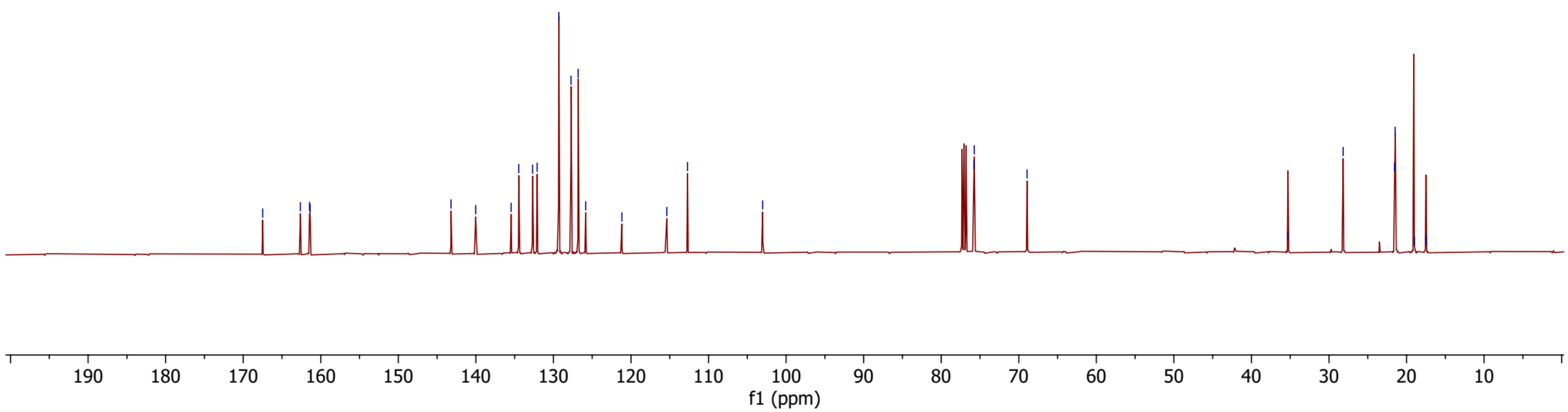

Go to table of contents

(*R*)-*N*-(Butyl(oxo)(phenyl)-λ⁶-sulfaneylidene)-4-methylbenzenesulfonamide (16)

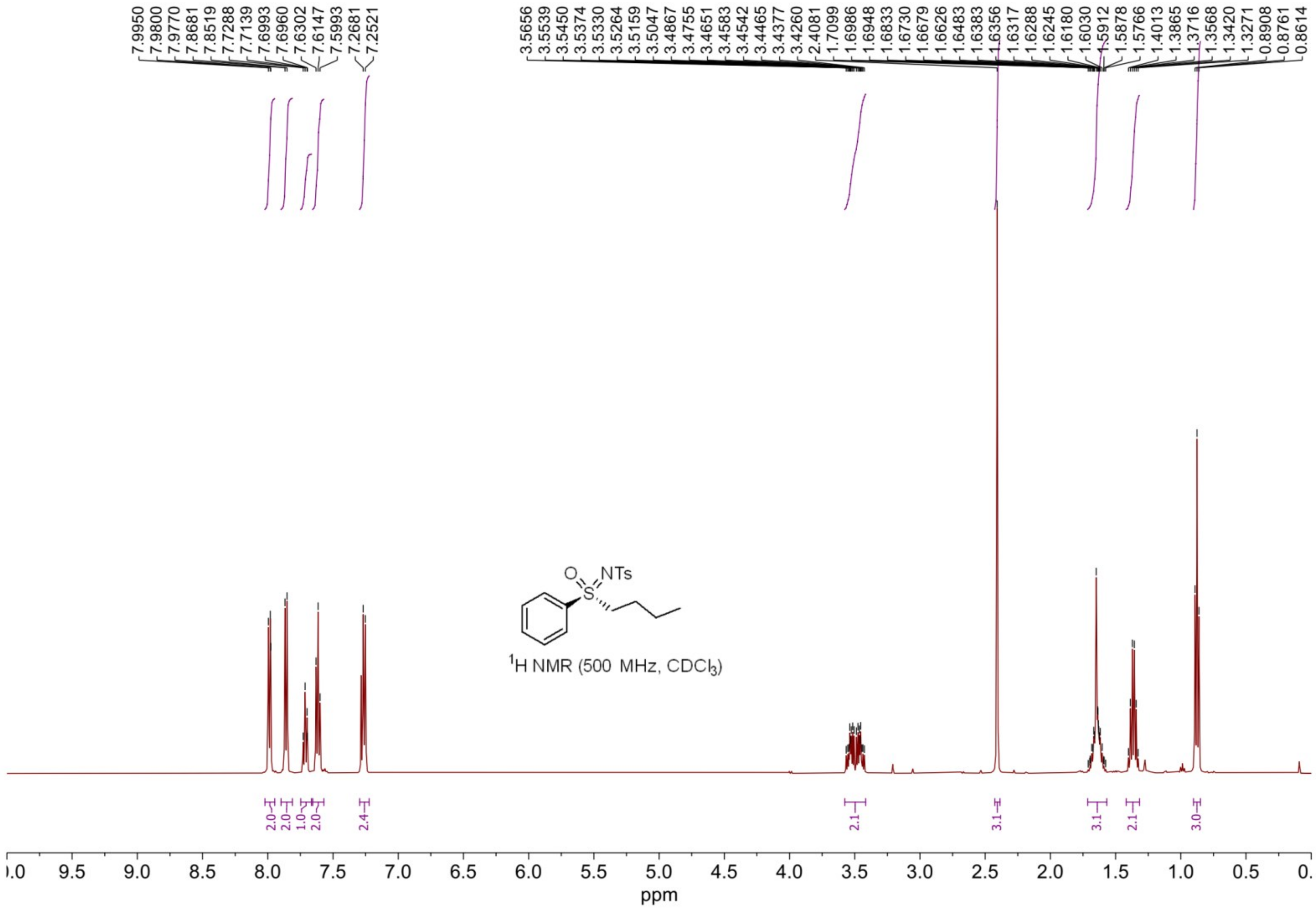

Go to table of contents

(*R*)-*N*-(Butyl(oxo)(phenyl)-λ⁶-sulfaneylidene)-4-methylbenzenesulfonamide (16)

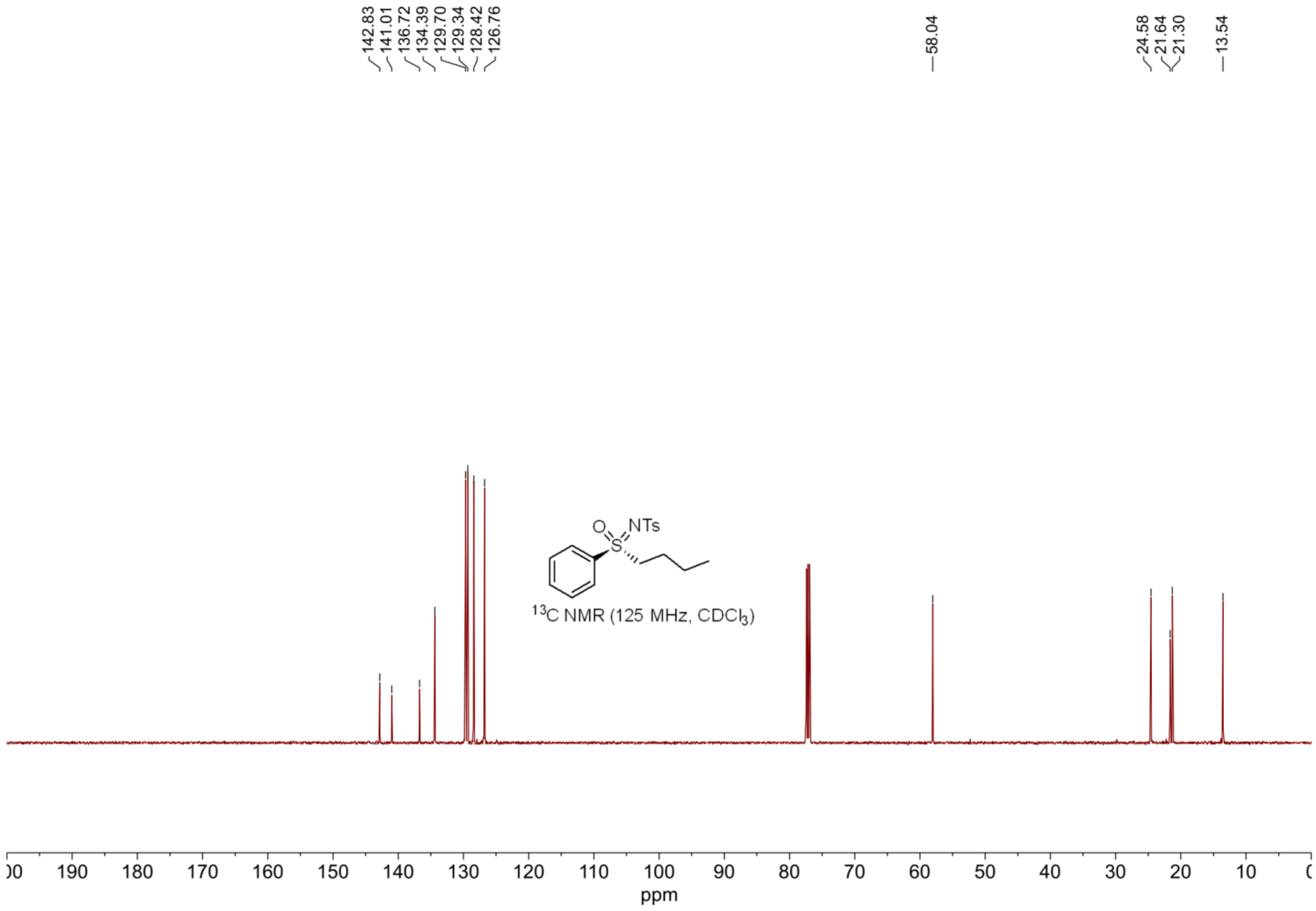

Go to table of contents

(*R*)-4-methyl-N-(methyl(oxo)(phenyl)- λ⁶-sulfaneylidene)benzenesulfonamide (17)

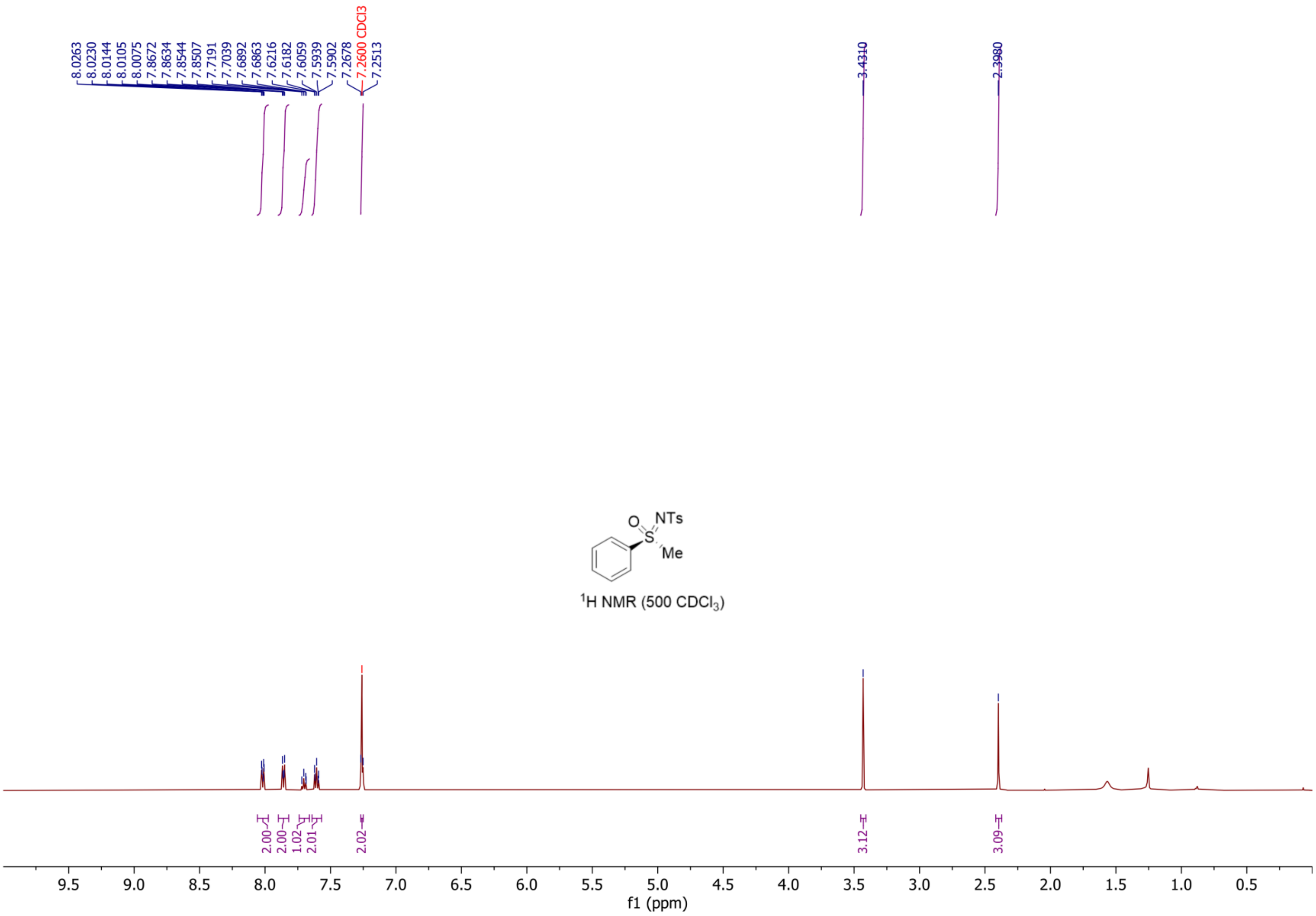

(*R*)-4-methyl-N-(methyl(oxo)(phenyl)- λ⁶-sulfaneylidene)benzenesulfonamide (17)

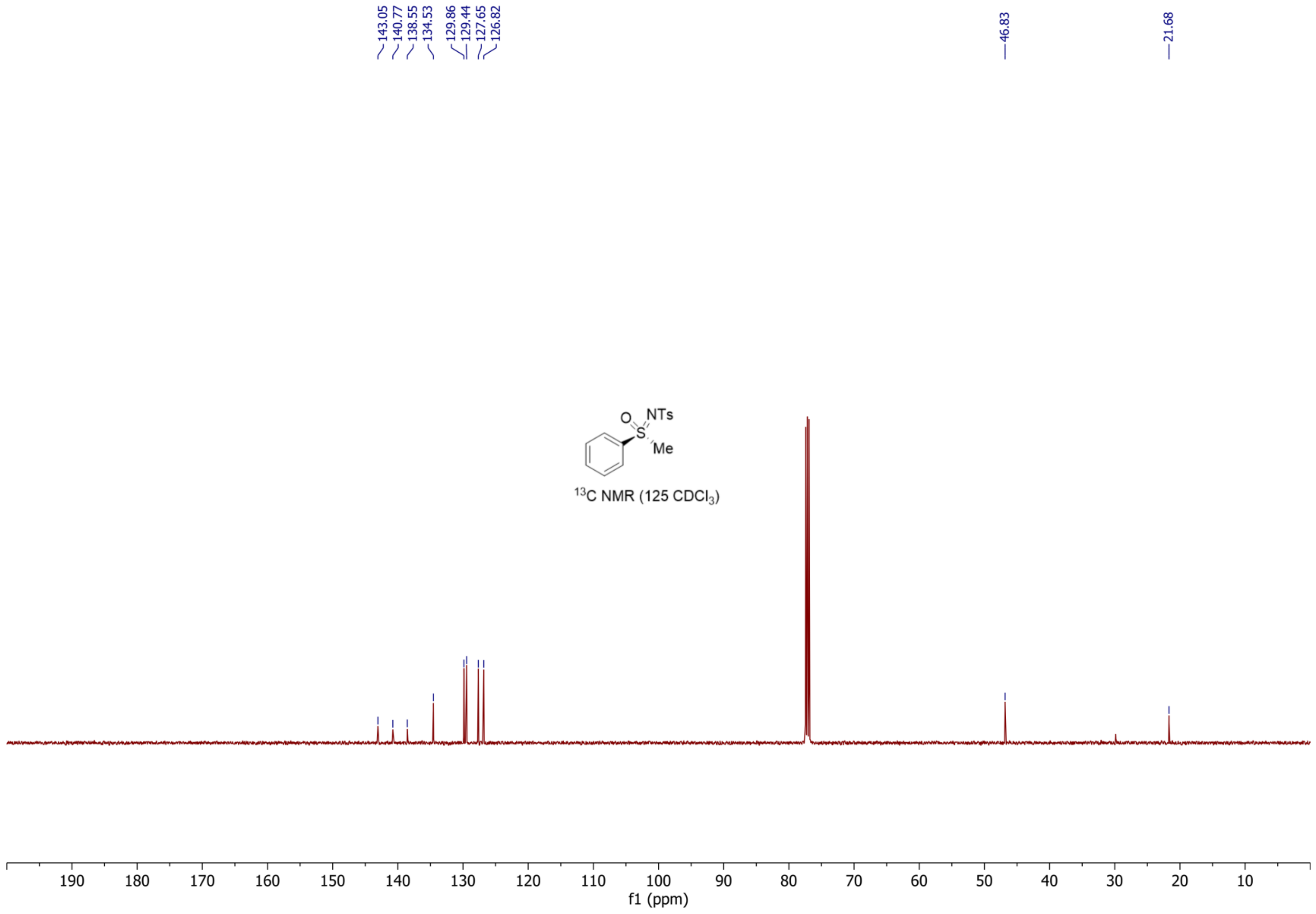

Go to table of contents

((*R*)-4-methyl-*N*-(oxo(phenethyl)(phenyl)- λ⁶-sulfaneylidene)benzenesulfonamide (18)

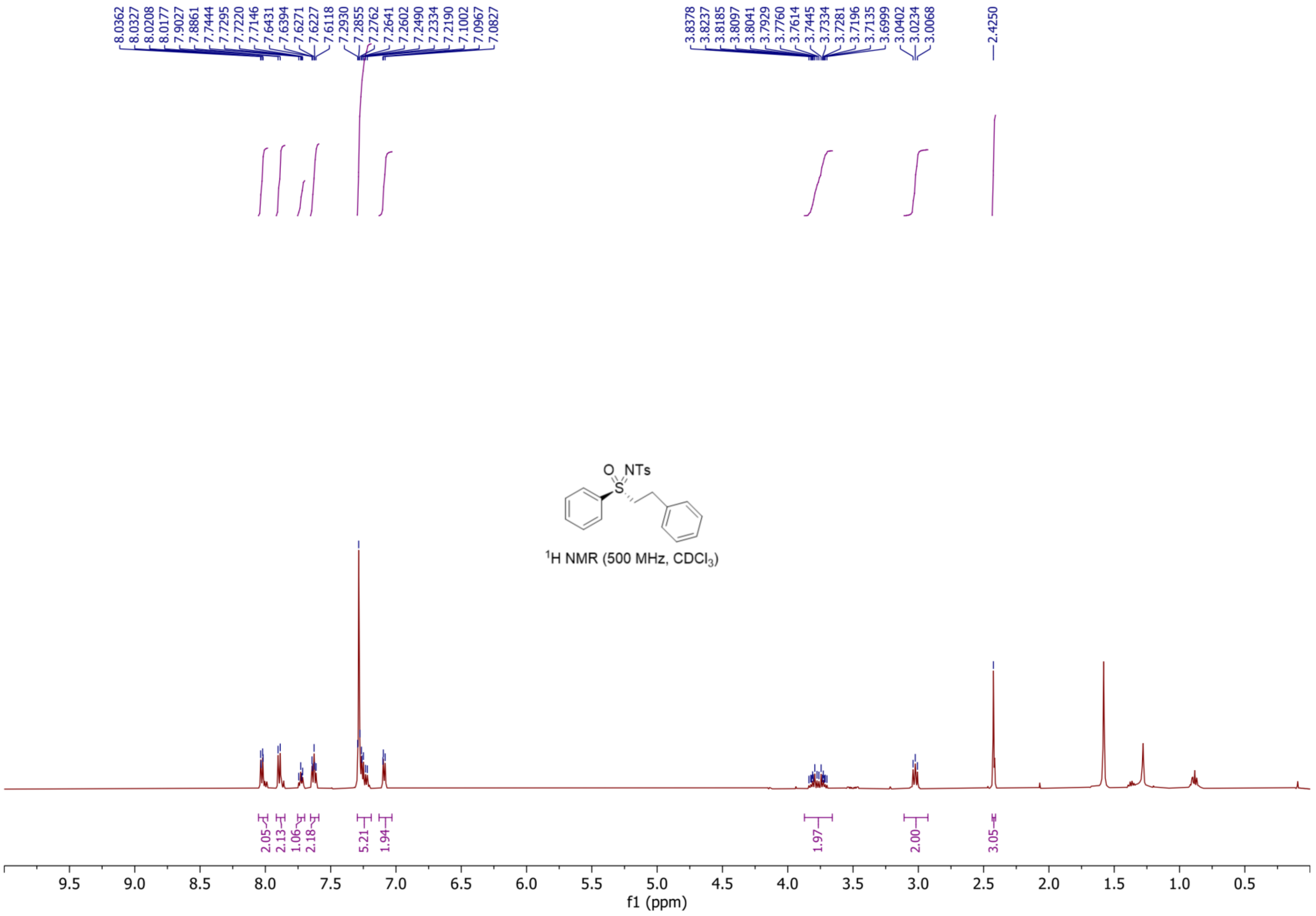

Go to table of contents

((*R*)-4-Methyl-*N*-(oxo(phenethyl)(phenyl)- λ⁶-sulfaneylidene)benzenesulfonamide (18)

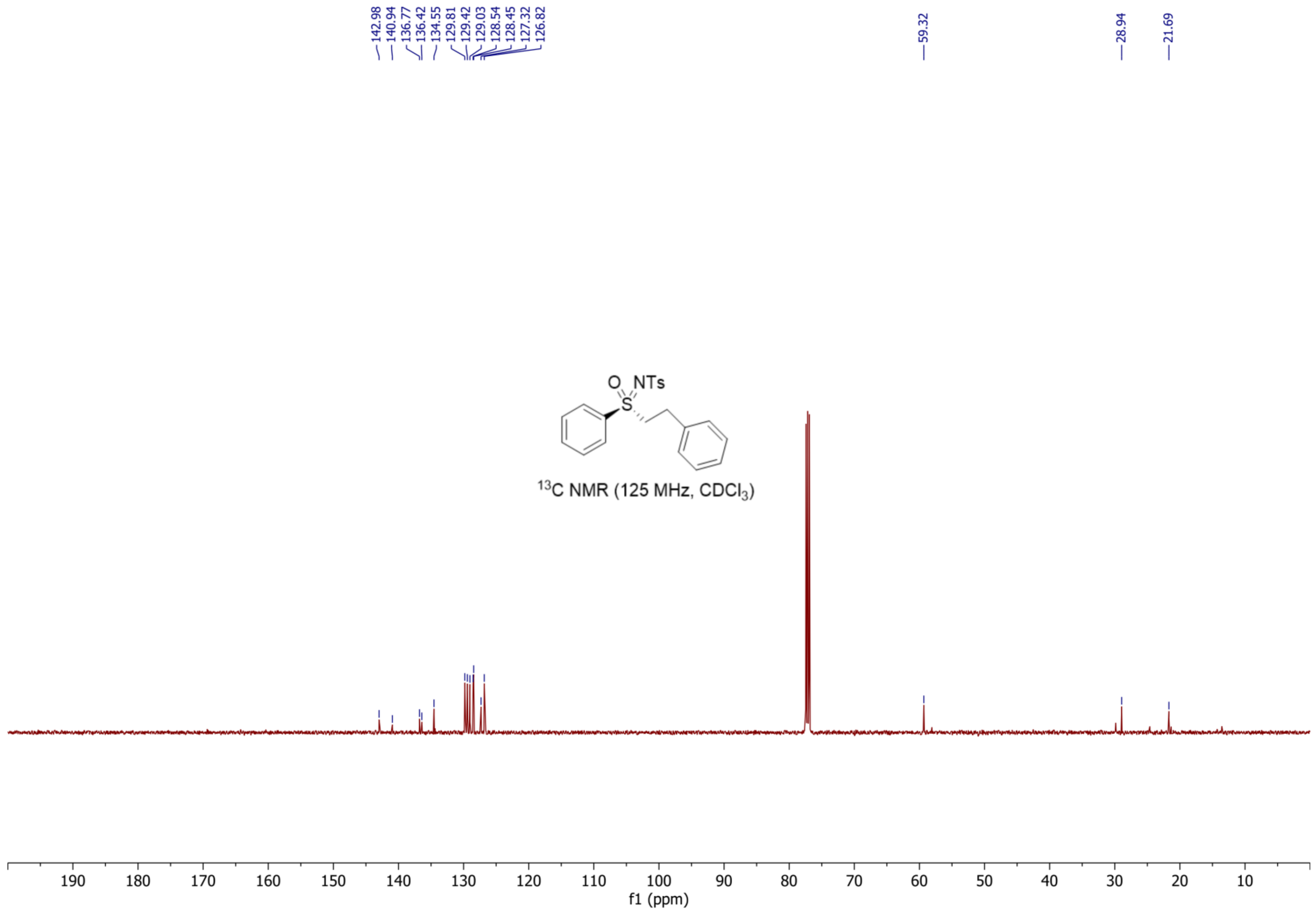

Go to table of contents

(*R*)-*N*-((4-Bromophenyl)(oxo)(phenyl)-λ⁶-sulfaneylidene)-4-methylbenzenesulfonamide (19)

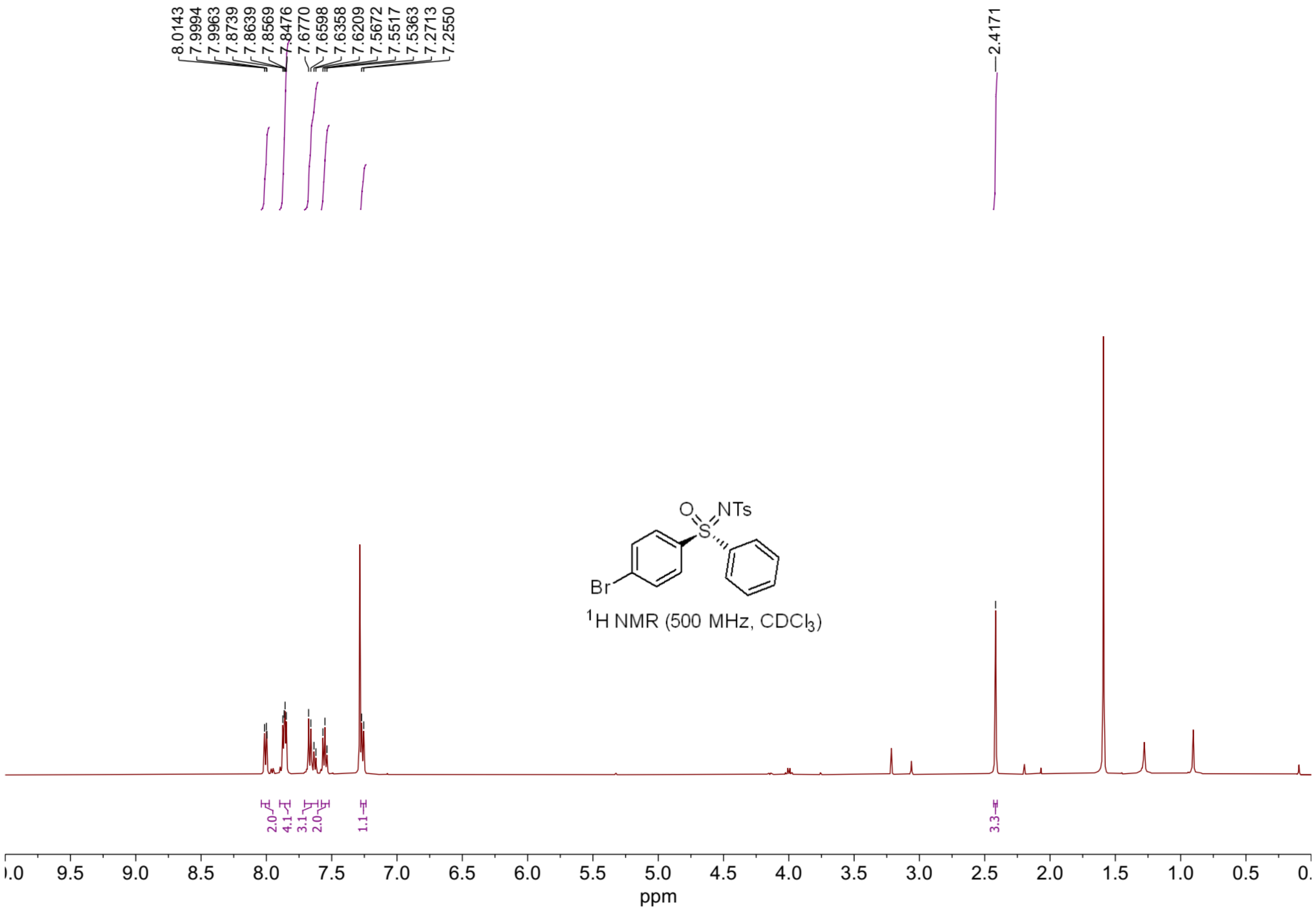

Go to table of contents

(*R*)-*N*-((4-Bromophenyl)(oxo)(phenyl)-λ^6-sulfaneylidene)-4-methylbenzenesulfonamide (19)

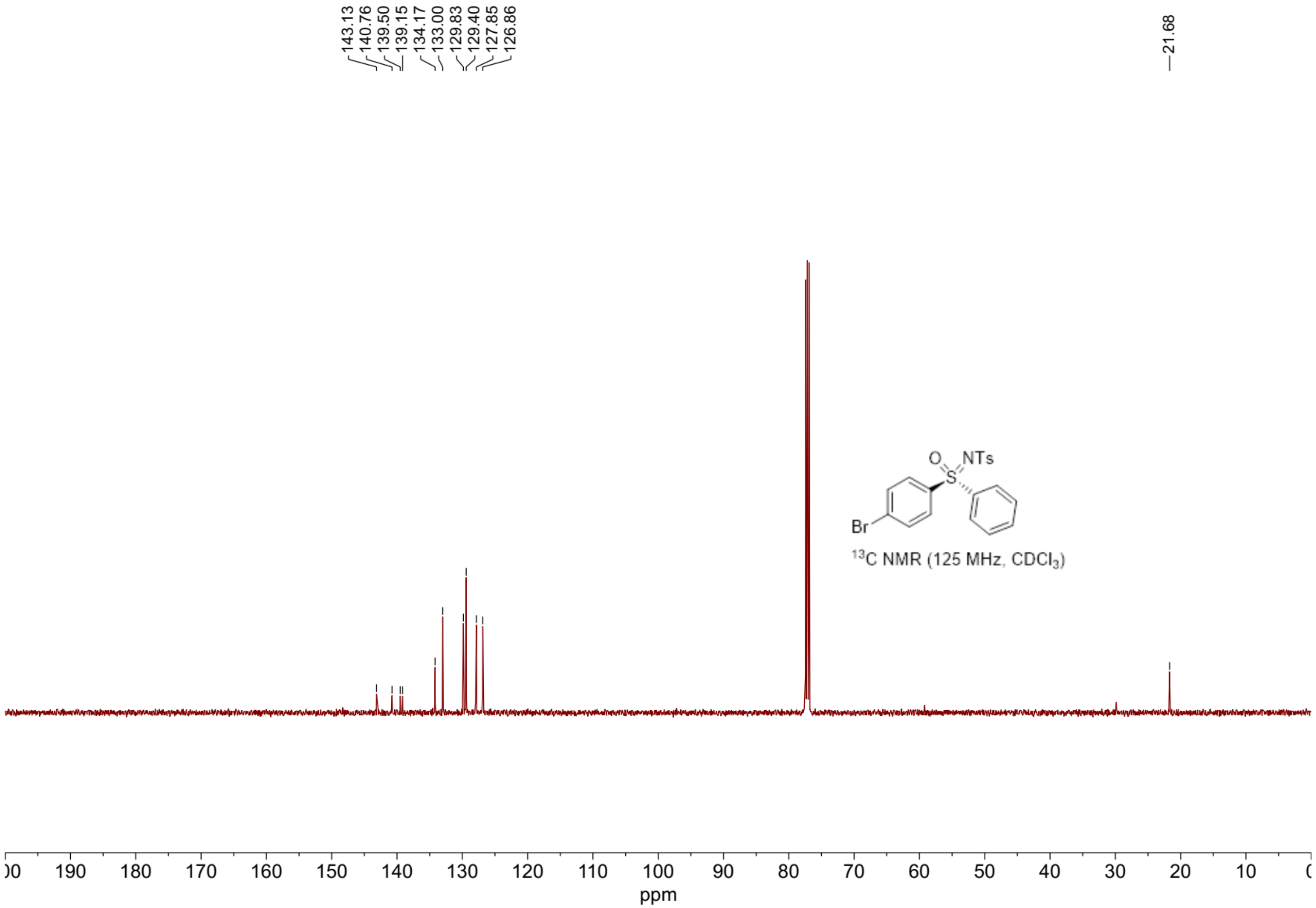

Go to table of contents

(*S*)-4-Methyl-*N*-(oxo(phenyl)(piperidin-1-yl)- λ⁶-sulfaneylidene)benzenesulfonamide (*ent*-7)

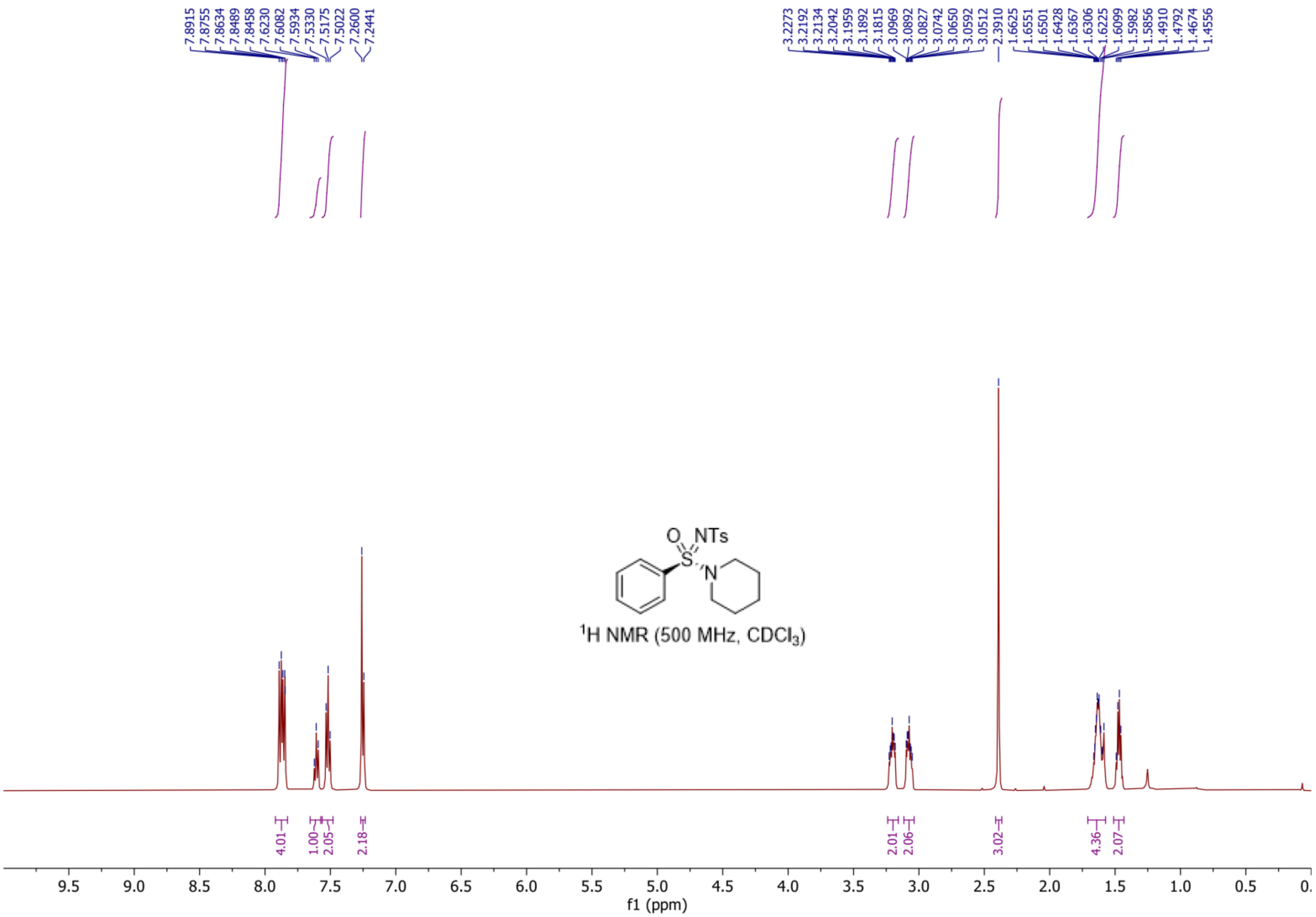

Go to table of contents

(*S*)-4-Methyl-*N*-(oxo(phenyl)(piperidin-1-yl)- λ⁶-sulfaneylidene)benzenesulfonamide (*ent*-7)

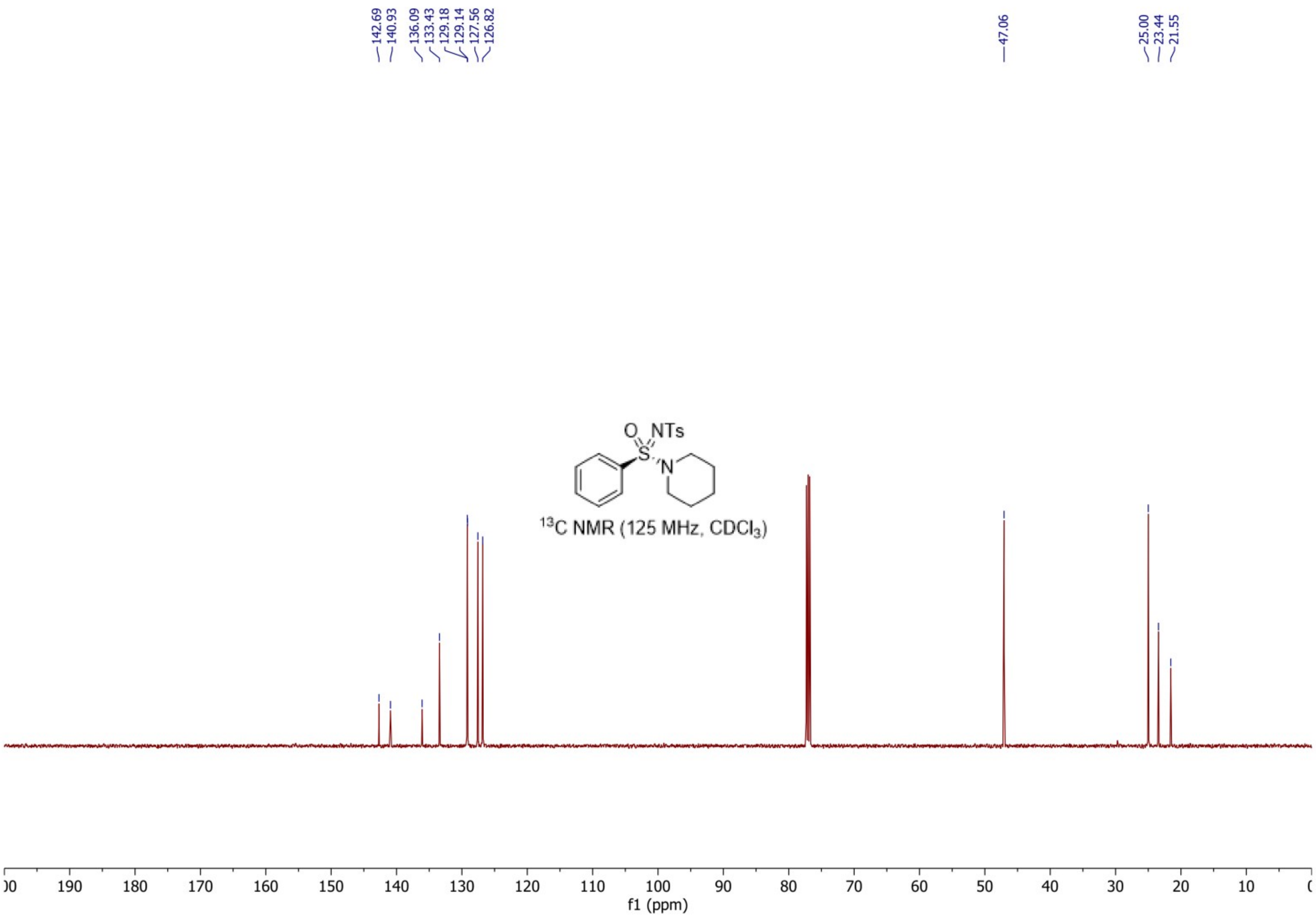

(*S*)-4-Methyl-*N*-(morpholino(oxo)(phenyl)- λ⁶-sulfaneylidene)benzenesulfonamide (20)

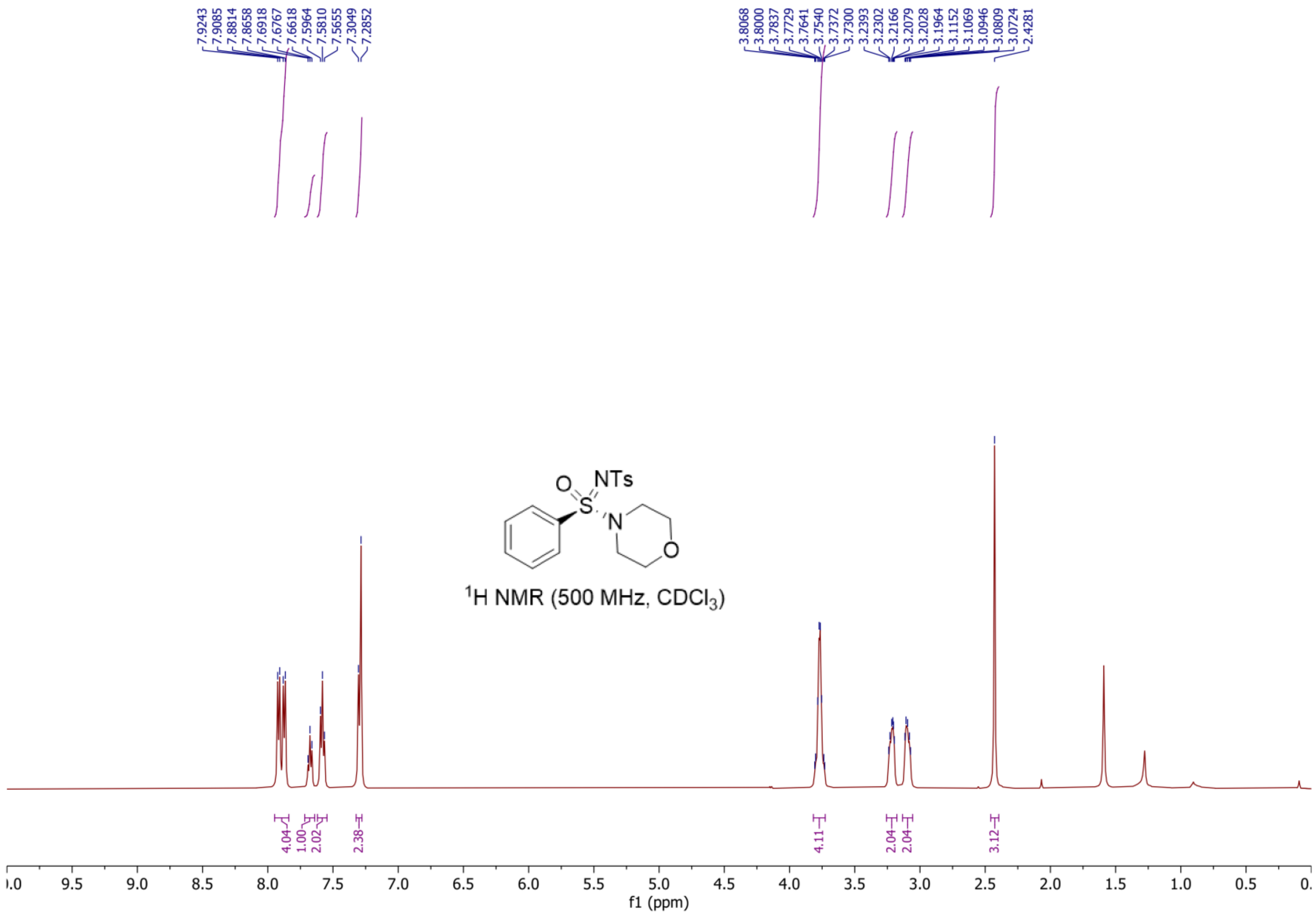

Go to table of contents

(*S*)-4-Methyl-*N*-(morpholino(oxo)(phenyl)- λ^6-sulfaneylidene)benzenesulfonamide (20)

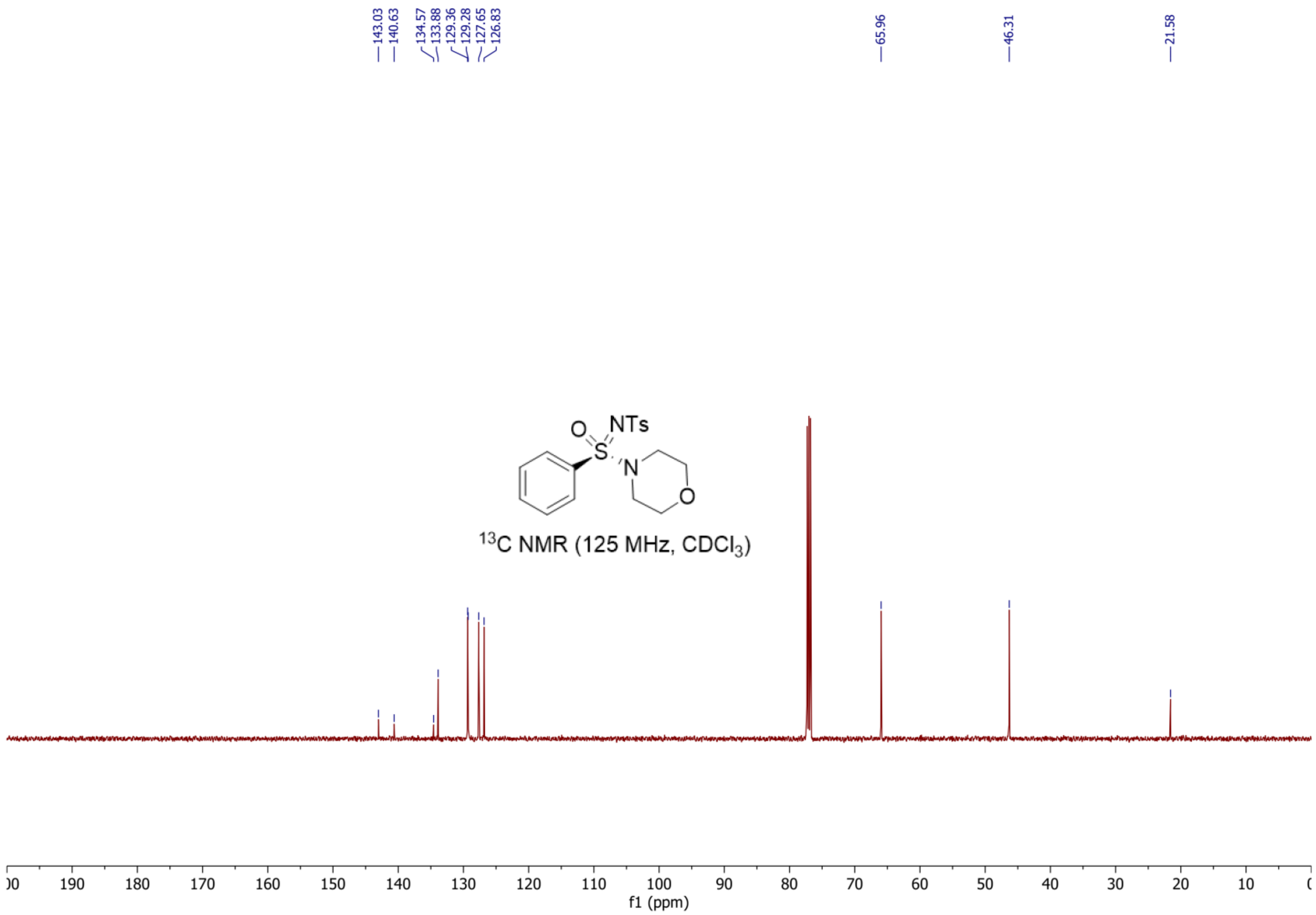

HPLC data

(S)-*N*-Tosylbenzenesulfonimidoyl chloride (*rac*-1a)

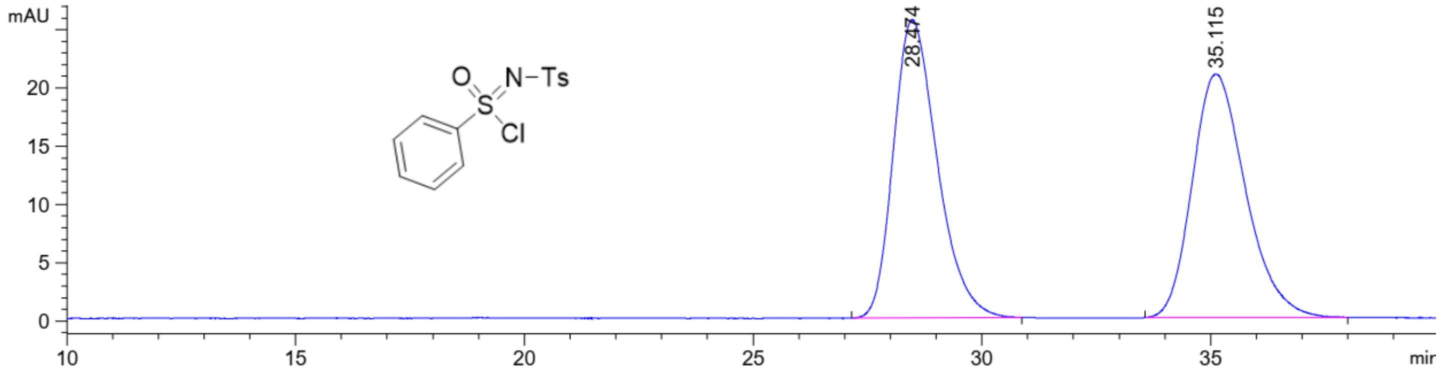

Peak #	RetTime [min]	Type	Width [min]	Area [mAU*s]	Height [mAU]	Area %
1	28.474	BB	1.0025	1707.29211	25.58694	50.2150
2	35.115	BB	1.1407	1692.67200	20.90346	49.7850

(S)-*N*-Tosylbenzenesulfonimidoyl chloride (1a)

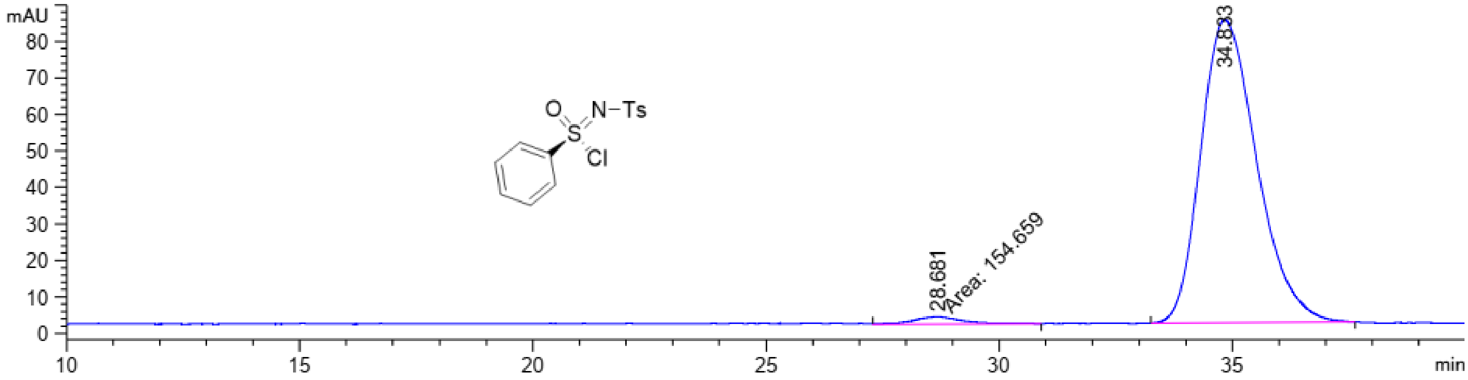

Peak #	RetTime [min]	Type	Width [min]	Area [mAU*s]	Height [mAU]	Area %
1	28.681	MM	1.2746	154.65944	2.02225	2.1787
2	34.833	BB	1.2305	6943.97070	82.96406	97.8213

3-Hydroxy-2,2-dimethylpropyl *(R)*-*N*-tosylbenzenesulfonimidate (*rac*-3a)

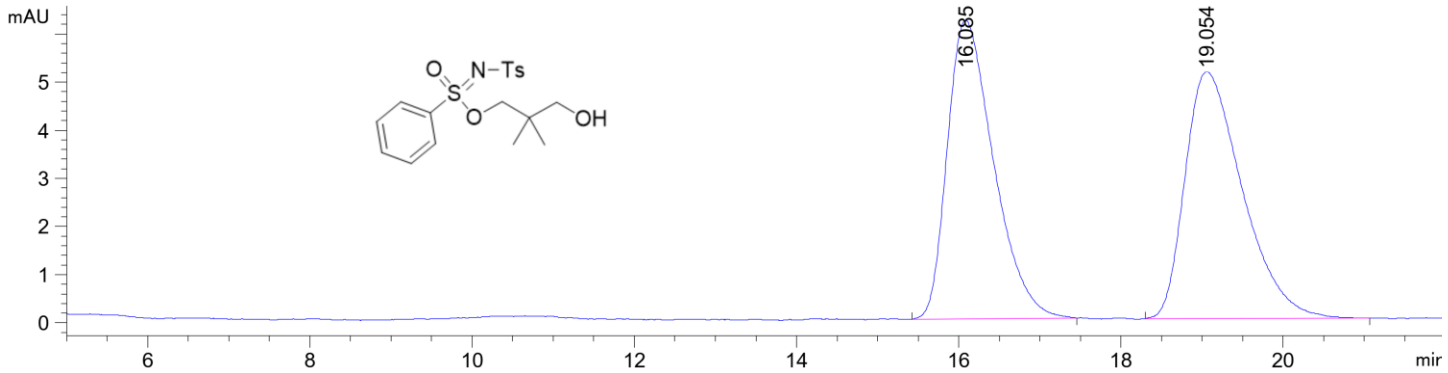

Peak #	RetTime [min]	Type	Width [min]	Area [mAU*s]	Height [mAU]	Area %
1	16.085	BB	0.5710	245.94707	6.19684	49.4585
2	19.054	BB	0.6722	251.33243	5.12421	50.5415

3-Hydroxy-2,2-dimethylpropyl *(R)*-*N*-tosylbenzenesulfonimidate (3a)

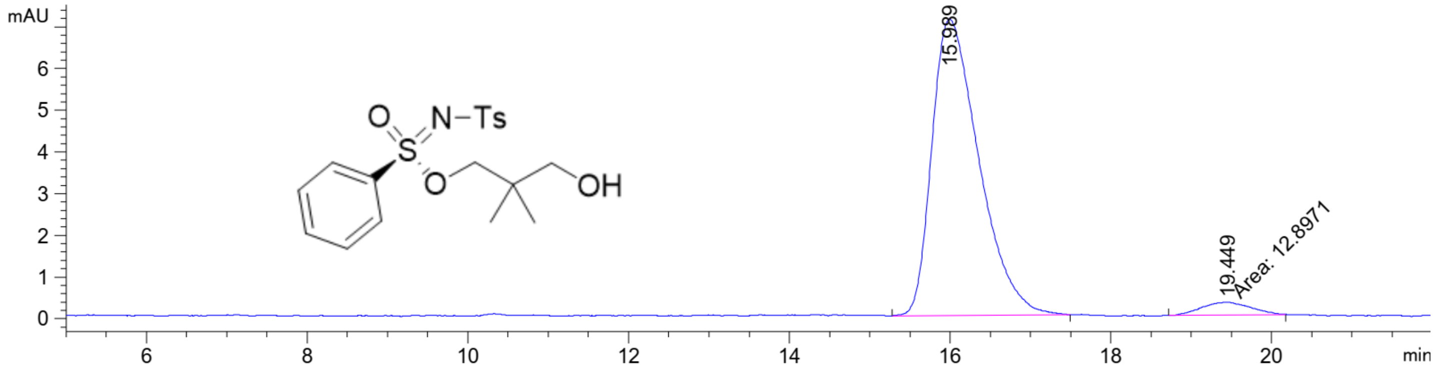

Peak #	RetTime [min]	Type	Width [min]	Area [mAU*s]	Height [mAU]	Area %
1	15.989	BB	0.6000	288.72620	7.10637	95.7241
2	19.449	MM	0.7077	12.89705	3.03712e-1	4.2759

(*S*)-2-Fluoro-*N*-tosylbenzenesulfonimidoyl chloride (*rac*-1b)

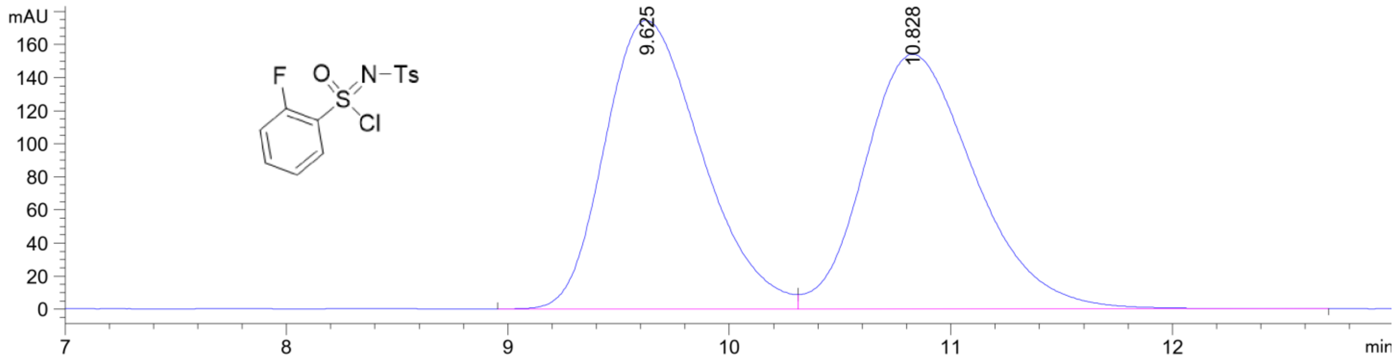

Peak #	RetTime [min]	Type	Width [min]	Area [mAU*s]	Height [mAU]	Area %
1	8.714	BV	0.3777	2615.77393	107.56317	48.7595
2	9.713	VB	0.4313	2748.86572	97.98135	51.2405

(*S*)-2-Fluoro-*N*-tosylbenzenesulfonimidoyl chloride (1b)

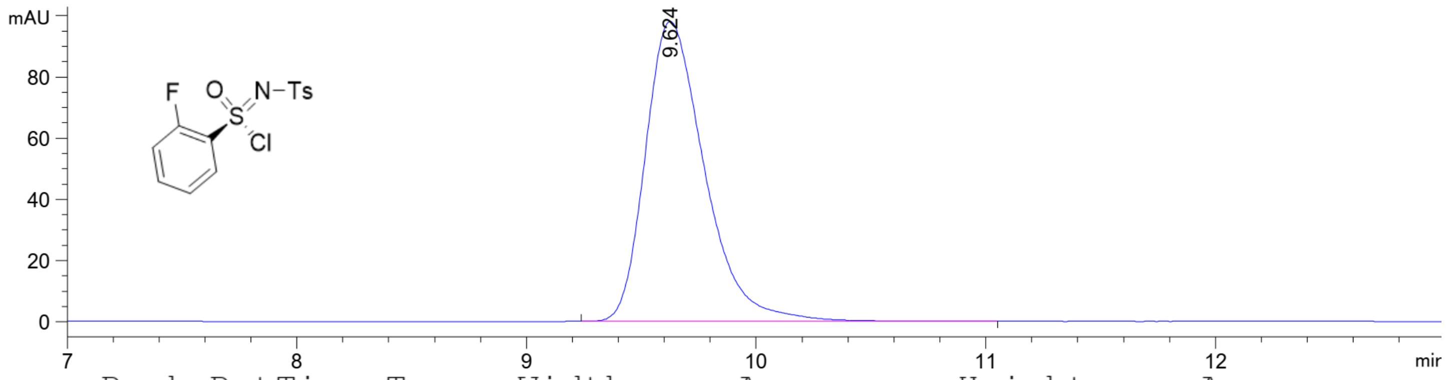

Peak #	RetTime [min]	Type	Width [min]	Area [mAU*s]	Height [mAU]	Area %
1	9.624	BB	0.2751	1756.78162	98.04026	100.0000

Go to table of contents

3-Hydroxy-2,2-dimethylpropyl *(R)*-2-fluoro-*N*-tosylbenzenesulfonimidate (*rac*-3b)

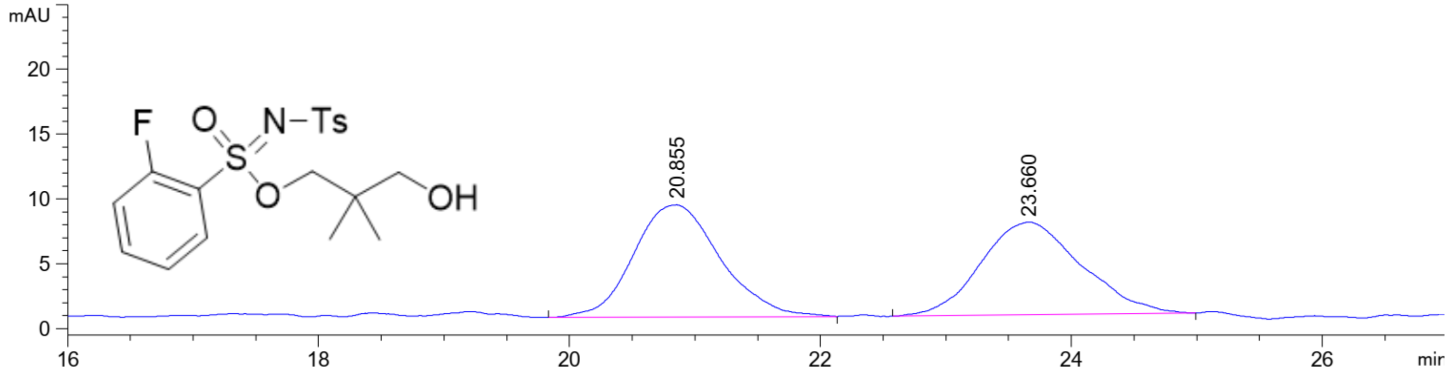

Peak #	RetTime [min]	Type	Width [min]	Area [mAU*s]	Height [mAU]	Area %
1	20.855	BB	0.6000	415.66040	8.65919	50.9975
2	23.660	BB	0.6882	399.39957	7.16671	49.0025

3-Hydroxy-2,2-dimethylpropyl *(R)*-2-fluoro-*N*-tosylbenzenesulfonimidate (3b)

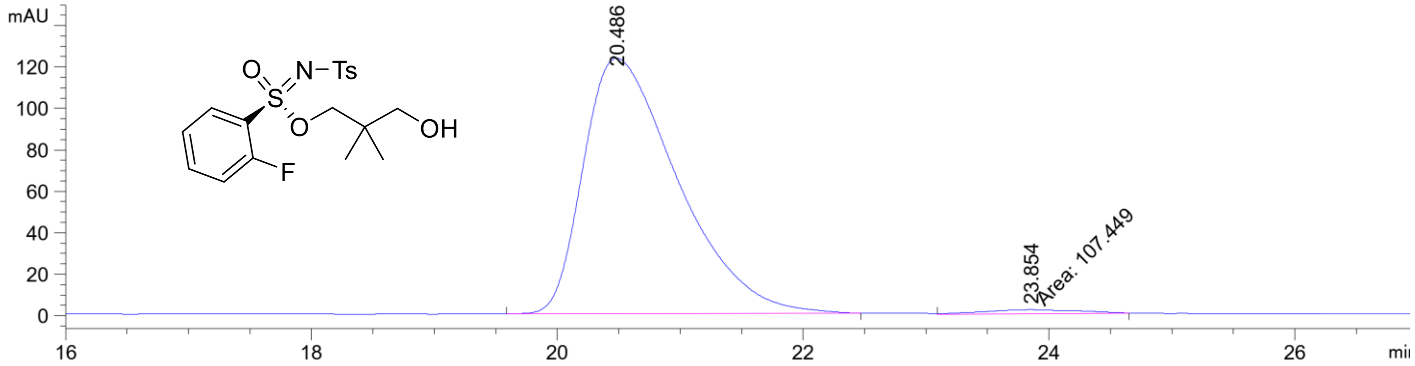

Peak #	RetTime [min]	Type	Width [min]	Area [mAU*s]	Height [mAU]	Area %
1	20.486	BB	0.8128	6579.10791	123.24340	98.3931
2	23.854	MM	0.9055	107.44920	1.97775	1.6069

Go to table of contents

(S)-4-Chloro-N-tosylbenzenesulfonimidoyl chloride (rac-1c)

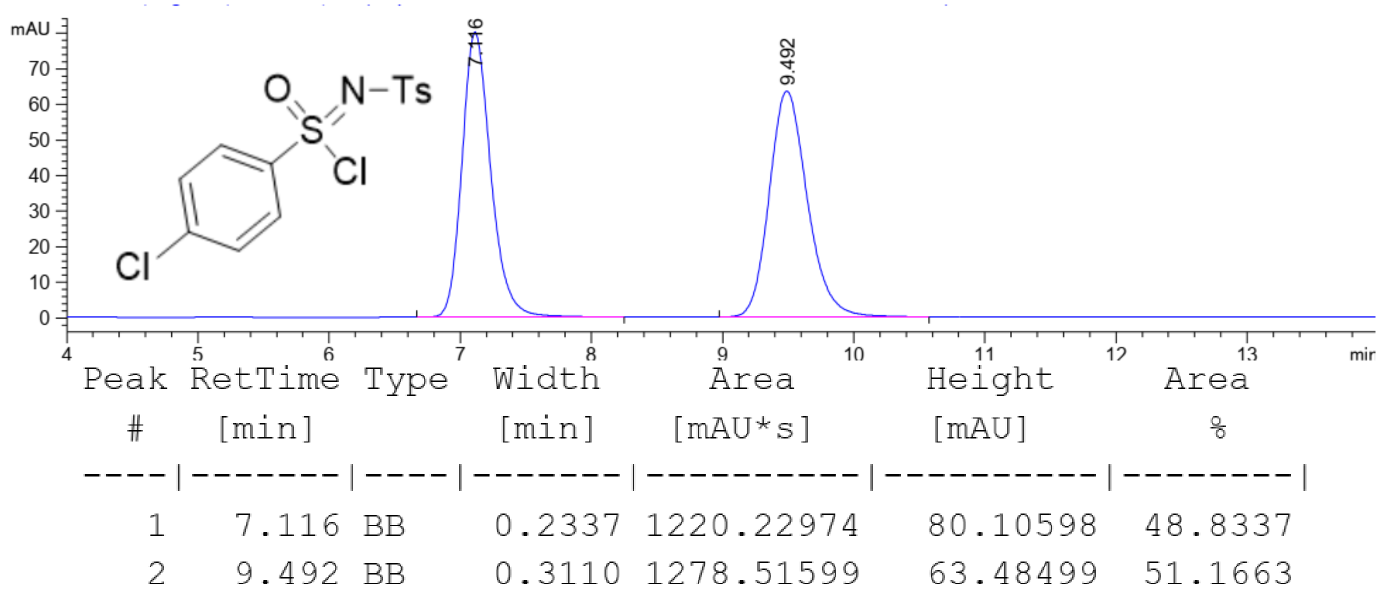

Peak #	RetTime [min]	Type	Width [min]	Area [mAU*s]	Height [mAU]	Area %
1	7.116	BB	0.2337	1220.22974	80.10598	48.8337
2	9.492	BB	0.3110	1278.51599	63.48499	51.1663

(S)-4-Chloro-N-tosylbenzenesulfonimidoyl chloride (1c)

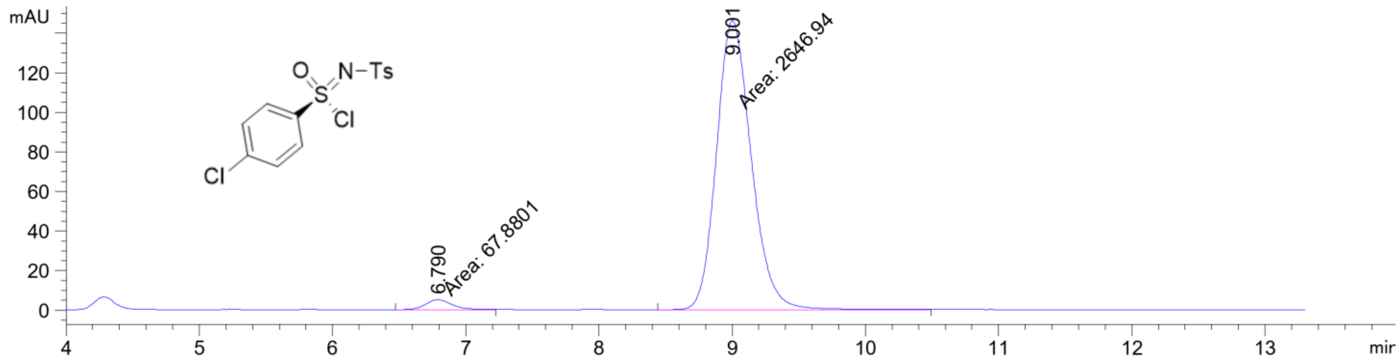

Peak #	RetTime [min]	Type	Width [min]	Area [mAU*s]	Height [mAU]	Area %
1	6.790	MF	0.2273	67.88013	4.97812	2.5004
2	9.001	MM	0.3017	2646.93555	146.23019	97.4996

3-Hydroxy-2,2-dimethylpropyl *(R)*-4-chloro-*N*-tosylbenzenesulfonimidate (*rac*-3c)

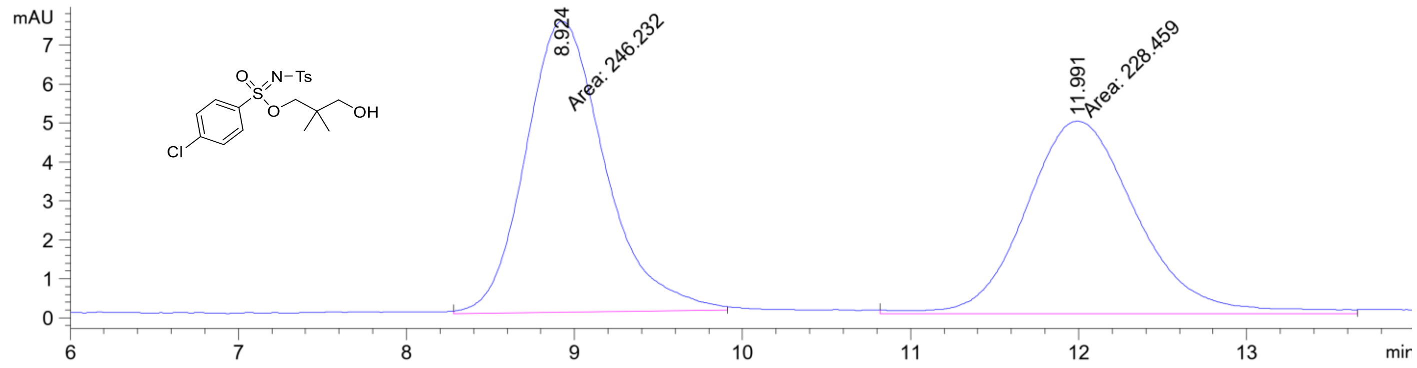

Peak #	RetTime [min]	Type	Width [min]	Area [mAU*s]	Height [mAU]	Area %
1	8.924	MM	0.5492	246.23216	7.47209	51.8721
2	11.991	MM	0.7706	228.45923	4.94118	48.1279

3-Hydroxy-2,2-dimethylpropyl *(R)*-4-chloro-*N*-tosylbenzenesulfonimidate (3c)

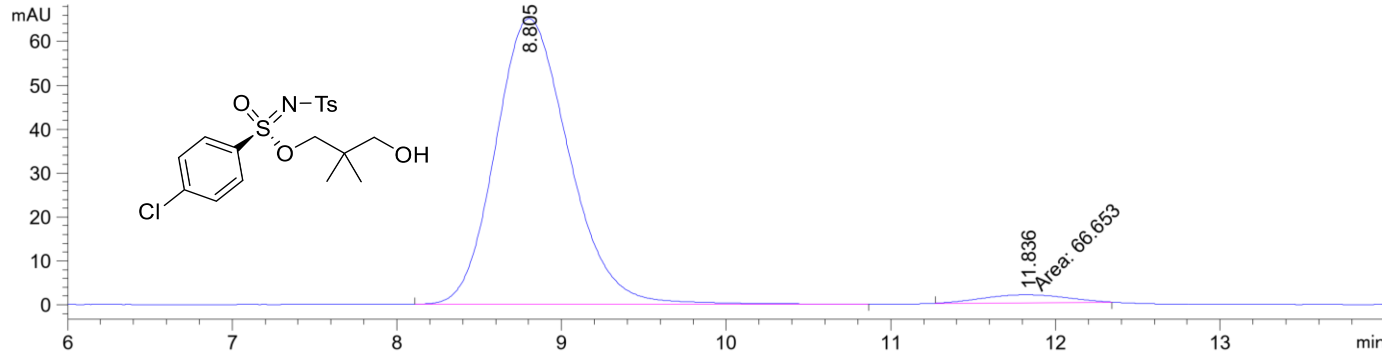

Peak #	RetTime [min]	Type	Width [min]	Area [mAU*s]	Height [mAU]	Area %
1	8.805	BB	0.4769	2012.98853	65.07691	95.5443
2	11.836	MM	0.7102	93.87476	2.20304	4.4557

(*S*)-4-Bromo-*N*-tosylbenzenesulfonimidoyl chloride (*rac*-1d)

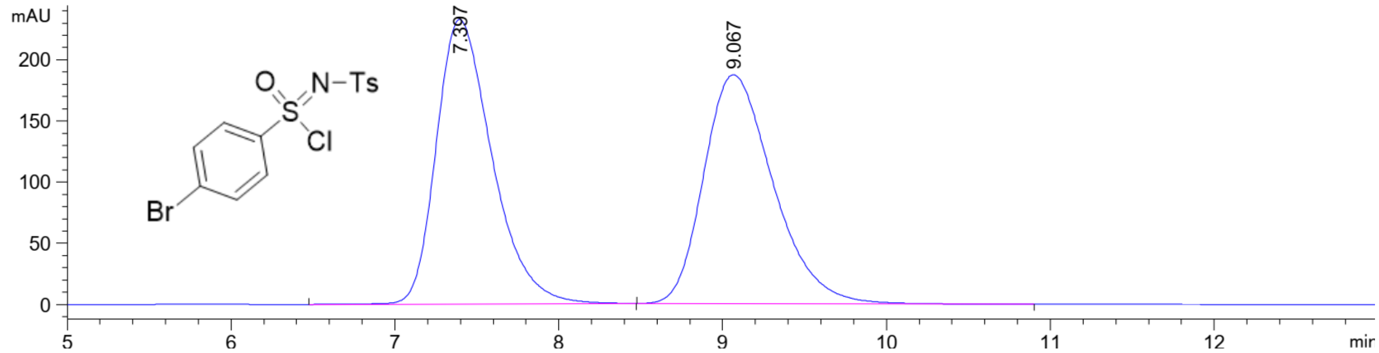

Peak #	RetTime [min]	Type	Width [min]	Area [mAU*s]	Height [mAU]	Area %
1	7.397	BB	0.3634	5492.26367	232.66353	49.8819
2	9.067	BB	0.4540	5518.27588	187.12410	50.1181

(*S*)-4-Bromo-*N*-tosylbenzenesulfonimidoyl chloride (1d)

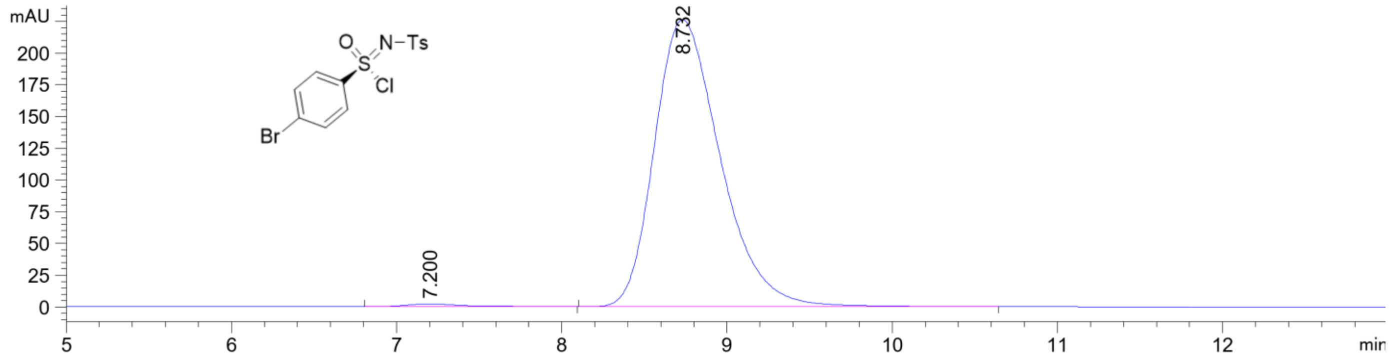

Peak #	RetTime [min]	Type	Width [min]	Area [mAU*s]	Height [mAU]	Area %
1	7.200	BB	0.3552	54.28333	2.18976	0.8578
2	8.732	BB	0.4255	6273.67578	226.22217	99.1422

3-Hydroxy-2,2-dimethylpropyl *(R)*-4-bromo-*N*-tosylbenzenesulfonimidate (*rac*-3d)

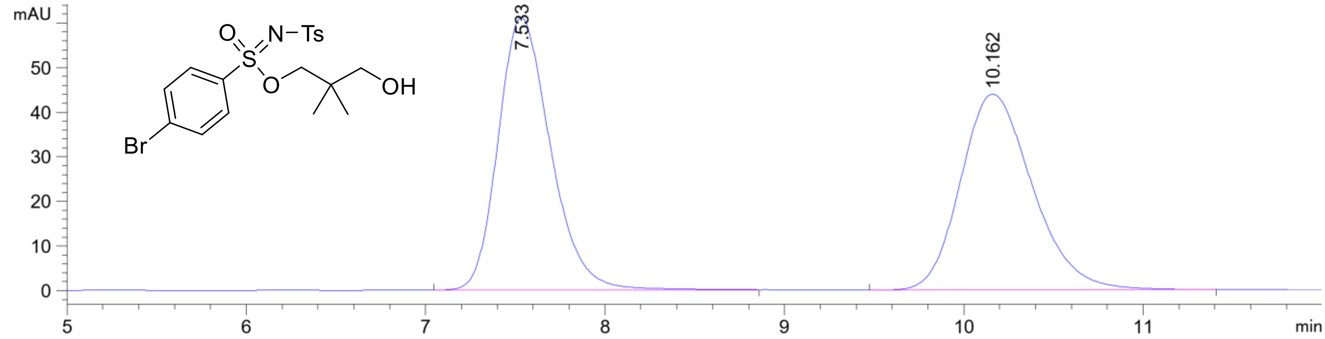

Peak #	RetTime [min]	Type	Width [min]	Area [mAU*s]	Height [mAU]	Area %
1	7.533	BB	0.3125	1237.97961	61.05941	50.2310
2	10.162	BB	0.4317	1226.59460	43.94054	49.7690

3-Hydroxy-2,2-dimethylpropyl *(R)*-4-bromo-*N*-tosylbenzenesulfonimidate (3d)

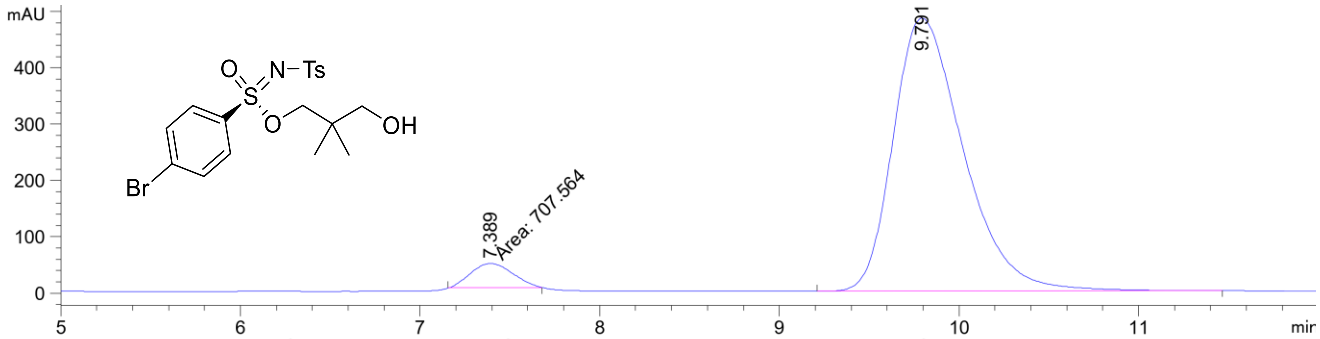

Peak #	RetTime [min]	Type	Width [min]	Area [mAU*s]	Height [mAU]	Area %
1	7.389	MM	0.2726	707.56366	43.26100	5.0945
2	9.791	BB	0.4167	1.31811e4	485.53607	94.9055

(*S*)-4-Iodo-*N*-tosylbenzenesulfonimidoyl chloride (*rac*-1e)

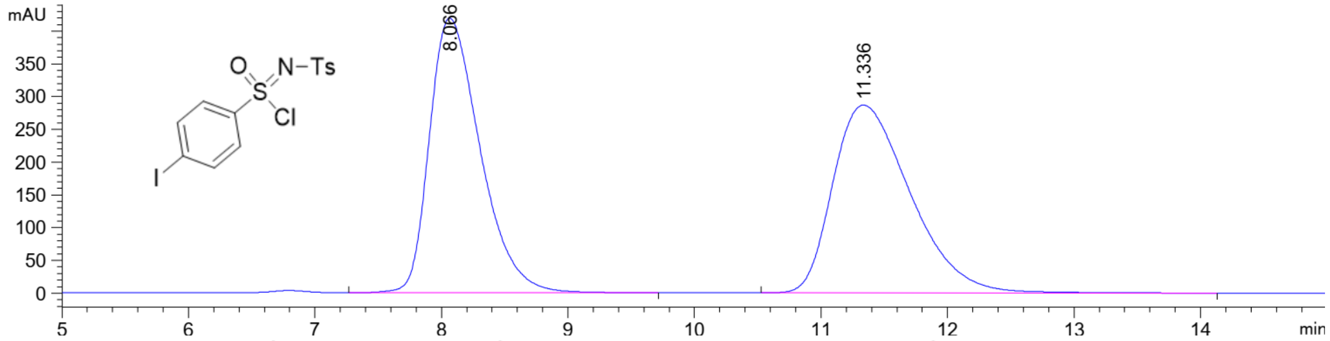

Peak #	RetTime [min]	Type	Width [min]	Area [mAU*s]	Height [mAU]	Area %
1	8.066	BB	0.4315	1.16998e4	419.34137	49.0171
2	11.336	BB	0.6634	1.21690e4	286.57755	50.9829

(*S*)-4-Iodo-*N*-tosylbenzenesulfonimidoyl chloride (1e)

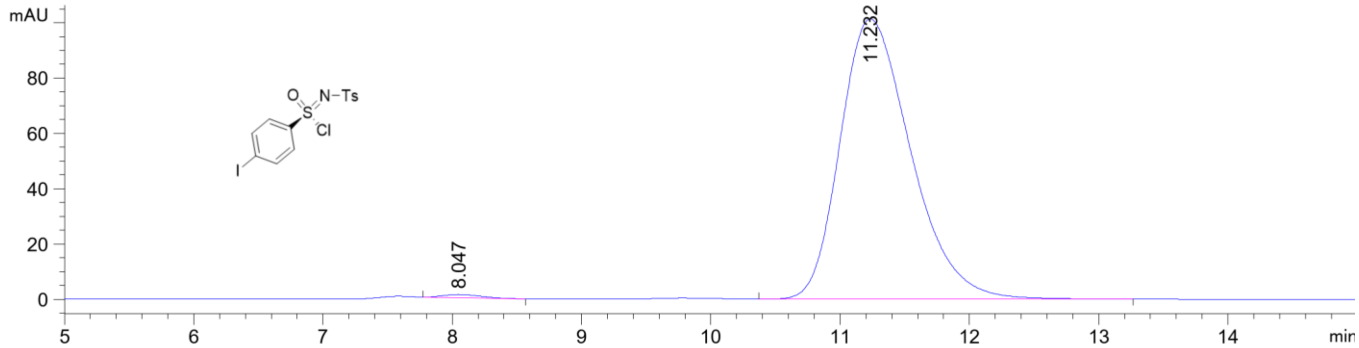

Peak #	RetTime [min]	Type	Width [min]	Area [mAU*s]	Height [mAU]	Area %
1	8.047	BB	0.2836	23.24009	1.16057	0.6003
2	11.232	BB	0.5870	3848.05981	101.33597	99.3997

3-Hydroxy-2,2-dimethylpropyl (*R*)-4-iodo-*N*-tosylbenzenesulfonimidate (*rac*-3e)

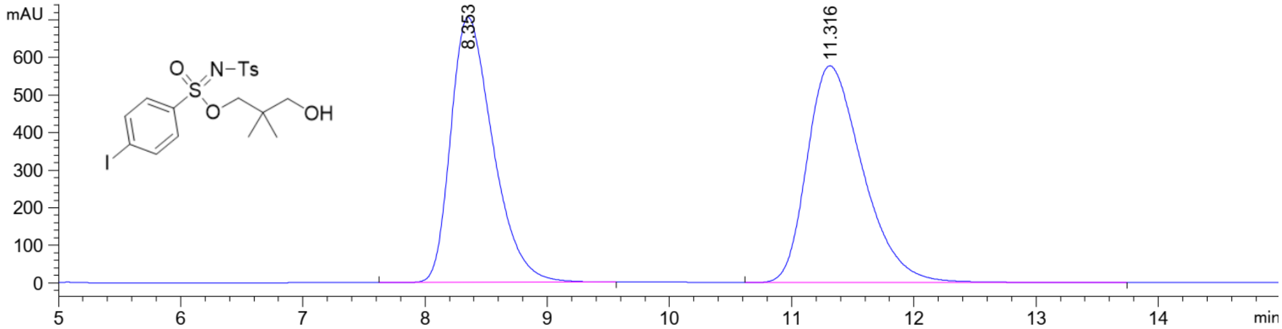

Peak #	RetTime [min]	Type	Width [min]	Area [mAU*s]	Height [mAU]	Area %
1	8.353	BB	0.3698	1.69324e4	706.01019	48.0059
2	11.316	BB	0.4867	1.83392e4	577.14209	51.9941

3-Hydroxy-2,2-dimethylpropyl (*R*)-4-iodo-*N*-tosylbenzenesulfonimidate (3e)

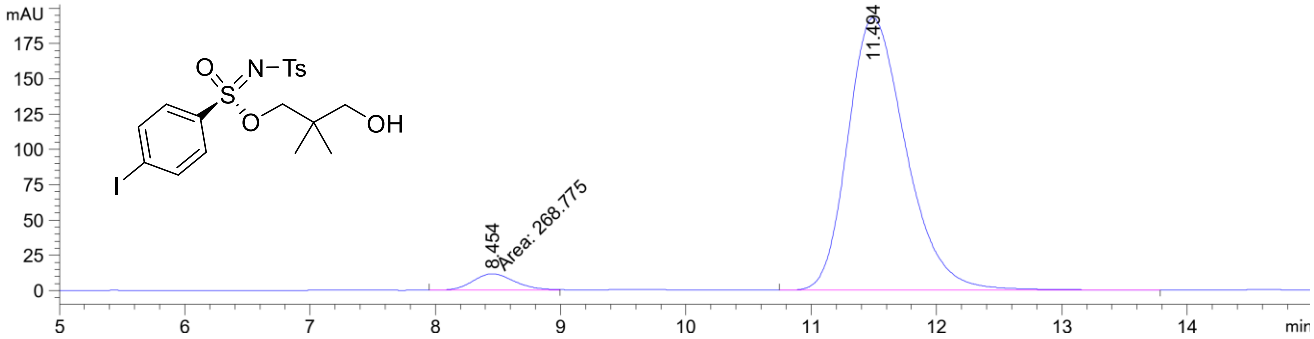

Peak #	RetTime [min]	Type	Width [min]	Area [mAU*s]	Height [mAU]	Area %
1	8.454	BB	0.3649	270.25540	11.55522	4.1625
2	11.494	BB	0.4961	6222.39893	193.02350	95.8375

Go to table of contents

(S)-4-Nitro-*N*-tosylbenzenesulfonimidoyl chloride (*rac*-1f)

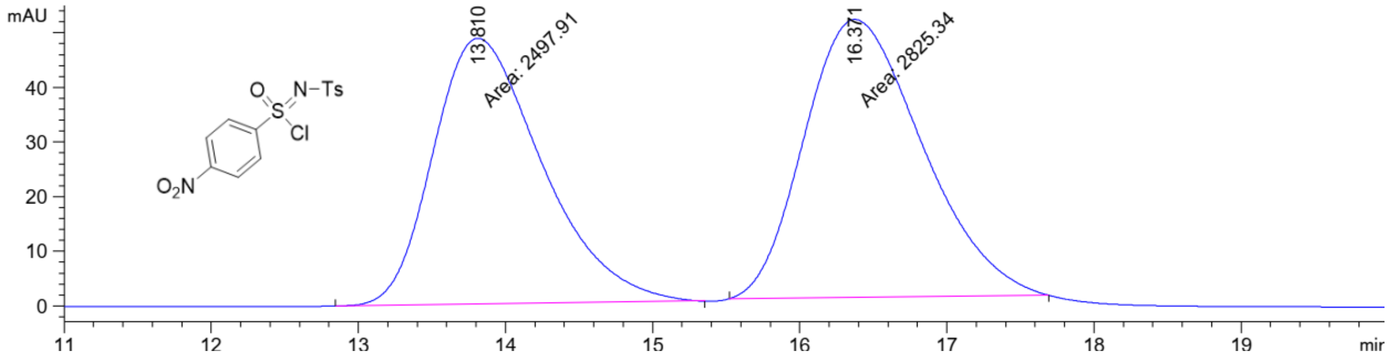

Peak #	RetTime [min]	Type	Width [min]	Area [mAU*s]	Height [mAU]	Area %
1	13.808	BB	0.7742	2297.22241	44.50562	48.4476
2	16.370	MM	0.9005	2444.44458	45.24480	51.5524

(S)-4-Nitro-*N*-tosylbenzenesulfonimidoyl chloride (1f)

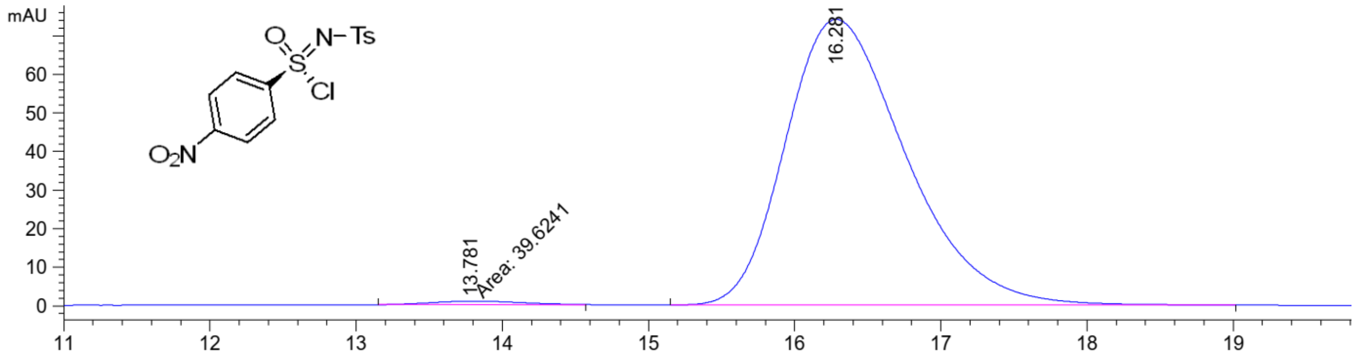

Peak #	RetTime [min]	Type	Width [min]	Area [mAU*s]	Height [mAU]	Area %
1	13.781	MM	0.7118	39.62409	9.27759e-1	0.9255
2	16.281	BB	0.8765	4241.70508	74.02173	99.0745

3-Hydroxy-2,2-dimethylpropyl (*R*)-4-nitro-*N*-tosylbenzenesulfonimidate (*rac*-3f)

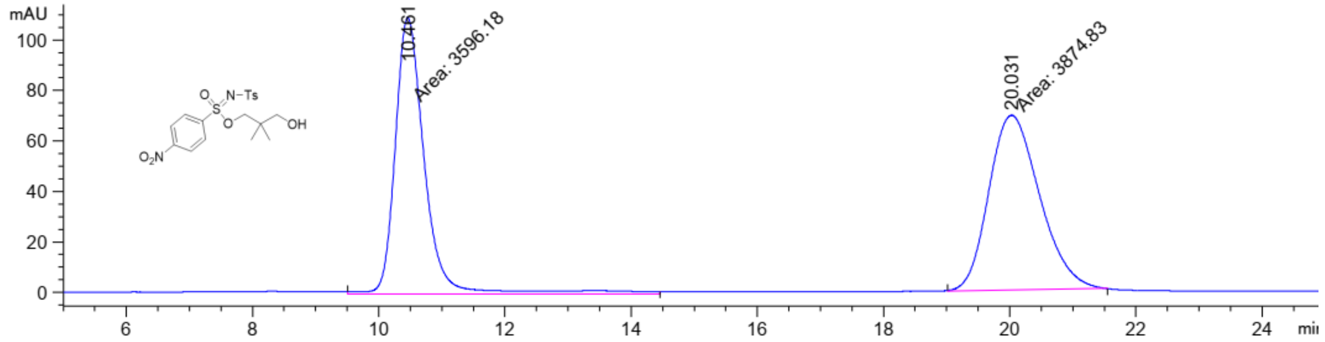

Peak #	RetTime [min]	Type	Width [min]	Area [mAU*s]	Height [mAU]	Area %
1	10.461	MM	0.5481	3596.18384	109.34753	48.1352
2	20.031	MM	0.9321	3874.82593	69.28537	51.8648

3-Hydroxy-2,2-dimethylpropyl (*R*)-4-nitro-*N*-tosylbenzenesulfonimidate (3f)

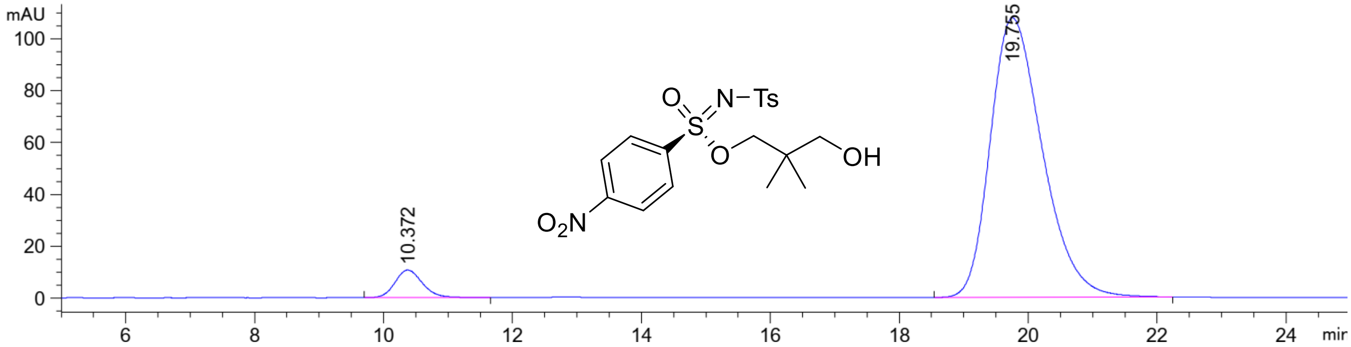

Peak #	RetTime [min]	Type	Width [min]	Area [mAU*s]	Height [mAU]	Area %
1	10.372	BB	0.4560	315.17953	10.62873	4.9531
2	19.755	BB	0.8655	6048.14063	107.64951	95.0469

Go to table of contents

(*S*)-4-Methoxy-*N*-tosylbenzenesulfonimidoyl chloride (*rac*-1g)

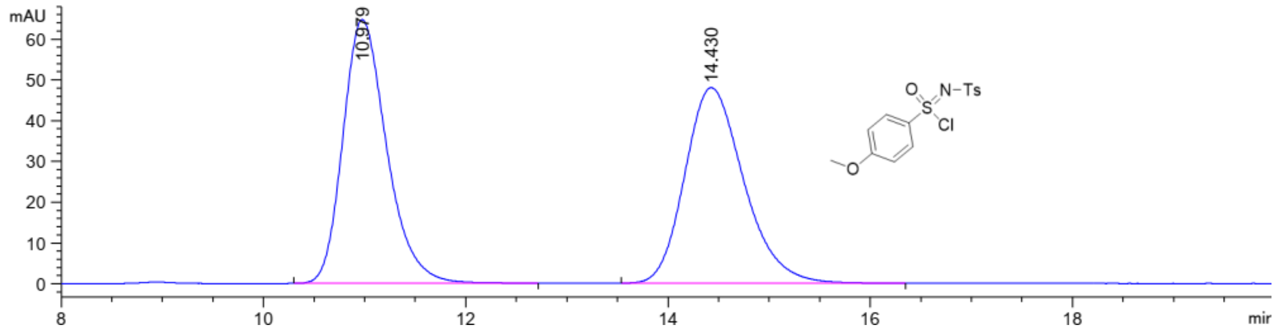

Peak #	RetTime [min]	Type	Width [min]	Area [mAU*s]	Height [mAU]	Area %
1	10.979	BB	0.4629	1945.42761	64.67532	49.9947
2	14.430	BB	0.6214	1945.84290	47.95629	50.0053

(*S*)-4-Methoxy-*N*-tosylbenzenesulfonimidoyl chloride (1g)

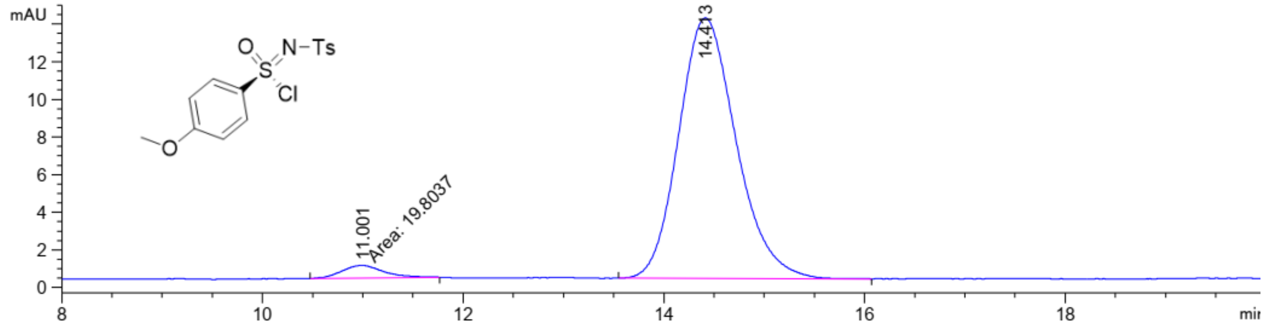

Peak #	RetTime [min]	Type	Width [min]	Area [mAU*s]	Height [mAU]	Area %
1	11.001	MM	0.4905	19.80368	6.72909e-1	3.4700
2	14.413	BB	0.6065	550.91095	13.83596	96.5300

3-Hydroxy-2,2-dimethylpropyl (*R*)-4-methoxy-*N*-tosylbenzenesulfonimidate (3g)

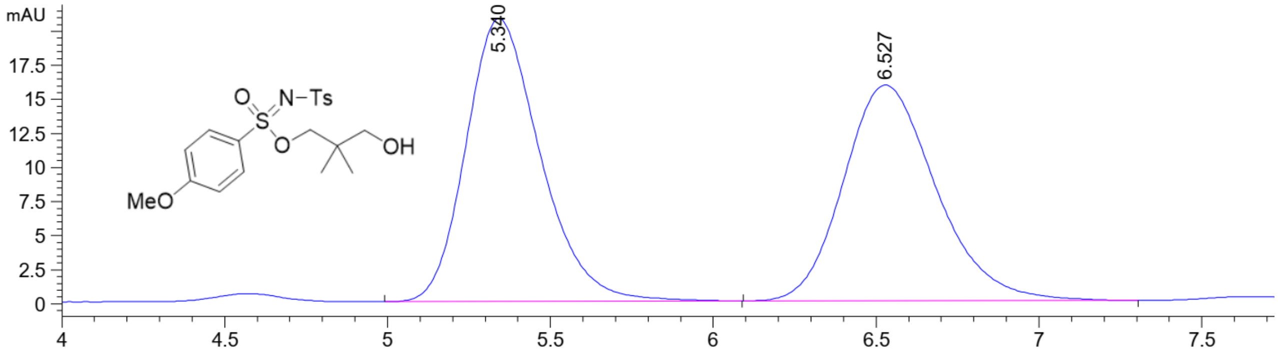

Peak #	RetTime [min]	Type	Width [min]	Area [mAU*s]	Height [mAU]	Area %
1	5.340	BB	0.2431	329.72150	20.77834	51.2404
2	6.527	BB	0.3051	313.75769	15.83717	48.7596

3-Hydroxy-2,2-dimethylpropyl (*R*)-4-methoxy-*N*-tosylbenzenesulfonimidate (3g)

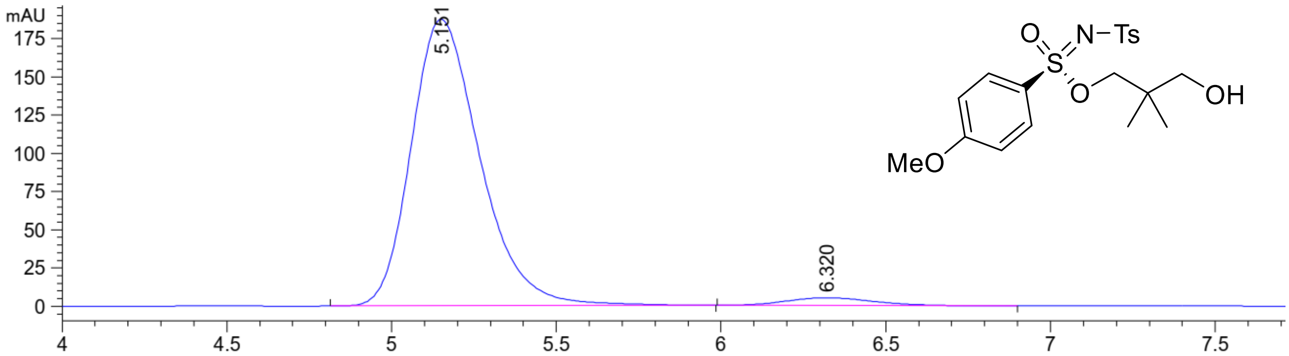

Peak #	RetTime [min]	Type	Width [min]	Area [mAU*s]	Height [mAU]	Area %
1	5.151	BB	0.2238	2726.57861	187.28975	96.6256
2	6.320	BB	0.2863	95.21866	5.13616	3.3744

(*S*)-4-(Difluoromethoxy)-*N*-tosylbenzenesulfonimidoyl chloride (*rac*-1h)

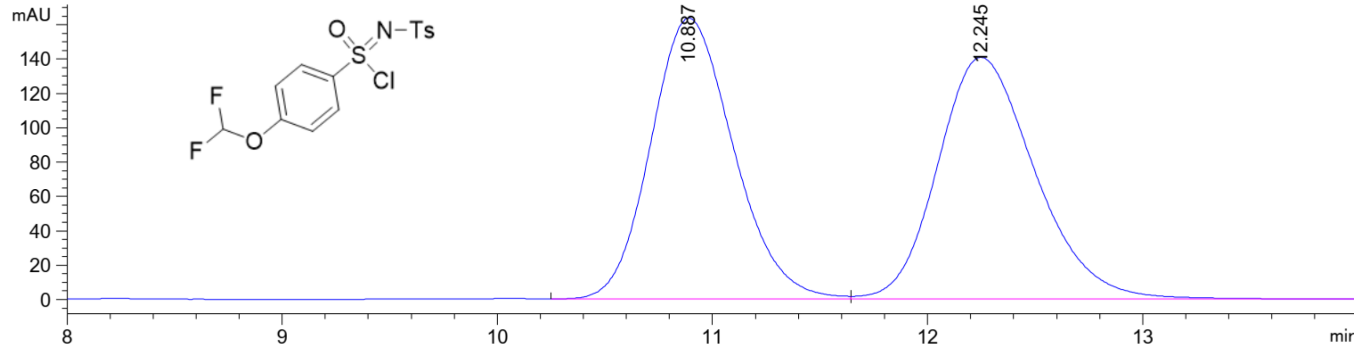

Peak #	RetTime [min]	Type	Width [min]	Area [mAU*s]	Height [mAU]	Area %
1	10.887	BV	0.4141	4369.14502	163.34166	49.6676
2	12.245	VB	0.4831	4427.62354	140.69675	50.3324

(*S*)-4-(Difluoromethoxy)-*N*-tosylbenzenesulfonimidoyl chloride (1h)

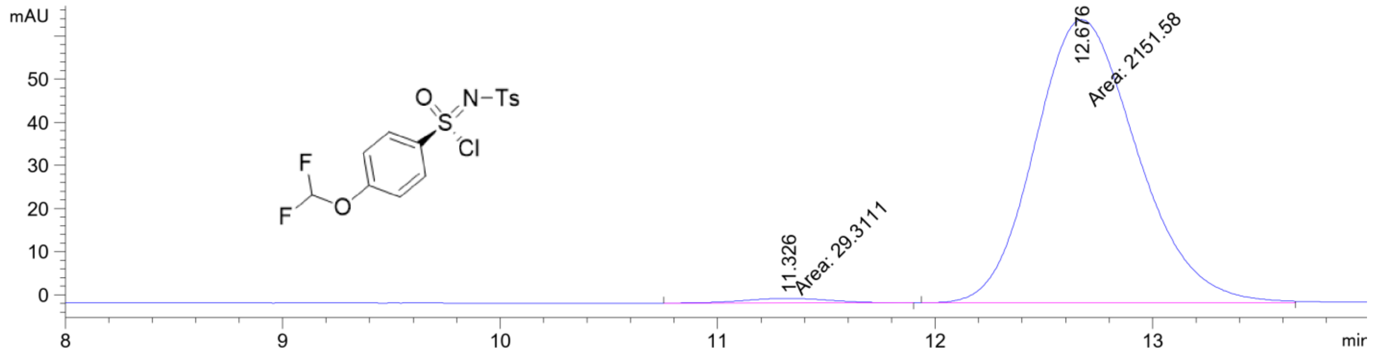

Peak #	RetTime [min]	Type	Width [min]	Area [mAU*s]	Height [mAU]	Area %
1	11.326	MM	0.4545	29.31107	1.07489	1.3440
2	12.676	MF	0.5460	2151.57837	65.68060	98.6560

3-Hydroxy-2,2-dimethylpropyl (*R*)-4-(difluoromethoxy)-*N*-tosylbenzenesulfonimidate (*rac*-3h)

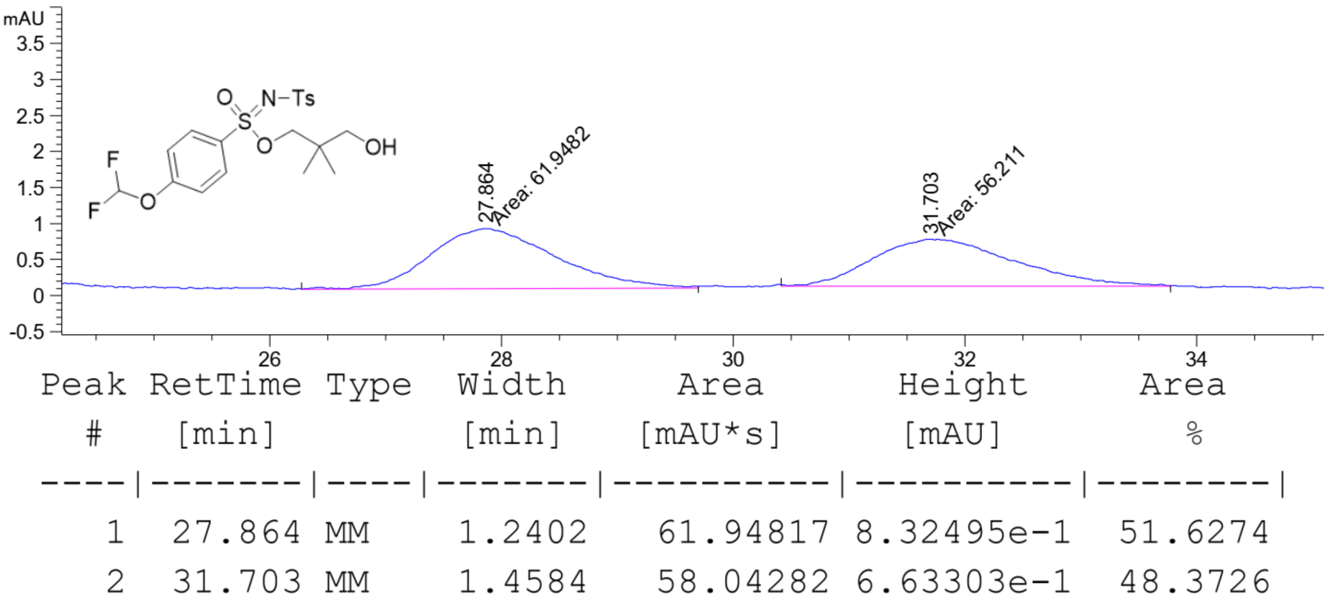

Peak #	RetTime [min]	Type	Width [min]	Area [mAU*s]	Height [mAU]	Area %
1	27.864	MM	1.2402	61.94817	8.32495e-1	51.6274
2	31.703	MM	1.4584	58.04282	6.63303e-1	48.3726

3-Hydroxy-2,2-dimethylpropyl (*R*)-4-(difluoromethoxy)-*N*-tosylbenzenesulfonimidate (3h)

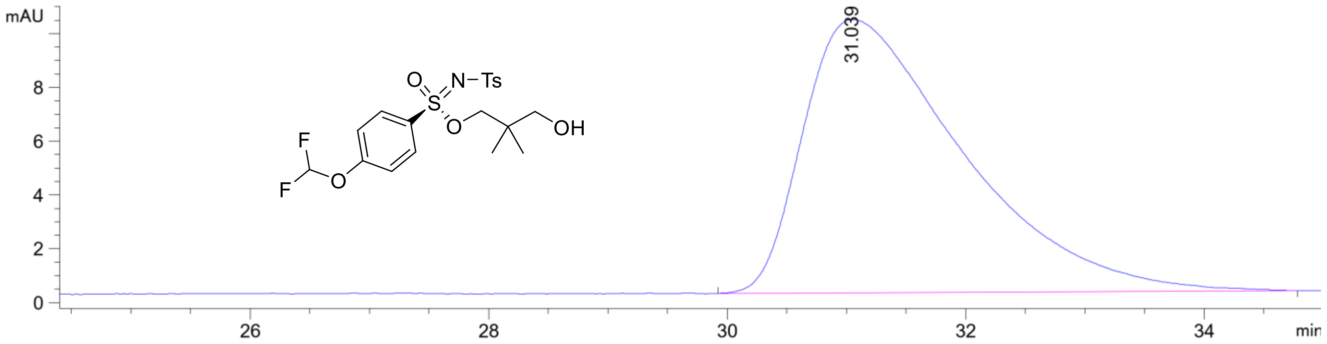

Peak #	RetTime [min]	Type	Width [min]	Area [mAU*s]	Height [mAU]	Area %
1	31.039	BB	1.1865	947.14929	10.17020	100.0000

(*S*)-*N*-Tosyl-3-(trifluoromethoxy)benzenesulfonimidoyl chloride (*rac*-1i)

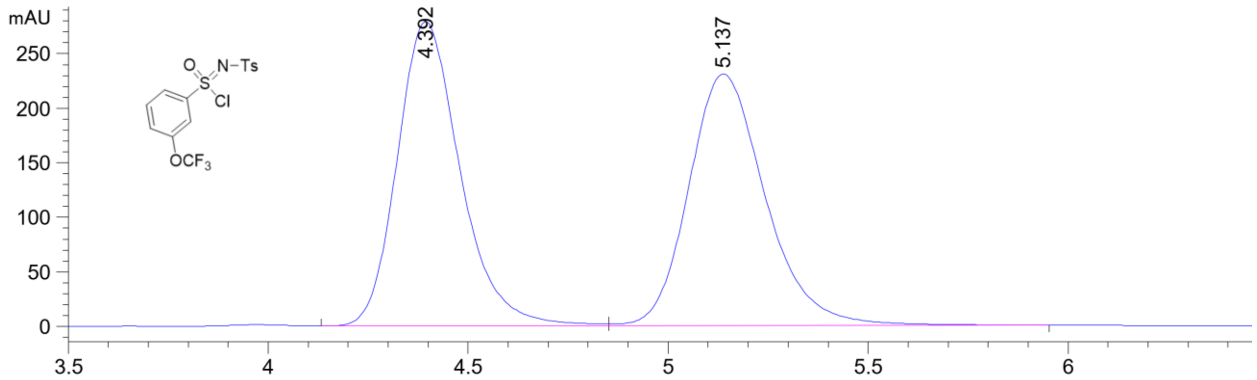

Peak #	RetTime [min]	Type	Width [min]	Area [mAU*s]	Height [mAU]	Area %
1	4.392	VV	0.1678	3055.26221	278.89441	49.8657
2	5.137	VB	0.2033	3071.72021	230.69977	50.1343

(*S*)-*N*-Tosyl-3-(trifluoromethoxy)benzenesulfonimidoyl chloride (1i)

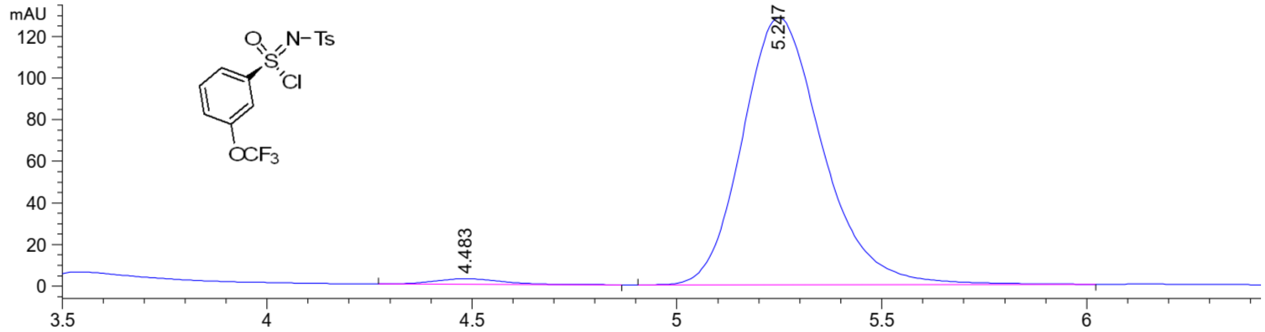

Peak #	RetTime [min]	Type	Width [min]	Area [mAU*s]	Height [mAU]	Area %
1	4.483	BB	0.1722	30.02319	2.69115	1.6904
2	5.247	BB	0.2087	1746.05359	128.32060	98.3096

Go to table of contents

3-Hydroxy-2,2-dimethylpropyl *(R)*-*N*-tosyl-3-(trifluoromethoxy)benzenesulfonimidate (3i)

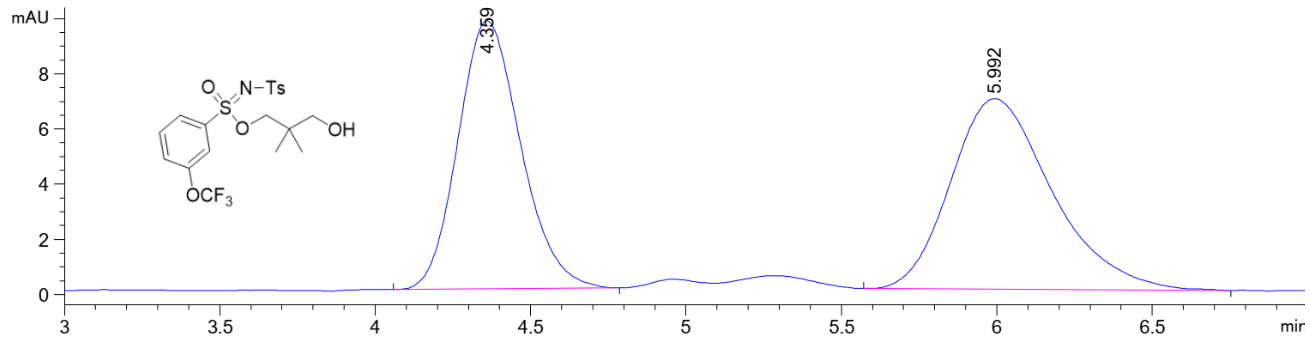

Peak #	RetTime [min]	Type	Width [min]	Area [mAU*s]	Height [mAU]	Area %
1	4.359	MM	0.2397	141.28099	9.82328	48.9833
2	5.992	MM	0.3623	147.14590	6.76858	51.0167

3-Hydroxy-2,2-dimethylpropyl *(R)*-*N*-tosyl-3-(trifluoromethoxy)benzenesulfonimidate (3i)

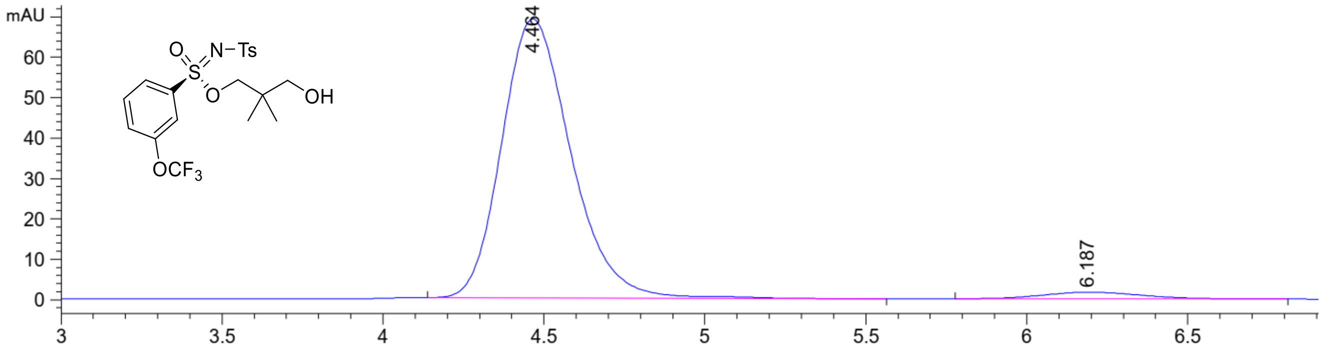

Peak #	RetTime [min]	Type	Width [min]	Area [mAU*s]	Height [mAU]	Area %
1	4.464	BB	0.2304	1031.95996	69.02146	96.6731
2	6.187	BB	0.3163	35.51350	1.62935	3.3269

Go to table of contents

***(S)*-3-(Methylsulfonyl)-*N*-tosylbenzenesulfonimidoyl chloride (*rac*-1j)**

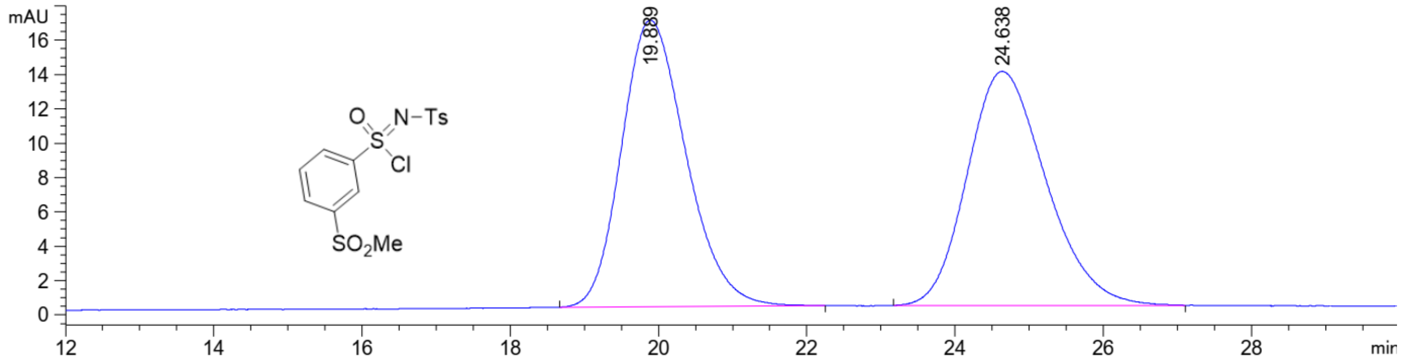

Peak #	RetTime [min]	Type	Width [min]	Area [mAU*s]	Height [mAU]	Area %
1	19.889	BB	0.9004	1007.71667	16.69081	49.9877
2	24.638	BB	1.0730	1008.21460	13.63002	50.0123

***(S)*-3-(Methylsulfonyl)-*N*-tosylbenzenesulfonimidoyl chloride (1j)**

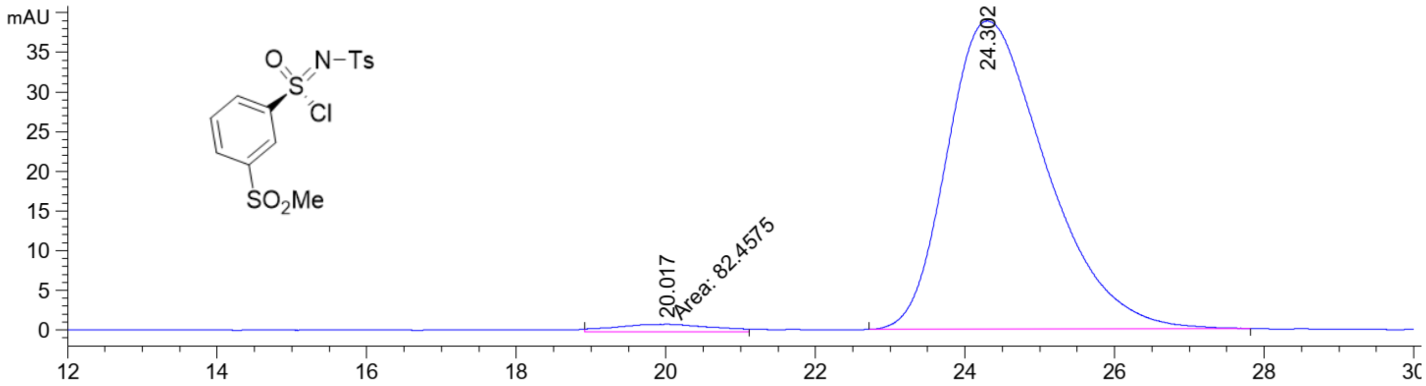

Peak #	RetTime [min]	Type	Width [min]	Area [mAU*s]	Height [mAU]	Area %
1	20.017	MM	1.4959	82.45747	9.18716e-1	2.2417
2	24.302	BB	1.3241	3595.83569	38.83910	97.7583

3-Hydroxy-2,2-dimethylpropyl *(R)*-3-(methylsulfonyl)-*N*-tosylbenzenesulfonimidate (*rac*-3j)

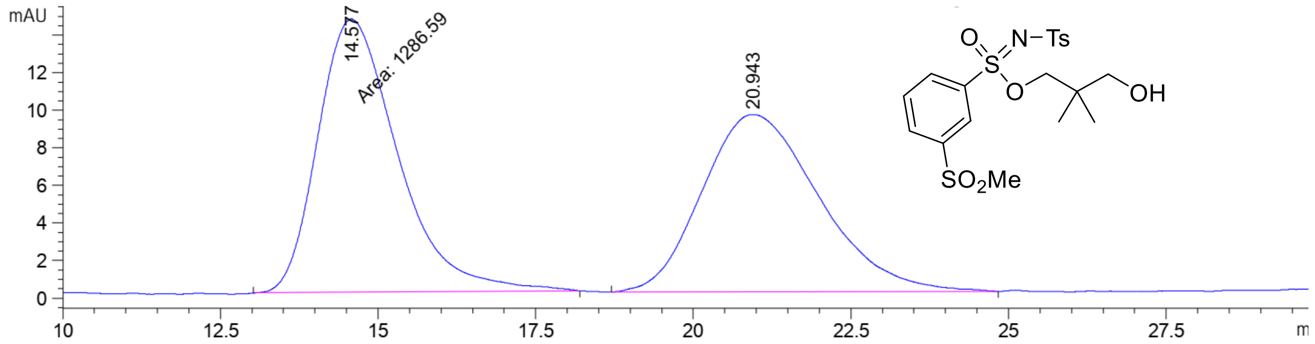

Peak #	RetTime [min]	Type	Width [min]	Area [mAU*s]	Height [mAU]	Area %
1	14.577	MM	1.4736	1286.59045	14.55193	51.4161
2	20.943	BB	1.5312	1215.72229	9.43528	48.5839

3-Hydroxy-2,2-dimethylpropyl *(R)*-3-(methylsulfonyl)-*N*-tosylbenzenesulfonimidate (3j)

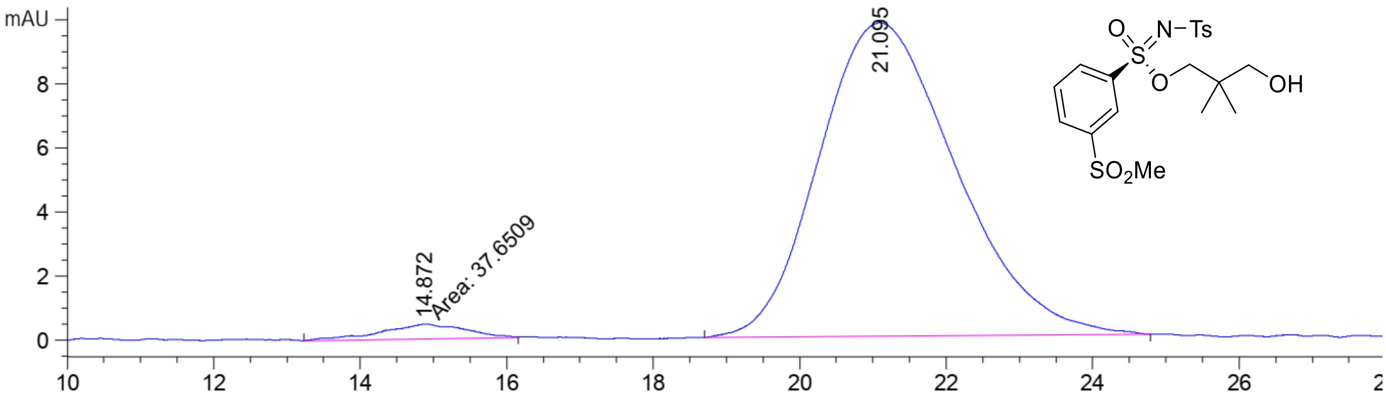

Peak #	RetTime [min]	Type	Width [min]	Area [mAU*s]	Height [mAU]	Area %
1	14.872	MM	1.3629	37.65091	4.60413e-1	2.8776
2	21.095	BB	1.5343	1270.75659	9.77726	97.1224

(*S*)-4-Cyano-*N*-tosylbenzenesulfonimidoyl chloride (*rac*-1k)

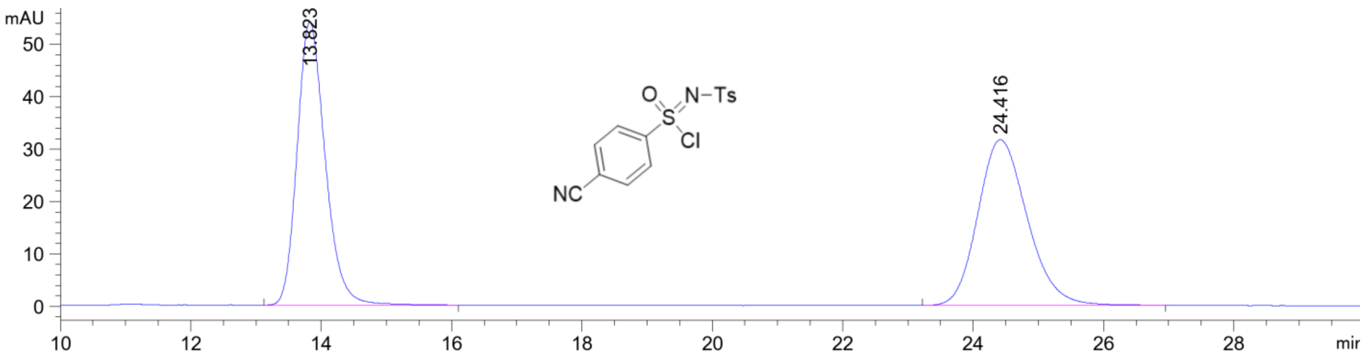

Peak #	RetTime [min]	Type	Width [min]	Area [mAU*s]	Height [mAU]	Area %
1	13.823	BB	0.4567	1617.66345	54.11826	49.2517
2	24.416	BB	0.8066	1666.82166	31.63894	50.7483

(*S*)-4-Cyano-*N*-tosylbenzenesulfonimidoyl chloride (1k)

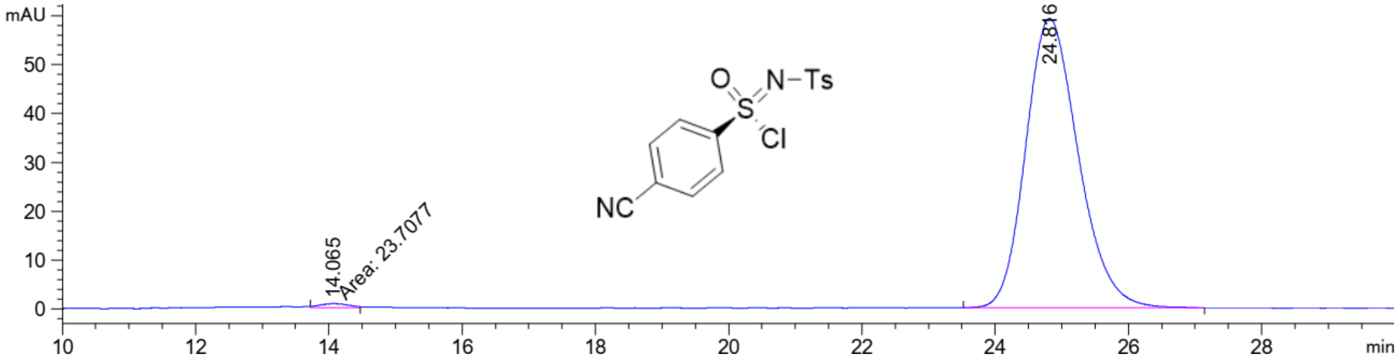

Peak #	RetTime [min]	Type	Width [min]	Area [mAU*s]	Height [mAU]	Area %
1	14.065	MM	0.4981	23.70766	7.93280e-1	0.7255
2	24.816	BB	0.8476	3244.28394	59.17834	99.2745

3-Hydroxy-2,2-dimethylpropyl (*R*)-4-cyano-*N*-tosylbenzenesulfonimidate (*rac*-3k)

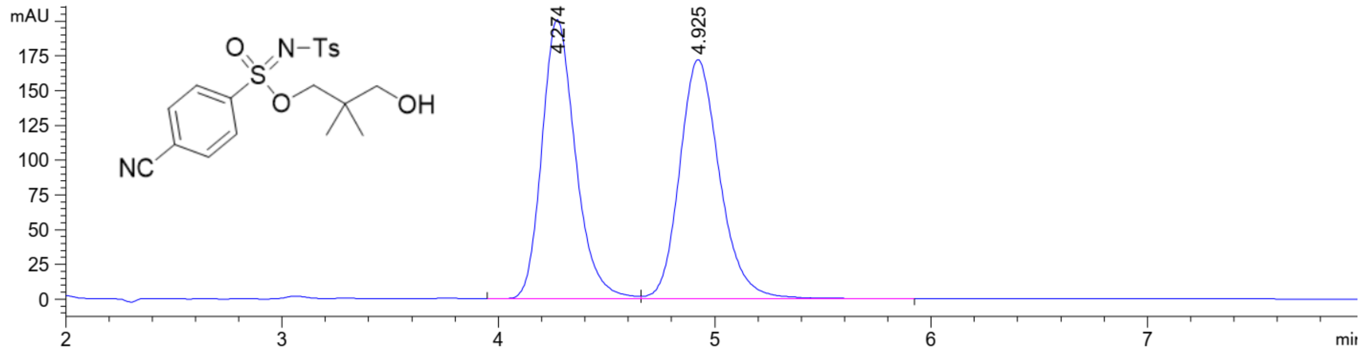

Peak #	RetTime [min]	Type	Width [min]	Area [mAU*s]	Height [mAU]	Area %
1	4.274	BV	0.1656	2162.02100	200.77869	49.3798
2	4.925	VB	0.1983	2216.33154	172.01622	50.6202

3-Hydroxy-2,2-dimethylpropyl (*R*)-4-cyano-*N*-tosylbenzenesulfonimidate (3k)

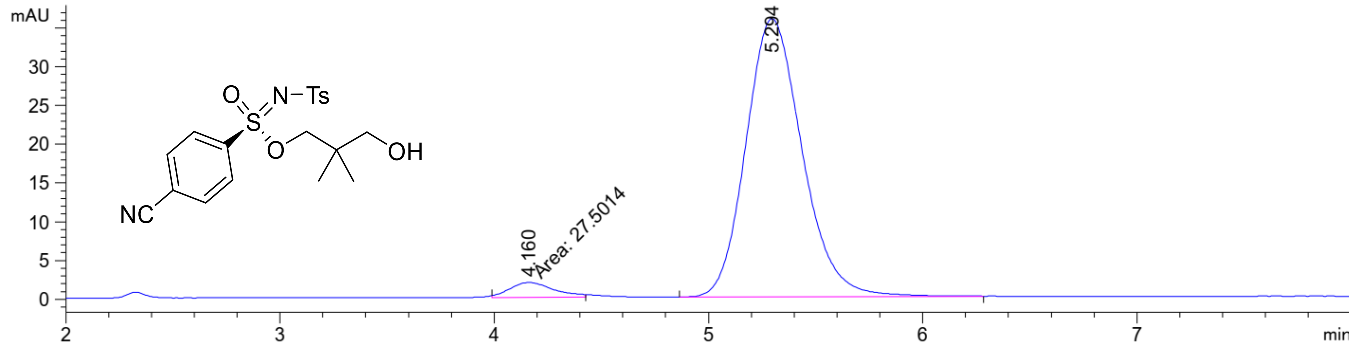

Peak #	RetTime [min]	Type	Width [min]	Area [mAU*s]	Height [mAU]	Area %
1	4.160	MF	0.2400	27.50136	1.90978	4.0321
2	5.294	BB	0.2808	654.56201	35.88826	95.9679

Go to table of contents

(*S*)-4-Methyl-*N*-tosylbenzenesulfonimidoyl chloride (*rac*-1l)

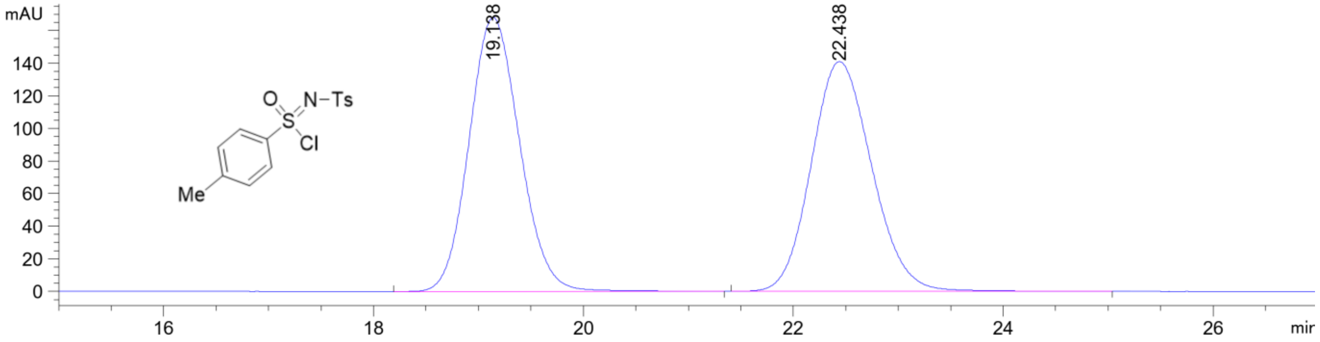

Peak #	RetTime [min]	Type	Width [min]	Area [mAU*s]	Height [mAU]	Area %
1	19.138	BB	0.5225	5626.16406	168.06711	49.9118
2	22.438	BB	0.6237	5646.04102	140.83417	50.0882

(*S*)-4-Methyl-*N*-tosylbenzenesulfonimidoyl chloride (1l)

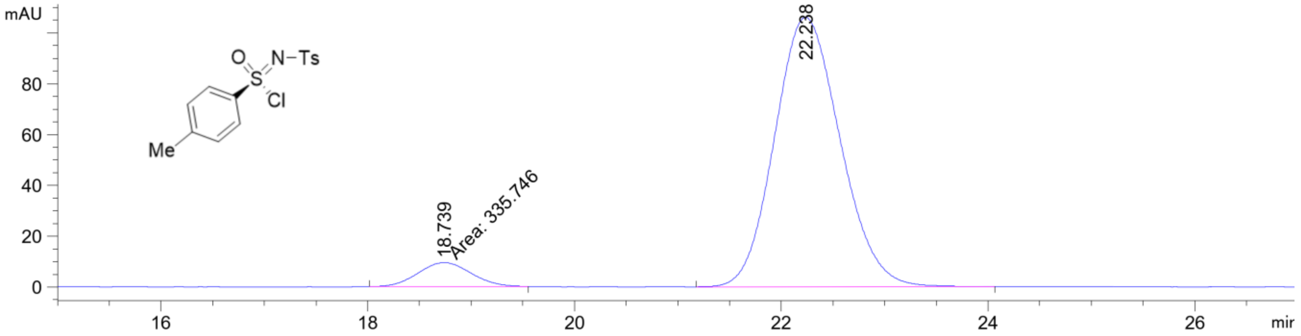

Peak #	RetTime [min]	Type	Width [min]	Area [mAU*s]	Height [mAU]	Area %
1	18.739	MM	0.5950	335.74622	9.40460	6.7096
2	22.238	BB	0.6838	4668.25586	106.00506	93.2904

3-Hydroxy-2,2-dimethylpropyl *(R)*-4-methyl-*N*-tosylbenzenesulfonimidate (*rac*-3l)

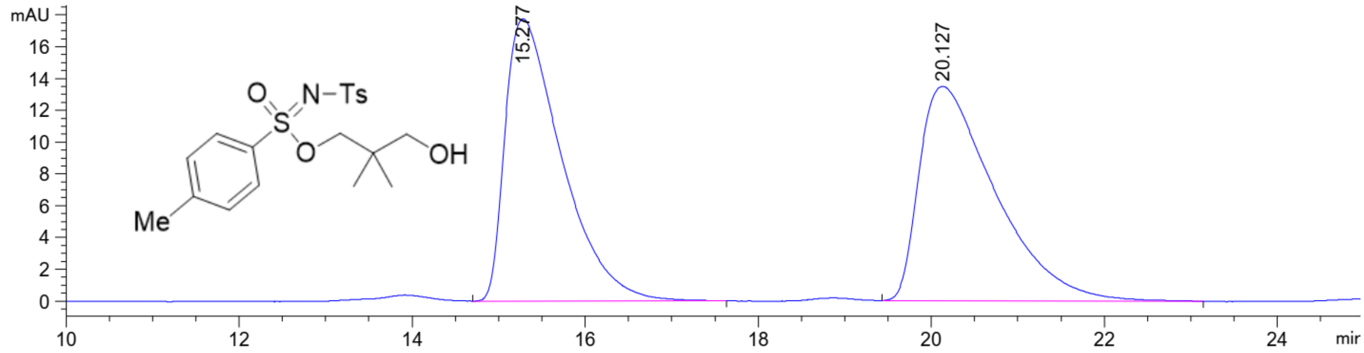

Peak #	RetTime [min]	Type	Width [min]	Area [mAU*s]	Height [mAU]	Area %
1	15.277	BB	0.6539	805.87939	17.74241	49.5207
2	20.127	BB	0.8551	821.47803	13.47726	50.4793

3-Hydroxy-2,2-dimethylpropyl *(R)*-4-methyl-*N*-tosylbenzenesulfonimidate (3l)

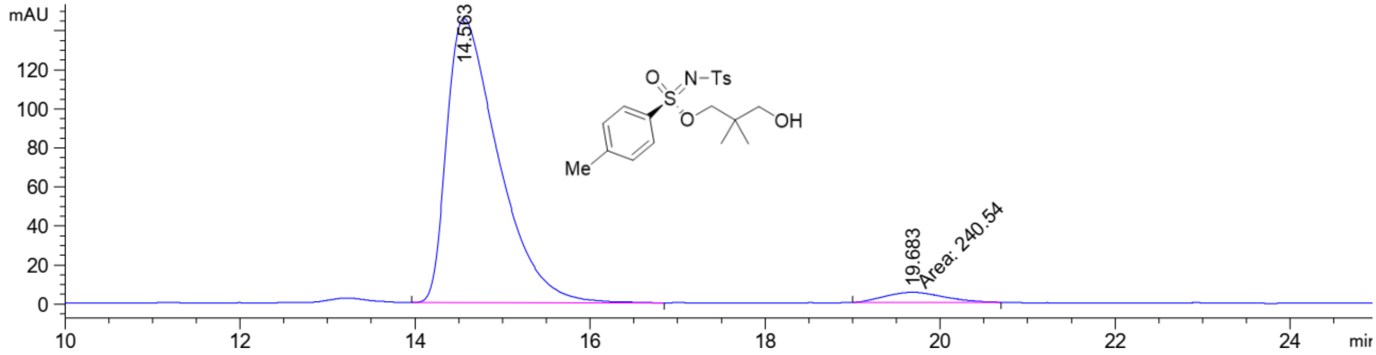

Peak #	RetTime [min]	Type	Width [min]	Area [mAU*s]	Height [mAU]	Area %
1	14.563	BB	0.6074	5962.83447	145.72714	96.1224
2	19.683	MM	0.7776	240.54031	5.15585	3.8776

(*S*)-4-(*tert*-butyl)-*N*-tosylbenzenesulfonimidoyl chloride (*rac*-1m)

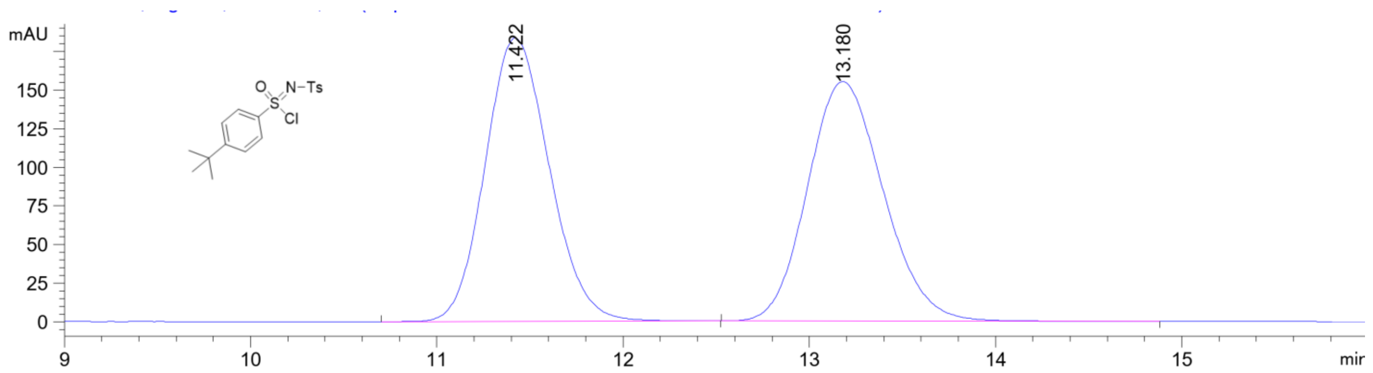

Peak #	RetTime [min]	Type	Width [min]	Area [mAU*s]	Height [mAU]	Area %
1	11.422	BB	0.3713	4389.88965	183.35060	50.0182
2	13.180	BB	0.4405	4386.70313	154.84978	49.9818

(*S*)-4-(*tert*-butyl)-*N*-tosylbenzenesulfonimidoyl chloride (1m)

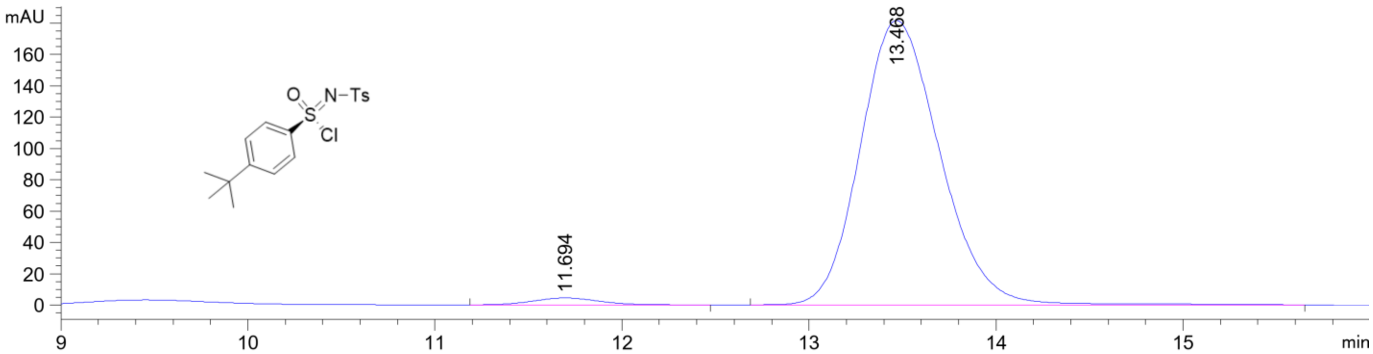

Peak #	RetTime [min]	Type	Width [min]	Area [mAU*s]	Height [mAU]	Area %
1	11.694	BB	0.3705	106.21300	4.35611	1.9396
2	13.468	BB	0.4571	5369.72412	181.52519	98.0604

3-Hydroxy-2,2-dimethylpropyl (*R*)-4-(*tert*-butyl)-*N*-tosylbenzenesulfonimidoyl chloride (3m)

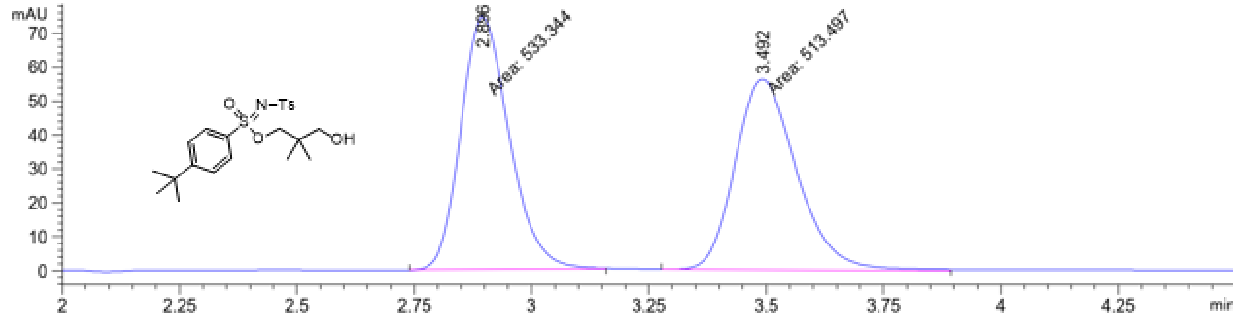

Peak #	RetTime [min]	Type	Width [min]	Area [mAU*s]	Height [mAU]	Area %
1	2.896	MM	0.1193	533.34406	74.52577	50.9479
2	3.492	MM	0.1522	513.49707	56.24666	49.0521

3-Hydroxy-2,2-dimethylpropyl (*R*)-4-(*tert*-butyl)-*N*-tosylbenzenesulfonimidoyl chloride (3m)

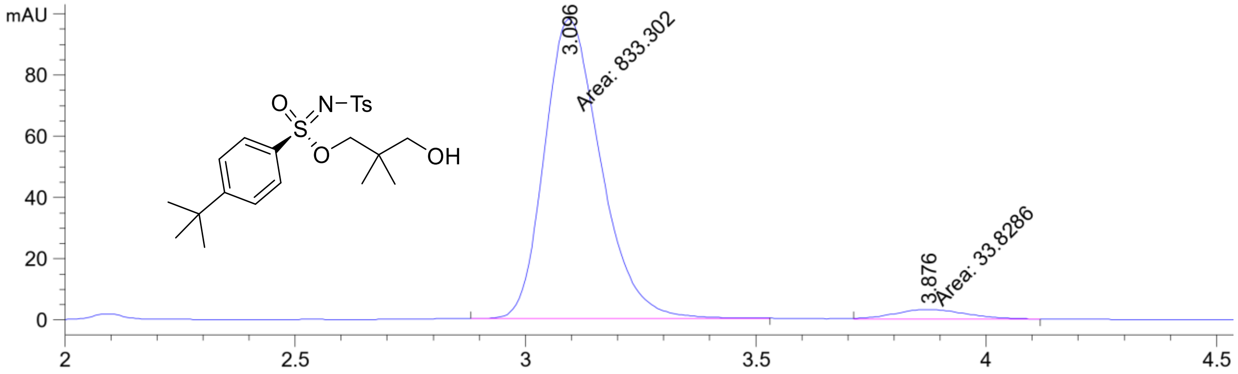

Peak #	RetTime [min]	Type	Width [min]	Area [mAU*s]	Height [mAU]	Area %
1	3.096	BB	0.1314	833.85773	97.84484	96.0695
2	3.876	BB	0.1715	34.11560	3.12057	3.9305

Go to table of contents

(*S*)-*N*-Tosyl-[1,1'-biphenyl]-4-sulfonimidoyl chloride (*rac*-1n)

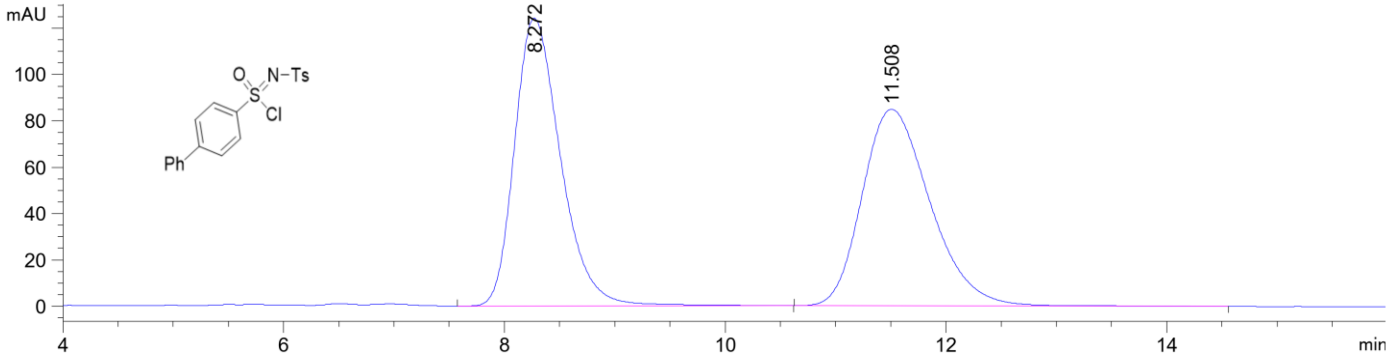

Peak #	RetTime [min]	Type	Width [min]	Area [mAU*s]	Height [mAU]	Area %
1	8.272	BB	0.4444	3603.30103	124.23397	49.8280
2	11.508	BB	0.6588	3628.17114	84.85886	50.1720

(*S*)-*N*-Tosyl-[1,1'-biphenyl]-4-sulfonimidoyl chloride (1n)

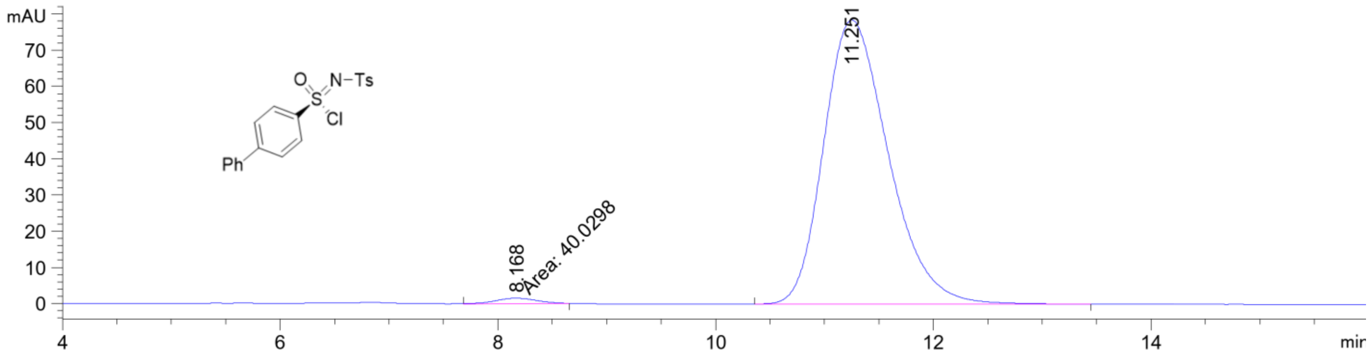

Peak #	RetTime [min]	Type	Width [min]	Area [mAU*s]	Height [mAU]	Area %
1	8.168	MM	0.4427	40.02977	1.50715	1.2191
2	11.251	BB	0.6403	3243.41724	78.46113	98.7809

Go to table of contents

3-Hydroxy-2,2-dimethylpropyl (*R*)-*N*-tosyl-[1,1'-biphenyl]-4-sulfonimidate (*rac*-3n)

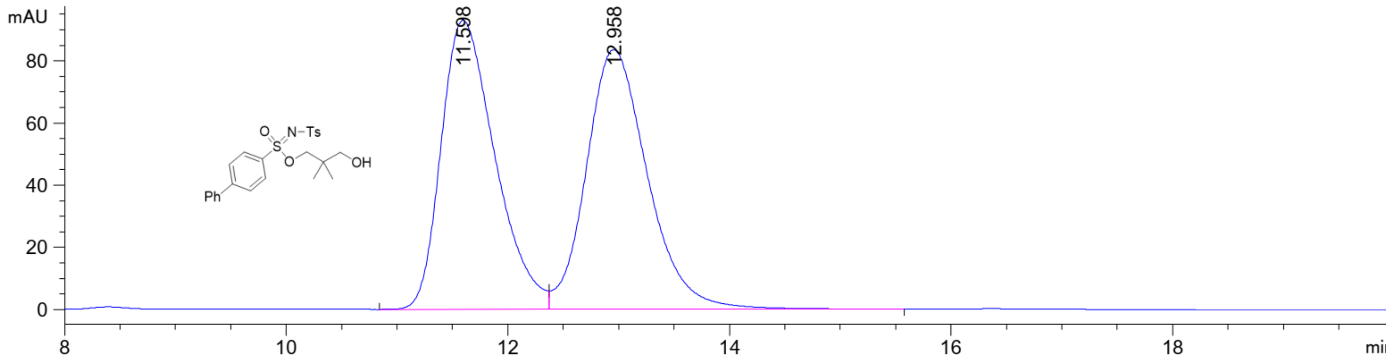

Peak #	RetTime [min]	Type	Width [min]	Area [mAU*s]	Height [mAU]	Area %
1	11.598	BV	0.5200	3140.64478	93.00333	49.4841
2	12.958	VB	0.5837	3206.12744	83.54938	50.5159

3-Hydroxy-2,2-dimethylpropyl (*R*)-*N*-tosyl-[1,1'-biphenyl]-4-sulfonimidate (3n)

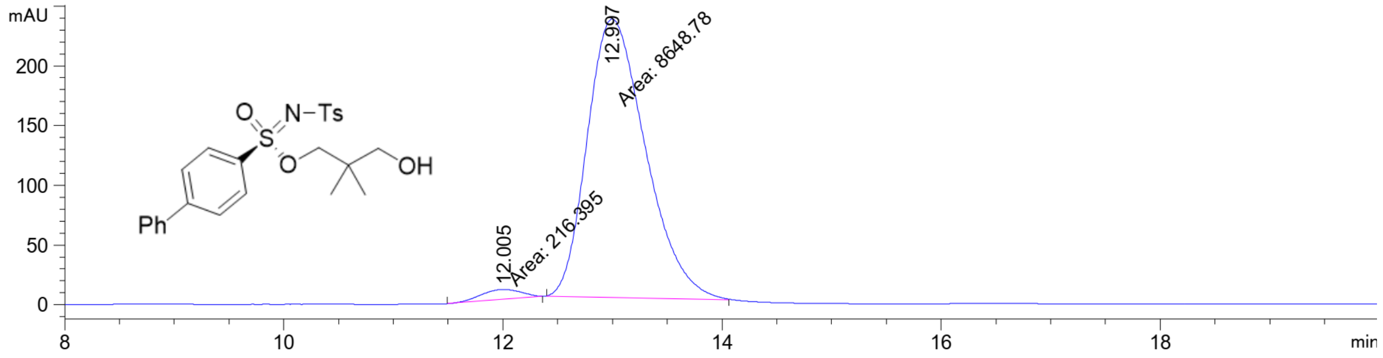

Peak #	RetTime [min]	Type	Width [min]	Area [mAU*s]	Height [mAU]	Area %
1	12.005	MM	0.4310	216.39471	8.36799	2.4410
2	12.997	MM	0.6178	8648.77539	233.32040	97.5590

(S)-4'-Fluoro-N-tosyl-[1,1'-biphenyl]-4-sulfonimidoyl chloride (rac-1o)

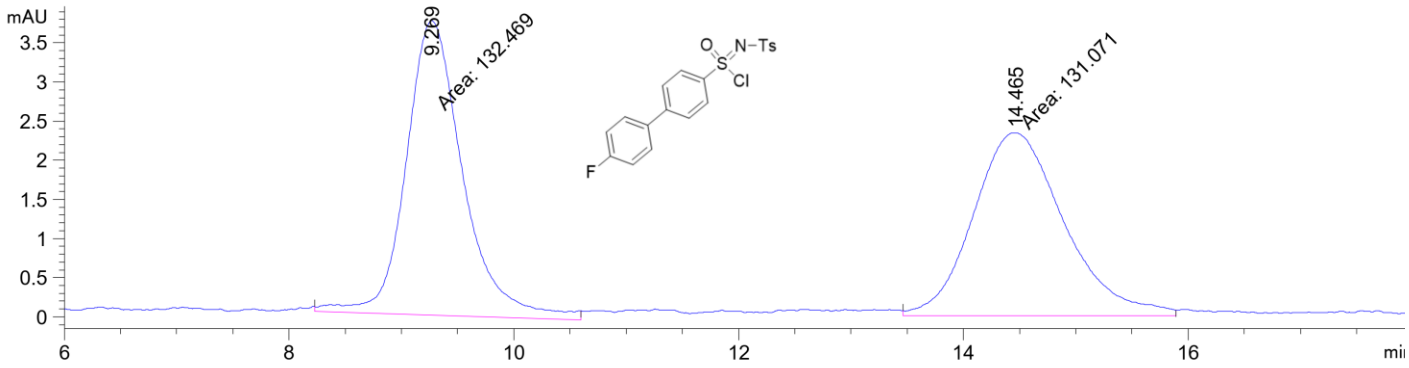

Peak #	RetTime [min]	Type	Width [min]	Area [mAU*s]	Height [mAU]	Area %
1	9.269	BB	0.4646	118.85872	3.66298	49.5451
2	14.465	MM	0.8891	121.04153	2.26911	50.4549

(S)-4'-Fluoro-N-tosyl-[1,1'-biphenyl]-4-sulfonimidoyl chloride (1o)

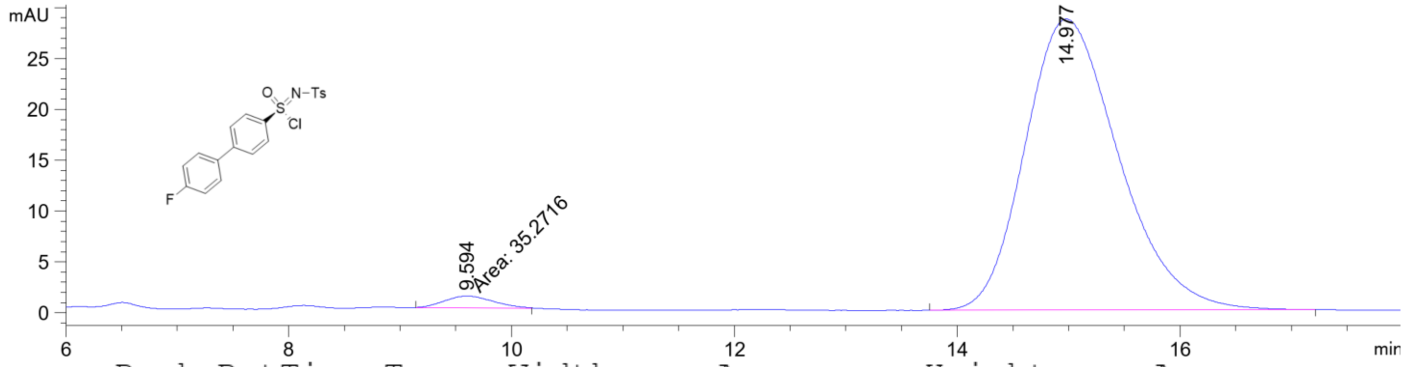

Peak #	RetTime [min]	Type	Width [min]	Area [mAU*s]	Height [mAU]	Area %
1	9.594	MM	0.5081	35.33644	1.15905	2.0640
2	14.977	BB	0.8870	1676.72913	28.64057	97.9360

3-hydroxy-2,2-dimethylpropyl (*R*)-4'-fluoro-*N*-tosyl-[1,1'-biphenyl]-4-sulfonimidate (*rac*-3o)

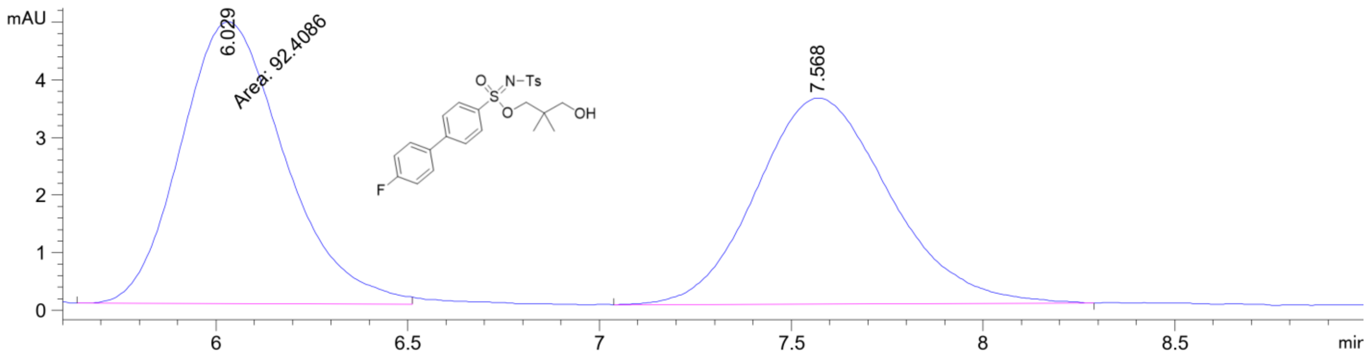

Peak #	RetTime [min]	Type	Width [min]	Area [mAU*s]	Height [mAU]	Area %
1	6.029	BB	0.2909	93.46066	4.89129	51.9816
2	7.568	BB	0.3602	86.33504	3.56914	48.0184

3-hydroxy-2,2-dimethylpropyl (*R*)-4'-fluoro-*N*-tosyl-[1,1'-biphenyl]-4-sulfonimidate (3o)

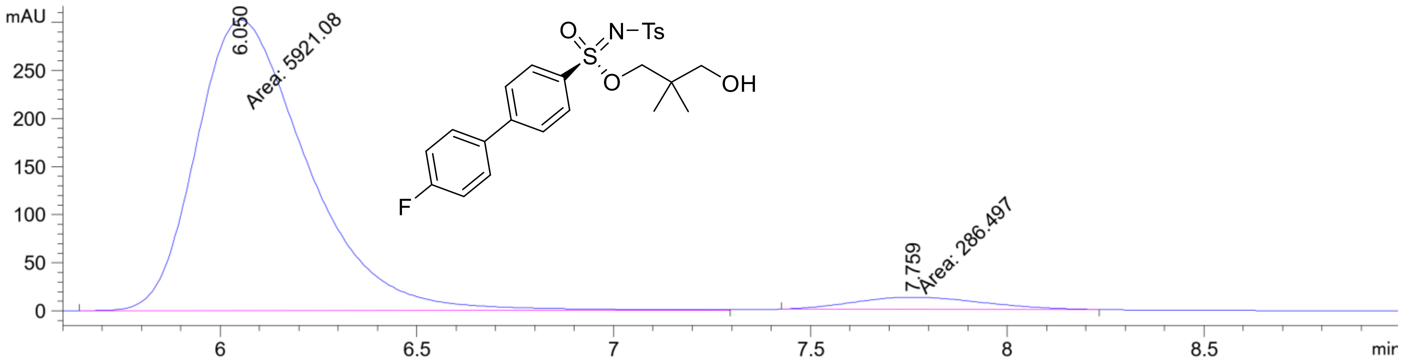

Peak #	RetTime [min]	Type	Width [min]	Area [mAU*s]	Height [mAU]	Area %
1	6.050	MM	0.3277	5949.27148	302.60565	95.9686
2	7.759	MM	0.3515	249.91568	11.84961	4.0314

Go to table of contents

(*S*)-2,5-Difluoro-*N*-tosylbenzenesulfonimidoyl chloride (*rac*-1p)

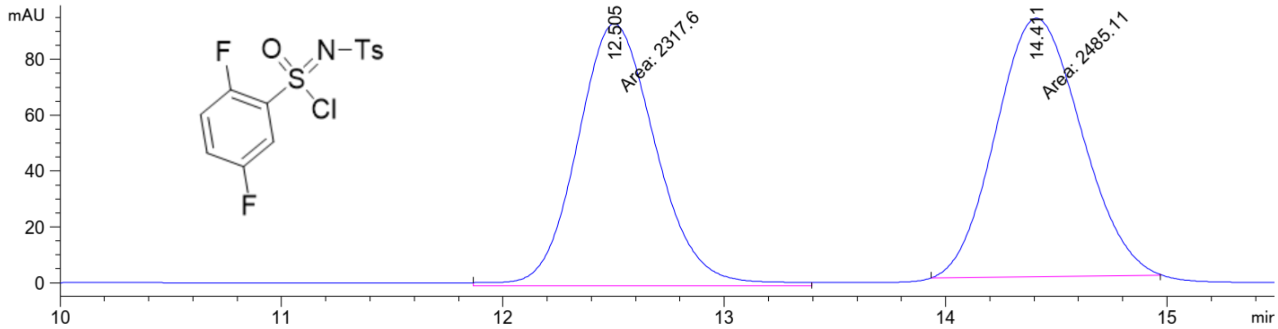

Peak #	RetTime [min]	Type	Width [min]	Area [mAU*s]	Height [mAU]	Area %
1	12.505	MM	0.4122	2317.60254	93.69776	48.2561
2	14.411	MM	0.4476	2485.10718	92.52932	51.7439

(*S*)-2,5-Difluoro-*N*-tosylbenzenesulfonimidoyl chloride (1p)

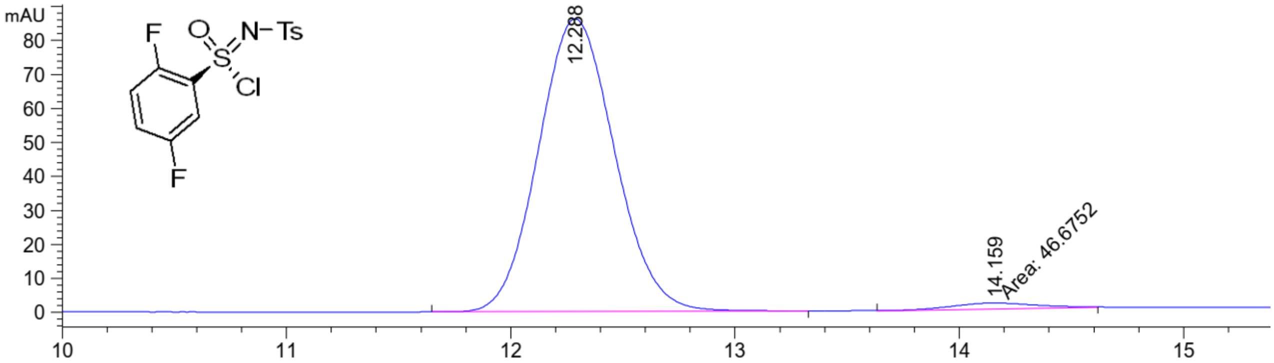

Peak #	RetTime [min]	Type	Width [min]	Area [mAU*s]	Height [mAU]	Area %
1	12.288	BB	0.3695	2033.81287	86.12432	97.7565
2	14.159	MM	0.4464	46.67522	1.74269	2.2435

Go to table of contents

3-Hydroxy-2,2-dimethylpropyl (*R*)-2,5-difluoro-*N*-tosylbenzenesulfonimidate (*rac*-3p)

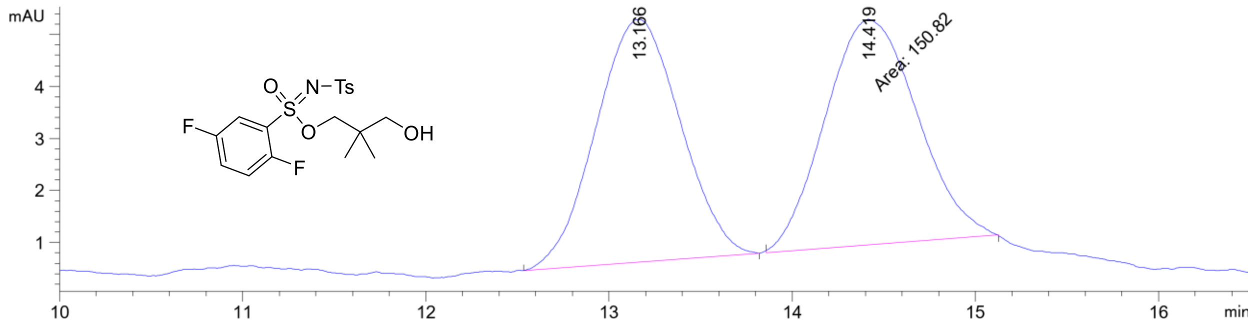

Peak #	RetTime [min]	Type	Width [min]	Area [mAU*s]	Height [mAU]	Area %
1	13.166	BB	0.4502	149.52011	4.66146	44.9729
2	14.419	BB	0.4798	182.94693	4.58228	55.0271

3-Hydroxy-2,2-dimethylpropyl (*R*)-2,5-difluoro-*N*-tosylbenzenesulfonimidate (3p)

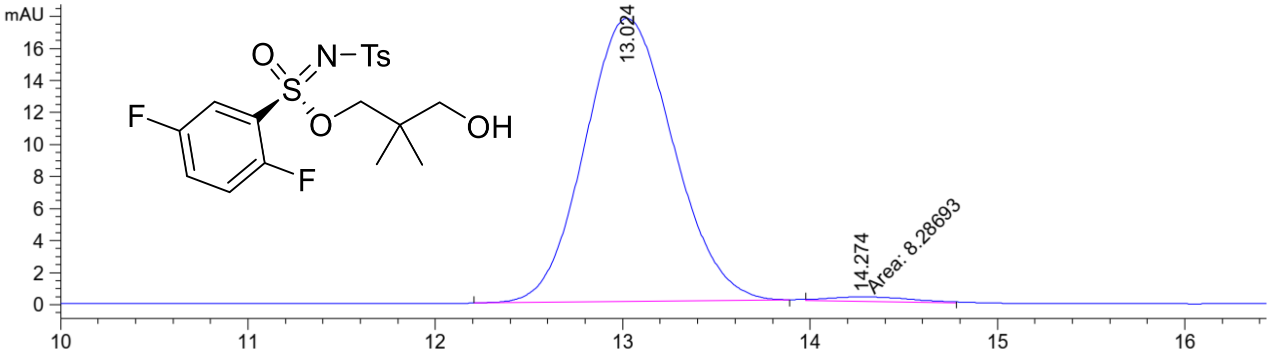

Peak #	RetTime [min]	Type	Width [min]	Area [mAU*s]	Height [mAU]	Area %
1	13.024	BB	0.5067	579.98706	17.68089	98.5913
2	14.274	MM	0.5049	8.28693	2.73574e-1	1.4087

Go to table of contents

(S)-3,5-Dichloro-N-tosylbenzenesulfonimidoyl chloride (rac-1q)

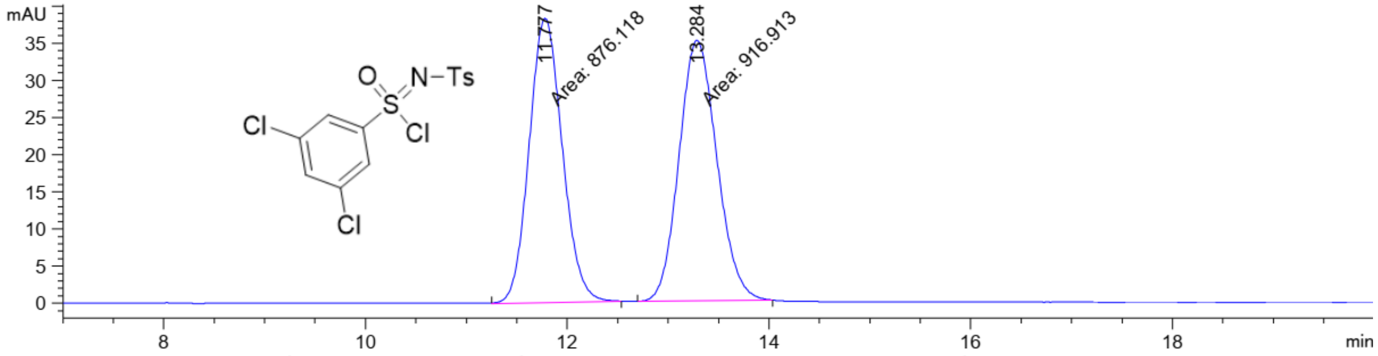

Peak #	RetTime [min]	Type	Width [min]	Area [mAU*s]	Height [mAU]	Area %
1	11.777	MM	0.3897	903.09210	38.62693	49.2939
2	13.284	MM	0.4390	928.96588	35.26501	50.7061

(S)-3,5-Dichloro-N-tosylbenzenesulfonimidoyl chloride (1q)

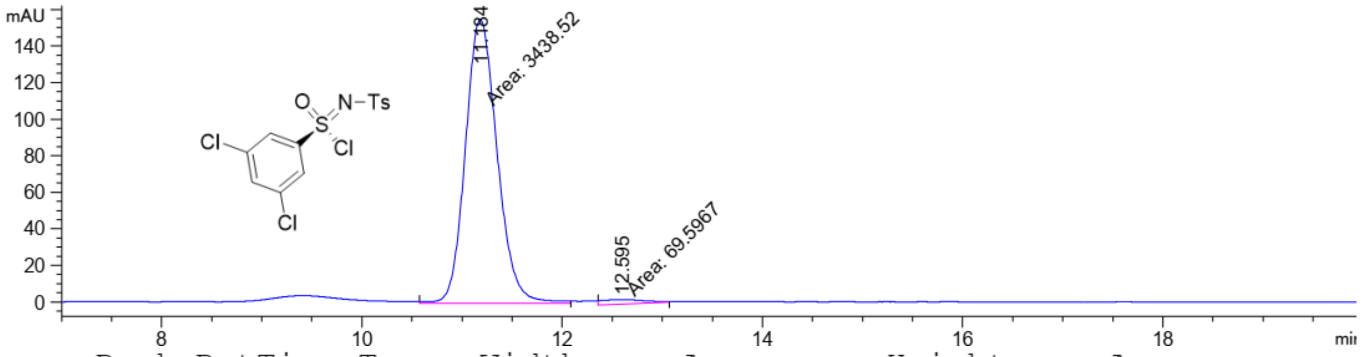

Peak #	RetTime [min]	Type	Width [min]	Area [mAU*s]	Height [mAU]	Area %
1	11.184	BB	0.3360	3333.97974	154.33589	99.3234
2	12.595	MM	0.3731	22.71291	1.01456	0.6766

3-Hydroxy-2,2-dimethylpropyl *(R)*-3,5-dichloro-*N*-tosylbenzenesulfonimidate (*rac*-3q)

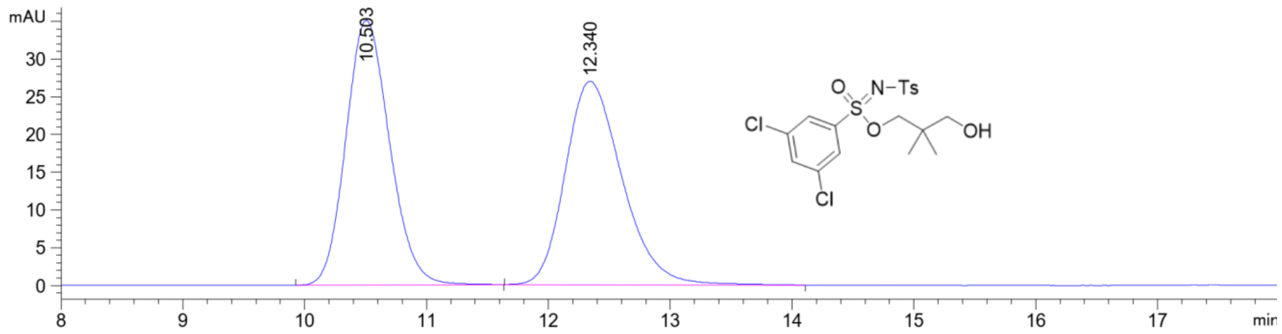

Peak #	RetTime [min]	Type	Width [min]	Area [mAU*s]	Height [mAU]	Area %
1	10.503	BB	0.3848	873.55475	35.05161	49.9656
2	12.340	BB	0.4990	874.75702	26.92651	50.0344

3-Hydroxy-2,2-dimethylpropyl *(R)*-3,5-dichloro-*N*-tosylbenzenesulfonimidate (3q)

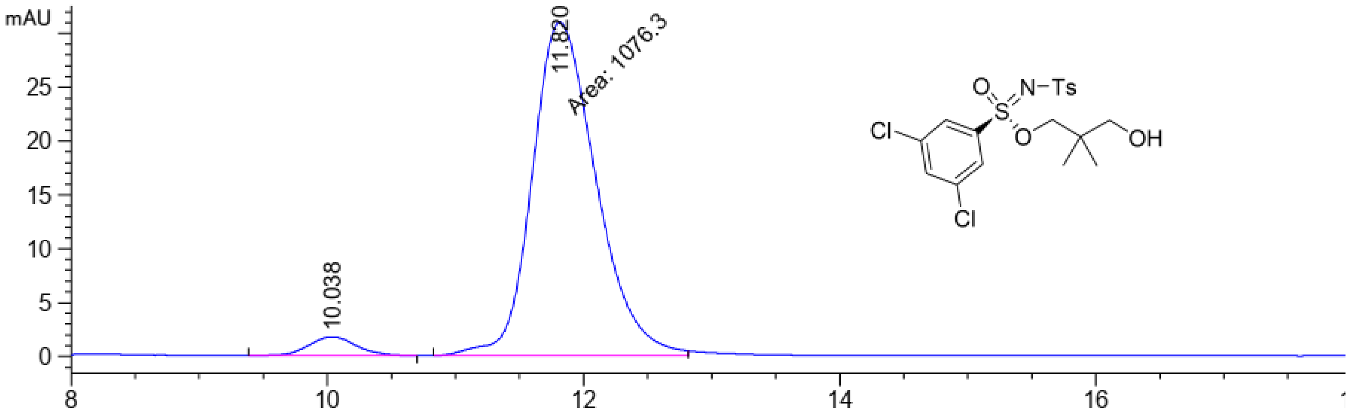

Peak #	RetTime [min]	Type	Width [min]	Area [mAU*s]	Height [mAU]	Area %
1	10.038	BB	0.3834	45.21756	1.72731	4.0043
2	11.820	BB	0.5344	1084.00012	30.96912	95.9957

(S)-3-Chloro-4-fluoro-N-tosylbenzenesulfonimidoyl chloride (rac-1r)

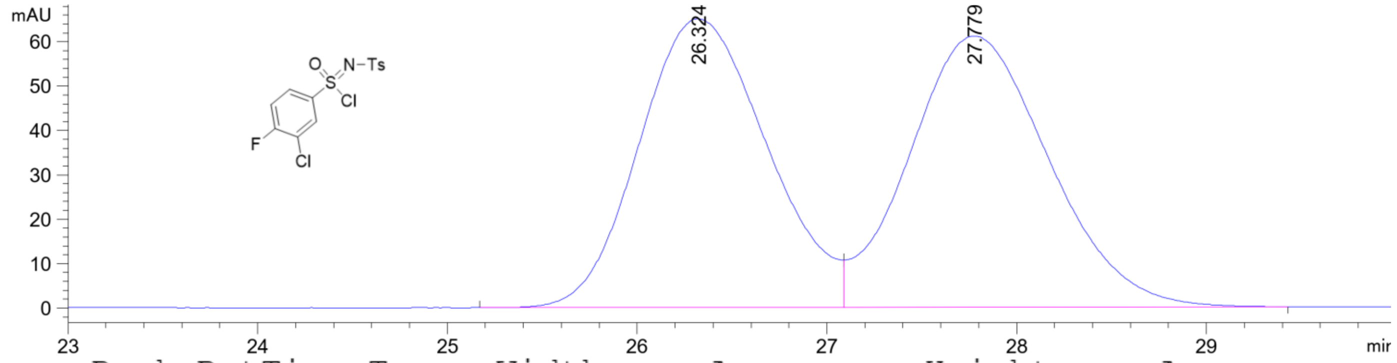

Peak #	RetTime [min]	Type	Width [min]	Area [mAU*s]	Height [mAU]	Area %
1	26.324	BV	0.7308	3048.73560	65.05097	49.3744
2	27.779	VB	0.7929	3125.99243	61.09572	50.6256

(S)-3-Chloro-4-fluoro-N-tosylbenzenesulfonimidoyl chloride (1r)

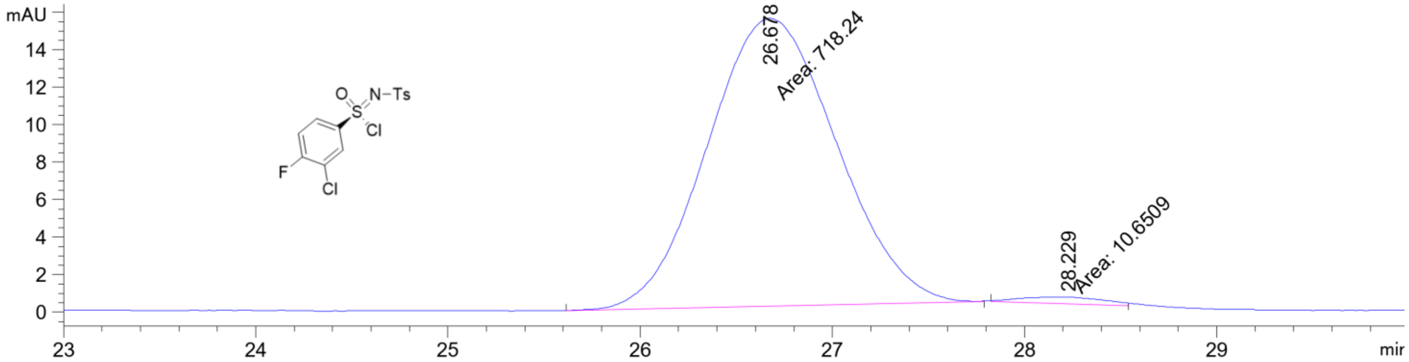

Peak #	RetTime [min]	Type	Width [min]	Area [mAU*s]	Height [mAU]	Area %
1	26.678	MM	0.7790	718.24005	15.36672	98.5388
2	28.229	MF	0.4934	10.65089	3.59751e-1	1.4612

Go to table of contents

3-Hydroxy-2,2-dimethylpropyl *(R)*-3-chloro-4-fluoro-*N*-tosylbenzenesulfonimidate (*rac*-3r)

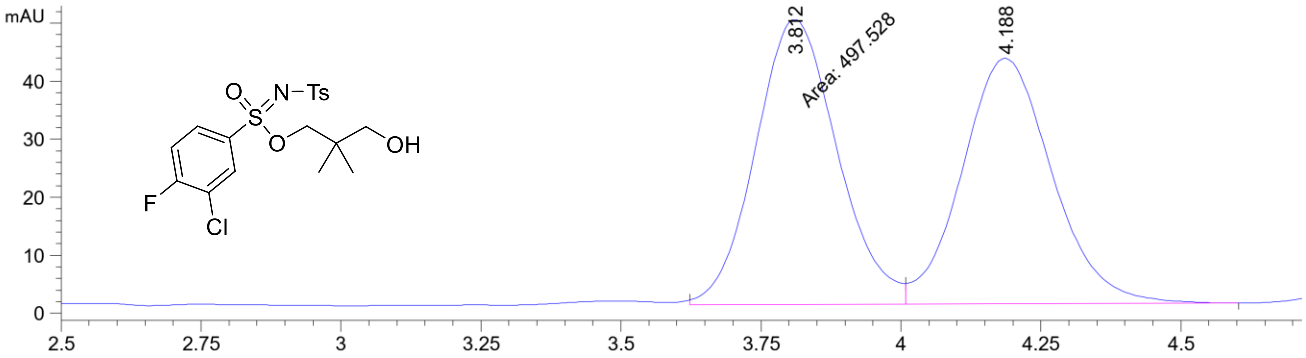

Peak #	RetTime [min]	Type	Width [min]	Area [mAU*s]	Height [mAU]	Area %
1	3.812	FM	0.1688	497.52783	49.10958	51.3034
2	4.188	VB	0.1723	472.24725	42.29914	48.6966

3-Hydroxy-2,2-dimethylpropyl *(R)*-3-chloro-4-fluoro-*N*-tosylbenzenesulfonimidate (3r)

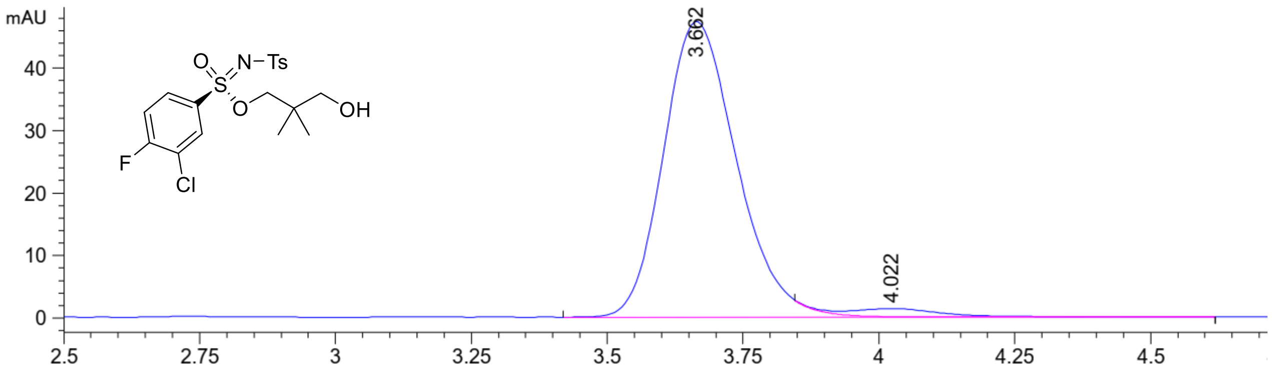

Peak #	RetTime [min]	Type	Width [min]	Area [mAU*s]	Height [mAU]	Area %
1	3.662	BV R	0.1452	442.93707	47.31397	96.6982
2	4.022	VB E	0.1709	15.12404	1.28862	3.3018

***(S)*-4-Fluoro-*N*-tosyl-3-(trifluoromethyl)benzenesulfonimidoyl chloride (*rac*-1s)**

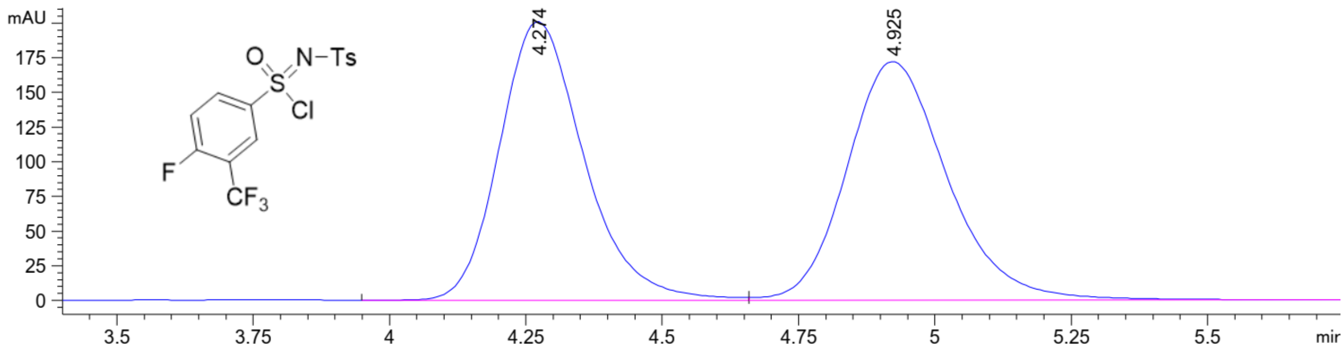

Peak #	RetTime [min]	Type	Width [min]	Area [mAU*s]	Height [mAU]	Area %
1	4.274	BV	0.1656	2162.02100	200.77869	49.3798
2	4.925	VB	0.1983	2216.33154	172.01622	50.6202

***(S)*-4-Fluoro-*N*-tosyl-3-(trifluoromethyl)benzenesulfonimidoyl chloride (1s)**

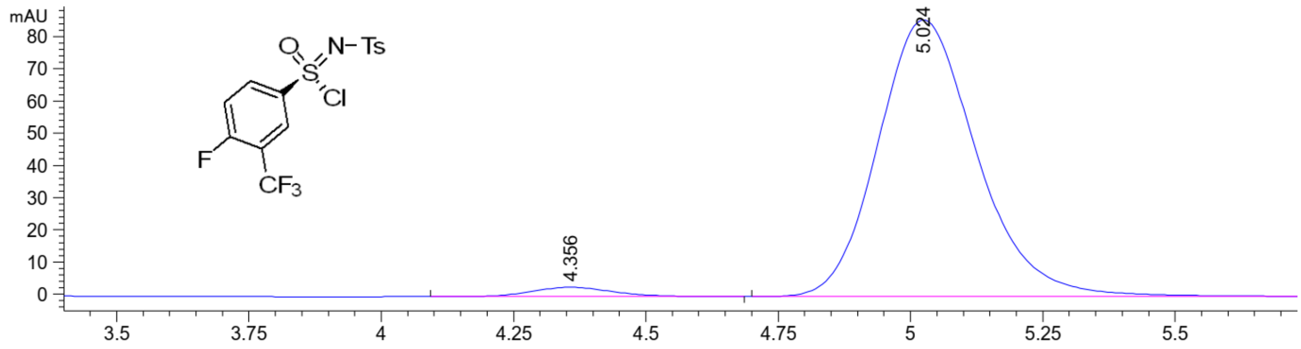

Peak #	RetTime [min]	Type	Width [min]	Area [mAU*s]	Height [mAU]	Area %
1	4.356	BB	0.1667	30.88589	2.88935	2.6893
2	5.024	BB	0.1998	1117.60632	85.89284	97.3107

3-Hydroxy-2,2-dimethylpropyl *(R)*-4-fluoro-*N*-tosyl-3-(trifluoromethyl)benzenesulfonimidate (*rac*-3s)

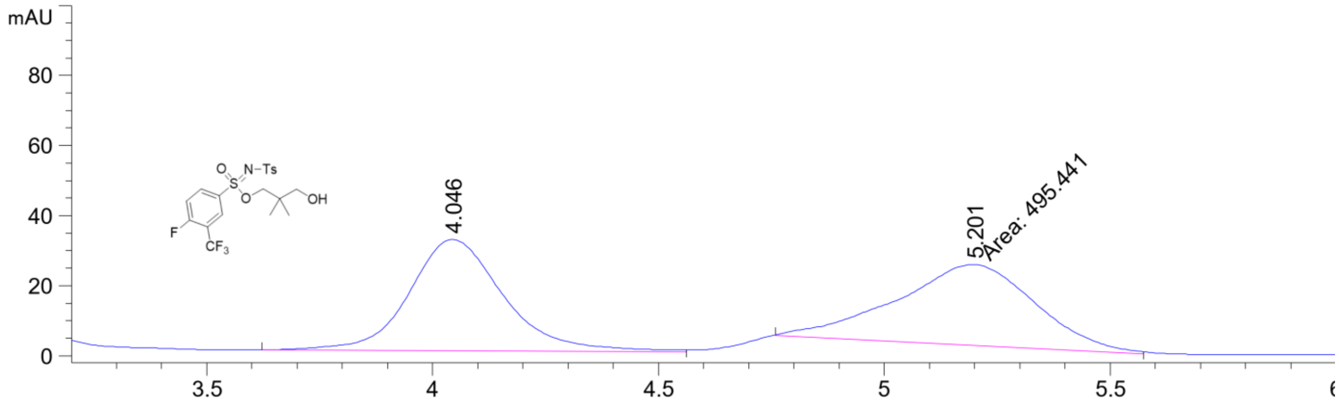

Peak #	RetTime [min]	Type	Width [min]	Area [mAU*s]	Height [mAU]	Area %
1	4.046	BV	0.2204	463.53238	31.73411	48.3363
2	5.201	MM	0.3569	495.44110	23.13847	51.6637

3-Hydroxy-2,2-dimethylpropyl *(R)*-4-fluoro-*N*-tosyl-3-(trifluoromethyl)benzenesulfonimidate (3s)

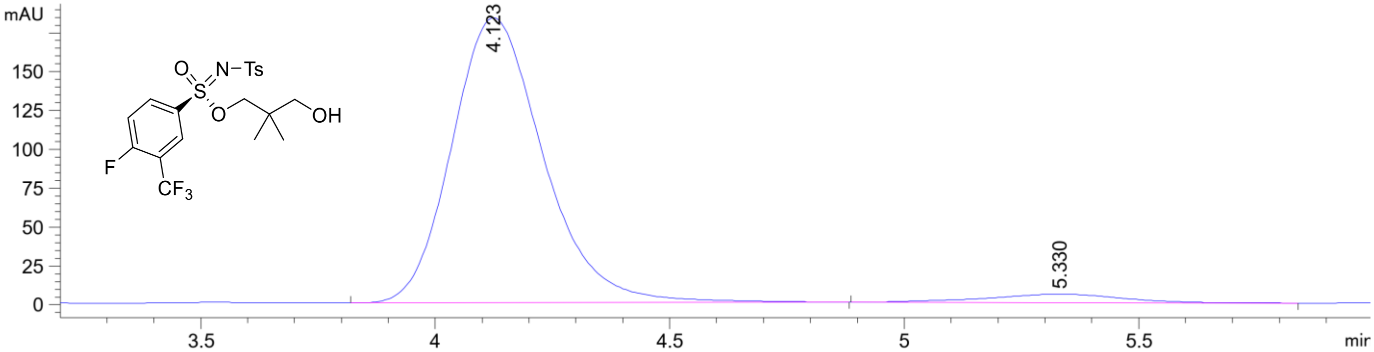

Peak #	RetTime [min]	Type	Width [min]	Area [mAU*s]	Height [mAU]	Area %
1	4.123	BB	0.2084	2491.86694	183.49062	95.8745
2	5.330	BB	0.2903	107.22580	5.38539	4.1255

(*S*)-4-Chloro-*N*-tosyl-3-(trifluoromethyl)benzenesulfonimidoyl chloride (*rac*-1t)

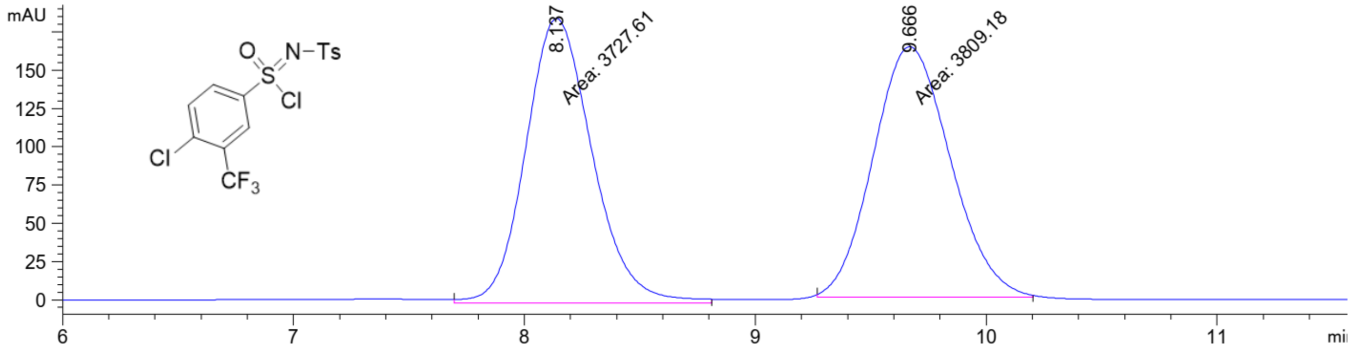

Peak #	RetTime [min]	Type	Width [min]	Area [mAU*s]	Height [mAU]	Area %
1	8.137	MM	0.3344	3727.60522	185.79988	49.4588
2	9.666	MM	0.3876	3809.17944	163.78166	50.5412

(*S*)-4-Chloro-*N*-tosyl-3-(trifluoromethyl)benzenesulfonimidoyl chloride (1t)

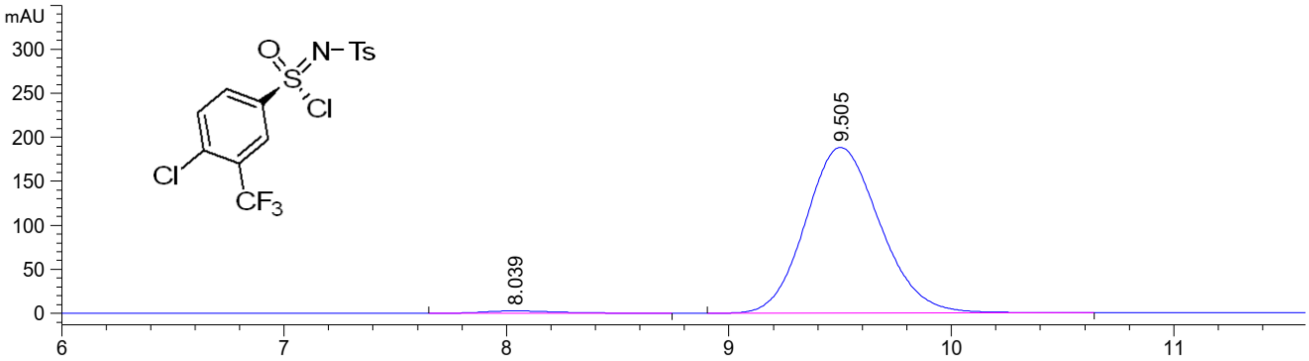

Peak #	RetTime [min]	Type	Width [min]	Area [mAU*s]	Height [mAU]	Area %
1	8.039	BB	0.3398	61.06796	2.68086	1.3769
2	9.505	BB	0.3590	4374.17969	188.30112	98.6231

Go to table of contents

3-Hydroxy-2,2-dimethylpropyl *(R)*-4-chloro-*N*-tosyl-3-(trifluoromethyl)benzenesulfonimidate (*rac*-3t)

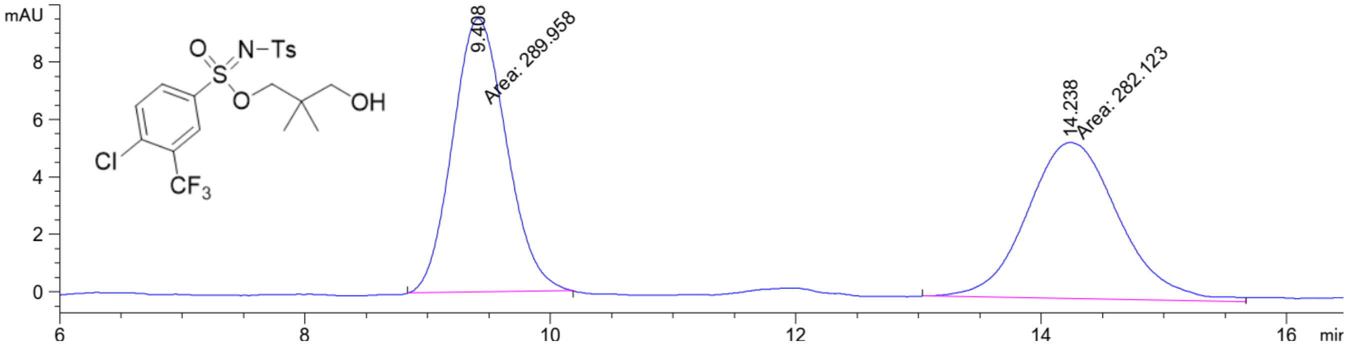

Peak #	RetTime [min]	Type	Width [min]	Area [mAU*s]	Height [mAU]	Area %
1	9.408	MM	0.5074	289.95810	9.52393	50.6848
2	14.238	MM	0.8651	282.12292	5.43540	49.3152

3-Hydroxy-2,2-dimethylpropyl *(R)*-4-chloro-*N*-tosyl-3-(trifluoromethyl)benzenesulfonimidate (3t)

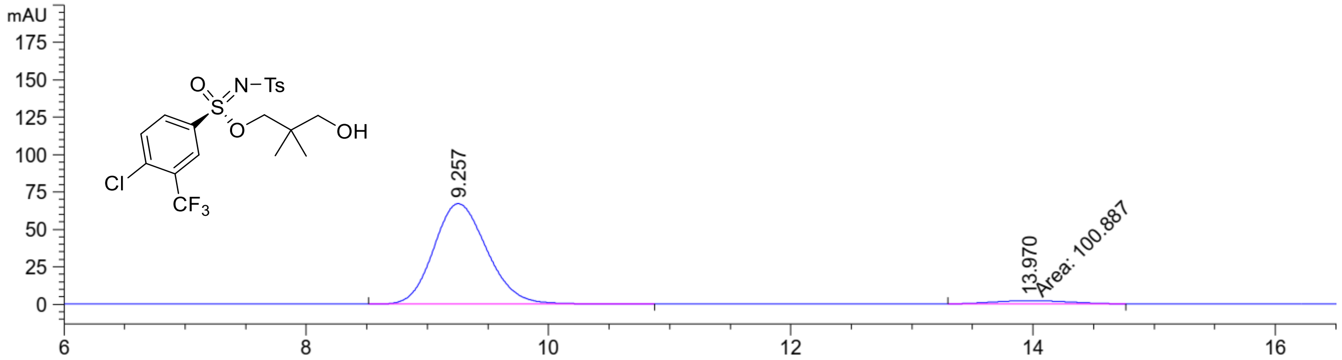

Peak #	RetTime [min]	Type	Width [min]	Area [mAU*s]	Height [mAU]	Area %
1	9.257	BB	0.4749	2052.85449	67.10452	95.3157
2	13.970	MM	0.7427	100.88711	2.26391	4.6843

(*S*)-4-Bromo-3-methyl-*N*-tosylbenzenesulfonimidoyl chloride (*rac*-1u)

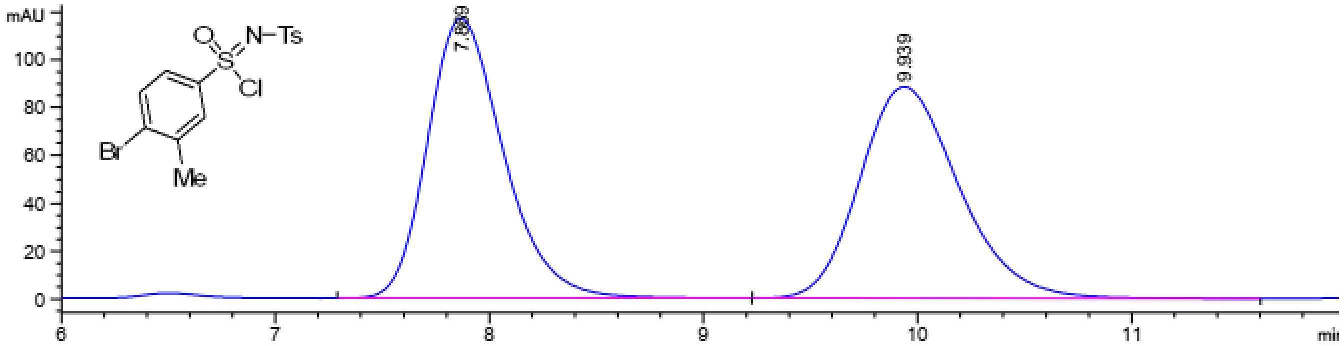

Peak #	RetTime [min]	Type	Width [min]	Area [mAU*s]	Height [mAU]	Area %
1	7.869	BB	0.3787	2898.91187	117.16417	50.3645
2	9.939	BB	0.4990	2856.95630	88.40675	49.6355

(*S*)-4-Bromo-3-methyl-*N*-tosylbenzenesulfonimidoyl chloride (1u)

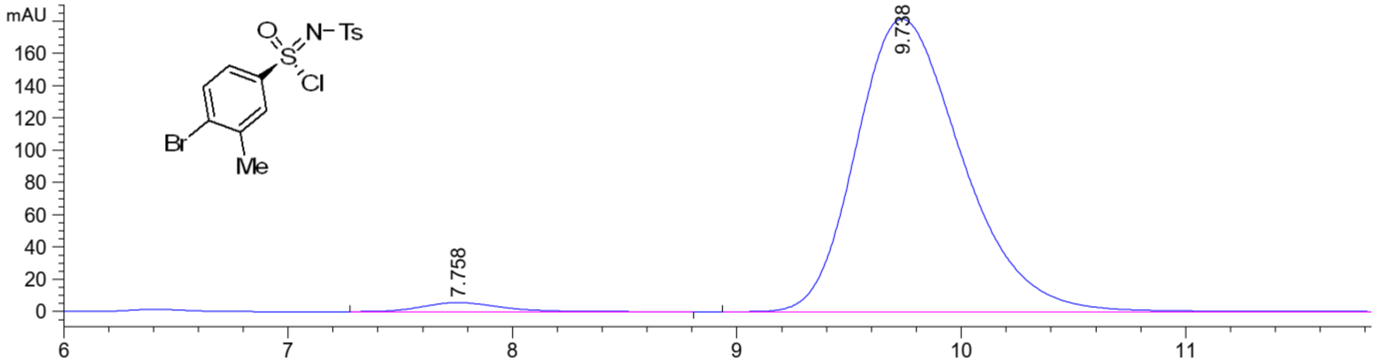

Peak #	RetTime [min]	Type	Width [min]	Area [mAU*s]	Height [mAU]	Area %
1	7.758	BB	0.3648	136.23640	5.61981	2.2288
2	9.738	BB	0.5080	5976.30713	181.55212	97.7712

3-Hydroxy-2,2-dimethylpropyl (*R*)-4-bromo-3-methyl-*N*-tosylbenzenesulfonimidate (*rac*-3u)

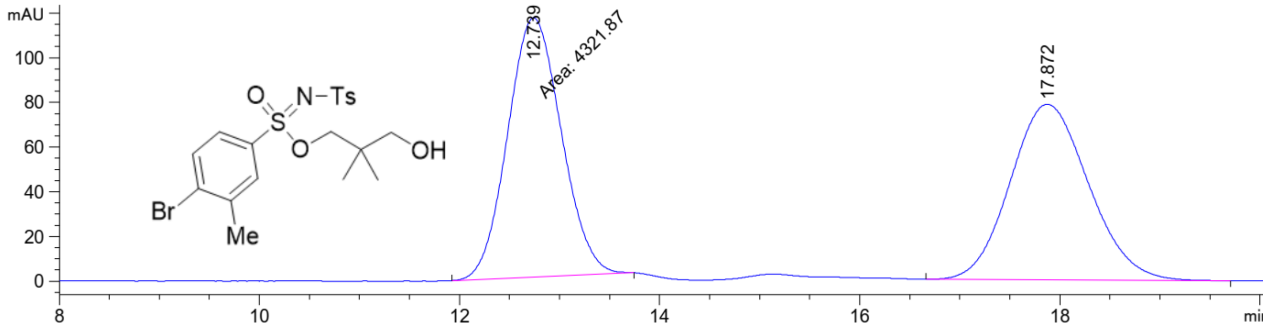

Peak #	RetTime [min]	Type	Width [min]	Area [mAU*s]	Height [mAU]	Area %
1	12.739	MM	0.6204	4321.86572	116.09524	50.1934
2	17.872	BB	0.8400	4288.55469	78.66502	49.8066

3-Hydroxy-2,2-dimethylpropyl (*R*)-4-bromo-3-methyl-*N*-tosylbenzenesulfonimidate (3u)

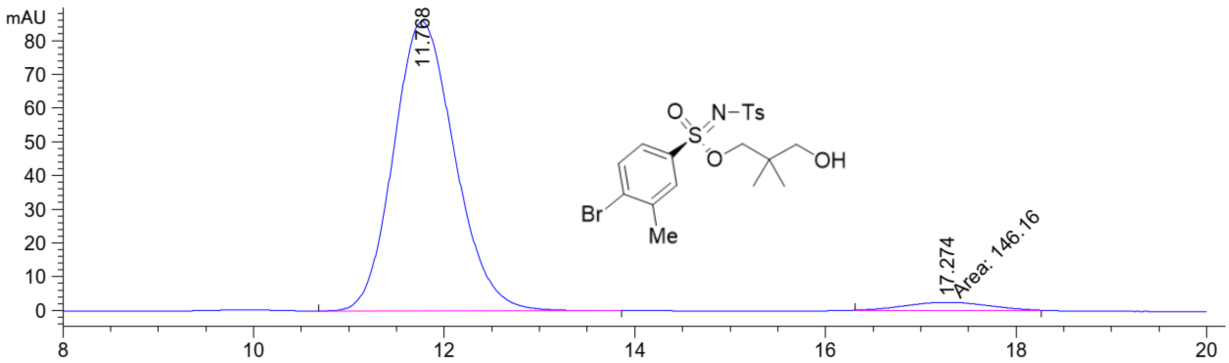

Peak #	RetTime [min]	Type	Width [min]	Area [mAU*s]	Height [mAU]	Area %
1	11.768	BB	0.6911	3831.29150	85.78227	96.3253
2	17.274	MM	1.0120	146.16031	2.40719	3.6747

Go to table of contents

(*S*)-*N*-Tosylnaphthalene-2-sulfonimidoyl chloride (1v)

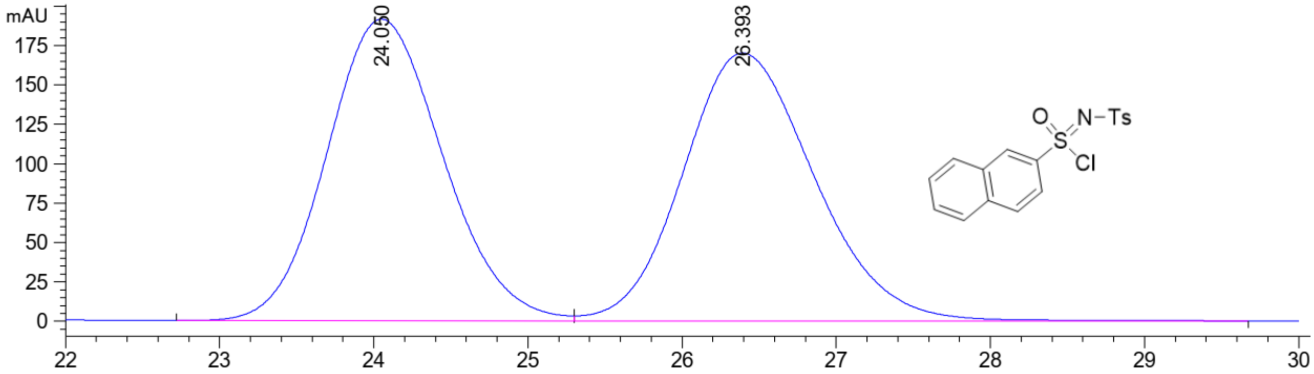

Peak #	RetTime [min]	Type	Width [min]	Area [mAU*s]	Height [mAU]	Area %
1	24.050	BV	0.8126	1.00150e4	191.34685	49.9019
2	26.393	VB	0.9102	1.00544e4	169.95276	50.0981

(*S*)-*N*-Tosylnaphthalene-2-sulfonimidoyl chloride (1v)

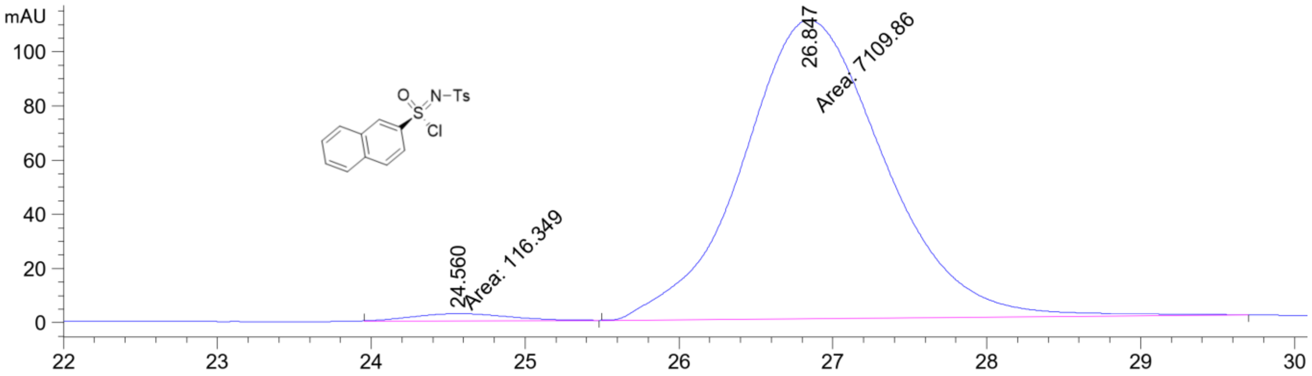

Peak #	RetTime [min]	Type	Width [min]	Area [mAU*s]	Height [mAU]	Area %
1	24.560	BB	0.5231	117.40484	2.70119	1.5986
2	26.847	BB	0.9854	7226.88135	110.45059	98.4014

Go to table of contents

3-Hydroxy-2,2-dimethylpropyl (*R*)-*N*-tosylnaphthalene-2-sulfonimidate (*rac*-3v)

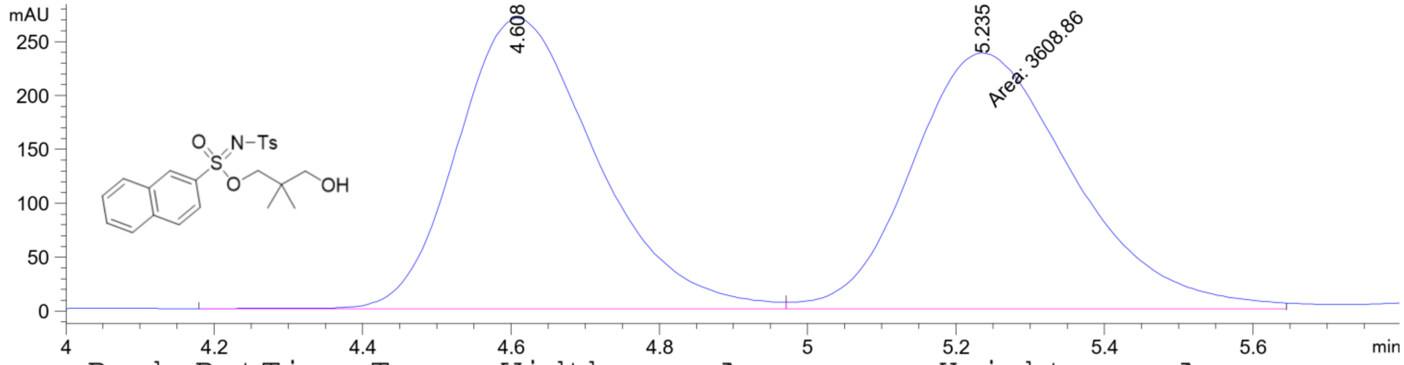

Peak #	RetTime [min]	Type	Width [min]	Area [mAU*s]	Height [mAU]	Area %
1	4.608	BV	0.2001	3514.08936	269.51578	49.3347
2	5.235	MF	0.2533	3608.86255	237.43176	50.6653

3-Hydroxy-2,2-dimethylpropyl (*R*)-*N*-tosylnaphthalene-2-sulfonimidate (3v)

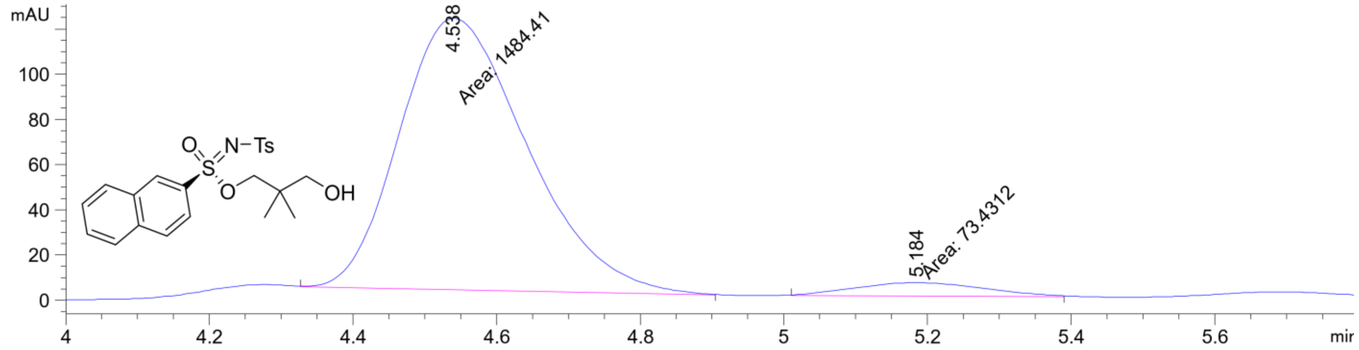

Peak #	RetTime [min]	Type	Width [min]	Area [mAU*s]	Height [mAU]	Area %
1	4.538	MM	0.2058	1484.40698	120.19198	95.2863
2	5.184	MM	0.2082	73.43118	5.87886	4.7137

(*S*)-*N*-((4-Fluorophenyl)sulfonyl)benzenesulfonimidoyl chloride (*rac*-1w)

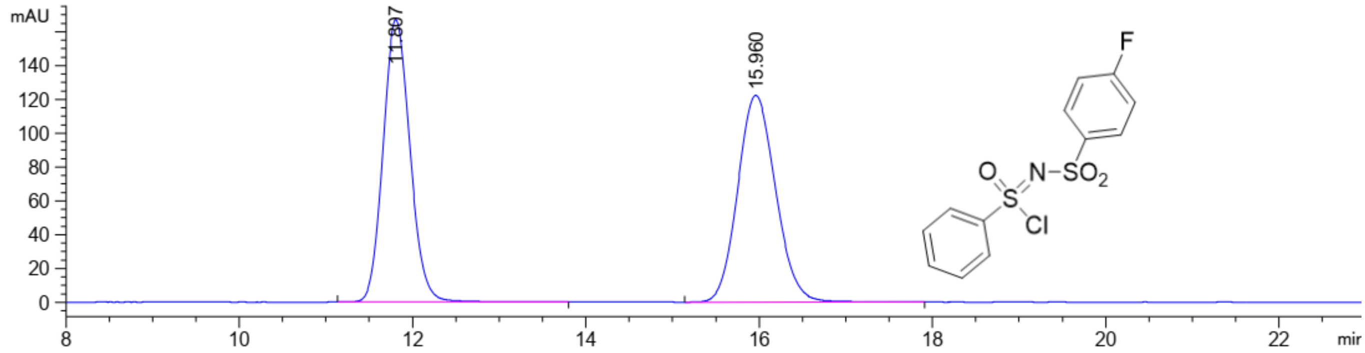

Peak #	RetTime [min]	Type	Width [min]	Area [mAU*s]	Height [mAU]	Area %
1	11.807	BB	0.3359	3606.65845	167.07021	49.2204
2	15.960	BB	0.4729	3720.91455	122.30434	50.7796

(*S*)-*N*-((4-Fluorophenyl)sulfonyl)benzenesulfonimidoyl chloride (1w)

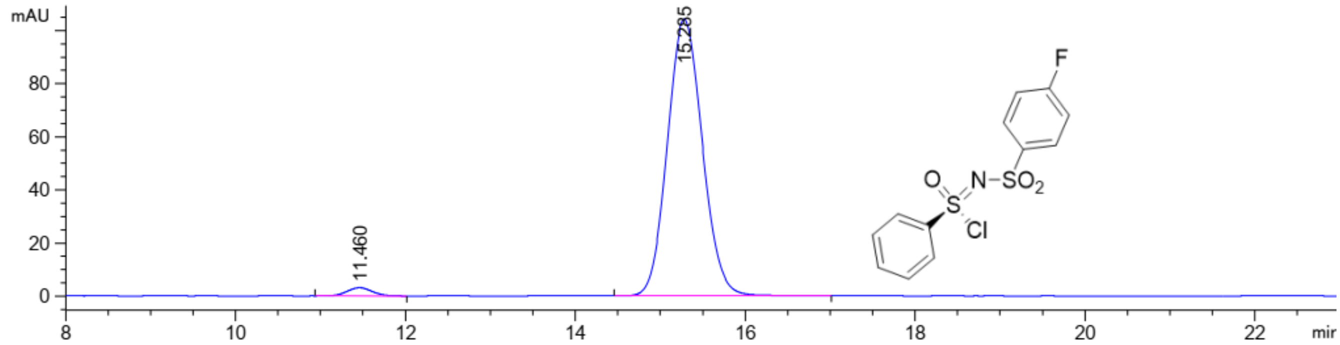

Peak #	RetTime [min]	Type	Width [min]	Area [mAU*s]	Height [mAU]	Area %
1	11.460	BB	0.3135	63.95521	3.11565	2.1062
2	15.285	BB	0.4451	2972.60352	104.12341	97.8938

3-Hydroxy-2,2-dimethylpropyl (*R*)-*N*-((4-fluorophenyl)sulfonyl)benzenesulfonimidate (*rac*-3w)

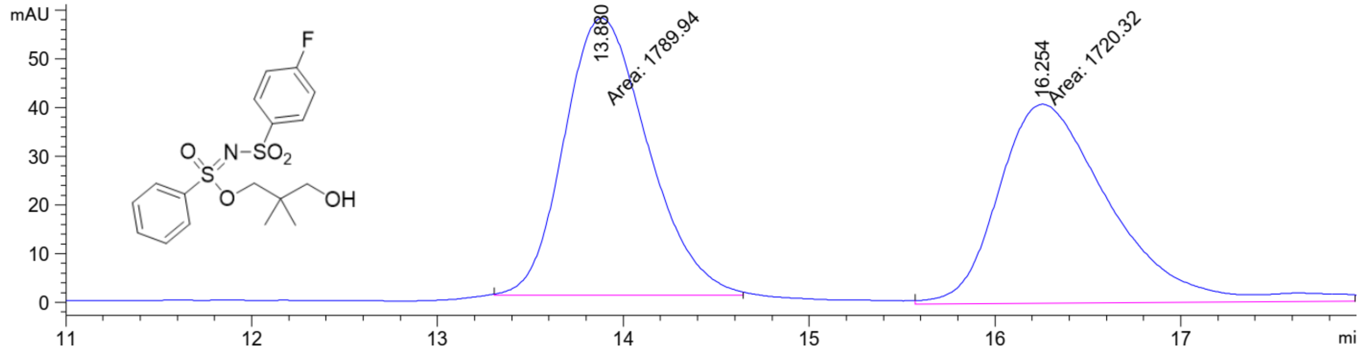

Peak #	RetTime [min]	Type	Width [min]	Area [mAU*s]	Height [mAU]	Area %
1	13.880	MM	0.5234	1789.93628	56.99667	50.9917
2	16.254	MM	0.7017	1720.31519	40.85975	49.0083

3-Hydroxy-2,2-dimethylpropyl (*R*)-*N*-((4-fluorophenyl)sulfonyl)benzenesulfonimidate (3w)

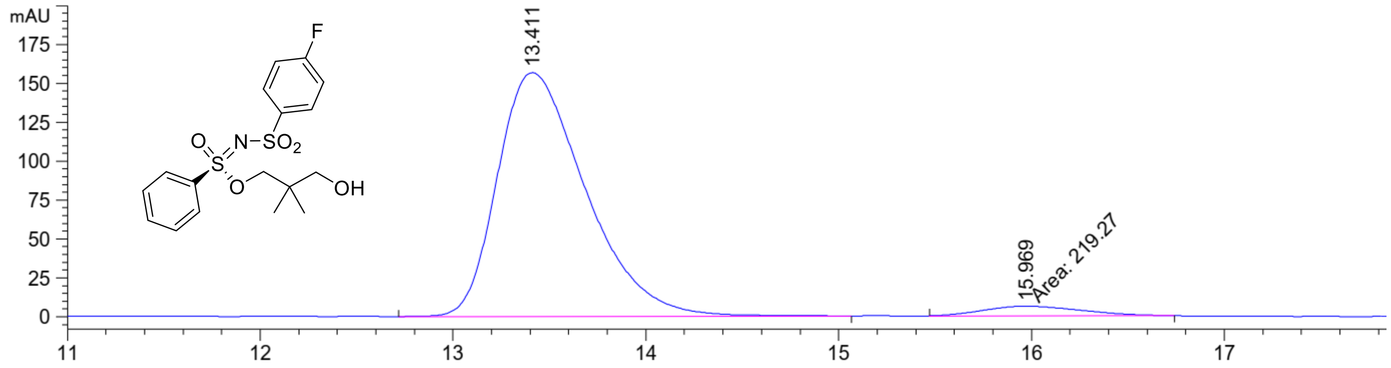

Peak #	RetTime [min]	Type	Width [min]	Area [mAU*s]	Height [mAU]	Area %
1	13.411	BB	0.4862	4967.31299	156.54079	95.7724
2	15.969	MM	0.5923	219.27007	6.16991	4.2276

Go to table of contents

(*S*)-*N*-((3-Fluorophenyl)sulfonyl)benzenesulfonimidoyl chloride (*rac*-1x)

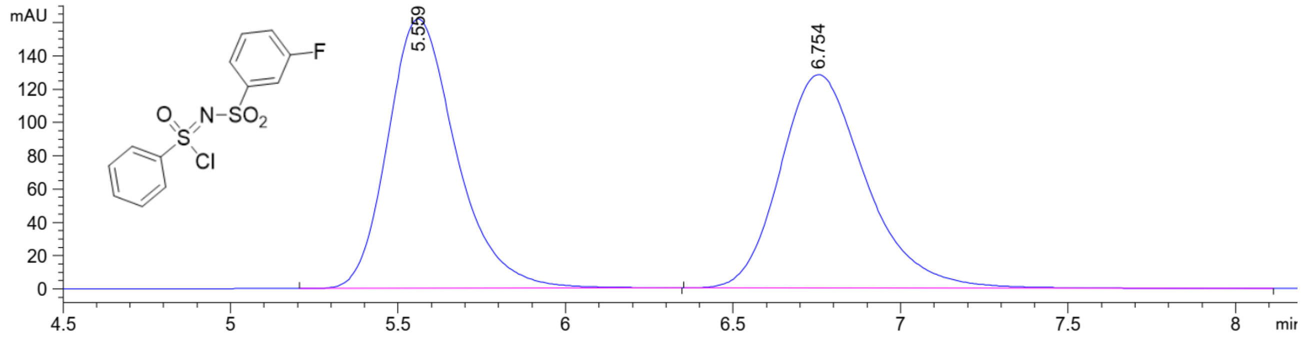

Peak #	RetTime [min]	Type	Width [min]	Area [mAU*s]	Height [mAU]	Area %
1	5.559	BB	0.2182	2307.37549	161.92833	50.2379
2	6.754	BB	0.2739	2285.52539	128.24902	49.7621

(*S*)-*N*-((3-Fluorophenyl)sulfonyl)benzenesulfonimidoyl chloride (1x)

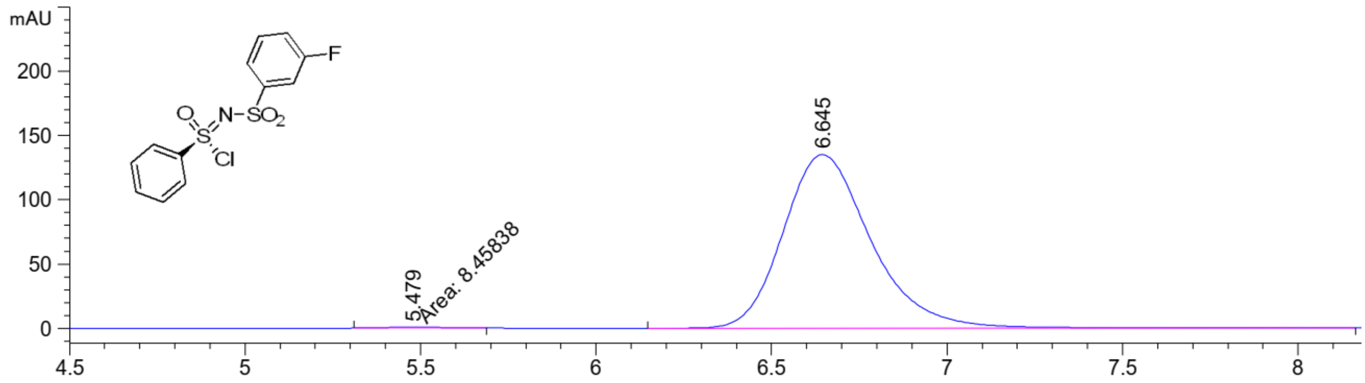

Peak #	RetTime [min]	Type	Width [min]	Area [mAU*s]	Height [mAU]	Area %
1	5.479	MM	0.1882	8.45838	7.49205e-1	0.3579
2	6.645	BB	0.2676	2355.03345	134.96587	99.6421

Go to table of contents

3-Hydroxy-2,2-dimethylpropyl (*R*)-*N*-((3-fluorophenyl)sulfonyl)benzenesulfonimidate (*rac*-3x)

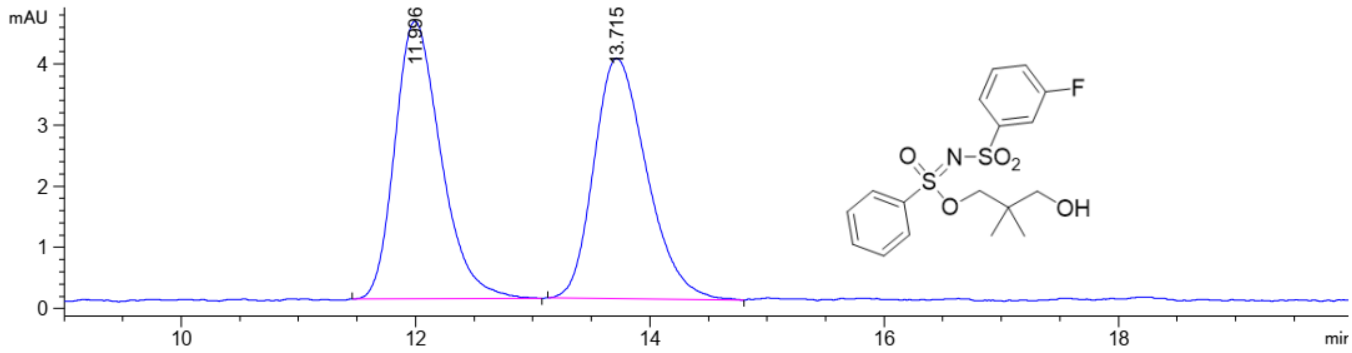

Peak #	RetTime [min]	Type	Width [min]	Area [mAU*s]	Height [mAU]	Area %
1	11.996	BB	0.4104	123.88248	4.53956	50.0909
2	13.715	BB	0.4525	123.43273	3.92889	49.9091

3-Hydroxy-2,2-dimethylpropyl (*R*)-*N*-((3-fluorophenyl)sulfonyl)benzenesulfonimidate (3x)

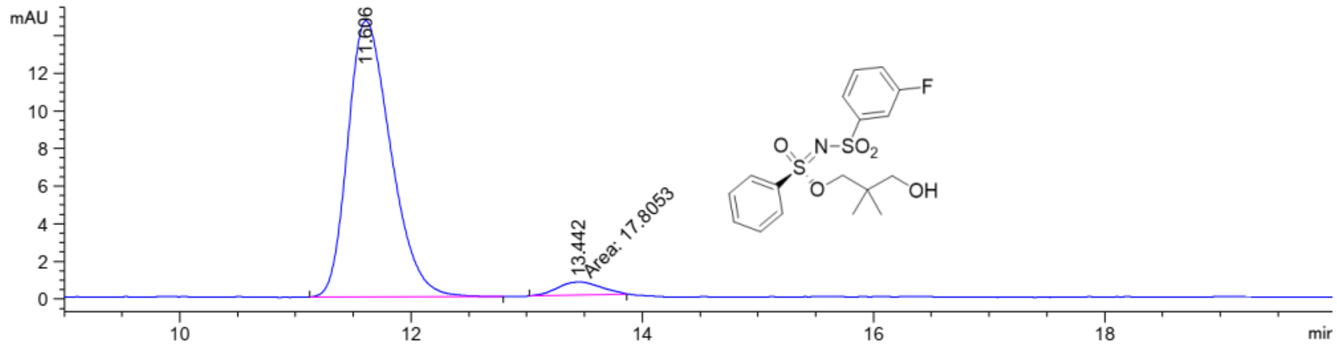

Peak #	RetTime [min]	Type	Width [min]	Area [mAU*s]	Height [mAU]	Area %
1	11.606	BB	0.3997	379.71997	14.68172	95.5210
2	13.442	MM	0.4286	17.80527	6.92345e-1	4.4790

(*S*)-*N*-((3,4-Difluorophenyl)sulfonyl)benzenesulfonimidoyl chloride (*rac*-1y)

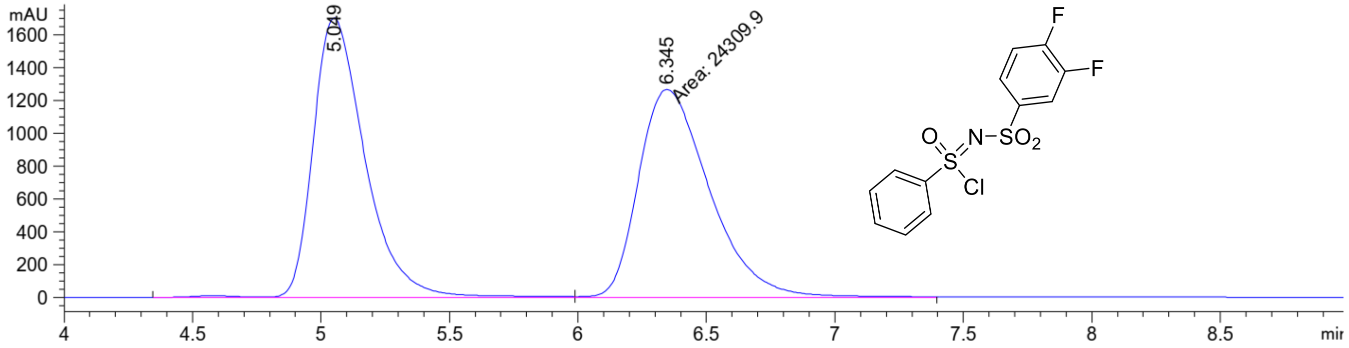

Peak #	RetTime [min]	Type	Width [min]	Area [mAU*s]	Height [mAU]	Area %
1	5.049	VV R	0.2128	2.38516e4	1701.30115	49.5243
2	6.345	MF	0.3204	2.43099e4	1264.68115	50.4757

(*S*)-*N*-((3,4-Difluorophenyl)sulfonyl)benzenesulfonimidoyl chloride (1y)

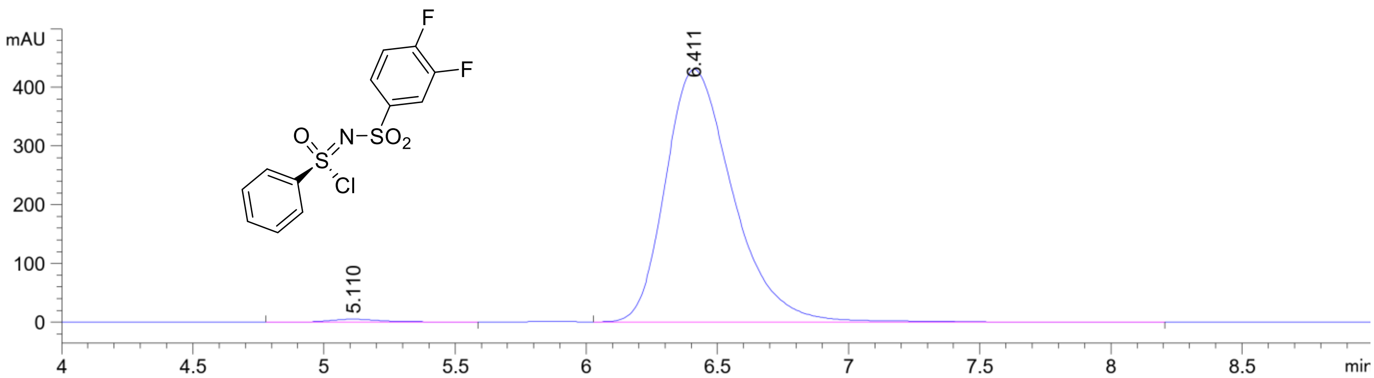

Peak #	RetTime [min]	Type	Width [min]	Area [mAU*s]	Height [mAU]	Area %
1	5.110	BB	0.1979	63.72556	4.83135	0.8252
2	6.411	BB	0.2737	7658.58057	430.28870	99.1748

3-Hydroxy-2,2-dimethylpropyl (*R*)-*N*-((3,4-difluorophenyl)sulfonyl)benzenesulfonimidate (*rac*-3y)

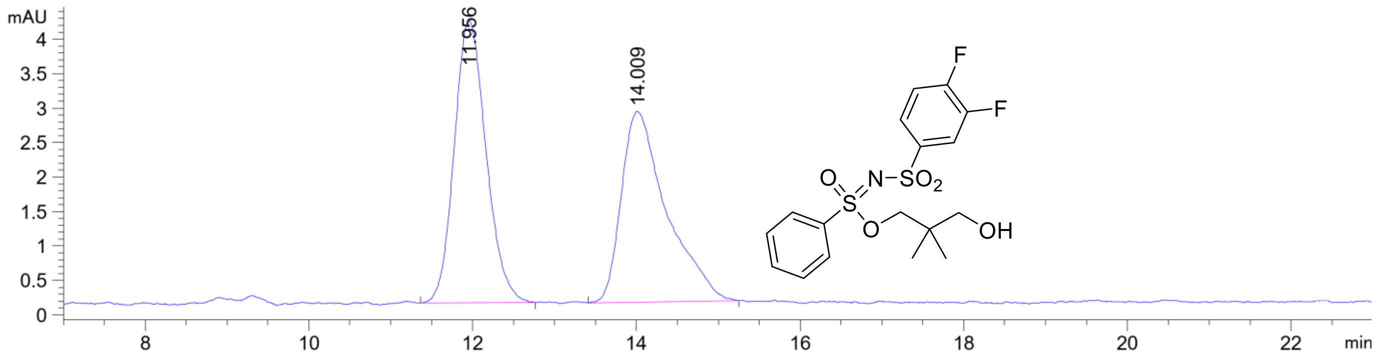

Peak #	RetTime [min]	Type	Width [min]	Area [mAU*s]	Height [mAU]	Area %
1	11.956	BB	0.4059	110.86021	4.09363	51.0256
2	14.009	BB	0.5348	106.40389	2.76577	48.9744

3-Hydroxy-2,2-dimethylpropyl (*R*)-*N*-((3,4-difluorophenyl)sulfonyl)benzenesulfonimidate (3y)

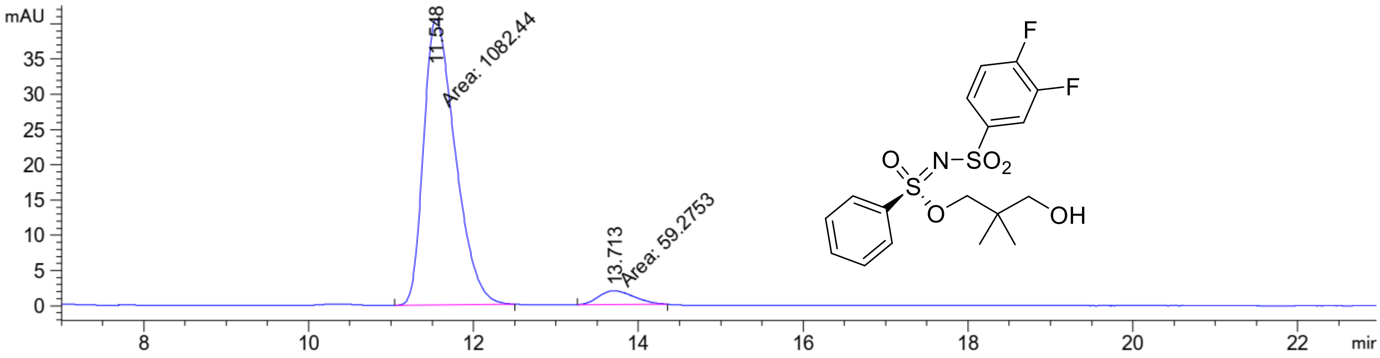

Peak #	RetTime [min]	Type	Width [min]	Area [mAU*s]	Height [mAU]	Area %
1	11.548	MM	0.4450	1082.43884	40.54405	94.8082
2	13.713	MM	0.5014	59.27535	1.97046	5.1918

(*S*)-*N*-((4-Bromophenyl)sulfonyl)benzenesulfonimidoyl chloride (*rac*-1z)

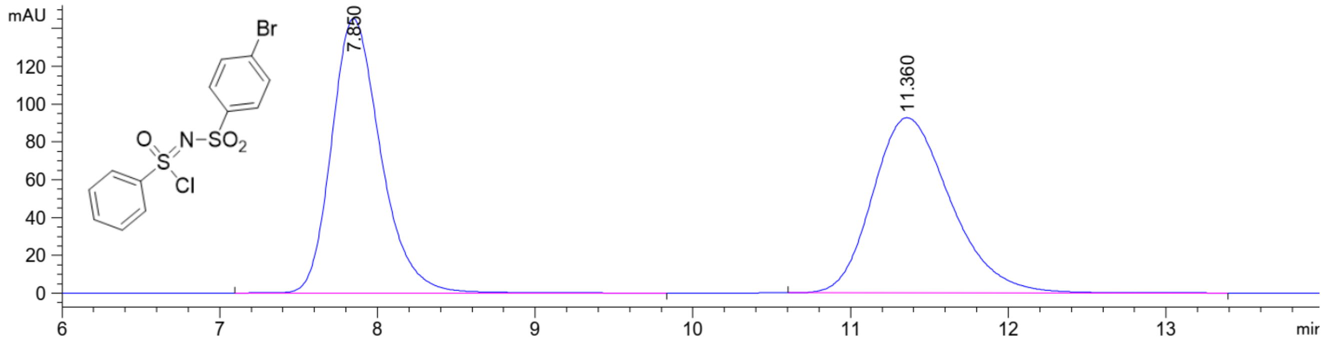

Peak #	RetTime [min]	Type	Width [min]	Area [mAU*s]	Height [mAU]	Area %
1	7.850	BB	0.3323	3137.81738	145.07175	49.6469
2	11.360	BB	0.5309	3182.44531	92.61087	50.3531

(*S*)-*N*-((4-Bromophenyl)sulfonyl)benzenesulfonimidoyl chloride (1z)

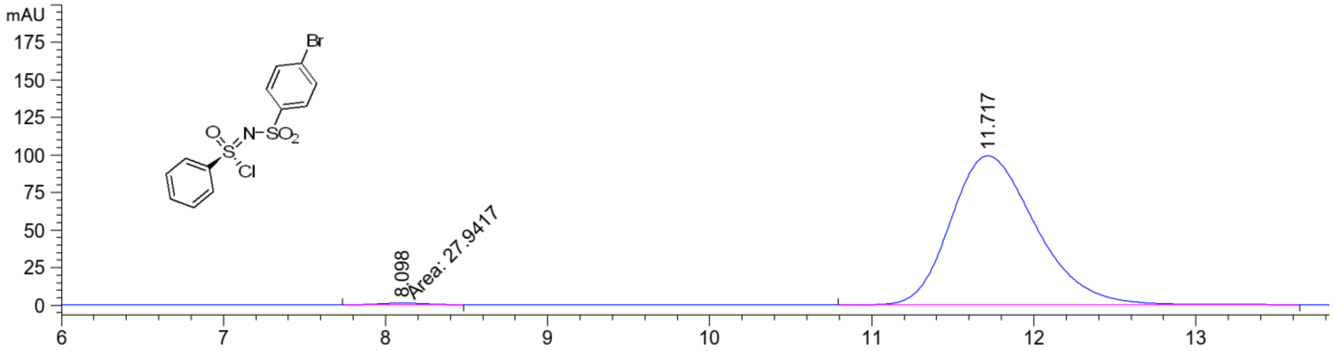

Peak #	RetTime [min]	Type	Width [min]	Area [mAU*s]	Height [mAU]	Area %
1	8.098	MM	0.3613	27.94170	1.28902	0.7672
2	11.717	BB	0.5638	3614.10132	99.02023	99.2328

3-Hydroxy-2,2-dimethylpropyl (*R*)-*N*-((4-bromophenyl)sulfonyl)benzenesulfonimidate (*rac*-3z)

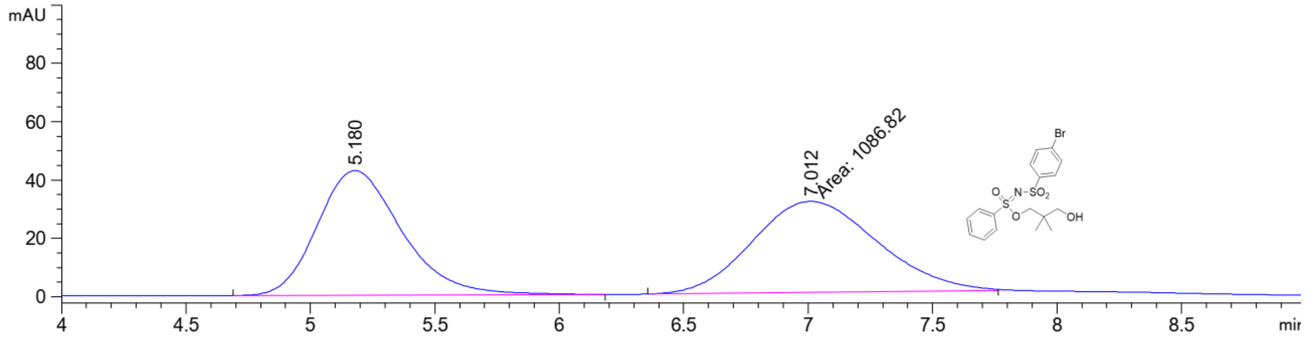

Peak #	RetTime [min]	Type	Width [min]	Area [mAU*s]	Height [mAU]	Area %
1	5.180	BB	0.3615	1008.95355	42.72131	48.1424
2	7.012	MM	0.5815	1086.81628	31.14883	51.8576

3-Hydroxy-2,2-dimethylpropyl (*R*)-*N*-((4-bromophenyl)sulfonyl)benzenesulfonimidate (3z)

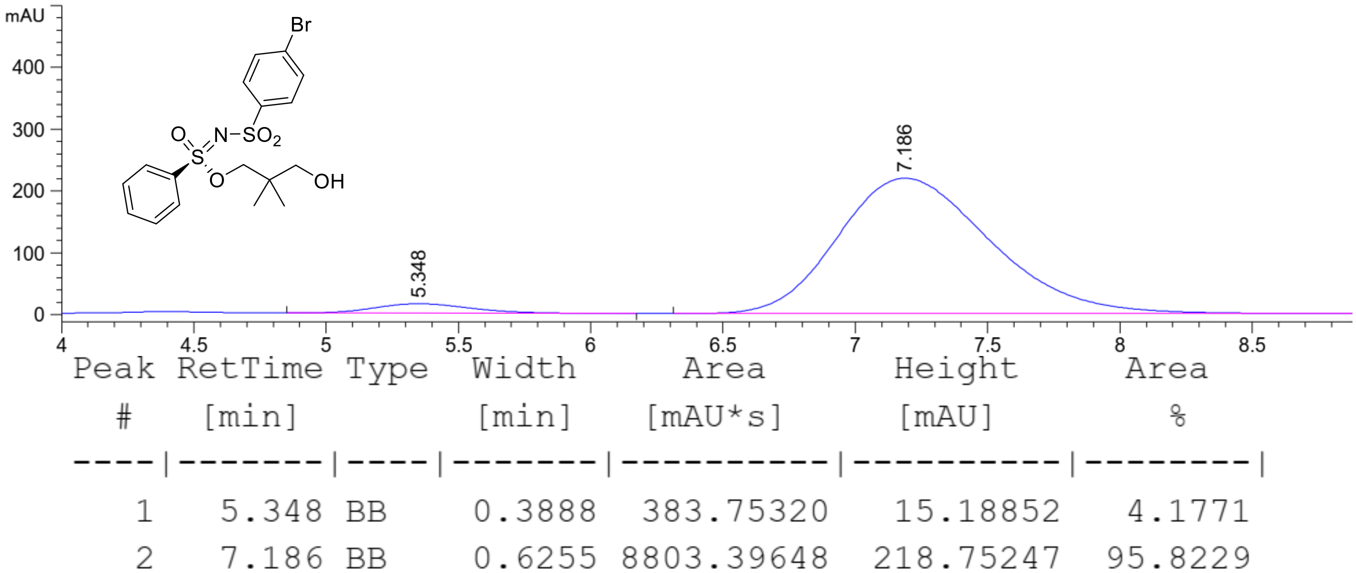

Peak #	RetTime [min]	Type	Width [min]	Area [mAU*s]	Height [mAU]	Area %
1	5.348	BB	0.3888	383.75320	15.18852	4.1771
2	7.186	BB	0.6255	8803.39648	218.75247	95.8229

(*S*)-*N*-((3-(Trifluoromethyl)phenyl)sulfonyl)benzenesulfonimidoyl chloride (*rac*-1aa)

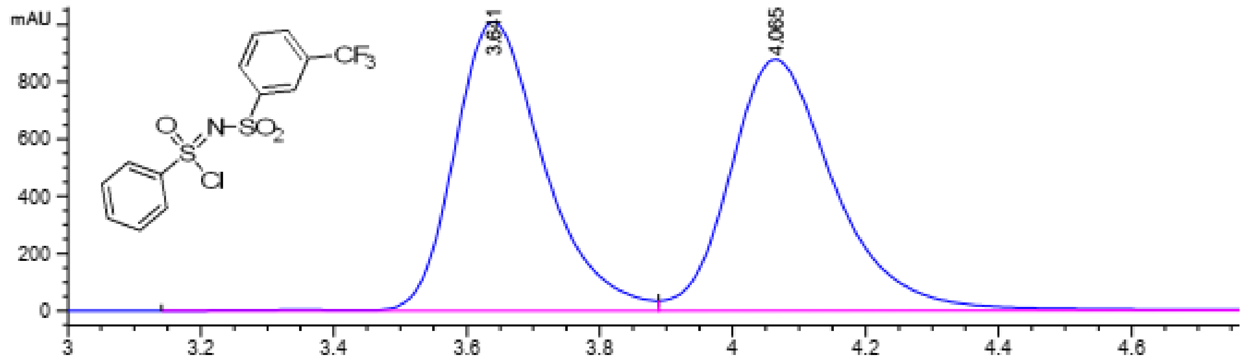

Peak #	RetTime [min]	Type	Width [min]	Area [mAU*s]	Height [mAU]	Area %
1	3.641	VV R	0.1424	9415.42871	1010.92987	49.3382
2	4.065	VB	0.1685	9668.02539	877.95215	50.6618

(*S*)-*N*-((3-(Trifluoromethyl)phenyl)sulfonyl)benzenesulfonimidoyl chloride (1aa)

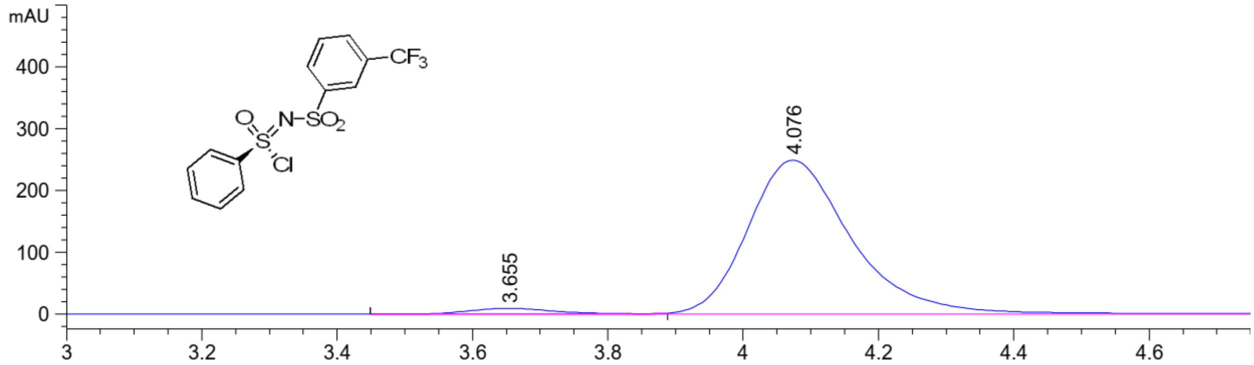

Peak #	RetTime [min]	Type	Width [min]	Area [mAU*s]	Height [mAU]	Area %
1	3.655	BV E	0.1367	82.95789	9.24433	3.0126
2	4.076	VB R	0.1632	2670.74976	248.83681	96.9874

3-Hydroxy-2,2-dimethylpropyl (*R*)-*N*-((3-(trifluoromethyl)phenyl)sulfonyl)benzenesulfonimidate (*rac*-3aa)

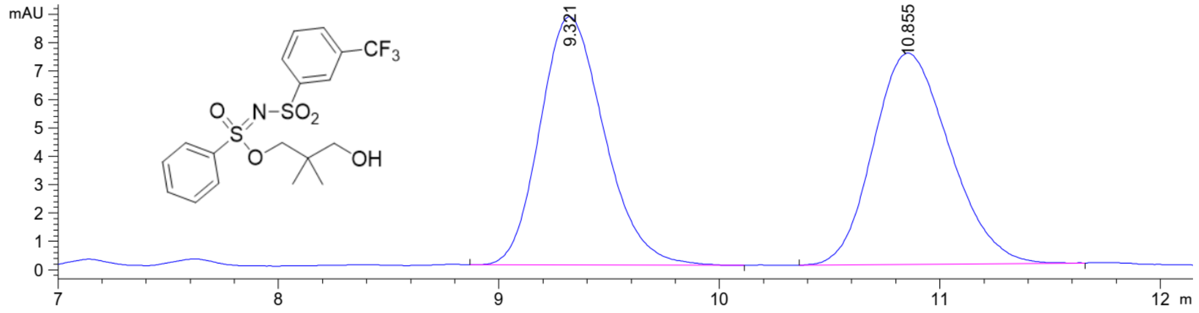

Peak #	RetTime [min]	Type	Width [min]	Area [mAU*s]	Height [mAU]	Area %
1	9.321	BB	0.3107	177.55840	8.75347	49.7069
2	10.855	BB	0.3753	179.65236	7.45015	50.2931

3-Hydroxy-2,2-dimethylpropyl (*R*)-*N*-((3-(trifluoromethyl)phenyl)sulfonyl)benzenesulfonimidate (3aa)

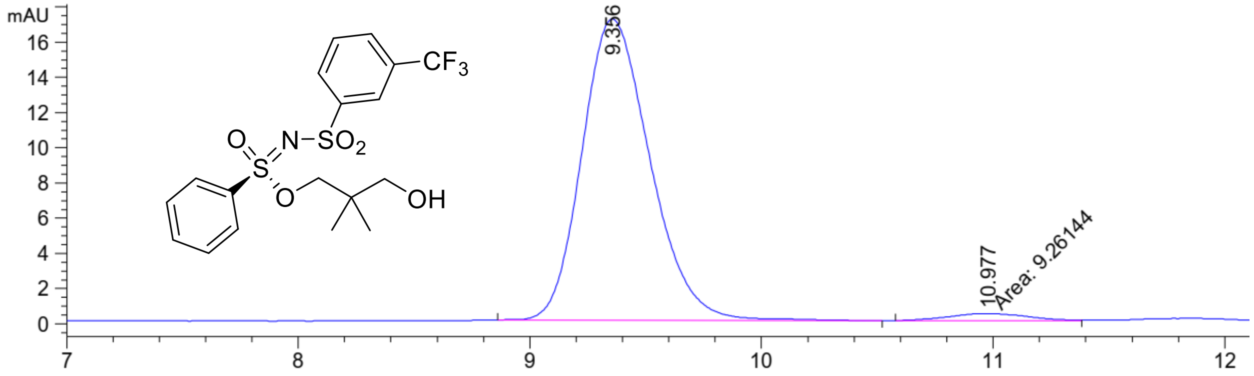

Peak #	RetTime [min]	Type	Width [min]	Area [mAU*s]	Height [mAU]	Area %
1	9.356	BB	0.3186	349.85828	17.10761	97.4211
2	10.977	MM	0.3884	9.26144	3.97466e-1	2.5789

(*S*)-*N*-((3-Chloro-4-methoxyphenyl)sulfonyl)benzenesulfonimidoyl chloride (*rac*-1ab)

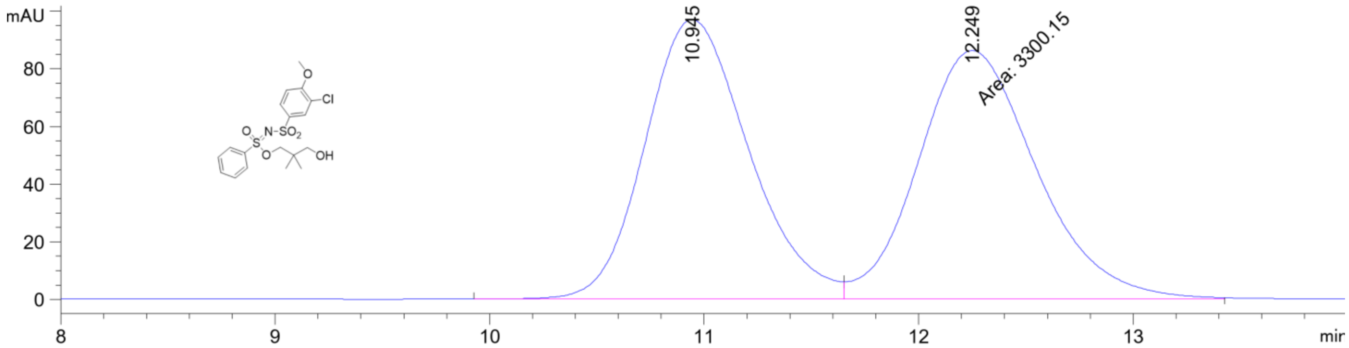

Peak #	RetTime [min]	Type	Width [min]	Area [mAU*s]	Height [mAU]	Area %
1	10.945	BV	0.5186	3263.17212	96.97520	49.7183
2	12.249	MF	0.6382	3300.14575	86.17857	50.2817

(*S*)-*N*-((3-Chloro-4-methoxyphenyl)sulfonyl)benzenesulfonimidoyl chloride (1ab)

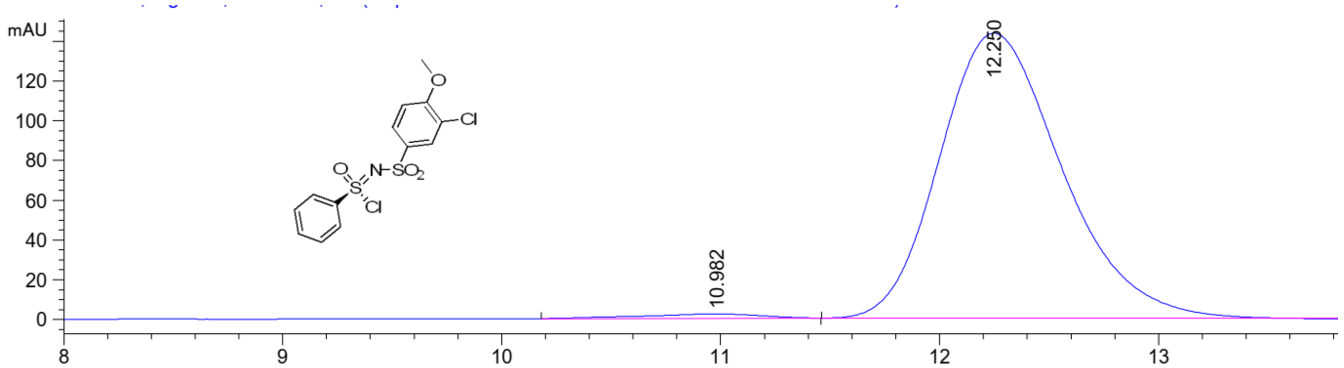

Peak #	RetTime [min]	Type	Width [min]	Area [mAU*s]	Height [mAU]	Area %
1	10.982	BB	0.5032	82.93223	2.20315	1.4661
2	12.250	BB	0.6015	5573.78711	143.40225	98.5339

Go to table of contents

3-Hydroxy-2,2-dimethylpropyl (*R*)-*N*-((3-chloro-4-methoxyphenyl)sulfonyl)benzenesulfonimidate (*rac*-3ab)

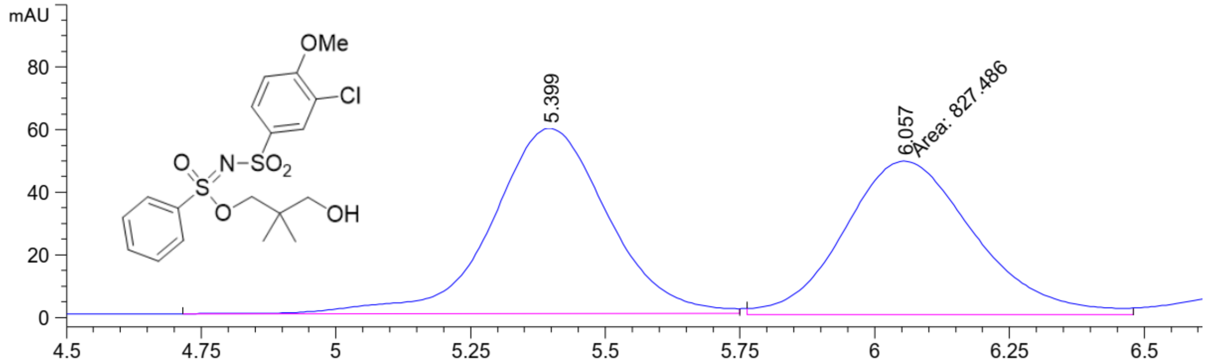

Peak #	RetTime [min]	Type	Width [min]	Area [mAU*s]	Height [mAU]	Area %
1	5.399	BV	0.2272	890.31128	59.28403	51.8287
2	6.057	MM	0.2815	827.48627	48.98494	48.1713

3-Hydroxy-2,2-dimethylpropyl (*R*)-*N*-((3-chloro-4-methoxyphenyl)sulfonyl)benzenesulfonimidate (3ab)

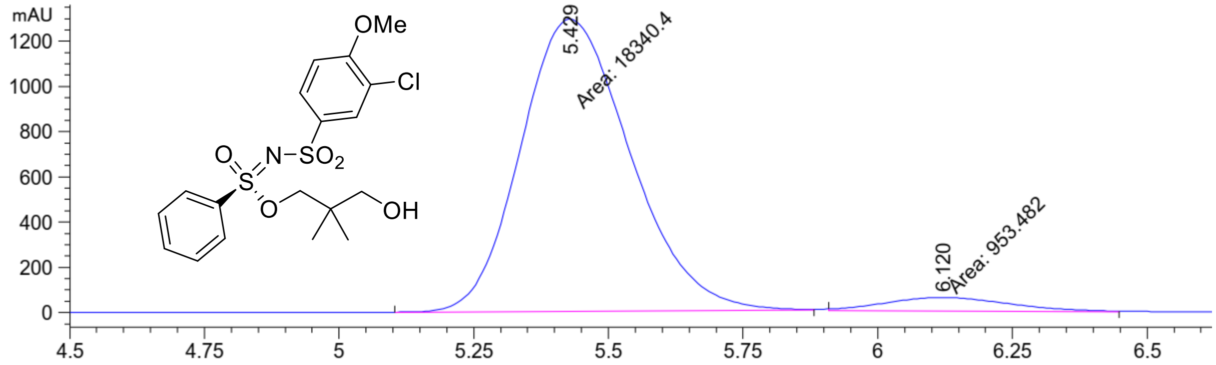

Peak #	RetTime [min]	Type	Width [min]	Area [mAU*s]	Height [mAU]	Area %
1	5.429	MM	0.2361	1.83404e4	1294.50684	95.0581
2	6.120	MM	0.2669	953.48157	59.53228	4.9419

Go to table of contents

(*S*)-*N*-(Cyclopropylsulfonyl)benzenesulfonimidoyl chloride (*rac*-1ac)

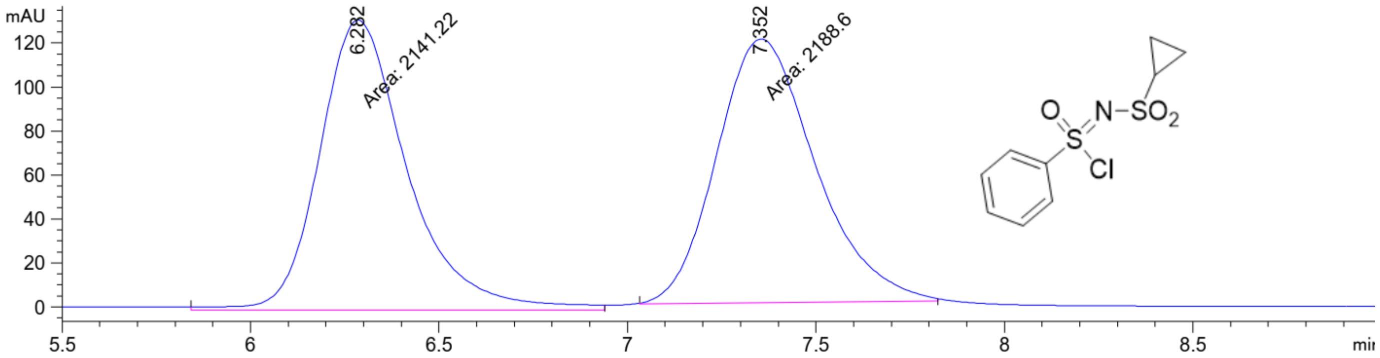

Peak #	RetTime [min]	Type	Width [min]	Area [mAU*s]	Height [mAU]	Area %
1	6.282	MM	0.2708	2141.21948	131.78722	49.4529
2	7.352	MM	0.3038	2188.59839	120.06880	50.5471

(*S*)-*N*-(Cyclopropylsulfonyl)benzenesulfonimidoyl chloride (1ac)

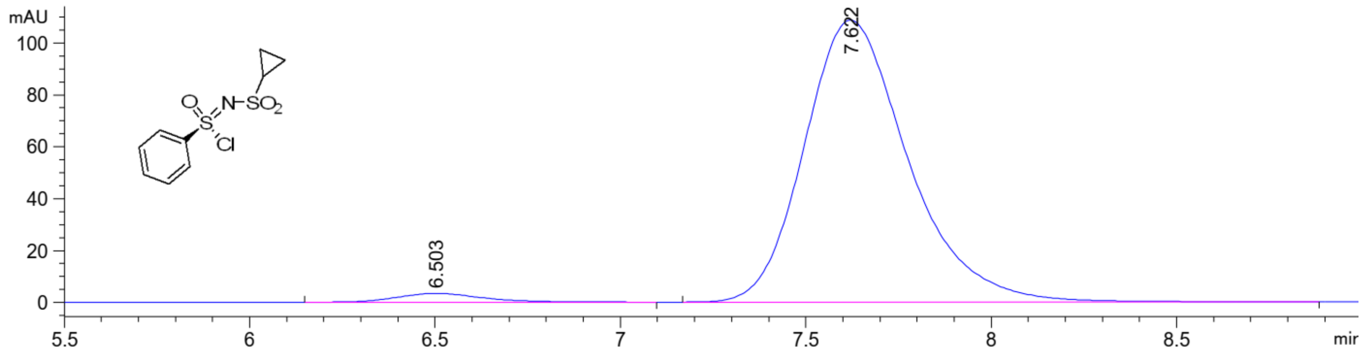

Peak #	RetTime [min]	Type	Width [min]	Area [mAU*s]	Height [mAU]	Area %
1	6.503	BB	0.2434	54.42023	3.38839	2.5094
2	7.622	BB	0.2971	2114.21533	108.59925	97.4906

3-Hydroxy-2,2-dimethylpropyl (*R*)-*N*-(cyclopropylsulfonyl)benzenesulfonimidate (*rac*-3ac)

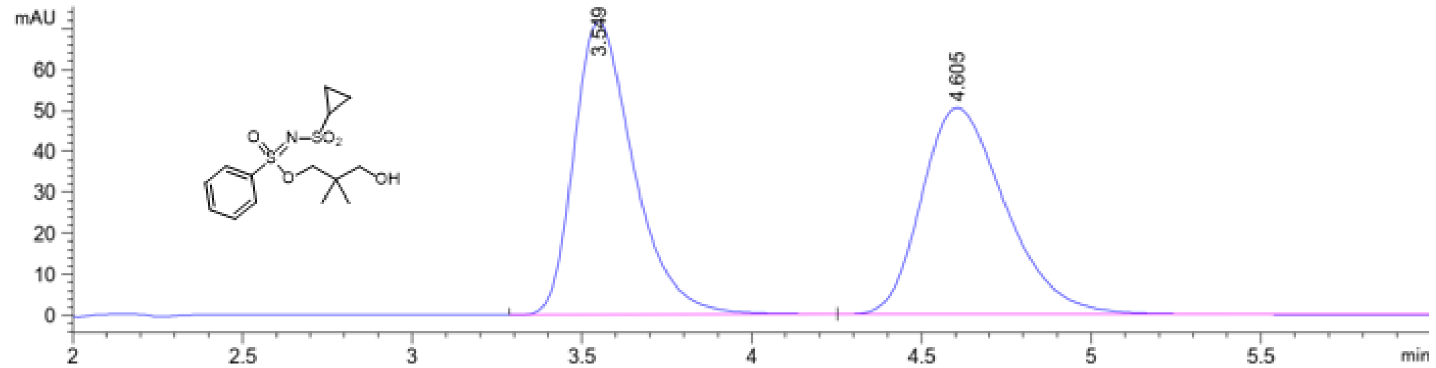

Peak #	RetTime [min]	Type	Width [min]	Area [mAU*s]	Height [mAU]	Area %
1	3.549	BB	0.1864	875.97925	71.75061	50.1489
2	4.605	BB	0.2676	870.77734	50.38012	49.8511

3-Hydroxy-2,2-dimethylpropyl (*R*)-*N*-(cyclopropylsulfonyl)benzenesulfonimidate (3ac)

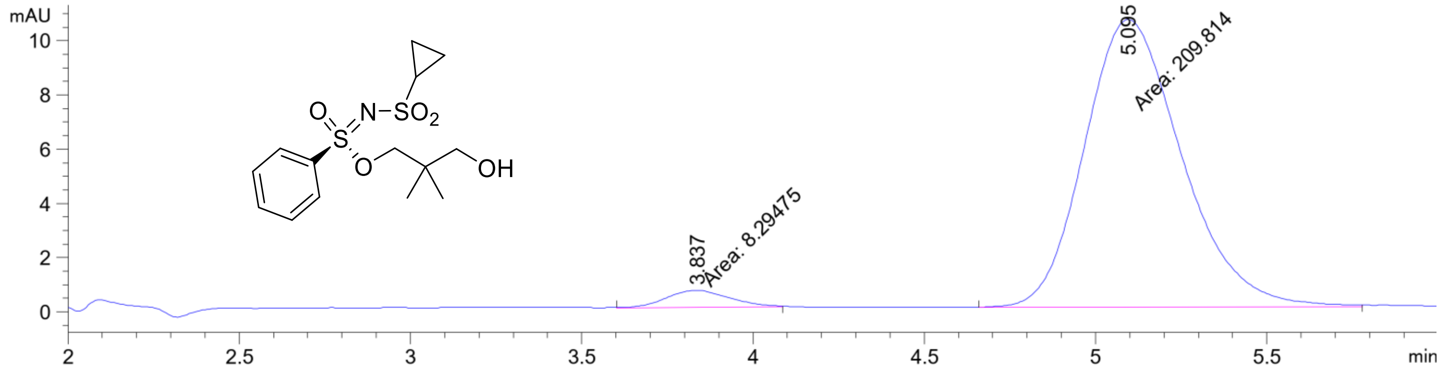

Peak #	RetTime [min]	Type	Width [min]	Area [mAU*s]	Height [mAU]	Area %
1	3.837	MM	0.2161	8.29475	6.39763e-1	3.8030
2	5.095	MF	0.3294	209.81354	10.61726	96.1970

Go to table of contents

(*R*)-*N*-Tosylbenzenesulfonimidoyl fluoride (*rac*-4)

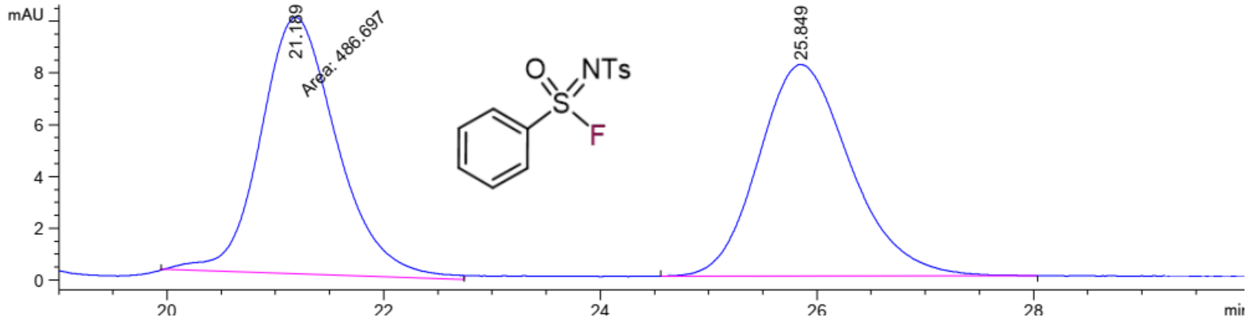

Peak #	RetTime [min]	Type	Width [min]	Area [mAU*s]	Height [mAU]	Area %
1	21.189	MM	0.8196	486.69650	9.89645	50.1647
2	25.849	BB	0.8156	483.50085	8.17984	49.8353

(*R*)-*N*-Tosylbenzenesulfonimidoyl fluoride (4)

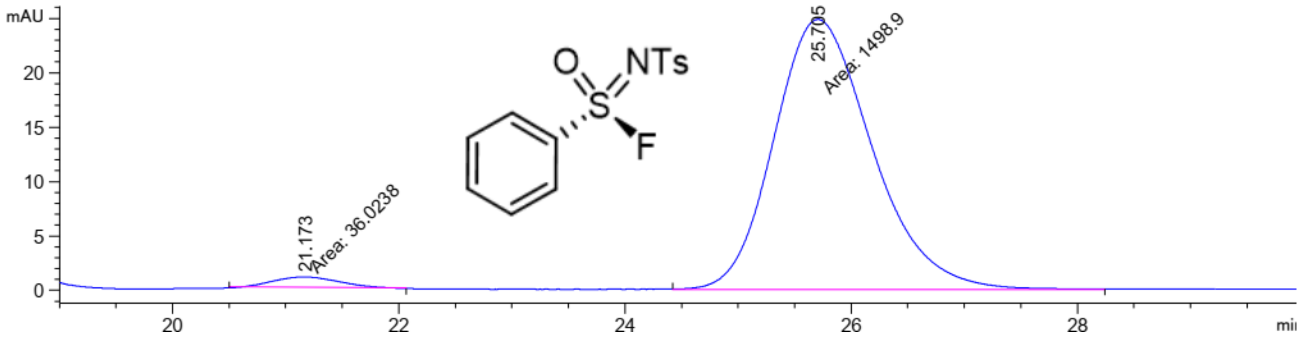

Peak #	RetTime [min]	Type	Width [min]	Area [mAU*s]	Height [mAU]	Area %
1	21.173	MM	0.6516	36.02376	9.21480e-1	2.3469
2	25.705	MM	1.0060	1498.90002	24.83199	97.6531

(*R*)-*N*'-Tosylbenzenesulfonimidoyl azide (*rac*-5)

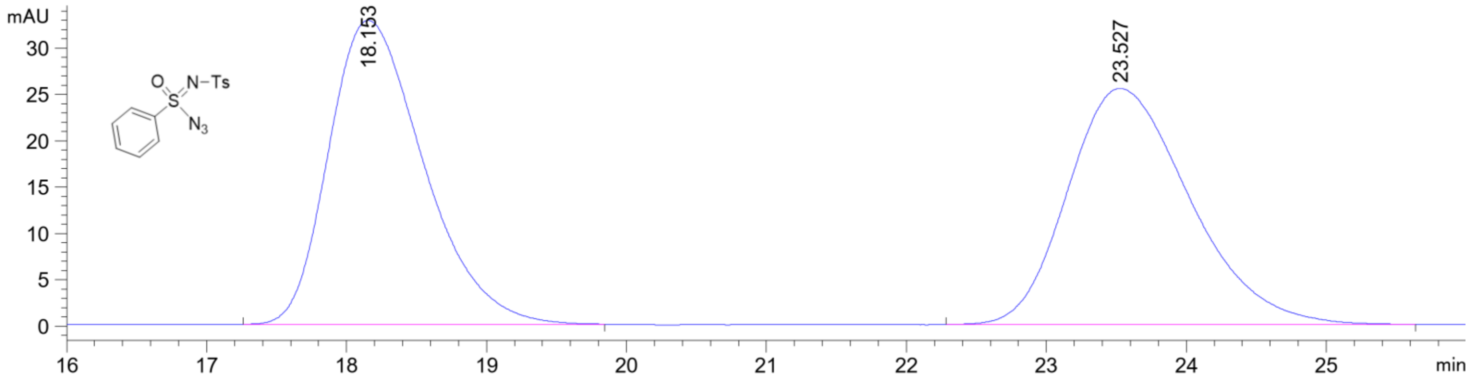

Peak #	RetTime [min]	Type	Width [min]	Area [mAU*s]	Height [mAU]	Area %
1	18.153	BB	0.7257	1545.88867	32.80812	50.0053
2	23.527	BB	0.9091	1545.55945	25.50212	49.9947

(*R*)-*N*'-Tosylbenzenesulfonimidoyl azide (5)

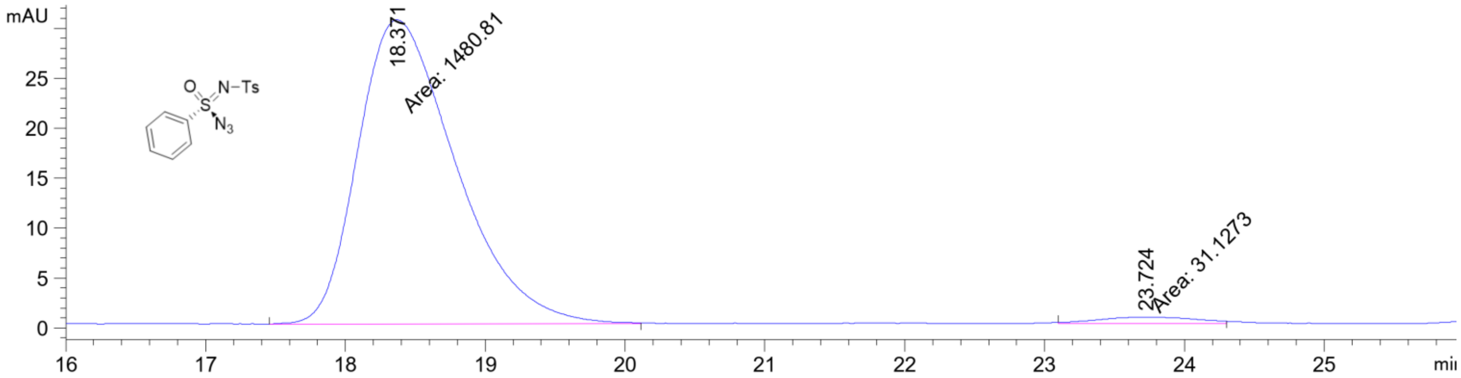

Peak #	RetTime [min]	Type	Width [min]	Area [mAU*s]	Height [mAU]	Area %
1	18.371	BB	0.7316	1481.15820	30.44357	97.7682
2	23.724	MM	0.8540	33.81076	6.59846e-1	2.2318

Phenyl (*S*)-*N*-tosylbenzenesulfonimidate (*rac*-6)

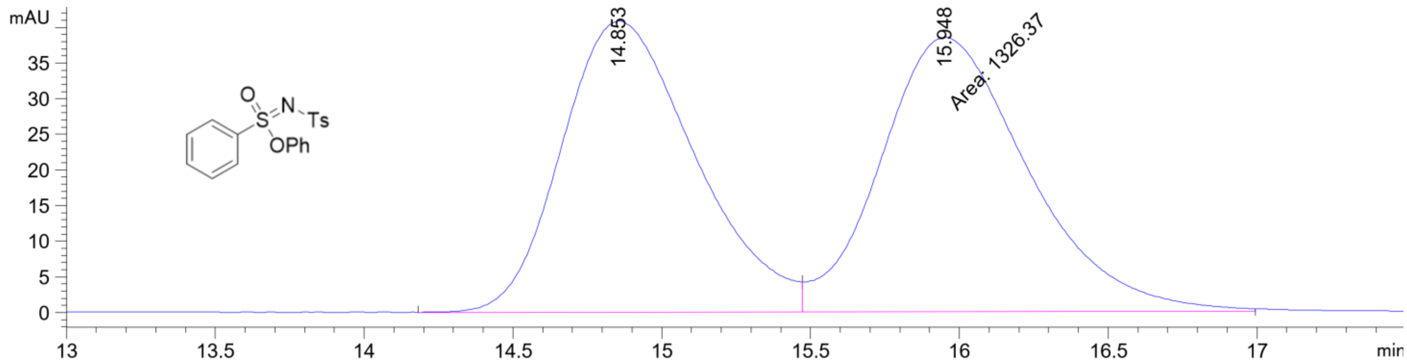

Peak #	RetTime [min]	Type	Width [min]	Area [mAU*s]	Height [mAU]	Area %
1	14.853	BV	0.4821	1.25701e4	402.80536	48.7392
2	15.948	VB	0.5319	1.32205e4	379.94675	51.2608

Phenyl (*S*)-*N*-tosylbenzenesulfonimidate (6)

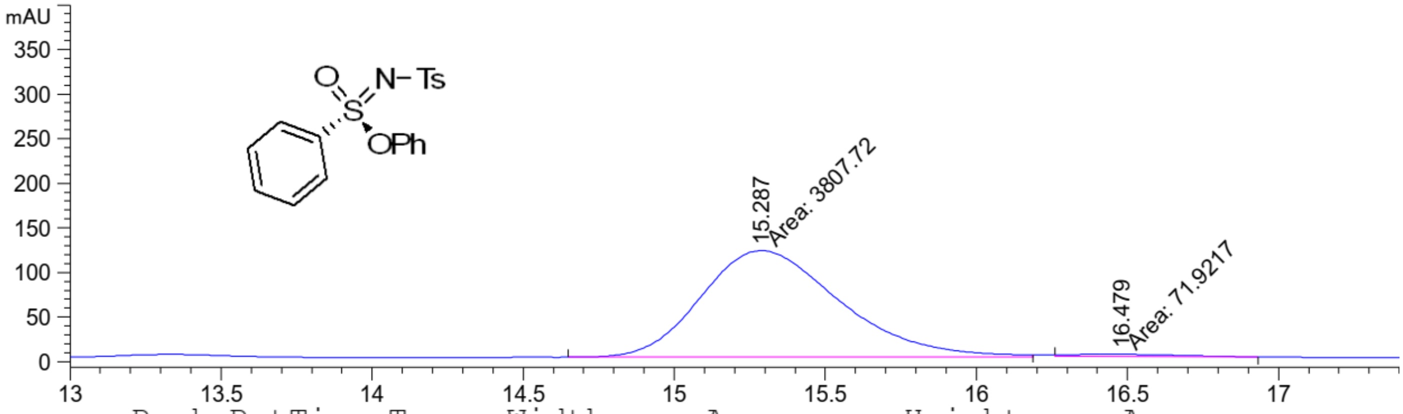

Peak #	RetTime [min]	Type	Width [min]	Area [mAU*s]	Height [mAU]	Area %
1	15.287	MM	0.5330	3807.71606	119.07511	98.1462
2	16.479	MM	0.4528	71.92168	2.64749	1.8538

(*R*)-4-Methyl-*N*-(oxo(phenyl)(piperidin-1-yl)- λ⁶-sulfaneylidene)benzenesulfonamide (*rac*-7)

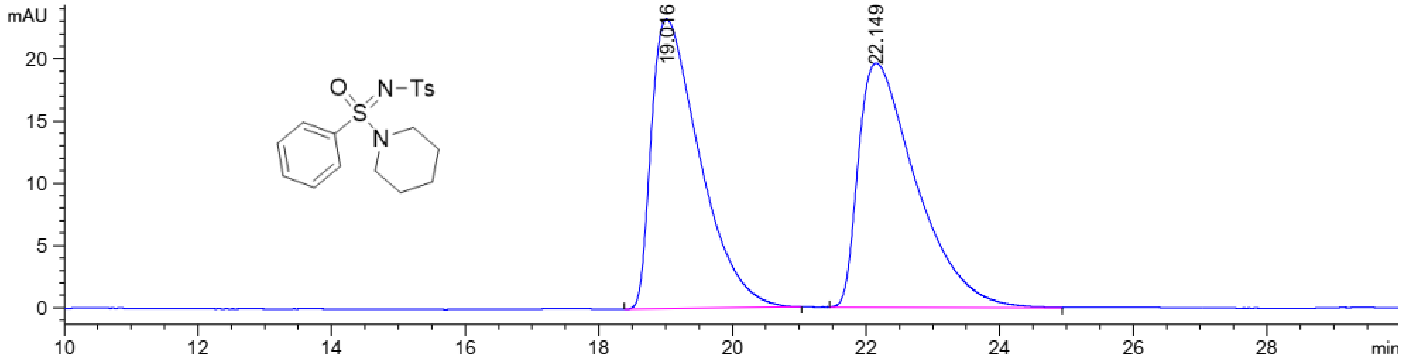

Peak #	RetTime [min]	Type	Width [min]	Area [mAU*s]	Height [mAU]	Area %
1	19.016	BB	0.7294	1171.10559	23.26087	49.7707
2	22.149	BB	0.8597	1181.89624	19.59454	50.2293

(*R*)-4-Methyl-*N*-(oxo(phenyl)(piperidin-1-yl)- λ⁶-sulfaneylidene)benzenesulfonamide (7)

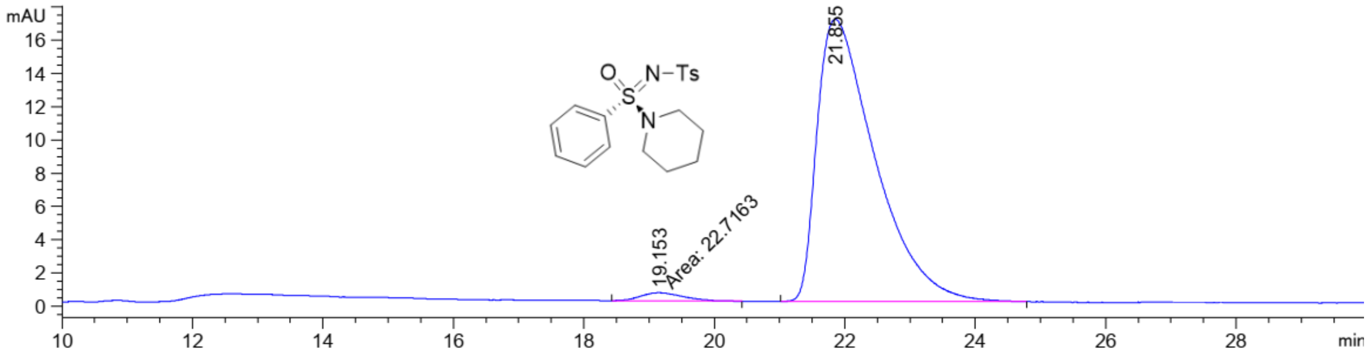

Peak #	RetTime [min]	Type	Width [min]	Area [mAU*s]	Height [mAU]	Area %
1	19.153	MM	0.7540	22.71633	5.02151e-1	2.1194
2	21.855	BB	0.8890	1049.11597	16.97652	97.8806

(*S*)-*N*-((4-(8-Chloro-5,6-dihydro-11H-benzo[5,6]cyclohepta[1,2-b]pyridin-11-ylidene)piperidin-1-yl)(oxo)(phenyl)- λ⁶-sulfaneylidene)-4-methylbenzenesulfonamide (*rac*-8)

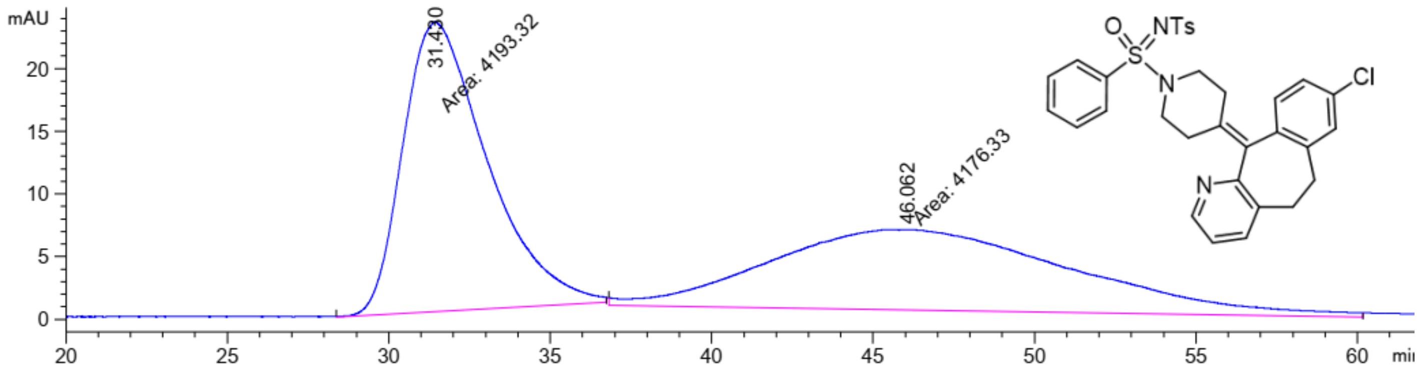

Peak #	RetTime [min]	Type	Width [min]	Area [mAU*s]	Height [mAU]	Area %
1	31.430	MM	3.0242	4193.32471	23.10954	50.1015
2	46.062	MM	10.8864	4176.33057	6.39380	49.8985

(*S*)-*N*-((4-(8-Chloro-5,6-dihydro-11H-benzo[5,6]cyclohepta[1,2-b]pyridin-11-ylidene)piperidin-1-yl)(oxo)(phenyl)- λ⁶-sulfaneylidene)-4-methylbenzenesulfonamide (8)

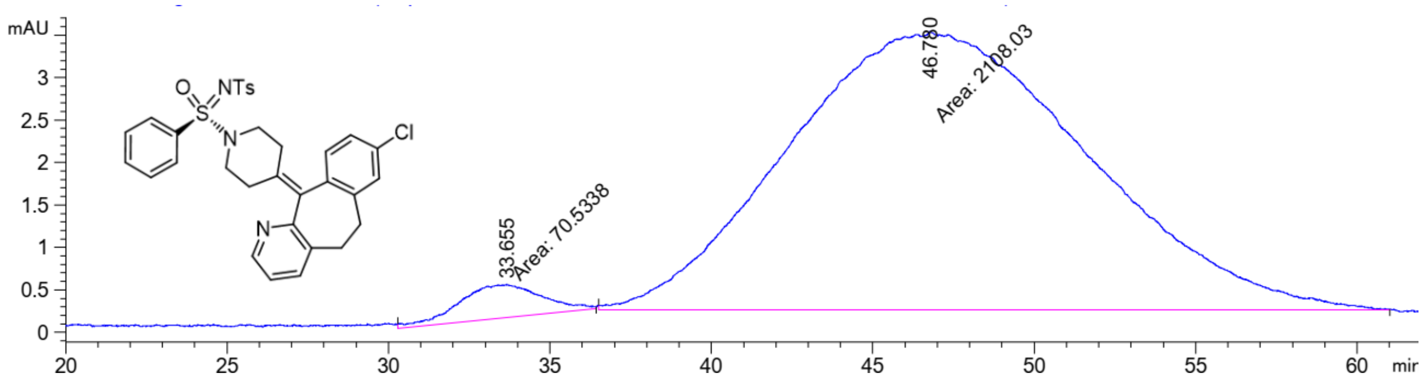

Peak #	RetTime [min]	Type	Width [min]	Area [mAU*s]	Height [mAU]	Area %
1	33.655	MM	3.0418	70.53375	3.86471e-1	3.2376
2	46.780	MM	10.7274	2108.03491	3.27514	96.7624

(*S*)-*N*-((4-(6-Fluorobenzo[d]isoxazol-3-yl)piperidin-1-yl)(oxo)(phenyl)- λ^6-sulfaneylidene)-4-methylbenzenesulfonamide (*rac*-9)

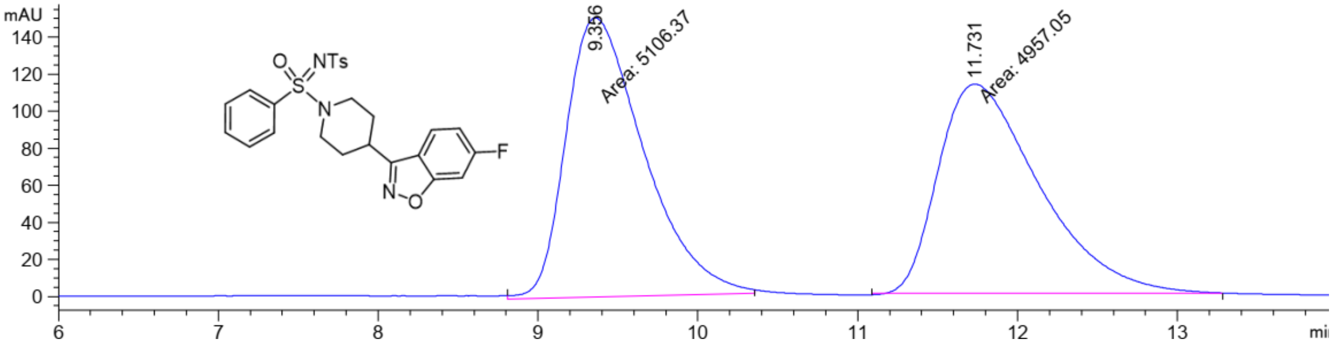

Peak #	RetTime [min]	Type	Width [min]	Area [mAU*s]	Height [mAU]	Area %
1	9.356	MM	0.5651	5106.36621	150.60446	50.7419
2	11.731	MM	0.7316	4957.05176	112.92818	49.2581

(*S*)-*N*-((4-(6-Fluorobenzo[d]isoxazol-3-yl)piperidin-1-yl)(oxo)(phenyl)- λ^6-sulfaneylidene)-4-methylbenzenesulfonamide (9)

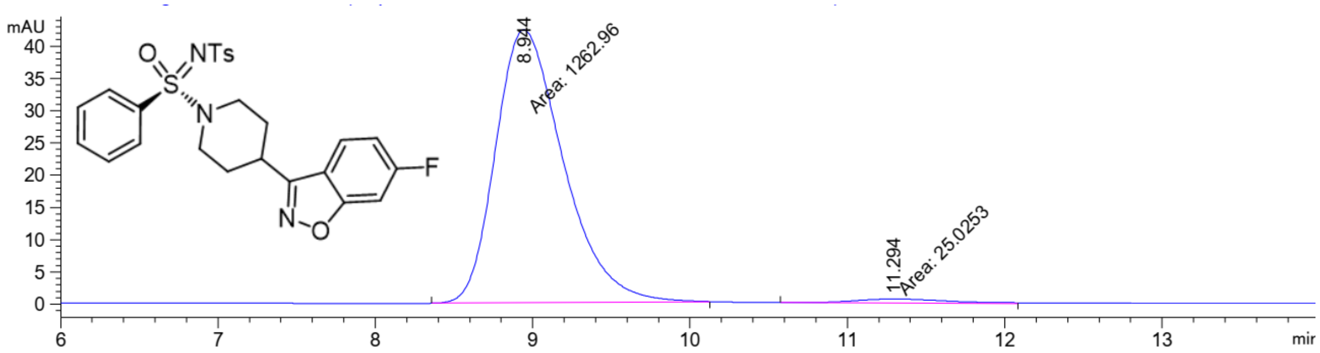

Peak #	RetTime [min]	Type	Width [min]	Area [mAU*s]	Height [mAU]	Area %
1	8.944	MM	0.4981	1262.96472	42.26224	98.0570
2	11.294	MM	0.6835	25.02529	6.10195e-1	1.9430

5-Methyl (*R*)-2-((*tert*-butoxycarbonyl)amino)-3-(4-(((*S*)-N-tosylphenylsulfonimidoyl)oxy)phenyl)propanoate (*rac*-10)

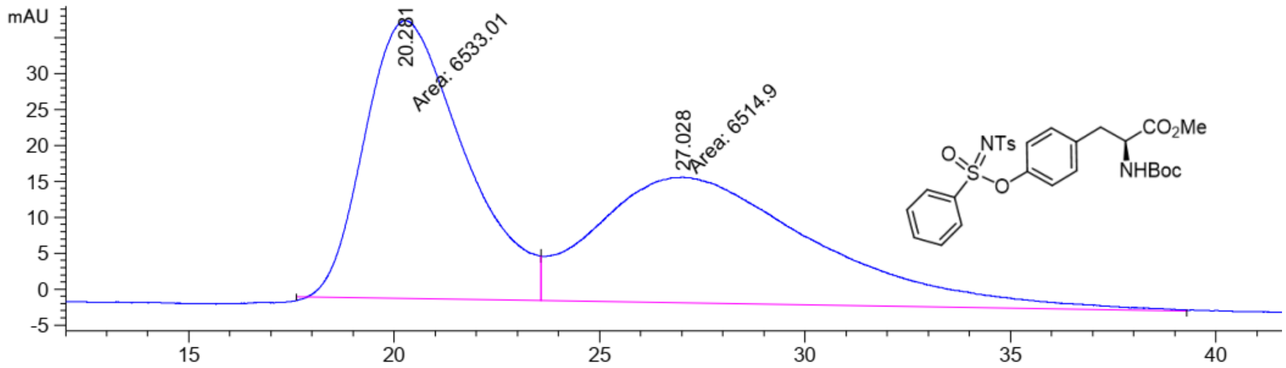

Peak #	RetTime [min]	Type	Width [min]	Area [mAU*s]	Height [mAU]	Area %
1	20.281	MF	2.8153	6533.01123	38.67604	50.0694
2	27.028	FM	6.2044	6514.90479	17.50084	49.9306

5-Methyl (*R*)-2-((*tert*-butoxycarbonyl)amino)-3-(4-(((*S*)-N-tosylphenylsulfonimidoyl)oxy)phenyl)propanoate (10)

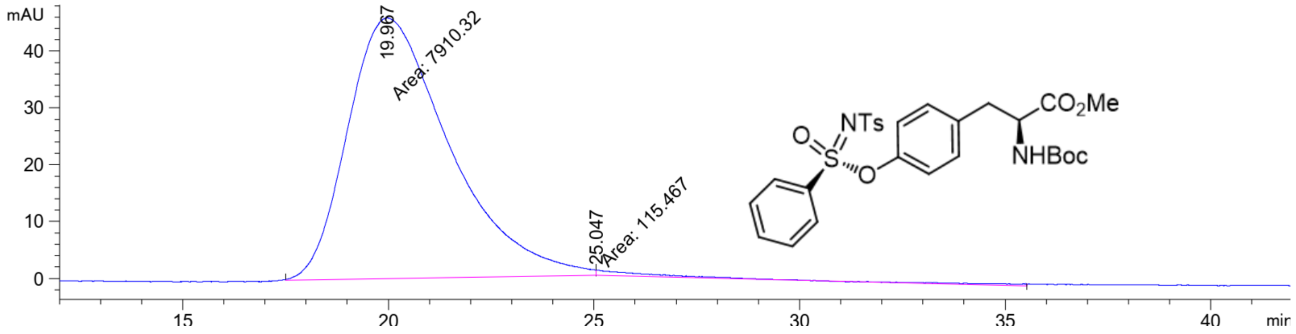

Peak #	RetTime [min]	Type	Width [min]	Area [mAU*s]	Height [mAU]	Area %
1	19.967	MM	2.8701	7910.31641	45.93564	98.5613
2	25.047	MM	1.4911	115.46652	9.06978e-1	1.4387

4-((2*S*,3*R*)-1-(4-Fluorophenyl)-3-((*R*)-3-(4-fluorophenyl)-3-hydroxypropyl)-4-oxoazetidin-2-yl)phenyl (*S*)-*N*-tosylbenzenesulfonimidate (*rac*-11)

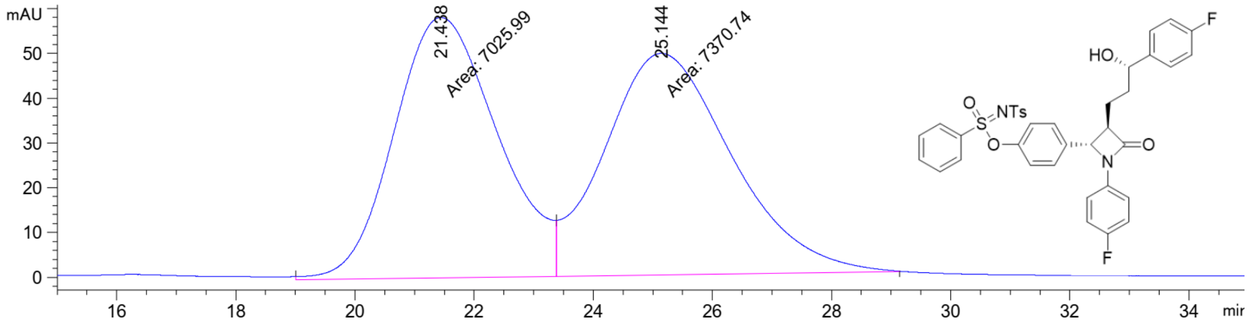

Peak #	RetTime [min]	Type	Width [min]	Area [mAU*s]	Height [mAU]	Area %
1	21.438	MF	2.0155	7025.98926	58.09854	48.8027
2	25.144	FM	2.4839	7370.73633	49.45622	51.1973

4-((2*S*,3*R*)-1-(4-Fluorophenyl)-3-((*R*)-3-(4-fluorophenyl)-3-hydroxypropyl)-4-oxoazetidin-2-yl)phenyl (*S*)-*N*-tosylbenzenesulfonimidate (11)

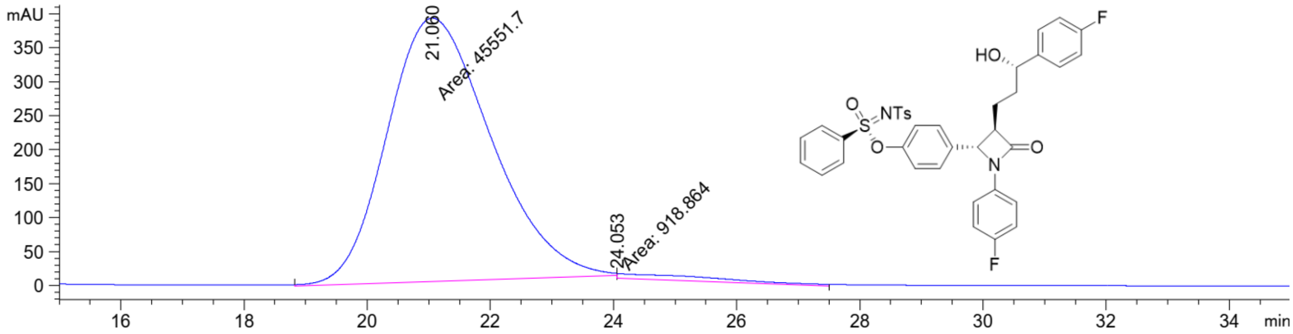

Peak #	RetTime [min]	Type	Width [min]	Area [mAU*s]	Height [mAU]	Area %
1	21.060	MM	1.9570	4.55517e4	387.93243	98.0227
2	24.053	MM	1.5781	918.86353	6.79754	1.9773

Go to table of contents

2,2-Dimethyl-3-(((*R*)-*N*-tosylphenylsulfonimidoyl)oxy)propyl ($3^5S,9^1R,9^2R,5S$)-5-(tert-butyl)-17-methoxy-4,7-dioxo-2,8-dioxa-6-aza-1(2,3)-quinoxalina-3(3,1)-pyrrolidina-9(1,2)-cyclopropanacyclotetradecaphane-3^5-carboxylate (*rac*-12)

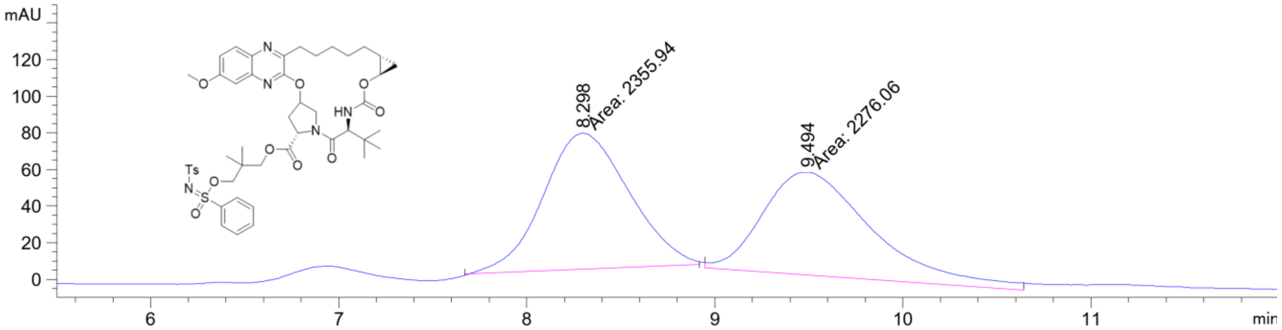

Peak #	RetTime [min]	Type	Width [min]	Area [mAU*s]	Height [mAU]	Area %
1	8.298	MM	0.5292	2355.93774	74.19962	50.8623
2	9.494	MM	0.6762	2276.05542	56.09650	49.1377

2,2-Dimethyl-3-(((*R*)-*N*-tosylphenylsulfonimidoyl)oxy)propyl ($3^5S,9^1R,9^2R,5S$)-5-(tert-butyl)-17-methoxy-4,7-dioxo-2,8-dioxa-6-aza-1(2,3)-quinoxalina-3(3,1)-pyrrolidina-9(1,2)-cyclopropanacyclotetradecaphane-3^5-carboxylate (12)

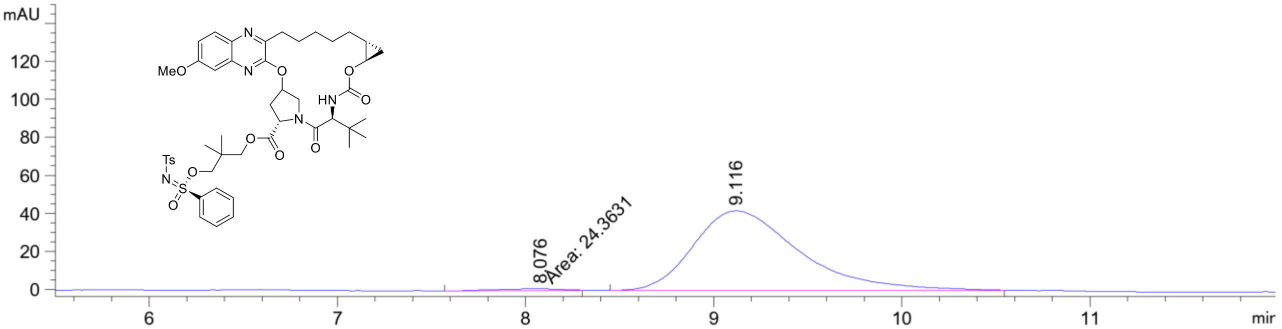

Peak #	RetTime [min]	Type	Width [min]	Area [mAU*s]	Height [mAU]	Area %
1	8.076	MM	0.4066	24.36308	9.98580e-1	1.4694
2	9.116	BB	0.5695	1633.63281	41.84591	98.5306

(*R*)-2-(2,2-dimethyl-3-((*N*-tosylphenylsulfonimidoyl)oxy)propoxy)-2-oxoethyl 2-(1-(4-chlorobenzoyl)-5-methoxy-2-methyl-1*H*-indol-3-yl)acetate (*rac*-13)

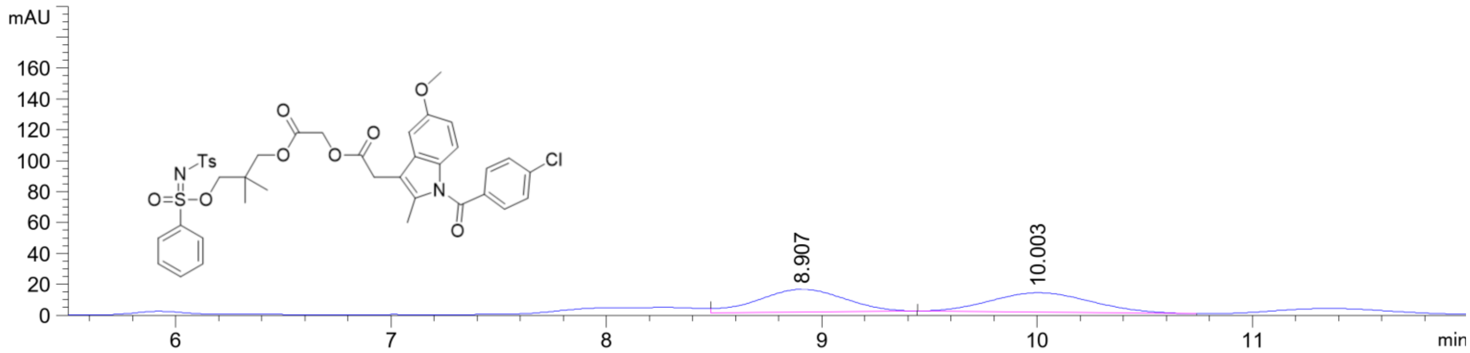

Peak #	RetTime [min]	Type	Width [min]	Area [mAU*s]	Height [mAU]	Area %
1	8.907	VB	0.4331	407.72937	14.63304	49.8108
2	10.003	BB	0.4885	410.82639	12.59807	50.1892

(*R*)-2-(2,2-dimethyl-3-((*N*-tosylphenylsulfonimidoyl)oxy)propoxy)-2-oxoethyl 2-(1-(4-chlorobenzoyl)-5-methoxy-2-methyl-1*H*-indol-3-yl)acetate (13)

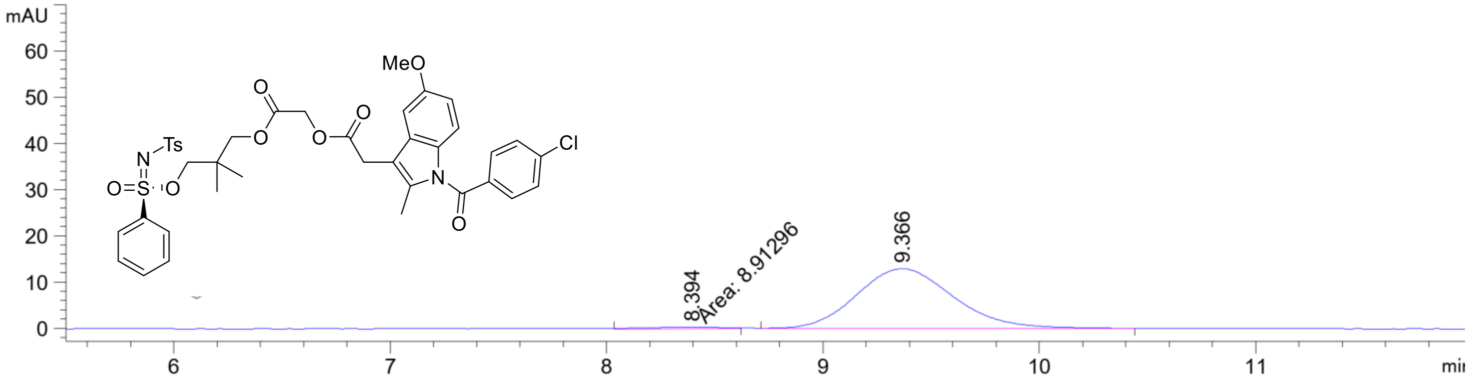

Peak #	RetTime [min]	Type	Width [min]	Area [mAU*s]	Height [mAU]	Area %
1	8.394	MM	0.4022	8.91296	3.69341e-1	2.1831
2	9.366	BB	0.4463	399.34903	12.86117	97.8169

Go to table of contents

2,2-Dimethyl-3-(((*R*)-*N*-tosylphenylsulfonimidoyl)oxy)propyl 6-(3-((3r,5r,7r)-adamantan-1-yl)-4-methoxyphenyl)-2-naphthoate (*rac*-14)

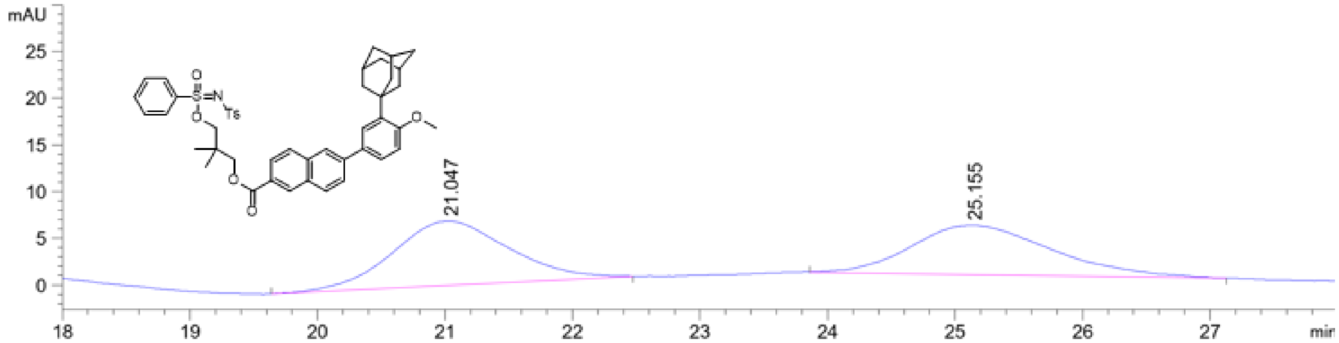

Peak #	RetTime [min]	Type	Width [min]	Area [mAU*s]	Height [mAU]	Area %
1	21.047	BB	0.7633	435.38800	6.85222	51.8707
2	25.155	BB	0.9071	403.98300	5.28906	48.1293

2,2-Dimethyl-3-(((*R*)-*N*-tosylphenylsulfonimidoyl)oxy)propyl 6-(3-((3r,5r,7r)-adamantan-1-yl)-4-methoxyphenyl)-2-naphthoate (14)

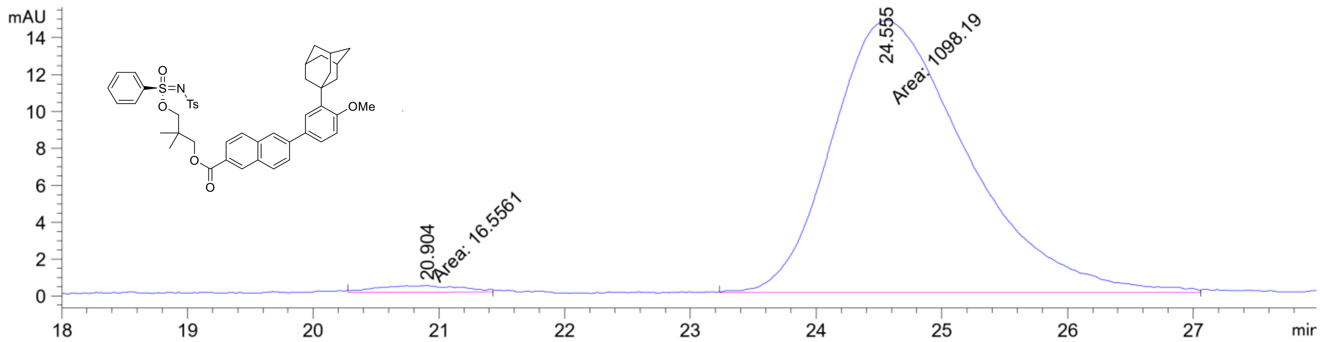

Peak #	RetTime [min]	Type	Width [min]	Area [mAU*s]	Height [mAU]	Area %
1	20.904	MM	0.9961	22.57542	3.77732e-1	2.0875
2	24.555	BB	0.8833	1058.85754	14.58048	97.9125

(*R*)-2,2-dimethyl-3-((*N*-tosylphenylsulfonimidoyl)oxy)propyl 2-(3-cyano-4-isobutoxyphenyl)-4-methylthiazole-5-carboxylate (*rac*-15)

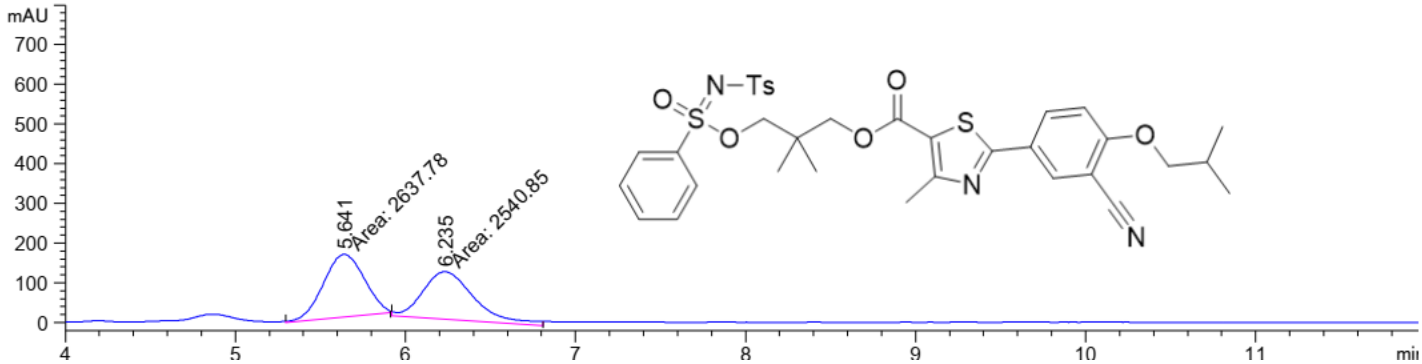

Peak #	RetTime [min]	Type	Width [min]	Area [mAU*s]	Height [mAU]	Area %
1	5.641	MM	0.2790	2637.77734	157.55666	50.9359
2	6.235	MM	0.3531	2540.84692	119.93472	49.0641

(*R*)-2,2-dimethyl-3-((*N*-tosylphenylsulfonimidoyl)oxy)propyl 2-(3-cyano-4-isobutoxyphenyl)-4-methylthiazole-5-carboxylate (15)

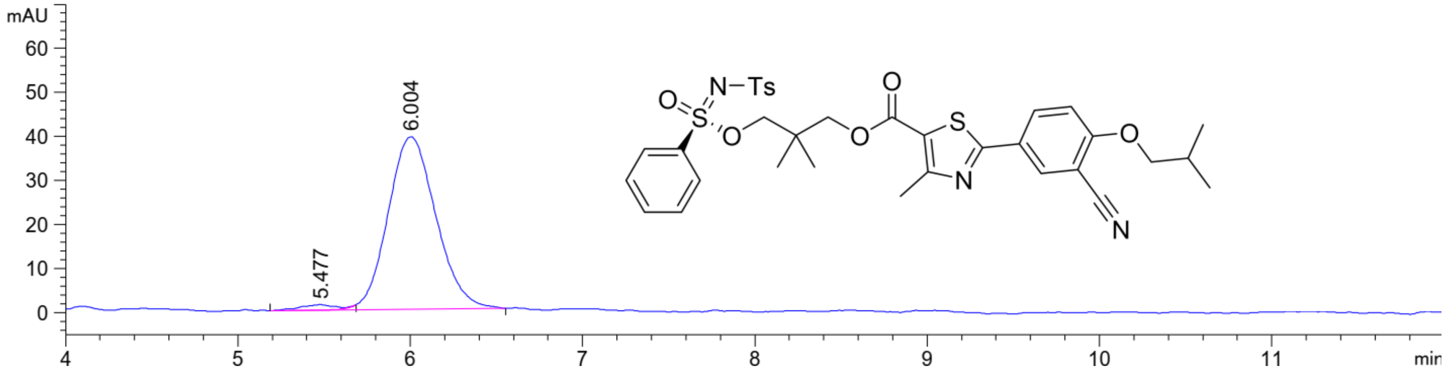

Peak #	RetTime [min]	Type	Width [min]	Area [mAU*s]	Height [mAU]	Area %
1	5.477	BV E	0.1761	17.35139	1.26415	2.2440
2	6.004	VB R	0.2974	755.88538	39.12733	97.7560

Go to table of contents

(*R*)-*N*-(Butyl(oxo)(phenyl)-λ⁶-sulfaneylidene)-4-methylbenzenesulfonamide (*rac*-16)

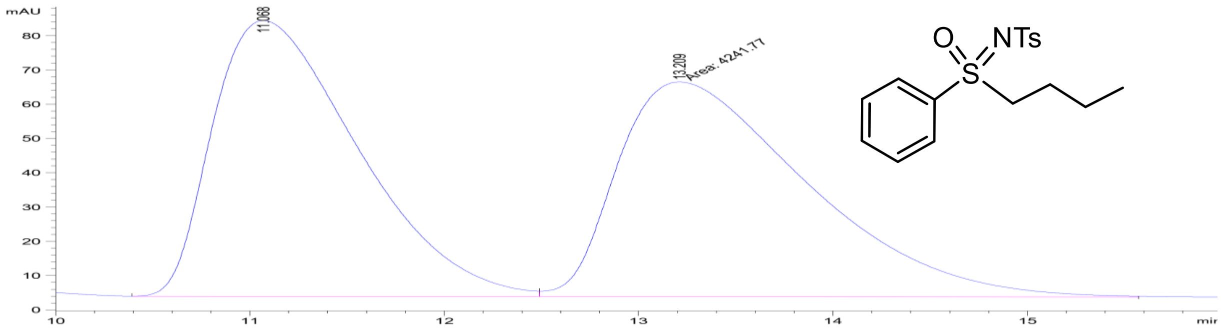

Peak #	RetTime [min]	Type	Width [min]	Area [mAU*s]	Height [mAU]	Area %
1	11.068	BV	0.8105	4166.38818	80.41367	49.5517
2	13.209	MF	1.1295	4241.77441	62.59092	50.4483

(*R*)-*N*-(Butyl(oxo)(phenyl)-λ⁶-sulfaneylidene)-4-methylbenzenesulfonamide (16)

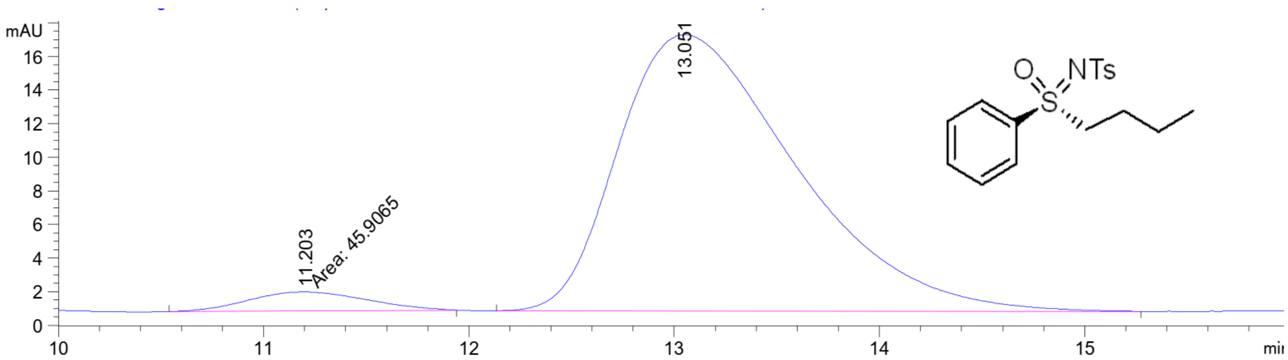

Peak #	RetTime [min]	Type	Width [min]	Area [mAU*s]	Height [mAU]	Area %
1	11.203	MM	0.6692	45.90647	1.14334	4.4386
2	13.051	BB	0.8852	988.36023	16.44377	95.5614

Go to table of contents

(*R*)-4-methyl-N-(methyl(oxo)(phenyl)- λ⁶-sulfaneylidene)benzenesulfonamide (*rac*-17)

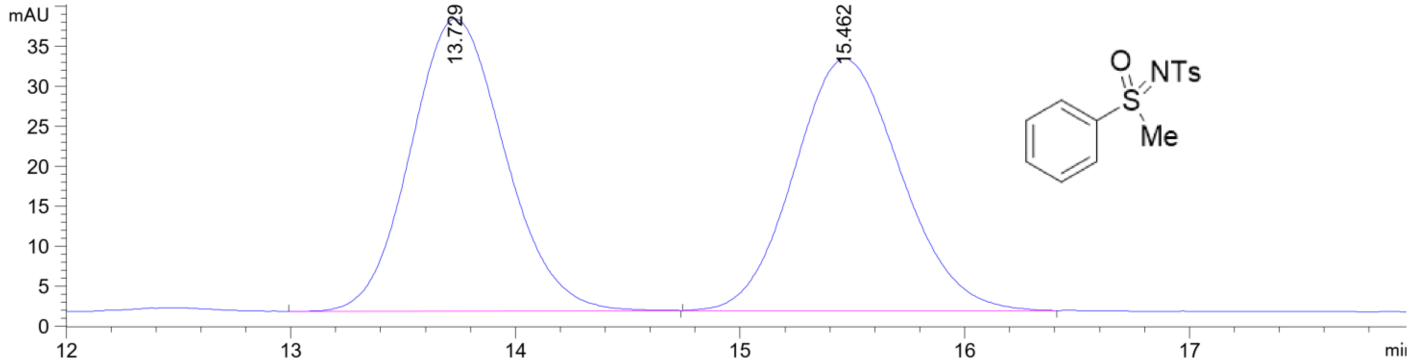

Peak #	RetTime [min]	Type	Width [min]	Area [mAU*s]	Height [mAU]	Area %
1	13.730	BB	0.4269	75.26188	2.60638	50.2759
2	15.455	BB	0.4341	74.43577	2.24942	49.7241

(*R*)-4-methyl-N-(methyl(oxo)(phenyl)- λ⁶-sulfaneylidene)benzenesulfonamide (17)

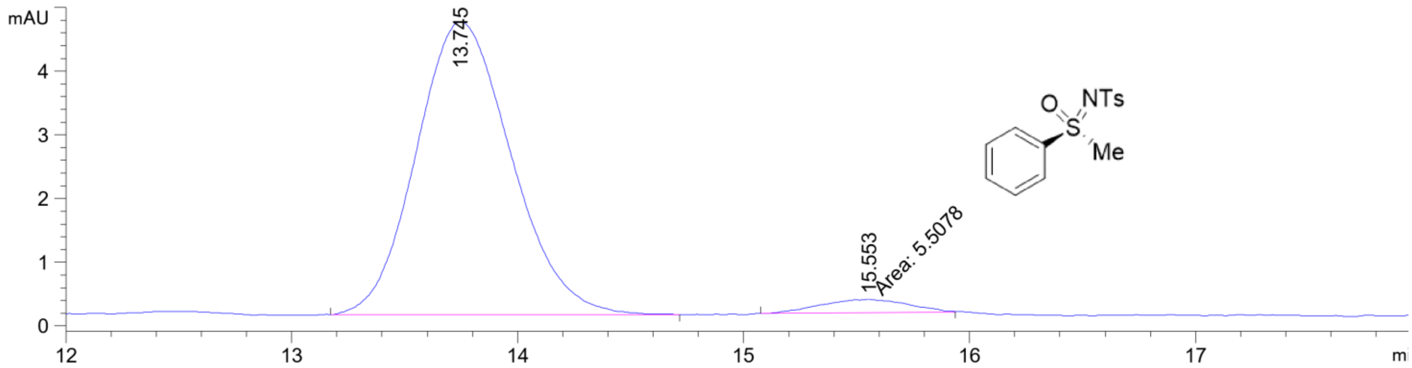

Peak #	RetTime [min]	Type	Width [min]	Area [mAU*s]	Height [mAU]	Area %
1	13.745	BB	0.4465	134.15451	4.59661	96.0563
2	15.553	MM	0.4416	5.50780	2.07861e-1	3.9437

((*R*)-4-methyl-N-(oxo(phenethyl)(phenyl)- λ⁶-sulfaneylidene)benzenesulfonamide (*rac*-18)

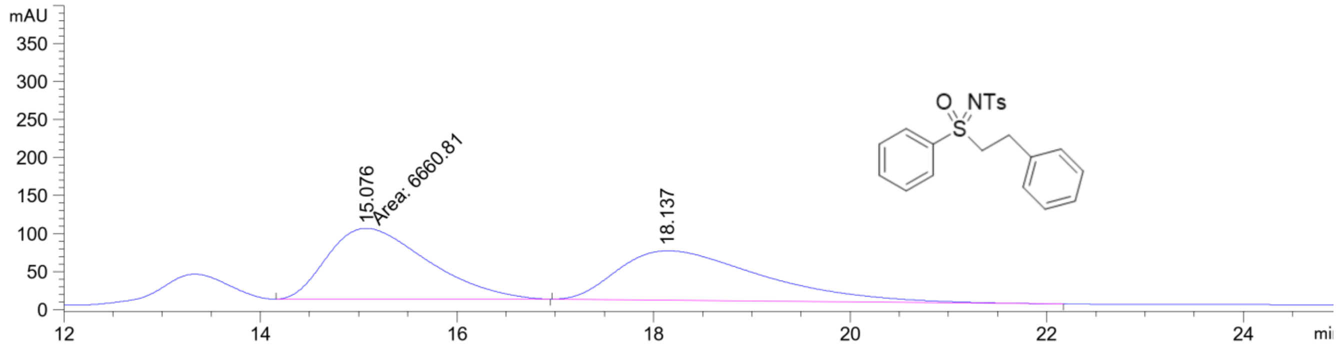

Peak #	RetTime [min]	Type	Width [min]	Area [mAU*s]	Height [mAU]	Area %
1	15.076	MM	1.1883	6660.81445	93.42534	49.9949
2	18.137	BB	1.4053	6662.17529	65.02525	50.0051

((*R*)-4-methyl-N-(oxo(phenethyl)(phenyl)- λ⁶-sulfaneylidene)benzenesulfonamide (18)

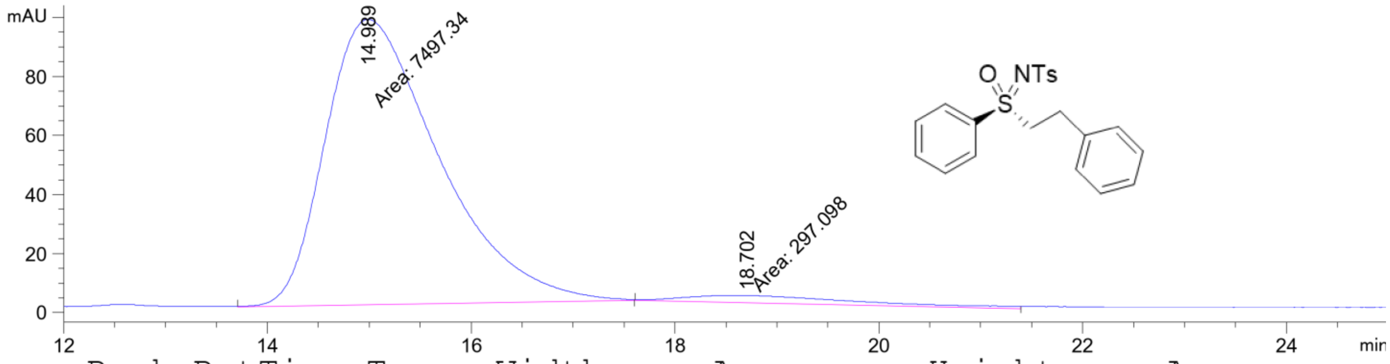

Peak #	RetTime [min]	Type	Width [min]	Area [mAU*s]	Height [mAU]	Area %
1	14.989	MM	1.2929	7497.33936	96.64411	96.1883
2	18.702	MM	1.9921	297.09750	2.48568	3.8117

(*R*)-*N*-((4-Bromophenyl)(oxo)(phenyl)-λ⁶-sulfaneylidene)-4-methylbenzenesulfonamide (*rac*-19)

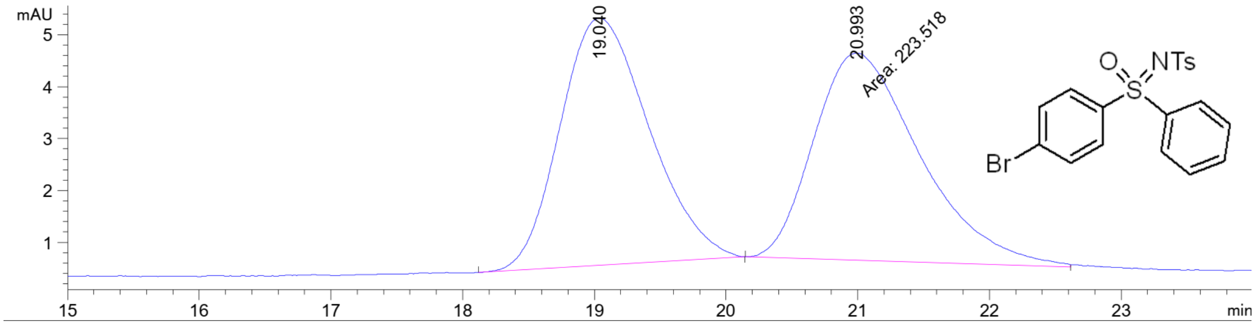

Peak #	RetTime [min]	Type	Width [min]	Area [mAU*s]	Height [mAU]	Area %
1	19.040	BB	0.6577	223.56697	4.76249	50.0054
2	20.993	MF	0.9333	223.51831	3.99158	49.9946

(*R*)-*N*-((4-Bromophenyl)(oxo)(phenyl)-λ⁶-sulfaneylidene)-4-methylbenzenesulfonamide (19)

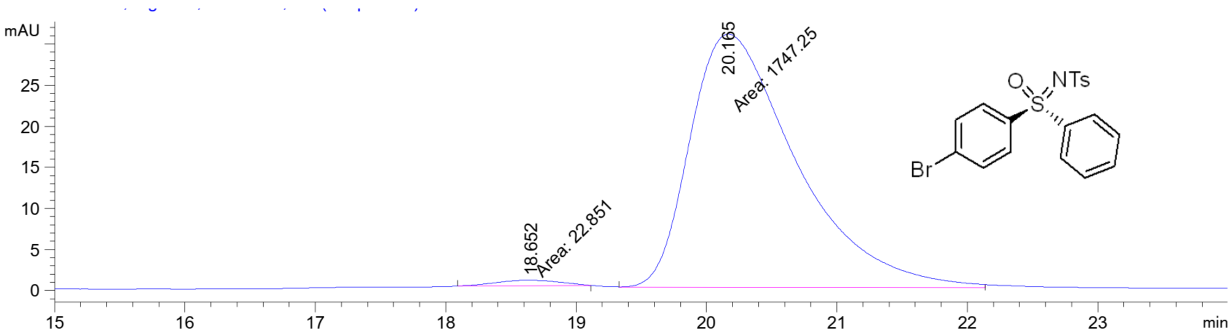

Peak #	RetTime [min]	Type	Width [min]	Area [mAU*s]	Height [mAU]	Area %
1	18.652	MM	0.5703	22.85096	6.67840e-1	1.2909
2	20.165	MF	0.9454	1747.25464	30.80319	98.7091

(S)-4-Methyl-N-(oxo(phenyl)(piperidin-1-yl)- λ⁶-sulfaneylidene)benzenesulfonamide (rac-7)

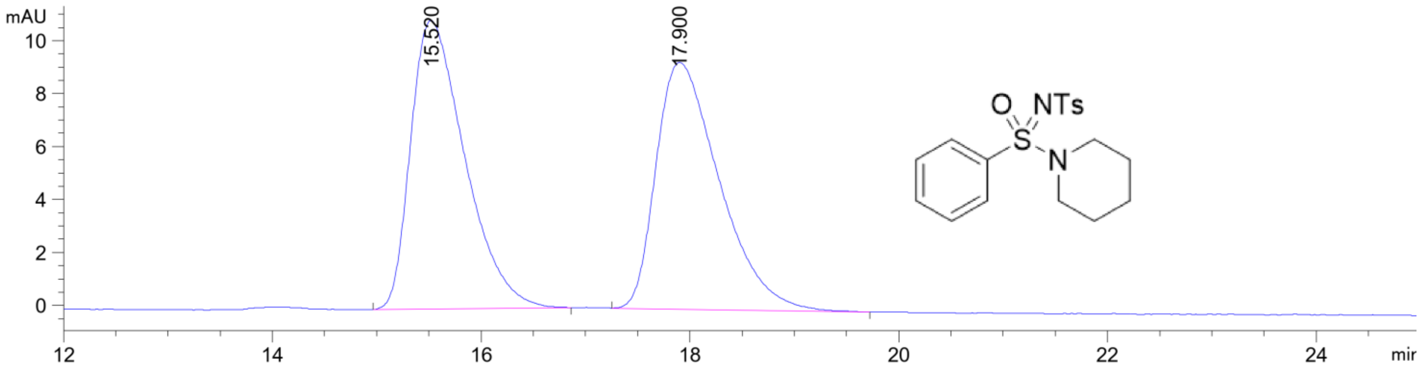

Peak #	RetTime [min]	Type	Width [min]	Area [mAU*s]	Height [mAU]	Area %
1	15.520	BB	0.5269	393.24484	10.89931	50.0157
2	17.900	BB	0.6060	392.99832	9.31870	49.9843

(S)-4-Methyl-N-(oxo(phenyl)(piperidin-1-yl)- λ⁶-sulfaneylidene)benzenesulfonamide (ent-7)

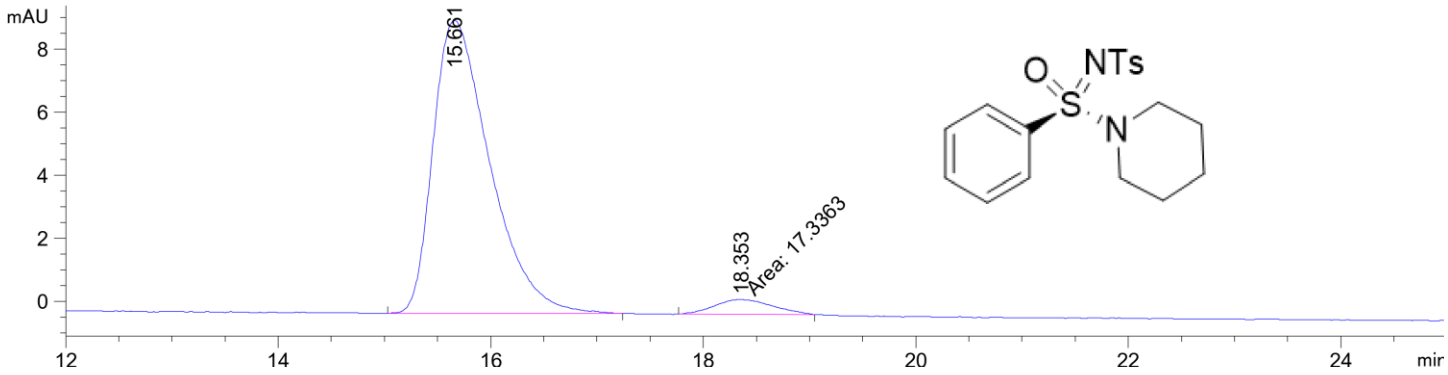

Peak #	RetTime [min]	Type	Width [min]	Area [mAU*s]	Height [mAU]	Area %
1	15.661	BB	0.5556	345.24271	9.28930	95.2186
2	18.353	MM	0.6243	17.33632	4.62798e-1	4.7814

(*S*)-4-Methyl-*N*-(morpholino(oxo)(phenyl)- λ^6-sulfaneylidene)benzenesulfonamide (*rac*-20)

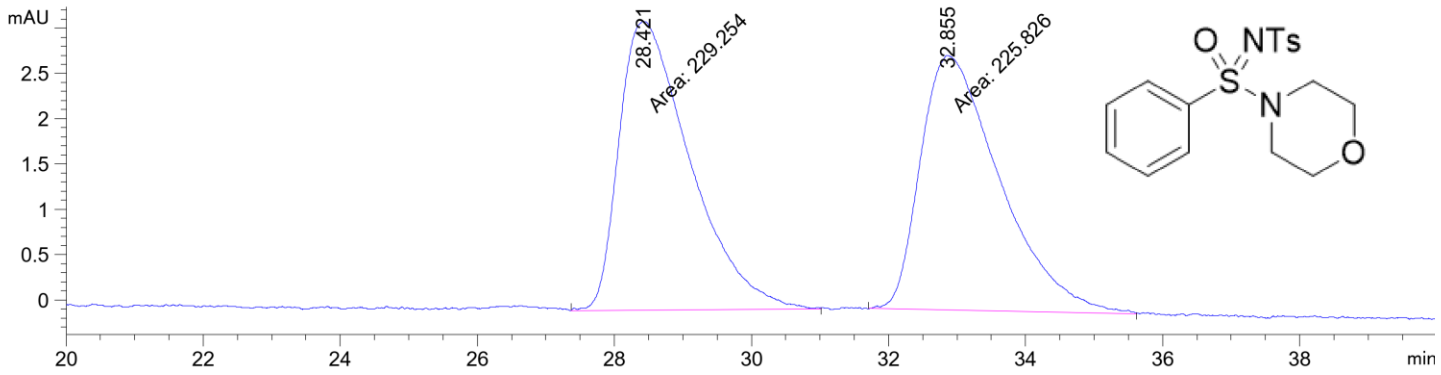

Peak #	RetTime [min]	Type	Width [min]	Area [mAU*s]	Height [mAU]	Area %
1	28.421	MM	1.1992	229.25420	3.18609	50.3766
2	32.855	MM	1.3394	225.82617	2.81002	49.6234

(*S*)-4-Methyl-*N*-(morpholino(oxo)(phenyl)- λ^6-sulfaneylidene)benzenesulfonamide (20)

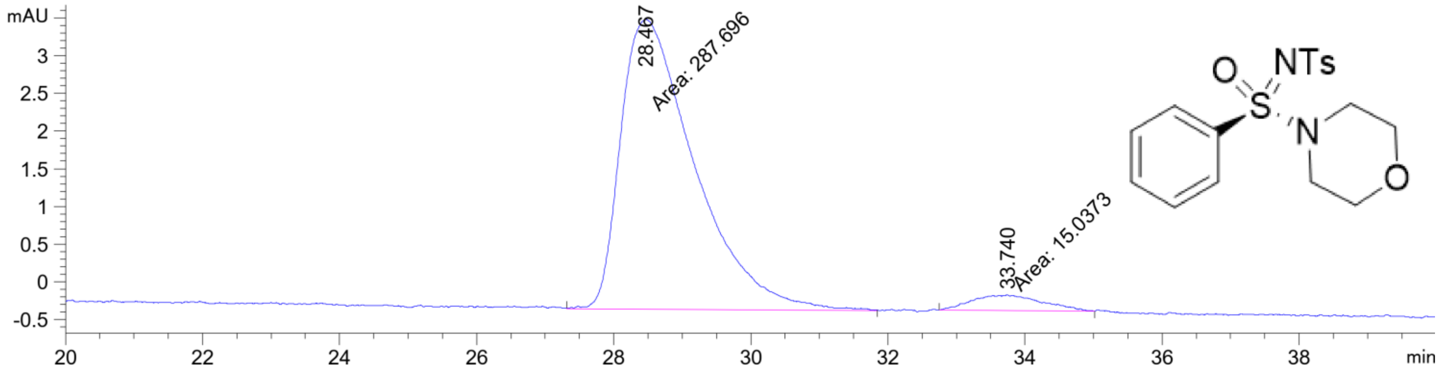

Peak #	RetTime [min]	Type	Width [min]	Area [mAU*s]	Height [mAU]	Area %
1	28.467	MM	1.2485	287.69565	3.84070	95.0328
2	33.740	MM	1.1968	15.03728	2.09402e-1	4.9672

References

[1] M. D. Greenhalgh, J. E. Taylor, A. D. Smith, *Tetrahedron* **2018**, *74*, 5554–5560.

[2] T. O. Luukas, C. Girard, D. R. Fenwick, H. B. Kagan, *J. Am. Chem. Soc.* **1999**, *121*, 9299–9306.

[3] Gaussian 16, Revision A.03, M. J. Frisch,; G. W. Trucks,; H. B. Schlegel,; G. E. Scuseria,; M. A. Robb,; J. R. Cheeseman,; G. Scalmani; V. Barone,; G. A. Petersson,; H. Nakatsuji,; X. Li,; M. Caricato,; A. V. Marenich,; J. Bloino,; B. G. Janesko,; R. Gomperts,; B. Mennucci,; H. P. Hratchian,; J. V. Ortiz,; A. F. Izmaylov,; J. L. Sonnenberg,; D. Williams-Young,; F. Ding,; F. Lipparini,; F. Egidi,; J. Goings,; B. Peng,; A. Petrone,; T. Henderson,; D. Ranasinghe,; V. G. Zakrzewski,; J. Gao,; N. Rega,; G. Zheng,; W. Liang,; M. Hada,; M. Ehara,; K. Toyota,; R. Fukuda,; J. Hasegawa,; M. Ishida,; T. Nakajima,; Y. Honda,; O. Kitao,; H. Nakai,; T. Vreven,; K. Throssell,; J. A. Montgomery, Jr., J. E. Peralta,; F. Ogliaro,; M. J. Bearpark,; J. J. Heyd,; E. N. Brothers,; K. N. Kudin,; V. N. Staroverov,; T. A. Keith,; R. Kobayashi,; J. Normand,; K. Raghavachari,; A. P. Rendell,; J. C. Burant,; S. S. Iyengar,; J. Tomasi,; M. Cossi,; J. M. Millam,; M. Klene,; C. Adamo,; R. Cammi,; J. W. Ochterski,; R. L. Martin,; K. Morokuma,; O. Farkas,; J. B. Foresman,; D. J. Fox, Gaussian, Inc., Wallingford CT, 2016.

[4] Chemcraft – graphical software for visualization of quantum chemistry computations. Version 1.8, build 682. https://www.chemcraftprog.com

[5] R. Z. Khaliullin, E. A. Cobar, R. C. Lochan, A. T. Bell, M. Head-Gordon, *J. Phys. Chem. A* **2007**, *111*, 8753–8765.

[6] Y. Shao, Z. Gan, E. Epifanovsky, A. T. B. Gilbert, M. Wormit, J. Kussmann, A. W. Lange, A. Behn, J. Deng, X. Feng, D. Ghosh, M. Goldey, P. R. Horn, L. D. Jacobson, I. Kaliman, R. Z. Khaliullin, T. Kuś, A. Landau, J. Liu, E. I. Proynov, Y. M. Rhee, R. M. Richard, M. A. Rohrdanz, R. P. Steele, E. J. Sundstrom, H. L. Woodcock, P. M. Zimmerman, D. Zuev, B. Albrecht, E. Alguire, B. Austin, G. J. O. Beran, Y. A. Bernard, E. Berquist, K. Brandhorst, K. B. Bravaya, S. T. Brown, D. Casanova, C.-M. Chang, Y. Chen, S. H. Chien, K. D. Closser, D. L. Crittenden, M. Diedenhofen, R. A. DiStasio, H. Do, A. D. Dutoi, R. G. Edgar, S. Fatehi, L. Fusti-Molnar, A. Ghysels, A. Golubeva-Zadorozhnaya, J. Gomes, M. W. D. Hanson-Heine, P. H. P. Harbach, A. W. Hauser, E. G. Hohenstein, Z. C. Holden, T.-C. Jagau, H. Ji, B. Kaduk, K. Khistyaev, J. Kim, J. Kim, R. A. King, P. Klunzinger, D. Kosenkov, T. Kowalczyk, C. M. Krauter, K. U. Lao, A. D. Laurent, K. V. Lawler, S. V. Levchenko, C. Y. Lin, F. Liu, E. Livshits, R. C. Lochan, A. Luenser, P. Manohar, S. F. Manzer, S.-P. Mao, N. Mardirossian, A. V. Marenich, S. A. Maurer, N. J. Mayhall, E. Neuscamman, C. M. Oana, R. Olivares-Amaya, D. P. O'Neill, J. A. Parkhill, T. M. Perrine, R. Peverati, A. Prociuk, D. R. Rehn, E. Rosta, N. J. Russ, S. M. Sharada, S. Sharma, D. W. Small, A. Sodt, T. Stein, D. Stück, Y.-C. Su, A. J. W. Thom, T. Tsuchimochi, V. Vanovschi, L. Vogt, O. Vydrov, T. Wang, M. A. Watson, J. Wenzel, A. White, C. F. Williams, J. Yang, S. Yeganeh, S. R. Yost, Z.-Q. You, I. Y. Zhang, X. Zhang, Y. Zhao, B. R. Brooks, G. K. L. Chan, D. M. Chipman, C. J. Cramer, W. A. Goddard, M. S. Gordon, W. J. Hehre, A. Klamt, H. F. Schaefer, M. W. Schmidt, C. D. Sherrill, D. G. Truhlar, A. Warshel, X. Xu, A. Aspuru-Guzik, R. Baer, A. T. Bell, N. A. Besley, J.-D. Chai, A. Dreuw, B. D. Dunietz, T. R. Furlani, S. R. Gwaltney, C.-P. Hsu, Y. Jung, J. Kong, D. S. Lambrecht, W. Liang, C. Ochsenfeld, V. A. Rassolov, L. V. Slipchenko, J. E. Subotnik, T. Van Voorhis, J. M. Herbert, A. I. Krylov, P. M. W. Gill, M. HeadGordon, *Mol. Phys.* **2015**, *113*, 184–215.

[7] C. Y. Legault, CYLview2.0, 2020. http://www.cylview.org

[8] W. Humphrey, A. Dalke, K. Schulten, VMD – Visual Molecular Dynamics. *J. Mol. Graphics.* **1996**, *14*, 33–38.

[9] pASDI, https://github.com/grahamhaug/LarionovLabComputationalScripts/tree/main/pASDI.

[10] F. M. Bickelhaupt, K. N. Houk, *Angew. Chem., Int. Ed.* **2017**, *56*, 10070–10086.

[11] G. Luchini, J. V. Alegre-Requena, Y. Guan, I. Funes-Ardoiz, R. S. Paton, *F1000Research* **2020**, *9*, 291.

[12] See the supplemental information file for: L. De Angelis, G. C. Haug, G. Rivera, S. Biswas, A. Al-Sayyed, H. Arman, O. Larionov, M. P. Doyle. Site Reversal in Nucleophilic Addition to 1,2,3-Triazine 1-Oxides. *J. Am. Chem. Soc.* **2023**, 145, 13059–13068.

[13] T. Lu and F. Chen, *J. Comp. Chem.,* 2012, **33**, 580–592.

[14] For details of the calculations, see the supplemental information file for: Jin, S.; Haug, G. C.; Trevino, R.; Nguyen, V. D.; Arman, H. D.; Larionov, O. V.; Photoinduced C(sp^3)–H sulfination empowers the direct and chemoselective introduction of the sulfonyl group. *Chem. Sci*. **2021**, 12, 13914–13921.

[15] E. Caldeweyher, S. Ehlert, A. Hansen, H. Neugebauer, S. Spicher, C. Bannwarth, S. Grimme, *The Journal of Chemical Physics* **2019**, *150*, 154122.
